# Facile Access to Hindered Ethers via Photoinduced O–H Bond Insertions

**DOI:** 10.1021/acscentsci.5c00099

**Published:** 2025-04-23

**Authors:** Yu Zhang, Xinyu Han, Dong Li, Dinggang Wang, Jinxin Wang, Xin Luan, Shao-Fei Ni, Shoubhik Das, Wei-Dong Zhang

**Affiliations:** 1 Shanghai Frontiers Science Center for Chinese Medicine Chemical Biology, Institute of Interdisciplinary Integrative Medicine Research, 66322 Shanghai University of Traditional Chinese Medicine, No. 1200, Cailun Road, Shanghai 201203, China; 2 School of Chemistry and Chemical Engineering, Henan Normal University, Xinxiang, Henan 453007, People’s Republic of China; 3 Department of Chemistry, Key Laboratory for Preparation and Application of Ordered Structural Materials of Guangdong Province, 12386 Shantou University, Shantou 515063, China; 4 School of Pharmacy, Second Military Medical University, Shanghai 200433, China; 5 Department of Chemistry, 26523 University of Bayreuth, Bayreuth, 95447, Germany

## Abstract

The synthesis of the hindered and polyfluorinated dialkyl ethers poses challenges owing to the bulkiness of tertiary alcohols and the low nucleophilicity of polyfluorinated alcohols. Additionally, associated competitive side reactions always provide poor reactivities. Although certain strategies, such as electrocatalytic decarboxylation and hydroalkoxylation, have been explored, a straightforward method for obtaining ethers with structural diversity remains elusive. In this study, we have exploited the photoinduced approach that involves the *in situ* formation of singlet carbenes followed by O–H insertions to access the hindered and polyfluorinated ethers with congested or polyfluorinated alcohols. Moreover, other nucleophiles such as phenols, H_2_O, thiols, silanols, tributyltin hydride, *etc*., are also tolerable to obtain valuable products. The gram-scale synthesis of marketed drugs and the modification of complex molecules demonstrate the practicality of this approach. The detailed mechanistic studies have elucidated the key intermediates and reaction mechanism, which were distinct from traditional metal-carbenoid O–H insertions.

## Graphical abstract

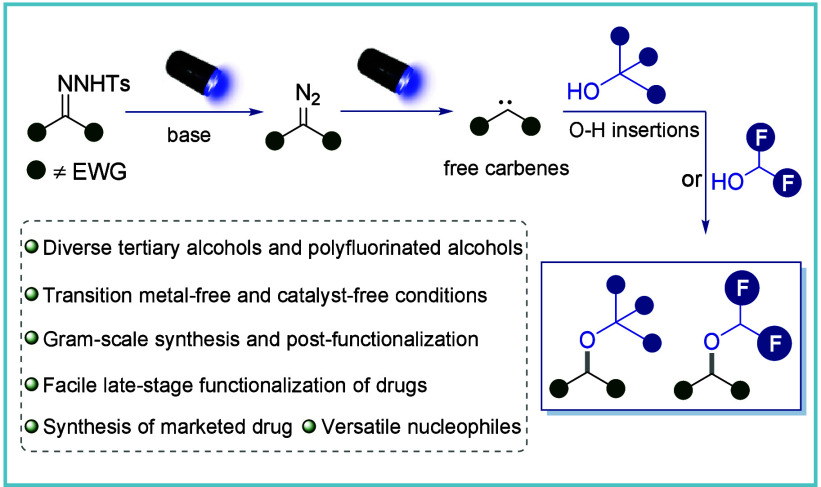

## Introduction

The facile synthesis of ethers has long been a long-standing task in drug design, as the incorporation of the ether group enhances the solubility and bioavailability.^1–3^ The hindered dialkyl ethers can aid in the escape-from-flatland of bioactive molecules and improve the drug–target interaction to promote the drug efficacy and selectivity.^4,5^ Moreover, the polyfluorinated dialkyl ethers exhibit the improved lipophilicity and metabolic stability compared to their nonfluorinated counterparts.^6–8^ Notably, the polyfluorinated ethers are integral for enhancing the performance of lithium-based batteries.^9^ Therefore, the practical and straightforward synthesis of hindered and polyfluorinated dialkyl ethers holds substantial significance in the pharmaceutical and chemical industries. Nowadays, the Williamson ether synthesis and Mitsunobu reactions have been widely recognized as reliable methods for accessing ethers, particularly primary dialkyl ethers.^10,11^ Recent decades have also seen the advancements in C–H activation,^12–15^ cyclopropane ring opening reactions,^16,17^ transition-metal catalysis,^18–24^ and other methodologies.^25^ Despite these improvements, the synthesis of the hindered dialkyl ethers remains a formidable challenge because of the bulkiness of reagents and competitive side reactions, such as halide elimination. Additionally, synthesizing the polyfluorinated ethers is inhibited by the specific properties such as their low nucleophilicity, high polarity, and acidity of polyfluorinated alcohols (Figure 1A).^26^

**Figure 1:**
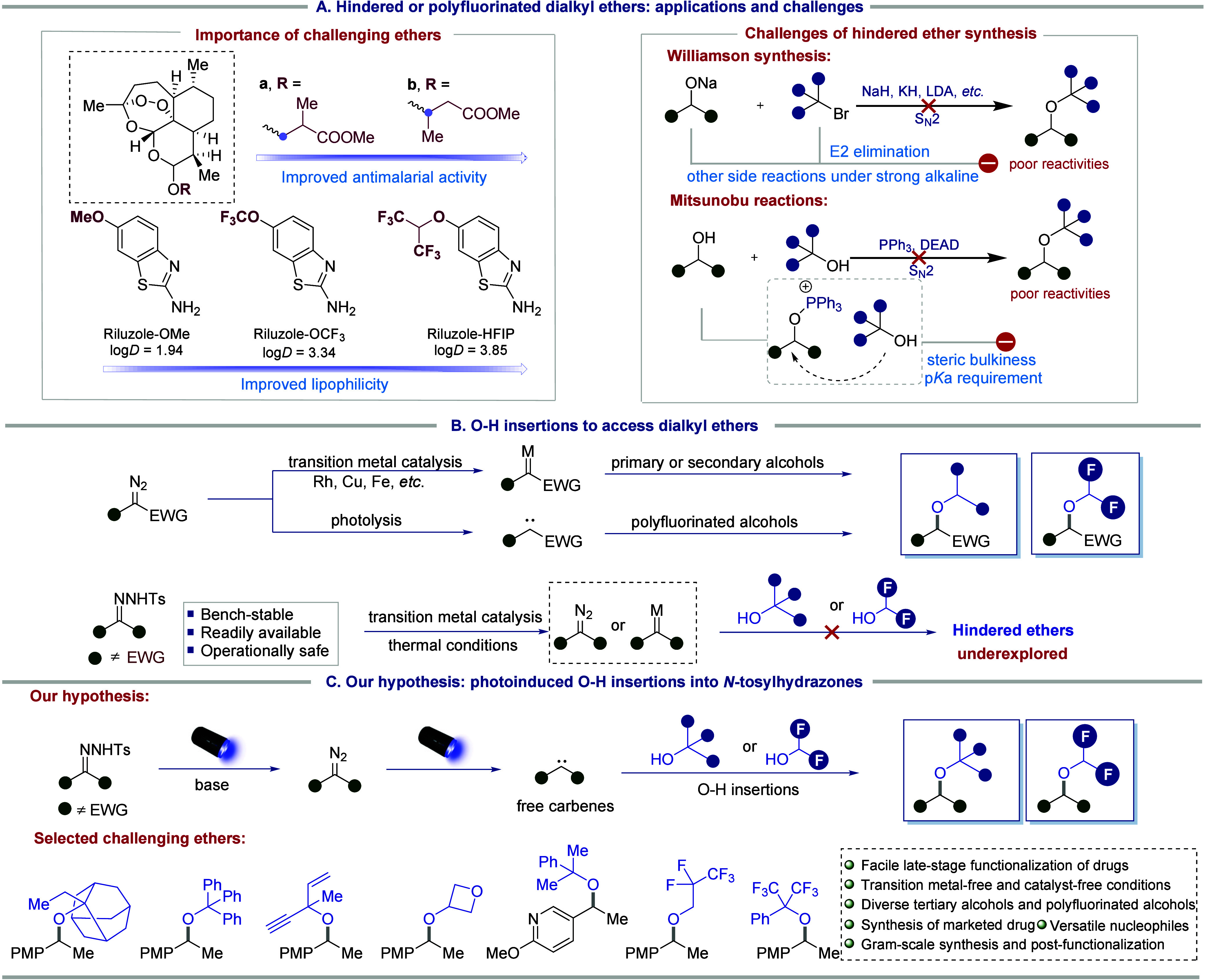
Background of hindered and polyfluorinated dialkyl ethers and our hypothesis.

To address these challenges, Baran et al. developed an effective electrochemical strategy in which the carbocations generated from carboxylic acids were efficiently captured by tertiary alcohols to produce the hindered dialkyl ethers.^27^ This method had a broad substrate scope, whereas carboxylic acid synthesis typically involved multiple steps. Alternative approaches, such as C–H functionalization, have been reported, while they show limited applicability to tertiary or polyfluorinated alcohols.^12–15,28^ Similarly, the hydroalkoxylation of alkenes using the cobalt catalysis can offer an elegant route to the hindered ethers, while it is restricted to simple tertiary alcohols.^29,30^ Another robust method to afford dialkyl ethers involves carbenoid O−H insertions, which necessitates the activation of diazo esters under transition-metal catalytic conditions to generate metal carbenes. However, the hindered alcohols are often impractical for this approach.^31,32^ Recently, Koenigs et al. demonstrated the application of diazo compounds to form ethers with the polyfluorinated alcohols under blue light irradiation, marking a significant advancement.^33,34^ Concurrently, Rovis et al. reported the photocatalytic O–H insertions into the α-stabilized diazo compounds but limited to primary alcohols.^35^ Despite their utility, diazo compounds as common carbene precursors are constrained by the structural limitations, instability, and susceptibility to competing reactions, necessitating diverse and readily available carbene precursors (Figure 1B).^36^

*N*-Tosylhydrazones have been recognized as significant alternatives to diazo compounds among various carbene precursors. They are widely used in the organic synthesis because of their safety, easy preparation from ketones, and structural diversity.^37–40^ Meanwhile, the photoinduced reactions^41–47^ have offered attractive opportunities for activating and transforming the carbene precursors,^40^ particularly *N*-tosylhydrazones,^48–51^ which traditionally relied on the transition metal catalysis or thermal conditions.^52,53^ Despite these advances, the O–H insertion into *N*-tosylhydrazones with the hindered or polyfluorinated alcohols remains challenging.^54^ We hypothesize that the photoinduced O–H insertions into *N*-tosylhydrazones could offer a viable solution to this issue, drawing from our extensive experience in the photochemical activation and transformation of these compounds.^52,55–58^ The photoinduced activation of *N*-tosylhydrazones initiates with the formation of an *in situ* donor/donor diazo intermediate, which further undergoes the O–H insertion either directly into the diazo compounds or into the free carbene following the nitrogen release. And the subsequent proton transfer yields the final product. Building on this method, the hindered alcohols and weakly nucleophilic polyfluorinated alcohols smoothly underwent the O–H insertions to produce the previously challenging ethers under photoinduced conditions (Figure 1C). In addition to alcohols, various nucleophiles, including phenols, H_2_O, thiols, silanols, and tributyltin hydride, were also compatible with this strategy. Furthermore, our system successfully incorporated the ether groups into various pharmaceutical and natural product analogs. We also demonstrated the gram-scale synthesis of the dopamine reuptake inhibitor vanoxerine and natural product derivatives, highlighting the potential of this system. At last, detailed experimental investigations and DFT studies illustrated the reaction mechanism, which was distinct from traditional metal-carbenoid O–H insertions.

## Results and Discussion

At the beginning of our investigation, hindered dialkyl ethers were synthesized using *N*-tosylhydrazone **1b** (derived from ketone **1a**) and the tertiary alcohol (**1c** 2-phenyl-2-propanol) as model substrates (Table 1). Following the systematic optimization, the desired hindered ether (**1d**) was obtained with a high yield of 85% under the irradiation of 40 W 427 nm Kessil lamps (**entry 1**). Alternative light sources, such as 456 and 390 nm Kessil lamps, were also tested but exhibited lower reactivity than the 427 nm Kessil lamps (**entries 2–3**). Several bases were subsequently evaluated for their impact on the reaction. However, Cs_2_CO_3_, K_2_CO_3_, and DBN were found to be less effective than DBU (**entries 4–6**). Further exploration revealed 1,4-dioxane as the optimal solvent for this system (**entries 7–8**, please see Table S1 for further optimizations). The control experiments confirmed the essential roles of both the base and light in the reaction (**entries 9–10**). Additionally, the thermal conditions were tested, revealing no product formation even at the elevated temperature of 100 °C, demonstrating the critical role of light in this method (**entry 11**).

**Table 1:**
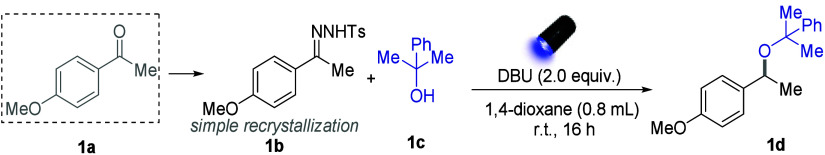
Optimization of Synthesizing Hindered Dialkyl Ethers

Entry	Deviation from standard conditions	Yield [%]^a^ ^,^ ^b^
1	none	85 (80)^c^
2	390 nm instead of 427 nm	78
3	456 nm instead of 427 nm	60
4	Cs_2_CO_3_ instead of DBU	59
5	K_2_CO_3_ instead of DBU	45
6	DBN instead of DBU	60
7	DCM instead of 1,4-dioxane	54
8	ACN instead of 1,4-dioxane	55
9	without light	N.R.
10	without DBU	N.R.
11	no light and heating to 100 °C	N.R.

a General reaction conditions: **1b** (0.2 mmol), **1c** (10.0 equiv), base (2.0 equiv), solvent (0.8 mL), 40 W 427 nm Kessil lamps, room temperature, 16 h.

b Yields were determined by ^1^H NMR analysis using 1,3,5-trimethoxybenzene as an internal standard.

c Isolated yields.

After determining the optimal reaction conditions, we successfully synthesized a wide range of hindered dialkyl ethers using various tertiary alcohols. Initially, the tertiary alkyl alcohols with different chain lengths were evaluated, all of which reacted smoothly, yielding the corresponding hindered ethers in 54–85% yield (Figure 2, **1d**–**5d**). Subsequently, the tertiary alcohols with multiple aryl rings, including highly congested triphenylmethanol, were suitable for hindered ether synthesis (**6d**–**8d**). Additionally, the tertiary alcohols bearing functional groups, such as chlorine, ketone, and ester, were accommodated in our system, forming the corresponding hindered ethers (**9d**–**11d**). The cyclic tertiary alcohols, such as 2-ethyl-2-adamantanol and 1-methylcyclopropan-1-ol, which had strong ring strain and significant steric hindrance, typically posed challenges for the etherification. However, our synthetic strategy successfully overcame these obstacles, enabling the etherification of cyclic tertiary alcohols containing three to six rings, including the adamantane ring (**12d**–**17d**). To broaden the applicability of our reaction, tertiary alcohols bearing reactive groups, such as pyridine, alkene, and alkyne, were evaluated and demonstrated excellent compatibility in our system, highlighting the versatility of the reaction (**18d**–**24d**). We further expanded the scope to include various secondary and primary alcohols with different functional groups, including amine (**27d**), cyclopropyl (**29d**), chlorine (**33d**), and alkene (**34d**) functionalities, all of which were well-tolerated (**25d**–**34d**). Notably, hydroxyl amino acid was also converted into a product in acceptable yield (**35d**). Moreover, phenol, water, and benzoic acid were also suitable nucleophiles, forming valuable aryl/alkyl ether, alcohol, and ester (**36d**–**38d**). In addition, the tributyltin hydride and tributylsilane were effective nucleophilic reagents for the generation of hindered products (**39d**, **40d**). The alkyl- or aryl-substituted thiols, commonly used in organic synthesis as building blocks, successfully delivered the highly valuable hindered thioethers (**41d**–**43d**). Similarly, various tertiary silanols were accommodated in our system, yielding the hindered silyl ethers in moderate to good yields (**44d**–**47d**). Considering that using lower amount of alcohols will be advantageous, especially for expensive alcohols, we selected several examples to investigate whether reducing the amount of alcohol (such as using 3.0 equiv of alcohols) could still achieve satisfactory results. And the results indicated that employing reduced amount of alcohols under this strategy still enabled hindered ethers in acceptable yields (**1d**, **2d**, **9d**). Given the unavailability of those hindered ethers, the moderate reactivities would also contribute to the expansion and application of the hindered ethers.

**Figure 2:**
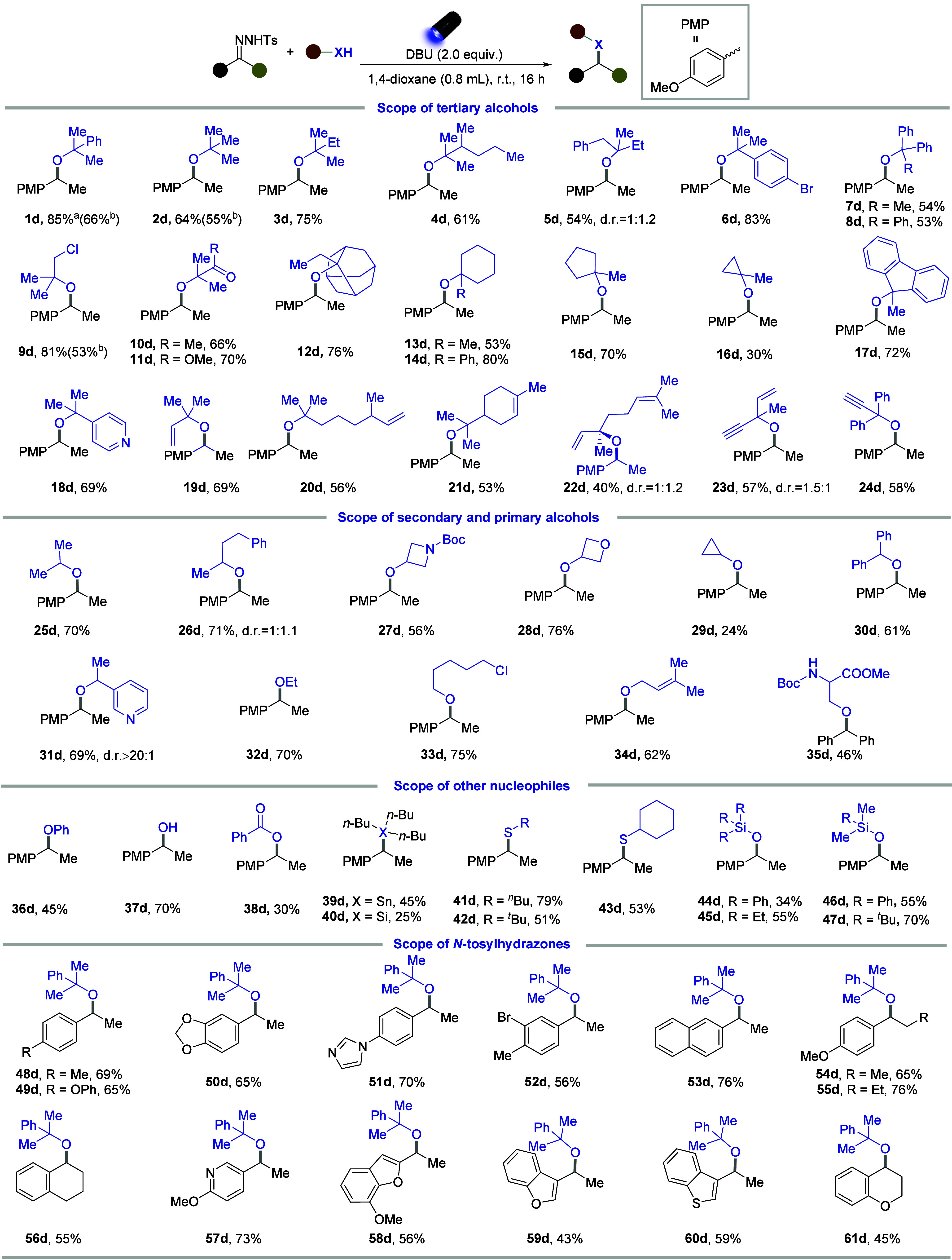
Substrate scope of hindered ether synthesis with different alcohols and *N*-tosylhydrazones. (a) General reaction conditions: *N*-tosylhydrazone (0.2 mmol), alcohol (10.0 equiv), DBU (2.0 equiv), 1,4-Dioxane (0.8 mL), 40 W 427 nm Kessil lamps, room temperature, 16 h. (b) 3.0 equiv of alcohol was used.

We further evaluated the scope of *N*-tosylhydrazones with the electron-donating or electron-withdrawing substituents, which yielded the corresponding hindered ethers in moderate to high yields (**48d**–**52d**). Expanding the versatility of *N*-tosylhydrazones, those derived from various aryl alkyl ketones with different chain lengths were viable in our system (**54d**–**55d**). Additionally, the *N*-tosylhydrazone derived from the benzocyclic ketone reacted effectively to produce the ether product (**56d**). The heteroaromatic compounds, such as pyridine, benzofuran, thianaphthene, and benzopyrane, have been known for their pharmaceutical potential.^59^ Therefore, we explored the synthesis of the hindered ethers using *N*-tosylhydrazones bearing these heteroarenes, achieving acceptable yields of the corresponding hindered ethers and indicating the broad diversity of this approach (**57d**–**61d**).

Intrigued by these results, we shifted our focus to the synthesis of polyfluorinated alkyl ethers. Initially, 1,1,1,3,3,3-hexafluoro-2-propanol (HFIP), as the alcohol partner, yielded the desired product in only a 37% yield under standard conditions. To enhance the yield, we made slight modifications: replacing the base with K_2_CO_3_ and using 390 nm Kessil lamps (please see Table S2, Supporting Information). This adjustment led to the synthesis of polyfluorinated alkyl ethers in an excellent yield (90%). Using these optimized conditions, we explored a broad range of polyfluorinated alcohols (Figure 3). The etherification between *N*-tosylhydrazones (**1b**) and primary polyfluorinated alcohols, such as 2,2,2-trifluoroethanol, pentafluoro-1-propanol, and 2,3,4,5,6-pentafluorobenzyl alcohol, demonstrated moderate to excellent reactivities (**62d**–**69d**). Furthermore, the secondary polyfluorinated alcohols, such as 1-phenyl-trifluoroethanol, were compatible with producing the desired ether (**70d**). Inspired by this compatibility, various tertiary polyfluorinated alcohols, including 2-trifluoromethyl-2-propanol, hexafluoro-2-methylisopropanol, and 1,1,1,3,3,3-hexafluoro-2-phenyl-2-propanol, were investigated and suitable for coupling in our system (**71d**–**73d**). In this context, the decreased nucleophilicity caused by an increased number of fluorine atoms makes the construction of ethers more challenging. Therefore, some alcohols were used in over stoichiometric quantities (10–20 equiv) to achieve the good reactivities. But lower amount of the alcohols was also explored and we found that reducing the quantity to 5.0 equiv could also form the polyfluorinated dialkyl ethers, whereas the reactivities were relatively limited (**65d**, **67d**, **70d**, **71d**, **74d**).

**Figure 3:**
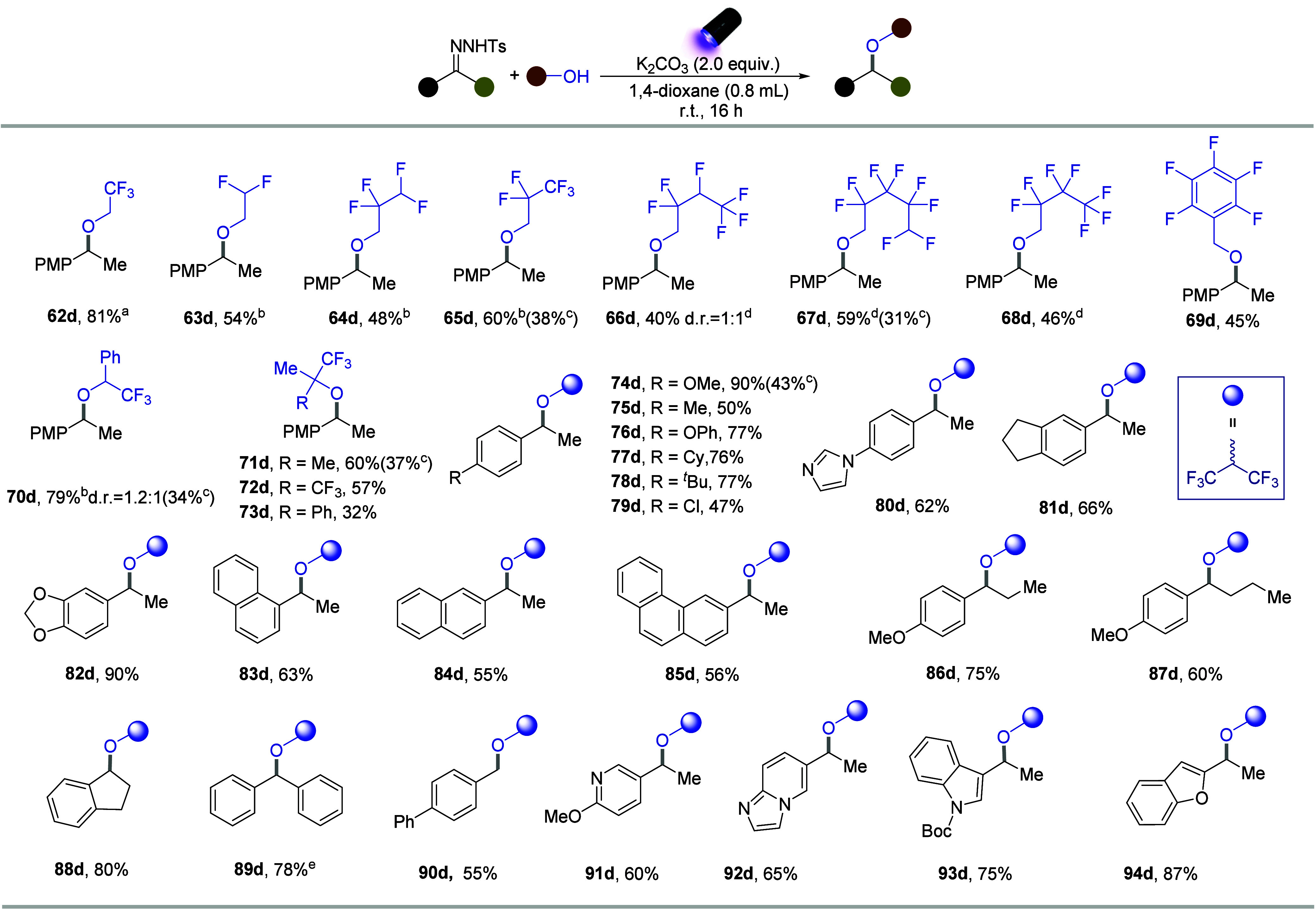
Substrate scope of polyfluorinated ether synthesis with various polyfluorinated alcohols and *N*-tosylhydrazones. (a) General reaction conditions: *N*-tosylhydrazone (0.2 mmol), K_2_CO_3_(2.0 equiv), alcohol (10.0 equiv), 1,4-dioxane (0.8 mL), 40 W 390 nm Kessil lamps, rt 16 h. (b) 20.0 equiv of alcohol was used. (c) 5.0 equiv of alcohol was used. (d) Alcohol was the solvent. (e) 3.5 equiv of alcohol was used.

We further explored the scope of *N*-tosylhydrazones to assess the generality of this strategy and investigated the tolerance of *N*-tosylhydrazones to various electron-withdrawing or electron-donating groups (**74d**–**82d**). The mono- and multisubstituted *N*-tosylhydrazones derived from acetophenones yielded the desired polyfluorinated ethers in moderate to excellent yields. The *N*-tosylhydrazones containing naphthalenes or phenanthrenes with different substituents also provided the desired products (**83d**–**85d**). Furthermore, the *N*-tosylhydrazones with varying alkyl chain lengths exhibited moderate to good reactivities (**86d, 87d**). The *N*-tosylhydrazone derived from 1-indanone, provided the corresponding ether in excellent yield (**88d**). In addition, the *N*-tosylhydrazones derived from benzophenone and benzaldehyde demonstrated good reactivities (**89d, 90d**). Notably, when using benzophenone-derived *N*-tosylhydrazone (**89d**), the amount of HFIP can be reduced to 3.5 equiv, indicating that the aryl–aryl diazo intermediates were susceptible to O–H insertions.^34^ Furthermore, the system tolerated *N*-tosylhydrazones substituted with heteroarenes such as pyridine, imidazo­[1,2-*a*]­pyridine, indole, and benzofuran (**91d**–**94d**), demonstrating the broad substrate scope of this strategy.

After the successful construction of diverse hindered ethers, a systematic discussion about the substituent tolerance was conducted. Experimental results showed that alcohols bearing functional groups, such as halides (-I, -Cl, -F), ketones, esters, alkenes, alkynes, and oxetanes groups exhibited good tolerance in this strategy, indicating that these functional groups can coexist in the reaction environment without significantly affecting the reaction efficiency. *N*-Protected serine was also a suitable substrate. In addition, other nucleophilic substrates, such as phenols, water, acids, Sn, Si, thiols, and silanols, were accommodated in our system. Regarding the aryl/alkyl *N*-tosylhydrazones, most of the substituents were accommodated. *N*-tosylhydrazones bearing different heteroarenes also reacted well to furnish the hindered ethers. However, it should be noted that the aryl/alkyl *N*-tosylhydrazones bearing strong EWGs might be challenging (please see Figure S3, Supporting Information), probably due to the inefficiency to activate under the irradiation at 390 nm. What’s more, the application and utilization of dialkyl *N*-tosylhydrazones in O–H insertions remain challenging. To address this issue, we conducted extensive screening of dialkyl *N*-tosylhydrazones under photoinduced conditions. Unfortunately, despite our efforts, we have not yet achieved successful O–H insertion using dialkyl *N*-tosylhydrazones (please see Figure S3, Supporting Information). We speculate that the difficulty may arise from the combined effects of the instability of dialkyl carbenes, the weak nucleophilicity of alcohols, and the steric hindrance. We are very regretful about this outcome; but we believe current research progress has already promoted the practical synthesis of hindered ethers and the O–H insertion reaction of *N*-tosylhydrazones.

Following the successful construction of hindered and polyfluorinated dialkyl ethers, we aimed to validate the practicality of this method. To achieve this, we successfully incorporated the challenging ether motifs into drugs and natural products, such as sertraline, flavanones, cetirizine, salicylic acid, and ketoprofen (Figure 4, **95d–102d**), obtaining satisfactory yields ranging from 36% to 76% within 16 h of reaction time. Given the prevalence of tertiary alcohols in nature, we applied a range of naturally occurring tertiary alcohols, including lercanidipine side chain, α-terpineol, cedrol, stiripentol, d-menthol, cholesterol, diacetonefructose, and geraniol, in the synthesis of the hindered and polyfluorinated ethers (**103d**–**112d**). This indicated the robustness of our strategy for synthesizing challenging ethers.

**Figure 4:**
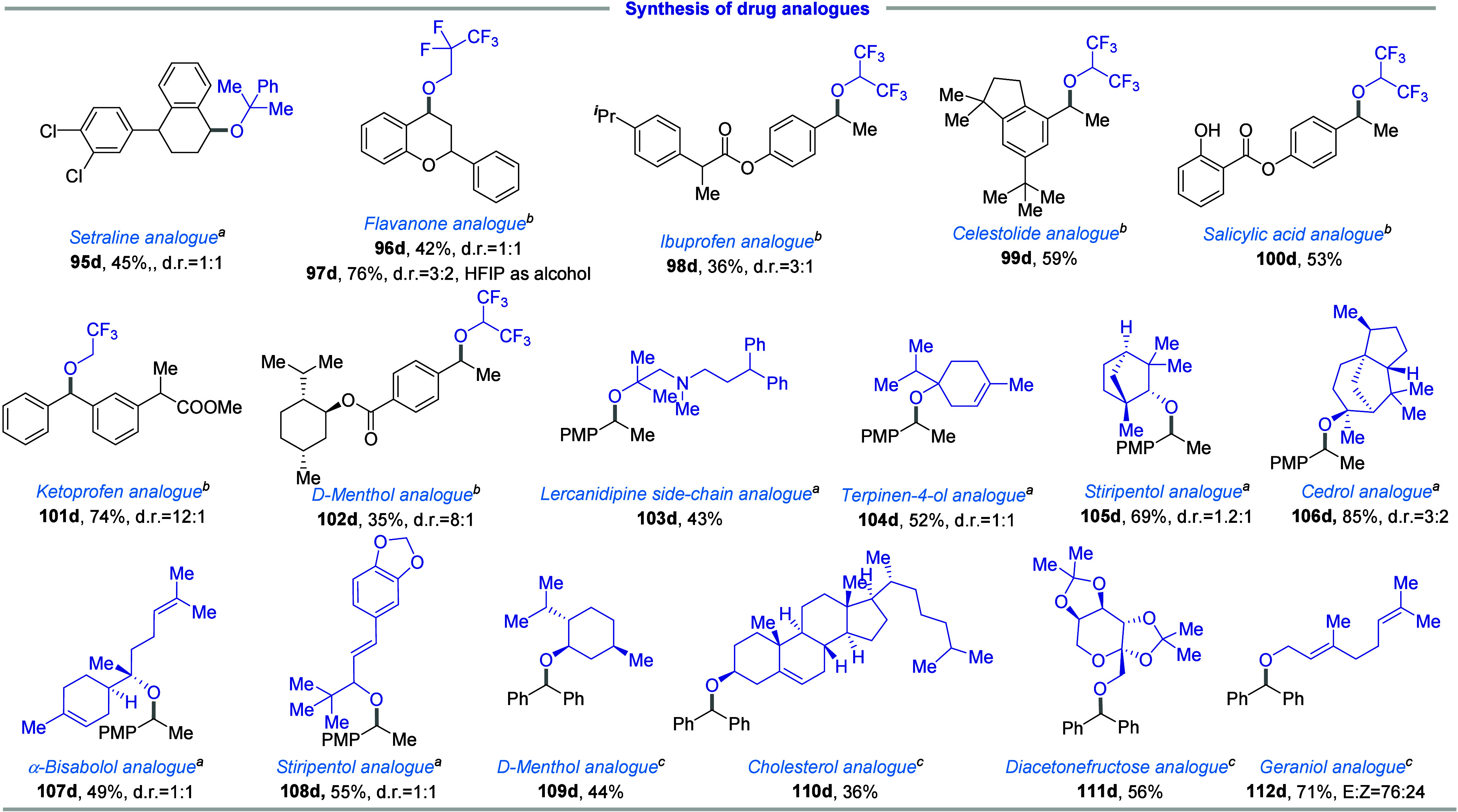
Facile etherification of natural products and pharmaceuticals. (a) *N*-tosylhydrazone (0.2 mmol), DBU (2.0 equiv), alcohol (10.0 equiv), 1,4-dioxane (0.8 mL), 40 W 427 nm Kessil lamps, rt 16 h. (b) *N*-tosylhydrazone (0.2 mmol), K_2_CO_3_(2.0 equiv), alcohol (10.0 equiv), 1,4-dioxane (0.8 mL), 40 W 390 nm Kessil lamps, rt 16 h. (c) **b** (0.2 mmol), K_2_CO_3_(2.0 equiv), **c** (3.5 equiv), 1,4-dioxane (0.8 mL), 40 W 390 nm Kessil lamps, r.t. 16 h.

To further demonstrate the practical application of this strategy in drug discovery, we conducted a rapid and modular derivatization of the marketed drug fenofibrate, resulting in 13 drug analogues with challenging ether groups (Figure 5A). Fenofibrate has been widely used to reduce triglycerides, uric acid levels, and treat type 2 diabetes and metabolic syndrome. Importantly, the repurposing marketed drugs and their derivatives can be a prevalent strategy in drug development because of their safety, cost-effectiveness, and shorter R&D cycles. Our approach proposed a convenient method for quickly modifying carbonyl functional group-based drugs, facilitating the discovery of new therapeutic applications for fenofibrate. Additionally, we efficiently synthesized another important marketed drug, the dopamine reuptake inhibitor, vanoxerine, using this sustainable strategy. The key intermediate (**126d**) for vanoxerine synthesis was obtained on the gram scale using this catalyst-free strategy at room temperature,^60^ yielding 65% isolated yields (Figure 5B). Additionally, the polyfluorinated ether **74d** was successfully synthesized on a gram scale, and importantly, the postfunctionalization of **74d** yielded another unprecedented diether. To further investigate the value of the synthesized polyfluorinated ethers, we prepared a gram-scale derivative of flavanone bearing a hexafluoroisopropoxy group by using this method (Figure 5C). This derivative was efficiently converted into other valuable compounds by using 1,1-diaryl scaffolds. Finally, the one-pot synthesis of hindered ethers directly from ketones was developed, offering a straightforward route despite slightly lower yields compared to the use of *N*-tosylhydrazones (Figure 5D).

**Figure 5:**
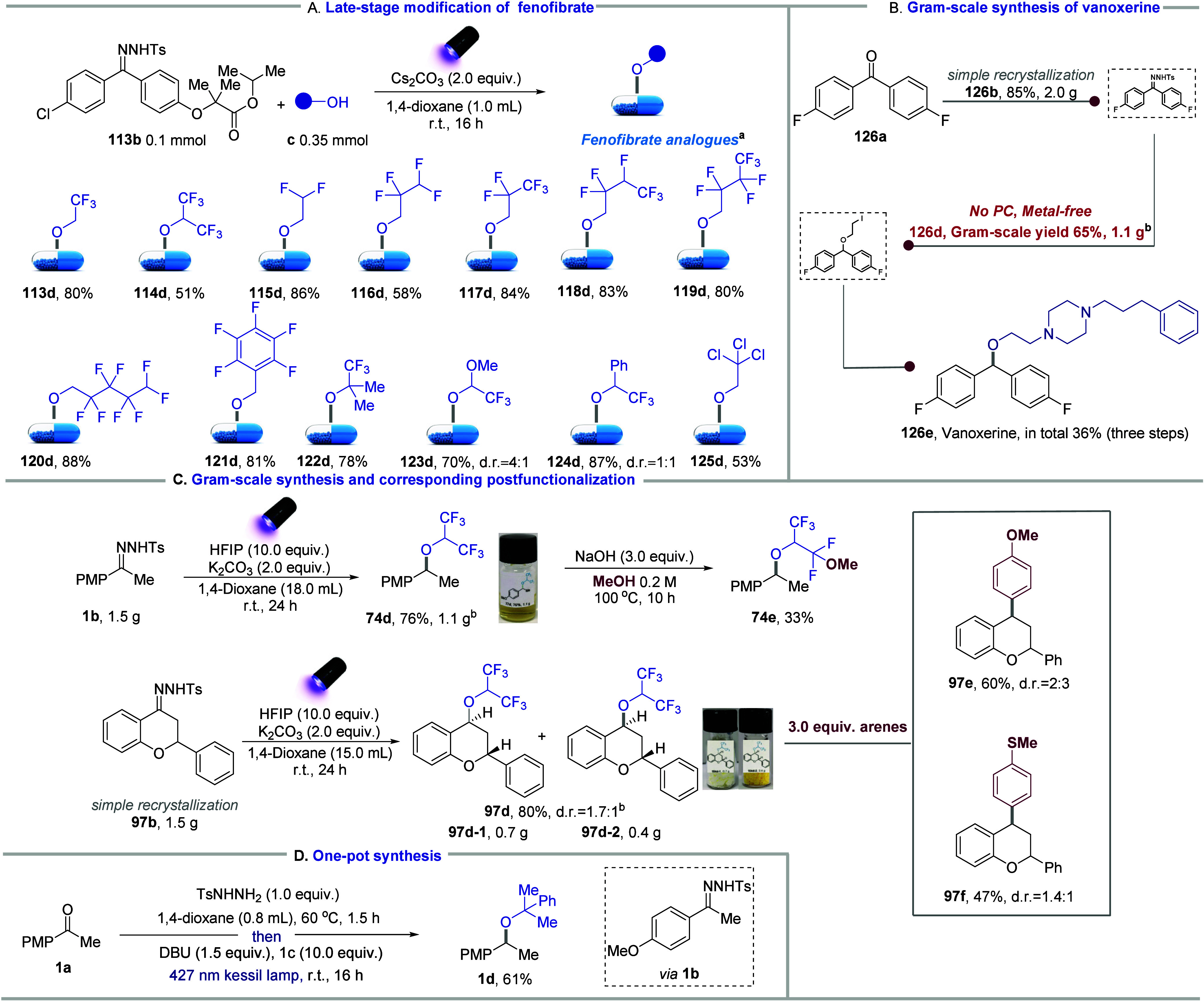
Practical application of hindered and polyfluoronated dialkyl ethers. (a) General reaction conditions: *N*-tosylhydrazone (0.2 mmol), alcohol (3.5 equiv), K_2_CO_3_(2.0 equiv), 1,4-dioxane (1.0 mL), 40 W 390 nm kessil lamps, r.t., 16 h. (b) *N*-Tosylhydrazone (1.5 g, 3.9 mmol), DBU (2.0 equiv), alcohol (10.0 equiv), 1,4-dioxane (20.0 mL), 40 W 427 nm kessil lamps, rt, 24 h.

Finally, the mechanism of the reaction was investigated. Our initial studies, 16 h was necessary to achieve the optimal yield (Figure 6A). Furthermore, the on–off experiments confirmed that the presence of light was essential for product formation (Figure 6B). To elucidate the role of light in our system, the ultraviolet/visible (UV/vis) absorption spectra were obtained, revealing a clear red (bathochromic) shift between the organic base DBU and the *N*-tosylhydrazone anion, with the visible light absorption extending to 350–450 nm, indicative of the noncovalent complex formation (Figure 6C).^55^ The radical quenching experiments confirmed the absence of radical processes in the system, indicating that the triplet carbene species did not play a significant role (Figure 6D).^52^ Subsequent carbene trapping experiments with styrene and alkynes demonstrated the formation of cyclopropane, and cyclopropene confirming the presence of singlet carbene species in our system (Figure 6E).^61,62^ Labeling experiments suggested that proton transfer was involved in this reaction (Figure 6G). To further elucidate the mechanism, DFT studies were conducted (see Figure S9 and Figure S10 for Supporting Information). It showed that the noncovalent complex was formed between the excited *N*-tosylhydrazone anion and base, which furnished the diazo intermediate after light irradiation. The diazo intermediate further formed the singlet carbene species *via* photolysis, which subsequently underwent the stepwise or concerted transition states TS3 and TS3′ to afford the final products. It should be noted that the stepwise barrier transition state TS3 is observed for the polyfluorinated alcohols, while the three-membered-ring transition state TS3′ was located when tertiary alcohols were used. There is the other pathway *via* the formation of ylide species in previous metal-carbenoid O–H insertions.^32,63^ However, this process is not located, probably due to the low nucleophilicity or bulkiness of (polyfluorinated)­alcohols. Based on the DFT calculation and our mechanistic studies, we supposed that concerted insertion of an O–H atom *via* singlet carbene was the favorable route to obtain challenging ethers (Figure 6H).

**Figure 6:**
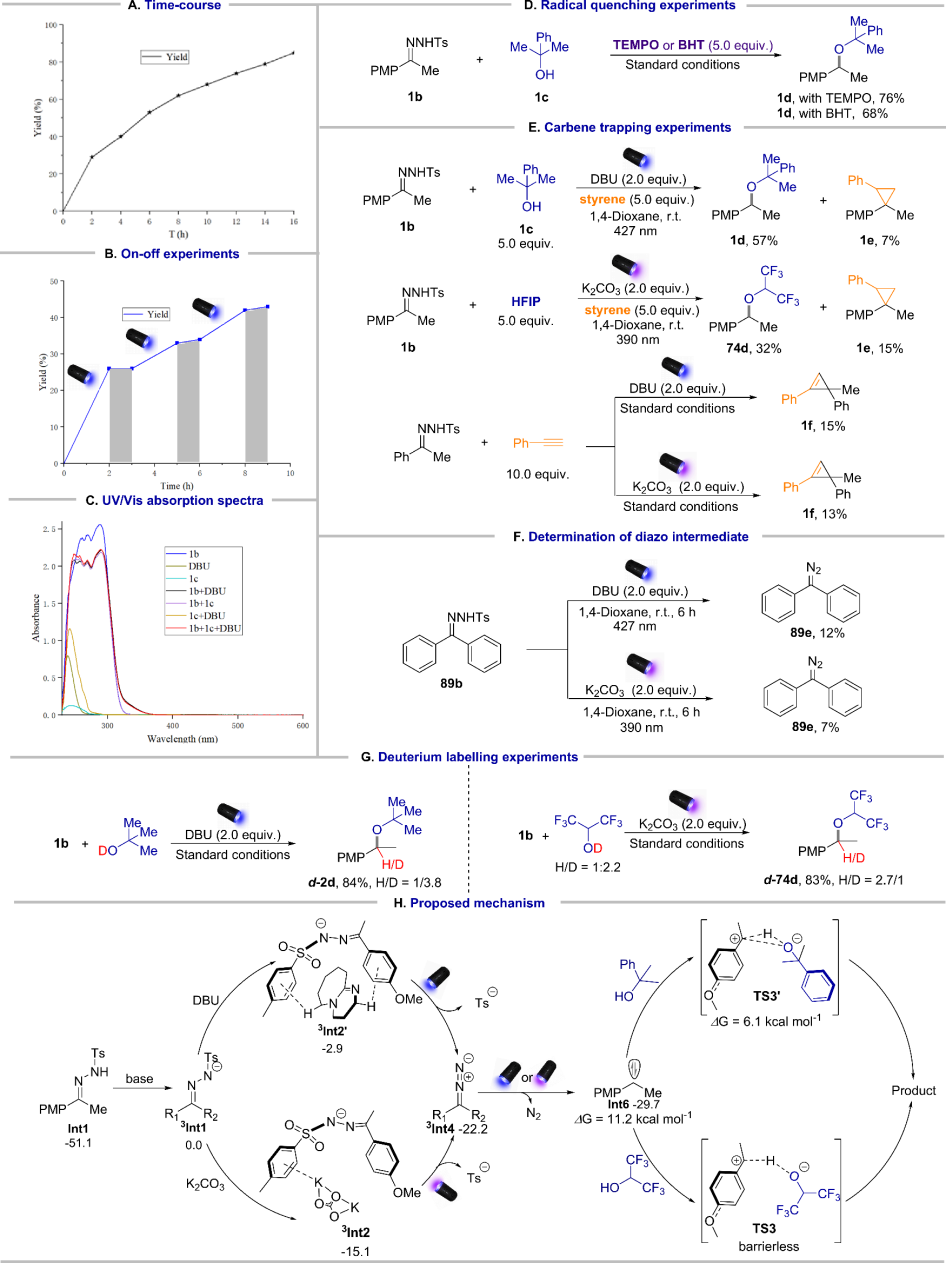
Mechanistic studies and the proposed mechanism.

## Conclusion

We demonstrated the light-induced synthesis of hindered and polyfluorinated dialkyl ethers using congested tertiary alcohols and polyfluorinated alcohols. A wide range of tertiary alcohols bearing active groups or bulky structures were successfully accommodated in this system. Moreover, other nucleophilic coupling partners, including water, benzoic acids, thiols, silanols, and tributyltin hydride, were compatible, highlighting the broad tolerance of this strategy. The gram-scale synthesis and effective incorporation of challenging ether motifs into drug analogues underscore the practical utility of this approach. Moreover, the rapid and modular derivatization of a marketed drug exhibited its potential in expanding chemical diversity to aid drug discovery efforts. Finally, mechanistic investigations and DFT studies revealed that the major process involved the photoinduced formation of singlet carbenes followed by metal-free O–H insertion.

## Supplementary Material



## Notes

Experimental details, characterization data, and spectra for the compounds of the synthesized compounds (PDF)
