# Supplementary material for: Facile Access to Hindered Ethers via Photoinduced O–H Bond Insertions
Source: ACS Cent Sci. 2025 Apr 23;11(5):742–52. doi: 10.1021/acscentsci.5c00099 (PMC12123463; doi:10.1021/acscentsci.5c00099)
Supplement: Supplementary file 1 [file oc5c00099_si_001.pdf]

Supporting information

Facile Access to Hindered Ethers *via* Photoinduced O-H Bond Insertions

Yu Zhang,[1,2†] Xinyu Han,[1†] Dong Li,[3†] Dinggang Wang,[4] Jinxin Wang,[4] Xin Luan,[1] Shao-Fei Ni,[3*] Shoubhik Das,[5*] Wei-Dong Zhang[1,2,4*]

1. Shanghai Frontiers Science Center for Chinese Medicine Chemical Biology, Institute of Interdisciplinary Integrative Medicine Research, Shanghai University of Traditional Chinese Medicine, No. 1200, Cailun Road, Shanghai 201203, China
2. School of Chemistry and Chemical Engineering, Henan Normal University, Xinxiang, Henan 453007, People's Republic of China
3. Department of Chemistry, Key Laboratory for Preparation and Application of Ordered Structural Materials of Guangdong Province, Shantou University, Shantou 515063, China
4. School of Pharmacy, Second Military Medical University, Shanghai 200433, China
5. Department of Chemistry, University of Bayreuth, Bayreuth, 95447, Germany

[*]Corresponding authors. *E-mail addresses:* sfni@stu.edu.cn (S. F. Ni), Shoubhik.Das@uni-bayreuth.de (S. Das), wdzhangy@hotmail.com (W. D. Zhang).

1 Materials and methods S3
2 Setup for photochemical reactions S4
3 Optimization of reaction conditions S5
4 General procedures for the synthesis of starting materials and products S8
5 Mechanistic studies S12
6 Characterization data of synthesized hindered ethers and polyfluorinated ethers S30
7 Characterization data of synthesized starting material S100
8 Cartesian coordinates of the optimized structures S102
9 Spectra of hindered ethers and polyfluorinated ethers S121
10 Partial spectra of synthesized starting material S278
11 References S281

1 Materials and methods

All reagents and solvents were purchased from certified chemical vendors and used without prior purification. Gases were purchased in high-pressured cylinders (200 bar) and converted to the desired pressure by means of a pressure regulator.

^{1}H NMR (500 MHz), ^{13}C NMR (126 MHz) and ^{19}F NMR (376 MHz) spectra were recorded on a Bruker Avance 500 MHz NMR spectrometer with $CDCl_3$, D_2O or DMSO-d_6 as solvent. The yield was determined by ^{1}H NMR using the 1,3,5-trimethoxybenzene as the internal standard. The spectra were calibrated by using residual undeuterated solvents (for ^{1}H NMR) and deuterated solvents (for ^{13}C NMR) as internal references: undeuterated chloroform (δ_H=7.26 ppm) and $CDCl_3$ (δ_C=77.16 ppm); undeuterated D_2O (δ_H=4.79 ppm); undeuterated DMSO-d_6 (δ_H=2.50 ppm) and DMSO-d_6 (δ_C=39.52 ppm). Chemical shifts (δ) were expressed in ppm and coupling constants (*J*) in Hertz (Hz). Splitting patterns are reported as: s (singlet), d (doublet), t (triplet), q (quadruplet), p (pentuplet), dd (doublet of doublet), dt (doublet of triplet), m (multiplet) or combinations thereof. Integration of the signals is presented as the number of hydrogen atoms. The assignments of individual NMR signals were based on additional 2D NMR experiments (NOE). High-resolution mass spectra (HRMS) were recorded on an Agilent MSD-Trap-XCT or Q-Tof micro mass spectrometer. Kessil lamps were purchased from Tansoole, with precise wavelengths (427 nm or 390 nm). Ultraviolet-visible absorption experiments were performed using a Hitachi U-2910 spectrophotometer.

Thin layer chromatography (TLC) was performed using a petroleum ether/ethyl acetate (EtOAc) solvent system as mobile phase and using MilliporeSigma glass TLC plates (silica gel 60 coated with F_{254}, 250 μm) and spots were visualized using UV light (254 nm). SiliaFlash® P60 silica gel (particle size: 40-63 μm, pore size: 60 Å) was used for flash column chromatography and petroleum ether/EtOAc solvent system was used as mobile phase.

2 Setup for photochemical reactions

The reaction setups are depicted in **Figure S1 and Figure S2**. The reaction setup consists of 4 commercially available Kessil lamps which were purchased from Tansoole, with precise wavelengths (427 nm or 390 nm), cooling of the setup was performed by two commercially available fans to keep the temperature around 30 °C. Magnetic stirring was performed at 500 rpm.

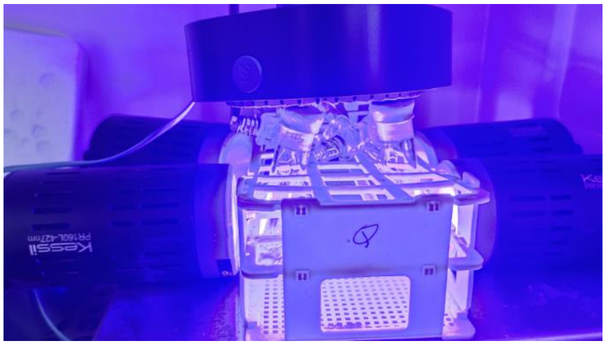

Figure S1. Photochemical reaction setup of 427 nm Kessil Lamps.

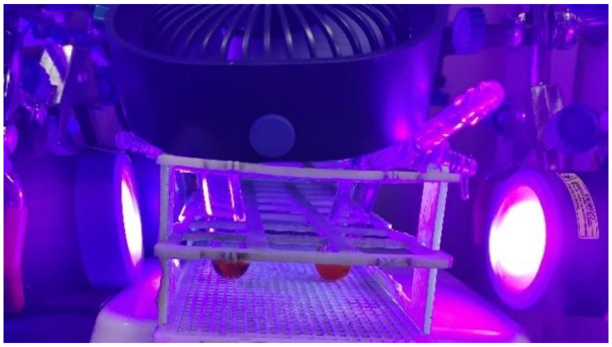

Figure S2. Photochemical reaction setup of 390 nm Kessil Lamps.

3 Optimization of reaction conditions

3.1 Optimization of synthesizing hindered dialkyl ethers

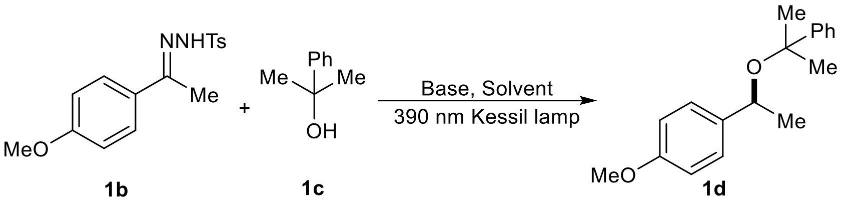

Entry[a]	1c (eq.)	Base (eq.)	Solvent	Wavelength	Yield (%)[b]
1	3.5 eq.	K_2CO_3 2.0 eq.	1,4-Dioxane	390 nm	28
2	10.0 eq.	K_2CO_3 2.0 eq.	1,4-Dioxane	390 nm	45
3	10.0 eq.	DBU 2.0 eq.	1,4-Dioxane	390 nm	78
4	10.0 eq.	DBN 2.0 eq.	1,4-Dioxane	390 nm	60
5	10.0 eq.	DBU 1.5 eq.	1,4-Dioxane	390 nm	68
6	10.0 eq.	DBU 3.0 eq.	1,4-Dioxane	390 nm	68
7	8.0 eq.	DBU 2.0 eq.	1,4-Dioxane	390 nm	65
8	5.0 eq.	DBU 2.0 eq.	1,4-Dioxane	390 nm	35
9	10.0 eq.	DBU 2.0 eq.	1,4-Dioxane	427 nm	85
10[c]	10.0 eq.	DBU 2.0 eq.	1,4-Dioxane	427 nm	79
11	10.0 eq.	DBU 2.0 eq.	1,4-Dioxane	456 nm	75
12	3.0 eq.	DBU 2.0 eq.	1,4-Dioxane	427 nm	66
13	10.0 eq.	DBU 2.0 eq.	Toluene	427 nm	74
14	10.0 eq.	DBU 2.0 eq.	EA	427 nm	80
15	10.0 eq.	DBU 2.0 eq.	DCM	427 nm	72
16	10.0 eq.	DBU 2.0 eq.	1,4-Dioxane	-	N.R.
17	10.0 eq.	-	1,4-Dioxane	427 nm	N.R.
18[d]	10.0 eq.	DBU 2.0 eq.	1,4-Dioxane	-	N.R.[e]

Table S1: [a] General reaction conditions: **1b** (0.2 mmol), **1c**, base, solvent (0.8 mL), 40 W 427 nm Kessil lamps, room temperature, 16 h. [b] Yields were determined by ^1H NMR analysis using 1,3,5-trimethoxybenzene as an internal standard. [c] **1b** (0.2 mmol), **1c** (10.0 equiv.), DBU (2.0 equiv.), 1,4-Dioxane (0.8 mL), 40 W 427 nm Kessil lamps, room temperature, 14 h. [d] No light and heating to 100 °C. [e] N.R. = no reaction.

3.2 Optimization of synthesizing polyfluorinated ethers

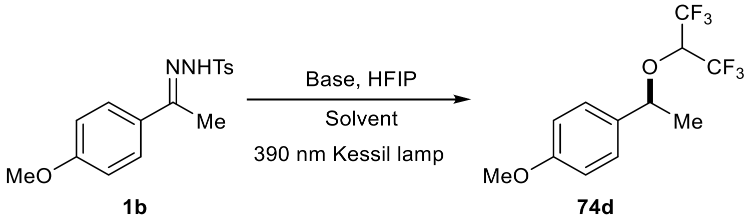

Entry[a]	HFIP	Solvent (mL)	Base (eq.)	Wavelength	T (h)	Yield (%)[b]
1	0.5 mL	1,4-Dioxane 0.5	K_2CO_3 2.0	390 nm	10	45
2	0.5 mL	1,4-Dioxane 0.5	K_2CO_3 2.0	390 nm	16	81
3	0.3 mL	1,4-Dioxane 0.7	K_2CO_3 2.0	390 nm	16	86
4	5.0 eq.	1,4-Dioxane 0.8	K_2CO_3 2.0	390 nm	16	43
5	10.0 eq.	1,4-Dioxane 0.8	K_2CO_3 2.0	390 nm	16	90
6	10.0 eq.	1,4-Dioxane 0.8	Cs_2CO_3 2.0	390 nm	16	85
7	10.0 eq.	1,4-Dioxane 0.8	$NaHCO_3$ 2.0	390 nm	16	N.R.
8	10.0 eq.	1,4-Dioxane 0.8	DBU 2.0	390 nm	16	37
9	10.0 eq.	1,4-Dioxane 0.8	K_2CO_3 1.5	390 nm	16	70
10	10.0 eq.	1,4-Dioxane 0.8	K_2CO_3 2.5	390 nm	16	70
11[c]	10.0 eq.	1,4-Dioxane 0.8	K_2CO_3 2.0	390 nm	14	79
12	10.0 eq.	EA 0.8	K_2CO_3 2.0	390 nm	16	69
13	10.0 eq.	DCM 0.8	K_2CO_3 2.0	390 nm	16	51
14	10.0 eq.	1,4-Dioxane 0.8	K_2CO_3 2.0	427 nm	16	77
15	10.0 eq.	1,4-Dioxane 0.8	K_2CO_3 2.0	456 nm	16	20
16	10.0 eq.	1,4-Dioxane 0.8	-		16	N.R.
17	10.0 eq.	1,4-Dioxane 0.8	K_2CO_3 2.0	-	16	N.R.
18[d]	10.0 eq.	1,4-Dioxane 0.8	K_2CO_3 2.0	-	16	N.R.[e]

Table S2: [a] General reaction conditions: **1b** (0.2 mmol), HFIP, base, solvent, 40 W 390 nm Kessil lamps, room temperature, 16 h. [b] Yields were determined by ^{1}H NMR analysis using 1,3,5-trimethoxybenzene as an internal standard. [c] **1b** (0.2 mmol), HFIP (10.0 equiv.), K_2CO_3 (2.0 equiv.), 1,4-Dioxane (0.8 mL), 40 W 390 nm Kessil lamps, room temperature, 14 h. [d] No light and heating to 70 °C. [e] N.R. = no reaction.

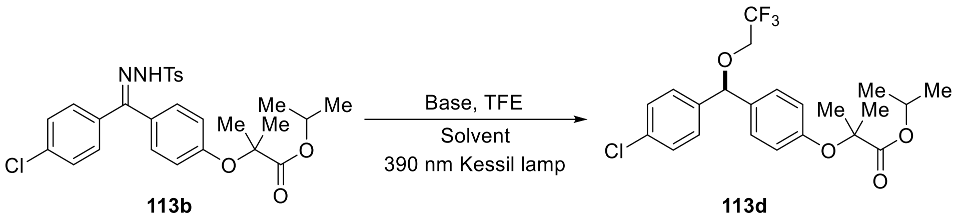

Entry[a]	TFE	Solvent	Base (eq.)	T (h)	Yield (%)[b]
1	3.5 eq.	1,4-Dioxane 1.0 mL	K_2CO_3 2.0 eq.	10	40
2	3.5 eq.	1,4-Dioxane 1.0 mL	K_2CO_3 2.0 eq.	16	64
3	3.5 eq.	DCM 1.0 mL	K_2CO_3 2.0 eq.	16	21
4	3.5 eq.	Acetone 1.0 mL	K_2CO_3 2.0 eq.	16	36
5	3.5 eq.	EA 1.0 mL	K_2CO_3 2.0 eq.	16	41
6	3.5 eq.	ACN 1.0 mL	K_2CO_3 2.0 eq.	16	38
7	3.5 eq.	1,4-Dioxane 1.0 mL	Cs_2CO_3 2.0 eq.	16	83
8	3.5 eq.	1,4-Dioxane 1.0 mL	$NaHCO_3$ 2.0 eq.	16	15
9	3.5 eq.	1,4-Dioxane 1.0 mL	Na_2CO_3 2.0 eq.	16	25
10	4.0 eq.	1,4-Dioxane 1.0 mL	Cs_2CO_3 2.0 eq.	16	79
11	3.0 eq.	1,4-Dioxane 1.0 mL	Cs_2CO_3 2.0 eq.	16	77
12	3.5 eq.	1,4-Dioxane 1.0 mL	Cs_2CO_3 1.5 eq.	16	81
13	3.5 eq.	1,4-Dioxane 1.0 mL	Cs_2CO_3 2.5 eq.	16	80
14	3.5 eq.	1,4-Dioxane 1.0 mL	-	16	N.R.
15[c]	3.5 eq.	1,4-Dioxane 1.0 mL	K_2CO_3 2.0	16	N.R.[d]

Table S3: [a] General reaction conditions: **1b** (0.2 mmol), TFE, base, solvent, 40 W 390 nm Kessil lamps, room temperature, 16 h. [b] Yields were determined by ^{1}H NMR analysis using 1,3,5-trimethoxybenzene as an internal standard. [c] No light. [d] N.R. = no reaction.

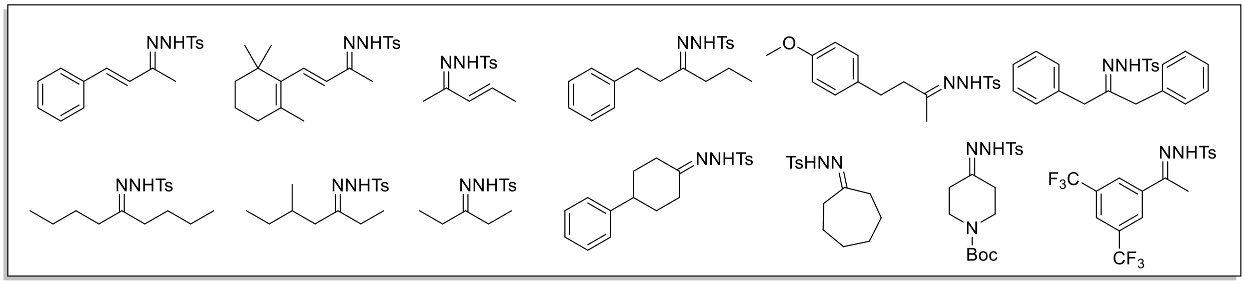

Figure S3. Unsuccessful dialkyl *N*-tosylhydrazones or aryl/alkyl *N*-tosylhydrazone bearing strong EWGs

4 General procedures for the synthesis of starting materials and products

4.1 General procedure for the synthesis of *N*-tosylhydrazones

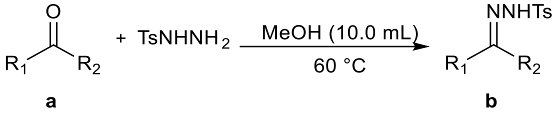

N-tosylhydrazones were prepared according a reported procedure.[1] To a stirred solution of tosylhydrazide (5.0 mmol) in MeOH (10 mL, 0.5 M) at 60 °C, ketone (1.0 equiv.) was added dropwise (or portionwise if solid). The reaction was completed within 0.5-3.0 h. After that, the solvent was removed directly under reduced pressure, and the crude mixture was either directly used after recrystallization or further purified by chromatography if recrystallization doesn't work.

4.2 General procedure for the synthesis of hindered ethers (Procedure A)

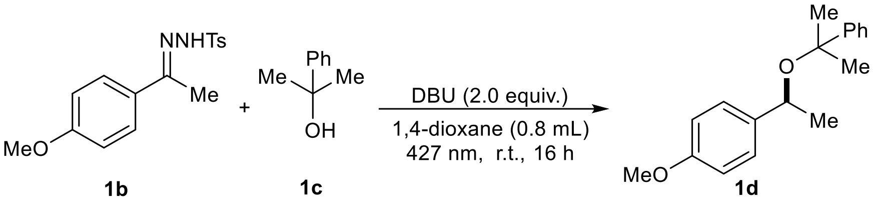

A dry 5 mL Schlenk tube containing a stirring bar was charged with *N*-tosylhydrazone (0.2 mmol, 1.0 equiv.). After purging the flask three times under vacuum and three times under argon, it was charged with **1c** (2.0 mmol, 10.0 equiv.), DBU (0.4 mmol, 2.0 equiv.) and anhydrous 1,4-Dioxane (0.8 mL), successively. The reaction was kept for 16 h under 40 W 427 nm Kessil lamp reaction setup (the progress can be monitored *via* TLC). Then, the resulting mixture is concentrated in vacuo. Products were purified *via* column chromatography with ethyl acetate and hexane as solvents.

4.3 General procedure for the synthesis of polyfluorinated ethers (Procedure B)

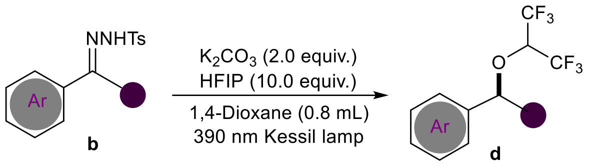

A dry 5 mL Schlenk tube containing a stirring bar was charged with *N*-tosylhydrazone (0.2 mmol, 1.0 equiv.) and K_2CO_3 (0.4 mmol, 2.0 equiv.). After purging the flask three times under vacuum and three times under argon, it was charged with 2.0 mmol of alcohols (10.0 equiv.) and anhydrous 1,4-Dioxane (0.8 mL), successively. The reaction was kept for 16 h under 40 W 390 nm Kessil lamp reaction setup (the progress can be monitored *via* TLC). Then, the resulting mixture is concentrated in vacuo. Products were purified *via* column chromatography with ethyl acetate and hexane as solvents.

4.4 General procedure for the synthesis of fenofibrate analogues (Procedure C)

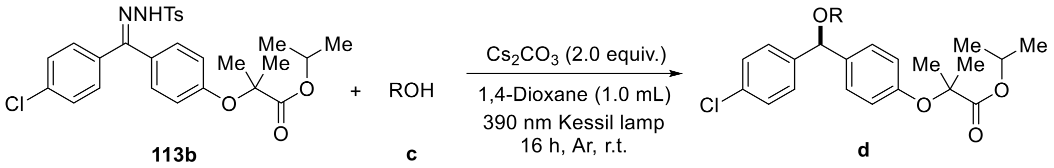

A dry 5 mL Schlenk tube containing a stirring bar was charged with *N*-tosylhydrazone **113b** (0.2 mmol, 1.0 equiv.) and Cs_2CO_3 (0.4 mmol, 2.0 equiv.). After purging the flask three times under vacuum and three times under argon, it was charged with HFIP (0.7 mmol, 3.5 equiv.) and anhydrous 1,4-Dioxane (1.0 mL), successively. The reaction was kept for 16 h under 40 W 390 nm Kessil lamp reaction setup (the progress can be monitored *via* TLC). Then, the resulting mixture is concentrated in vacuo. Products were purified *via* column chromatography with ethyl acetate and hexane as solvents.

4.5 General procedure for the synthesis of 74d or 97d at gram scale

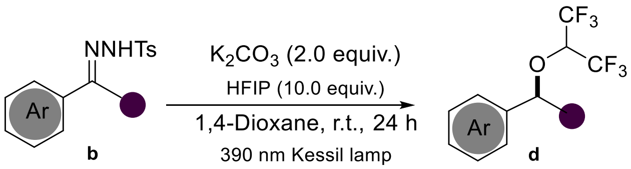

A dry 100 mL Schlenk round bottom flask containing a stirring bar was charged with 1.5 g of *N*-tosylhydrazone (1.0 equiv.) and K_2CO_3 (2.0 equiv.). After purging the flask for three times under vacuum and three times under argon, it was charged with HFIP (10.0 equiv.) and anhydrous 1,4-Dioxane (0.2 M), successively. The reaction was kept for 24 h under 40 W 390 nm Kessil lamp reaction setup (the progress can be monitored *via* TLC). Then, the resulting mixture concentrated in vacuo. Products were purified *via* column chromatography with ethyl acetate and hexane as solvents.

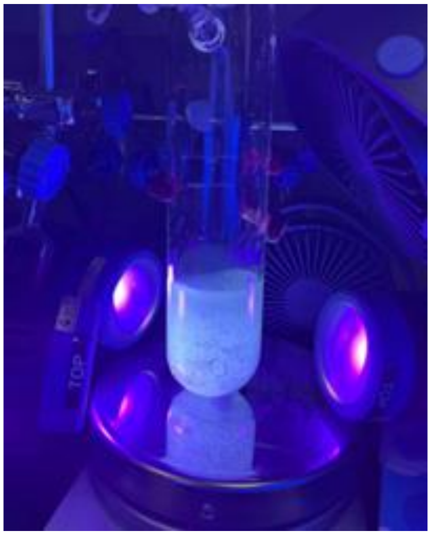

Figure S4. Photochemical reaction at gram-scale.

4.6 Method A for the transformation of compound 74d

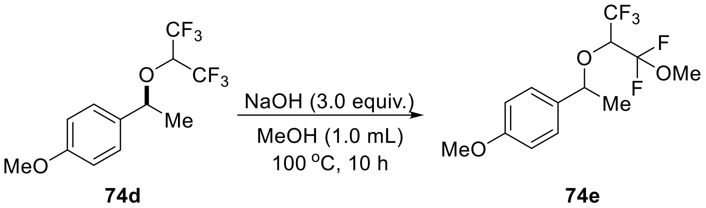

In a 4 mL vial, **74d** (60.4 mg, 0.2 mmol, 1.0 equiv.) and NaOH (24 mg, 0.6 mmol, 3.0 equiv.) was dissolved in 1.0 mL of dry MeOH.[2] The resulting mixture was stirred at 100 °C for 10 hours. The reaction mixture was cooled to room temperature, and then it was passed through a short pad of

celite with DCM. The solution was concentrated in vacuo. The residue was purified by column chromatography on silica to afford pure **74e** (20.7 mg, 33%).

4.7 Method B for the transformation of compound 97d

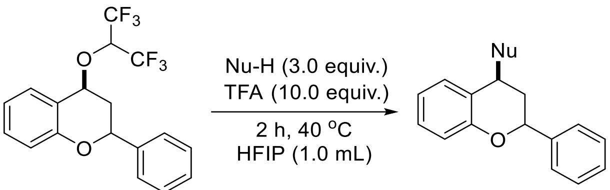

97d **97e, 97f**

A dry 5 mL Schlenk tube containing a stirring bar was charged with **97d** (75.2 mg, 0.2 mmol, 1.0 equiv.) and arenes (0.6 mmol, 3.0 equiv.).[3] After purging the flask three times under vacuum and three times under argon, it was charged with TFA (153.2 μL, 2.0 mmol, 10.0 equiv.) and HFIP (1.0 mL), successively. The reaction was kept for 2 h under 40 °C (the progress can be monitored *via* TLC). Then, the resulting mixture is concentrated in vacuo. Products were purified *via* column chromatography with ethyl acetate and hexane as solvents.

4.8 Gram-scale synthesis ovanoxerine

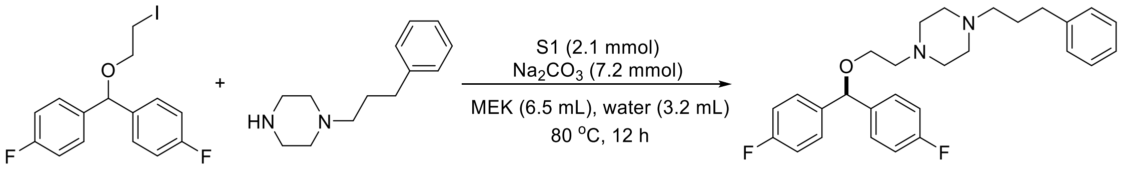

126d **S1** **126e**

A mixture of compound **126d** (1.0 g, 2.7 mmol), Na_2CO_3 (0.8 g, 7.2 mmol), **S1** (0.9 g, 2.1 mmol), MEK (6.5 mL) and water (3.2 mL) was heated at 75-80 °C for 12 h, then cooled to <35 °C.[4] The aqueous layer was separated and back-extracted with MEK (3 × 3 mL). The combined organic extracts were washed with saturated aqueous sodium chloride solution (3.0 mL) and concentrated in vacuo. The concentrate was dissolved in ethanol (4.0 mL), clarified by filtration (to remove extraneous matter) and treated with concentrated hydrochloric acid (0.5 mL) at 15-25 °C. The batch was stirred at 15-25 °C for 2 h, treated with acetone (6.0 mL), cooled to 0-5 °C, and stirred for 2 h. Products were purified *via* column chromatography with ethyl acetate and hexane as solvents.

5 Mechanistic studies

5.1 Time course

A dry 5 mL Schlenk tube containing a stirring bar was charged with *N*-tosylhydrazone (0.2 mmol, 1.0 equiv.). After purging the flask three times under vacuum and three times under argon, it was charged with 2.0 mmol of **1c** (10.0 equiv.), DBU (0.4 mmol, 2.0 equiv.) and anhydrous 1,4-dioxane (0.8 mL), successively. The reaction was kept for 2 h, 4 h, 6 h, 8 h, 10, 12 h, 14 h, 16 h under 40 W 427 nm Kessil lamp reaction setup (the progress can be monitored *via* TLC). Then, the resulting mixture is concentrated in vacuo. Products were purified *via* column chromatography with ethyl acetate and hexane as solvents.

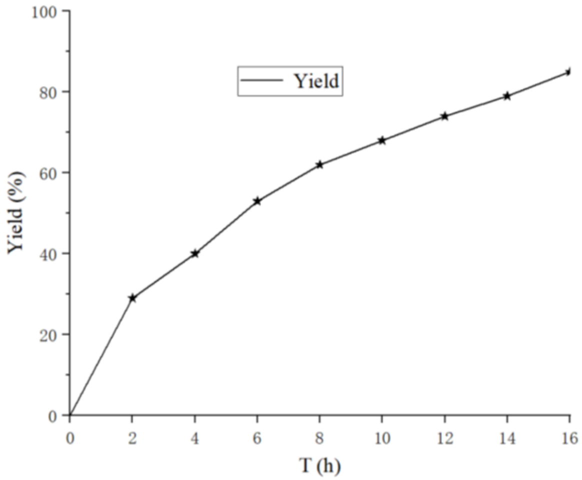

Figure S5. Time course experiment, yield was determined by ^{1}H NMR with 1,3,5-trimethoxybenzene as the internal standard.

5.2 On-off Experiments

A dry 10 mL Schlenk tube containing a stirring bar was charged with *N*-tosylhydrazone **1b** (0.8 mmol, 1.0 equiv.) and 1,3,5-trimethoxybenzene (0.2 mmol, as the internal standard). After purging the flask three times under vacuum and three times under argon, it was charged with HFIP (8.0 mmol, 10.0 equiv.), DBU (1.6 mmol, 2.0 equiv.) and anhydrous 1,4-Dioxane (3.2 mL), successively. Then the mixture was irradiated by 40 W 427 nm Kessil lamps reaction setup at room temperature. After 2 h, the Kessil lamps were turned off, and 0.5 mL of reaction solvent was taken for NMR analysis. Then the tube was reacted in the absence of light for an additional 2 h, and 0.5 mL of reaction solvent was removed for analysis, and the Kessil lamps were turned back on to irradiate the analyzed mixtures. So repeatedly, the yields were determined by ^{1}H NMR spectroscopy.

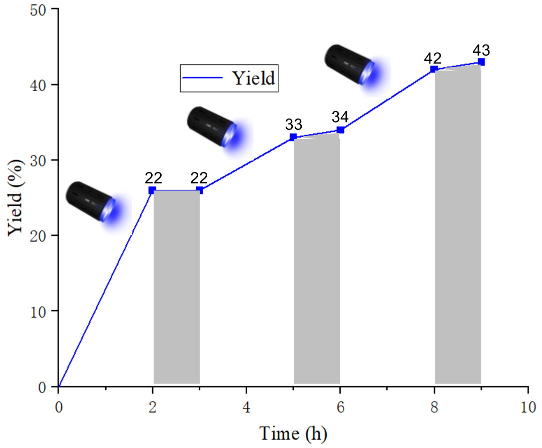

Figure S6. The comparison of On-off experiment yields determined by hydrogen spectrometry.

5.3 UV-Vis absorption studies

UV/Vis absorption spectra were measured in a 1 cm quartz cuvette using a gilent Cary 100 spectrophotometer. Absorption spectra of individual reaction components and mixtures thereof were recorded. A bathochromic shift was observed for a mixture of **1b** and DBU in 1,4-dioxane (200 μM), this indicates the formation of a non-covalent interactions (**Figure S6**, black band). What's more, the UV/Vis absorption of the mixture of **1b**, **1c** and DBU (**Figure S5**, red band) shows no significant difference to the mixture of **1b** and DBU, indicating that **1c** didn't participate in the formation of non-covalent interactions. In addition, a bathochromic shift was observed for a mixture of **1b** and K_2CO_3 in ACN (80 μM) (**Figure S7**, red band).

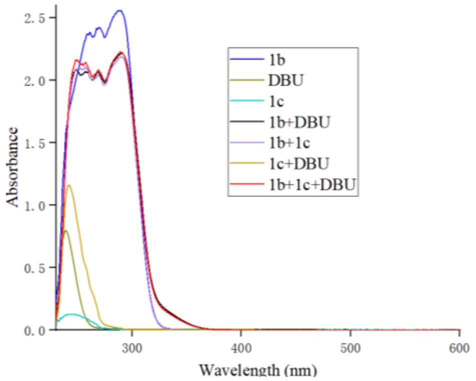

Figure S7. UV/vis absorption spectra of individual reaction components and a combination thereof. All spectra were measured in 1,4-dioxane and with a concentration of 200 μM **1b**, 200 μM DBU.

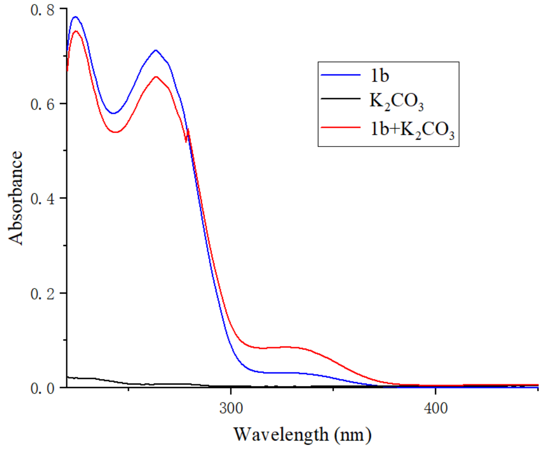

Figure S8. UV/vis absorption spectra of individual reaction components and a combination thereof. All spectra were measured in ACN and with a concentration of 80 μM **1b**, 80 μM K_2CO_3.

5.4 Quenching experiments

The reaction was operated under standard conditions with extra 2,2,6,6-tetramethyl-1-piperinedinyloxy (TEMPO) or Butylated Hydroxytoluene (BHT), and the yields were not significantly inhibited, indicating that the reaction was not proceeding through radical mechanism.

Table S4. Quenching experiments.

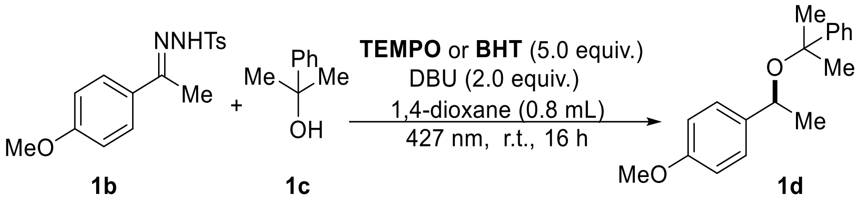

Entry	Quencher (5.0 equiv.)	Yield (%)[a]
1	TEMPO	76
2	BHT	68

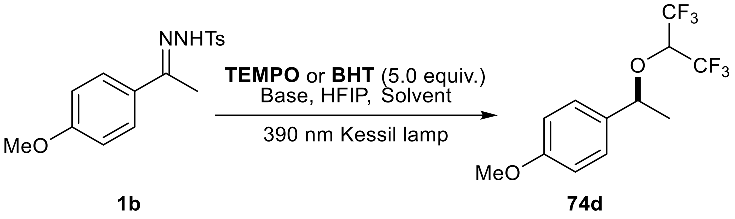

Entry	Quencher (5.0 equiv.)	Yield (%)[a]
1	TEMPO	74
2	BHT	83

[a]Yield was determined by ^{1}H NMR with 1,3,5-trimethoxybenzene as the internal standard.

5.5 Carbene trapping experiment

5.5.1 Cyclopropane trapping reaction

A dry 5 mL Schlenk tube containing a stirring bar was charged with 0.2 mmol of *N*-tosylhydrazone (1.0 equiv.). After purging the flask three times under vacuum and three times under argon, it was charged with base (2.0 equiv.), alcohol (5.0 equiv.), styrene (5.0 equiv.) and 1,4-dioxane (0.8 mL). The reaction was kept for 16 h under 40 W Kessil lamps reaction setup. Then, the resulting mixture underwent an aqueous workup (using distilled water) and was extracted three times with dichloromethane. The combined organic layers were dried over anhydrous Na_2SO_4, filtered and concentrated in vacuo. Then the product was purified by column chromatography and determined

by NMR. Following workup, the product was purified by column chromatography (hexane:EtOAc, 100:1) to give the title compound as a colorless oil (isolated yield: 7%/15%, d.r. = 3:2).

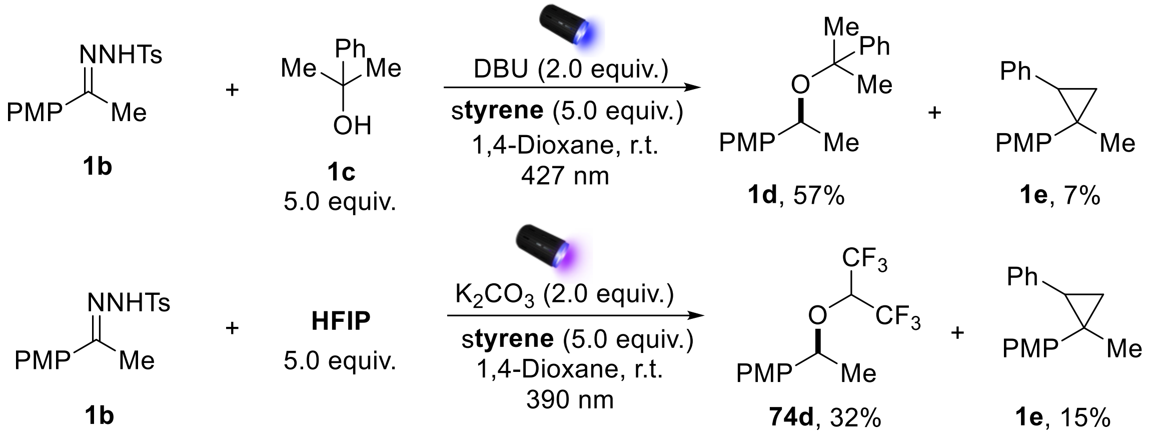

1-Methoxy-4-(1-methyl-2-phenylcyclopropyl)benzene (1e)

^{1}H NMR (500 MHz, $CDCl_3$) δ 7.34 (td, *J* = 7.3, 5.7 Hz, 6.2H), 7.27 – 7.23 (m, 1H), 7.08 (tt, *J* = 7.9, 1.6 Hz, 1.3H), 7.04 – 7.00 (m, 2H), 6.93 – 6.89 (m, 2H), 6.79 – 6.75 (m, 1.3H), 6.73 – 6.69 (m, 1.3H), 3.85 (s, 3H), 3.75 (s, 1.7H), 2.39 (dd, *J* = 8.8, 6.3 Hz, 1H), 2.21 (dd, *J* = 8.6, 5.9 Hz, 0.6H), 1.53 (s, 1.8H)1.47 (t, *J* = 5.5 Hz, 0.7H), 1.42 (dd, *J* = 8.8, 5.1 Hz, 1H), 1.27 (dd, *J* = 8.6, 5.1 Hz, 0.6H), 1.23 (dd, *J* = 6.3, 5.0 Hz, 1H), 1.12 (s, 3H). **^{13}C NMR** (126 MHz, $CDCl_3$) δ 157.7, 157.7, 140.2, 140.1, 139.3, 134.5, 130.8, 129.2, 128.1, 128.1, 127.5, 127.5, 125.9, 125.0, 113.8, 113.3, 55.3, 55.1, 31.1, 31.1, 30.5, 29.9, 26.5, 21.4, 20.0, 18.4.

HRMS (ESI+), *m/z*: calculated for $C_{17}H_{19}O$ $[M + H]^+$: 239.1436, found: 239.1445.

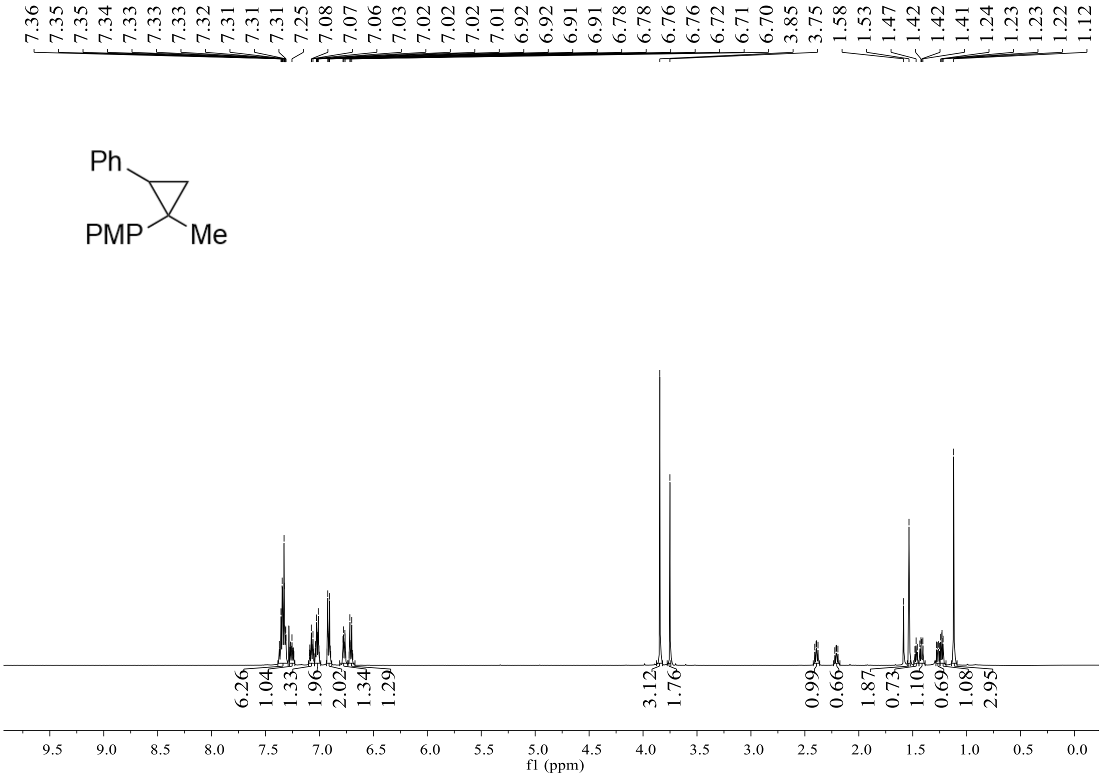

^{1}H NMR spectrum in $CDCl_3$.

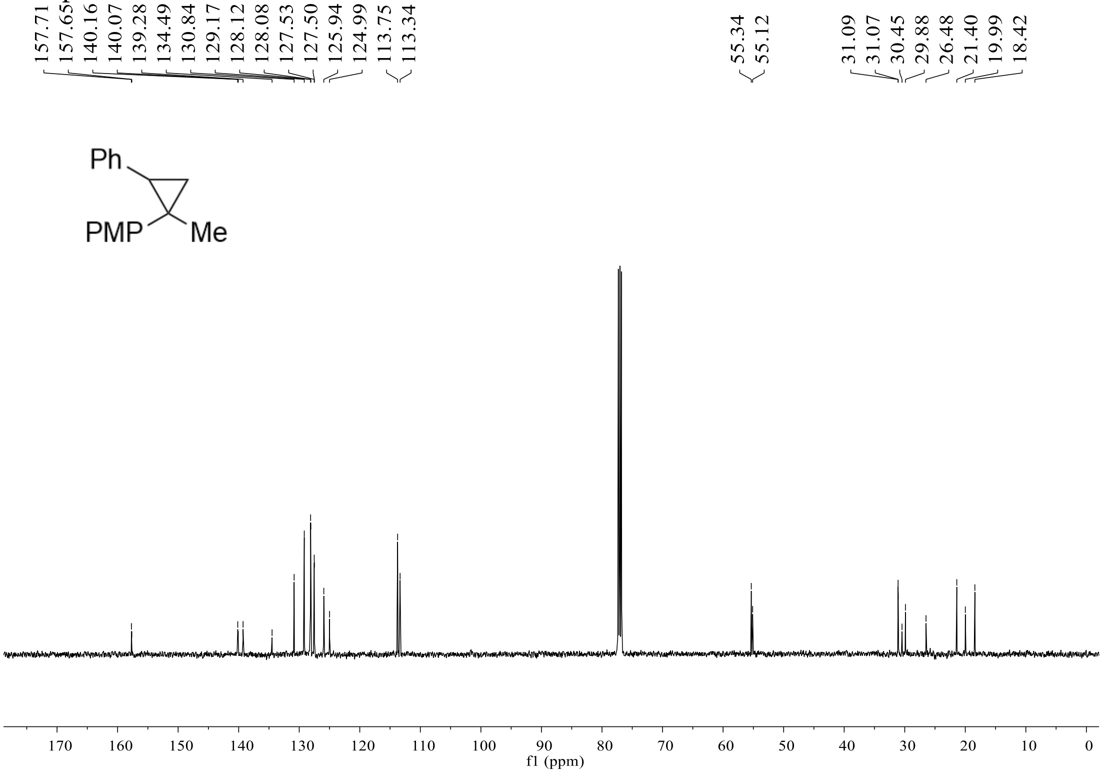

^{13}C NMR spectrum in $CDCl_3$.

5.5.2 Cyclopropene trapping reaction

A dry 5 mL Schlenk tube containing a stirring bar was charged with 0.2 mmol of *N*-tosylhydrazone (1.0 equiv.). After purging the flask three times under vacuum and three times under argon, it was charged with base (2.0 equiv.), phenylacetylene (10.0 equiv.) and 1,4-dioxane (0.8 mL). The reaction was kept for 16 h under 40 W Kessil lamps reaction setup. Then, the resulting mixture underwent an aqueous workup (using distilled water) and was extracted three times with dichloromethane. The combined organic layers were dried over anhydrous Na_2SO_4, filtered and concentrated in vacuo. Then the product was purified by column chromatography (hexane:EtOAc, 200:1) to give the title compound as a colorless oil (isolated yield: 15%/13%).

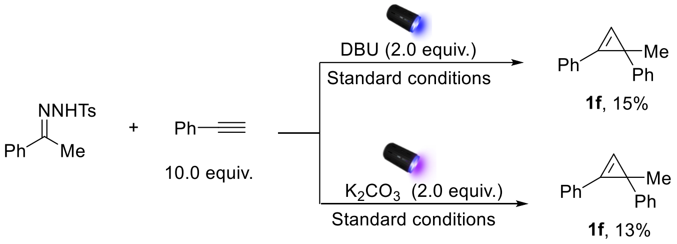

(1-Methylcycloprop-2-ene-1,2-diyl)dibenzene (1f)

^{1}H NMR (500 MHz, $CDCl_3$) δ 7.55 – 7.52 (m, 2H), 7.42 – 7.38 (m, 2H), 7.37 – 7.27 (m, 6H), 7.19 – 7.14 (m, 1H), 1.81 (s, 3H). **^{13}C NMR** (126 MHz, $CDCl_3$) δ 153.5, 134.0, 133.6, 133.4, 132.6, 132.4, 130.8, 129.7, 128.8, 113.3, 29.8, 28.3.

EI-MS calculated for [$C_{16}H_{14}$] m/z 206.1096, found m/z 206.1088.

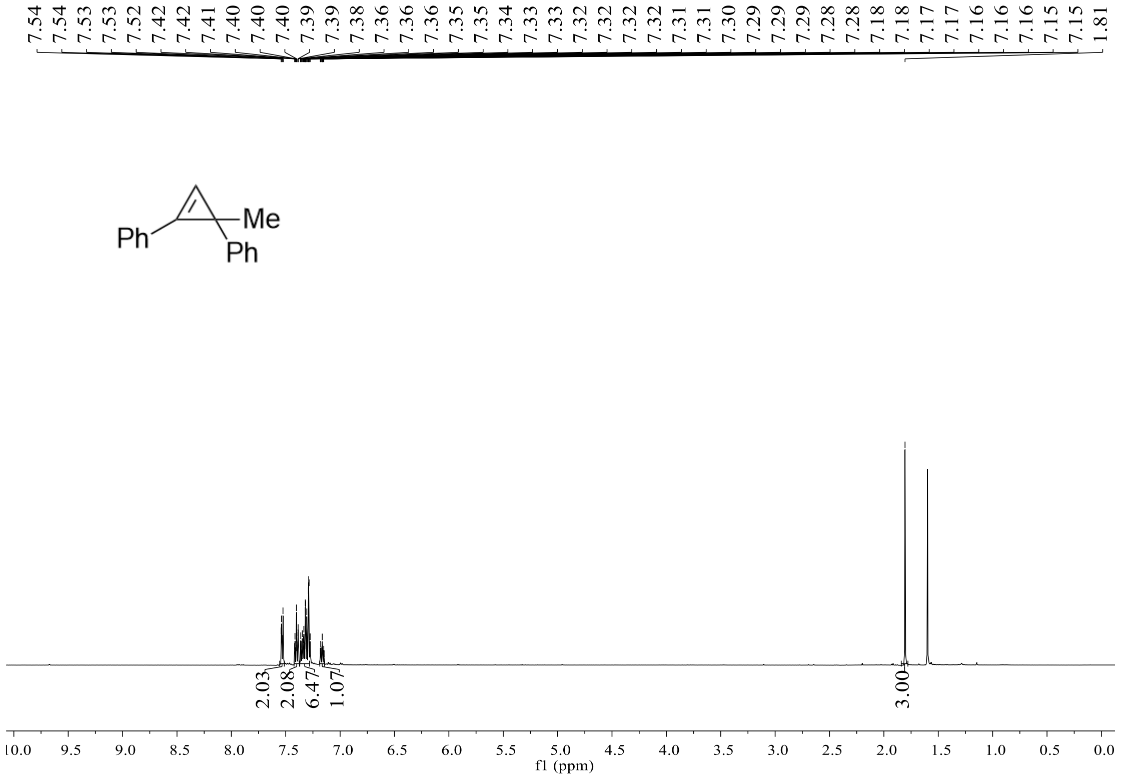

^{1}H NMR spectrum in $CDCl_3$.

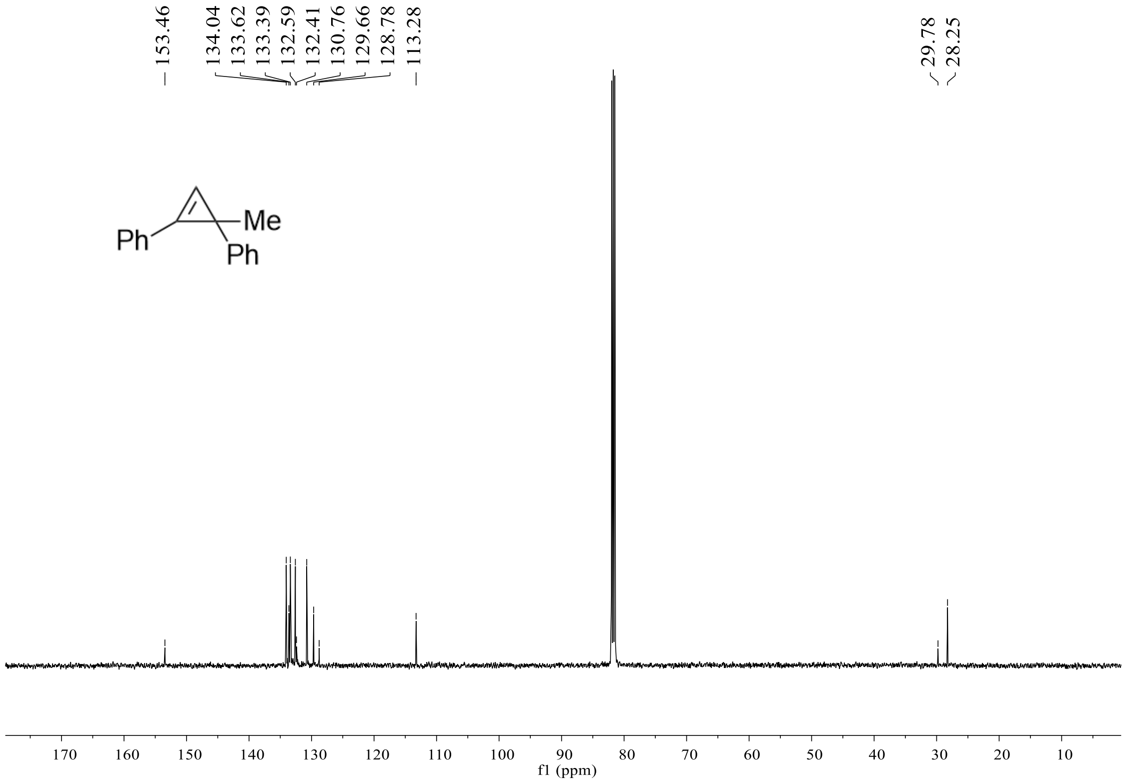

^{13}C NMR spectrum in $CDCl_3$.

5.6 Determination of diazo intermediate

A dry 5 mL Schlenk tube containing a stirring bar was charged with 0.2 mmol of *N*-tosylhydrazone **89b** (1.0 equiv.). After purging the flask three times under vacuum and three times under argon, it was charged with base (2.0 equiv.) and 1,4-dioxane (0.8 mL). The reaction was kept for 6 h under 40 W Kessil lamps reaction setup. Then, the resulting mixture underwent an aqueous workup (using distilled water) and was extracted three times with dichloromethane. The combined organic layers were dried over anhydrous Na_2SO_4, filtered and concentrated in vacuo. Then the product was purified by column chromatography (hexane:EtOAc, 50:1) to give the compound **89e** as a dark red solid.

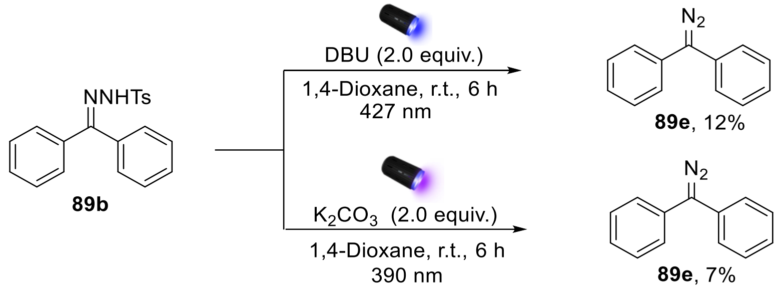

(Diazomethylene)dibenzene (89e)

^{1}H NMR (500 MHz, $CDCl_3$) δ 7.52 – 7.48 (m, 1H), 7.44 – 7.39 (m, 1H), 7.37 – 7.34 (m, 1H), 7.31 (dt, *J* = 7.2, 1.8 Hz, 1H), 7.28 (d, *J* = 2.5 Hz, 1H). **^{13}C NMR** (126 MHz, $CDCl_3$) δ 134.3, 134.0, 133.4, 132.7, 132.6.

HRMS (ESI+), *m/z*: calculated for $C_{13}H_{11}N_2$ $[M + H]^+$: 195.0917, found: 195.0923.

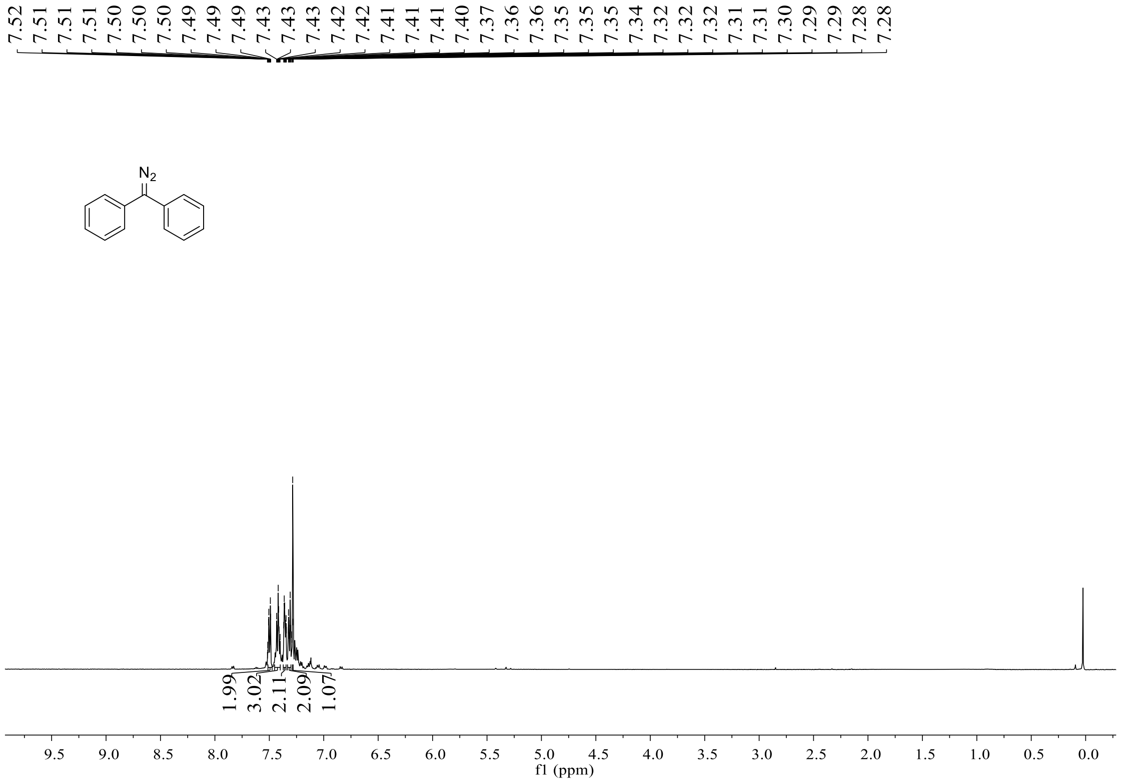

^{1}H NMR spectrum in $CDCl_3$.

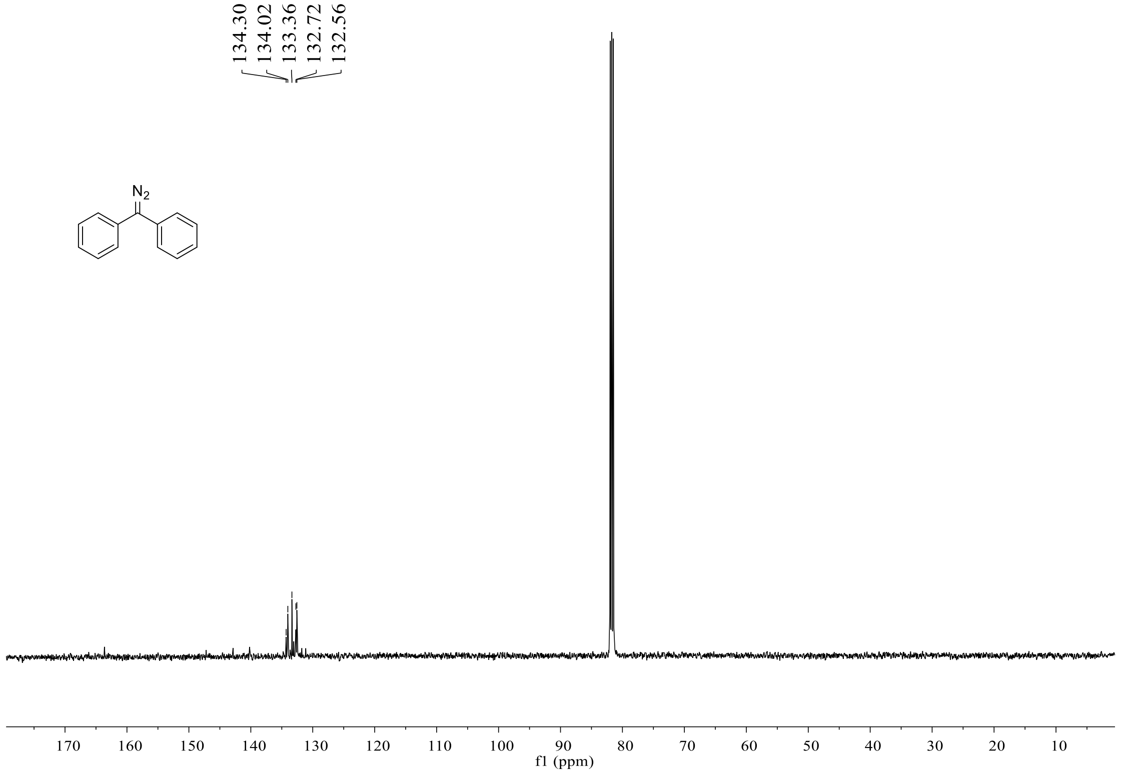

^{13}C NMR spectrum in $CDCl_3$.

5.7 Labeling experiments

5.7.1 Preparation of sodium 1,1,1,3,3,3-hexafluoropropan-2-olate

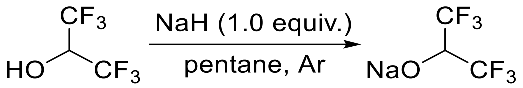

An oven dried 100 mL round bottom flask pre-equipped with a magnetic stirrer was charged with NaH (640 mg, 27 mmol, 1.0 equiv.) and pentane (80 mL) was added under argon gas atmosphere. A condenser with an argon inlet was connected to the flask and the mixture was kept stirring under argon atmosphere. HFIP (2.25 mL, 32.4 mmol, 1.2 equiv.) was slowly added dropwise to the mixture at room temperature *via* a syringe and the reaction mixture was stirred until the completion of H_2 gas evolution. The solution was filtered and the residue was washed with pentane (8 mL). The residue was dissolved in Et_2O (2.5 mL) and concentrated under reduced pressure. The solid was dried under high vacuum atmosphere and the title compound was obtained as a colorless solid (2.63 g, 13.84 mmol, 51%).

^{1}H NMR (500 MHz, D_2O) δ 4.34 (hept, J = 7.0 Hz, 1H). **^{13}C NMR** (126 MHz, D_2O) δ 124.2 (q, $J_{C\text{=}F}$ = 287.4, 286.9 Hz), 72.8 (hept, $J_{C\text{=}F}$ = 29.5 Hz). **^{19}F NMR** (376 MHz, D_2O) δ -76.01 (d, J = 6.9 Hz). **HRMS (ESI+), *m/z***: calculated for C_3HClF_6NaO $[M + Cl]^-$: 224.9523, found: 224.9529.

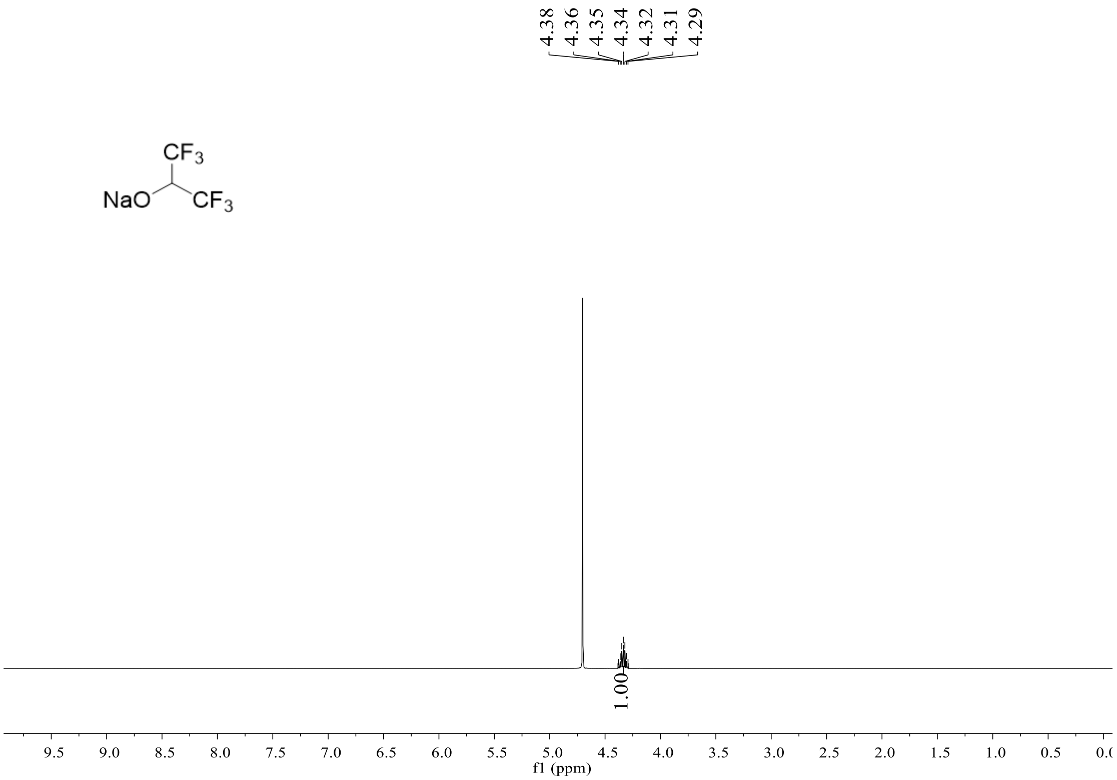

^{1}H NMR spectrum in D_2O.

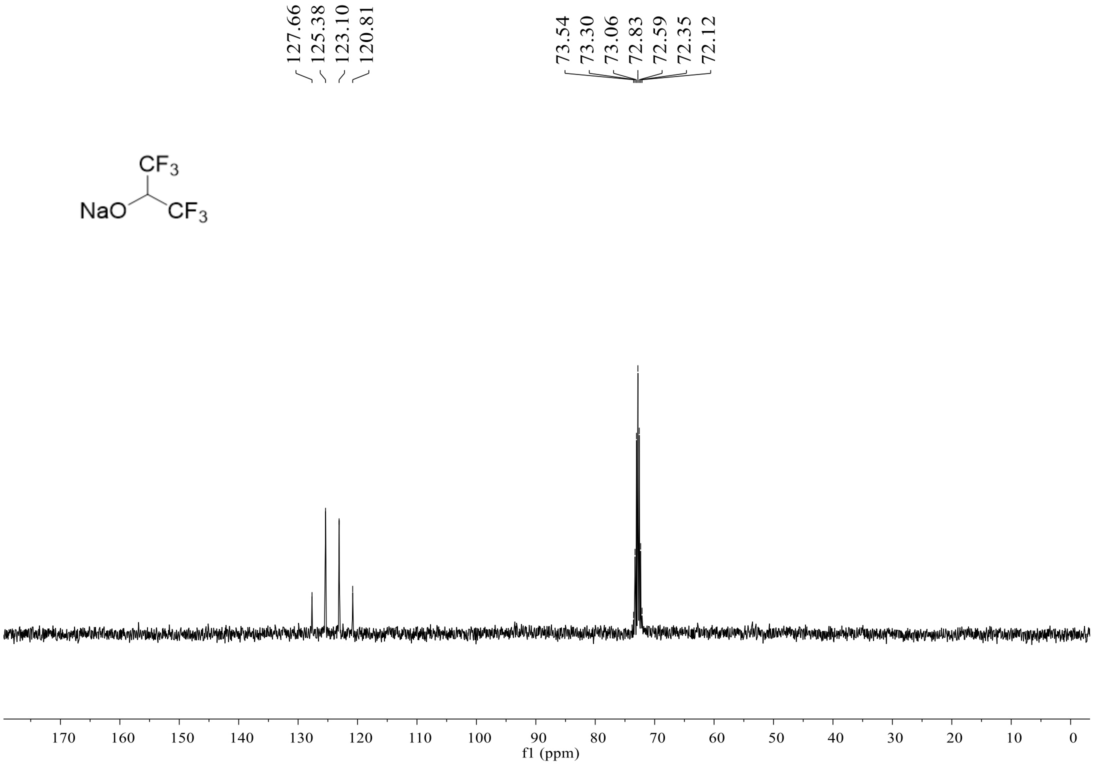

^{13}C NMR spectrum in D_2O.

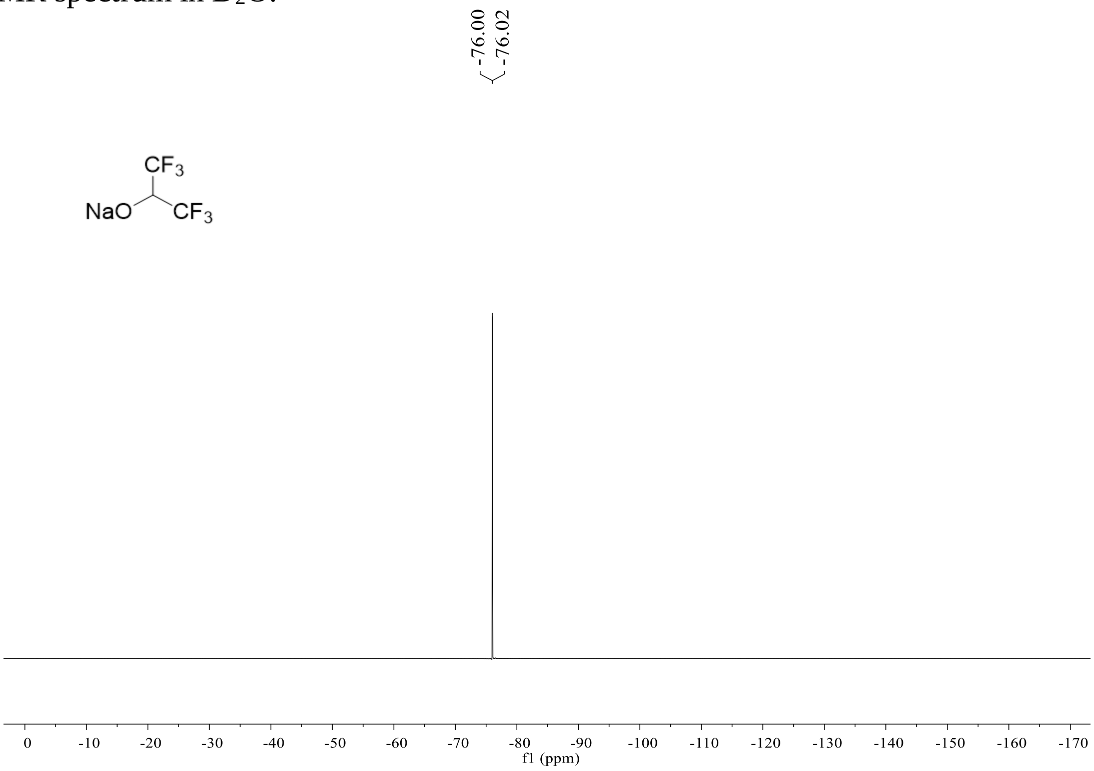

^{19}F NMR spectrum in D_2O.

5.7.2 Preparation of 1,1,1,3,3,3-hexafluoropropan-2-ol-*d*

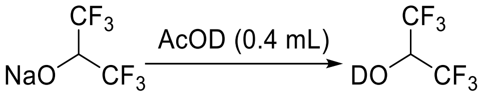

A 10 mL round bottom flask was charged with sodium 1,1,1,3,3,3-hexafluoroisopropan-2-olate (1.7 g, 8.95 mmol, 1.2 equiv.) and closed with a septum. Acetid acid-d_1 (0.43 mL, 7.46 mmol) was slowly added dropwise *via* syringe. The resulting mixture was distilled under ambient pressure and the product was obtained as a colorless liquid (0.9 g, 5.3 mmol, 59%) with an impurity of 30% hexafluoroisopropanol as determined by ^{1}H NMR analysis.

^{1}H NMR (500 MHz, $CDCl_3$) δ 4.41 (hept, J = 5.9 Hz, 1H), 3.44 (s, 0.3H). **^{13}C NMR** (126 MHz, $CDCl_3$) δ 121.3 (q, $J_{C=F}$ = 282.5 Hz), 69.5 (hept, $J_{C=F}$ = 33.7 Hz). **^{19}F NMR** (376 MHz, $CDCl_3$) δ -75.74.

HRMS (ESI+), *m/z*: calculated for $C_3H_2DF_6O$ [M + H]$^+$: 170.0145, found: 170.0139.

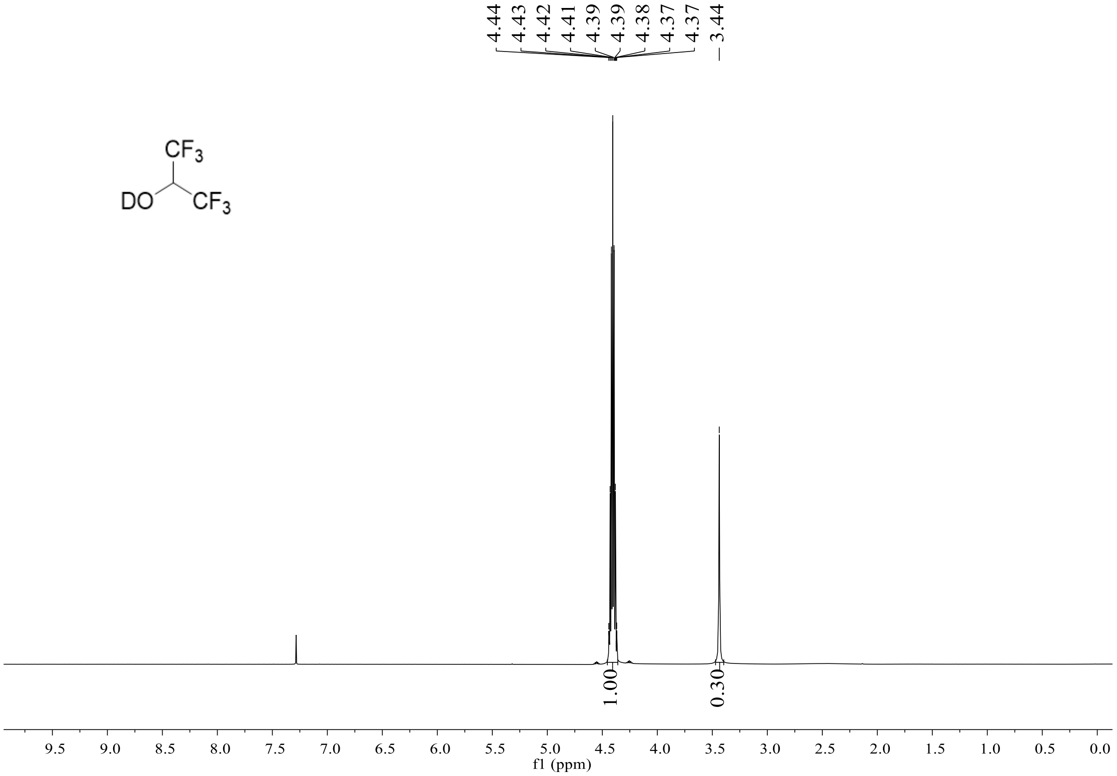

^{1}H NMR spectrum in $CDCl_3$.

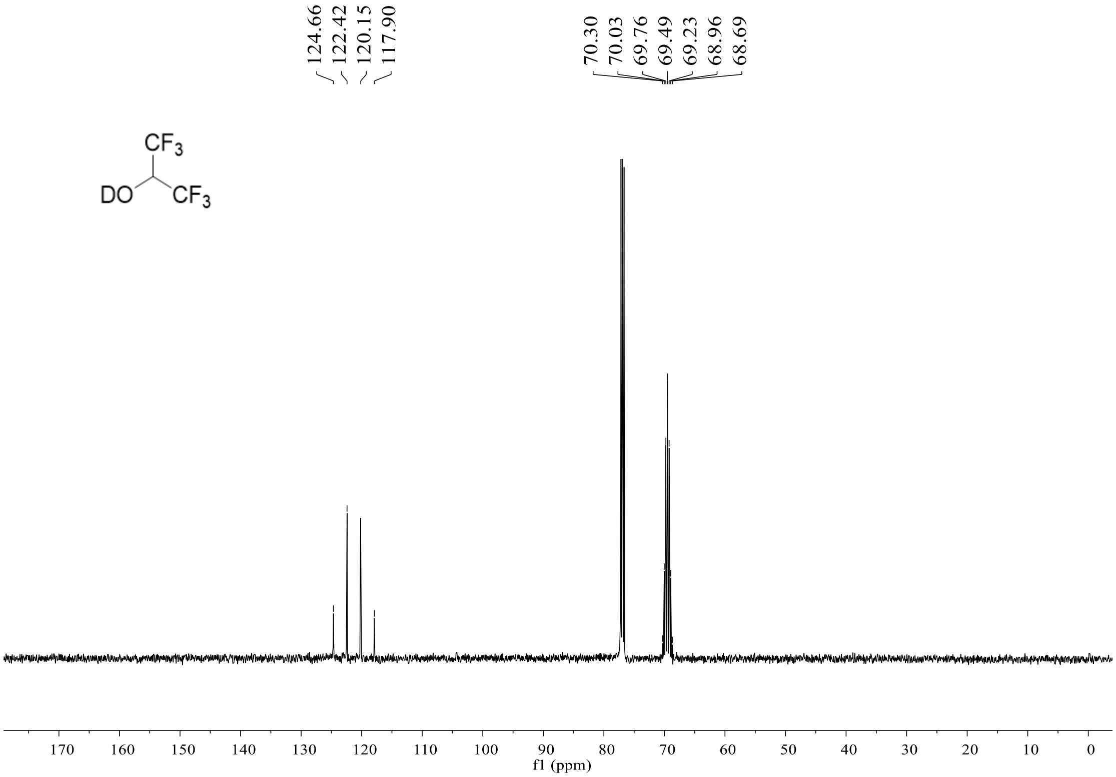

^{13}C NMR spectrum in $CDCl_3$.

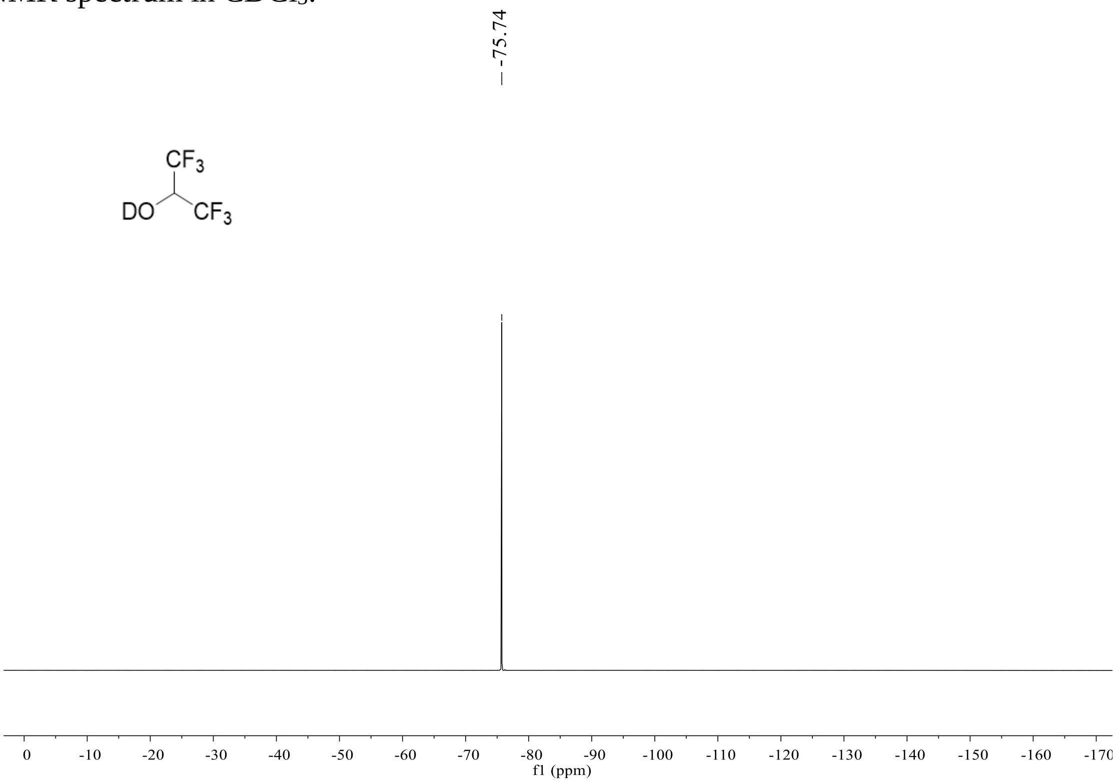

^{19}F NMR spectrum in $CDCl_3$.

5.7.3 Deuterium labelling experiments

A dry 5 mL Schlenk tube containing a stirring bar was charged with 0.2 mmol of *N*-tosylhydrazone (1.0 equiv.). After purging the flask three times under vacuum and three times under argon, it was charged with base (2.0 equiv.), deuterated alcohol (10.0 equiv.) and 1,4-dioxane (0.8 mL). The reaction was kept for 16 h under 40 W Kessil lamps reaction setup. Then, the resulting mixture underwent an aqueous workup (using distilled water) and was extracted three times with dichloromethane. The combined organic layers were dried over anhydrous Na_2SO_4, filtered and concentrated in vacuo. Then the product was purified by column chromatography and determined by NMR. (*Tert*-butylalcohol-O*d* was purchased from Tansoole).

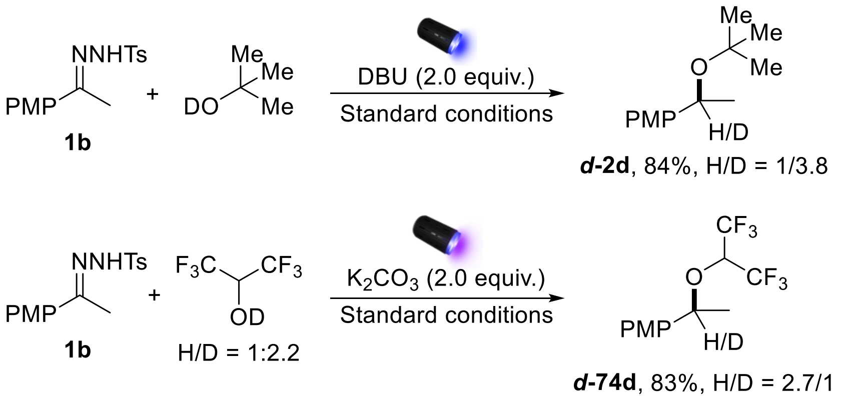

Deuterated 1-(1-(*tert*-butoxy)ethyl)-4-methoxybenzene (*d*-2d)

^{1}H NMR (500 MHz, $CDCl_3$) δ 7.29 (d, J = 8.7 Hz, 2H), 6.87 (d, J = 8.7 Hz, 2H), 4.65 (q, J = 6.5 Hz, 0.2H), 3.82 (s, 3H), 1.37 (d, J = 6.6 Hz, 3H), 1.18 (s, 9H).

HRMS (ESI+), *m/z*: calculated for $C_{13}H_{20}DO_2$ $[M + H]^+$: 210.1599, found: 210.1586.

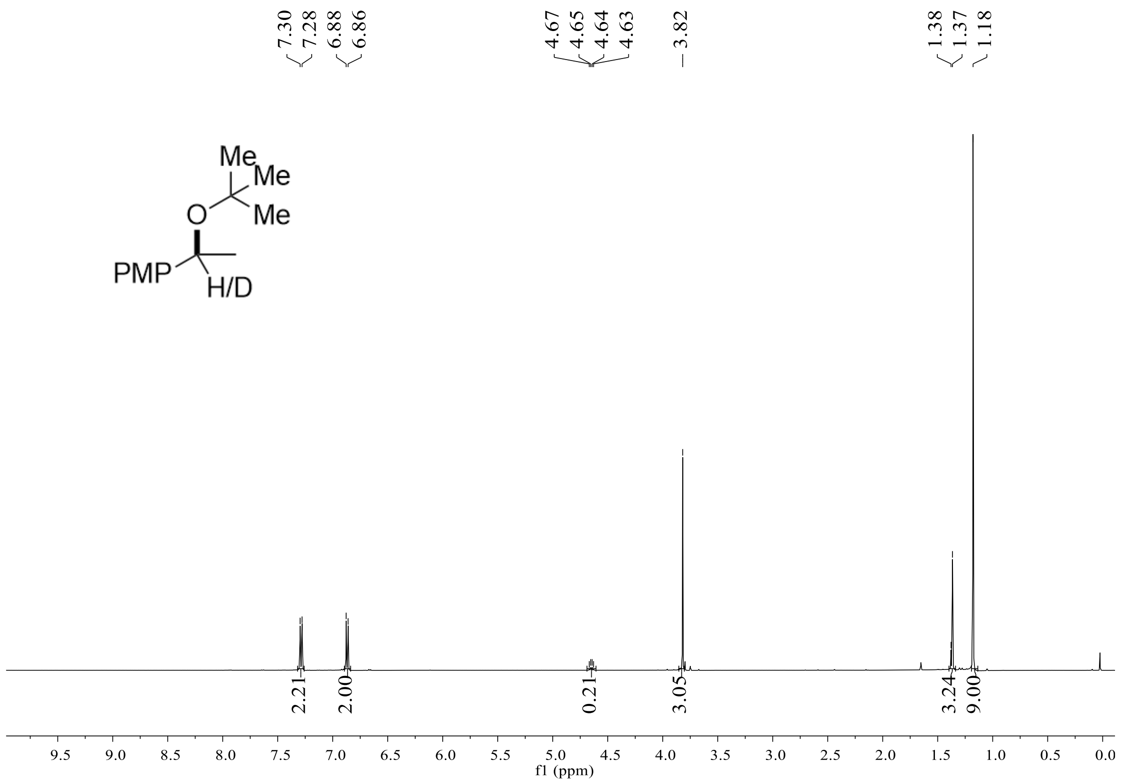

^{1}H NMR spectrum in $CDCl_3$.

Deuterated 1-(1-((1,1,1,3,3,3-hexafluoropropan-2-yl)oxy)ethyl)-4-methoxybenzene (*d*-74d)

^{1}H NMR (500 MHz, $CDCl_3$) δ 7.34 – 7.26 (m, 2H), 6.98 – 6.88 (m, 2H), 4.82 (d, *J* = 6.4 Hz, 0.75H), 3.98 (pd, *J* = 6.0, 1.6 Hz, 1H), 3.85 (s, 3H), 1.59 (d, *J* = 6.5 Hz, 3H).

HRMS (ESI+), *m/z*: calculated for $C_{12}H_{12}DF_6O_2$ $[M + H]^+$: 304.0877, found: 304.0875.

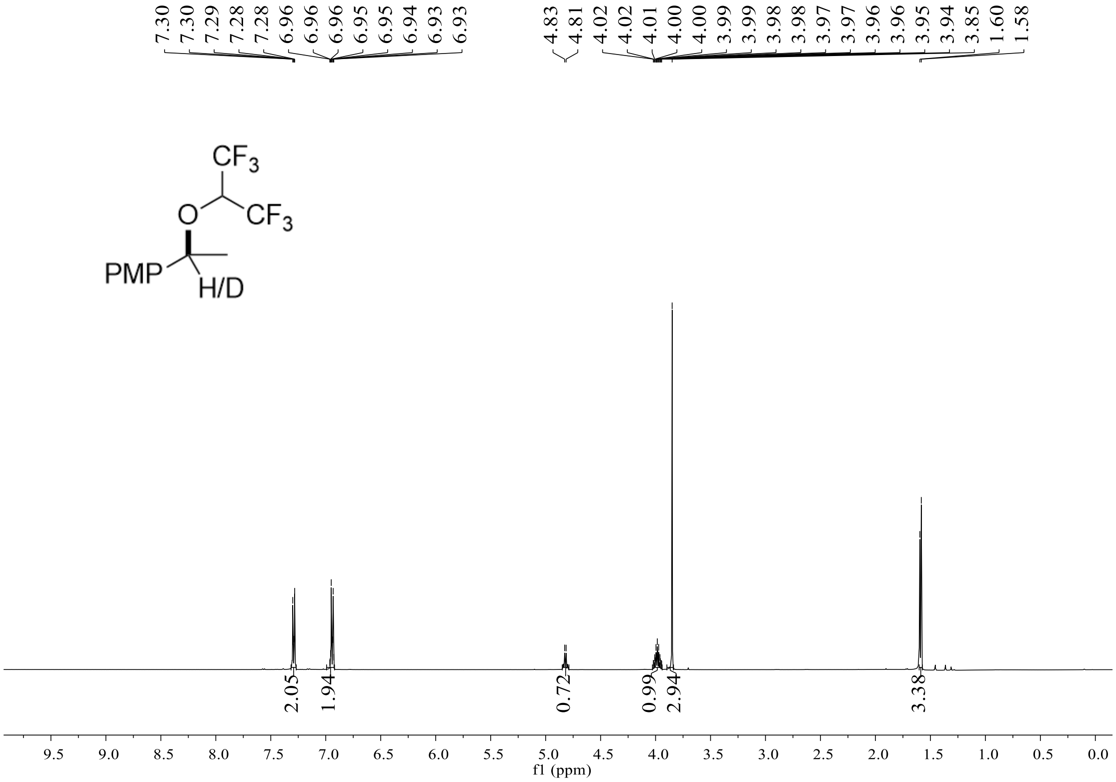

^{1}H NMR spectrum in $CDCl_3$.

5.8 DFT computational details

All calculations were performed using Gaussian 16, Revision A.03 package.[5] All of the reactants, intermediates, transition states, products were optimized by the DFT with the M06-2X functional.[6] For geometry optimizations and frequency calculations, BS-I basis set system was employed. In BS-I, we employed def2-SVP basis sets for all atom. All the stationary structures were characterized with no imaginary frequency and the transition state structures (TSs) were characterized with a single imaginary frequency. Intrinsic reaction coordinate (IRC) calculations were performed on the TSs. The solvent effect of 1,4-dioxane was evaluated through the SMD method,[7] in which a better basis system BS-II was used. In BS-II, we employed def2-TZVP basis sets for all atom. All reported energies are free energies at 1 M.

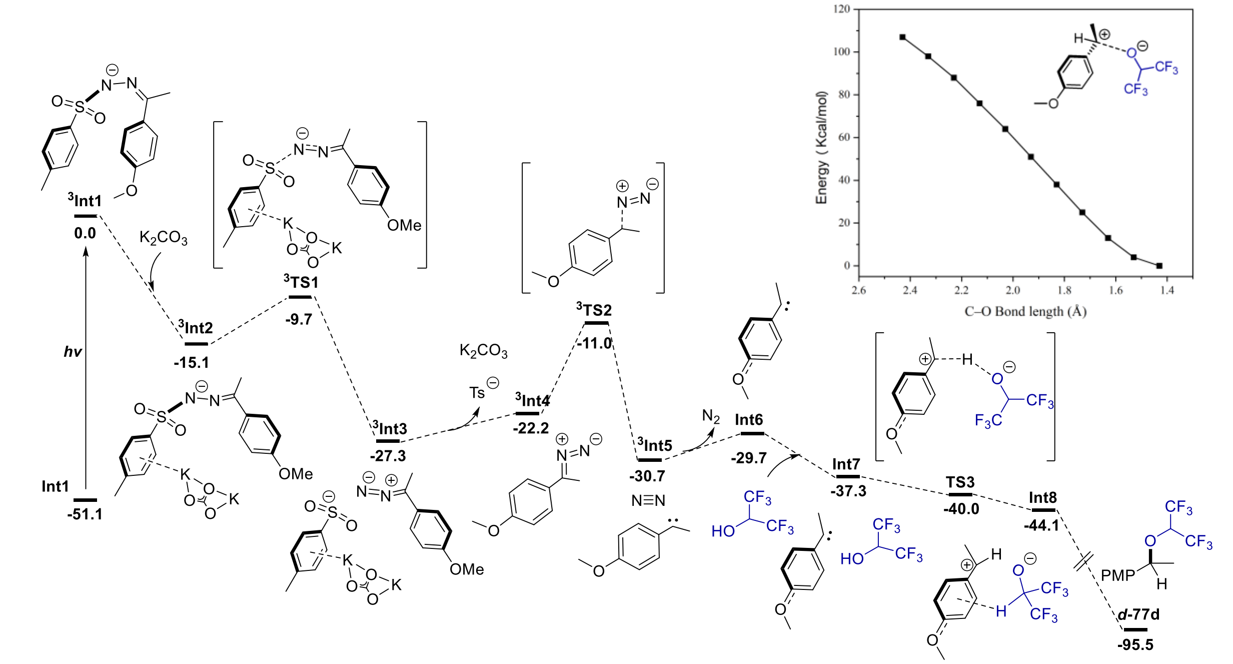

Figure S9. The DFT calculated Gibbs free energy profiles for the polyfluorinated alcohol involved reaction. (in kcal/mol)

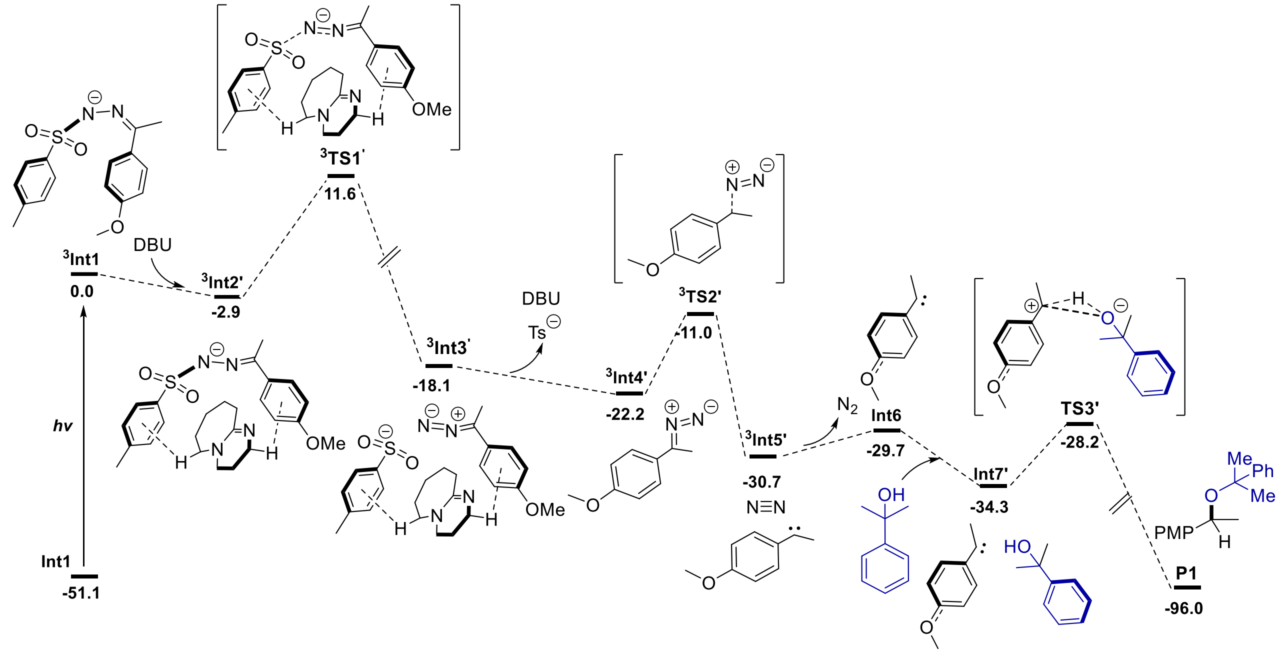

Figure S10. The DFT calculated Gibbs free energy profiles for the hindered alcohol involved reaction. (in kcal/mol).

6 Characterization data of synthesized hindered ethers and polyfluorinated ethers

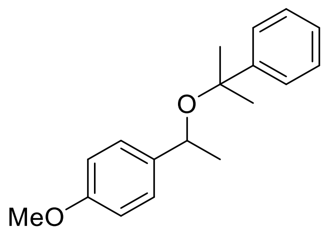

1-Methoxy-4-(1-((2-phenylpropan-2-yl)oxy)ethyl)benzene (1d)

Prepared according to the general procedure A for the Etherification. Following workup, the product was purified by column chromatography (hexane:EtOAc, 20:1) to give the title compound as a colorless oil (isolated yield: 85%).

^{1}H NMR (600 MHz, $CDCl_3$) δ 7.46 (d, *J* = 7.3 Hz, 2H), 7.33 (t, *J* = 7.7 Hz, 2H), 7.27 (d, *J* = 7.6 Hz, 1H), 7.18 (d, *J* = 8.7 Hz, 2H), 6.83 (d, *J* = 8.6 Hz, 2H), 4.27 (q, *J* = 6.5 Hz, 1H), 3.80 (s, 3H), 1.51 (s, 3H), 1.38 (s, 3H), 1.30 (d, *J* = 6.5 Hz, 3H). **^{13}C NMR** (151 MHz, $CDCl_3$) δ 158.2, 146.9, 139.6, 128.0, 126.9, 126.7, 126.1, 113.4, 77.8, 71.3, 55.2, 31.5, 27.2, 26.5.

HRMS (ESI+), *m/z*: calculated for $C_{18}H_{23}O_2$ $[M + H]^+$: 271.1698, found: 271.1681.

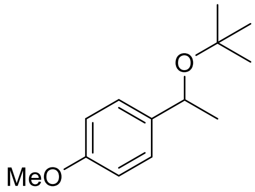

1-(1-(*Tert*-butoxy)ethyl)-4-methoxybenzene (2d)

Prepared according to the general procedure A for the Etherification. Following workup, the product was purified by column chromatography (hexane:EtOAc, 20:1) to give the title compound as a colorless oil (isolated yield: 64%).

^{1}H NMR (500 MHz, $CDCl_3$) δ 7.29 (d, *J* = 8.6 Hz, 2H), 6.87 (d, *J* = 8.7 Hz, 2H), 4.65 (q, *J* = 6.5 Hz, 1H), 3.82 (s, 3H), 1.37 (d, *J* = 6.5 Hz, 3H), 1.18 (s, 9H). **^{13}C NMR** (126 MHz, $CDCl_3$) δ 158.2, 139.7, 126.6, 113.5, 74.0, 69.4, 55.2, 28.6, 26.7.

HRMS (ESI+), *m/z*: calculated for $C_{18}H_{20}O_2Na$ $[M + Na]^+$: 231.1361, found: 231.1347.

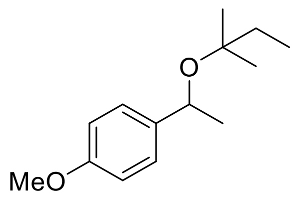

1-Methoxy-4-(1-(*tert*-pentyloxy)ethyl)benzene (3d)

Prepared according to the general procedure A for the Etherification. Following workup, the product was purified by column chromatography (hexane:EtOAc, 20:1) to give the title compound as a colorless oil (isolated yield: 75%).

^{1}H NMR (500 MHz, $CDCl_3$) δ 7.29 (d, *J* = 8.7 Hz, 2H), 6.87 (d, *J* = 8.6 Hz, 2H), 4.63 (q, *J* = 6.5 Hz, 1H), 3.82 (s, 3H), 1.58 (dq, *J* = 14.8, 7.5 Hz, 1H), 1.46 (dq, *J* = 14.7, 7.5 Hz, 1H), 1.37 (d, *J* = 6.5 Hz, 3H), 1.11 (s, 3H), 1.07 (s, 3H), 0.88 (t, *J* = 7.5 Hz, 3H). **^{13}C NMR** (126 MHz, $CDCl_3$) δ 158.2, 139.9, 126.7, 113.4, 76.3, 69.1, 55.2, 34.1, 26.8, 26.0, 25.7, 8.7.

HRMS (ESI+), *m/z*: calculated for $C_{14}H_{22}O_2Na$ $[M + Na]^+$: 245.1512, found: 245.1534.

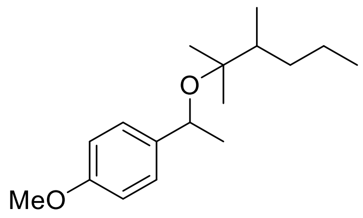

1-(1-((2,3-Dimethylhexan-2-yl)oxy)ethyl)-4-methoxybenzene (4d)

Prepared according to the general procedure A for the Etherification. Following workup, the product was purified by column chromatography (hexane:EtOAc, 20:1) to give the title compound as a colorless oil (isolated yield: 61%).

^{1}H NMR (500 MHz, $CDCl_3$) δ 7.28 (d, *J* = 8.7 Hz, 2H), 6.87 (d, *J* = 8.4 Hz, 2H), 4.64 (qd, *J* = 6.5, 2.9 Hz, 1H), 3.82 (d, *J* = 1.1 Hz, 3H), 1.72 – 1.64 (m, 0.7H), 1.64 – 1.53 (m, 1H), 1.52 – 1.39 (m, 1.3H), 1.35 (dd, *J* = 6.5, 3.4 Hz, 3H), 1.29 – 1.18 (m, 0.6H), 1.08 (d, *J* = 11.7 Hz, 3H), 1.05 – 0.88 (m, 7.4H), 0.85 (dt, *J* = 7.0, 3.7 Hz, 3H). **^{13}C NMR** (126 MHz, $CDCl_3$) δ 158.2, 158.1, 140.2, 140.1, 126.7, 126.7, 113.4, 78.9, 78.9, 68.9, 68.8, 55.2, 55.2, 42.0, 42.0, 34.0, 33.6, 26.9, 26.8, 23.6, 23.3, 23.2, 23.1, 21.4, 21.4, 14.6, 14.6, 14.4, 14.4.

HRMS (ESI+), *m/z*: calculated for $C_{17}H_{29}O_2$ $[M + H]^+$: 265.2168, found: 265.2172.

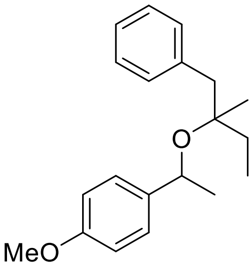

1-Methoxy-4-(1-((2-methyl-4-phenylbutan-2-yl)oxy)ethyl)benzene (5d)

Prepared according to the general procedure A for the Etherification. Following workup, the product was purified by column chromatography (hexane:EtOAc, 20:1) to give the title compound as a colorless oil (isolated yield: 54%, d.r .= 0.8:1, We just isolated this product of a single configuration).

^{1}H NMR (500 MHz, $CDCl_3$) δ 7.36 – 7.26 (m, 4H), 7.24 – 7.20 (m, 1H), 7.20 – 7.11 (m, 2H), 6.94 – 6.87 (m, 2H), 4.68 (q, *J* = 6.5 Hz, 1H), 3.84 (s, 3H), 2.73 (td, *J* = 13.0, 5.1 Hz, 1H), 2.62 (td, *J* = 12.9, 5.1 Hz, 1H), 1.85 (ddd, *J* = 13.8, 12.2, 5.1 Hz, 1H), 1.77 (ddd, *J* = 13.7, 12.3, 5.1 Hz, 1H), 1.42 (d, *J* = 6.5 Hz, 3H), 1.20 (d, *J* = 8.6 Hz, 6H). **^{13}C NMR** (126 MHz, $CDCl_3$) δ 158.3, 143.1, 139.8, 128.4, 128.3, 126.8, 125.6, 113.6, 75.8, 69.3, 55.6, 43.5, 30.6, 27.0, 26.6, 26.6.

HRMS (ESI+), *m/z*: calculated for $C_{20}H_{27}O_2$ $[M + H]^+$: 299.2011, found: 299.2003.

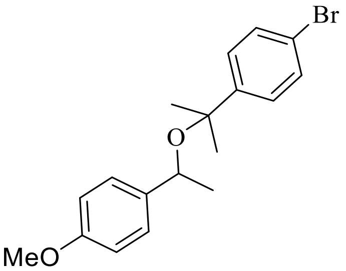

1-Bromo-4-(2-(1-(4-methoxyphenyl)ethoxy)propan-2-yl)benzene (6d)

Prepared according to the general procedure A for the Etherification. Following workup, the product was purified by column chromatography (hexane:EtOAc, 20:1) to give the title compound as a colorless oil (isolated yield: 83%).

^{1}H NMR (500 MHz, $CDCl_3$) δ 7.46 (d, *J* = 8.4 Hz, 2H), 7.34 (d, *J* = 8.4 Hz, 2H), 7.18 (d, *J* = 8.4 Hz, 2H), 6.85 (d, *J* = 8.4 Hz, 2H), 4.26 (q, *J* = 6.4 Hz, 1H), 3.82 (s, 3H), 1.50 (s, 3H), 1.37 (s, 3H), 1.32 (d, *J* = 6.4 Hz, 3H). **^{13}C NMR** (126 MHz, $CDCl_3$) δ 158.3, 146.1, 139.3, 131.1, 128.0, 126.7, 120.8, 113.5, 71.5, 55.2, 31.2, 27.3, 26.5.

HRMS (ESI+), *m/z*: calculated for $C_{18}H_{22}BrO_2$ $[M + H]^+$: 349.0803, found: 349.0828.

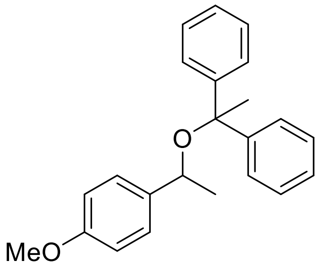

(1-(1-(4-Methoxyphenyl)ethoxy)ethane-1,1-diyl)dibenzene (7d)

Prepared according to the general procedure A for the Etherification. Following workup, the product was purified by column chromatography (hexane:EtOAc, 20:1) to give the title compound as a colorless oil (isolated yield: 55%).

^{1}H NMR (500 MHz, $CDCl_3$) δ 7.49 (d, *J* = 7.7 Hz, 2H), 7.37 (dd, *J* = 16.6, 7.8 Hz, 4H), 7.32 (d, *J* = 7.3 Hz, 1H), 7.29 (d, *J* = 4.4 Hz, 2H), 7.26 (t, *J* = 7.6 Hz, 2H), 7.19 (t, *J* = 7.2 Hz, 1H), 6.87 (d, *J* = 8.6 Hz, 2H), 4.40 (q, *J* = 6.4 Hz, 1H), 3.83 (s, 3H), 1.65 (s, 3H), 1.33 (d, *J* = 6.4 Hz, 3H). **^{13}C NMR** (126 MHz, $CDCl_3$) δ 158.3, 148.7, 146.2, 139.7, 127.9, 127.7, 127.1, 126.8, 126.4, 113.5, 81.8, 71.3, 55.3, 27.6, 26.5.

HRMS (ESI+), *m/z*: calculated for $C_{23}H_{25}O_2$ $[M + H]^+$: 333.1855, found: 333.1851.

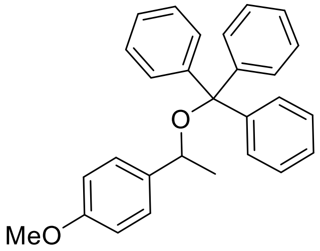

((1-(4-Methoxyphenyl)ethoxy)methanetriyl)tribenzene (8d)

Prepared according to the general procedure A for the Etherification. Following workup, the product was purified by column chromatography (hexane:EtOAc, 20:1) to give the title compound as a colorless oil (isolated yield: 53%).

^{1}H NMR (500 MHz, $CDCl_3$) δ 7.50 (d, *J* = 7.9 Hz, 6H), 7.22 (dt, *J* = 23.0, 7.1 Hz, 9H), 7.09 (d, *J* = 8.4 Hz, 2H), 6.74 (d, *J* = 8.4 Hz, 2H), 4.61 (q, *J* = 6.3 Hz, 1H), 3.80 (s, 3H), 1.02 (d, *J* = 6.3 Hz, 3H). **^{13}C NMR** (126 MHz, $CDCl_3$) δ 157.9, 145.1, 138.8, 129.1, 127.6, 126.8, 126.7, 113.2, 87.7, 72.4, 55.3, 25.8.

HRMS (ESI+), *m/z*: calculated for $C_{28}H_{26}O_2Na$ $[M + Na]^+$: 417.1830, found: 417.1839.

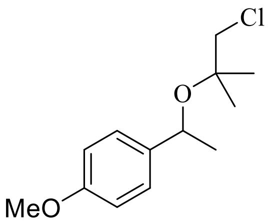

1-(1-((1-Chloro-2-methylpropan-2-yl)oxy)ethyl)-4-methoxybenzene (9d)

Prepared according to the general procedure A for the Etherification. Following workup, the product was purified by column chromatography (hexane:EtOAc, 20:1) to give the title compound as a colorless oil (isolated yield: 81%).

^{1}H NMR (500 MHz, $CDCl_3$) δ 7.29 (d, *J* = 8.6 Hz, 2H), 6.88 (d, *J* = 8.7 Hz, 2H), 4.68 (q, *J* = 6.5 Hz, 1H), 3.82 (s, 3H), 3.50 (d, *J* = 11.1 Hz, 1H), 3.39 (d, *J* = 11.1 Hz, 1H), 1.40 (d, *J* = 6.5 Hz, 3H), 1.25 (s, 3H), 1.22 (s, 3H). **^{13}C NMR** (126 MHz, $CDCl_3$) δ 158.5, 138.9, 126.7, 113.6, 75.9, 70.3, 55.2, 52.6, 26.6, 24.6, 24.4.

HRMS (ESI+), ***m/z***: calculated for $C_{13}H_{20}ClO_2$ $[M + H]^+$: 243.1152, found: 243.1148.

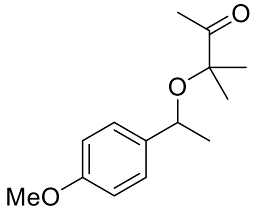

3-(1-(4-Methoxyphenyl)ethoxy)-3-methylbutan-2-one (10d)

Prepared according to the general procedure A for the Etherification. Following workup, the product was purified by column chromatography (hexane:EtOAc, 20:1) to give the title compound as a colorless oil (isolated yield: 66%).

^{1}H NMR (500 MHz, $CDCl_3$) δ 7.24 (d, *J* = 8.6 Hz, 2H), 6.87 (d, *J* = 8.7 Hz, 2H), 4.47 (q, *J* = 6.4 Hz, 1H), 3.82 (s, 3H), 2.16 (s, 3H), 1.42 (d, *J* = 6.5 Hz, 3H), 1.26 (s, 3H), 1.21 (s, 3H). **^{13}C NMR** (126 MHz, $CDCl_3$) δ 212.8, 158.6, 138.2, 126.8, 113.6, 82.4, 72.4, 55.2, 26.3, 24.5, 24.3, 23.3.

HRMS (ESI+), ***m/z***: calculated for $C_{14}H_{20}O_3Na$ $[M + Na]^+$: 259.1310, found: 259.1319.

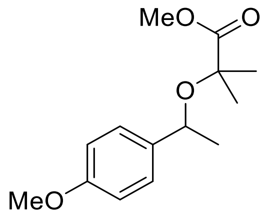

Methyl 2-(1-(4-methoxyphenyl)ethoxy)-2-methylpropanoate (11d)

Prepared according to the general procedure A for the Etherification. Following workup, the product was purified by column chromatography (hexane:EtOAc, 20:1) to give the title compound as a colorless oil (isolated yield: 70%).

^{1}H NMR (500 MHz, $CDCl_3$) δ 7.25 (d, J = 8.4 Hz, 2H), 6.86 (d, J = 8.5 Hz, 2H), 4.55 (q, J = 6.5 Hz, 1H), 3.81 (s, 3H), 3.61 (s, 3H), 1.47 (s, 3H), 1.43 (d, J = 6.4 Hz, 3H), 1.27 (s, 3H). **^{13}C NMR** (126 MHz, $CDCl_3$) δ 175.4, 158.5, 138.1, 126.8, 113.5, 78.1, 73.3, 55.2, 51.9, 26.2, 26.0, 25.1.

HRMS (ESI+), *m/z*: calculated for $C_{14}H_{20}O_4Na$ $[M + Na]^+$: 275.1254, found: 275.1233.

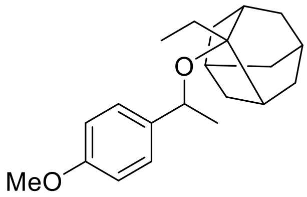

2-Ethyl-2-(1-(4-methoxyphenyl)ethoxy)adamantane (12d)

Prepared according to the general procedure A for the Etherification. Following workup, the product was purified by column chromatography (hexane:EtOAc, 20:1) to give the title compound as a white solid (isolated yield: 76%).

^{1}H NMR (500 MHz, $CDCl_3$) δ 7.30 (d, J = 8.6 Hz, 2H), 6.85 (d, J = 8.6 Hz, 2H), 4.59 (q, J = 6.4 Hz, 1H), 3.81 (s, 3H), 2.21 – 2.16 (m, 3H), 1.80 – 1.76 (m, 2H), 1.73 (dd, J = 4.7, 2.8 Hz, 5H), 1.60 – 1.56 (m, 4H), 1.41 (d, J = 6.4 Hz, 3H), 0.91 (t, J = 7.5 Hz, 5H). **^{13}C NMR** (126 MHz, $CDCl_3$) δ 158.2, 139.6, 127.2, 113.3, 81.0, 74.9, 67.9, 55.2, 38.4, 36.6, 34.6, 34.3, 33.0, 30.5, 27.5, 27.3, 26.5, 24.4, 6.4.

HRMS (ESI+), *m/z*: calculated for $C_{21}H_{31}O_2$ $[M + H]^+$: 315.2324, found: 315.2302.

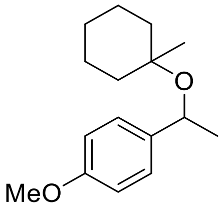

1-Methoxy-4-(1-((1-methylcyclohexyl)oxy)ethyl)benzene (13d)

Prepared according to the general procedure A for the Etherification. Following workup, the product was purified by column chromatography (hexane:EtOAc, 20:1) to give the title compound as a colorless oil (isolated yield: 53%).

^{1}H NMR (500 MHz, $CDCl_3$) δ 7.30 (d, *J* = 8.5 Hz, 2H), 6.87 (d, *J* = 8.7 Hz, 2H), 4.64 (q, *J* = 6.5 Hz, 1H), 3.82 (s, 3H), 1.79 – 1.71 (m, 1H), 1.69 – 1.58 (m, 2H), 1.51 – 1.39 (m, 4H), 1.38 (d, *J* = 6.5 Hz, 3H), 1.35 – 1.22 (m, 3H), 1.06 (s, 3H). **^{13}C NMR** (126 MHz, $CDCl_3$) δ 162.9, 144.6, 131.5, 118.1, 79.8, 73.3, 59.9, 42.3, 41.9, 31.6, 30.6, 30.4, 27.4, 27.2.

HRMS (ESI+), *m/z*: calculated for $C_{16}H_{25}O_2$ $[M + H]^+$: 249.1855, found: 249.1859.

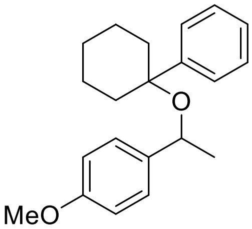

1-Methoxy-4-(1-((1-phenylcyclohexyl)oxy)ethyl)benzene (14d)

Prepared according to the general procedure A for the Etherification. Following workup, the product was purified by column chromatography (hexane:EtOAc, 20:1) to give the title compound as a colorless oil (isolated yield: 80%).

^{1}H NMR (500 MHz, $CDCl_3$) δ 7.48 (d, *J* = 7.7 Hz, 2H), 7.34 (t, *J* = 7.5 Hz, 2H), 7.27 (d, *J* = 7.1 Hz, 1H), 7.15 (d, *J* = 8.6 Hz, 2H), 6.81 (d, *J* = 8.6 Hz, 2H), 4.12 (q, *J* = 6.5 Hz, 1H), 3.81 (s, 3H), 2.11 (dd, *J* = 10.5, 6.6 Hz, 1H), 1.92 – 1.78 (m, 3H), 1.78 – 1.70 (m, 1H), 1.55 – 1.40 (m, 2H), 1.40 – 1.20 (m, 3H), 1.18 (d, *J* = 6.4 Hz, 3H). **^{13}C NMR** (126 MHz, $CDCl_3$) δ 158.2, 145.6, 139.2, 127.8, 127.2, 127.0, 113.3, 70.8, 55.2, 39.0, 34.2, 26.0, 25.7, 22.6, 22.2.

HRMS (ESI+), *m/z*: calculated for $C_{21}H_{27}O_2$ $[M + H]^+$: 311.2011, found: 311.2034.

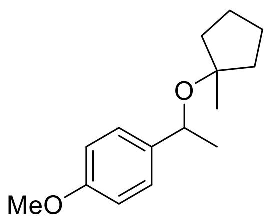

1-Methoxy-4-(1-((1-methylcyclopentyl)oxy)ethyl)benzene (15d)

Prepared according to the general procedure A for the Etherification. Following workup, the product was purified by column chromatography (hexane:EtOAc, 20:1) to give the title compound as a colorless oil (isolated yield: 70%).

^{1}**H NMR** (500 MHz, $CDCl_3$) δ 7.29 (d, *J* = 8.6 Hz, 2H), 6.87 (d, *J* = 8.6 Hz, 2H), 4.58 (q, *J* = 6.5 Hz, 1H), 3.82 (s, 3H), 1.95 – 1.86 (m, 1H), 1.84 – 1.71 (m, 2H), 1.65 – 1.55 (m, 2H), 1.55 – 1.43 (m, 2H), 1.37 (d, *J* = 6.6 Hz, 4H), 1.26 – 1.20 (m, 3H). ^{13}**C NMR** (126 MHz, $CDCl_3$) δ 158.2, 139.6, 126.6, 113.5, 85.6, 70.4, 55.2, 39.0, 38.4, 26.8, 24.9, 23.9, 23.8.

HRMS (ESI+), *m/z*: calculated for $C_{15}H_{23}O_2$ $[M + H]^+$: 235.1698, found: 235.1699.

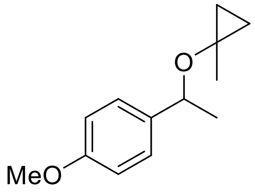

1-Methoxy-4-(1-(1-methylcyclopropoxy)ethyl)benzene (16d)

Prepared according to the general procedure A for the Etherification. Following workup, the product was purified by column chromatography (hexane:EtOAc, 20:1) to give the title compound as a colorless oil (isolated yield: 30%).

^{1}**H NMR** (500 MHz, $CDCl_3$) δ 7.26 (d, *J* = 8.4 Hz, 2H), 6.88 (d, *J* = 8.4 Hz, 2H), 4.65 (q, *J* = 6.6 Hz, 1H), 3.82 (s, 3H), 1.40 (d, *J* = 6.5 Hz, 3H), 1.28 (s, 3H), 0.82 (dt, *J* = 10.9, 5.7 Hz, 1H), 0.67 (dt, *J* = 11.2, 5.7 Hz, 1H), 0.41 – 0.36 (m, 1H), 0.31 – 0.26 (m, 1H). ^{13}**C NMR** (126 MHz, $CDCl_3$) δ 158.6, 137.9, 127.1, 113.6, 75.1, 57.8, 55.3, 25.2, 22.1, 13.7, 13.2.

HRMS (ESI+), *m/z*: calculated for $C_{13}H_{19}O_2$ $[M + H]^+$: 207.1385, found: 207.1383.

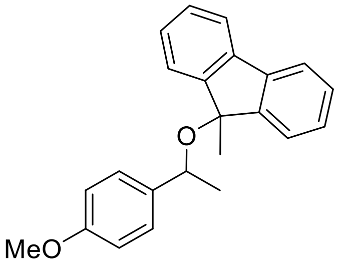

9-(1-(4-Methoxyphenyl)ethoxy)-9-methyl-9*H*-fluorene (17d)

Prepared according to the general procedure A for the Etherification. Following workup, the product was purified by column chromatography (hexane:EtOAc, 20:1) to give the title compound as a colorless oil (isolated yield: 72%).

^{1}H NMR (500 MHz, $CDCl_3$) δ 7.70 (d, *J* = 7.4 Hz, 1H), 7.63 (d, *J* = 7.4 Hz, 1H), 7.59 (d, *J* = 7.5 Hz, 1H), 7.44 (td, *J* = 7.4, 1.3 Hz, 1H), 7.39 (td, *J* = 7.3, 1.2 Hz, 1H), 7.22 (td, *J* = 7.4, 1.2 Hz, 1H), 6.91 (td, *J* = 7.4, 1.1 Hz, 1H), 6.86 (d, *J* = 7.3 Hz, 1H), 6.81 (d, *J* = 8.6 Hz, 2H), 6.65 (d, *J* = 8.7 Hz, 2H), 3.78 (s, 3H), 3.72 (q, *J* = 6.5 Hz, 1H), 1.70 (s, 3H), 1.19 (d, *J* = 6.5 Hz, 3H). **^{13}C NMR** (126 MHz, $CDCl_3$) δ 158.2, 147.7, 147.0, 140.2, 139.3, 138.1, 128.8, 128.2, 127.7, 127.1, 127.0, 124.9, 124.0, 119.8, 119.4, 113.0, 84.7, 72.5, 55.2, 27.3, 25.2.

HRMS (ESI+), *m/z*: calculated for $C_{23}H_{23}O_2$ $[M + H]^+$: 331.1698, found: 331.1677.

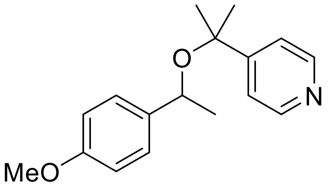

4-(2-(1-(4-Methoxyphenyl)ethoxy)propan-2-yl)pyridine (18d)

Prepared according to the general procedure A for the Etherification. Following workup, the product was purified by column chromatography (hexane:EtOAc, 20:1) to give the title compound as a colorless oil (isolated yield: 69%).

^{1}H NMR (500 MHz, $CDCl_3$) δ 8.57 (d, *J* = 5.6 Hz, 2H), 7.39 – 7.34 (m, 2H), 7.19 (d, *J* = 8.6 Hz, 2H), 6.85 (d, *J* = 8.6 Hz, 2H), 4.30 (q, *J* = 6.4 Hz, 1H), 3.82 (s, 3H), 1.48 (s, 3H), 1.39 (s, 3H), 1.36 (d, *J* = 6.4 Hz, 3H). **^{13}C NMR** (126 MHz, $CDCl_3$) δ 158.4, 156.4, 149.7, 138.9, 126.7, 121.1, 113.6, 71.8, 55.3, 30.4, 27.0, 26.5.

HRMS (ESI+), *m/z*: calculated for $C_{17}H_{22}NO_2$ $[M + H]^+$: 272.1651, found: 272.1635.

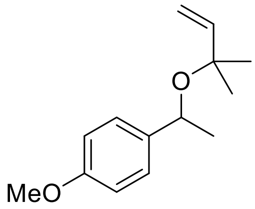

1-Methoxy-4-(1-((2-methylbut-3-en-2-yl)oxy)ethyl)benzene (19d)

Prepared according to the general procedure A for the Etherification. Following workup, the product was purified by column chromatography (hexane:EtOAc, 20:1) to give the title compound as a colorless oil (isolated yield: 69%).

^{1}H NMR (500 MHz, $CDCl_3$) δ 7.26 (d, *J* = 8.6 Hz, 2H), 6.86 (d, *J* = 8.6 Hz, 2H), 5.85 (dd, *J* = 17.6, 10.8 Hz, 1H), 5.17 – 5.04 (m, 2H), 4.53 (q, *J* = 6.5 Hz, 1H), 3.82 (s, 3H), 1.36 (d, *J* = 6.5 Hz, 3H), 1.32 (s, 3H), 1.16 (s, 3H). **^{13}C NMR** (126 MHz, $CDCl_3$) δ 158.2, 144.7, 139.5, 126.7, 113.4, 113.4, 76.4, 70.5, 55.2, 27.5, 26.6, 26.1.

HRMS (ESI+), *m/z*: calculated for $C_{14}H_{21}O_2$ $[M + H]^+$: 221.1542, found: 221.1569.

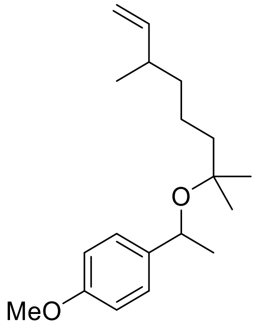

1-(1-((2,6-Dimethyloct-7-en-2-yl)oxy)ethyl)-4-methoxybenzene (20d)

Prepared according to the general procedure A for the Etherification. Following workup, the product was purified by column chromatography (hexane:EtOAc, 20:1) to give the title compound as a colorless oil (isolated yield: 56%).

^{1}H NMR (500 MHz, $CDCl_3$) δ 7.28 (d, *J* = 8.6 Hz, 2H), 6.86 (d, *J* = 8.7 Hz, 2H), 5.69 (dddd, *J* = 17.6, 10.3, 7.6, 6.2 Hz, 1H), 5.00 – 4.88 (m, 2H), 4.61 (q, *J* = 6.5 Hz, 1H), 3.82 (s, 3H), 2.10 (dq, *J* = 10.6, 6.8 Hz, 1H), 1.55 – 1.42 (m, 1H), 1.43 – 1.31 (m, 2H), 1.36 (d, *J* = 6.5 Hz, 4H), 1.31 – 1.13 (m, 3H), 1.11 (s, 3H), 1.09 (d, *J* = 1.8 Hz, 3H), 0.98 (dd, *J* = 6.7, 4.2 Hz, 3H). **^{13}C NMR** (126 MHz, $CDCl_3$) δ 158.2, 144.9, 139.9, 126.7, 113.4, 112.3, 112.3, 76.1, 69.1, 55.2, 41.6, 41.5, 37.8, 37.7, 37.2, 26.8, 26.5, 26.4, 26.4, 21.8, 21.8, 20.2, 20.1.

HRMS (ESI+), *m/z*: calculated for $C_{19}H_{31}O_2$ $[M + H]^+$: 291.2324, found: 291.2331.

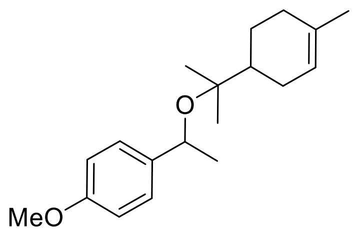

1-Methoxy-4-(1-((2-(4-methylcyclohex-3-en-1-yl)propan-2-yl)oxy)ethyl)benzene (21d)

Prepared according to the general procedure A for the Etherification. Following workup, the product was purified by column chromatography (hexane:EtOAc, 20:1) to give the title compound as a colorless oil (isolated yield: 53%).

^{1}H NMR (500 MHz, $CDCl_3$) δ 7.30 – 7.24 (m, 2H), 6.89 – 6.77 (m, 2H), 5.41 (p, *J* = 2.6 Hz, 1H), 4.66 (dq, *J* = 9.0, 6.5 Hz, 1H), 3.82 (s, 3H), 2.14 – 1.94 (m, 2.5H), 1.91 – 1.74 (m, 2.5H), 1.73 – 1.59 (m, 4H), 1.35 (dd, *J* = 6.5, 1.7 Hz, 3H), 1.25 (dddd, *J* = 24.8, 12.3, 11.0, 5.9 Hz, 1H), 1.12 (d, *J* = 8.7 Hz, 3H), 1.02 (s, 1.5H), 0.98 (s, 1.5zH). **^{13}C NMR** (126 MHz, $CDCl_3$) δ 158.2, 158.1, 140.1, 140.0, 126.7, 126.6, 121.0, 113.4, 78.0, 78.0, 68.9, 68.8, 55.2, 55.2, 43.4, 43.4, 31.2, 31.1, 27.2, 27.0, 26.9, 26.8, 24.2, 23.9, 23.8, 23.8, 23.4, 23.3, 23.1.

HRMS (ESI+), *m/z*: calculated for $C_{19}H_{28}O_2Na$ $[M + Na]^+$: 311.1987, found: 311.1978.

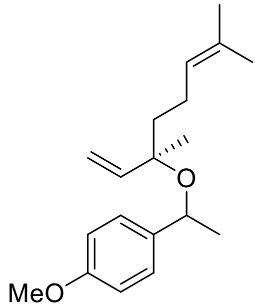

1-(1-(((*R*)-3,7-Dimethylocta-1,6-dien-3-yl)oxy)ethyl)-4-methoxybenzene (22d)

Prepared according to the general procedure A for the Etherification. Following workup, the product was purified by column chromatography (hexane:EtOAc, 20:1) to give the title compound as a colorless oil (isolated yield: 40%, d.r. = 1:1).

^{1}H NMR (500 MHz, $CDCl_3$) δ 7.26 (d, *J* = 8.6 Hz, 2H), 6.86 (d, *J* = 8.6 Hz, 2H), 5.85 (dd, *J* = 17.6, 10.9 Hz, 0.5H), 5.72 (dd, *J* = 17.7, 10.9 Hz, 0.5H), 5.22 – 4.99 (m, 3H), 4.54 (dq, *J* = 10.9, 6.5 Hz, 1H), 3.82 (s, 3H), 2.12 – 1.83 (m, 2H), 1.73 – 1.66 (m, 3H), 1.66 – 1.50 (m, 5H), 1.36 (dd, *J* = 14.4, 6.5 Hz, 3H), 1.29 (s, 1.5H), 1.06 (s, 1.5H). **^{13}C NMR** (126 MHz, $CDCl_3$) δ 158.2, 143.9, 143.8, 139.9, 139.4, 131.3, 131.2, 126.9, 126.7, 124.7, 114.4, 114.1, 113.4, 78.6, 70.3, 70.1, 55.2, 41.5, 40.7, 26.7, 26.6, 25.7, 23.0, 22.7, 22.7, 22.3, 17.7, 17.6.

HRMS (ESI+), *m/z*: calculated for $C_{19}H_{29}O_2$ [M + H]$^+$: 289.2168, found: 289.2162.

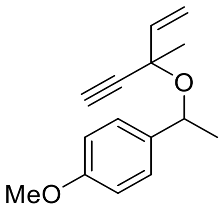

1-Methoxy-4-(1-((3-methylpent-1-en-4-yn-3-yl)oxy)ethyl)benzene (23d)

Prepared according to the general procedure A for the Etherification. Following workup, the product was purified by column chromatography (hexane:EtOAc, 20:1) to give the title compound as a colorless oil (isolated yield: 57%, d.r. = 1.5:1, We just isolated this product of a single configuration).

^{1}H NMR (500 MHz, $CDCl_3$) δ 7.26 – 7.18 (m, 2H), 6.90 – 6.81 (m, 2H), 5.57 – 5.44 (m, 2H), 5.02 (dd, *J* = 8.1, 3.3 Hz, 1H), 4.76 (q, *J* = 6.5 Hz, 1H), 3.82 (s, 3H), 2.62 (s, 1H), 1.58 (s, 3H), 1.47 (d, *J* = 6.5 Hz, 3H). **^{13}C NMR** (126 MHz, $CDCl_3$) δ 158.4, 140.7, 138.1, 127.3, 115.6, 113.4, 83.9, 74.7, 74.4, 72.9, 55.2, 30.0, 25.3.

HRMS (ESI+), *m/z*: calculated for $C_{15}H_{19}O_2$ [M + H]$^+$: 231.1385, found: 231.1388.

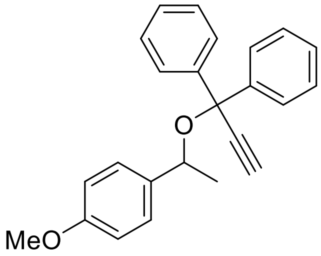

(1-(1-(4-Methoxyphenyl)ethoxy)prop-2-yne-1,1-diyl)dibenzene (24d)

Prepared according to the general procedure A for the Etherification. Following workup, the product was purified by column chromatography (hexane:EtOAc, 20:1) to give the title compound as a colorless oil (isolated yield: 58%).

^{1}H NMR (500 MHz, $CDCl_3$) δ 7.68 (d, *J* = 7.4 Hz, 2H), 7.48 (dd, *J* = 7.8, 1.8 Hz, 2H), 7.38 (t, *J* = 7.6 Hz, 2H), 7.32 (t, *J* = 7.3 Hz, 1H), 7.26 – 7.18 (m, 5H), 6.82 (d, *J* = 8.6 Hz, 2H), 4.81 (q, *J* = 6.5 Hz, 1H), 3.82 (s, 3H), 2.74 (s, 1H), 1.45 (d, *J* = 6.5 Hz, 3H). **^{13}C NMR** (126 MHz, $CDCl_3$) δ 158.4, 143.9, 143.9, 137.8, 128.1, 127.8, 127.7, 127.5, 127.3, 127.1, 127.0, 113.2, 84.2, 79.9, 77.8, 73.7, 55.2, 25.2.

HRMS (ESI+), *m/z*: calculated for $C_{24}H_{23}O_2$ [M + H]$^+$: 343.1698, found: 343.1688.

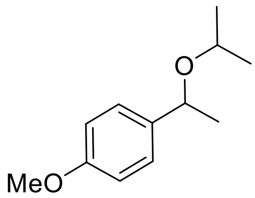

1-(1-Isopropoxyethyl)-4-methoxybenzene (25d)

Prepared according to the general procedure A for the Etherification. Following workup, the product was purified by column chromatography (hexane:EtOAc, 20:1) to give the title compound as a colorless oil (isolated yield: 70%).

^{1}H NMR (500 MHz, $CDCl_3$) δ 7.28 – 7.24 (m, 2H), 6.93 – 6.87 (m, 2H), 4.51 (q, *J* = 6.5 Hz, 1H), 3.83 (s, 3H), 3.49 (hept, *J* = 6.1 Hz, 1H), 1.41 (d, *J* = 6.4 Hz, 3H), 1.16 (d, *J* = 6.0 Hz, 3H), 1.10 (d, *J* = 6.2 Hz, 3H). **^{13}C NMR** (126 MHz, $CDCl_3$) δ 163.5, 141.6, 132.0, 118.4, 72.9, 60.0, 29.5, 28.1, 26.0.

HRMS (ESI+), *m/z*: calculated for $C_{12}H_{19}O_2$ $[M + H]^+$: 195.1385, found: 195.1379.

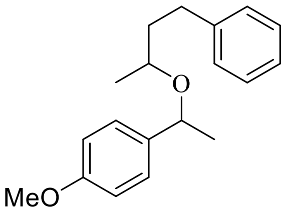

1-Methoxy-4-(1-((4-phenylbutan-2-yl)oxy)ethyl)benzene (26d)

Prepared according to the general procedure A for the Etherification. Following workup, the product was purified by column chromatography (hexane:EtOAc, 20:1) to give the title compound as a colorless oil (isolated yield: 71%, d.r. = 0.9:1).

^{1}H NMR (500 MHz, $CDCl_3$) δ 7.35 – 7.26 (m, 3H), 7.26 – 7.14 (m, 3H), 7.14 – 7.04 (m, 1H), 6.96 – 6.87 (m, 2H), 4.51 (dq, *J* = 16.1, 6.5 Hz, 1H), 3.85 (d, *J* = 5.4 Hz, 3H), 3.47 (h, *J* = 6.1 Hz, 0.5H), 3.37 (dqd, *J* = 7.8, 6.0, 4.7 Hz, 0.5H), 2.77 – 2.67 (m, 1.5H), 2.44 (ddd, *J* = 13.7, 10.4, 5.9 Hz, 0.5H), 1.98 – 1.75 (m, 1.5H), 1.64 (dddd, *J* = 13.7, 10.6, 5.9, 4.7 Hz, 0.5H), 1.46 (dd, *J* = 13.3, 6.5 Hz, 3H), 1.20 (d, *J* = 6.0 Hz, 1.5H), 1.10 (d, *J* = 6.2 Hz, 1.5H). **^{13}C NMR** (126 MHz, $CDCl_3$) δ 163.7, 163.5, 147.3, 147.2, 141.6, 141.1, 133.1, 133.1, 132.9, 132.5, 132.1, 130.4, 130.2, 118.4, 118.4, 79.8, 78.9, 77.0, 76.0, 50.0, 60.0, 44.0, 42.5, 36.8, 36.3, 29.3, 28.9, 25.6, 24.2.

HRMS (ESI+), *m/z*: calculated for $C_{19}H_{25}O_2$ $[M + H]^+$: 285.1855, found: 285.1862.

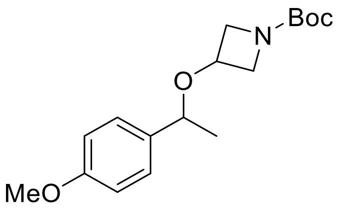

Tert-butyl 3-(1-(4-methoxyphenyl)ethoxy)azetidine-1-carboxylate (27d)

Prepared according to the general procedure A for the Etherification. Following workup, the product was purified by column chromatography (hexane:EtOAc, 20:1) to give the title compound as a colorless oil (isolated yield: 56%).

^{1}H NMR (500 MHz, $CDCl_3$) δ 7.23 (d, *J* = 8.7 Hz, 2H), 6.89 (d, *J* = 8.7 Hz, 2H), 4.35 (q, *J* = 6.5 Hz, 1H), 4.14 (tt, *J* = 6.6, 4.7 Hz, 1H), 4.03 (ddd, *J* = 9.2, 6.5, 1.0 Hz, 1H), 3.88 (dd, *J* = 9.1, 4.7 Hz, 1H), 3.84 – 3.78 (m, 4H), 3.70 – 3.65 (m, 1H), 1.47 (d, *J* = 6.5 Hz, 3H), 1.43 (s, 9H). **^{13}C NMR** (126 MHz, $CDCl_3$) δ 159.3, 156.4, 134.6, 127.6, 113.9, 79.4, 76.7, 65.6, 57.0, 55.3, 28.4, 23.6.

HRMS (ESI+), *m/z*: calculated for $C_{17}H_{26}NO_4$ $[M + H]^+$: 308.1862, found: 308.1838.

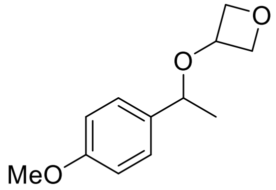

3-(1-(4-Methoxyphenyl)ethoxy)oxetane (28d)

Prepared according to the general procedure A for the Etherification. Following workup, the product was purified by column chromatography (hexane:EtOAc, 20:1) to give the title compound as a colorless oil (isolated yield: 76%).

^{1}H NMR (500 MHz, $CDCl_3$) δ 7.23 (d, *J* = 8.6 Hz, 2H), 6.89 (d, *J* = 8.6 Hz, 2H), 4.73 (t, *J* = 6.5 Hz, 1H), 4.68 (t, *J* = 6.2 Hz, 1H), 4.50 (p, *J* = 6.1 Hz, 1H), 4.44 – 4.39 (m, 2H), 4.35 (q, *J* = 6.5 Hz, 1H), 3.83 (s, 3H), 1.47 (d, *J* = 6.5 Hz, 3H). **^{13}C NMR** (126 MHz, $CDCl_3$) δ 159.3, 134.8, 127.6, 113.9, 79.4, 79.2, 77.0, 70.4, 55.3, 23.5.

HRMS (ESI+), *m/z*: calculated for $C_{12}H_{17}O_3$ $[M + H]^+$: 209.1178, found: 209.1152.

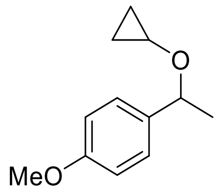

1-(1-Cyclopropoxyethyl)-4-methoxybenzene (29d)

Prepared according to the general procedure A for the Etherification. Following workup, the product was purified by column chromatography (hexane:EtOAc, 20:1) to give the title compound as a colorless oil (isolated yield: 24%).

^{1}H NMR (500 MHz, $CDCl_3$) δ 7.34 – 7.29 (m, 2H), 6.94 – 6.89 (m, 2H), 4.50 (q, *J* = 6.5 Hz, 1H), 3.84 (s, 3H), 3.14 (tt, *J* = 6.1, 3.1 Hz, 1H), 1.44 (d, *J* = 6.5 Hz, 3H), 0.66 – 0.50 (m, 2H), 0.47 – 0.32 (m, 2H). **^{13}C NMR** (126 MHz, $CDCl_3$) δ 159.0, 135.9, 127.6, 113.7, 77.6, 55.3, 5.13, 23.6, 6.0, 5.3.

HRMS (ESI+), *m/z*: calculated for $C_{12}H_{17}O_2$ $[M + H]^+$: 193.1229, found: 193.1233.

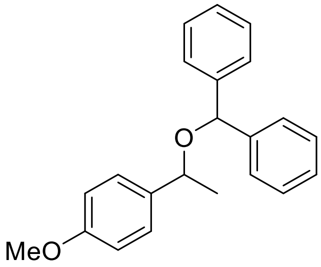

((1-(4-Methoxyphenyl)ethoxy)methylene)dibenzene (30d)

Prepared according to the general procedure A for the Etherification. Following workup, the product was purified by column chromatography (hexane:EtOAc, 20:1) to give the title compound as a colorless oil (isolated yield: 61%).

^{1}H NMR (500 MHz, $CDCl_3$) δ 7.43 – 7.38 (m, 4H), 7.34 (ddd, *J* = 10.8, 5.3, 1.6 Hz, 1H), 7.31 – 7.28 (m, 6H), 7.23 (ddd, *J* = 10.2, 5.8, 3.0 Hz, 1H), 6.94 (d, *J* = 8.6 Hz, 2H), 5.29 (s, 1H), 4.46 (q, *J* = 6.4 Hz, 1H), 3.86 (s, 3H), 1.52 (d, *J* = 6.5 Hz, 3H). **^{13}C NMR** (126 MHz, $CDCl_3$) δ 159.1, 142.9, 142.1, 135.7, 128.5, 128.2, 127.8, 127.6, 127.1, 127.0, 113.9, 79.7, 74.5, 55.3, 24.3.

HRMS (ESI+), *m/z*: calculated for $C_{22}H_{23}O_2$ $[M + H]^+$: 319.1698, found: 319.1686.

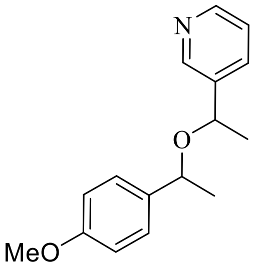

3-(1-(1-(4-Methoxyphenyl)ethoxy)ethyl)pyridine (31d)

Prepared according to the general procedure A for the Etherification. Following workup, the product was purified by column chromatography (hexane:EtOAc, 20:1) to give the title compound as a colorless oil (isolated yield: 69%, d.r. > 20:1, We just isolated this product of a single configuration).

^{1}H NMR (500 MHz, $CDCl_3$) δ 8.57 (dd, *J* = 4.8, 1.7 Hz, 1H), 8.49 (d, *J* = 2.2 Hz, 1H), 7.68 (dt, *J* = 7.8, 2.0 Hz, 1H), 7.33 (ddd, *J* = 7.8, 4.8, 0.9 Hz, 1H), 7.25 – 7.18 (m, 2H), 6.95 – 6.89 (m, 2H), 4.29 (q, *J* = 6.5 Hz, 1H), 4.18 (q, *J* = 6.5 Hz, 1H), 3.85 (s, 3H), 1.40 (dd, *J* = 6.5, 2.3 Hz, 6H). **^{13}C NMR** (126 MHz, $CDCl_3$) δ 159.1, 149.0, 148.4, 139.4, 135.5, 133.9, 127.5, 123.7, 114.0, 74.5, 72.0, 55.3, 24.6, 24.5.

HRMS (ESI+), *m/z*: calculated for $C_{16}H_{20}NO_2$ $[M + H]^+$: 258.1494, found: 258.1489.

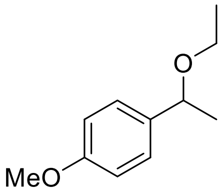

1-(1-Ethoxyethyl)-4-methoxybenzene (32d)

Prepared according to the general procedure A for the Etherification. Following workup, the product was purified by column chromatography (hexane:EtOAc, 20:1) to give the title compound as a yellow oil (isolated yield: 70%).

^{1}H NMR (500 MHz, $CDCl_3$) δ 7.26 (d, *J* = 8.5 Hz, 2H), 6.91 (d, *J* = 8.7 Hz, 2H), 4.39 (q, *J* = 6.5 Hz, 1H), 3.83 (s, 3H), 3.35 (q, *J* = 7.0 Hz, 2H), 1.45 (d, *J* = 6.5 Hz, 3H), 1.20 (t, *J* = 7.0 Hz, 3H). **^{13}C NMR** (126 MHz, $CDCl_3$) δ 158.9, 136.3, 127.3, 113.8, 77.3, 63.7, 55.3, 24.2, 15.4.

HRMS (ESI+), *m/z*: calculated for $C_{11}H_{17}O_2$ $[M + H]^+$: 181.1229, found: 181.1236.

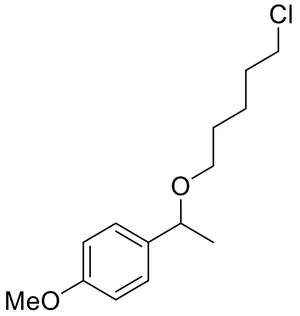

1-(1-((5-Chloropentyl)oxy)ethyl)-4-methoxybenzene (33d)

Prepared according to the general procedure A for the Etherification. Following workup, the product was purified by column chromatography (hexane:EtOAc, 20:1) to give the title compound as a colorless oil (isolated yield: 75%).

^{1}H NMR (500 MHz, $CDCl_3$) δ 7.25 (d, *J* = 8.6 Hz, 2H), 6.91 (d, *J* = 8.7 Hz, 2H), 4.36 (q, *J* = 6.5 Hz, 1H), 3.83 (s, 3H), 3.54 (t, *J* = 6.7 Hz, 2H), 3.29 (td, *J* = 6.4, 1.0 Hz, 2H), 1.78 (dq, *J* = 8.0, 6.8 Hz, 2H), 1.63 – 1.56 (m, 2H), 1.53 – 1.46 (m, 2H), 1.44 (d, *J* = 6.5 Hz, 3H). **^{13}C NMR** (126 MHz, $CDCl_3$) δ 158.9, 136.2, 127.3, 113.8, 77.5, 68.1, 55.3, 45.0, 32.4, 29.2, 24.1, 23.7.

HRMS (ESI+), *m/z*: calculated for $C_{14}H_{22}ClO_2$ $[M + H]^+$: 257.1308, found: 257.1335.

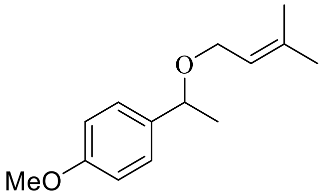

1-Methoxy-4-(1-((3-methylbut-2-en-1-yl)oxy)ethyl)benzene (34d)

Prepared according to the general procedure A for the Etherification. Following workup, the product was purified by column chromatography (hexane:EtOAc, 20:1) to give the title compound as a colorless oil (isolated yield: 62%).

^{1}H NMR (500 MHz, $CDCl_3$) δ 7.30 – 7.25 (m, 2H), 6.94 – 6.88 (m, 2H), 5.38 (tp, *J* = 6.9, 1.4 Hz, 1H), 4.41 (q, *J* = 6.5 Hz, 1H), 3.83 (s, 3H), 3.80 (q, *J* = 7.2 Hz, 2H).1.75 (s, 3H), 1.58 (s, 3H), 1.45 (d, *J* = 6.4 Hz, 3H). **^{13}C NMR** (126 MHz, $CDCl_3$) δ 163.6, 141.6, 140.8, 132.2, 126.0, 118.4, 81.4, 69.5, 60.0, 30.5, 30.0, 22.7.

HRMS (ESI+), *m/z*: calculated for $C_{14}H_{21}O_2$ $[M + H]^+$: 221.1542, found: 221.1535.

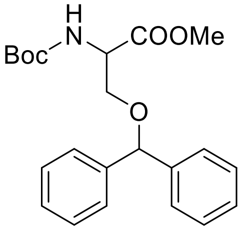

Methyl *O*-benzhydryl-*N*-(*tert*-butoxycarbonyl)serinate (35d)

Prepared according to the general procedure A for the Etherification. Following workup, the product was purified by column chromatography (hexane:EtOAc, 10:1) to give the title compound as a yellow solid (isolated yield: 46%,).

^{1}H NMR (500 MHz, $CDCl_3$) δ 7.35 – 7.32 (m, 5H), 7.29 (dq, *J* = 4.6, 2.5 Hz, 5H), 5.50 (d, *J* = 8.9 Hz, 1H), 5.36 (s, 1H), 4.51 (dt, *J* = 9.1, 3.2 Hz, 1H), 3.89 (dd, *J* = 9.3, 3.2 Hz, 1H), 3.77 (s, 3H), 3.74 – 3.70 (m, 1H), 1.49 (s, 9H). **^{13}C NMR** (126 MHz, $CDCl_3$) δ 171.2, 155.5, 141.6, 141.5, 128.5, 128.4, 127.7, 127.7, 126.9, 126.8, 83.9, 80.0, 69.0, 54.1, 52.4, 28.4.

HRMS (ESI+), *m/z*: calculated for $C_{22}H_{28}NO_5$ $[M + H]^+$: 386.1967, found: 386.1983.

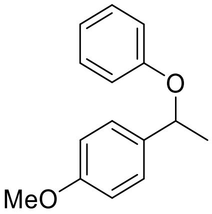

1-Methoxy-4-(1-phenoxyethyl)benzene (36d)

Prepared according to the general procedure A for the Etherification. Following workup, the product was purified by column chromatography (hexane:EtOAc, 20:1) to give the title compound as a colorless oil (isolated yield: 45%).

^{1}H NMR (500 MHz, $CDCl_3$) δ 7.35 – 7.30 (m, 2H), 7.26 – 7.20 (m, 2H), 6.93 – 6.88 (m, 5H), 5.31 (q, *J* = 6.4 Hz, 1H), 3.81 (s, 3H), 1.65 (d, *J* = 6.4 Hz, 3H). **^{13}C NMR** (126 MHz, $CDCl_3$) δ 158.9, 158.0, 135.3, 129.3, 126.8, 120.6, 116.0, 114.0, 75.5, 55.3, 24.5.

HRMS (ESI+), *m/z*: calculated for $C_{15}H_{17}O_2$ $[M + H]^+$: 229.1229, found: 229.1236.

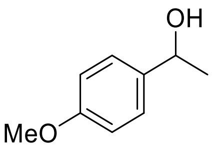

1-(4-Methoxyphenyl)ethan-1-ol (37d)

Prepared according to the general procedure A for the Etherification. Following workup, the product was purified by column chromatography (hexane:EtOAc, 20:1) to give the title compound as a colorless oil (isolated yield: 70%).

^{1}H NMR (500 MHz, $CDCl_3$) δ 7.28 (d, *J* = 8.5 Hz, 2H), 6.91 – 6.85 (m, 2H), 4.81 (q, *J* = 7.5, 6.4 Hz, 1H), 3.80 (s, 3H), 2.44 (s, 1H), 1.46 (d, *J* = 6.4 Hz, 3H). **^{13}C NMR** (126 MHz, $CDCl_3$) δ 163.5, 142.9, 131.4, 118.5, 74.5, 60.0, 29.8.

HRMS (ESI+), *m/z*: calculated for $C_9H_{13}O_2$ $[M + H]^+$: 153.0916, found: 153.0914.

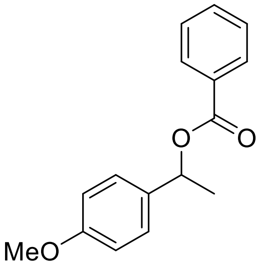

1-(4-Methoxyphenyl)ethyl benzoate (38d)

Prepared according to the general procedure A for the Etherification. Following workup, the product was purified by column chromatography (hexane:EtOAc, 20:1) to give the title compound as a colorless oil (isolated yield: 30%).

^{1}H NMR (500 MHz, $CDCl_3$) δ 8.08 (dd, *J* = 8.4, 1.4 Hz, 2H), 7.57 (ddt, *J* = 7.8, 6.9, 1.3 Hz, 1H), 7.45 (ddt, *J* = 7.8, 6.6, 1.1 Hz, 2H), 7.43 – 7.39 (m, 2H), 6.92 (d, *J* = 8.8 Hz, 2H), 6.12 (q, *J* = 6.6 Hz, 1H), 3.83 (s, 3H), 1.68 (d, *J* = 6.6 Hz, 3H). **^{13}C NMR** (126 MHz, $CDCl_3$) δ 170.6, 164.0, 138.6, 137.5, 135.3, 134.3, 133.0, 132.3, 118.6, 77.4, 60.0, 26.9.

HRMS (ESI+), *m/z*: calculated for $C_{16}H_{17}O_3$ $[M + H]^+$: 257.1178, found: 257.1186.

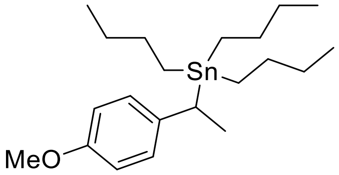

Tributyl(1-(4-methoxyphenyl)ethyl)stannane (39d)

Prepared according to the general procedure A for the Etherification. Following workup, the product was purified by column chromatography (hexane:EtOAc, 20:1) to give the title compound as a colorless oil (isolated yield: 45%).

^{1}H NMR (500 MHz, $CDCl_3$) δ 6.98 (d, *J* = 8.5 Hz, 2H), 6.81 (d, *J* = 8.7 Hz, 2H), 3.80 (s, 3H), 2.66 (q, *J* = 7.6 Hz, 1H), 1.57 (d, *J* = 7.7 Hz, 3H), 1.44 – 1.37 (m, 6H), 1.31 – 1.24 (m, 6.5H), 0.88 (t, *J* = 7.3 Hz, 9.5H), 0.82 – 0.76 (m, 6H). **^{13}C NMR** (126 MHz, $CDCl_3$) δ 156.0, 141.1, 126.4, 113.7, 55.3, 29.1, 27.5, 25.7, 17.9, 13.7, 8.7.

HRMS (ESI+), *m/z*: calculated for $C_{21}H_{39}OSn$ $[M + H]^+$: 427.2023, found: 427.2027.

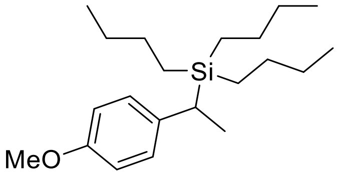

Tributyl(1-(4-methoxyphenyl)ethyl)silane (40d)

Prepared according to the general procedure A for the Etherification. Following workup, the product was purified by column chromatography (hexane:EtOAc, 20:1) to give the title compound as a colorless oil (isolated yield: 25%).

^{1}H NMR (500 MHz, $CDCl_3$) δ 7.01 – 6.94 (m, 2H), 6.82 (d, *J* = 8.7 Hz, 2H), 3.81 (s, 3H), 2.24 (q, *J* = 7.6 Hz, 1H), 1.35 (d, *J* = 7.7 Hz, 3H), 1.30 (q, *J* = 7.5 Hz, 6H), 1.25 – 1.16 (m, 6H), 0.88 (t, *J* = 7.2 Hz, 9H), 0.52 – 0.45 (m, 6H). **^{13}C NMR** (126 MHz, $CDCl_3$) δ 161.3, 143.1, 132.6, 118.2, 60.0, 31.6, 30.9, 30.8, 20.5, 18.5, 15.5.

HRMS (ESI+), *m/z*: calculated for $C_{21}H_{39}OSi$ $[M + H]^+$: 335.2770, found: 335.2759.

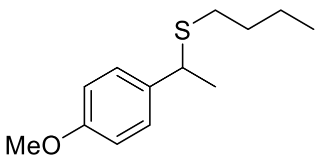

Butyl(1-(4-methoxyphenyl)ethyl)sulfane (41d)

Prepared according to the general procedure A for the Etherification. Following workup, the product was purified by column chromatography (hexane:EtOAc, 20:1) to give the title compound as a colorless oil (isolated yield: 79%).

^{1}H NMR (500 MHz, $CDCl_3$) δ 7.28 (d, *J* = 8.6 Hz, 2H), 6.87 (d, *J* = 8.7 Hz, 2H), 3.95 (q, *J* = 7.0 Hz, 1H), 3.82 (s, 3H), 2.32 (qdd, *J* = 12.5, 8.0, 6.8 Hz, 2H), 1.57 (d, *J* = 7.1 Hz, 3H), 1.54 – 1.44 (m, 2H), 1.35 (dqd, *J* = 14.0, 7.2, 5.5 Hz, 2H), 0.88 (t, *J* = 7.3 Hz, 3H). **^{13}C NMR** (126 MHz, $CDCl_3$) δ 158.5, 136.3, 128.3, 113.8, 55.3, 43.4, 31.5, 30.9, 22.8, 22.1, 13.7.

HRMS (ESI+), *m/z*: calculated for $C_{13}H_{21}OS$ $[M + H]^+$: 225.1313, found: 225.1305.

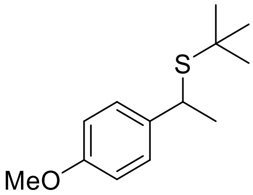

***Tert*-butyl(1-(4-methoxyphenyl)ethyl)sulfane (42d)**

Prepared according to the general procedure A for the Etherification. Following workup, the product was purified by column chromatography (hexane:EtOAc, 20:1) to give the title compound as a colorless oil (isolated yield: 51%).

^{1}H NMR (500 MHz, $CDCl_3$) δ 7.31 (d, *J* = 8.7 Hz, 2H), 6.86 (d, *J* = 8.4 Hz, 2H), 4.04 (q, *J* = 7.1 Hz, 1H), 3.82 (s, 3H), 1.56 (d, *J* = 7.1 Hz, 3H), 1.25 (s, 9H). **^{13}C NMR** (126 MHz, $CDCl_3$) δ 158.3, 138.5, 128.0, 113.8, 55.3, 43.7, 41.8, 31.5, 25.5.

HRMS (ESI+), *m/z*: calculated for $C_{13}H_{20}OSNa$ $[M + Na]^+$: 247.1130, found: 247.1156.

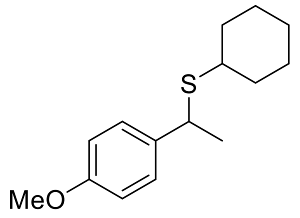

Cyclohexyl(1-(4-methoxyphenyl)ethyl)sulfane (43d)

Prepared according to the general procedure A for the Etherification. Following workup, the product was purified by column chromatography (hexane:EtOAc, 20:1) to give the title compound as a colorless oil (isolated yield: 53%).

^{1}H NMR (500 MHz, $CDCl_3$) δ 7.29 (d, J = 8.6 Hz, 2H), 6.87 (d, J = 8.7 Hz, 2H), 4.04 (d, J = 7.0 Hz, 1H), 3.83 (s, 3H), 2.40 (tt, J = 10.6, 3.6 Hz, 1H), 2.02 – 1.95 (m, 1H), 1.83 – 1.72 (m, 2H), 1.68 (ddt, J = 11.6, 4.6, 1.7 Hz, 1H), 1.63 – 1.55 (m, 1H), 1.55 (d, J = 7.0 Hz, 3H), 1.39 – 1.17 (m, 5H). **^{13}C NMR** (126 MHz, $CDCl_3$) δ 158.4, 136.7, 128.1, 113.8, 55.3, 42.7, 41.8, 33.9, 33.3, 25.9, 23.2.

HRMS (ESI+), *m/z*: calculated for $C_{15}H_{22}OSNa$ $[M + Na]^+$: 273.1289, found: 273.1265.

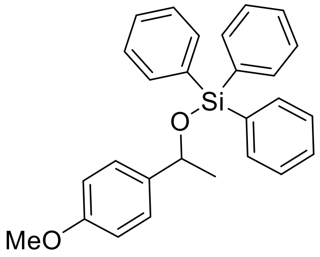

(1-(4-Methoxyphenyl)ethoxy)triphenylsilane (44d)

Prepared according to the general procedure A for the Etherification. Following workup, the product was purified by column chromatography (hexane:EtOAc, 20:1) to give the title compound as a colorless oil (isolated yield: 34%).

^{1}H NMR (500 MHz, $CDCl_3$) δ 7.63 – 7.59 (m, 6H), 7.46 – 7.41 (m, 3H), 7.41 – 7.33 (m, 6H), 7.26 – 7.20 (m, 2H), 6.85 – 6.81 (m, 2H), 5.01 (q, J = 6.3 Hz, 1H), 3.82 (s, 3H), 1.43 (d, J = 6.4 Hz, 3H). **^{13}C NMR** (126 MHz, $CDCl_3$) δ 163.2, 142.9, 140.2, 139.3, 134.6, 132.4, 131.4, 118.2, 76.3, 60.0, 31.5, 5.6.

HRMS (ESI+), *m/z*: calculated for $C_{27}H_{27}O_2Si$ $[M + H]^+$: 411.1780, found: 411.1784.

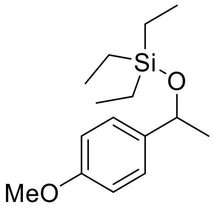

Triethyl(1-(4-methoxyphenyl)ethoxy)silane (45d)

Prepared according to the general procedure A for the Etherification. Following workup, the product was purified by column chromatography (hexane:EtOAc, 20:1) to give the title compound as a colorless oil (isolated yield: 55%).

^{1}**H NMR** (500 MHz, $CDCl_3$) δ 7.29 – 7.27 (m, 2H), 6.90 – 6.79 (m, 2H), 4.85 (q, J = 6.3 Hz, 1H), 3.82 (s, 3H), 1.43 (d, J = 6.3 Hz, 3H), 0.93 (t, J = 7.9 Hz, 9H), 0.65 – 0.50 (m, 6H). ^{13}**C NMR** (126 MHz, $CDCl_3$) δ 158.4, 139.2, 126.4, 113.4, 70.2, 55.2, 27.3, 6.8, 4.8.

HRMS (ESI+), *m/z*: calculated for $C_{15}H_{27}O_2Si$ $[M + H]^+$: 267.1780, found: 267.1782.

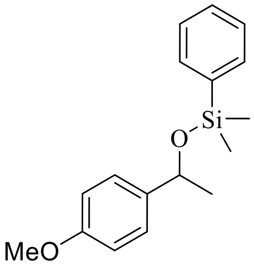

(1-(4-Methoxyphenyl)ethoxy)dimethyl(phenyl)silane (46d)

Prepared according to the general procedure A for the Etherification. Following workup, the product was purified by column chromatography (hexane:EtOAc, 20:1) to give the title compound as a colorless oil (isolated yield: 55%).

^{1}**H NMR** (500 MHz, $CDCl_3$) δ 7.60 – 7.54 (m, 2H), 7.45 – 7.33 (m, 3H), 7.27 – 7.19 (m, 2H), 6.89 – 6.83 (m, 2H), 4.83 (q, J = 6.4 Hz, 1H), 3.82 (s, 3H), 1.42 (d, J = 6.4 Hz, 3H), 0.35 (s, 3H), 0.30 (s, 3H). ^{13}**C NMR** (126 MHz, $CDCl_3$) δ 163.2, 143.2, 142.9, 138.3, 134.2, 132.5, 131.3, 118.2, 75.4, 60.0, 31.5, 3.9, 3.3.

HRMS (ESI+), *m/z*: calculated for $C_{17}H_{23}O_2Si$ $[M + H]^+$: 287.1467, found: 287.1444.

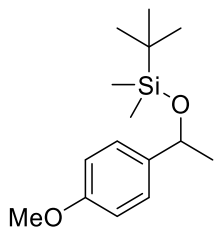

Tert-butyl(1-(4-methoxyphenyl)ethoxy)dimethylsilane (47d)

Prepared according to the general procedure A for the Etherification. Following workup, the product was purified by column chromatography (hexane:EtOAc, 20:1) to give the title compound as a colorless oil (isolated yield: 70%).

^{1}H NMR (500 MHz, $CDCl_3$) δ 7.32 – 7.23 (m, 2H), 6.91 – 6.65 (m, 2H), 4.85 (q, *J* = 6.3 Hz, 1H), 3.83 (s, 3H), 1.41 (d, *J* = 6.4 Hz, 3H), 0.92 (s, 9H), 0.07 (s, 3H), -0.01 (s, 3H). **^{13}C NMR** (126 MHz, $CDCl_3$) δ 158.4, 139.2, 126.3, 113.4, 70.4, 55.2, 27.3, 25.9, 18.3.

HRMS (ESI+), *m/z*: calculated for $C_{15}H_{27}O_2Si$ $[M + H]^+$: 267.1780, found: 267.1779.

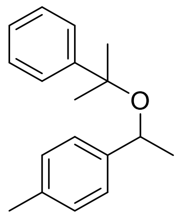

1-Methyl-4-(1-((2-phenylpropan-2-yl)oxy)ethyl)benzene (48d)

Prepared according to the general procedure A for the Etherification. Following workup, the product was purified by column chromatography (hexane:EtOAc, 20:1) to give the title compound as a colorless oil (isolated yield: 69%).

^{1}H NMR (500 MHz, $CDCl_3$) δ 7.52 – 7.49 (m, 2H), 7.40 – 7.35 (m, 2H), 7.32 – 7.28 (m, 1H), 7.20 (d, *J* = 8.1 Hz, 2H), 7.13 (d, *J* = 7.9 Hz, 2H), 4.30 (q, *J* = 6.5 Hz, 1H), 2.36 (s, 3H), 1.54 (s, 3H), 1.41 (s, 3H), 1.34 (d, *J* = 6.5 Hz, 3H). **^{13}C NMR** (126 MHz, $CDCl_3$) δ 147.0, 144.5, 136.0, 128.8, 128.0, 126.9, 126.1, 125.6, 77.9, 71.6, 31.7, 27.0, 26.5, 21.1.

HRMS (ESI+), *m/z*: calculated for $C_{18}H_{23}O$ $[M + H]^+$: 255.1749, found: 255.1745.

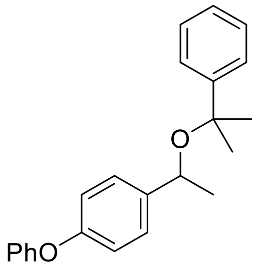

1-Phenoxy-4-(1-((2-phenylpropan-2-yl)oxy)ethyl)benzene (49d)

Prepared according to the general procedure A for the Etherification. Following workup, the product was purified by column chromatography (hexane:EtOAc, 20:1) to give the title compound as a colorless oil (isolated yield: 65%).

^{1}H NMR (500 MHz, $CDCl_3$) δ 7.52 (d, *J* = 7.7 Hz, 2H), 7.38 (td, *J* = 8.3, 7.9, 2.1 Hz, 4H), 7.32 (d, *J* = 7.2 Hz, 1H), 7.27 (d, *J* = 8.5 Hz, 2H), 7.14 (t, *J* = 7.4 Hz, 1H), 7.05 (d, *J* = 7.9 Hz, 2H), 6.97 (d, *J* = 8.5 Hz, 2H), 4.34 (q, *J* = 6.5 Hz, 1H), 1.58 (s, 3H), 1.45 (s, 3H), 1.37 (d, *J* = 6.5 Hz, 3H). **^{13}C NMR** (126 MHz, $CDCl_3$) δ 157.5, 155.7, 146.8, 142.5, 129.7, 128.0, 127.0, 127.0, 126.2, 123.1, 118.7, 118.6, 78.0, 71.4, 31.4, 27.3, 26.5.

HRMS (ESI+), *m/z*: calculated for $C_{23}H_{25}O_2$ $[M + H]^+$: 333.1855, found: 333.1869.

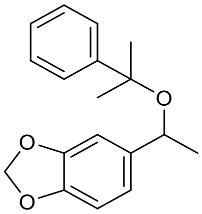

5-(1-((2-Phenylpropan-2-yl)oxy)ethyl)benzo[*d*][1,3]dioxole (50d)

Prepared according to the general procedure A for the Etherification. Following workup, the product was purified by column chromatography (hexane:EtOAc, 20:1) to give the title compound as a colorless oil (isolated yield: 65%).

^{1}H NMR (500 MHz, $CDCl_3$) δ 7.48 (d, *J* = 7.3 Hz, 2H), 7.36 (t, *J* = 7.6 Hz, 2H), 7.30 – 7.26 (m, 1H), 6.87 (d, *J* = 1.5 Hz, 1H), 6.73 (d, *J* = 7.9 Hz, 1H), 6.66 (dd, *J* = 7.9, 1.5 Hz, 1H), 5.97 – 5.95 (m, 2H), 4.23 (q, *J* = 6.5 Hz, 1H), 1.53 (s, 3H), 1.41 (s, 3H), 1.30 (d, *J* = 6.5 Hz, 3H). **^{13}C NMR** (126 MHz, $CDCl_3$) δ 147.4, 146.8, 146.0, 141.8, 128.0, 126.9, 126.1, 118.6, 107.8, 106.4, 100.8, 78.0, 71.6, 31.6, 27.0, 26.5.

HRMS (ESI+), *m/z*: calculated for $C_{18}H_{21}O_3$ $[M + H]^+$: 285.1491, found: 285.1484.

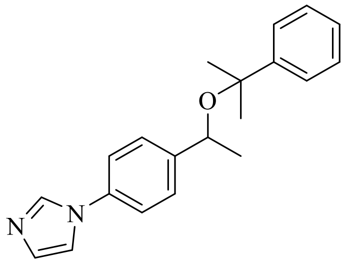

1-(4-(1-((2-Phenylpropan-2-yl)oxy)ethyl)phenyl)-1*H*-imidazole (51d)

Prepared according to the general procedure A for the Etherification. Following workup, the product was purified by column chromatography (hexane:EtOAc, 20:1) to give the title compound as a colorless oil (isolated yield: 70%).

^{1}H NMR (500 MHz, $CDCl_3$) δ 7.87 (s, 1H), 7.52 – 7.45 (m, 2H), 7.41 – 7.38 (m, 2H), 7.35 (dd, *J* = 14.3, 6.5 Hz, 3H), 7.32 – 7.28 (m, 3H), 7.22 (s, 1H), 4.36 (q, *J* = 6.5 Hz, 1H), 1.56 (s, 3H), 1.42 (s, 3H), 1.35 (d, *J* = 6.5 Hz, 3H). **^{13}C NMR** (126 MHz, $CDCl_3$) δ 147.0, 146.4, 135.8, 135.7, 135.6, 130.3, 128.1, 127.1, 126.1, 121.3, 118.4, 78.2, 71.1, 31.2, 27.4 26.4.

HRMS (ESI+), *m/z*: calculated for $C_{20}H_{23}N_2O$ $[M + H]^+$: 307.1810, found: 307.1813.

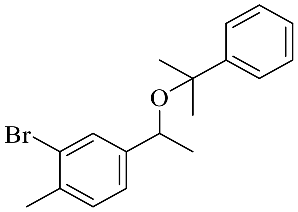

2-Bromo-1-methyl-4-(1-((2-phenylpropan-2-yl)oxy)ethyl)benzene (52d)

Prepared according to the general procedure A for the Etherification. Following workup, the product was purified by column chromatography (hexane:EtOAc, 20:1) to give the title compound as a colorless oil (isolated yield: 56%).

^{1}H NMR (500 MHz, $CDCl_3$) δ 7.50 – 7.45 (m, 3H), 7.39 – 7.34 (m, 2H), 7.32 – 7.28 (m, 1H), 7.17 (d, *J* = 7.8 Hz, 1H), 7.13 (dd, *J* = 7.8, 1.7 Hz, 1H), 4.25 (q, *J* = 6.5 Hz, 1H), 2.40 (s, 3H), 1.41 (s, 3H), 1.31 (d, *J* = 6.5 Hz, 3H). **^{13}C NMR** (126 MHz, $CDCl_3$) δ 151.7, 151.3, 140.5, 135.3, 134.3, 132.8, 131.7, 130.8, 129.3, 129.3, 82.8, 75.7, 36.1, 31.9, 31.1, 27.3.

HRMS (ESI+), *m/z*: calculated for $C_{18}H_{22}BrO$ $[M + H]^+$: 333.0854, found: 333.0857.

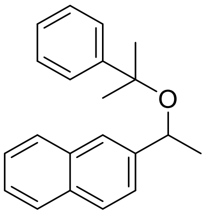

2-(1-((2-Phenylpropan-2-yl)oxy)ethyl)naphthalene (53d)

Prepared according to the general procedure A for the Etherification. Following workup, the product was purified by column chromatography (hexane:EtOAc, 20:1) to give the title compound as a colorless oil (isolated yield: 76%).

^{1}H NMR (500 MHz, $CDCl_3$) δ 7.85 (ddd, *J* = 11.9, 7.5, 2.4 Hz, 3H), 7.72 (d, *J* = 1.6 Hz, 1H), 7.57 – 7.46 (m, 5H), 7.40 (t, *J* = 7.6 Hz, 2H), 7.35 – 7.31 (m, 1H), 4.52 (q, *J* = 6.5 Hz, 1H), 1.61 (s, 3H), 1.44 (d, *J* = 6.8 Hz, 6H). **^{13}C NMR** (126 MHz, $CDCl_3$) δ 146.9, 145.0, 133.4, 132.6, 128.1, 127.9, 127.8, 127.7, 127.0, 126.2, 125.9, 125.4, 124.5, 123.9, 78.2, 72.0, 31.7, 27.2, 26.5.

HRMS (ESI+), *m/z*: calculated for $C_{21}H_{23}O$ $[M + H]^{+}$: 291.1749, found: 291.1746.

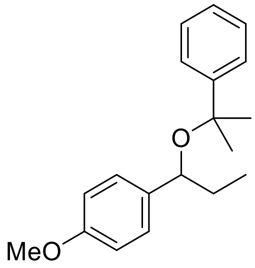

1-Methoxy-4-(1-((2-phenylpropan-2-yl)oxy)propyl)benzene (54d)

Prepared according to the general procedure A for the Etherification. Following workup, the product was purified by column chromatography (hexane:EtOAc, 20:1) to give the title compound as a colorless oil (isolated yield: 65%).

^{1}H NMR (500 MHz, $CDCl_3$) δ 7.49 (d, *J* = 7.5 Hz, 2H), 7.34 (d, *J* = 7.8 Hz, 2H), 7.28 (d, *J* = 5.2 Hz, 1H), 7.17 (d, *J* = 8.6 Hz, 2H), 6.84 (d, *J* = 8.6 Hz, 2H), 4.07 (t, *J* = 6.6 Hz, 1H), 3.83 (s, 3H), 1.76 (tq, *J* = 13.4, 7.4, 6.7 Hz, 1H), 1.61 (dt, *J* = 13.5, 7.3 Hz, 1H), 1.49 (s, 3H), 1.39 (s, 3H), 0.72 (t, *J* = 7.4 Hz, 3H). **^{13}C NMR** (126 MHz, $CDCl_3$) δ 158.3, 147.0, 138.0, 127.9, 127.6, 126.8, 126.1, 113.3, 77.5, 76.4, 55.2, 32.5, 31.0, 27.5, 10.1.

HRMS (ESI+), *m/z*: calculated for $C_{19}H_{24}O_2Na$ $[M + Na]^{+}$: 307.1669, found: 307.1641.

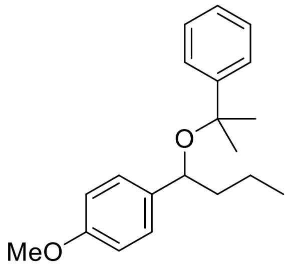

1-Methoxy-4-(1-((2-phenylpropan-2-yl)oxy)butyl)benzene (55d)

Prepared according to the general procedure A for the Etherification. Following workup, the product was purified by column chromatography (hexane:EtOAc, 20:1) to give the title compound as a colorless oil (isolated yield: 76%).

^{1}H NMR (500 MHz, $CDCl_3$) δ 7.52 – 7.46 (m, 2H), 7.37 – 7.32 (m, 2H), 7.30 – 7.25 (m, 1H), 7.20 – 7.14 (m, 2H), 6.86 – 6.82 (m, 2H), 4.15 (t, *J* = 6.7 Hz, 1H), 3.82 (s, 3H), 1.79 – 1.69 (m, 1H), 1.60 – 1.52 (m, 1H), 1.49 (s, 3H), 1.39 (s, 3H), 1.25 – 1.15 (m, 1H), 1.15 – 1.02 (m, 1H), 0.80 (t, *J* = 7.4 Hz, 3H). **^{13}C NMR** (126 MHz, $CDCl_3$) δ 158.3, 146.9, 138.4, 127.9, 127.5, 126.8, 126.2, 113.3, 77.5, 75.0, 55.2, 42.1, 31.0, 27.5, 18.8, 14.0.

HRMS (ESI+), *m/z*: calculated for $C_{20}H_{27}O_2$ $[M + H]^+$: 299.2011, found: 299.2006.

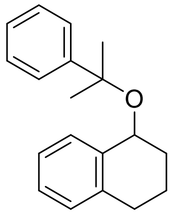

1-((2-Phenylpropan-2-yl)oxy)-1,2,3,4-tetrahydronaphthalene (56d)

Prepared according to the general procedure A for the Etherification. Following workup, the product was purified by column chromatography (hexane:EtOAc, 20:1) to give the title compound as a colorless oil (isolated yield: 55%).

^{1}H NMR (500 MHz, $CDCl_3$) δ 7.63 – 7.59 (m, 2H), 7.39 (dd, *J* = 8.5, 6.7 Hz, 2H), 7.33 – 7.29 (m, 1H), 7.27 – 7.24 (m, 1H), 7.19 – 7.13 (m, 2H), 7.09 – 7.05 (m, 1H), δ 4.58 (t, J = 5.6 Hz, 1H), 2.83 (dt, *J* = 16.5, 6.2 Hz, 1H), 2.68 (dt, *J* = 16.5, 6.4 Hz, 1H), 1.98 (ddt, *J* = 12.2, 5.3, 3.1 Hz, 1H), 1.75 (d, *J* = 2.1 Hz, 6H), 1.69 (dtd, *J* = 10.0, 5.9, 3.5 Hz, 3H). **^{13}C NMR** (126 MHz, $CDCl_3$) δ 146.9, 139.1, 137.6, 128.7, 128.5, 128.0, 127.1, 126.9, 126.3, 125.6, 76.8, 69.4, 31.4, 29.6, 29.0, 28.3, 19.4.

HRMS (ESI+), *m/z*: calculated for $C_{19}H_{23}O$ $[M + H]^+$: 267.1749, found: 267.1744.

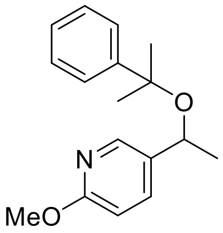

2-Methoxy-5-(1-((2-phenylpropan-2-yl)oxy)ethyl)pyridine (57d)

Prepared according to the general procedure A for the Etherification. Following workup, the product was purified by column chromatography (hexane:EtOAc, 20:1) to give the title compound as a colorless oil (isolated yield: 73%).

^{1}H NMR (500 MHz, $CDCl_3$) δ 7.96 (d, *J* = 2.4 Hz, 1H), 7.57 (dd, *J* = 8.5, 2.4 Hz, 1H), 7.49 – 7.44 (m, 2H), 7.37 – 7.32 (m, 1H), 7.31 – 7.25 (m, 1H), 6.71 (dd, *J* = 8.5, 0.7 Hz, 1H), 4.30 (q, *J* = 6.5 Hz, 1H), 3.94 (s, 3H), 1.41 (s, 3H), 1.32 (d, *J* = 6.5 Hz, 3H). **^{13}C NMR** (126 MHz, $CDCl_3$) δ 167.9, 151.1, 148.7, 141.3, 140.0, 132.8, 131.8, 130.8, 115.2, 82.7, 73.9, 58.1, 35.8, 32.2, 31.0.

HRMS (ESI+), *m/z*: calculated for $C_{17}H_{22}NO_2$ $[M + H]^+$: 272.1651, found: 272.1646.

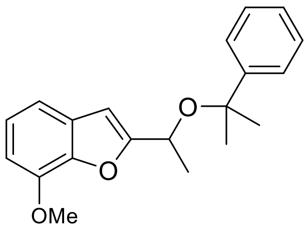

7-Methoxy-2-(1-((2-phenylpropan-2-yl)oxy)ethyl)benzofuran (58d)

Prepared according to the general procedure A for the Etherification. Following workup, the product was purified by column chromatography (hexane:EtOAc, 20:1) to give the title compound as a colorless oil (isolated yield: 56%).

^{1}H NMR (500 MHz, $CDCl_3$) δ 7.52 (d, *J* = 7.3 Hz, 2H), 7.35 (t, *J* = 7.6 Hz, 2H), 7.28 (t, *J* = 7.3 Hz, 1H), 7.14 (d, *J* = 1.8 Hz, 1H), 7.14 (s, 1H), 6.85 – 6.67 (m, 1H), 6.57 (s, 1H), 4.53 (q, *J* = 6.6 Hz, 1H), 4.02 (s, 3H), 1.61 (s, 3H), 1.57 (s, 3H), 1.46 (d, *J* = 6.6 Hz, 3H). **^{13}C NMR** (126 MHz, $CDCl_3$) δ 162.1, 145.9, 145.2, 143.6, 130.3, 128.0, 127.2, 126.2, 123.2, 113.2, 105.9, 102.2, 78.3, 65.7, 56.0, 30.7, 26.9, 22.9.

HRMS (ESI+), *m/z*: calculated for $C_{20}H_{23}O_3$ $[M + H]^+$: 311.1647, found: 311.1649.

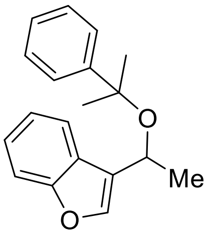

3-(1-((2-Phenylpropan-2-yl)oxy)ethyl)benzofuran (59d)

Prepared according to the general procedure A for the Etherification. Following workup, the product was purified by column chromatography (hexane:EtOAc, 20:1) to give the title compound as a colorless oil (isolated yield: 43%).

^{1}H NMR (500 MHz, $CDCl_3$) δ 7.53 (dt, *J* = 8.5, 1.7 Hz, 3H), 7.48 (dd, *J* = 8.1, 1.0 Hz, 1H), 7.39 – 7.35 (m, 2H), 7.32 – 7.28 (m, 1H), 7.24 (dtd, *J* = 14.7, 7.6, 1.2 Hz, 2H), 6.48 (d, *J* = 0.9 Hz, 1H), 4.51 (q, *J* = 6.6 Hz, 1H), 1.63 (s, 3H), 1.59 (s, 3H), 1.48 (d, *J* = 6.6 Hz, 3H). **^{13}C NMR** (126 MHz, $CDCl_3$) δ 161.3, 154.6, 145.8, 128.5, 128.1, 127.2, 126.3, 123.6, 122.5, 120.8, 111.2, 101.8, 78.3, 65.6, 30.7, 27.1, 22.6.

HRMS (ESI+), *m/z*: calculated for $C_{19}H_{21}O_2$ $[M + H]^+$: 281.1542, found: 281.1539.

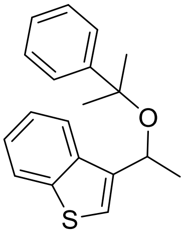

3-(1-((2-Phenylpropan-2-yl)oxy)ethyl)benzo[b]thiophene (60d)

Prepared according to the general procedure A for the Etherification. Following workup, the product was purified by column chromatography (hexane:EtOAc, 20:1) to give the title compound as a colorless oil (isolated yield: 59%).

^{1}H NMR (500 MHz, $CDCl_3$) δ 7.85 (dd, *J* = 8.0, 1.2 Hz, 1H), 7.72 – 7.67 (m, 1H), 7.59 – 7.55 (m, 2H), 7.43 – 7.38 (m, 2H), 7.33 (dqd, *J* = 14.8, 7.3, 1.3 Hz, 3H), 7.02 (s, 1H), 4.71 (q, *J* = 6.4 Hz, 1H), 1.63 (s, 3H), 1.60 (s, 3H), 1.50 (d, *J* = 6.5 Hz, 3H). **^{13}C NMR** (126 MHz, $CDCl_3$) δ 152.8, 146.3, 139.8, 139.3, 128.2, 127.2, 126.2, 124.1, 123.7, 123.2, 122.4, 118.5, 78.6, 68.3, 31.3, 26.6, 26.5.

HRMS (ESI+), *m/z*: calculated for $C_{19}H_{21}OS$ $[M + H]^+$: 297.1313, found: 297.1322.

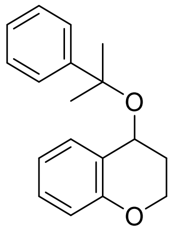

4-((2-Phenylpropan-2-yl)oxy)chromane (61d)

Prepared according to the general procedure A for the Etherification. Following workup, the product was purified by column chromatography (hexane:EtOAc, 20:1) to give the title compound as a colorless oil (isolated yield: 45%).

^{1}H NMR (500 MHz, $CDCl_3$) δ 7.58 (d, *J* = 7.6 Hz, 2H), 7.39 (t, *J* = 7.7 Hz, 2H), 7.32 (t, *J* = 7.3 Hz, 1H), 7.14 (t, *J* = 7.7 Hz, 1H), 6.98 (d, *J* = 7.6 Hz, 1H), 6.82 (t, *J* = 7.4 Hz, 1H), 6.78 (d, *J* = 8.2 Hz, 1H), 4.52 (t, *J* = 4.4 Hz, 1H), 4.35 (td, *J* = 10.3, 3.0 Hz, 1H), 4.16 (ddd, *J* = 10.8, 5.6, 3.6 Hz, 1H), 1.91 – 1.78 (m, 2H), 1.73 (d, *J* = 4.2 Hz, 6H), 1.59 (s, 2H). **^{13}C NMR** (126 MHz, $CDCl_3$) δ 154.8, 146.2, 129.9, 129.0, 128.1, 127.4, 126.5, 124.0, 120.0, 116.7, 77.1, 64.4, 62.6, 30.7, 29.1, 28.8.

HRMS (ESI+), ***m/z***: calculated for $C_{18}H_{21}O_2$ $[M + H]^+$: 269.1542, found: 269.1536.

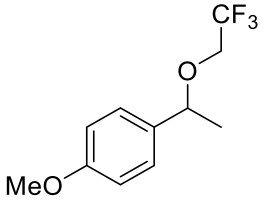

1-Methoxy-4-(1-(2,2,2-trifluoroethoxy)ethyl)benzene (62d)

Prepared according to the general procedure B for the Etherification. Following workup, the product was purified by column chromatography (hexane:EtOAc, 20:1) to give the title compound as a colorless oil (isolated yield: 81%).

^{1}H NMR (500 MHz, $CDCl_3$) δ 7.27 (d, *J* = 8.7 Hz, 2H), 6.93 (d, *J* = 8.7 Hz, 2H), 4.56 (q, *J* = 6.4 Hz, 1H), 3.84 (s, 3H), 3.71 – 3.57 (m, 2H), 1.51 (d, *J* = 6.4 Hz, 3H). **^{13}C NMR** (126 MHz, $CDCl_3$) δ 160.9, 135.1, 129.0, 125.6 (d, $J_{C=F}$ = 278.7 Hz), 115.5, 80.5, 66.9 (q, $J_{C=F}$ = 33.9 Hz), 56.7, 25.2. **^{19}F NMR** (282 MHz, $CDCl_3$) δ -74.08 (t, *J* = 8.8 Hz).

HRMS (ESI+), ***m/z***: calculated for $C_{11}H_{13}F_3O_2Na$ $[M + Na]^+$: 257.0765, found: 257.0744.

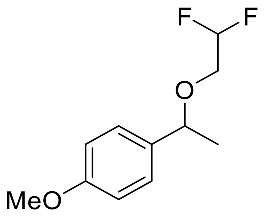

1-(1-(2,2-Difluoroethoxy)ethyl)-4-methoxybenzene (63d)

Prepared according to the general procedure B for the Etherification. Following workup, the product was purified by column chromatography (hexane:EtOAc, 20:1) to give the title compound as a colorless oil (isolated yield: 54%).

^{1}H NMR (500 MHz, $CDCl_3$) δ 7.26 (d, J = 8.7 Hz, 2H), 6.92 (d, J = 8.7 Hz, 2H), 5.84 (tt, J = 55.6, 4.2 Hz, 1H), 4.48 (q, J = 6.4 Hz, 1H), 3.84 (s, 3H), 3.50 (td, J = 14.1, 4.2 Hz, 2H), 1.49 (d, J = 6.4 Hz, 3H). **^{13}C NMR** (126 MHz, $CDCl_3$) δ 159.4, 134.4, 127.6, 114.7 (t, $J_{C\text{=}F}$ = 240.8 Hz), 114.0, 78.8, 67.6, 55.3, 23.7. **^{19}F NMR** (376 MHz, $CDCl_3$) δ -124.93.

HRMS (ESI+), *m/z*: calculated for $C_{11}H_{15}F_2O_2$ $[M + H]^+$: 217.1040, found: 217.1049.

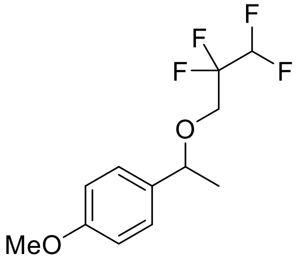

1-Methoxy-4-(1-(2,2,3,3-tetrafluoropropoxy)ethyl)benzene (64d)

Prepared according to the general procedure B for the Etherification. Following workup, the product was purified by column chromatography (hexane:EtOAc, 20:1) to give the title compound as a colorless oil (isolated yield: 48%).

^{1}H NMR (500 MHz, $CDCl_3$) δ 7.24 (d, J = 8.6 Hz, 2H), 6.93 (d, J = 8.7 Hz, 2H), 5.99 (tt, J = 53.3, 5.3 Hz, 1H), 4.48 (q, J = 6.4 Hz, 1H), 3.84 (s, 3H), 3.70 – 3.57 (m, 2H), 1.48 (d, J = 6.4 Hz, 3H). **^{13}C NMR** (126 MHz, $CDCl_3$) δ 159.5, 133.7, 127.5, 114.1, 109.2 (ddd, $J_{C\text{=}F}$ = 249.2, 215.5, 34.1 Hz), 79.2, 65.4 (t, $J_{C\text{=}F}$ = 28.5 Hz), 55.3, 23.6. **^{19}F NMR** (376 MHz, $CDCl_3$) δ -125.54 (d, J = 4.9 Hz), -125.65 (d, J = 4.9 Hz), -140.18 (d, J = 5.5 Hz), -140.36 (t, J = 5.1 Hz).

HRMS (ESI+), *m/z*: calculated for $C_{12}H_{15}F_4O_2$ $[M + H]^+$: 267.1008, found: 267.1036.

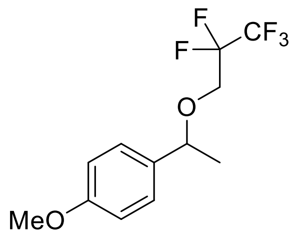

1-Methoxy-4-(1-(2,2,3,3,3-pentafluoropropoxy)ethyl)benzene (65d)

Prepared according to the general procedure B for the Etherification. Following workup, the product was purified by column chromatography (hexane:EtOAc, 20:1) to give the title compound as a yellow oil (isolated yield: 60%).

^{1}H NMR (500 MHz, $CDCl_3$) δ 7.26 (d, *J* = 8.6 Hz, 2H), 6.94 (d, *J* = 8.7 Hz, 2H), 4.53 (q, *J* = 6.4 Hz, 1H), 3.84 (s, 3H), 3.79 – 3.63 (m, 2H), 1.51 (d, *J* = 6.4 Hz, 3H). **^{13}C NMR** (126 MHz, $CDCl_3$) δ 159.5, 133.6, 127.6, 120.5 – 110.5 (m), 114.1, 79.4, 64.9 (t, $J_{C=F}$ = 26.4 Hz), 55.3, 23.6. **^{19}F NMR** (376 MHz, $CDCl_3$) δ -83.52, -123.26 – -123.37 (m).

HRMS (ESI+), *m/z*: calculated for $C_{12}H_{14}F_5O_2$ $[M + H]^+$: 285.0914, found: 285.0926.

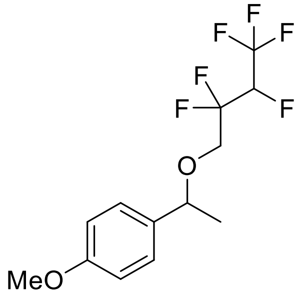

1-(1-(2,2,3,4,4,4-Hexafluorobutoxy)ethyl)-4-methoxybenzene (66d)

Prepared according to the general procedure C for the Etherification. Following workup, the product was purified by column chromatography (hexane:EtOAc, 20:1) to give the title compound as a colorless oil (isolated yield: 40%, d.r. = 1:1).

^{1}H NMR (500 MHz, $CDCl_3$) δ 7.24 (dd, *J* = 8.7, 3.2 Hz, 2H), 6.93 (d, *J* = 8.6 Hz, 2H), 5.22 – 5.13 (m, 0.5H), 5.12 – 5.03 (m, 0.5H), 4.53 – 4.47 (m, 1H), 3.84 (s, 3H), 3.73 – 3.63 (m, 1H), 3.59 (dddd, *J* = 14.8, 11.4, 8.9, 2.9 Hz, 1H), 1.49 (dd, *J* = 6.4, 1.5 Hz, 3H). **^{13}C NMR** (126 MHz, $CDCl_3$) δ 159.5, 133.6 (d, $J_{C=F}$ = 5.9 Hz), 130.7 – 112.8 (m), 127.5, 114.1 (d, $J_{C=F}$ = 2.6 Hz), 84.7 – 83.6 (m), 83.2 – 82.1 (m), 79.3 (d, $J_{C=F}$ = 5.8 Hz), 66.1 (ddd, $J_{C=F}$ = 35.1, 25.8, 11.5 Hz), 55.3, 23.6 (d, $J_{C=F}$ = 7.5 Hz). **^{19}F NMR** (376 MHz, $CDCl_3$) δ -73.94 – -74.09 (m), -116.80 – -116.92 (m), -117.59 (d, *J* = 5.7 Hz), -120.79 – -121.01 (m), -121.53 – -121.70 (m).

HRMS (ESI+), *m/z*: calculated for $C_{13}H_{15}F_6O_2$ $[M + H]^+$: 317.0976, found: 317.0985.

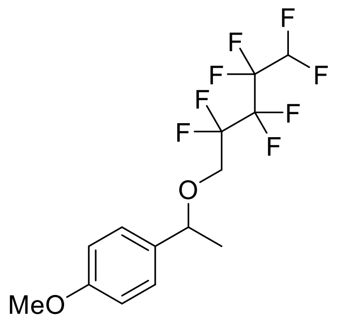

1-Methoxy-4-(1-((2,2,3,3,4,4,5,5-octafluoropentyl)oxy)ethyl)benzene (67d)

Prepared according to the general procedure C for the Etherification. Following workup, the product was purified by column chromatography (hexane:EtOAc, 20:1) to give the title compound as a yellow oil (isolated yield: 59%).

^{1}H NMR (500 MHz, $CDCl_3$) δ 7.26 (d, *J* = 8.6 Hz, 2H), 6.94 (d, *J* = 8.6 Hz, 2H), 6.09 (tt, *J* = 52.0, 5.6 Hz, 1H), 4.53 (q, *J* = 6.4 Hz, 1H), 3.84 (s, 3H), 3.82 – 3.67 (m, 2H), 1.51 (d, *J* = 6.4 Hz, 3H). **^{13}C NMR** (126 MHz, $CDCl_3$) δ 159.5, 133.5, 127.6, 114.1, 110.3 – 105.0(m), 79.6, 64.9 (t, $J_{C=F}$ = 25.6 Hz), 55.3, 23.6. **^{19}F NMR** (376 MHz, $CDCl_3$) δ -119.72 (t, *J* = 10.9 Hz), -125.77 (t, *J* = 8.3 Hz), -130.38 – -130.81 (m), -137.39 – -137.72 (m).

HRMS (ESI+), *m/z*: calculated for $C_{14}H_{15}F_8O_2$ $[M + H]^+$: 367.0944, found: 367.0969.

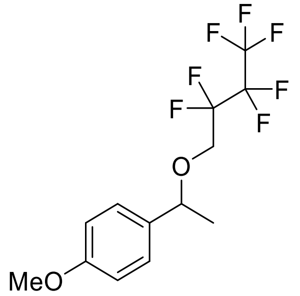

1-(1-(2,2,3,3,4,4,4-Heptafluorobutoxy)ethyl)-4-methoxybenzene (68d)

Prepared according to the general procedure C for the Etherification. Following workup, the product was purified by column chromatography (hexane:EtOAc, 20:1) to give the title compound as a colorless oil (isolated yield: 46%).

^{1}H NMR (500 MHz, $CDCl_3$) δ 7.27 (d, *J* = 8.6 Hz, 2H), 6.93 (d, *J* = 8.7 Hz, 2H), 4.53 (q, *J* = 6.4 Hz, 1H), 3.84 (s, 3H), 3.82 – 3.66 (m, 2H), 1.50 (d, *J* = 6.4 Hz, 3H). **^{13}C NMR** (126 MHz, $CDCl_3$) δ 159.5, 133.6, 127.6, 118.9, 117.2 – 116.6 (m), 115.4 – 114.8 (m), 79.5, 64.8 (t, *J* = 25.9 Hz), 55.3, 23.6. **^{19}F NMR** (376 MHz, $CDCl_3$) δ -80.96, -120.41, -127.55.

HRMS (ESI+), *m/z*: calculated for $C_{13}H_{14}F_7O_2$ $[M + H]^+$: 335.0882, found: 335.0879.

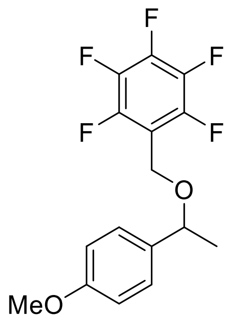

1,2,3,4,5-Pentafluoro-6-((1-(4-methoxyphenyl)ethoxy)methyl)benzene (69d)

Prepared according to the general procedure B for the Etherification. Following workup, the product was purified by column chromatography (hexane:EtOAc, 20:1) to give the title compound as a white solid (isolated yield: 45%).

^{1}H NMR (500 MHz, $CDCl_3$) δ 7.30 (d, J = 8.6 Hz, 2H), 6.93 (d, J = 8.7 Hz, 2H), 4.50 (q, J = 6.4 Hz, 1H), 4.47 – 4.38 (m, 2H), 3.85 (s, 3H), 1.46 (d, J = 6.5 Hz, 3H). **^{13}C NMR** (126 MHz, $CDCl_3$) δ 159.3, 145.6 (d, $J_{C\text{-}F}$ = 249.6 Hz), 141.2 (d, $J_{C\text{-}F}$ = 254.0 Hz), 137.4 (d, $J_{C\text{-}F}$ = 251.5 Hz), 134.7, 127.5, 113.9, 111.8 (t, $J_{C\text{-}F}$ = 18.6 Hz), 78.3, 57.3, 55.3, 24.1. **^{19}F NMR** (376 MHz, $CDCl_3$) δ -142.82 (dd, J = 22.5, 8.9 Hz), -154.43 (t, J = 20.7 Hz), -162.37 (td, J = 21.9, 8.8 Hz).

HRMS (ESI+), *m/z*: calculated for $C_{16}H_{14}F_5O_2$ $[M + H]^+$: 333.0914, found: 333.0926.

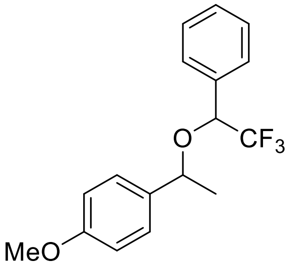

1-Methoxy-4-(1-(2,2,2-trifluoro-1-phenylethoxy)ethyl)benzene (70d)

Prepared according to the general procedure B for the Etherification. Following workup, the product was purified by column chromatography (hexane:EtOAc, 20:1) to give the title compound as a colorless oil (isolated yield: 79%, d.r. = 1.2:1).

^{1}H NMR (500 MHz, $CDCl_3$) δ 7.45 (m, 2.7H), 7.36 (s, 2.2H), 7.24 (d, J = 8.6 Hz, 1H), 7.19 – 7.15 (m, 1H), 6.95 (d, J = 8.7 Hz, 1H), 6.82 (d, J = 8.7 Hz, 1H), 4.79 (q, J = 6.4 Hz, 0.4H), 4.64 (q, J = 6.8 Hz, 0.4H), 4.46 (q, J = 6.9 Hz, 0.6H), 4.33 (q, J = 6.5 Hz, 0.6H), 3.86 (s, 1.6H), 3.80 (s, 1.3H), 1.56 (d, J = 6.4 Hz, 1.3H), 1.51 (d, J = 6.5 Hz, 1.6H). **^{13}C NMR** (126 MHz, $CDCl_3$) δ 159.6, 159.2, 134.3, 133.8, 133.5, 133.0, 129.5, 129.0, 128.7, 128.6, 128.3, 128.1, 128.0, 127.7, 126.0 – 122.5

(m), 114.1, 113.8, 78.3, 76.5 (dd, $J_{C\text{=}F}$ = 68.2, 30.7 Hz), 55.3, 55.2, 24.2, 23.2. ^{19}**F NMR** (376 MHz, $CDCl_3$) δ -75.75, -76.43.

HRMS (ESI+), *m/z*: calculated for $C_{17}H_{18}F_3O_2$ $[M + H]^+$: 311.1259, found: 311.1264.

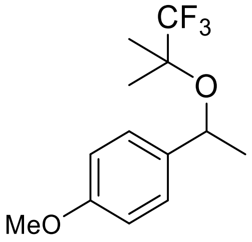

1-Methoxy-4-(1-((1,1,1-trifluoro-2-methylpropan-2-yl)oxy)ethyl)benzene (71d)

Prepared according to the general procedure B for the Etherification. Following workup, the product was purified by column chromatography (hexane:EtOAc, 20:1) to give the title compound as a colorless oil (isolated yield: 60%).

^{1}**H NMR** (500 MHz, $CDCl_3$) δ 7.28 – 7.24 (m, 2H), 6.91 – 6.84 (m, 2H), 4.83 (q, *J* = 6.5 Hz, 1H), 3.83 (s, 3H), 1.41 (d, *J* = 6.5 Hz, 3H), 1.32 (s, 3H), 1.27 (s, 3H). ^{13}**C NMR** (126 MHz, $CDCl_3$) δ 158.6, 138.3, 129.9, 127.6, 126.7, 126.5, 125.3, 113.7, 71.7, 55.2, 26.1, 22.1, 19.8. ^{19}**F NMR** (376 MHz, $CDCl_3$) δ -81.82.

HRMS (ESI+), *m/z*: calculated for $C_{13}H_{18}F_3O_2$ $[M + H]^+$: 263.1259, found: 263.1272.

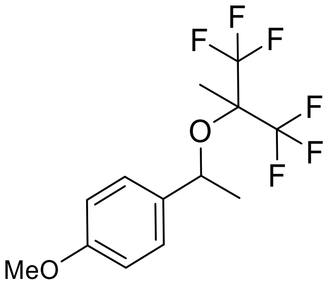

1-(1-((1,1,1,3,3,3-Hexafluoro-2-methylpropan-2-yl)oxy)ethyl)-4-methoxybenzene (72d)

Prepared according to the general procedure B for the Etherification. Following workup, the product was purified by column chromatography (hexane:EtOAc, 20:1) to give the title compound as a colorless oil (isolated yield: 57%).

^{1}**H NMR** (500 MHz, $CDCl_3$) δ 7.26 (d, *J* = 8.6 Hz, 2H), 6.91 (d, *J* = 8.6 Hz, 2H), 4.99 (q, *J* = 6.5 Hz, 1H), 3.83 (s, 3H), 1.49 (d, *J* = 6.5 Hz, 3H), 1.46 (s, 3H). ^{13}**C NMR** (126 MHz, $CDCl_3$) δ 159.0, 136.3, 126.5, 127.1 – 121.1 (m), 113.9, 78.6 (dt, $J_{C\text{=}F}$ = 58.1, 29.1 Hz), 74.5, 55.2, 25.6, 12.9. ^{19}**F NMR** (376 MHz, $CDCl_3$) δ -77.07 (q, $J_{C\text{=}F}$ = 9.7 Hz), -77.86 (q, $J_{C\text{=}F}$ = 9.7 Hz).

HRMS (ESI+), *m/z*: calculated for $C_{13}H_{15}F_6O_2$ $[M + H]^+$: 317.0976, found: 317.0984.

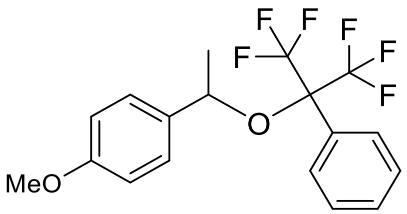

1-(1-((1,1,1,3,3,3-Hexafluoro-2-phenylpropan-2-yl)oxy)ethyl)-4-methoxybenzene (73d)

Prepared according to the general procedure B for the Etherification. Following workup, the product was purified by column chromatography (hexane:EtOAc, 20:1) to give the title compound as a colorless oil (isolated yield: 32%).

^{1}H NMR (500 MHz, $CDCl_3$) δ 7.52 (d, *J* = 8.0 Hz, 2H), 7.47 – 7.40 (m, 1H), 7.36 (dd, *J* = 8.5, 7.2 Hz, 2H), 7.18 (d, *J* = 8.6 Hz, 2H), 6.89 (d, *J* = 8.7 Hz, 2H), 4.73 (q, *J* = 6.4 Hz, 1H), 3.85 (s, 3H), 1.53 (d, *J* = 6.4 Hz, 3H). **^{13}C NMR** (126 MHz, $CDCl_3$) δ 158.8, 136.2, 130.2, 128.8, 128.2, 126.8, 124.2, 123.6, 121.9, 121.3, 113.6, 76.1, 55.2, 25.4. **^{19}F NMR** (376 MHz, $CDCl_3$) δ -69.49 (q, $J_{C=F}$ = 10.7 Hz), -71.67 (q, $J_{C=F}$ = 10.7 Hz).

HRMS (ESI+), *m/z*: calculated for $C_{18}H_{17}F_6O_2$ $[M + H]^+$: 379.1133, found: 379.1139.

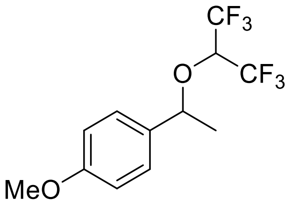

1-(1-((1,1,1,3,3,3-Hexafluoropropan-2-yl)oxy)ethyl)-4-methoxybenzene (74d)

Prepared according to the general procedure B for the Etherification. Following workup, the product was purified by column chromatography (hexane:EtOAc, 20:1) to give the title compound as a colorless oil (isolated yield: 90%).

^{1}H NMR (500 MHz, $CDCl_3$) δ 7.30 (d, *J* = 8.7 Hz, 2H), 6.95 (d, *J* = 8.8 Hz, 2H), 4.83 (q, *J* = 6.5 Hz, 1H), 4.00 (p, *J* = 6.0 Hz, 1H), 3.85 (s, 3H), 1.60 (d, *J* = 6.5 Hz, 3H). **^{13}C NMR** (126 MHz, $CDCl_3$) δ 160.1, 131.3, 128.7, 121.7 (qd, $J_{C=F}$ = 284.0, 282.2, 136.3 Hz), 114.1, 80.9, 72.4 (p, $J_{C=F}$ = 32.3 Hz), 55.2, 23.0. **^{19}F NMR** (282 MHz, $CDCl_3$) δ -72.91 (p, *J* = 8.8 Hz), -73.80 (p, *J* = 8.9 Hz).

HRMS (ESI+), *m/z*: calculated for $C_{12}H_{13}F_6O_2$ $[M + H]^+$: 303.0820, found: 303.0835.

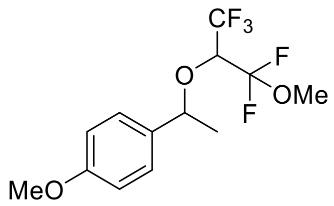

1-Methoxy-4-(1-((1,1,1,3,3-pentafluoro-3-methoxypropan-2-yl)oxy)ethyl)benzene (74e)

Following workup, the product was purified by column chromatography (hexane:EtOAc, 20:1) to give the title compound as a colorless oil (isolated yield: 33%).

^{1}H NMR (500 MHz, $CDCl_3$) δ 7.31 – 7.27 (m, 2H), 6.94 – 6.88 (m, 1H), 4.84 (q, *J* = 6.5 Hz, 0.5H), 4.79 (q, *J* = 6.5 Hz, 0.5H), 3.92 – 3.85 (m, 1H), 3.84 (d, *J* = 0.9 Hz, 3H), 3.67 (s, 1.5H), 3.57 (s, 1.5H), 1.57 (d, *J* = 2.5 Hz, 1.5H), 1.55 (d, *J* = 2.5 Hz, 1.5H). **^{13}C NMR** (126 MHz, $CDCl_3$) δ 159.8, 159.8, 132.3, 132.2, 128.7, 128.6, 114.0, 113.9, 80.4, 80.3, 73.9 (dt, $J_{C\text{=}F}$ = 63.0, 31.6 Hz), 55.3, 50.5 (q, $J_{C\text{=}F}$ = 8.0 Hz), 30.8 (d, $J_{C\text{=}F}$ = 156.6 Hz), 23.2, 23.1. **^{19}F NMR** (376 MHz, $CDCl_3$) δ -72.27 (t, *J* = 9.7 Hz), -73.19 (t, *J* = 9.9 Hz), -81.00 (dq, *J* = 13.8, 9.8 Hz), -81.39 (dq, *J* = 14.6, 9.8 Hz), -82.50 (q, *J* = 9.9 Hz), -82.88 (q, *J* = 9.8 Hz), -83.52 (q, *J* = 9.5 Hz), -83.90 (q, *J* = 9.5 Hz).

HRMS (ESI+), *m/z*: calculated for $C_{13}H_{16}F_5O_3$ $[M + H]^+$: 315.1020, found: 315.1029.

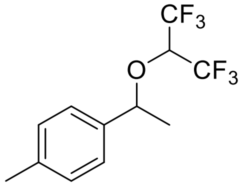

1-(1-((1,1,1,3,3,3-Hexafluoropropan-2-yl)oxy)ethyl)-4-methylbenzene (75d)

Prepared according to the general procedure B for the Etherification. Following workup, the product was purified by column chromatography (hexane:EtOAc, 20:1) to give the title compound as a colorless oil (isolated yield: 50%).

^{1}H NMR (500 MHz, $CDCl_3$) δ 7.24 (q, *J* = 8.1 Hz, 4H), 4.84 (q, *J* = 6.4 Hz, 1H), 4.00 (hept, *J* = 6.0 Hz, 1H), 2.40 (s, 3H), 1.59 (d, *J* = 6.5 Hz, 3H). **^{13}C NMR** (126 MHz, $CDCl_3$) δ 138.9, 136.4, 129.5, 127.3, 124.2 – 119.0 (m), 81.1, 72.6 (p, $J_{C\text{=}F}$ = 32.0 Hz), 23.1, 21.2. **^{19}F NMR** (282 MHz, $CDCl_3$) δ -72.85 (p, *J* = 8.9 Hz), -73.72 (p, *J* = 9.1 Hz).

HRMS (ESI+), *m/z*: calculated for $C_{12}H_{13}F_6O$ $[M + H]^+$: 287.0871, found: 287.0868.

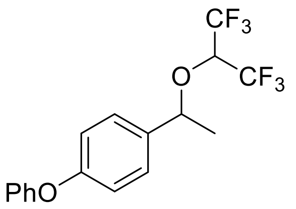

1-(1-((1,1,1,3,3,3-Hexafluoropropan-2-yl)oxy)ethyl)-4-phenoxybenzene (76d)

Prepared according to the general procedure B for the Etherification. Following workup, the product was purified by column chromatography (hexane:EtOAc, 20:1) to give the title compound as a colorless oil (isolated yield: 77%).

^{1}H NMR (500 MHz, $CDCl_3$) δ 7.44 – 7.38 (m, 2H), 7.34 (d, *J* = 8.6 Hz, 2H), 7.18 (tt, *J* = 7.4, 1.1 Hz, 1H), 7.10 – 7.02 (m, 4H), 4.86 (q, *J* = 6.4 Hz, 1H), 4.04 (hept, *J* = 6.0 Hz, 1H), 1.62 (d, *J* = 6.5 Hz, 3H). **^{13}C NMR** (126 MHz, $CDCl_3$) δ 158.1, 156.6, 133.9, 129.9, 128.8, 123.8, 123.5 – 120.0 (m), 119.4, 118.6, 80.9, 72.7 (p, $J_{C\text{=}F}$ = 32.1 Hz), 23.1. **^{19}F NMR** (282 MHz, $CDCl_3$) δ -72.93 (p, *J* = 8.8 Hz), -73.70 (p, *J* = 9.0 Hz).

HRMS (ESI+), *m/z*: calculated for $C_{17}H_{15}F_6O_2$ $[M + H]^+$: 365.0976, found: 365.0989.

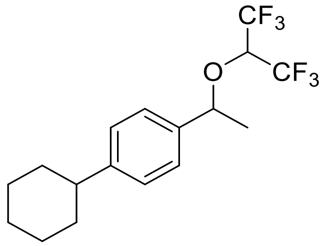

1-cyclohexyl-4-(1-((1,1,1,3,3,3-Hexafluoropropan-2-yl)oxy)ethyl)benzene (77d)

Prepared according to the general procedure B for the Etherification. Following workup, the product was purified by column chromatography (hexane:EtOAc, 20:1) to give the title compound as a white solid (isolated yield: 76%).

^{1}H NMR (500 MHz, $CDCl_3$) δ 7.30 – 7.25 (m, 4H), 4.85 (q, *J* = 6.4 Hz, 1H), 4.02 (hept, *J* = 6.0 Hz, 1H), 2.55 (ddt, *J* = 11.2, 6.5, 3.5 Hz, 1H), 1.94 – 1.85 (m, 4H), 1.79 (ddt, *J* = 12.4, 3.2, 1.6 Hz, 1H), 1.60 (d, *J* = 6.4 Hz, 3H), 1.50 – 1.38 (m, 4H), 1.34 – 1.24 (m, 1H). **^{13}C NMR** (126 MHz, $CDCl_3$) δ 149.0, 136.7, 127.2, 120.6 (td, $J_{C\text{=}F}$ = 283.9, 283.3, 131.2 Hz), 81.1, 72.6 (p, $J_{C\text{=}F}$ = 31.7 Hz), 44.3, 34.4 (d, $J_{C\text{=}F}$ = 8.5 Hz), 26.9, 26.1, 23.1. **^{19}F NMR** (282 MHz, $CDCl_3$) δ -72.82 (p, *J* = 8.8 Hz), -73.69 (p, *J* = 8.9 Hz).

HRMS (ESI+), *m/z*: calculated for $C_{17}H_{21}F_6O$ $[M + H]^+$: 355.1497, found: 355.1476.

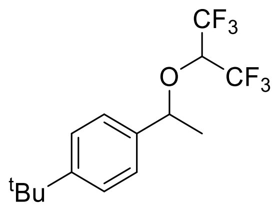

1-(*Tert*-butyl)-4-(1-((1,1,1,3,3,3-hexafluoropropan-2-yl)oxy)ethyl)benzene (78d)

Prepared according to the general procedure B for the Etherification. Following workup, the product was purified by column chromatography (hexane:EtOAc, 20:1) to give the title compound as a colorless oil (isolated yield: 77%).

^{1}H NMR (500 MHz, $CDCl_3$) δ 7.43 (d, *J* = 8.3 Hz, 2H), 7.29 (d, *J* = 8.3 Hz, 2H), 4.86 (q, *J* = 6.5 Hz, 1H), 4.02 (hept, *J* = 6.0 Hz, 1H), 1.60 (d, *J* = 6.5 Hz, 3H), 1.36 (s, 9H). **^{13}C NMR** (126 MHz, $CDCl_3$) δ 152.1, 136.3, 127.0, 125.7, 124.8 – 118.5 (m), 81.0, 72.6 (dt, $J_{C=F}$ = 64.4, 32.1 Hz), 34.7, 31.3, 23.1. **^{19}F NMR** (282 MHz, $CDCl_3$) δ -72.79 (p, *J* = 8.9 Hz), -73.66 (p, *J* = 9.0 Hz).

HRMS (ESI+), *m/z*: calculated for $C_{15}H_{19}F_6O$ $[M + H]^+$: 329.1340, found: 329.1369.

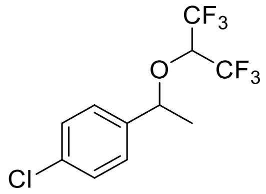

1-Chloro-4-(1-((1,1,1,3,3,3-hexafluoropropan-2-yl)oxy)ethyl)benzene (79d)

Prepared according to the general procedure B for the Etherification. Following workup, the product was purified by column chromatography (hexane:EtOAc, 20:1) to give the title compound as a colorless oil (isolated yield: 47%).

^{1}H NMR (500 MHz, $CDCl_3CDCl_3$) δ 7.41 – 7.37 (m, 2H), 7.32 – 7.29 (m, 2H), 4.83 (q, *J* = 6.4 Hz, 1H), 3.99 (hept, *J* = 5.9 Hz, 1H), 1.58 (d, *J* = 6.5 Hz, 3H). **^{13}C NMR** (126 MHz, $CDCl_3$) δ 139.6, 136.2, 130.5, 129.8, 126.3 – 121.1 (m), 82.2, 75.0 – 74.0 (m), 24.6. **^{19}F NMR** (282 MHz, $CDCl_3$) δ -73.02 (p, *J* = 8.9 Hz), -73.70 (p, *J* = 9.0 Hz).

HRMS (ESI+), *m/z*: calculated for $C_{11}H_{10}ClF_6O$ $[M + H]^+$: 307.0324, found: 307.0326.

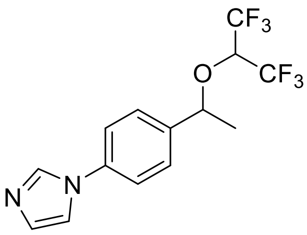

1-(4-(1-((1,1,1,3,3,3-Hexafluoropropan-2-yl)oxy)ethyl)phenyl)-1*H*-imidazole (80d)

Prepared according to the general procedure B for the Etherification. Following workup, the product was purified by column chromatography (hexane:EtOAc, 20:1) to give the title compound as a colorless oil (isolated yield: 62%).

^{1}H NMR (500 MHz, $CDCl_3$) δ 7.90 (d, J = 1.1 Hz, 1H), 7.49 (d, J = 8.5 Hz, 2H), 7.46 – 7.41 (m, 2H), 7.32 (t, J = 1.4 Hz, 1H), 7.24 (t, J = 1.2 Hz, 1H), 4.91 (q, J = 6.5 Hz, 1H), 4.05 (hept, J = 5.9 Hz, 1H), 1.62 (d, J = 6.5 Hz, 3H). **^{13}C NMR** (151 MHz, $CDCl_3$) δ 139.2, 137.7, 135.5, 130.6, 128.5, 121.7, 123.1 – 119.8 (m), 118.1, 80.8, 73.3 (dt, $J_{C=F}$ = 64.4, 32.1 Hz), 23.1. **^{19}F NMR** (376 MHz, $CDCl_3$) δ -73.06 (q, J = 9.3 Hz), -73.65 (q, J = 9.5 Hz).

HRMS (ESI+), *m/z*: calculated for $C_{14}H_{13}F_6N_2O$ $[M + H]^+$: 339.0932, found: 339.0934.

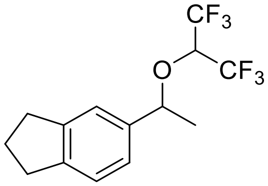

5-(1-((1,1,1,3,3,3-Hexafluoropropan-2-yl)oxy)ethyl)-2,3-dihydro-1*H*-indene (81d)

Prepared according to the general procedure B for the Etherification. Following workup, the product was purified by column chromatography (hexane:EtOAc, 20:1) to give the title compound as a colorless oil (isolated yield: 66%).

^{1}H NMR (500 MHz, $CDCl_3$) δ 7.27 – 7.22 (m, 2H), 7.13 (dd, J = 7.7, 1.7 Hz, 1H), 4.85 (q, J = 6.5 Hz, 1H), 4.02 (hept, J = 6.0 Hz, 1H), 2.95 (td, J = 7.4, 3.3 Hz, 4H), 2.13 (p, J = 7.4 Hz, 2H), 1.60 (d, J = 6.5 Hz, 3H). **^{13}C NMR** (126 MHz, $CDCl_3$) δ 145.3, 145.0, 137.2, 125.3, 124.6, 123.3, 123.6 – 119.7 (m), 81.3, 72.5 (p, $J_{C=F}$ = 32.1 Hz), 32.7, 32.6, 25.4, 23.3. **^{19}F NMR** (282 MHz, $CDCl_3$) δ -72.76 (p, $J_{C=F}$ = 8.9 Hz), -73.66 (p, $J_{C=F}$ = 9.0 Hz).

HRMS (ESI+), *m/z*: calculated for $C_{14}H_{15}F_6O$ $[M + H]^+$: 313.1027, found: 313.1016.

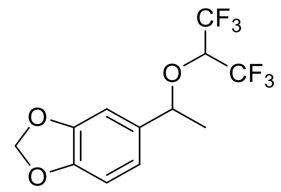

5-(1-((1,1,1,3,3,3-Hexafluoropropan-2-yl)oxy)ethyl)benzo[*d*][1,3]dioxole (82d)

Prepared according to the general procedure B for the Etherification. Following workup, the product was purified by column chromatography (hexane:EtOAc, 20:1) to give the title compound as a brown oil (isolated yield: 90%).

^{1}H NMR (500 MHz, $CDCl_3$) δ 6.88 (d, J = 1.6 Hz, 1H), 6.83 – 6.78 (m, 2H), 6.02 – 6.00 (m, 2H), 4.78 (q, J = 6.4 Hz, 1H), 4.02 (hept, J = 6.0 Hz, 1H), 1.57 (d, J = 6.4 Hz, 3H). **^{13}C NMR** (126 MHz, $CDCl_3$) δ 149.7 (d, $J_{C=F}$ = 9.1 Hz), 134.6, 124.9 – 121.3 (m), 122.8, 109.5, 108.5, 102.8, 82.5, 73.8 (p, $J_{C=F}$ = 32.3 Hz), 24.5. **^{19}F NMR** (282 MHz, $CDCl_3$) δ -72.88 (p, J = 8.8 Hz), -73.71 (p, J = 8.9 Hz).

HRMS (ESI+), *m/z*: calculated for $C_{12}H_{11}F_6O_3$ [M + H]$^+$: 317.0612, found: 317.0635.

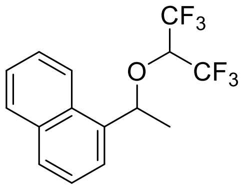

1-(1-((1,1,1,3,3,3-Hexafluoropropan-2-yl)oxy)ethyl)naphthalene (83d)

Prepared according to the general procedure B for the Etherification. Following workup, the product was purified by column chromatography (hexane:EtOAc, 20:1) to give the title compound as a yellow oil (isolated yield: 63%).

^{1}H NMR (500 MHz, $CDCl_3$) δ 8.15 – 8.12 (m, 1H), 7.96 – 7.93 (m, 1H), 7.90 (dt, J = 8.2, 1.1 Hz, 1H), 7.67 (dd, J = 7.2, 1.2 Hz, 1H), 7.61 – 7.53 (m, 3H), 5.67 (q, J = 6.5 Hz, 1H), 4.13 (hept, J = 5.9 Hz, 1H), 1.80 (d, J = 6.5 Hz, 3H). **^{13}C NMR** (126 MHz, $CDCl_3$) δ 137.0, 135.4, 132.0, 130.7, 130.6, 127.9, 127.3, 126.9, 126.5, 124.8 – 121.4 (m), 124.3, 80.3, 75.1 (dt, $J_{C=F}$ = 63.9, 32.1 Hz), 24.5. **^{19}F NMR** (282 MHz, $CDCl_3$) δ -72.76 (p, J = 8.9 Hz), -73.16 – -73.35 (m).

HRMS (ESI+), *m/z*: calculated for $C_{15}H_{13}F_6O$ [M + H]$^+$: 323.0871, found: 323.0876.

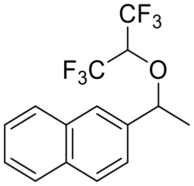

2-(1-((1,1,1,3,3,3-Hexafluoropropan-2-yl)oxy)ethyl)naphthalene (84d)

Prepared according to the general procedure B for the Etherification. Following workup, the product was purified by column chromatography (hexane:EtOAc, 20:1) to give the title compound as a white solid (isolated yield: 55%).

^{1}H NMR (500 MHz, $CDCl_3$) δ 7.94 (d, J = 8.5 Hz, 1H), 7.90 (dt, J = 6.3, 2.8 Hz, 2H), 7.79 (d, J = 1.8 Hz, 1H), 7.59 – 7.50 (m, 3H), 5.04 (t, J = 6.4 Hz, 1H), 4.07 (hept, J = 5.9 Hz, 1H), 1.71 (d, J = 6.5 Hz, 3H). **^{13}C NMR** (151 MHz, $CDCl_3$) δ 136.8, 133.6, 133.0, 129.0, 128.0, 127.8, 127.0, 126.7, 126.6, 124.0, 123.4 – 119.8 (m), 81.6, 72.8 (p, $J_{C\text{=}F}$ = 32.1 Hz), 23.1. **^{19}F NMR** (376 MHz, $CDCl_3$) δ -72.81 (q, J = 9.3 Hz), -73.61 (q, J = 9.4 Hz).

HRMS (ESI+), *m/z*: calculated for $C_{15}H_{13}F_6O$ $[M + H]^+$: 323.0871, found: 323.0877.

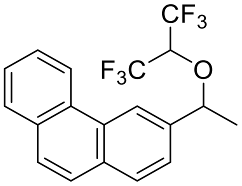

3-(1-((1,1,1,3,3,3-Hexafluoropropan-2-yl)oxy)ethyl)phenanthrene (85d)

Prepared according to the general procedure B for the Etherification. Following workup, the product was purified by column chromatography (hexane:EtOAc, 20:1) to give the title compound as a colorless oil (isolated yield: 56%).

^{1}H NMR (500 MHz, $CDCl_3$) δ 8.75 (d, J = 8.4 Hz, 1H), 8.70 – 8.61 (m, 1H), 8.02 – 7.95 (m, 2H), 7.86 – 7.77 (m, 2H), 7.74 (ddd, J = 8.4, 7.0, 1.5 Hz, 1H), 7.70 – 7.60 (m, 2H), 5.17 (q, J = 6.4 Hz, 1H), 4.13 (dt, J = 11.9, 5.9 Hz, 1H), 1.77 (d, J = 6.4 Hz, 3H). **^{13}C NMR** (126 MHz, $CDCl_3$) δ 137.6, 132.4, 132.3, 130.2, 130.1, 129.5, 128.8, 127.7, 127.0, 126.9, 126.5, 125.0, 123.6 – 120.9 (m), 122.6, 121.7, 81.9, 73.4 – 72.4 (m), 23.6. **^{19}F NMR** (376 MHz, $CDCl_3$) δ -72.73 (q, J = 9.5 Hz), -73.53 (q, J = 9.3 Hz).

HRMS (ESI+), *m/z*: calculated for $C_{19}H_{15}F_6O$ $[M + H]^+$: 373.1027, found: 373.1035.

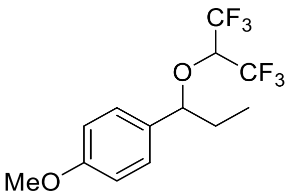

1-(1-((1,1,1,3,3,3-Hexafluoropropan-2-yl)oxy)propyl)-4-methoxybenzene (86d)

Prepared according to the general procedure B for the Etherification. Following workup, the product was purified by column chromatography (hexane:EtOAc, 20:1) to give the title compound as a colorless oil (isolated yield: 75%).

^{1}H NMR (500 MHz, $CDCl_3$) δ 7.26 (d, J = 8.6 Hz, 2H), 6.94 (d, J = 8.7 Hz, 2H), 4.52 (t, J = 6.9 Hz, 1H), 3.99 (hept, J = 6.0 Hz, 1H), 3.85 (s, 3H), 2.04 (dt, J = 13.8, 7.3 Hz, 1H), 1.81 – 1.72 (m, 1H), 0.93 (t, J = 7.4 Hz, 3H). **^{13}C NMR** (126 MHz, $CDCl_3$) δ 160.1, 130.1, 129.2, 127.1 – 119.7 (m), 114.1, 86.5, 72.3 (dt, $J_{C=F}$ = 63.9, 31.8 Hz), 55.2, 30.3, 10.1. **^{19}F NMR** (282 MHz, $CDCl_3$) δ -72.56 (p, $J_{C=F}$ = 8.9 Hz), -73.66 (p, $J_{C=F}$ = 9.1 Hz).

HRMS (ESI+), *m/z*: calculated for $C_{13}H_{15}F_6O_2$ $[M + H]^+$: 317.0976, found: 317.0962.

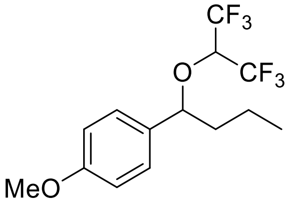

(1-((1,1,1,3,3,3-Hexafluoropropan-2-yl)oxy)dodecyl)benzene (87d)

Prepared according to the general procedure B for the Etherification. Following workup, the product was purified by column chromatography (hexane:EtOAc, 20:1) to give the title compound as a colorless oil (isolated yield: 60%).

^{1}H NMR (500 MHz, $CDCl_3$) δ 7.25 (d, J = 8.6 Hz, 2H), 6.93 (d, J = 8.6 Hz, 2H), 4.61 (t, J = 6.9 Hz, 1H), 3.97 (p, J = 6.0 Hz, 1H), 3.85 (s, 3H), 2.01 (dddd, J = 13.3, 9.9, 7.6, 5.4 Hz, 1H), 1.69 (ddt, J = 13.7, 10.0, 5.9 Hz, 1H), 1.45 (dddd, J = 17.5, 12.9, 8.8, 6.5 Hz, 1H), 1.27 (dddd, J = 13.3, 10.0, 7.5, 5.8 Hz, 1H), 0.93 (t, J = 7.4 Hz, 3H). **^{13}C NMR** (126 MHz, $CDCl_3$) δ 160.1, 130.3, 129.2, 114.1, 84.8, 72.9 – 71.4 (m), 55.3, 39.4, 18.9, 13.7. **^{19}F NMR** (376 MHz, $CDCl_3$) δ -72.51 (q, J = 9.5 Hz), -73.63 (q, J = 9.4 Hz).

HRMS (ESI+), *m/z*: calculated for $C_{14}H_{16}F_6O_2Na$ $[M + Na]^+$: 353.0952, found: 353.0943.

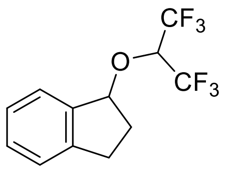

1-((1,1,1,3,3,3-Hexafluoropropan-2-yl)oxy)-2,3-dihydro-1*H*-indene (88d)

Prepared according to the general procedure B for the Etherification. Following workup, the product was purified by column chromatography (hexane:EtOAc, 20:1) to give the title compound as a yellow oil (isolated yield: 80%).

^{1}H NMR (500 MHz, $CDCl_3$) δ 7.45 (d, *J* = 7.4 Hz, 1H), 7.38 – 7.32 (m, 2H), 7.31 – 7.27 (m, 1H), 5.30 (dd, *J* = 6.3, 3.6 Hz, 1H), 4.32 (hept, *J* = 6.0 Hz, 1H), 3.22 – 3.12 (m, 1H), 2.88 (ddd, *J* = 16.0, 8.2, 4.6 Hz, 1H), 2.42 (ddt, *J* = 12.8, 8.2, 6.3 Hz, 1H), 2.28 (dddd, *J* = 13.7, 8.1, 4.6, 3.5 Hz, 1H). **^{13}C NMR** (126 MHz, $CDCl_3$) δ 144.5, 140.1, 129.5, 126.7, 125.4, 125.2, 123.0 – 119.9 (m), 87.9, 74.6 (p, $J_{C=F}$ = 32.4 Hz), 32.7, 29.9. **^{19}F NMR** (376 MHz, $CDCl_3$) δ -73.49 – -73.62 (m), -73.93 – -74.09 (m).

HRMS (ESI+), *m/z*: calculated for $C_{12}H_{11}F_6O$ $[M + H]^+$: 285.0714, found: 285.0726.

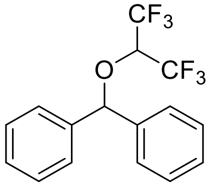

(((1,1,1,3,3,3-Hexafluoropropan-2-yl)oxy)methylene)dibenzene (89d)

Prepared according to the general procedure B for the Etherification. Following workup, the product was purified by column chromatography (hexane:EtOAc, 20:1) to give the title compound as a colorless oil (isolated yield: 78%).

^{1}H NMR (500 MHz, $CDCl_3$) δ 7.44 – 7.35 (m, 10H), 5.88 (s, 1H), 4.22 (hept, *J* = 5.9 Hz, 1H). **^{13}C NMR** (126 MHz, $CDCl_3$) δ 140.3, 130.1, 130.1, 129.1, 123.1 (dd, J = 284.7, 3.0 Hz), 87.0, 73.9 (dt, $J_{C=F}$ = 64.5, 32.3 Hz). **^{19}F NMR** (282 MHz, $CDCl_3$) δ -72.75 (d, $J_{C=F}$ = 5.9 Hz).

HRMS (ESI+), *m/z*: calculated for $C_{16}H_{13}F_6O$ $[M + H]^+$: 335.0871, found: 335.0868.

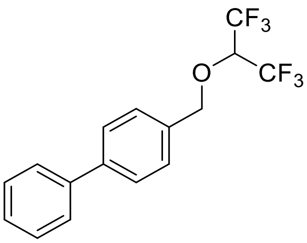

4-(((1,1,1,3,3,3-Hexafluoropropan-2-yl)oxy)methyl)-1,1'-biphenyl (90d)

Prepared according to the general procedure B for the Etherification. Following workup, the product was purified by column chromatography (hexane:EtOAc, 20:1) to give the title compound as a colorless oil (isolated yield: 55%).

^{1}H NMR (500 MHz, $CDCl_3$) δ 7.68 – 7.62 (m, 4H), 7.52 – 7.46 (m, 4H), 7.44 – 7.38 (m, 1H), 4.95 (s, 2H), 4.20 (dq, *J* = 11.7, 5.9 Hz, 1H). **^{13}C NMR** (126 MHz, $CDCl_3$) δ 142.0, 140.5, 133.6, 129.2, 128.9, 127.7, 127.5, 127.2, 125.2 – 119.8 (m), 75.8, 74.4 (p, $J_{C=F}$ = 32.5 Hz). **^{19}F NMR** (376 MHz, $CDCl_3$) δ -73.46.

HRMS (ESI+), *m/z*: calculated for $C_{16}H_{13}F_6O$ $[M + H]^+$: 335.0871, found: 335.0869.

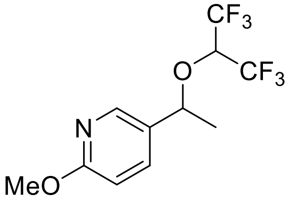

5-(1-((1,1,1,3,3,3-Hexafluoropropan-2-yl)oxy)ethyl)-2-methoxypyridine (91d)

Prepared according to the general procedure B for the Etherification. Following workup, the product was purified by column chromatography (hexane:EtOAc, 20:1) to give the title compound as a colorless oil (isolated yield: 60%).

^{1}H NMR (500 MHz, $CDCl_3$) δ 8.08 (d, *J* = 2.4 Hz, 1H), 7.64 (dd, *J* = 8.6, 2.5 Hz, 1H), 6.82 (d, *J* = 8.6 Hz, 1H), 4.81 (q, *J* = 6.5 Hz, 1H), 4.01 – 3.97 (m, 1H), 3.96 (s, 3H), 1.59 (d, *J* = 6.5 Hz, 3H). **^{13}C NMR** (126 MHz, $CDCl_3$) δ 164.8, 146.0, 137.2, 127.7, 121.5 (dd, $J_{C=F}$ = 282.9, 120.9 Hz), 111.8, 78.8, 72.8 (p, $J_{C=F}$ = 32.3 Hz), 53.6, 22.7. **^{19}F NMR** (282 MHz, $CDCl_3$) δ -73.18 (p, *J* = 8.6 Hz), -73.86 (p, *J* = 8.8 Hz).

HRMS (ESI+), *m/z*: calculated for $C_{11}H_{11}F_6NO_2Na$ $[M + Na]^+$: 326.0592, found: 326.0603.

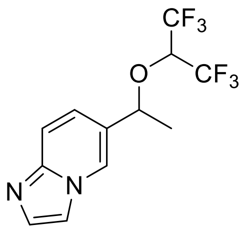

6-(1-((1,1,1,3,3,3-Hexafluoropropan-2-yl)oxy)ethyl)imidazo[1,2-a]pyridine (92d)

Prepared according to the general procedure B for the Etherification. Following workup, the product was purified by column chromatography (hexane:EtOAc, 10:1) to give the title compound as a colorless oil (isolated yield: 65%).

^{1}H NMR (500 MHz, $CDCl_3$) δ 8.16 – 8.12 (m, 1H), 7.69 – 7.66 (m, 2H), 7.62 (t, *J* = 1.0 Hz, 1H), 7.20 (dd, *J* = 9.3, 1.7 Hz, 1H), 4.85 (q, *J* = 6.4 Hz, 1H), 4.09 (hept, *J* = 5.9 Hz, 1H), 1.65 (d, *J* = 6.5 Hz, 3H). **^{13}C NMR** (151 MHz, $CDCl_3$) δ 145.1, 134.3, 124.7, 124.2, 122.9, 122.8 – 119.0 (m), 118.6, 112.9, 79.0, 73.7 – 72.4 (m), 22.5. **^{19}F NMR** (376 MHz, $CDCl_3$) δ -73.05 (q, *J* = 9.2 Hz), -73.63 (q, *J* = 9.2 Hz).

HRMS (ESI+), *m/z*: calculated for $C_{12}H_{11}F_6N_2O$ $[M + H]^+$: 313.0776, found: 313.0769.

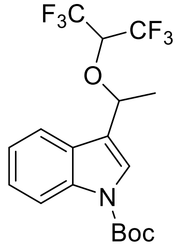

***Tert*-butyl 3-(1-((1,1,1,3,3,3-hexafluoropropan-2-yl)oxy)ethyl)-1*H*-indole-1-carboxylate (93d)**

Prepared according to the general procedure B for the Etherification. Following workup, the product was purified by column chromatography (hexane:EtOAc, 10:1) to give the title compound as a colorless oil (isolated yield: 75%).

^{1}H NMR (500 MHz, $CDCl_3$) δ 8.17 (d, *J* = 7.5 Hz, 1H), 7.70 (d, *J* = 7.9 Hz, 1H), 7.63 (s, 1H), 7.37 (ddd, *J* = 8.4, 7.1, 1.3 Hz, 1H), 7.28 (ddd, *J* = 8.3, 7.3, 1.1 Hz, 1H), 5.13 (q, *J* = 6.5 Hz, 1H), 4.18 (hept, *J* = 6.0 Hz, 1H), 1.76 (d, *J* = 6.6 Hz, 3H), 1.70 (s, 9H). **^{13}C NMR** (151 MHz, $CDCl_3$) δ 149.5, 136.1, 127.9, 125.0 (d, $J_{C=F}$ = 4.9 Hz), 122.8, 123.3 – 120.9 (m), 120.3, 118.9, 115.5, 84.3, 74.9, 72.7 (dt, $J_{C=F}$ = 64.3, 32.2 Hz), 28.2, 21.5. **^{19}F NMR** (376 MHz, $CDCl_3$) δ -72.83 (q, *J* = 9.5 Hz), -73.45 (q, *J* = 9.6 Hz).

HRMS (ESI+), *m/z*: calculated for $C_{18}H_{20}F_6NO_3$ $[M + H]^+$: 412.1347, found: 412.1346.

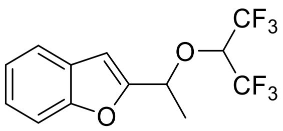

2-(1-((1,1,1,3,3,3-Hexafluoropropan-2-yl)oxy)ethyl)benzofuran (94d)

Prepared according to the general procedure B for the Etherification. Following workup, the product was purified by column chromatography (hexane:EtOAc, 20:1) to give the title compound as a yellow solid (isolated yield: 87%).

^{1}H NMR (500 MHz, $CDCl_3$) δ 7.61 – 7.58 (m, 1H), 7.50 (dq, *J* = 8.3, 0.9 Hz, 1H), 7.34 (ddd, *J* = 8.4, 7.3, 1.4 Hz, 1H), 7.28 – 7.24 (m, 1H), 6.78 (d, *J* = 0.9 Hz, 1H), 4.99 (q, *J* = 6.6 Hz, 1H), 4.28 (hept, *J* = 5.9 Hz, 1H), 1.74 (d, *J* = 6.6 Hz, 3H). **^{13}C NMR** (126 MHz, $CDCl_3$) δ 155.2, 154.3, 127.4, 125.2, 123.2, 121.5, 111.5, 106.1, 74.5, 73.4 (dd, $J_{C=F}$ = 65.2, 32.3 Hz), 19.3. **^{19}F NMR** (376 MHz, $CDCl_3$) δ -73.44 (q, *J* = 9.2 Hz), -74.11 (q, *J* = 9.2 Hz).

HRMS (ESI+), *m/z*: calculated for $C_{13}H_{11}F_6O_2$ $[M + H]^+$: 313.0663, found: 313.0652.

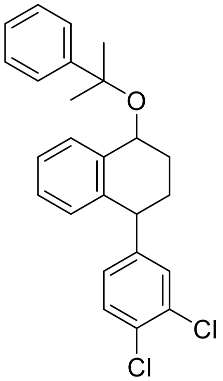

1-(3,4-Dichlorophenyl)-4-((2-phenylpropan-2-yl)oxy)-1,2,3,4-tetrahydronaphthalene (95d)

Prepared according to the general procedure A for the Etherification. Following workup, the product was purified by column chromatography (hexane:EtOAc, 20:1) to give the title compound as a colorless oil (isolated yield: 53%, d.r. = 1:1, We just isolated this product of a single configuration).

^{1}H NMR (500 MHz, $CDCl_3$) δ 7.59 – 7.54 (m, 2H), 7.37 – 7.33 (m, 3H), 7.30 – 7.27 (m, 2H), 7.20 – 7.13 (m, 2H), 7.10 (td, *J* = 7.4, 1.8 Hz, 1H), 7.00 – 6.98 (m, 1H), 6.78 (d, *J* = 7.7 Hz, 1H), 4.59 – 4.53 (m, 1H), 3.93 (t, *J* = 7.3 Hz, 1H), 2.07 (ddd, *J* = 11.0, 6.8, 2.7 Hz, 1H), 1.97 (dtd, *J* = 13.6, 6.9, 3.3 Hz, 1H), 1.77 (tdd, *J* = 9.4, 4.7, 2.4 Hz, 1H), 1.72 (d, *J* = 2.7 Hz, 6H), 1.63 – 1.53 (m, 1H). **^{13}C NMR** (126 MHz, $CDCl_3$) δ 147.7, 146.7, 139.4, 138.6, 132.2, 130.8, 130.2, 130.0, 129.7, 129.0, 128.4, 128.0, 127.4, 127.2, 126.4, 126.3, 68.9, 44.5, 29.5, 29.0, 28.6, 28.4.

HRMS (ESI+), *m/z*: calculated for $C_{25}H_{25}Cl_2O$ $[M + H]^+$: 411.1282, found: 411.1288.

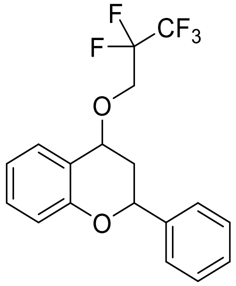

4-(2,2,3,3,3-Pentafluoropropoxy)-2-phenylchromane (96d)

Prepared according to the general procedure B for the Etherification. Following workup, the product was purified by column chromatography (hexane:EtOAc, 20:1) to give the title compound as a yellow oil (isolated yield: 42%, d.r. = 1.2:1).

^{1}H NMR (500 MHz, $CDCl_3$) δ 7.51 – 7.48 (m, 2.5H), 7.45 (td, *J* = 7.8, 7.4, 1.7 Hz, 2H), 7.42 – 7.37 (m, 1H), 7.34 (ddd, *J* = 8.6, 7.2, 1.7 Hz, 0.5H), 7.30 – 7.24 (m, 1H), 7.05 – 6.99 (m, 1.5H), 6.94 (dd, *J* = 8.2, 1.2 Hz, 0.5H), 5.33 (dd, *J* = 12.3, 2.1 Hz, 0.5H), 5.20 – 5.16 (m, 0.5H), 5.12 (dd, *J* = 10.7, 6.2 Hz, 0.5H), 4.61 (t, *J* = 2.8 Hz, 0.5H), 4.15 – 3.98 (m, 2H), 2.54 (ddd, *J* = 12.9, 6.2, 1.9 Hz, 0.5H), 2.39 (dt, *J* = 14.4, 2.4 Hz, 0.5H), 2.24 (ddd, *J* = 13.0, 12.0, 10.7 Hz, 0.5H), 2.14 (ddd, *J* = 14.4, 12.3, 3.1 Hz, 0.5H). **^{13}C NMR** (126 MHz, $CDCl_3$) δ 155.3, 155.0, 140.7, 140.3, 130.8, 130.7, 129.6, 128.8, 128.7, 128.5, 128.2, 127.3, 126.3, 126.1, 121.1, 120.3, 119.1, 117.7, 117.0, 76.7, 74.4, 73.1, 73.0, 64.6 (d, $J_{C\text{-}F}$ = 30.2 Hz), 35.4, 35.3. **^{19}F NMR** (376 MHz, $CDCl_3$) δ -83.25, -83.31, -122.94, -123.42.

HRMS (ESI+), *m/z*: calculated for $C_{18}H_{16}F_5O_2$ $[M + H]^+$: 359.1070, found: 359.1073.

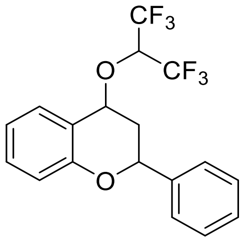

4-((1,1,1,3,3,3-Hexafluoropropan-2-yl)oxy)-2-phenylchromane (97d)

Prepared according to the general procedure B for the Etherification. Following workup, the product was purified by column chromatography (hexane:EtOAc, 20:1) to give the title compound as a white solid (isolated yield: 76%, d.r. = 1.5:1).

^{1}H NMR (500 MHz, $CDCl_3$) δ 7.56 (dt, *J* = 7.9, 1.3 Hz, 0.5H), 7.53 – 7.46 (m, 4H), 7.45 – 7.28 (m, 0.5H), 7.09 – 7.04 (m, 1.5H), 6.97 (dd, *J* = 8.3, 1.2 Hz, 0.5H), 5.34 (dd, *J* = 12.4, 2.0 Hz, 0.6H),

5.25 (dd, J = 10.7, 6.2 Hz, 0.4H), 5.16 (dd, J = 12.2, 1.8 Hz, 0.4H), 4.89 (d, J = 2.7 Hz, 0.6H), 4.48 – 4.34 (m, 1H), 2.57 (ddd, J = 13.1, 6.3, 1.9 Hz, 0.4H), 2.43 (dt, J = 14.8, 2.3 Hz, 0.6H), 2.34 (td, J = 12.6, 10.6 Hz, 0.4H), 2.17 (ddd, J = 15.0, 12.4, 2.9 Hz, 0.6H). **^{13}C NMR** (126 MHz, $CDCl_3$) δ 155.5, 154.8, 140.4, 139.9, 131.3, 131.1, 130.1, 128.9, 128.8, 128.6, 128.3, 127.4, 126.2, 126.2, 121.2, 121.0, 120.8, 118.1, 117.8, 117.0, 77.4, 76.5, 75.2, 74.9 – 74.0 (m), 73.6 – 73.0 (m), 72.8, 35.7, 35.2. **^{19}F NMR** (282 MHz, $CDCl_3$) δ -73.18 (p, J = 8.7 Hz), -73.48 (q, J = 7.8 Hz), -73.62 (q, J = 8.3 Hz).

HRMS (ESI+), *m/z*: calculated for $C_{18}H_{15}F_6O_2$ $[M + H]^+$: 377.0976, found: 377.0971.

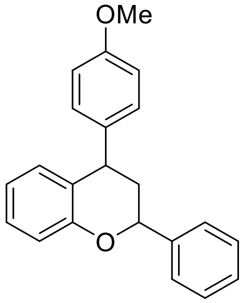

4-(4-Methoxyphenyl)-2-phenylchromane (97e)

Following workup, the product was purified by column chromatography (hexane:EtOAc, 20:1) to give the title compound as a colorless oil (isolated yield: 60%, d.r. = 0.7:1).

^{1}H NMR (500 MHz, $CDCl_3$) δ 7.54 – 7.50 (m, 1H), 7.45 – 7.41 (m, 1H), 7.39 – 7.36 (m, 2.5H), 7.35 – 7.30 (m, 0.6H), 7.24 (ddd, J = 8.5, 7.0, 1.8 Hz, 0.6H), 7.20 – 7.15 (m, 1.3H), 7.11 – 7.06 (m, 1.6H), 7.06 – 7.01 (m, 1H), 7.00 – 6.97 (m, 0.4H), 6.93 (dd, J = 7.3, 1.3 Hz, 0.5H), 6.91 – 6.87 (m, 2H), 6.84 – 6.82 (m, 0.7H), 5.25 (dd, J = 11.4, 2.0 Hz, 0.4H), 5.08 (dd, J = 10.5, 2.4 Hz, 0.6H), 4.35 (dd, J = 12.2, 5.8 Hz, 0.4H), 4.22 (dd, J = 5.6, 3.4 Hz, 0.6H), 3.83 (d, J = 0.9 Hz, 3H), 2.50 – 2.39 (m, 1H), 2.33 – 2.22 (m, 1H). **^{13}C NMR** (126 MHz, $CDCl_3$) δ 158.4, 158.2, 155.5, 155.4, 141.5, 141.3, 138.3, 136.5, 130.8, 129.8, 129.6, 129.5, 128.6, 128.5, 128.1, 127.8, 127.7, 126.1, 126.1, 123.5, 120.6, 120.5, 117.1, 117.0, 114.1, 113.8, 78.2, 73.3, 55.30,42.7, 40.7, 39.4, 38.5.

HRMS (ESI+), *m/z*: calculated for $C_{22}H_{21}O_2$ $[M + H]^+$: 317.1542, found: 317.1546.

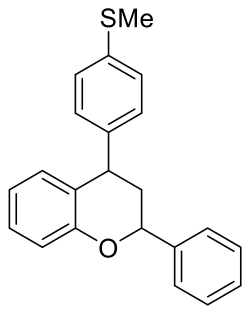

4-(4-(Methylthio)phenyl)-2-phenylchromane (97f)

Following workup, the product was purified by column chromatography (hexane:EtOAc, 20:1) to give the title compound as a white solid (isolated yield: 47%, d.r. = 1.4:1).

^{1}H NMR (500 MHz, $CDCl_3$) δ 7.54 – 7.49 (m, 1H), 7.45 – 7.40 (m, 1.5H), 7.40 – 7.35 (m, 2.5H), 7.27 – 7.23 (m, 2.5H), 7.18 (dd, *J* = 8.5, 2.2 Hz, 1.5H), 7.12 – 7.08 (m, 1H), 7.05 (dd, *J* = 8.3, 1.2 Hz, 0.5H), 7.00 (ddd, *J* = 12.5, 7.9, 1.4 Hz, 1H), 6.92 (td, *J* = 7.4, 1.2 Hz, 0.5H), 6.86 – 6.79 (m, 1H), 5.24 (dd, *J* = 11.5, 1.9 Hz, 0.6H), 5.06 (dd, *J* = 10.6, 2.5 Hz, 0.4H), 4.36 (dd, *J* = 12.2, 5.9 Hz, 0.6H), 4.23 (dd, *J* = 5.7, 3.4 Hz, 0.4H), 2.51 (s, 3H), 2.41 (s, 1H), 2.32 – 2.22 (m, 1H). **^{13}C NMR** (126 MHz, $CDCl_3$) δ 155.5, 155.4, 143.1, 141.5, 141.3, 141.1, 136.6, 136.3, 130.7, 129.7, 129.2, 129.1, 128.6, 128.5, 128.2, 128.1, 127.9, 127.8, 127.1, 126.8, 126.1, 126.0, 125.5, 122.9, 120.6, 120.6, 117.1, 117.1, 78.1, 73.2, 43.0, 40.6, 39.7, 38.3, 16.0, 16.0.

HRMS (ESI+), *m/z*: calculated for $C_{22}H_{21}OS$ $[M + H]^+$: 333.1313, found: 333.1321.

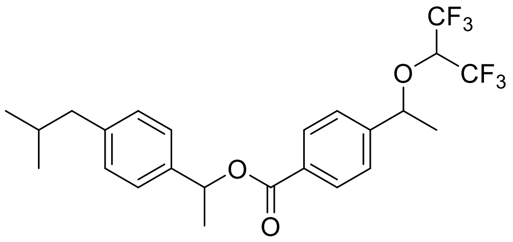

1-(4-Isobutylphenyl)ethyl 4-(1-((1,1,1,3,3,3-hexafluoropropan-2-yl)oxy)ethyl)benzoate (98d)

Prepared according to the general procedure B for the Etherification. Following workup, the product was purified by column chromatography (hexane:EtOAc, 20:1) to give the title compound as a colorless oil (isolated yield: 36%, d.r. = 3:1, We just isolated this product of a single configuration).

^{1}H NMR (500 MHz, $CDCl_3$) δ 7.36 – 7.30 (m, 4H), 7.18 (d, *J* = 8.1 Hz, 2H), 7.06 (d, *J* = 8.5 Hz, 2H), 4.85 (q, *J* = 6.4 Hz, 1H), 4.04 – 3.93 (m, 2H), 2.51 (d, *J* = 7.2 Hz, 2H), 1.90 (dt, *J* = 13.6, 6.8

Hz, 1H), 1.64 (dd, J = 7.1, 1.0 Hz, 3H), 1.57 (d, J = 6.4 Hz, 3H), 0.94 (d, J = 6.6 Hz, 6H). **^{13}C NMR** (126 MHz, $CDCl_3$) δ 177.8, 155.9, 145.7, 141.8, 141.7, 134.3, 132.9, 131.9, 126.6, 127.9 – 124.7 (m), 85.5, 78.5 – 76.6 (m), 50.0, 49.8, 34.9, 27.9, 27.1, 23.2. **^{19}F NMR** (376 MHz, $CDCl_3$) δ -72.92 (qd, J = 9.1, 1.5 Hz), -73.70 (q, J = 9.5 Hz).

HRMS (ESI+), *m/z*: calculated for $C_{24}H_{27}F_6O_3$ $[M + H]^+$: 477.1864, found: 477.1856.

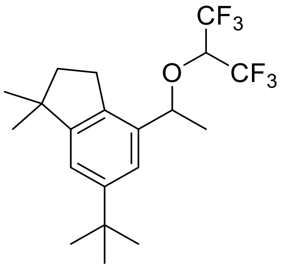

6-(*Tert*-butyl)-4-(1-((1,1,1,3,3,3-hexafluoropropan-2-yl)oxy)ethyl)-1,1-dimethyl-2,3-dihydro-1*H*-indene (99d)

Prepared according to the general procedure B for the Etherification. Following workup, the product was purified by column chromatography (hexane:EtOAc, 20:1) to give the title compound as a colorless oil (isolated yield: 59%).

^{1}H NMR (500 MHz, $CDCl_3$) δ 7.26 (d, J = 1.8 Hz, 1H), 7.17 (d, J = 1.8 Hz, 1H), 5.04 (q, J = 6.5 Hz, 1H), 4.00 (hept, J = 6.0 Hz, 1H), 2.86 – 2.77 (m, 2H), 1.98 – 1.93 (m, 2H), 1.59 (d, J = 6.5 Hz, 4H), 1.34 (s, 9H), 1.31 (s, 3H), 1.27 (s, 3H). **^{13}C NMR** (151 MHz, $CDCl_3$) δ 153.0, 150.6, 138.0, 134.3, 121.3, 119.2, 78.9, 73.0 – 72.4 (m), 43.9, 41.4, 34.8, 31.5, 28.8, 28.6, 27.6, 22.5. **^{19}F NMR** (376 MHz, $CDCl_3$) δ -72.57 (q, J = 9.5 Hz), -73.37 (q, J = 9.5 Hz).

HRMS (ESI+), *m/z*: calculated for $C_{20}H_{27}F_6O$ $[M + H]^+$: 397.1966, found: 397.1978.

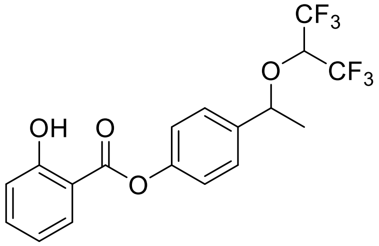

4-(1-((1,1,1,3,3,3-Hexafluoropropan-2-yl)oxy)ethyl)phenyl 2-hydroxybenzoate (100d)

Prepared according to the general procedure B for the Etherification. Following workup, the product was purified by column chromatography (hexane:EtOAc, 20:1) to give the title compound as a colorless oil (isolated yield: 53%).

^{1}H NMR (500 MHz, $CDCl_3$) δ 10.46 (s, 1H), 8.10 (dd, *J* = 8.0, 1.7 Hz, 1H), 7.58 (ddd, *J* = 8.7, 7.2, 1.7 Hz, 1H), 7.47 (d, *J* = 8.6 Hz, 2H), 7.29 (d, *J* = 1.9 Hz, 2H), 7.08 (dd, *J* = 8.4, 1.1 Hz, 1H), 7.01 (ddd, *J* = 8.1, 7.2, 1.1 Hz, 1H), 4.91 (q, *J* = 6.5 Hz, 1H), 4.05 (hept, *J* = 5.9 Hz, 1H), 1.63 (d, *J* = 6.4 Hz, 3H). **^{13}C NMR** (126 MHz, $CDCl_3$) δ 173.5, 167.0, 155.1, 142.6, 141.4, 135.0, 133.1, 126.8, 124.3, 122.6, 116.3, 85.5, 78.2 – 77.4 (m), 28.0. **^{19}F NMR** (376 MHz, $CDCl_3$) δ -72.95 (q, *J* = 9.1 Hz), -73.64 (q, *J* = 9.6 Hz).
HRMS (ESI+), *m/z*: calculated for $C_{18}H_{15}F_6O_4$ $[M + H]^+$: 409.0875, found: 409.0879.

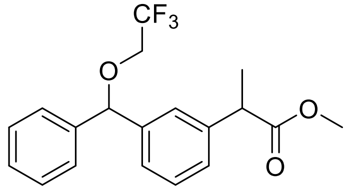

Methyl 2-(3-(phenyl(2,2,2-trifluoroethoxy)methyl)phenyl)propanoate (101d)

Prepared according to the general procedure B for the Etherification. Following workup, the product was purified by column chromatography (hexane:EtOAc, 20:1) to give the title compound as a colorless oil (isolated yield: 74%, d.r. = 12:1, We just isolated this product of a single configuration).

^{1}H NMR (500 MHz, $CDCl_3$) δ 7.38 (d, *J* = 4.5 Hz, 4H), 7.36 – 7.31 (m, 3H), 7.29 – 7.25 (m, 2H), 5.56 (s, 1H), 3.85 (q, *J* = 8.7 Hz, 2H), 3.76 (qd, *J* = 7.2, 3.0 Hz, 1H), 3.68 (d, *J* = 1.8 Hz, 3H), 1.52 (dd, *J* = 7.2, 2.3 Hz, 3H). **^{13}C NMR** (126 MHz, $CDCl_3$) δ 174.8 (d, $J_{C=F}$ = 3.2 Hz), 140.9, 140.3, 128.9, 128.7, 128.1, 127.1 (d, $J_{C=F}$ = 2.7 Hz), 126.3 (d, $J_{C=F}$ = 5.1 Hz), 125.8 (d, $J_{C=F}$ = 8.5 Hz), 125.2, 123.0, 84.5, 66.0 (q, $J_{C=F}$ = 34.2 Hz), 52.0, 45.4 (d, $J_{C=F}$ = 5.2 Hz), 18.6 (d, $J_{C=F}$ = 10.1 Hz). **^{19}F NMR** (282 MHz, $CDCl_3$) δ -73.73 (t, *J* = 8.7 Hz).
HRMS (ESI+), *m/z*: calculated for $C_{19}H_{20}F_3O_3$ $[M + H]^+$: 375.1184, found: 375.1192.

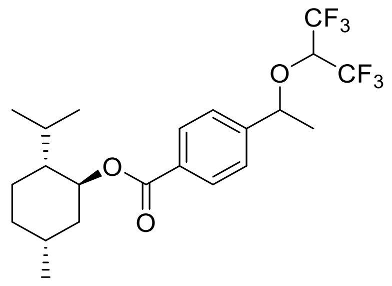

(1*S*,2*R*,5*R*)-2-Isopropyl-5-methylcyclohexyl 4-(1-((1,1,1,3,3,3-hexafluoropropan-2-yl)oxy)ethyl)benzoate (102d)

Prepared according to the general procedure B for the Etherification. Following workup, the product was purified by column chromatography (hexane:EtOAc, 20:1) to give the title compound as a yellow oil (isolated yield: 35%, d.r. = 8:1, We just isolated this product of a single configuration).

^{1}H NMR (500 MHz, $CDCl_3$) δ 8.09 (dd, *J* = 8.3, 1.9 Hz, 2H), 7.46 – 7.42 (m, 2H), 4.97 (tdd, *J* = 10.8, 4.4, 2.1 Hz, 1H), 4.91 (q, *J* = 6.5 Hz, 1H), 4.06 – 3.96 (m, 1H), 2.14 (dqt, *J* = 9.7, 3.7, 2.7, 1.6 Hz, 1H), 1.98 (td, *J* = 7.0, 2.8 Hz, 1H), 1.79 – 1.71 (m, 2H), 1.60 (d, *J* = 6.4 Hz, 5H), 1.22 – 1.07 (m, 2H), 0.95 (dd, *J* = 6.8, 3.9 Hz, 7H), 0.82 (d, *J* = 6.9 Hz, 3H). **^{13}C NMR** (126 MHz, $CDCl_3$) δ 165.6, 144.5, 131.4, 130.1, 126.8 (d, $J_{C\text{–}F}$ = 5.8 Hz), 124.9 – 118.1 (m), 81.1, 75.1, 73.5 (ddd, $J_{C\text{–}F}$ = 65.0, 32.6, 5.6 Hz), 47.3, 41.0, 34.3, 31.5, 26.5 (d, $J_{C\text{–}F}$ = 5.1 Hz), 23.6 (d, $J_{C\text{–}F}$ = 3.8 Hz), 23.2, 22.0, 20.8, 16.5 (d, $J_{C\text{–}F}$ = 4.7 Hz). **^{19}F NMR** (376 MHz, $CDCl_3$) δ -73.01 (p, *J* = 9.0 Hz), -73.64 (q, *J* = 9.3 Hz).

HRMS (ESI+), *m/z*: calculated for $C_{22}H_{29}F_6O_3$ $[M + H]^+$: 455.2021, found: 455.2016.

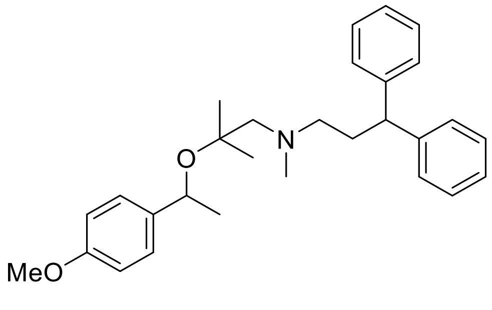

***N*-(3,3-Diphenylpropyl)-2-(1-(4-methoxyphenyl)ethoxy)-*N*,2-dimethylpropan-1-amine (103d)**

Prepared according to the general procedure A for the Etherification. Following workup, the product was purified by column chromatography (hexane:EtOAc, 20:1) to give the title compound as a colorless oil (isolated yield: 43%).

^{1}H NMR (500 MHz, $CDCl_3$) δ 7.34 – 7.23 (m, 10H), 7.19 (d, *J* = 1.4 Hz, 2H), 6.86 (d, *J* = 8.7 Hz, 2H), 4.65 (q, *J* = 6.4 Hz, 1H), 4.00 (t, *J* = 7.7 Hz, 1H), 3.82 (s, 3H), 2.42 (td, *J* = 6.7, 1.9 Hz, 2H), 2.35 (s, 3H), 2.32 (d, *J* = 2.7 Hz, 2H), 2.23 (q, *J* = 7.5 Hz, 2H), 1.32 (d, *J* = 6.4 Hz, 3H), 1.16 (s, 3H), 1.01 (s, 3H). **^{13}C NMR** (126 MHz, $CDCl_3$) δ 158.2, 145.1, 139.9, 128.4, 127.9, 126.7, 126.1, 113.4, 77.6, 69.4, 68.0, 58.4, 55.2, 49.1, 45.0, 33.6, 26.7, 25.3, 24.3.

HRMS (ESI+), *m/z*: calculated for $C_{29}H_{38}NO_2$ $[M + H]^+$: 432.2903, found: 432.2912.

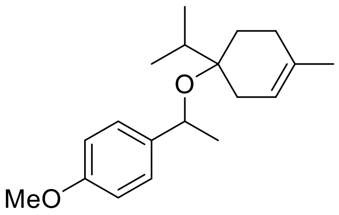

1-(1-((1-Isopropyl-4-methylcyclohex-3-en-1-yl)oxy)ethyl)-4-methoxybenzene (104d)

Prepared according to the general procedure A for the Etherification. Following workup, the product was purified by column chromatography (hexane:EtOAc, 20:1) to give the title compound as a colorless oil (isolated yield: 52%, d.r. = 1.1:1).

^{1}H NMR (500 MHz, $CDCl_3$) δ 7.28 – 7.21 (m, 2H), 6.88 – 6.80 (m, 2H), 5.27 (ddt, *J* = 5.3, 3.7, 1.5 Hz, 0.5H), 4.99 (ddq, *J* = 4.8, 3.3, 1.6 Hz, 0.5H), 4.62 (dq, *J* = 12.7, 6.4 Hz, 1H), 3.82 (d, *J* = 2.4 Hz, 3H), 2.14 – 1.95 (m, 2.5H), 1.92 – 1.74 (m, 3.5H), 1.69 – 1.57 (m, 4H), 1.54 – 1.45 (m, 0.5H), 1.35 (dd, *J* = 16.5, 6.4 Hz, 3H), 0.95 (dd, *J* = 6.8, 3.4 Hz, 3H), 0.87 (d, *J* = 6.8 Hz, 1.5H), 0.81 (d, *J* = 6.8 Hz, 1.5H). **^{13}C NMR** (126 MHz, $CDCl_3$) δ 158.2, 158.1, 140.0, 139.8, 133.7, 133.2, 126.9, 126.7, 119.2, 118.8, 113.3, 113.1, 78.0, 68.8, 68.5, 55.2, 32.7, 32.6, 31.1, 30.7, 28.2, 28.2, 28.1, 26.8, 26.6, 23.2, 23.1, 17.6, 17.5, 17.4, 17.3.

HRMS (ESI+), *m/z*: calculated for $C_{19}H_{29}O_2$ $[M + H]^+$: 289.2168, found: 289.2171.

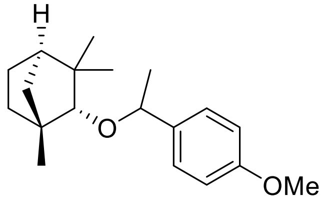

(1*R*,2*R*,4*R*)-2-(1-(4-Methoxyphenyl)ethoxy)-1,3,3-trimethylbicyclo[2.2.1]heptane (105d)

Prepared according to the general procedure A for the Etherification. Following workup, the product was purified by column chromatography (hexane:EtOAc, 20:1) to give the title compound as a colorless oil (isolated yield: 69%, d.r. = 1.2:1).

^{1}H NMR (500 MHz, $CDCl_3$) δ 7.30 – 7.24 (m, 2H), 6.92 – 6.87 (m, 2H), 4.38 (q, *J* = 6.4 Hz, 0.5H), 4.32 (q, *J* = 6.4 Hz, 0.5H), 3.84 (s, 3H), 3.01 (d, *J* = 1.9 Hz, 0.5H), 2.87 (d, *J* = 1.8 Hz, 0.5H), 1.91 – 1.80 (m, 1H), 1.72 (dddt, *J* = 21.4, 12.1, 9.0, 3.0 Hz, 1H), 1.65 – 1.57 (m, 1H), 1.41 (d, *J* = 6.5 Hz, 3H), 1.40 – 1.31 (m, 2H), 1.18 (s, 1.7H), 1.05 (s, 3H), 1.03 – 0.91 (m, 2.3H), 0.87 (s, 1.5H), 0.79 (s, 1.5H), 0.74 (s, 1.5H). **^{13}C NMR** (126 MHz, $CDCl_3$) δ 158.8, 158.7, 137.4, 136.6, 128.0, 127.6, 113.4, 113.4, 89.7, 88.4, 55.2, 49.4, 49.1, 48.8, 48.5, 41.4, 41.4, 39.3, 39.2, 31.8, 30.9, 26.3, 26.2, 26.0, 24.3, 23.5, 21.6, 20.9, 20.4, 19.8.

HRMS (ESI+), *m/z*: calculated for $C_{19}H_{29}O_2$ $[M + H]^+$: 289.2168, found: 289.2163.

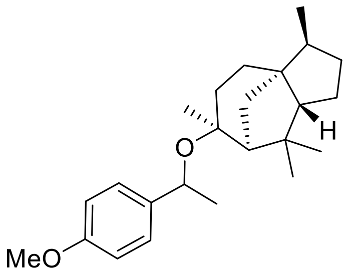

(3*S*,3a*R*,6*S*,7*S*,8a*R*)-6-(1-(4-Methoxyphenyl)ethoxy)-3,6,8,8-tetramethyloctahydro-1*H*-3a,7-methanoazulene (106d)

Prepared according to the general procedure A for the Etherification. Following workup, the product was purified by column chromatography (hexane:EtOAc, 20:1) to give the title compound as a colorless oil (isolated yield: 85%, d.r. = 1.5:1).

^{1}H NMR (500 MHz, $CDCl_3$) δ 7.29 – 7.24 (m, 2H), 6.87 – 6.83 (m, 2H), 4.70 (dq, *J* = 21.1, 6.5 Hz, 1H), 3.82 (s, 3H), 2.02 – 1.84 (m, 2H), 1.83 – 1.76 (m, 1H), 1.75 – 1.63 (m, 3H), 1.59 (dtd, *J* = 12.2, 5.2, 4.7, 2.9 Hz, 1H), 1.55 – 1.40 (m, 2H), 1.38 (d, *J* = 6.5 Hz, 3H), 1.36 – 1.27 (m, 4H), 1.26 (s, 3H), 1.11 (s, 1H), 0.99 (s, 5H), 0.85 (d, *J* = 7.1 Hz, 3H). **^{13}C NMR** (126 MHz, $CDCl_3$) δ 158.3, 158.2, 140.2, 139.6, 127.6, 127.0, 113.4, 113.3, 80.2, 79.6, 69.5, 68.9, 60.2, 58.3, 57.0, 56.8, 55.2, 54.0, 53.8, 43.4, 43.3, 41.5, 41.5, 41.3, 41.1, 37.1, 37.0, 33.3, 31.5, 31.5, 31.3, 29.2, 29.1, 28.3, 28.3, 26.6, 26.4, 25.5, 25.4, 25.4, 25.3, 15.7, 15.6.

HRMS (ESI+), *m/z*: calculated for $C_{24}H_{37}O_2$ $[M + H]^+$: 357.2794, found: 357.2796.

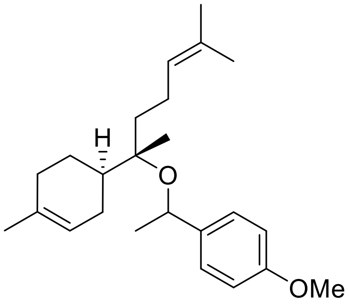

1-Methoxy-4-(1-(((*S*)-6-methyl-2-((*R*)-4-methylcyclohex-3-en-1-yl)hept-5-en-2-yl)oxy)ethyl)benzene (107d)

Prepared according to the general procedure A for the Etherification. Following workup, the product was purified by column chromatography (hexane:EtOAc, 20:1) to give the title compound as a colorless oil (isolated yield: 49%, d.r. = 1:1).

^{1}H NMR (500 MHz, $CDCl_3$) δ 7.28 (ddd, *J* = 8.3, 5.4, 2.1 Hz, 2H), 6.86 (td, *J* = 6.1, 3.0 Hz, 2H), 5.45 – 5.36 (m, 1H), 5.11 (dt, *J* = 13.9, 7.5 Hz, 0.5H), 5.00 – 4.89 (m, 0.5H), 4.62 (dq, *J* = 10.0, 6.5 Hz, 1H), 3.82 (s, 3H), 2.17 – 1.75 (m, 7.3H), 1.74 – 1.61 (m, 9.2H), 1.52 (d, *J* = 12.6 Hz, 2H), 1.46 – 1.16 (m, 5.5H), 1.05 (s, 1.5H), 0.87 (s, 1.5H). **^{13}C NMR** (126 MHz, $CDCl_3$) δ 158.3, 158.2, 140.3, 140.2, 140.1, 139.8, 134.4, 134.3, 134.0, 134.0, 131.2, 131.2, 130.9, 127.0, 126.8, 126.7, 125.1, 125.0, 124.9, 124.8, 121.4, 120.9, 120.7, 113.5, 113.4, 79.8, 79.7, 79.5, 79.5, 68.9, 68.7, 68.7, 68.6, 55.3, 55.2, 41.2, 41.0, 40.9, 40.4, 36.5, 36.3, 36.0, 35.9, 31.3, 31.1, 27.0, 26.9, 26.8, 26.5, 25.8, 25.7, 24.1, 23.9, 23.7, 23.5, 22.3, 22.2, 22.1, 21.9, 20.9, 20.7, 20.5, 17.7, 17.7, 17.6.

HRMS (ESI+), *m/z*: calculated for $C_{24}H_{37}O_2$ $[M + H]^+$: 357.2794, found: 357.2799.

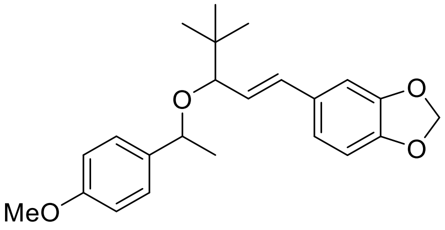

(*E*)-5-(3-(1-(4-Methoxyphenyl)ethoxy)-4,4-dimethylpent-1-en-1-yl)benzo[d][1,3]dioxole (108d)

Prepared according to the general procedure A for the Etherification. Following workup, the product was purified by column chromatography (hexane:EtOAc, 20:1) to give the title compound as a colorless oil (isolated yield: 55%, d.r. = 1:1, We just isolated this product of a single configuration).

^{1}H NMR (500 MHz, $CDCl_3$) δ 7.24 (d, *J* = 8.6 Hz, 2H), 7.01 (d, *J* = 1.6 Hz, 1H), 6.90 (d, *J* = 8.6 Hz, 2H), 6.85 (dd, *J* = 8.0, 1.7 Hz, 1H), 6.81 (d, *J* = 8.0 Hz, 1H), 6.26 (d, *J* = 15.9 Hz, 1H), 6.01 – 5.94 (m, 3H), 4.45 (q, *J* = 6.5 Hz, 1H), 3.85 (s, 3H), 3.22 (d, *J* = 8.5 Hz, 1H), 1.39 (d, *J* = 6.5 Hz, 3H), 0.89 (s, 9H). **^{13}C NMR** (126 MHz, $CDCl_3$) δ 158.8, 148.1, 147.1, 136.2, 133.0, 131.5, 128.1, 126.8, 121.0, 113.5, 108.3, 105.7, 101.1, 84.5, 73.4, 55.2, 34.9, 26.4, 24.6.

HRMS (ESI+), *m/z*: calculated for $C_{23}H_{29}O_4$ $[M + H]^+$: 369.2066, found: 369.2058.

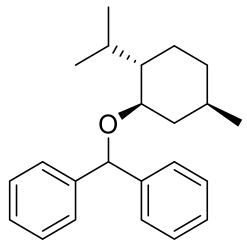

((((1*R*,2*S*,5*R*)-2-Isopropyl-5-methylcyclohexyl)oxy)methylene)dibenzene (109d)

Prepared according to the general procedure A for the Etherification. Following workup, the product was purified by column chromatography (hexane:EtOAc, 20:1) to give the title compound as a white solid (isolated yield: 44%).

^{1}H NMR (500 MHz, $CDCl_3$) δ 7.41 (td, *J* = 7.9, 1.5 Hz, 4H), 7.34 (ddd, *J* = 10.4, 8.4, 6.9 Hz, 4H), 7.31 – 7.22 (m, 2H), 5.58 (s, 1H), 3.19 (td, *J* = 10.5, 4.2 Hz, 1H), 2.43 (pd, *J* = 7.0, 2.5 Hz, 1H), 2.22 (dtd, *J* = 12.2, 3.8, 1.9 Hz, 1H), 1.68 – 1.62 (m, 2H), 1.43 – 1.36 (m, 1H), 1.31 (ddt, *J* = 8.4, 4.8, 1.5 Hz, 1H), 1.03 – 0.97 (m, 1H), 0.94 (dd, *J* = 6.8, 4.4 Hz, 6H), 0.90 (dd, *J* = 5.7, 3.7 Hz, 2H), 0.49 (d, *J* = 6.9 Hz, 3H). **^{13}C NMR** (126 MHz, $CDCl_3$) δ 145.3, 144.0, 129.7, 129.6, 129.4, 128.9, 128.4, 128.2, 81.3, 77.3, 50.2, 41.9, 36.0, 32.9, 26.5, 24.3, 23.9, 22.8, 17.1.

HRMS (ESI+), *m/z*: calculated for $C_{23}H_{31}O$ $[M + H]^+$: 323.2375, found: 323.2377.

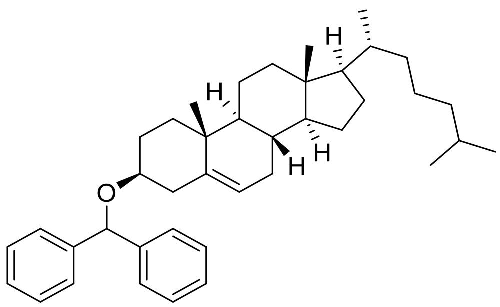

(3*S*,8*S*,9*S*,10*R*,13*R*,14*S*,17*R*)-3-(Benzhydryloxy)-10,13-dimethyl-17-((*R*)-6-methylheptan-2-yl)-2,3,4,7,8,9,10,11,12,13,14,15,16,17-tetradecahydro-1*H*-cyclopenta[a]phenanthrene (110d)

Prepared according to the general procedure A for the Etherification. Following workup, the product was purified by column chromatography (hexane:EtOAc, 20:1) to give the title compound as a white solid (isolated yield: 36%).

^{1}H NMR (500 MHz, $CDCl_3$) δ 7.37 (dt, *J* = 8.1, 1.6 Hz, 4H), 7.33 (t, *J* = 7.5 Hz, 4H), 7.27 – 7.23 (m, 2H), 5.59 (s, 1H), 5.33 – 5.31 (m, 1H), 3.31 (tt, *J* = 11.0, 4.7 Hz, 1H), 2.47 – 2.33 (m, 2H), 2.04 – 1.93 (m, 3H), 1.88 – 1.78 (m, 2H), 1.68 – 1.61 (m, 1H), 1.59 (s, 1H), 1.57 – 1.54 (m, 1H), 1.53 – 1.43 (m, 4H), 1.41 – 1.32 (m, 3H), 1.29 – 1.25 (m, 1H), 1.20 – 1.05 (m, 6H), 1.03 (s, 3H), 1.02 – 0.96 (m, 3H), 0.93 (d, *J* = 6.5 Hz, 3H), 0.89 (dd, *J* = 6.6, 2.3 Hz, 7H), 0.69 (s, 3H). **^{13}C NMR** (126 MHz, $CDCl_3$) δ 144.4, 144.4, 142.5, 129.8, 129.8, 128.7, 128.7, 128.5, 123.0, 81.8, 58.2, 57.6, 51.6, 43.7, 41.2, 41.0, 40.8, 38.7, 38.3, 37.6, 37.2, 33.4, 33.3, 30.0, 29.7, 29.5, 25.7, 25.2, 24.3, 24.0, 22.5, 20.9, 20.2, 13.3.

HRMS (ESI+), *m/z*: calculated for $C_{40}H_{57}O$ $[M + H]^+$: 553.4409, found: 553.4411.

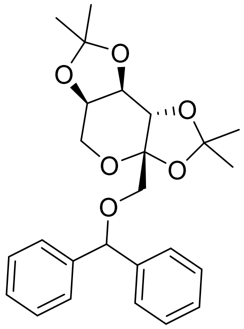

(3*aS*,5*aR*,8*aR*,8*bS*)-3*a*-((Benzhydryloxy)methyl)-2,2,7,7-tetramethyltetrahydro-5*H*-bis([1,3]dioxolo)[4,5-b:4',5'-d]pyran (111d)

Prepared according to the general procedure A for the Etherification. Following workup, the product was purified by column chromatography (hexane:EtOAc, 20:1) to give the title compound as a yellow solid (isolated yield: 56%).

^{1}**H NMR** (500 MHz, $CDCl_3$) δ 7.40 (tt, *J* = 6.3, 1.3 Hz, 4H), 7.33 (ddd, *J* = 7.8, 6.8, 4.9 Hz, 4H), 7.28 – 7.22 (m, 2H), 5.50 (s, 1H), 4.67 – 4.60 (m, 2H), 4.26 (d, *J* = 1.4 Hz, 1H), 3.96 (dd, *J* = 13.0, 1.8 Hz, 1H), 3.74 (d, *J* = 10.3 Hz, 1H), 3.68 (d, *J* = 10.5 Hz, 1H), 3.58 (d, *J* = 10.6 Hz, 1H), 1.59 (s, 3H), 1.46 (s, 3H), 1.36 (d, *J* = 3.9 Hz, 6H). ^{13}**C NMR** (126 MHz, $CDCl_3$) δ 143.5, 143.4, 129.8, 129.7, 128.9, 128.8, 128.6, 128.2, 110.3, 110.0, 104.2, 85.9, 72.5, 71.6, 71.4, 71.4, 68.5, 62.4, 28.1, 27.2, 27.1, 25.5.

HRMS (ESI+), ***m/z***: calculated for $C_{25}H_{31}O_6$ $[M + H]^+$: 427.2121, found: 427.2119.

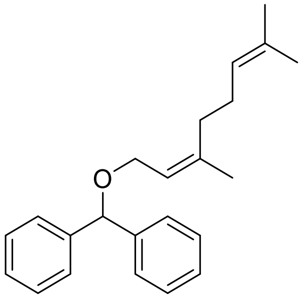

(*Z*)-(((3,7-Dimethylocta-2,6-dien-1-yl)oxy)methylene)dibenzene (112d)

Prepared according to the general procedure A for the Etherification. Following workup, the product was purified by column chromatography (hexane:EtOAc, 20:1) to give the title compound as a yellow oil (isolated yield: 71%, *Z/E* = 76:24).

^{1}**H NMR** (500 MHz, $CDCl_3$) δ 7.43 – 7.40 (m, 4H), 7.39 – 7.34 (m, 4H), 7.31 – 7.26 (m, 2H), 5.54 – 5.48 (m, 1H), 5.47 (s, 0.23H), 5.45 (s, 0.77H), 5.17 (ddp, *J* = 7.0, 5.8, 1.5 Hz, 0.22H), 5.08 (ddt, *J* = 6.9, 4.1, 1.5 Hz, 0.75H), 4.09 (d, *J* = 6.8 Hz, 0.46H), 4.05 (dd, *J* = 6.9, 1.2 Hz, 1.6H), 2.20 – 2.14 (m, 0.49H), 2.13 – 2.02 (m, 3.55H), 1.81 (d, *J* = 1.3 Hz, 2.37H), 1.74 (d, *J* = 1.4 Hz, 0.71H), 1.70 (d, *J* = 1.4 Hz, 2.34H), 1.67 (d, *J* = 1.4 Hz, 0.7H), 1.62 (d, *J* = 1.3 Hz, 0.7H), 1.59 (d, *J* = 1.4 Hz, 2.32H). ^{13}**C NMR (126 MHz, $CDCl_3$)** δ 143.9, 142.0, 141.7, 133.3, 133.1, 130.4, 129.9, 129.8, 128.8, 128.6, 128.6, 127.5, 125.5, 125.3, 123.4, 122.5, 84.1, 83.7, 66.9, 66.7, 43.4, 41.1, 33.7, 28.2, 27.8, 27.2, 27.1, 25.0, 19.2, 19.1, 18.0.

HRMS (ESI+), ***m/z***: calculated for $C_{23}H_{29}O$ $[M + H]^+$: 321.2218, found: 321.2213.

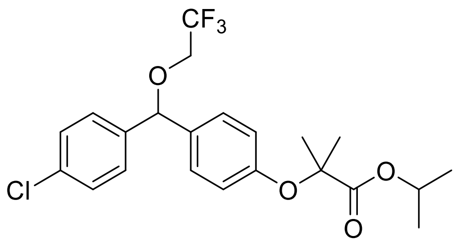

Isopropyl 2-(4-((4-chlorophenyl)(2,2,2-trifluoroethoxy)methyl)phenoxy)-2-methylpropanoate (113d)

Prepared according to the general procedure D for the Etherification. Following workup, the product was purified by column chromatography (hexane:EtOAc, 20:1) to give the title compound as a colorless oil (isolated yield: 80%).

^{1}H NMR (500 MHz, $CDCl_3$) δ 7.35 – 7.32 (m, 2H), 7.29 – 7.24 (m, 2H), 7.18 (d, *J* = 8.7 Hz, 2H), 6.85 – 6.81 (m, 2H), 5.46 (s, 1H), 5.09 (hept, *J* = 6.3 Hz, 1H), 3.79 (q, *J* = 8.6 Hz, 2H), 1.60 (s, 6H), 1.22 (d, *J* = 6.3 Hz, 6H). **^{13}C NMR** (126 MHz, $CDCl_3$) δ 173.5, 155.7, 139.3, 133.7, 133.0, 128.7, 128.3, 128.1, 124.0 (q, $J_{C=F}$ = 278.8 Hz), 118.8, 83.4, 79.2, 69.0, 65.9 (q, $J_{C=F}$ = 34.3 Hz), 25.4, 21.5. **^{19}F NMR** (282 MHz, $CDCl_3$) δ -73.75 (t, *J* = 8.6 Hz).

HRMS (ESI+), *m/z*: calculated for $C_{22}H_{25}ClF_3O_4$ $[M + H]^+$: 445.1393, found: 445.1394.

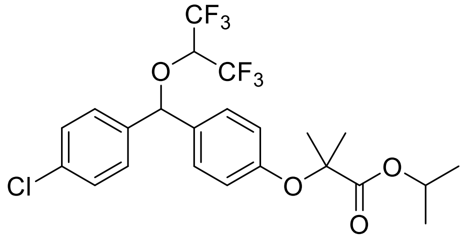

Isopropyl 2-(4-((4-chlorophenyl)((1,1,1,3,3,3-hexafluoropropan-2-yl)oxy)methyl)phenoxy)-2-methylpropanoate (114d)

Prepared according to the general procedure D for the Etherification. Following workup, the product was purified by column chromatography (hexane:EtOAc, 20:1) to give the title compound as a colorless oil (isolated yield: 51%).

^{1}H NMR (500 MHz, $CDCl_3$) δ 7.35 (d, *J* = 8.6 Hz, 2H), 7.28 (d, *J* = 8.5 Hz, 2H), 7.19 (d, *J* = 8.7 Hz, 2H), 6.85 (d, *J* = 8.8 Hz, 2H), 5.74 (s, 1H), 5.09 (hept, *J* = 6.3 Hz, 1H), 4.13 (hept, *J* = 5.9 Hz, 1H), 1.62 (s, 6H), 1.21 (d, *J* = 6.3 Hz, 6H). **^{13}C NMR** (126 MHz, $CDCl_3$) δ 173.4, 156.3, 137.9, 134.3, 130.9, 129.0, 128.7, 128.6, 125.9 – 119.6 (m), 118.6, 84.5, 79.2, 72.3 (dt, $J_{C=F}$ = 65.0, 32.3 Hz), 69.1, 25.4 (d, $J_{C=F}$ = 9.5 Hz), 21.5. **^{19}F NMR** (282 MHz, $CDCl_3$) δ -72.64 (p, *J* = 8.8 Hz), -73.01 (p, *J* = 8.8 Hz).

HRMS (ESI+), *m/z*: calculated for $C_{23}H_{24}ClF_6O_4$ [M + H]$^+$: 513.1267, found: 513.1258.

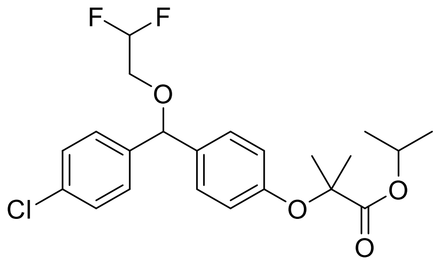

Isopropyl 2-(4-((4-chlorophenyl)(2,2-difluoroethoxy)methyl)phenoxy)-2-methylpropanoate (115d)

Prepared according to the general procedure D for the Etherification. Following workup, the product was purified by column chromatography (hexane:EtOAc, 20:1) to give the title compound as a colorless oil (isolated yield: 86%).

^{1}H NMR (500 MHz, $CDCl_3$) δ 7.34 – 7.30 (m, 2H), 7.29 – 7.24 (m, 2H), 7.18 (d, J = 8.7 Hz, 2H), 6.85 – 6.79 (m, 2H), 5.93 (tt, J = 55.5, 4.1 Hz, 1H), 5.39 (s, 1H), 5.09 (hept, J = 6.2 Hz, 1H), 3.65 (td, J = 13.9, 4.2 Hz, 2H), 1.60 (s, 6H), 1.22 (d, J = 6.3 Hz, 6H). **^{13}C NMR** (126 MHz, $CDCl_3$) δ 173.6, 155.5, 139.8, 133.7, 133.5, 128.6, 128.3, 128.0, 118.8, 114.5 (t, $J_{C\text{=}F}$ = 241.0 Hz), 83.3, 79.1, 69.0, 68.0 (t, $J_{C\text{=}F}$ = 28.0 Hz), 25.4, 21.5. **^{19}F NMR** (376 MHz, $CDCl_3$) δ -124.72 (td, J = 13.8, 2.9 Hz), -124.87 (td, J = 13.9, 2.9 Hz).

HRMS (ESI+), *m/z*: calculated for $C_{22}H_{26}ClF_2O_4$ [M + H]$^+$: 427.1488, found: 427.1486.

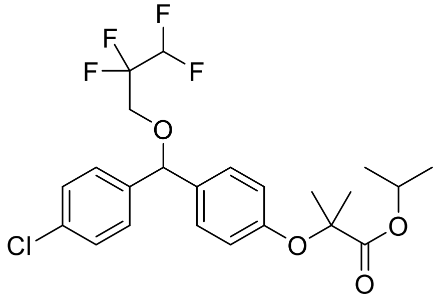

Isopropyl 2-(4-((4-chlorophenyl)(2,2,3,3-tetrafluoropropoxy)methyl)phenoxy)-2-methylpropanoate (116d)

Prepared according to the general procedure D for the Etherification. Following workup, the product was purified by column chromatography (hexane:EtOAc, 20:1) to give the title compound as a colorless oil (isolated yield: 58%).

^{1}H NMR (500 MHz, $CDCl_3$) δ 7.33 (d, J = 8.5 Hz, 2H), 7.24 (d, J = 8.5 Hz, 2H), 7.15 (d, J = 8.7 Hz, 2H), 6.83 (d, J = 8.7 Hz, 2H), 6.00 (tt, J = 53.2, 5.0 Hz, 1H), 5.39 (s, 1H), 5.09 (hept, J = 6.3

Hz, 1H), 3.79 (tt, J = 12.5, 1.6 Hz, 2H), 1.61 (s, 6H), 1.21 (d, J = 6.3 Hz, 6H). **^{13}C NMR** (126 MHz, $CDCl_3$) δ 173.5, 155.7, 139.2, 133.7, 133.0, 128.7, 128.2, 128.0, 118.8, 111.6 – 106.8 (m), 83.7, 79.2, 69.0, 65.8 (t, $J_{C=F}$ = 28.9 Hz), 25.4, 21.5. **^{19}F NMR** (376 MHz, $CDCl_3$) δ -124.96 (dt, J = 7.9, 4.5 Hz), -139.61 (dd, J = 9.5, 4.5 Hz).

HRMS (ESI+), *m/z*: calculated for $C_{23}H_{26}ClF_4O_4$ $[M + H]^+$: 477.1456, found: 477.1451.

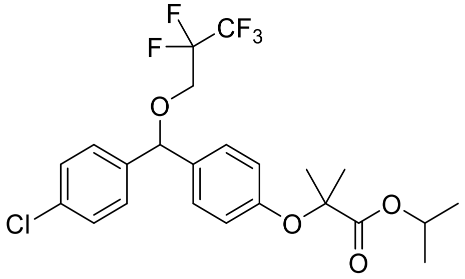

Isopropyl 2-(4-((4-chlorophenyl)(2,2,3,3,3-pentafluoropropoxy)methyl)phenoxy)-2-methylpropanoate (117d)

Prepared according to the general procedure D for the Etherification. Following workup, the product was purified by column chromatography (hexane:EtOAc, 20:1) to give the title compound as a yellow oil (isolated yield: 84%).

^{1}H NMR (500 MHz, $CDCl_3$) δ 7.35 – 7.31 (m, 2H), 7.27 – 7.24 (m, 2H), 7.18 – 7.14 (m, 2H), 6.86 – 6.80 (m, 2H), 5.42 (s, 1H), 5.09 (hept, J = 6.3 Hz, 1H), 3.87 – 3.81 (m, 2H), 1.61 (s, 6H), 1.21 (d, J = 6.3 Hz, 6H). **^{13}C NMR** (126 MHz, $CDCl_3$) δ 173.5, 155.7, 139.2, 133.7, 132.9, 128.7, 128.1 (d, $J_{C=F}$ = 13.4 Hz), 118.8, 118.0 – 114.5 (m), 113.7 – 112.6 (m), 83.7, 79.2, 69.0, 65.0 (t, $J_{C=F}$ = 27.0 Hz), 25.4, 21.5. **^{19}F NMR** (282 MHz, $CDCl_3$) δ -83.36, -123.08 (t, J = 12.8 Hz).

HRMS (ESI+), *m/z*: calculated for $C_{23}H_{25}ClF_5O_4$ $[M + H]^+$: 495.1362, found: 495.1369.

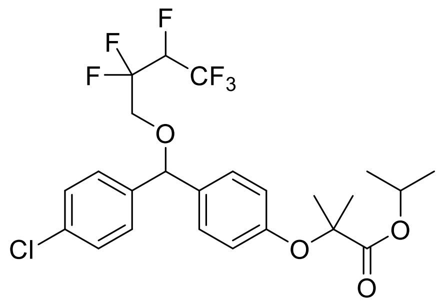

Isopropyl 2-(4-((4-chlorophenyl)(2,2,3,4,4,4-hexafluorobutoxy)methyl)phenoxy)-2-methylpropanoate (118d)

Prepared according to the general procedure D for the Etherification. Following workup, the product was purified by column chromatography (hexane:EtOAc, 20:1) to give the title compound as a colorless oil (isolated yield: 83%).

^{1}H NMR (500 MHz, $CDCl_3$) δ 7.34 (dd, J = 8.5, 3.3 Hz, 2H), 7.25 (dd, J = 8.5, 1.6 Hz, 2H), 7.15 (d, J = 8.5 Hz, 2H), 6.84 (dd, J = 8.7, 2.8 Hz, 2H), 5.41 (s, 1H), 5.21 – 5.02 (m, 2H), 3.84 (dddd, J = 23.1, 11.2, 6.1, 3.6 Hz, 1H), 3.73 (tdd, J = 11.3, 8.3, 2.4 Hz, 1H), 1.61 (s, 6H), 1.22 (d, J = 6.2 Hz, 6H). **^{13}C NMR** (126 MHz, $CDCl_3$) δ 173.5, 155.7 (d, $J_{C\text{-}F}$ = 5.6 Hz), 139.1 (d, $J_{C\text{-}F}$ = 10.0 Hz), 133.8 (d, $J_{C\text{-}F}$ = 6.1 Hz), 132.9 (d, $J_{C\text{-}F}$ = 12.5 Hz), 128.8 (d, $J_{C\text{-}F}$ = 6.0 Hz), 128.2 (d, $J_{C\text{-}F}$ = 3.2 Hz), 128.0 (d, $J_{C\text{-}F}$ = 5.5 Hz), 121.9 (q, $J_{C\text{-}F}$ = 25.5, 22.5 Hz), 120.0 – 118.9 (m), 117.4 – 114.6 (m), 118.8, 83.8 (d, $J_{C\text{-}F}$ = 6.2 Hz), 79.2, 69.0, 66.5 (t, $J_{C\text{-}F}$ = 35.1 Hz), 25.4, 21.5. **^{19}F NMR** (376 MHz, $CDCl_3$) δ -73.96 (qd, J = 10.4, 5.1 Hz), -115.95 – -117.24 (m), -120.18 – -121.44 (m). **HRMS (ESI+), *m/z***: calculated for $C_{24}H_{26}ClF_6O_4$ $[M + H]^+$: 527.1424, found: 527.1419.

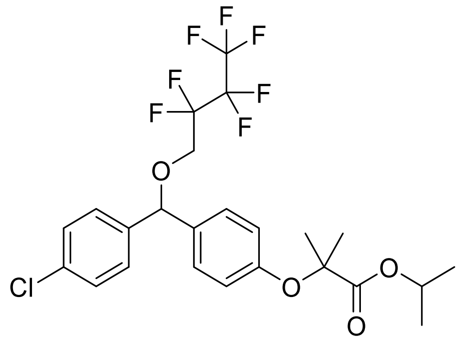

Isopropyl 2-(4-((4-chlorophenyl)(2,2,3,3,4,4,4-heptafluorobutoxy)methyl)phenoxy)-2-methylpropanoate (119d)

Prepared according to the general procedure D for the Etherification. Following workup, the product was purified by column chromatography (hexane:EtOAc, 20:1) to give the title compound as a colorless oil (isolated yield: 80%).

^{1}H NMR (500 MHz, $CDCl_3$) δ 7.33 (d, *J* = 8.5 Hz, 2H), 7.26 (d, *J* = 8.5 Hz, 2H), 7.17 (d, *J* = 8.7 Hz, 2H), 6.84 (d, *J* = 8.7 Hz, 2H), 5.43 (s, 1H), 5.09 (hept, *J* = 6.3 Hz, 1H), 3.88 (t, *J* = 13.4 Hz, 2H), 1.61 (s, 6H), 1.21 (d, *J* = 6.3 Hz, 6H). **^{13}C NMR** (126 MHz, $CDCl_3$) δ 173.5, 155.7, 139.2, 133.7, 132.9, 128.7, 128.2 (d, $J_{C\text{–}F}$ = 10.9 Hz), 118.7, 116.8 (d, $J_{C\text{–}F}$ = 49.1 Hz), 114.9, 112.7 (d, $J_{C\text{–}F}$ = 42.8 Hz), 83.8, 79.2, 69.0, 65.2 (t, $J_{C\text{–}F}$ = 26.3 Hz), 25.4, 21.5. **^{19}F NMR** (282 MHz, $CDCl_3$) δ -80.92 (t, *J* = 9.3 Hz), -120.17, -127.37.

HRMS (ESI+), *m/z*: calculated for $C_{24}H_{25}ClF_7O_4$ $[M + H]^+$: 545.1330, found: 545.1326.

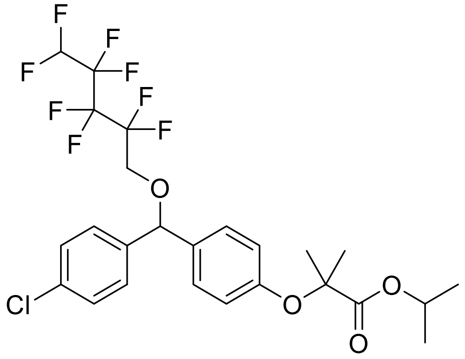

Isopropyl 2-(4-((4-chlorophenyl)((2,2,3,3,4,4,5,5-octafluoropentyl)oxy)methyl)phenoxy)-2-methylpropanoate (120d)

Prepared according to the general procedure D for the Etherification. Following workup, the product was purified by column chromatography (hexane:EtOAc, 20:1) to give the title compound as a colorless oil (isolated yield: 88%).

^{1}H NMR (500 MHz, $CDCl_3$) δ 7.33 (d, *J* = 8.6 Hz, 2H), 7.26 (d, *J* = 8.5 Hz, 2H), 7.17 (d, *J* = 8.6 Hz, 2H), 6.84 (d, *J* = 8.7 Hz, 2H), 6.06 (tt, *J* = 52.0, 5.5 Hz, 1H), 5.43 (s, 1H), 5.09 (hept, *J* = 6.3 Hz, 1H), 3.89 (tt, *J* = 13.7, 1.6 Hz, 2H), 1.61 (s, 6H), 1.21 (d, *J* = 6.3 Hz, 6H). **^{13}C NMR** (126 MHz, $CDCl_3$) δ 173.5, 155.7, 139.2, 133.7, 132.9, 128.7, 128.2, 128.1, 118.8, 116.6 (d, $J_{C\text{–}F}$ = 257.0 Hz), 111.1 – 109.0 (m), 108.2 – 107.2 (m), 106.2 – 104.6 (m), 83.8, 79.2, 69.0, 65.3 (t, $J_{C\text{–}F}$ = 26.0 Hz), 25.4, 21.5. **^{19}F NMR** (376 MHz, $CDCl_3$) δ -119.36 (p, *J* = 12.9 Hz), -125.24 (t, *J* = 8.9 Hz), -130.29 (td, *J* = 11.1, 5.5 Hz), -137.32 (dq, *J* = 52.0, 5.8, 3.8 Hz).

HRMS (ESI+), *m/z*: calculated for $C_{25}H_{26}ClF_8O_4$ $[M + H]^+$: 577.1392, found: 577.1386.

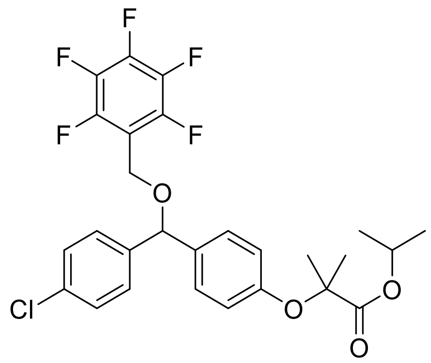

Isopropyl 2-(4-((4-chlorophenyl)((perfluorophenyl)methoxy)methyl)phenoxy)-2-methylpropanoate (121d)

Prepared according to the general procedure D for the Etherification. Following workup, the product was purified by column chromatography (hexane:EtOAc, 20:1) to give the title compound as a yellow oil (isolated yield: 81%).

^{1}H NMR (500 MHz, $CDCl_3$) δ 7.32 (d, J = 8.5 Hz, 2H), 7.27 (d, J = 8.5 Hz, 2H), 7.19 – 7.14 (m, 2H), 6.85 – 6.80 (m, 2H), 5.40 (s, 1H), 5.09 (hept, J = 6.3 Hz, 1H), 4.58 (t, J = 1.8 Hz, 2H), 1.60 (s, 6H), 1.22 (d, J = 6.3 Hz, 6H). **^{13}C NMR** (126 MHz, $CDCl_3$) δ 173.6, 155.4, 146.6 (d, $J_{C=F}$ = 3.0 Hz), 144.7 (d, $J_{C=F}$ = 6.9 Hz), 139.9, 138.4 (t, $J_{C=F}$ = 17.6 Hz), 137.2 – 135.7 (m), 133.9, 133.5, 128.6, 128.4, 128.0, 118.8, 111.3 (td, $J_{C=F}$ = 18.2, 3.3 Hz), 82.8, 79.1, 69.0, 57.9, 25.4 (d, $J_{C=F}$ = 3.6 Hz), 21.5. **^{19}F NMR** (376 MHz, $CDCl_3$) δ -142.63 (dd, J = 22.7, 8.8 Hz), -153.73 (t, J = 20.6 Hz), -161.90 – -162.23 (m).

HRMS (ESI+), *m/z*: calculated for $C_{27}H_{25}ClF_5O_4$ $[M + H]^+$: 543.1362, found: 543.1376.

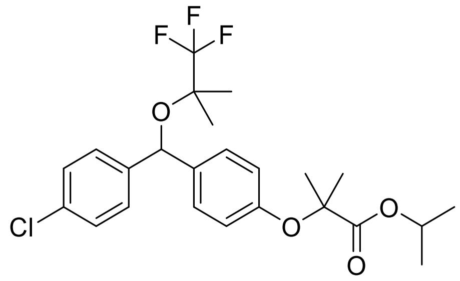

Isopropyl 2-(4-((4-chlorophenyl)((1,1,1-trifluoro-2-methylpropan-2-yl)oxy)methyl)phenoxy)-2-methylpropanoate (122d)

Prepared according to the general procedure D for the Etherification. Following workup, the product was purified by column chromatography (hexane:EtOAc, 20:1) to give the title compound as a colorless oil (isolated yield: 78%).

^{1}H NMR (500 MHz, $CDCl_3$) δ 7.30 – 7.24 (m, 4H), 7.18 – 7.14 (m, 2H), 6.82 – 6.78 (m, 2H), 5.70 (s, 1H), 5.09 (hept, *J* = 6.3 Hz, 1H), 1.59 (s, 6H), 1.34 (d, *J* = 5.8 Hz, 6H), 1.21 (d, *J* = 6.3 Hz, 6H). **^{13}C NMR** (126 MHz, $CDCl_3$) δ 173.6, 155.0, 142.2, 136.4, 133.0, 128.5, 128.0, 127.5, 127.5 – 122.7 (m), 118.7, 79.1, 76.3, 68.9, 25.4 (d, $J_{C\text{=}F}$ = 6.6 Hz), 21.5, 21.1, 20.9. **^{19}F NMR** (282 MHz, $CDCl_3$) δ -81.61.

HRMS (ESI+), *m/z*: calculated for $C_{24}H_{29}ClF_3O_4$ $[M + H]^+$: 473.1706, found: 473.1723.

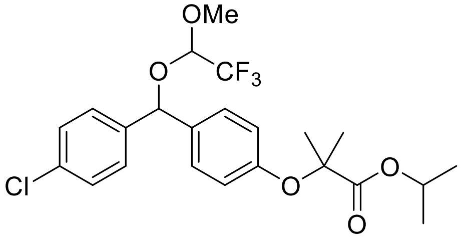

Isopropyl 2-(4-((4-chlorophenyl)(2,2,2-trifluoro-1-methoxyethoxy)methyl)phenoxy)-2-methylpropanoate (123d)

Prepared according to the general procedure D for the Etherification. Following workup, the product was purified by column chromatography (hexane:EtOAc, 20:1) to give the title compound as a colorless oil (isolated yield: 70%, d.r. = 4:1).

^{1}H NMR (500 MHz, $CDCl_3$) δ 7.29 (d, *J* = 8.9 Hz, 5H), 7.22 – 7.19 (m, 0.4H),7.17 (d, *J* = 8.3 Hz, 1.6H), 6.87 – 6.84 (m, 0.4H), 6.82 (d, *J* = 8.7 Hz, 1.6H), 5.71 (s, 0.2H), 5.17 (s, 0.8H), 5.09 (pd, *J* = 6.3, 3.3 Hz, 1H), 3.43 (s, 0.6H), 3.35 (s, 2.4H), 1.62 (s, 1.2H), 1.59 (s, 4.8H), 1.21 (d, *J* = 6.3 Hz, 4.8H), 1.20 (d, *J* = 2.1 Hz, 1.2H). **^{13}C NMR** (126 MHz, $CDCl_3$) δ 173.7, 155.1, 140.8, 134.9, 133.1, 128.5, 128.2, 127.8, 118.8, 96.0 – 94.7 (m), 84.2, 79.1, 68.9, 56.9, 25.4 (d, $J_{C\text{=}F}$ = 3.0 Hz), 21.5. **^{19}F NMR** (376 MHz, $CDCl_3$) δ -79.80 (d, *J* = 4.1 Hz).

HRMS (ESI+), *m/z*: calculated for $C_{23}H_{27}ClF_3O_5$ $[M + H]^+$: 475.1499, found: 475.1488.

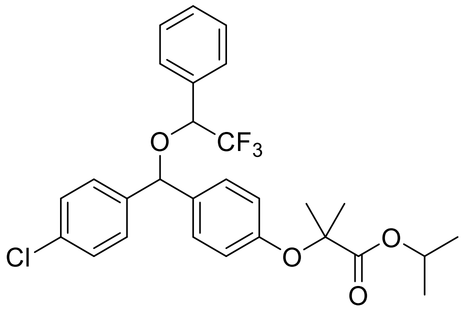

Isopropyl 2-(4-((4-chlorophenyl)(2,2,2-trifluoro-1-phenylethoxy)methyl)phenoxy)-2-methylpropanoate (124d)

Prepared according to the general procedure D for the Etherification. Following workup, the product was purified by column chromatography (hexane:EtOAc, 20:1) to give the title compound as a colorless oil (isolated yield: 87%, d.r. = 1:1).

^{1}H NMR (500 MHz, $CDCl_3$) δ 7.46 – 7.41 (m, 5H), 7.38 (d, J = 8.4 Hz, 1H), 7.30 – 7.26 (m, 2H), 7.24 (d, J = 8.6 Hz, 1H), 7.17 (d, J = 8.6 Hz, 1H), 7.12 (d, J = 8.7 Hz, 1H), 6.87 (d, J = 8.6 Hz, 1H), 6.77 (d, J = 8.7 Hz, 1H), 5.36 (s, 0.5H), 5.29 (s, 0.5H), 5.10 (dp, J = 15.5, 6.3 Hz, 1H), 4.61 (p, J = 6.7 Hz, 1H), 1.64 (s, 3H), 1.59 (d, J = 1.5 Hz, 3H), 1.22 (dt, J = 6.3, 3.9 Hz, 6H). **^{13}C NMR** (126 MHz, $CDCl_3$) δ 173.6 (d, $J_{C\text{=}F}$ = 7.7 Hz), 155.9, 155.2, 140.0, 138.7, 134.0 (d, $J_{C\text{=}F}$ = 16.0 Hz), 133.3, 132.4, 132.3 (d, $J_{C\text{=}F}$ = 5.1 Hz), 129.72 (d, $J_{C\text{=}F}$ = 7.6 Hz), 129.0 (d, $J_{C\text{=}F}$ = 6.7 Hz), 128.8 (d, $J_{C\text{=}F}$ = 2.6 Hz), 128.7, 128.6 (d, $J_{C\text{=}F}$ = 4.9 Hz), 128.4, 128.0, 127.7, 123.9 (dd, $J_{C\text{=}F}$ = 281.7, 10.0 Hz), 118.7 (d, $J_{C\text{=}F}$ = 10.6 Hz), 80.4, 79.9, 79.1 (d, $J_{C\text{=}F}$ = 6.3 Hz), 69.0 (d, $J_{C\text{=}F}$ = 5.7 Hz), 25.6 – 25.3 (m), 21.5 (d, $J_{C\text{=}F}$ = 5.2 Hz). **^{19}F NMR** (282 MHz, $CDCl_3$) δ -75.97 (d, J = 6.6 Hz), -76.12 (d, J = 6.7 Hz).

HRMS (ESI+), *m/z*: calculated for $C_{28}H_{29}ClF_3O_4$ $[M + H]^+$: 521.1706, found: 521.1722.

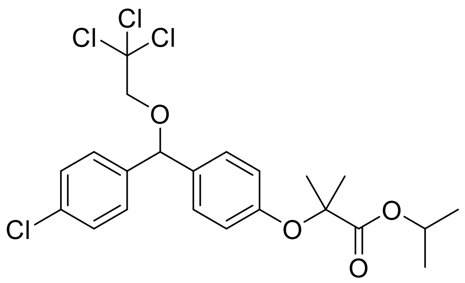

Isopropyl 2-(4-((4-chlorophenyl)(2,2,2-trichloroethoxy)methyl)phenoxy)-2-methylpropanoate (125d)

Prepared according to the general procedure D for the Etherification. Following workup, the product was purified by column chromatography (hexane:EtOAc, 20:1) to give the title compound as a colorless oil (isolated yield: 53%).

^{1}H NMR (500 MHz, $CDCl_3$) δ 7.35 – 7.30 (m, 4H), 7.22 (d, *J* = 8.6 Hz, 2H), 6.86 – 6.82 (m, 2H), 5.66 (s, 1H), 5.09 (hept, *J* = 6.3 Hz, 1H), 4.04 (s, 2H), 1.61 (s, 6H), 1.22 (d, *J* = 6.3 Hz, 6H). **^{13}C NMR** (126 MHz, $CDCl_3$) δ 173.6, 155.6, 139.6, 133.7, 133.4, 128.6, 128.5, 128.2, 118.7, 97.0, 83.7, 80.7, 79.2, 69.0, 25.4, 21.6.

HRMS (ESI+), *m/z*: calculated for $C_{22}H_{25}Cl_4O_4$ $[M + H]^+$: 493.0507, found: 493.0513.

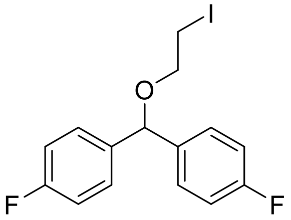

4,4'-((2-Iodoethoxy)methylene)bis(fluorobenzene) (126d)

Prepared according to the general procedure A for the Etherification. Following workup, the product was purified by column chromatography (hexane:EtOAc, 20:1) to give the title compound as a colorless oil (isolated yield: 53%).

^{1}H NMR (600 MHz, $CDCl_3$) δ 7.32 – 7.26 (m, 4H), 7.01 (t, *J* = 8.7 Hz, 4H), 5.38 (s, 1H), 3.68 (t, *J* = 6.5 Hz, 2H), 3.28 (t, *J* = 6.5 Hz, 2H). **^{13}C NMR** (126 MHz, $CDCl_3$) δ 162.3 (d, $J_{C\text{=}F}$ = 246.4 Hz), 137.4 (d, $J_{C\text{=}F}$ = 3.3 Hz), 128.7 (d, $J_{C\text{=}F}$ = 8.1 Hz), 115.4 (d, $J_{C\text{=}F}$ = 21.5 Hz), 82.4, 69.5, 3.1.

HRMS (ESI+), *m/z*: calculated for $C_{15}H_{14}F_2IO$ $[M + H]^+$: 375.0057, found: 375.0069.

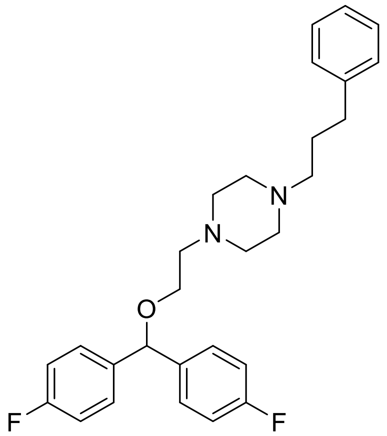

1-(2-(Bis(4-fluorophenyl)methoxy)ethyl)-4-(3-phenylpropyl)piperazine (126e)

Following workup, the product was purified by column chromatography (hexane:EtOAc, 10:1) to give the title compound as a white solid (isolated yield: 53%).

^{1}H NMR (400 MHz, DMSO-d_6) δ 7.49 (dd, J = 8.4, 5.4 Hz, 4H), 7.31 (d, J = 7.4 Hz, 2H), 7.21 (dt, J = 26.5, 8.0 Hz, 7H), 5.61 (s, 1H), 3.89 – 3.38 (m, 10H), 3.12 (s, 2H), 2.65 (t, J = 7.8 Hz, 2H), 2.51 (q, J = 2.1 Hz, 2H), 2.11 – 1.86 (m, 2H). **^{13}C NMR** (101 MHz, DMSO-d_6) δ 163.2, 160.7, 141.0, 138.3, 129.2 (d, $J_{C=F}$ = 8.1 Hz), 128.8 (d, $J_{C=F}$ = 15.0 Hz), 126.6, 115.7 (d, $J_{C=F}$ = 21.3 Hz), 82.0, 63.3, 55.9, 55.4, 49.1, 48.5, 32.4, 25.3. **^{19}F NMR** (376 MHz, DMSO-d_6) δ -114.89.

HRMS (ESI+), *m/z*: calculated for $C_{28}H_{33}F_2N_2O$ $[M + H]^+$: 451.2561, found: 451.2573.

7 Characterization data of synthesized starting material

The NMR dates of **1b**, **48b**, **49b**, **50b**, **51b**, **52b**, **53b**, **54b**, **56b**, **57b**, **58b**, **59b**, **60b**, **61b**, **75b**, **77b**, **78b**, **79b**, **80b**, **81b, 85b**, **88b**, **89b**, **90b**, **93b**, **95b**, **96b**, **98b**, **99b**, **101b**, **102b**, **113b** had been reported by our group's previous articles.[1,8-11]

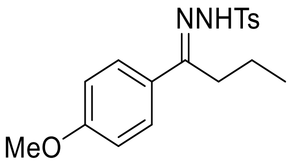

(*Z*)-4-Methyl-*N*'-(1-phenyldodecylidene)benzenesulfonohydrazide (55b)

Prepared according to the synthesis of hydrazone. Following the general procedure, was obtained as a white solid (isolated yield: 92%).

^{1}H NMR (500 MHz, $CDCl_3$) δ 7.93 (d, *J* = 8.3 Hz, 2H), 7.85 (s, 1H), 7.61 (d, *J* = 8.9 Hz, 2H), 7.34 (d, *J* = 8.0 Hz, 2H), 6.87 (d, *J* = 8.9 Hz, 2H), 3.83 (s, 3H), 2.59 – 2.51 (m, 2H), 2.43 (s, 3H), 1.57 – 1.45 (m, 2H), 0.95 (t, *J* = 7.4 Hz, 3H). **^{13}C NMR** (126 MHz, $CDCl_3$) δ 165.5, 160.6, 148.7, 140.1, 134.3, 133.7, 132.8, 132.6, 118.4, 60.0, 33.3, 26.3, 24.1, 18.8.

HRMS (ESI+), *m/z*: calculated for $C_{18}H_{23}N_2O_3S$ $[M + H]^+$: 347.1429, found: 347.1428.

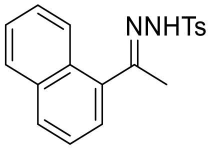

(*Z*)-4-Methyl-*N*'-(1-(naphthalen-1-yl)ethylidene)benzenesulfonohydrazide (83b)

Prepared according to the synthesis of hydrazone. Following the general procedure, was obtained as a white solid (isolated yield: 79%). **89b** was known in the published literature.[13]

^{1}H NMR (500 MHz, $CDCl_3$) δ 7.92 (d, *J* = 8.3 Hz, 3H), 7.85 – 7.79 (m, 3H), 7.48 – 7.40 (m, 2H), 7.36 – 7.32 (m, 4H), 2.46 (s, 3H), 2.31 (s, 3H).

HRMS (ESI+), *m/z*: calculated for $C_{19}H_{19}N_2O_2S$ $[M + H]^+$: 339.1167, found: 339.1154.

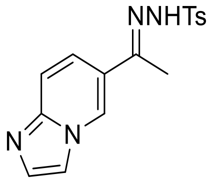

(*Z*)-*N*'-(1-(Imidazo[1,2-*a*]pyridin-6-yl)ethylidene)-4-methylbenzenesulfonohydrazide (92b)

Prepared according to the synthesis of hydrazone. Following the general procedure, was obtained as a white solid (isolated yield: 69%).

^{1}**H NMR** (500 MHz, DMSO-d_6) δ 10.60 (s, 1H), 8.86 (dd, *J* = 1.9, 1.0 Hz, 1H), 7.91 (t, *J* = 1.0 Hz, 1H), 7.82 (d, *J* = 8.4 Hz, 2H), 7.55 (td, *J* = 5.1, 1.8 Hz, 2H), 7.49 (dt, *J* = 9.5, 0.9 Hz, 1H), 7.42 – 7.38 (m, 2H), 2.35 (s, 3H), 2.17 (s, 3H).

HRMS (ESI+), *m/z*: calculated for $C_{16}H_{18}N_4O_2S$ $[M + H]^+$: 329.1067, found: 329.1076.

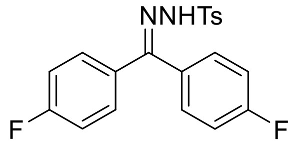

(*Z*)-4-(1-(2-Tosylhydrazono)ethyl)phenyl 2-hydroxybenzoate (126b)

Prepared according to the synthesis of hydrazone. Following the general procedure, was obtained as a yellow solid (isolated yield: 67%). **136b** was known in the published literature.[14]

^{1}**H NMR** (500 MHz, $CDCl_3$) δ 7.87 (d, *J* = 8.4 Hz, 2H), 7.54 (s, 1H), 7.44 – 7.40 (m, 2H), 7.37 (d, *J* = 8.1 Hz, 2H), 7.25 (t, *J* = 8.6 Hz, 2H), 7.17 (dd, *J* = 8.5, 5.4 Hz, 2H), 7.03 – 6.96 (m, 2H), 2.46 (s, 3H).

HRMS (ESI+), *m/z*: calculated for $C_{20}H_{17}F_2N_2O_2S$ $[M + H]^+$: 387.0979, found: 387.0973.

8 Cartesian coordinates of the optimized structures

Cartesian coordinates of the optimized structures:

Int1

E = -1351.99621902 a.u.

-1 1

C	-12.94694400	12.14384700	7.80966800
C	-11.65540700	11.80052000	7.42129900
C	-10.85339400	12.66720700	6.65855600
C	-11.41217900	13.91079000	6.31005900
C	-12.70232100	14.26532100	6.69294800
C	-13.48271900	13.38560500	7.44422900
H	-13.52528900	11.43594300	8.40356400
H	-11.22296500	10.84183100	7.71056100
C	-9.48790600	12.28492100	6.25271500
H	-13.13210200	15.22992800	6.41792100
N	-9.10133300	11.08132100	6.53275400
N	-7.87392400	10.70032600	6.19148200
S	-7.61755400	9.15295100	6.62910200
O	-8.13785700	8.80126000	7.95353100
O	-6.22297600	8.84404700	6.31368100
C	-8.60670700	8.17585400	5.47857700
C	-9.95516500	7.94443800	5.74763600
C	-8.02878400	7.69864900	4.30640700
C	-10.72563500	7.24060100	4.82621900
H	-10.37199100	8.31389000	6.68558700
C	-8.80927500	6.98915300	3.39428000
H	-6.96657900	7.88498600	4.13891700
C	-10.16726600	6.75553700	3.63612600
H	-11.78356400	7.05748000	5.03368300
H	-8.35591900	6.60477300	2.47643700
C	-11.02054400	6.01955300	2.63416100
H	-11.62975600	6.72160100	2.04278600
H	-10.40274400	5.44263000	1.93204300
H	-11.71317900	5.32572500	3.13246100
H	-10.82615100	14.61849200	5.72147000
C	-8.61088500	13.28789500	5.55125300
H	-8.49909600	14.21179400	6.14285000
H	-7.62521200	12.83448300	5.39577800
H	-9.02770900	13.57616000	4.57104500
O	-14.73902100	13.81066500	7.77954300
C	-15.53437400	12.94673700	8.53226000
H	-16.49302900	13.45453600	8.69714100
H	-15.72081600	11.99396000	8.00572100
H	-15.07961300	12.71556100	9.51179500

^{3}Int1

E = -1351.92214000 a.u.

-1 3

C	-12.99186200	11.47973800	6.11364700
C	-11.68993800	11.58250700	5.65622400
C	-10.69888800	12.31913600	6.38252200
C	-11.13479700	12.95443400	7.58607800
C	-12.44291100	12.84541100	8.03023500
C	-13.38600600	12.10579500	7.30493400
H	-13.73563600	10.90533700	5.55455200
H	-11.39031900	11.09742400	4.72586500
C	-9.35411000	12.40018100	5.92729200
H	-12.75820800	13.32485000	8.96062000
N	-9.01868700	11.85282400	4.74099100
N	-7.90312400	11.42036300	4.26104800
S	-7.25454700	10.09365000	5.15482700
O	-7.13632400	10.43420400	6.57680300
O	-6.07423800	9.63192000	4.42869200
C	-8.50561500	8.82432700	5.03736900
C	-9.64766500	8.91585800	5.83129400
C	-8.37341000	7.82345300	4.07688000
C	-10.66929200	7.98424100	5.65327400
H	-9.73002600	9.71203200	6.57503700
C	-9.39529500	6.89136800	3.92429000
H	-7.46691300	7.79594300	3.46991200
C	-10.55726200	6.96015300	4.70656200
H	-11.57521300	8.06709400	6.25876200
H	-9.29742600	6.09738200	3.17912900
C	-11.65174000	5.93638500	4.54394600
H	-11.75230700	5.62305000	3.49505500
H	-11.43865300	5.03366100	5.13879700
H	-12.61917100	6.33355900	4.88000800
H	-10.41990800	13.52716300	8.17898300
C	-8.31722500	13.19332200	6.68030200
H	-8.20874900	12.81231000	7.70765200
H	-7.34401000	13.08557300	6.18846400
H	-8.58302400	14.26387400	6.73791700
O	-14.69097100	11.99953100	7.75570600
C	-15.53420500	13.00625900	7.26949500
H	-16.53971600	12.83389900	7.68019900
H	-15.18923100	14.01031400	7.57702300
H	-15.59125800	12.99244500	6.16586000

^{3}Int2

E = -2815.28814351 a.u.

-1 3

C	-1.71206600	10.59545700	3.58753800
C	-1.70079300	10.16474800	4.91070700
C	-0.48547400	10.04186600	5.64679300
C	0.71356700	10.39196700	4.95363500
C	0.69456500	10.80732100	3.63618200
C	-0.51650300	10.91530100	2.93136900
H	-2.67955400	10.69086400	3.09381600
H	-2.66690600	9.96549400	5.38922400
C	-0.46159400	9.62737900	7.01088300
H	1.61679900	11.06398200	3.11211900
N	-1.65242900	9.45301900	7.69652300
N	-1.99468600	8.44267100	8.46432500
S	-1.44241100	6.89329800	8.05541000
O	-1.74343300	6.04567400	9.20408200
O	-0.09550900	6.88480600	7.48141900
C	-2.58252200	6.43074600	6.75765900
C	-3.70408600	5.66289000	7.07015900
C	-2.43761200	7.00949400	5.49641800
C	-4.70729900	5.50508100	6.11412300
H	-3.77190800	5.21330000	8.06295500
C	-3.45791200	6.86007600	4.56125900
H	-1.53820300	7.58420500	5.26637600
C	-4.61182600	6.12159800	4.85806800
H	-5.58901600	4.90370300	6.34903200
H	-3.36212700	7.34428000	3.58669700
C	-5.73505000	6.02897000	3.85956500
H	-6.41307200	5.19781600	4.09518700
H	-6.32468300	6.95875400	3.86305100
H	-5.34974600	5.88659200	2.84032700
H	1.67238800	10.31795500	5.46844100
C	0.81101100	9.63233400	7.81180700
H	1.24974600	10.64327700	7.89509800
H	0.61257900	9.24388200	8.81791000
H	1.55491500	8.96972300	7.34507200
O	-0.43351600	11.34732000	1.64010000
C	-1.62762500	11.47708700	0.92590200
H	-1.36321900	11.83133200	-0.07797200
H	-2.15900100	10.51334900	0.83829200
H	-2.31021200	12.20554500	1.39661100
C	-5.33751500	11.24760000	7.04775100
O	-4.53079700	10.62281500	6.20140100
O	-5.10246400	12.47639700	7.30655200
O	-6.24929400	10.57226700	7.62741400
K	-2.68023100	12.07659400	7.00290400
K	-4.72365100	8.60292200	7.63875700

^{3}TS1

E = -2815.28368955 a.u.

-1 3

C	-1.65348700	10.61782700	3.48014800
C	-1.67488900	10.14120300	4.78898900
C	-0.49843900	10.10195400	5.58653300
C	0.70073600	10.56441800	4.97351700
C	0.71822900	11.02450000	3.66888800
C	-0.45725200	11.05962200	2.90223700
H	-2.59642100	10.64632400	2.93345300
H	-2.64148000	9.84879200	5.21403100
C	-0.50606100	9.62649700	6.94152700
H	1.64344500	11.37103300	3.20544200
N	-1.68606100	9.25414300	7.50036200
N	-1.93837000	8.51966900	8.48935900
S	-1.38843100	6.66859500	8.11175700
O	-1.66867400	5.83840500	9.28941300
O	-0.03746300	6.69908700	7.52804300
C	-2.53868600	6.23323200	6.82992500
C	-3.69360500	5.51494000	7.15474800
C	-2.42397100	6.88829500	5.59735500
C	-4.74223800	5.46238900	6.23883000
H	-3.74715400	5.02035100	8.12706100
C	-3.49605700	6.85008000	4.70899000
H	-1.50799600	7.43313700	5.35678800
C	-4.67282000	6.15252200	5.01677100
H	-5.64351100	4.89310800	6.48002000
H	-3.42438600	7.39450400	3.76453500
C	-5.84848800	6.19048400	4.07680200
H	-6.56080000	5.38311600	4.29376300
H	-6.38203300	7.14933900	4.17210300
H	-5.52400500	6.09783500	3.03092400
H	1.63427200	10.54737800	5.53774900
C	0.74734800	9.58058200	7.77081000
H	1.19753800	10.58082900	7.89691600
H	0.52069200	9.16176400	8.75819700
H	1.49466100	8.92254000	7.30140900
O	-0.34295700	11.54188900	1.63075800
C	-1.50344100	11.59286900	0.85427900
H	-1.21742300	12.00197700	-0.12243300
H	-1.94323400	10.59072400	0.70887900
H	-2.27146800	12.24437100	1.30614600
C	-5.36612200	11.12951500	6.87652400
O	-4.54372900	10.53100500	6.02539100
O	-5.17349100	12.36802900	7.12706800

O	-6.24851900	10.42525400	7.46438600
K	-2.75959400	12.10426100	6.70565700
K	-4.66253700	8.50336100	7.46039300

^{3}Int3

E = -2815.31366454 a.u.

-1 3

C	-1.78271800	10.61829400	3.84146300
C	-1.78011300	10.67008900	5.23024900
C	-0.65239000	10.24768700	5.96837600
C	0.47115000	9.75403100	5.26023000
C	0.47366300	9.72217400	3.88020200
C	-0.65264400	10.15253100	3.15631000
H	-2.68683000	10.92490700	3.31697600
H	-2.70024500	10.99002200	5.73556000
C	-0.63698400	10.30179400	7.41017200
H	1.32565900	9.33169700	3.32387300
N	-1.69378200	10.92585800	8.01608500
N	-1.89289500	11.13857700	9.18718100
S	-0.73607100	6.63769500	7.32635600
O	-0.60105100	5.29808600	7.98532000
O	0.46499300	7.09285400	6.51937200
C	-2.02906700	6.36050600	6.04027200
C	-3.15149100	5.60878900	6.39573000
C	-2.07615100	7.17328100	4.90804900
C	-4.34794000	5.76619200	5.69440300
H	-3.07329500	4.95426800	7.26875100
C	-3.27742000	7.32536300	4.21037200
H	-1.17499500	7.72542200	4.62756200
C	-4.44750500	6.67441100	4.62744500
H	-5.24171700	5.22141600	6.01045800
H	-3.32527400	8.01521900	3.36308700
C	-5.78682400	7.03827700	4.04478300
H	-6.48715600	6.19205000	4.08439000
H	-6.21072000	7.86805100	4.64010900
H	-5.69413400	7.37071200	3.00119800
H	1.32249500	9.34899800	5.80390300
C	0.49840600	9.77729200	8.22594100
H	1.36122200	10.46303800	8.16226200
H	0.20033300	9.69059000	9.27747900
H	0.80332100	8.79423100	7.82871500
O	-0.56502700	10.06966500	1.81116300
C	-1.69020500	10.42045900	1.05101700
H	-1.42250200	10.26078300	0.00034900
H	-2.56000900	9.79160600	1.30246500
H	-1.96549200	11.47791100	1.19845300

C	-5.74135000	10.54598300	6.88381100
O	-4.51927400	10.87239400	6.48010200
O	-6.41146400	11.42689400	7.52021100
O	-6.13771300	9.35283500	6.69441900
K	-4.33299400	12.53269500	8.26714200
K	-3.76417300	8.54612000	7.05817200

^{3}Int4

E = -532.978171399 a.u.

0 3

C	-13.12942500	12.10397300	6.09819500
C	-11.83207600	11.98989600	5.61906100
C	-10.72703500	12.45397900	6.36341800
C	-10.98646600	13.04398500	7.62398300
C	-12.27500900	13.16103800	8.10715100
C	-13.36288700	12.69270700	7.35079200
H	-13.95415800	11.73277000	5.49177900
H	-11.65048200	11.53295400	4.64627600
C	-9.38195700	12.33475000	5.86449600
H	-12.48203000	13.61420300	9.07670500
N	-9.21813400	11.74701200	4.62489200
N	-8.18194900	11.56487700	4.05405600
H	-10.15994500	13.41542500	8.23091400
C	-8.18947100	12.81565800	6.62882800
H	-8.11229200	12.30531000	7.60167600
H	-7.27469900	12.62312700	6.05690500
H	-8.25954400	13.89593300	6.83099100
O	-14.58113300	12.84821000	7.89966700
C	-15.70470100	12.39643400	7.18721400
H	-16.57912100	12.61783200	7.80823100
H	-15.80499800	12.91850200	6.22176900
H	-15.65533400	11.31049700	7.00557600

^{3}TS2

E = -532.954282058 a.u.

0 3

C	-13.13298000	12.11515600	6.12169000
C	-11.82712400	12.01083500	5.66136700
C	-10.73307600	12.48329200	6.42522800
C	-11.02754400	13.07142900	7.68833000
C	-12.32512900	13.17522800	8.14594400
C	-13.39590500	12.69882700	7.36958700
H	-13.94367500	11.73786400	5.49988500
H	-11.63545900	11.55564900	4.69050800
C	-9.39446800	12.39229400	5.98601800
H	-12.55343100	13.62510500	9.11261700

N	-9.19722500	11.59359800	4.30805500
N	-8.16346500	11.48301100	3.88310500
H	-10.21049000	13.44756500	8.30656500
C	-8.12523000	12.80969800	6.60360300
H	-7.95386100	12.29959100	7.56744600
H	-7.28102100	12.56647100	5.93980900
H	-8.10247600	13.89694800	6.79315500
O	-14.62850600	12.84332500	7.89857600
C	-15.73087500	12.38277200	7.16262500
H	-16.62137000	12.59327500	7.76471800
H	-15.81725300	12.90529400	6.19558300
H	-15.66796700	11.29756100	6.97824000

^{3}Int5

E = -532.976959004 a.u.

0 3

C	-13.12036800	12.14676400	6.15784000
C	-11.80104000	12.06813900	5.73058400
C	-10.73192400	12.54537600	6.52678600
C	-11.06550400	13.11258000	7.79025000
C	-12.37678900	13.19075700	8.21527600
C	-13.42090200	12.70982000	7.40661600
H	-13.91045100	11.76662000	5.51158700
H	-11.57266400	11.62876900	4.75804200
C	-9.39635000	12.46335200	6.09037800
H	-12.63684700	13.62415300	9.18165300
N	-9.15582900	11.05456700	3.26090200
N	-8.07200600	11.17205000	3.34796000
H	-10.26816900	13.49178200	8.43255500
C	-8.09559900	12.85201300	6.65420800
H	-7.89535800	12.34008800	7.61301600
H	-7.27621200	12.59306000	5.96716400
H	-8.03787600	13.93989300	6.84027100
O	-14.67042700	12.82996600	7.90828600
C	-15.74519500	12.36523700	7.13745600
H	-16.65496000	12.55410800	7.71785700
H	-15.81426000	12.89974800	6.17523600
H	-15.66278700	11.28359800	6.93849600

Int6

E = -423.561770033 a.u.

0 1

C	1.93701300	-0.37059100	0.67421600
C	3.31637900	-0.51984900	0.90415500
C	4.15464400	0.56307700	0.73278400
C	3.67484800	1.82964900	0.31043000

C	2.28259100	1.94329800	0.08919500
C	1.41870600	0.87354900	0.26881900
H	3.68066600	-1.49668900	1.22213800
H	5.22761800	0.48111600	0.91380400
H	1.86046900	2.89919800	-0.22412100
H	0.35148900	1.00346400	0.09571600
O	1.18519300	-1.46102300	0.86937400
C	-0.20423300	-1.38458300	0.65938100
H	-0.43680400	-1.11546000	-0.38310700
H	-0.60601600	-2.38034100	0.87337100
H	-0.67113800	-0.65153700	1.33611000
C	4.66430500	2.87512900	0.16149600
C	4.20999300	4.15091800	-0.43277500
H	3.13148400	4.37465900	-0.53923300
H	4.72181200	5.00364900	0.03841800
H	4.64728000	4.11475300	-1.45093300

Int7

E = -1212.55423047 a.u.

0 1

C	-9.23275400	8.10407100	7.39441400
C	-9.17736600	9.16814100	6.50881800
C	-8.97684100	10.50156700	6.94713800
C	-8.83523400	10.71938000	8.34851900
C	-8.89338300	9.67573700	9.23998800
C	-9.09096300	8.35612500	8.76955000
H	-9.38233400	7.09318500	7.02008200
H	-9.27390300	9.00303300	5.43315500
C	-8.93617000	11.50276400	5.93298000
H	-8.79150800	9.82112000	10.31545600
H	-8.68333400	11.73066600	8.72729000
C	-8.64009100	12.90414400	6.27192800
H	-8.58748000	13.20279200	7.33117600
H	-7.66205100	13.09868500	5.79163500
H	-9.32520500	13.56810400	5.72115700
O	-9.13007100	7.41021500	9.70625400
C	-9.32254900	6.06577400	9.32389800
H	-9.31881000	5.47881300	10.24759200
H	-10.28753100	5.93490800	8.81069400
H	-8.50888000	5.72105900	8.66758600
C	-7.93407100	10.58466200	2.81806400
O	-9.11332700	10.40941800	3.48160200
H	-9.09607500	10.93497400	4.40464300
C	-7.81060400	12.01629500	2.28689300
F	-8.02038500	12.91489900	3.25190000
F	-6.60451400	12.25575900	1.76747500

F	-8.71313800	12.23150600	1.33684700
H	-7.88525200	9.93012000	1.92984100
C	-6.70445000	10.21024000	3.65891700
F	-6.93012000	9.07991100	4.32490900
F	-5.62817500	10.02291100	2.89761600
F	-6.39300100	11.14966600	4.56205300

TS3

E = -1212.55254562 a.u.

0 1

C	-9.22748800	8.13556600	7.35862800
C	-9.16964900	9.20628600	6.48430900
C	-8.96425600	10.53441200	6.94738900
C	-8.82014100	10.74103300	8.35181600
C	-8.88301400	9.68782500	9.22771500
C	-9.08429800	8.37277800	8.73757900
H	-9.37771800	7.12874400	6.97397900
H	-9.26236400	9.06554900	5.40318100
C	-8.91714900	11.54194500	5.95736100
H	-8.78111500	9.81714000	10.30506200
H	-8.66469400	11.74850600	8.73942100
C	-8.61016000	12.94619400	6.25613100
H	-8.53744900	13.24849800	7.31061600
H	-7.64487400	13.13399700	5.74928800
H	-9.31328100	13.59681700	5.71228500
O	-9.12398900	7.41895300	9.66083600
C	-9.31641000	6.07579100	9.26703300
H	-9.31162900	5.48261600	10.18649100
H	-10.28172900	5.95057400	8.75386200
H	-8.50229100	5.73877100	8.60791100
C	-7.97947400	10.54667600	2.88932900
O	-9.10694900	10.43804200	3.62446600
H	-9.05057100	11.07550000	4.68782600
C	-7.83245300	11.96463100	2.32395400
F	-7.94529300	12.88651100	3.29093600
F	-6.65717400	12.16686600	1.72150100
F	-8.78832600	12.20790100	1.43429100
H	-7.96753900	9.87759200	2.00262300
C	-6.72667300	10.15738300	3.68779500
F	-6.92373000	8.99291600	4.30878000
F	-5.64166800	10.02530700	2.92757100
F	-6.43552200	11.06032500	4.64160600

Int8

E = -1212.55652275 a.u.

0 1

C	-9.25226400	8.21036100	7.28073300
C	-9.20209900	9.30701100	6.44307500
C	-8.95704500	10.61295900	6.96512300
C	-8.75471100	10.77053000	8.37193500
C	-8.81248800	9.68686200	9.20330000
C	-9.05962100	8.39349300	8.66362600
H	-9.42671500	7.22024300	6.86428400
H	-9.30447400	9.23017300	5.34808300
C	-8.92414800	11.65342800	6.03512800
H	-8.67176600	9.76713100	10.28080700
H	-8.56551700	11.76025500	8.78932300
C	-8.56241300	13.04983600	6.29320800
H	-8.39172300	13.32421800	7.34036100
H	-7.64511500	13.22022100	5.70010600
H	-9.30899300	13.71183500	5.82725400
O	-9.08711100	7.41061700	9.54802900
C	-9.32292900	6.08261500	9.11745100
H	-9.30146900	5.46193500	10.01797000
H	-10.30687700	5.99805800	8.63334900
H	-8.53739100	5.75257900	8.42204500
C	-7.99894800	10.40643200	2.95821800
O	-9.11737600	10.28389100	3.66997500
H	-9.12738200	11.31996700	4.96687000
C	-7.88427500	11.81712000	2.35747200
F	-8.02649100	12.76475300	3.30529600
F	-6.71390000	12.05392000	1.75298500
F	-8.84326900	12.02108700	1.46086600
H	-7.89716300	9.72339100	2.07519100
C	-6.74716500	10.10495200	3.80317900
F	-6.88228800	8.92691700	4.41987200
F	-5.61004500	10.05840400	3.10980200
F	-6.56899900	11.02225200	4.78135400

***d*-77d**

E = -1212.65850712 a.u.

0 1

C	-0.53593300	-1.08177400	0.32837700
C	0.73909500	-0.89622700	-0.18686700
C	1.26803200	0.38896300	-0.35719900
C	0.48860000	1.48446100	0.01470300
C	-0.79148700	1.31622500	0.54168000
C	-1.31230700	0.02554300	0.69712500
H	-0.95976400	-2.07659600	0.46627200
H	1.33763200	-1.76774200	-0.46429600
H	0.89637600	2.49168600	-0.09188800
H	-1.36958600	2.19313900	0.82878700

O	-2.53739900	-0.24291600	1.19463100
C	2.63865800	0.59290200	-0.96504800
H	3.18175900	-0.36636700	-0.94000900
C	2.58122700	1.09965800	-2.39432300
H	3.59745800	1.23582800	-2.78731900
H	2.03834200	0.38420400	-3.02525900
H	2.05614100	2.06465500	-2.42585000
O	3.39083900	1.56938700	-0.23571300
C	3.73552600	1.22707900	1.06034100
H	3.05094400	0.48341100	1.50708800
C	3.63081600	2.49324600	1.90982200
C	5.14617700	0.63072200	1.09765500
F	2.35668100	2.86200600	2.01749400
F	4.30346000	3.50085300	1.37606100
F	4.10046400	2.27813900	3.13712600
F	5.40825400	0.09338300	2.28549400
F	5.24842700	-0.33499200	0.18283100
F	6.07770700	1.53765500	0.83606700
C	-3.35233100	0.83174800	1.58316300
H	-3.57307300	1.49874400	0.73347900
H	-4.28852400	0.39892500	1.95180200
H	-2.88547000	1.42164100	2.38914400

^{3}Int2'

E = -1813.47931231 a.u.

-1 3

C	-7.86225400	10.44604500	4.81757500
C	-9.23918200	10.31781900	4.67433800
C	-9.99244800	11.24512800	3.90588300
C	-9.27123000	12.29516800	3.27346800
C	-7.89984700	12.41600900	3.41526700
C	-7.17858800	11.49790300	4.19156900
H	-7.33001600	9.71105100	5.42142900
H	-9.76065200	9.48838500	5.15730200
C	-11.41207000	11.11965700	3.80237600
H	-7.35011900	13.23051300	2.94087100
N	-12.05305600	10.16777700	4.56307100
N	-12.13635400	8.92179800	4.09301800
S	-12.41497500	7.88081100	5.33555600
O	-11.30320400	7.84436500	6.29911400
O	-12.87177100	6.61788800	4.76574800
C	-13.79530600	8.58967900	6.25571100
C	-13.69163500	8.78504100	7.62681100
C	-14.95136900	8.95375100	5.56823300
C	-14.75351800	9.37121500	8.31691400
H	-12.76386400	8.49627900	8.12352500

C	-15.99915900	9.54727100	6.26474400
H	-15.00079300	8.79265700	4.48937700
C	-15.91191700	9.77415200	7.64618400
H	-14.67332300	9.53845300	9.39426900
H	-16.90248600	9.85216300	5.72928600
C	-17.02134300	10.48796700	8.37437000
H	-16.91841100	11.57956000	8.25985300
H	-18.00751700	10.20701400	7.97841600
H	-17.00307600	10.26427600	9.44977500
H	-9.80758700	13.03825900	2.68150200
C	-12.22442900	12.07856300	2.98254900
H	-11.88322900	12.10613200	1.93282200
H	-13.27703500	11.76713700	2.99422900
H	-12.15960300	13.11524500	3.36586700
O	-5.82926900	11.70030300	4.27824900
C	-5.09531700	10.82333100	5.07700000
H	-4.04961700	11.15387400	5.03983000
H	-5.43903000	10.83855800	6.12643600
H	-5.15450600	9.78328600	4.71027100
C	-11.71959900	14.01951200	6.43072300
N	-12.20906200	12.78002100	6.77827300
C	-11.32242600	11.78419800	7.38099600
C	-10.02329800	12.40783800	7.85963700
C	-9.50037400	13.35519400	6.78675300
N	-10.48022000	14.35753400	6.43161000
H	-11.86128300	11.33237500	8.23367000
H	-10.19770000	12.97952100	8.78705500
H	-8.58449300	13.86236200	7.13009400
H	-11.14662400	10.97036400	6.65643200
H	-9.29676100	11.61277700	8.08288800
H	-9.21770800	12.77045900	5.89459200
C	-12.68822800	15.12791400	6.03936500
H	-12.91868800	15.05473700	4.96281900
H	-12.09617300	16.04129800	6.16453200
C	-13.98399200	15.18242100	6.84991900
H	-14.33732600	16.22246200	6.92548800
H	-13.77018900	14.85461100	7.88046200
C	-15.08046300	14.30562900	6.24323400
H	-15.86515400	14.11541800	6.99475400
H	-15.56892400	14.84233000	5.41265000
C	-13.57706700	12.28137100	6.69052900
H	-14.03397400	12.25973100	7.70408000
H	-13.48397800	11.23534700	6.36103300
C	-14.52334200	12.98041000	5.72698800
H	-15.34988200	12.28250500	5.52167700
H	-14.00131300	13.12075900	4.77006000

[3]TS1'

E = -1813.46453313 a.u.

-1 3

C	-7.87835200	10.53156900	4.54915800
C	-9.24626900	10.30254500	4.43712500
C	-10.08703600	11.15328200	3.66899000
C	-9.44253800	12.24860500	3.01768000
C	-8.08047600	12.46395900	3.12203600
C	-7.27626600	11.61114700	3.89133500
H	-7.29213900	9.85413200	5.17056100
H	-9.67806400	9.47206700	4.99289500
C	-11.50312700	10.97939500	3.56255700
H	-7.60299600	13.30627000	2.61826400
N	-12.17419100	9.87221300	3.97057400
N	-11.82872800	8.67373700	4.14322300
S	-11.90994500	8.07138600	5.95813500
O	-10.89033800	8.59599900	6.89775100
O	-12.05704100	6.61803200	5.85108800
C	-13.47899000	8.73971400	6.56014100
C	-13.58247100	9.12835200	7.89145400
C	-14.56897100	8.84335700	5.69856500
C	-14.79386800	9.62658200	8.36757300
H	-12.70195300	9.04167500	8.53180300
C	-15.76438600	9.37377800	6.17883600
H	-14.46308900	8.53391800	4.65753800
C	-15.89396500	9.77453000	7.51511900
H	-14.88282500	9.92604100	9.41519900
H	-16.61755100	9.47912100	5.50372300
C	-17.17386200	10.39634300	8.01086600
H	-17.32147900	10.20604800	9.08293100
H	-17.15427000	11.48901100	7.86821200
H	-18.04533900	10.00774800	7.46605200
H	-10.03345800	12.93970400	2.41547200
C	-12.36032000	12.06710000	2.98144400
H	-12.20731700	12.18962600	1.89293400
H	-13.41835200	11.81877200	3.14266500
H	-12.14881200	13.04458400	3.44995300
O	-5.93884300	11.90658600	3.94329600
C	-5.13926100	11.10701700	4.75727400
H	-4.11729200	11.50258000	4.69469100
H	-5.46934800	11.13293600	5.81136900
H	-5.13437300	10.05289300	4.42593600
C	-11.70847000	14.17783600	6.46941300
N	-12.19343600	12.88801700	6.52677000
C	-11.33026400	11.79455700	6.97482400

C	-9.98544100	12.29118300	7.47018900
C	-9.50939700	13.40735100	6.54840000
N	-10.46573500	14.49458300	6.50940800
H	-11.85624400	11.24871000	7.77740200
H	-10.07617200	12.69117000	8.49415200
H	-8.53458600	13.80057200	6.87548400
H	-11.19745900	11.07244600	6.15522600
H	-9.28413300	11.44464600	7.49067700
H	-9.36149800	12.99311300	5.53474600
C	-12.68204700	15.34696100	6.39725100
H	-12.91945300	15.56737700	5.34263000
H	-12.09208800	16.19647200	6.75984800
C	-13.96995100	15.17104200	7.20235300
H	-14.33512000	16.14993700	7.54947300
H	-13.74092300	14.58793700	8.10918200
C	-15.06514500	14.46818300	6.39752400
H	-15.83564000	14.07379400	7.08088300
H	-15.57217500	15.19719800	5.74339000
C	-13.54897500	12.41149600	6.28576300
H	-14.01529100	12.11189000	7.24772800
H	-13.44192400	11.47890000	5.70399000
C	-14.50270900	13.33446100	5.54167500
H	-15.32842700	12.71202500	5.16186600
H	-13.99276700	13.73600800	4.65407100

^{3}Int3'

E = -1813.52843995 a.u.

-1 3

C	-9.80051600	10.83206500	3.96614300
C	-10.61198200	11.85928200	3.50006300
C	-10.08653800	13.09791700	3.07688300
C	-8.68084500	13.24925100	3.10567500
C	-7.85614700	12.22124200	3.52359600
C	-8.40761200	11.00801900	3.97577600
H	-10.27155700	9.91867800	4.35090900
H	-11.68937800	11.70062300	3.50522700
C	-10.92907900	14.21913600	2.70099200
H	-6.77133200	12.33163700	3.54048300
N	-12.21388700	14.08367000	2.24804600
N	-12.84927500	13.08039400	2.02725800
S	-11.14959900	7.95179900	6.68384700
O	-11.34656400	8.48827000	5.26724000
O	-10.19951400	8.79601600	7.51775500
C	-12.77015000	8.41438000	7.43102600
C	-12.82833500	8.84911700	8.74919500
C	-13.93076400	8.35014700	6.66155700

C	-14.05441900	9.23306100	9.29871500
H	-11.89279700	8.91635900	9.31009200
C	-15.14884500	8.73768800	7.21345500
H	-13.84331300	8.04526200	5.61550000
C	-15.22859500	9.18706200	8.54059400
H	-14.10057200	9.59120000	10.33109900
H	-16.05759100	8.70925400	6.60439300
C	-16.54987800	9.63164200	9.11578600
H	-16.46541500	9.84041500	10.19097500
H	-16.90706800	10.54901500	8.62237600
H	-17.32527400	8.86333300	8.97677300
H	-8.22993200	14.19162300	2.79261500
C	-10.45495500	15.62818900	2.85193800
H	-9.52665800	15.82108300	2.29246700
H	-11.23236500	16.31173600	2.49055500
H	-10.27016700	15.82056300	3.92292800
O	-7.54186200	10.07729100	4.40003600
C	-8.07097000	8.90086000	4.99221300
H	-7.20535700	8.27593400	5.24127900
H	-8.64920600	9.12175500	5.90424200
H	-8.73256600	8.36237000	4.29598700
C	-11.95689500	13.78638600	6.02630400
N	-11.98714600	12.46885200	6.40224600
C	-10.88061100	11.87702600	7.15229200
C	-9.91487000	12.93809900	7.63857700
C	-9.67383100	13.91432900	6.49279100
N	-10.90897600	14.53899400	6.06745700
H	-11.30242000	11.29906400	7.99187000
H	-10.34366800	13.49007400	8.49157300
H	-8.95835400	14.70042400	6.78277200
H	-10.37276300	11.11974100	6.53277600
H	-8.98536800	12.45893900	7.97751300
H	-9.21596500	13.35869500	5.65309100
C	-13.24447800	14.47080300	5.58797500
H	-13.37774000	14.36167900	4.49925100
H	-13.04929100	15.53500800	5.76682800
C	-14.50115300	14.00203300	6.32483100
H	-15.22915900	14.82606200	6.38189600
H	-14.22946700	13.75611200	7.36429200
C	-15.14816800	12.78561400	5.65696200
H	-15.81447000	12.27677100	6.37399600
H	-15.77962700	13.11547300	4.81544200
C	-13.01728600	11.46424100	6.14591100
H	-13.48947500	11.17769600	7.10788700
H	-12.48104800	10.56158400	5.80035800
C	-14.10389700	11.79975100	5.13864400

H	-14.59217600	10.85021200	4.87095200
H	-13.65418500	12.17921800	4.20856100

Int7'

E = -848.492809413 a.u.

0 1

C	-8.38167800	8.83946900	8.46700600
C	-8.70610900	8.97209200	7.12497000
C	-9.39159500	10.10491100	6.62314700
C	-9.74922100	11.11930000	7.55549800
C	-9.44183000	11.00141700	8.89125200
C	-8.75235000	9.85970500	9.35841400
H	-7.85050100	7.95475100	8.81317400
H	-8.42817500	8.19847500	6.40374200
C	-9.65583200	10.10799800	5.21374700
H	-9.71182700	11.76495500	9.62103600
H	-10.28311400	12.00625600	7.21191300
C	-10.27130400	11.28938500	4.58748700
H	-10.66140600	12.10675200	5.21855200
H	-9.47808600	11.69386600	3.92383600
H	-11.03891100	10.96602900	3.86552400
O	-8.49500800	9.83449200	10.66782400
C	-7.81306400	8.72836000	11.21399600
H	-7.71575200	8.92366100	12.28661900
H	-8.38098500	7.79741900	11.06215200
H	-6.81130600	8.62081100	10.76993000
C	-7.29904100	8.59726600	3.28768200
O	-8.37717500	7.93874300	3.90479900
H	-8.94441400	8.65057200	4.33402300
C	-6.64237100	7.58178800	2.34935400
C	-6.28633400	9.04683600	4.34840200
H	-6.71605600	9.82803000	4.99410200
H	-6.03619100	8.17751900	4.97322800
H	-5.35513100	9.42894200	3.90582700
H	-5.78265400	8.02233200	1.82467200
H	-6.30715700	6.71318800	2.93465300
H	-7.37382200	7.23685000	1.60577200
C	-7.81876300	9.78033300	2.45779600
C	-7.13942300	10.99664100	2.34383100
C	-9.03481000	9.63222700	1.77538600
C	-7.66412600	12.04186400	1.57813300
H	-6.19294900	11.14715600	2.86432900
C	-9.55763900	10.66901200	1.00842500
H	-9.57587800	8.69027900	1.88119500
C	-8.87518300	11.88393300	0.90936400
H	-7.11996000	12.98554500	1.50810500

H	-10.50803600	10.53340300	0.48854000
H	-9.28589400	12.70048400	0.31337700

TS3'

E = -848.473244365 a.u.

0 1

C	-8.87885100	8.32360100	8.15676800
C	-8.99999700	9.17449700	7.07459500
C	-9.22855900	10.56781800	7.26170300
C	-9.32186100	11.07137000	8.59273000
C	-9.22892000	10.22531800	9.66802200
C	-9.00382900	8.84131500	9.46008300
H	-8.68339000	7.26585100	7.99061600
H	-8.85360200	8.83879000	6.04001700
C	-9.35594400	11.35986600	6.10656500
H	-9.31618700	10.57745500	10.69553600
H	-9.49297400	12.13455600	8.76512100
C	-9.39086100	12.83223500	6.09170200
H	-9.28023100	13.33010800	7.06209200
H	-8.58625900	13.16430800	5.41478700
H	-10.32200900	13.16175400	5.60072300
O	-8.91916000	8.10410900	10.56123200
C	-8.69281000	6.71329500	10.45369200
H	-8.66694400	6.32677900	11.47702000
H	-9.50571300	6.22438800	9.89613300
H	-7.73166400	6.50835500	9.95885500
C	-7.32870100	10.10592700	3.44715400
O	-8.27519000	9.83013900	4.39020700
H	-9.27297400	10.80024100	5.11938000
C	-6.04378400	9.30713100	3.75283000
C	-6.99395900	11.61606100	3.45568700
H	-7.90336000	12.19162600	3.21938600
H	-6.64331200	11.88963500	4.46462700
H	-6.21523000	11.89436900	2.72981600
H	-5.23138400	9.49803600	3.03472100
H	-5.69961900	9.57305700	4.76409600
H	-6.28226300	8.23390900	3.73882600
C	-7.80778000	9.72974000	2.02762900
C	-7.02343500	9.94893800	0.88698800
C	-9.06177900	9.13756100	1.86415700
C	-7.48276300	9.59277100	-0.37980500
H	-6.03468200	10.40453800	0.98552700
C	-9.52618300	8.77889800	0.59791300
H	-9.65272600	8.96811600	2.76431500
C	-8.73992200	9.00577100	-0.53024400
H	-6.85588900	9.77382000	-1.25535600

H	-10.50991600	8.31648600	0.49074700
H	-9.10134400	8.72610800	-1.52147900

P1

E = -848.603438840 a.u.

0 1

C	-8.77808400	7.50316700	6.49438900
C	-9.28055800	8.75451700	6.12526900
C	-8.76718300	9.93006500	6.66083000
C	-7.72857600	9.83883500	7.59814000
C	-7.21500300	8.60852300	7.97463400
C	-7.73400000	7.42827500	7.41917900
H	-9.19412300	6.60526400	6.03995200
H	-10.07148000	8.80582900	5.37231400
C	-9.27143500	11.29053600	6.21901100
H	-6.39998900	8.52123900	8.69394900
H	-7.29873100	10.75713100	8.00523600
C	-10.07687700	11.98675300	7.30725900
H	-9.43456100	12.17895300	8.17870300
H	-10.45647700	12.94967500	6.93792400
H	-10.92007400	11.35978200	7.62725400
O	-7.16182300	6.27248300	7.83091100
C	-7.62618800	5.07117600	7.27939200
H	-7.03345900	4.26648800	7.72847600
H	-8.69202900	4.90414000	7.50888900
H	-7.49227800	5.05060600	6.18443200
C	-7.83170100	12.24753700	4.50386200
O	-8.19545700	12.15133200	5.88212600
C	-8.92024700	13.00630400	3.74118500
C	-6.55616000	13.08779700	4.49945700
H	-9.06098900	13.98587300	4.21776700
H	-8.62742800	13.16400800	2.69378300
H	-9.88483000	12.48067300	3.74808700
H	-6.76595900	14.06755700	4.95039000
H	-6.19313600	13.22986400	3.47245300
H	-5.76883700	12.60182900	5.08910100
C	-7.55730000	10.85138800	3.93108500
C	-8.30337600	10.29738300	2.88921000
C	-6.55969300	10.06409500	4.52454400
C	-8.06800200	8.98891100	2.45607200
H	-9.08711900	10.87914800	2.40294700
C	-6.32353300	8.76186500	4.10041500
H	-5.98713200	10.46687500	5.36154100
C	-7.08215400	8.21698700	3.06155500
H	-8.66412000	8.57537100	1.64076900
H	-5.55397100	8.16381100	4.59156200

H	-6.90283100	7.19367100	2.72756500
H	-9.92155500	11.13144300	5.34101000

9 Spectra of hindered ethers and polyfluorinated ethers

1d

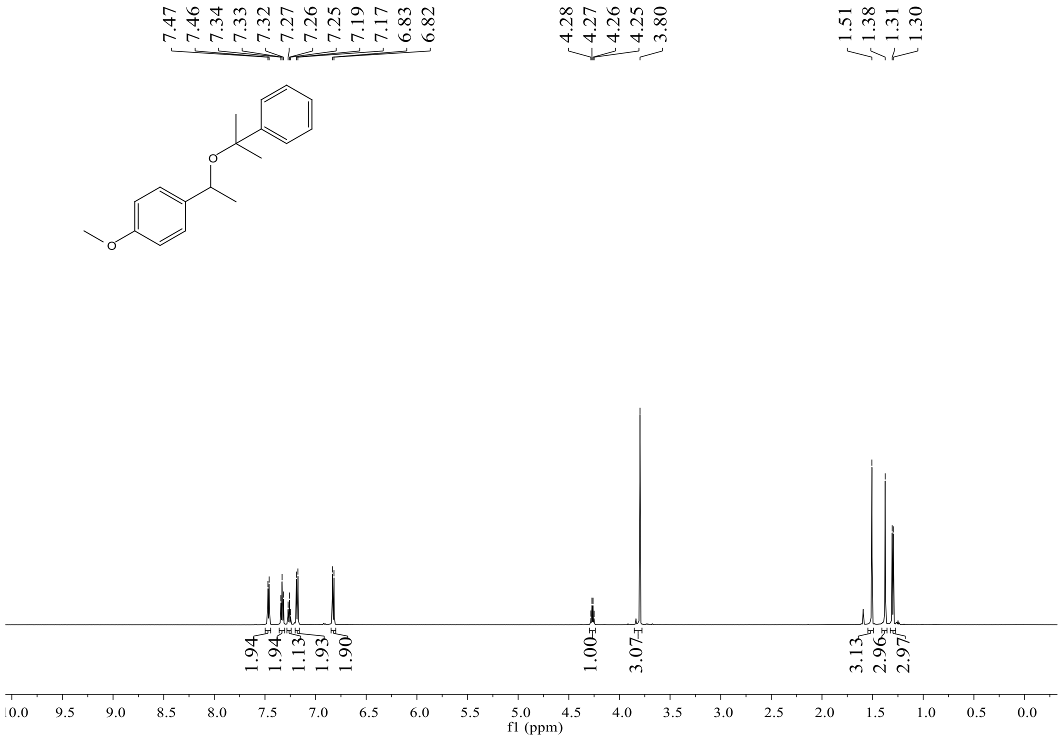

^{1}H NMR spectrum in $CDCl_3$.

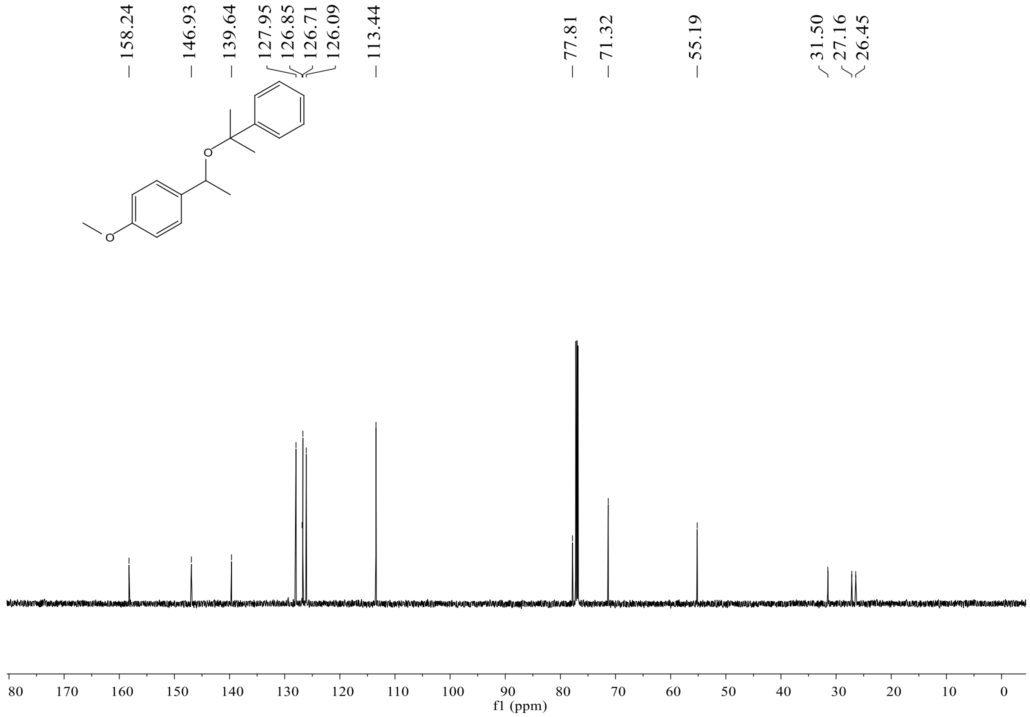

^{13}C NMR spectrum in $CDCl_3$.

2d

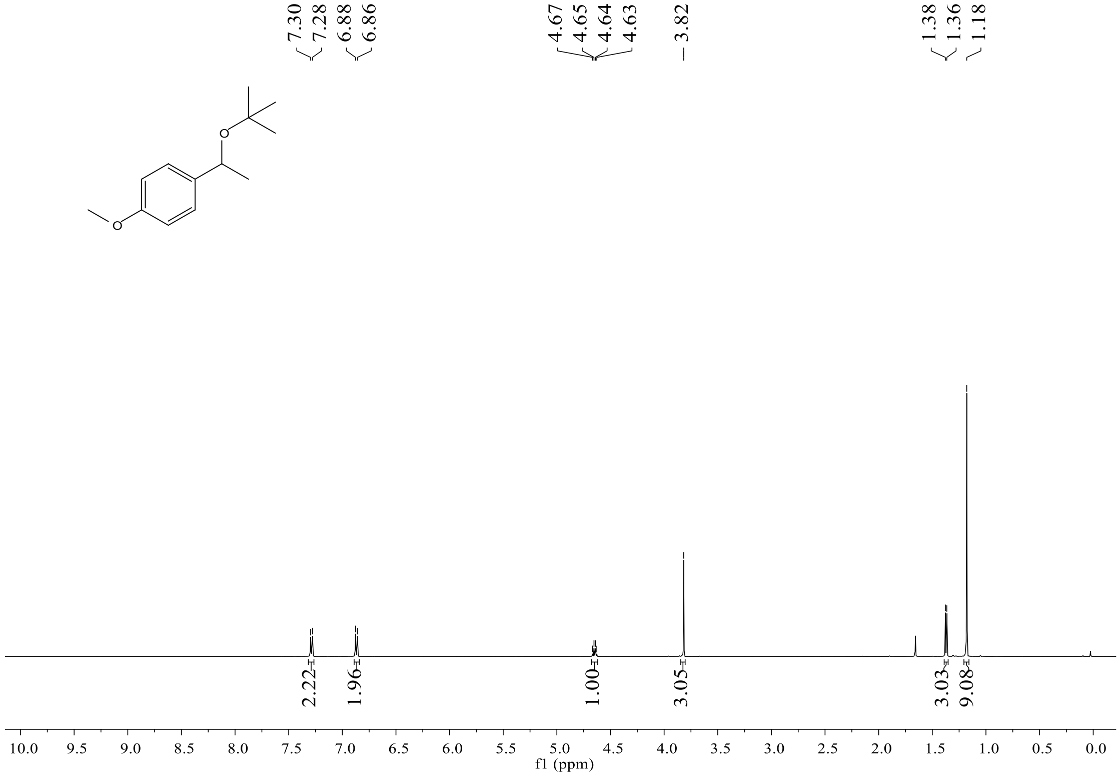

^{1}H NMR spectrum in $CDCl_3$.

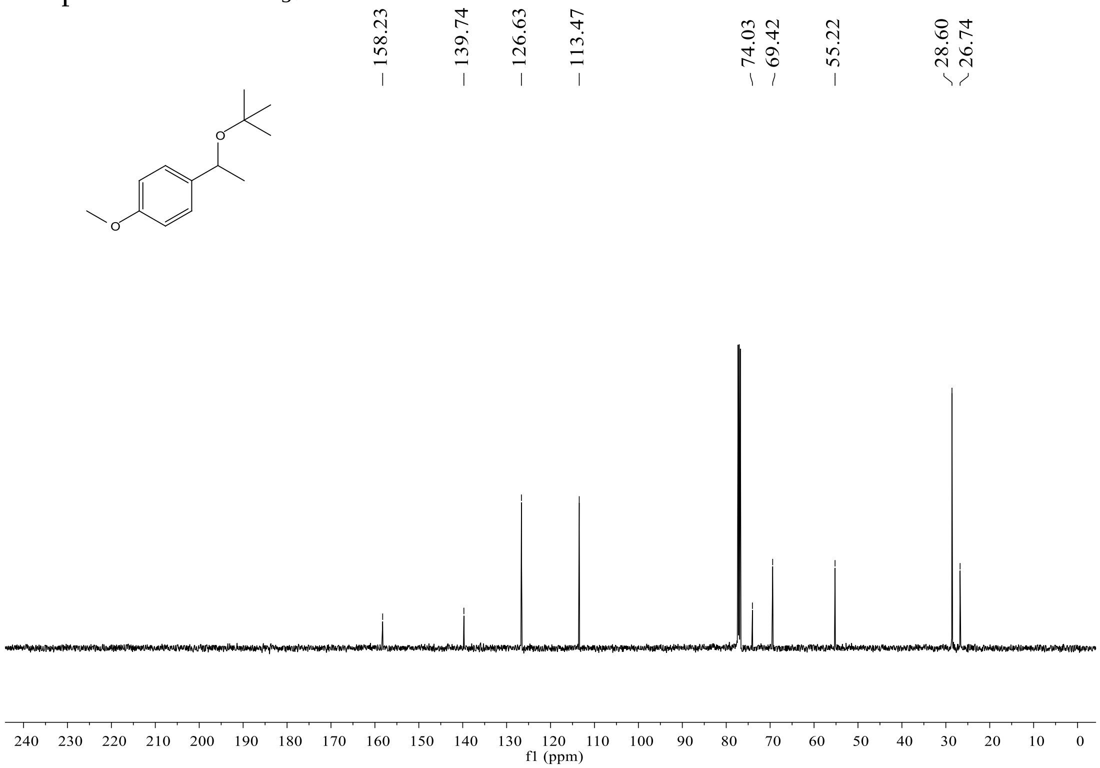

^{13}C NMR spectrum in $CDCl_3$.

3d

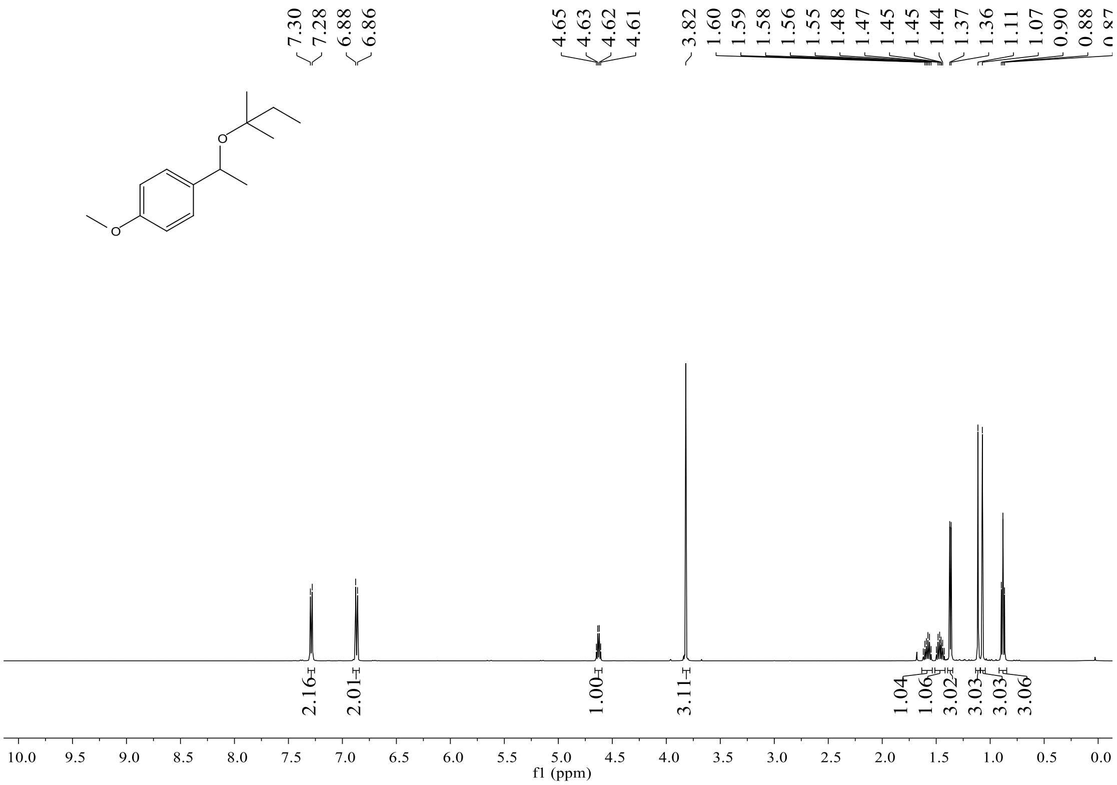

^{1}H NMR spectrum in $CDCl_3$.

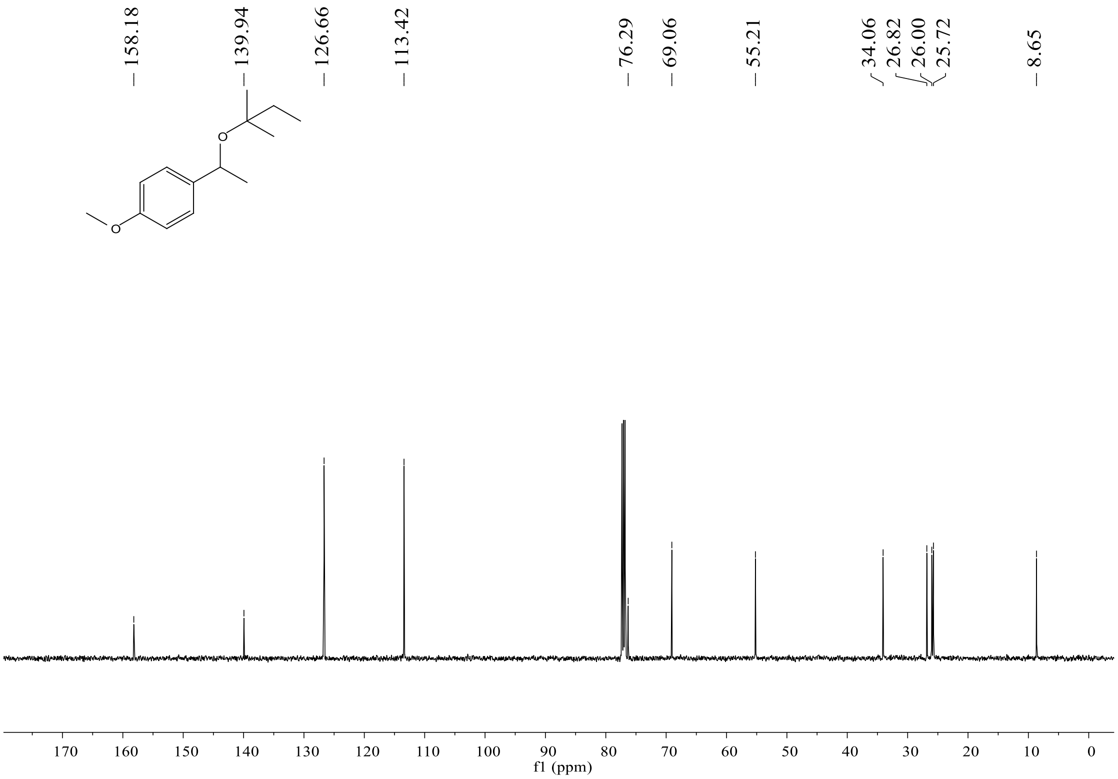

^{13}C NMR spectrum in $CDCl_3$.

4d

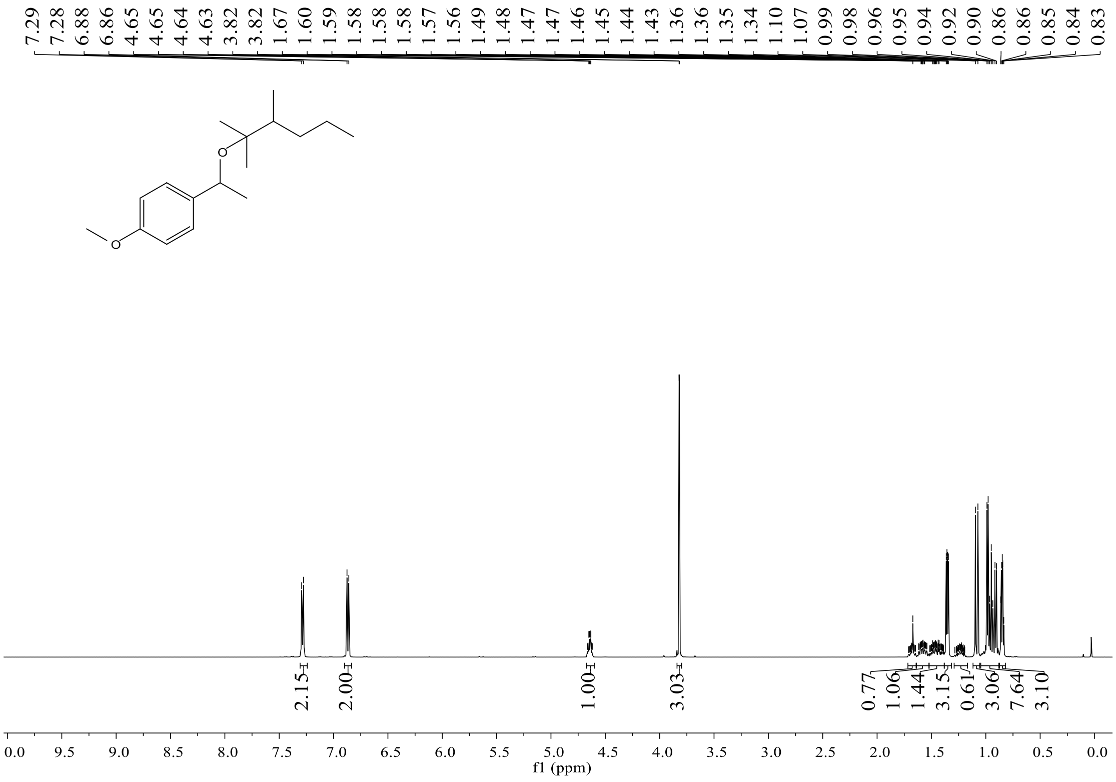

^{1}H NMR spectrum in $CDCl_3$.

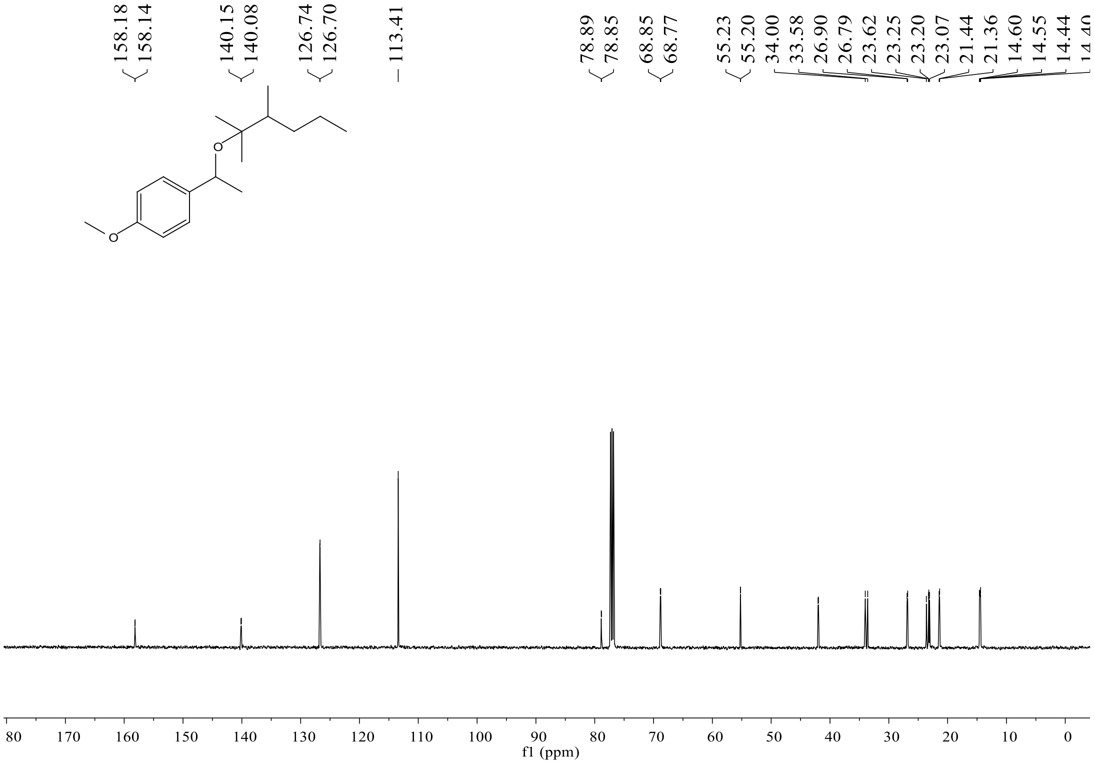

^{13}C NMR spectrum in $CDCl_3$.

5d

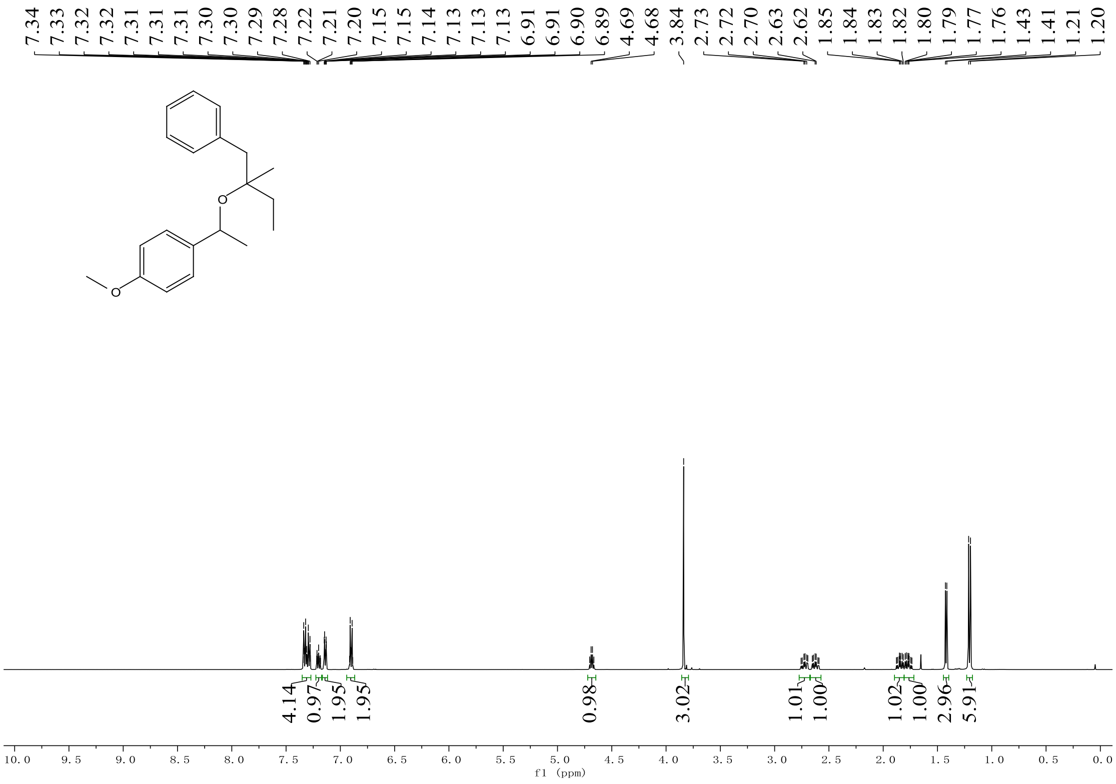

^{1}H NMR spectrum in $CDCl_3$.

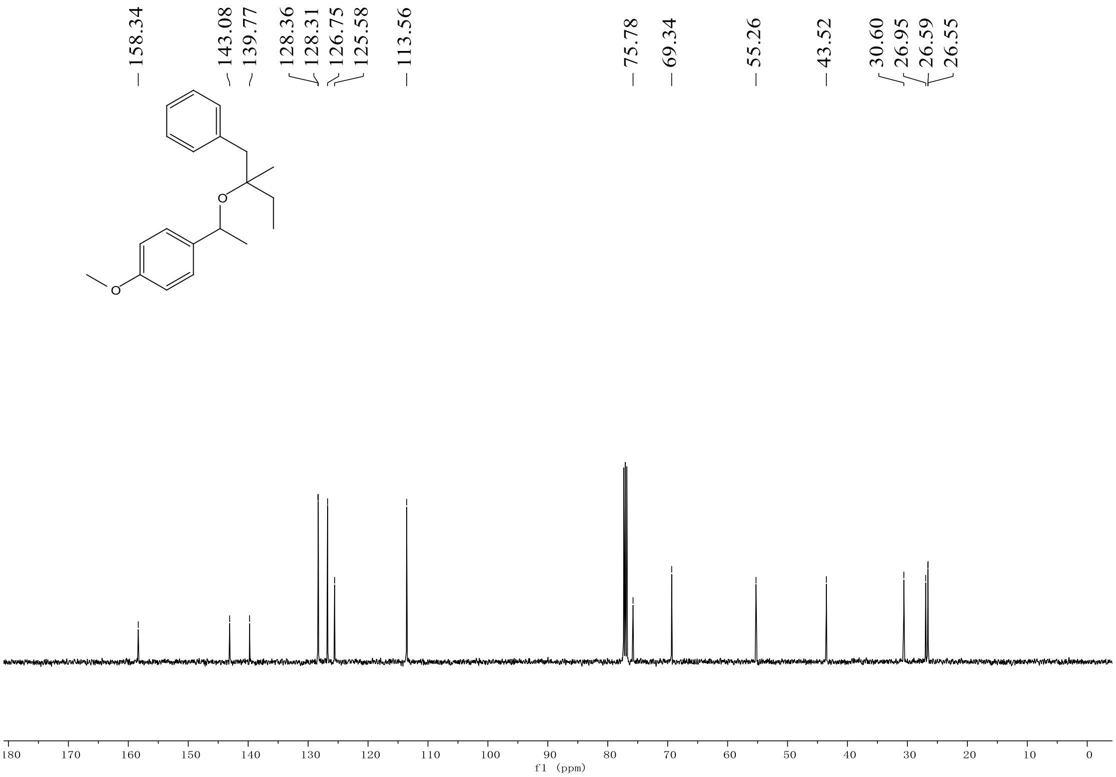

^{13}C NMR spectrum in $CDCl_3$.

6d

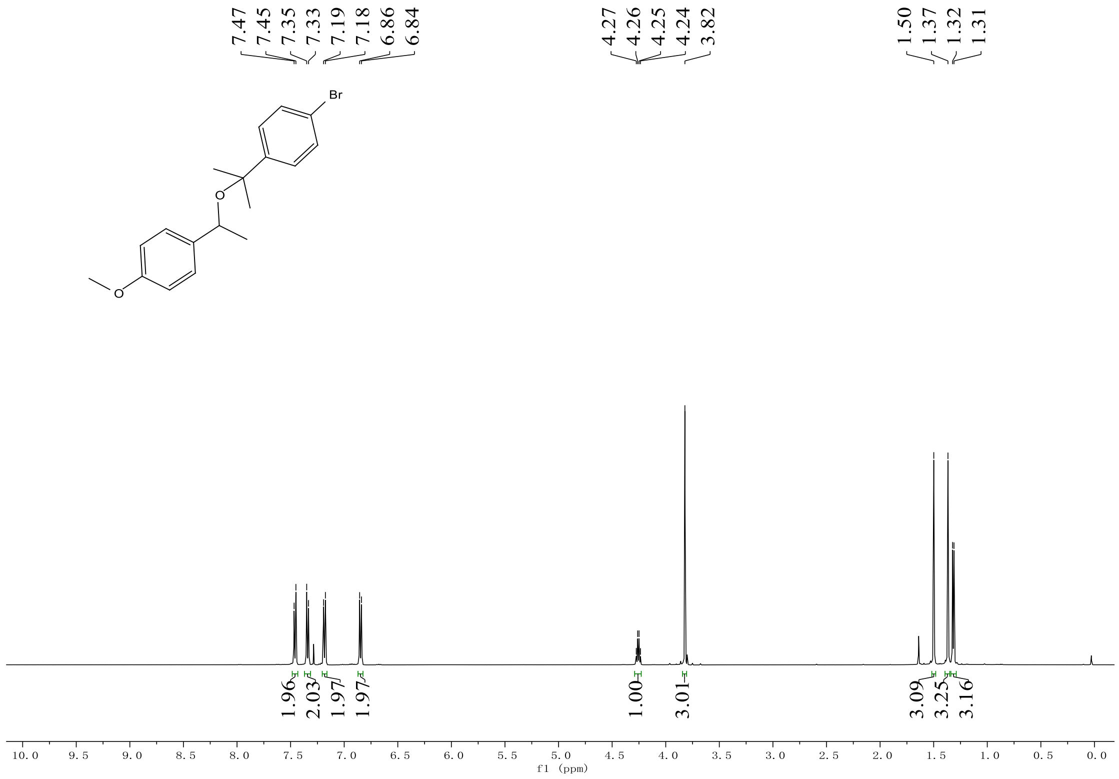

^{1}H NMR spectrum in $CDCl_3$.

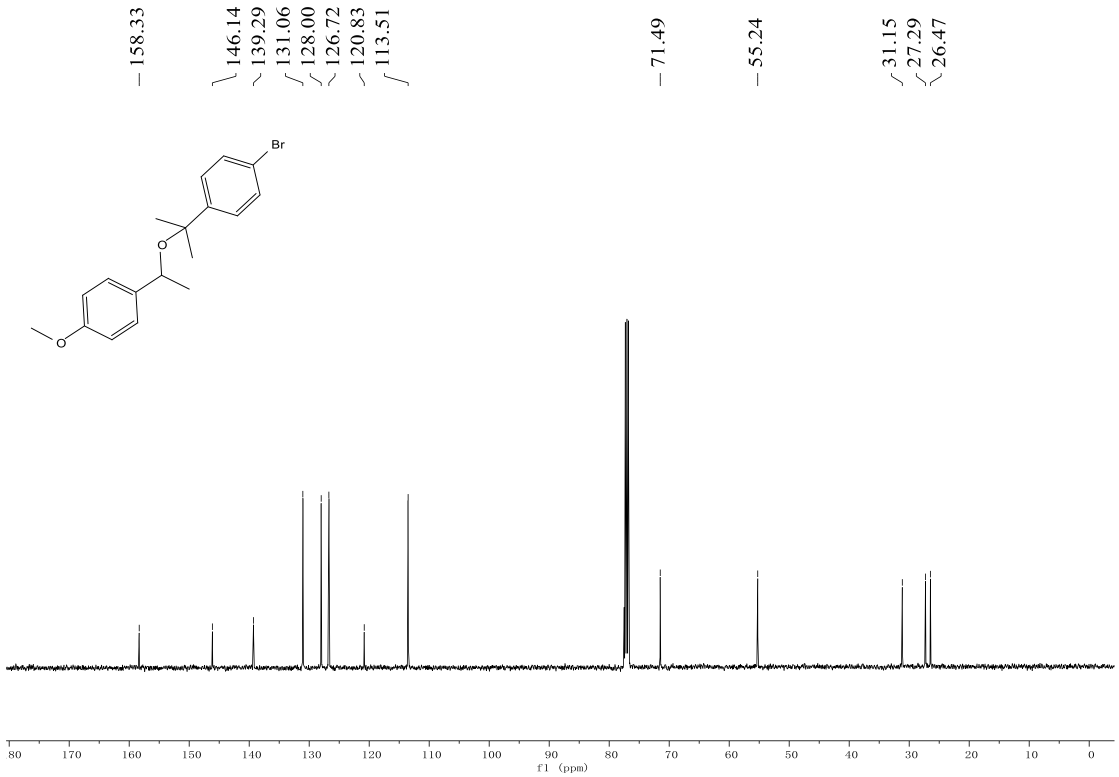

^{13}C NMR spectrum in $CDCl_3$.

7d

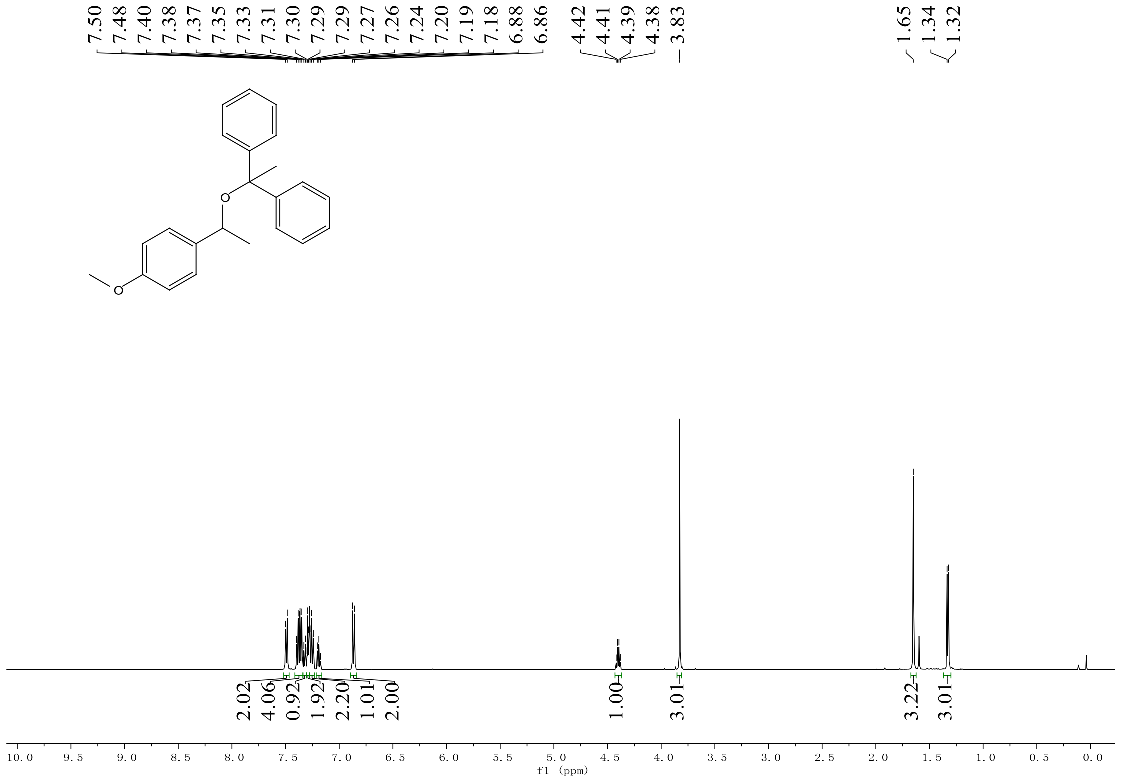

^{1}H NMR spectrum in $CDCl_3$.

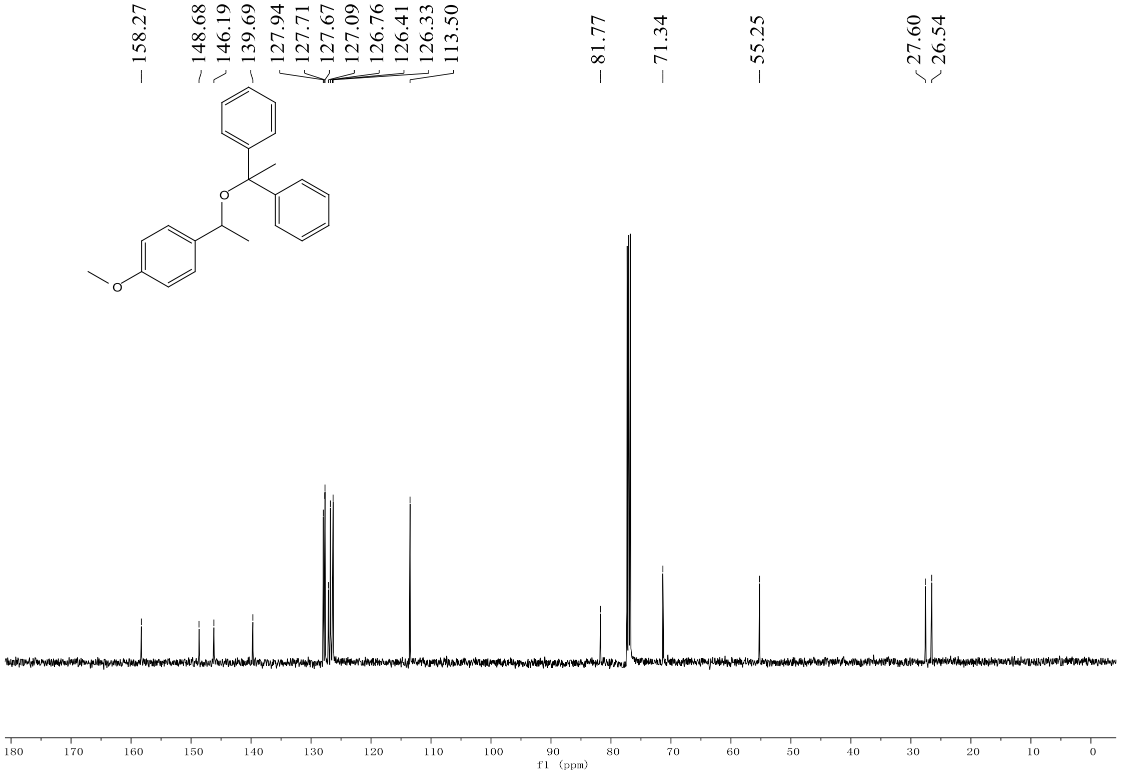

^{13}C NMR spectrum in $CDCl_3$.

8d

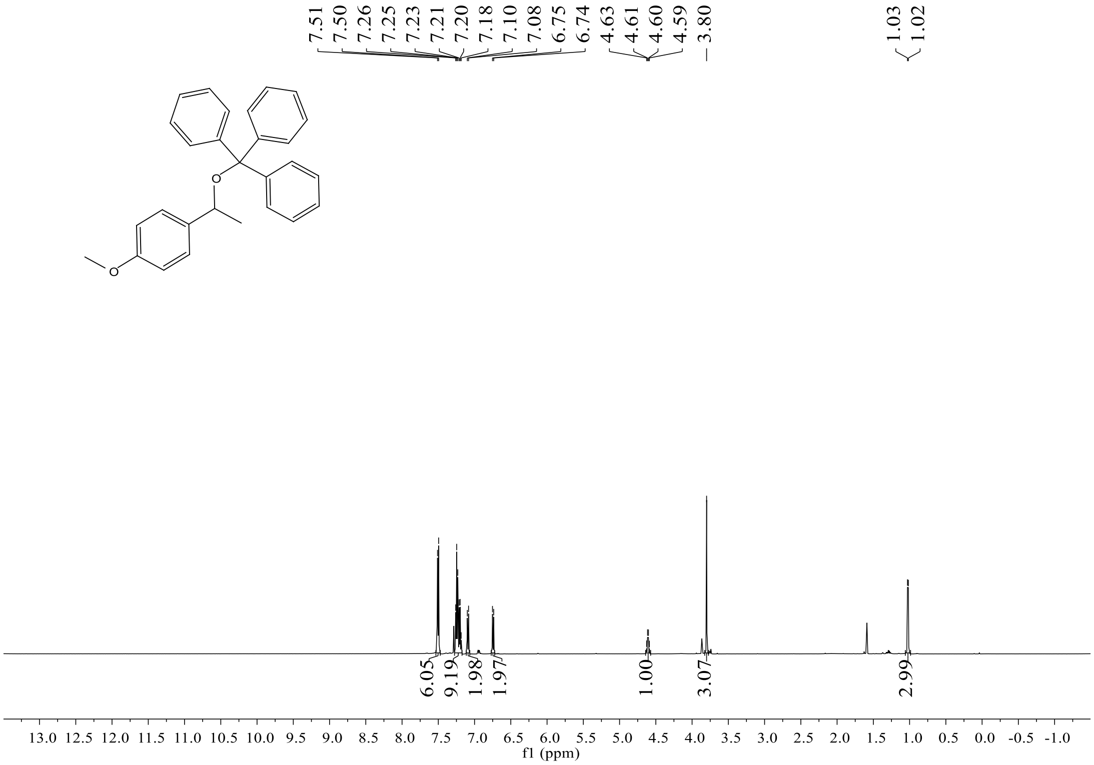

^{1}H NMR spectrum in $CDCl_3$.

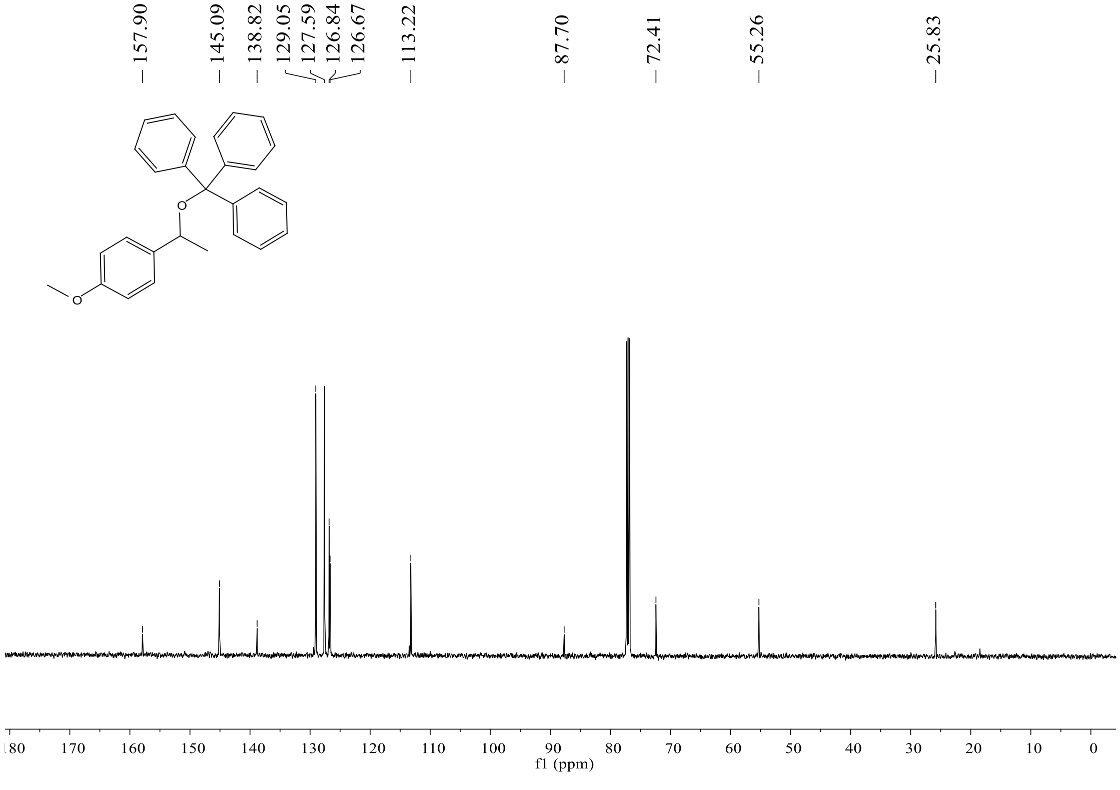

^{13}C NMR spectrum in $CDCl_3$.

9d

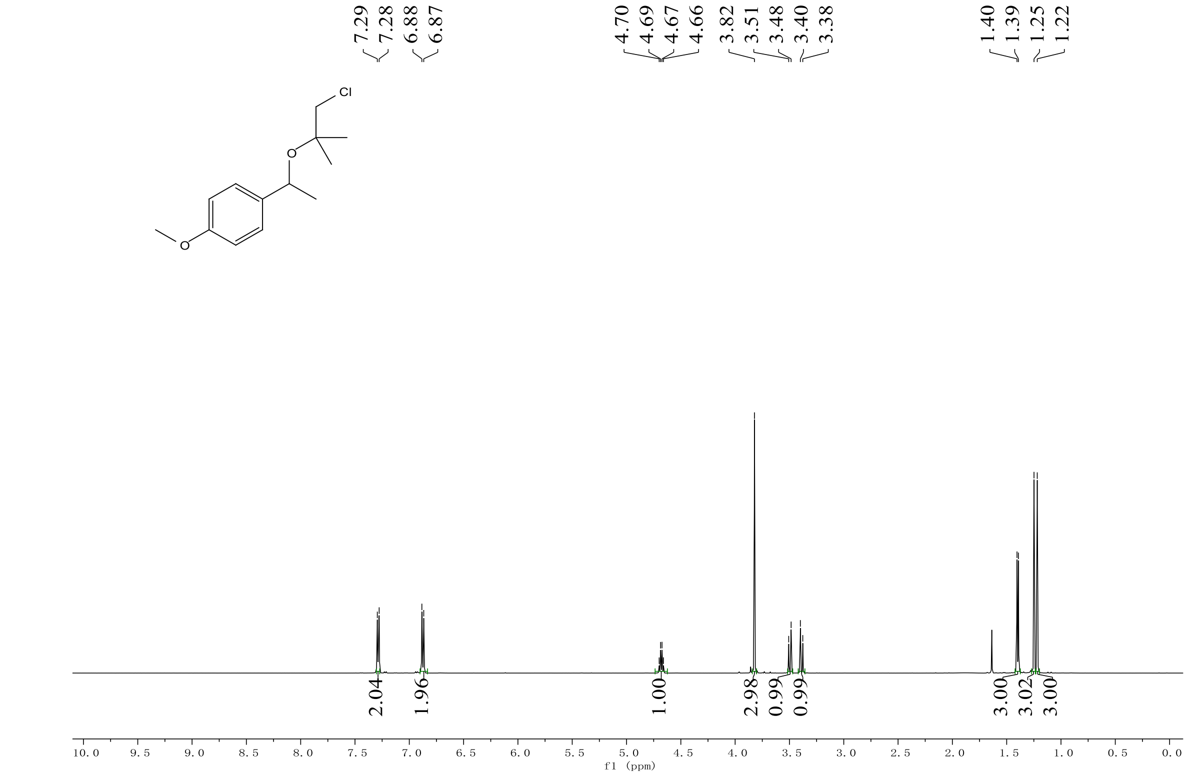

^{1}H NMR spectrum in $CDCl_3$.

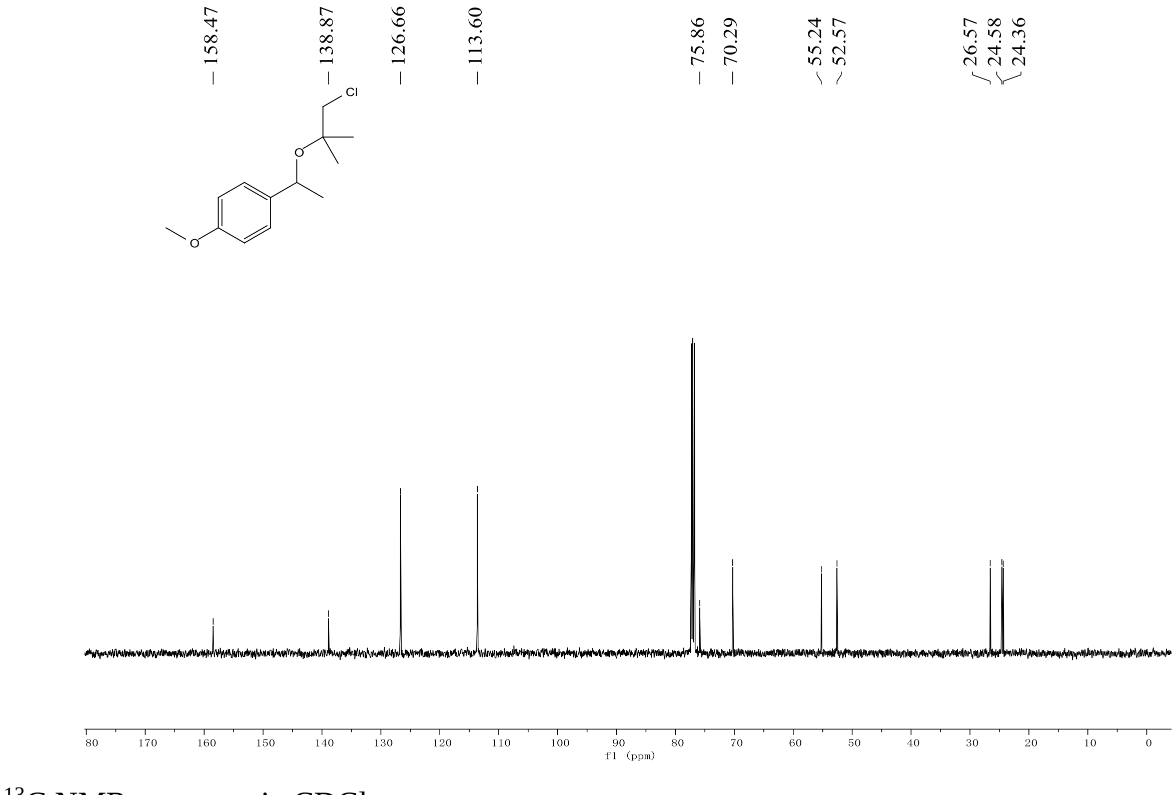

^{13}C NMR spectrum in $CDCl_3$.

10d

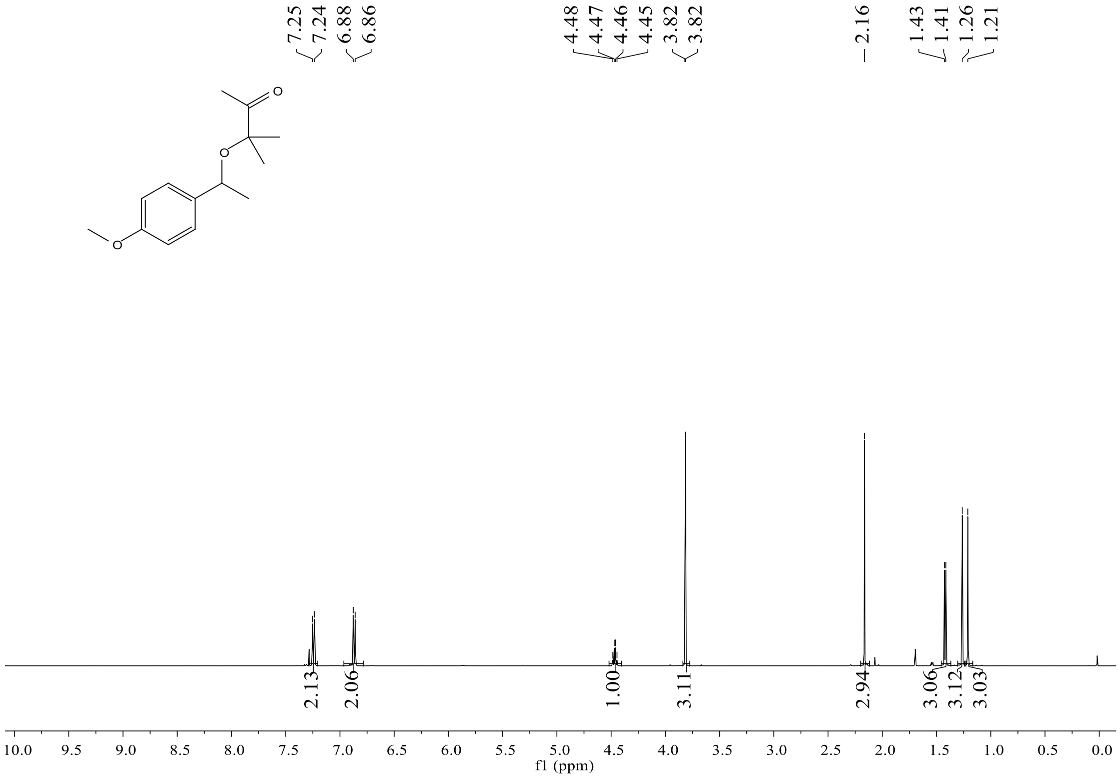

^{1}H NMR spectrum in $CDCl_3$.

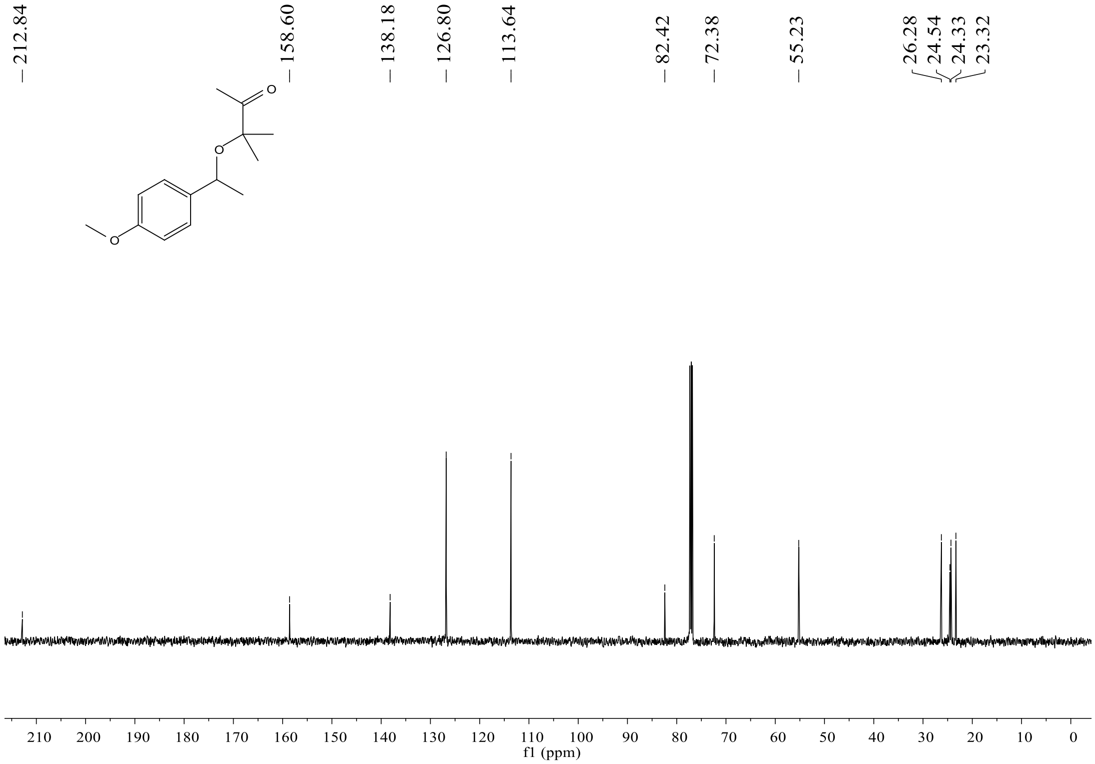

^{13}C NMR spectrum in $CDCl_3$.

11d

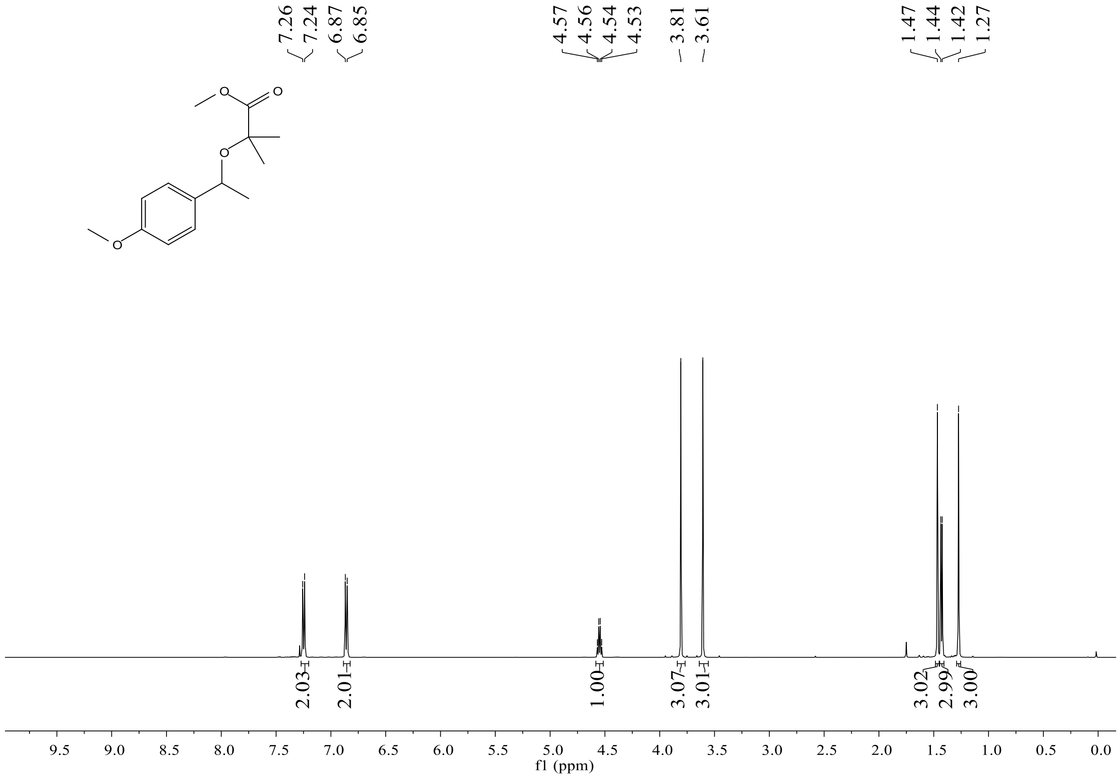

^{1}H NMR spectrum in $CDCl_3$.

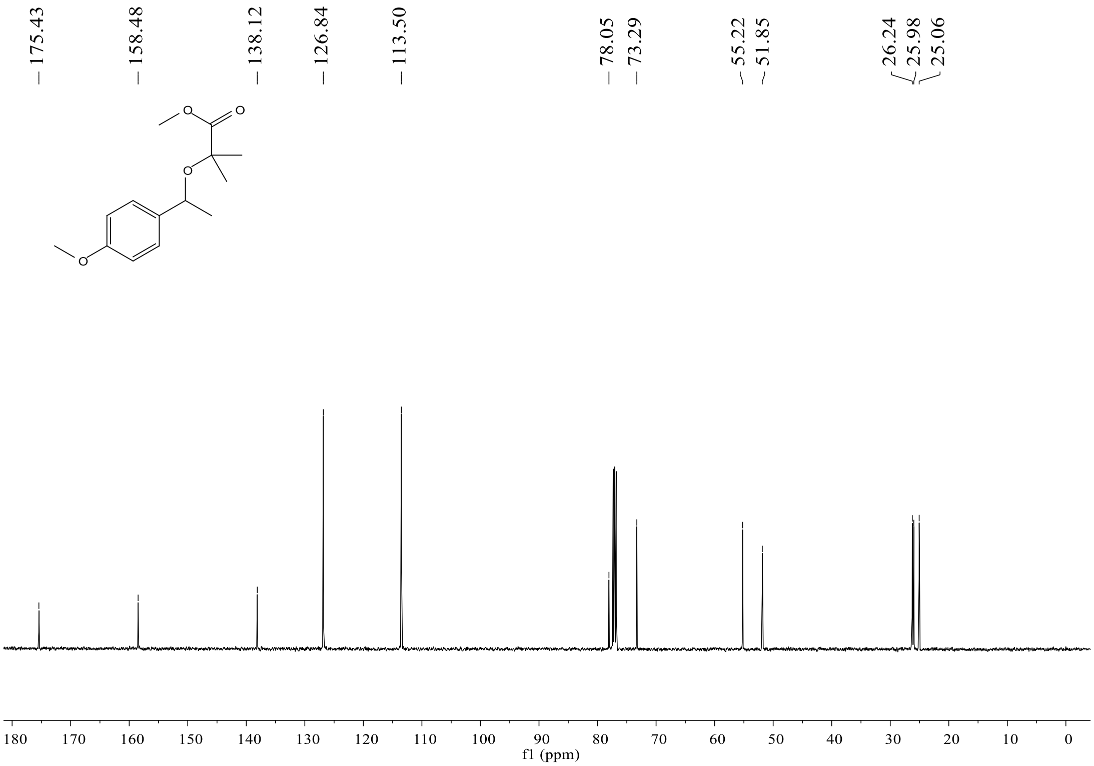

^{13}C NMR spectrum in $CDCl_3$.

12d

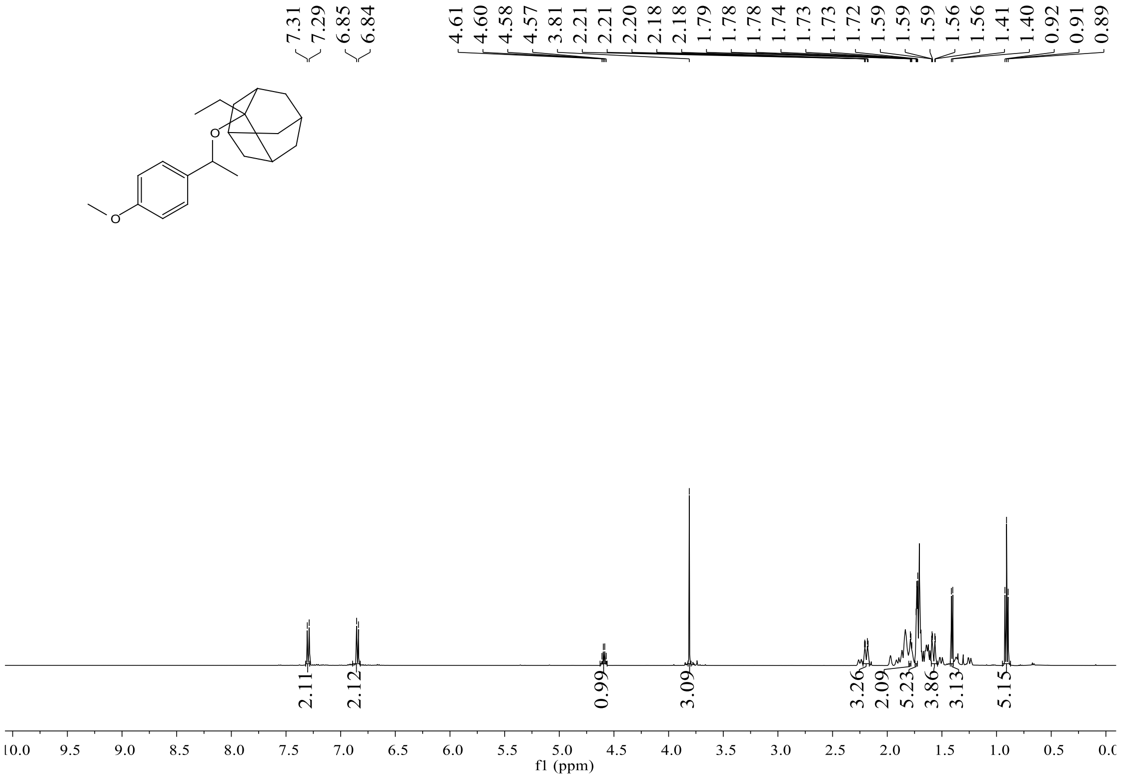

^{1}H NMR spectrum in $CDCl_3$.

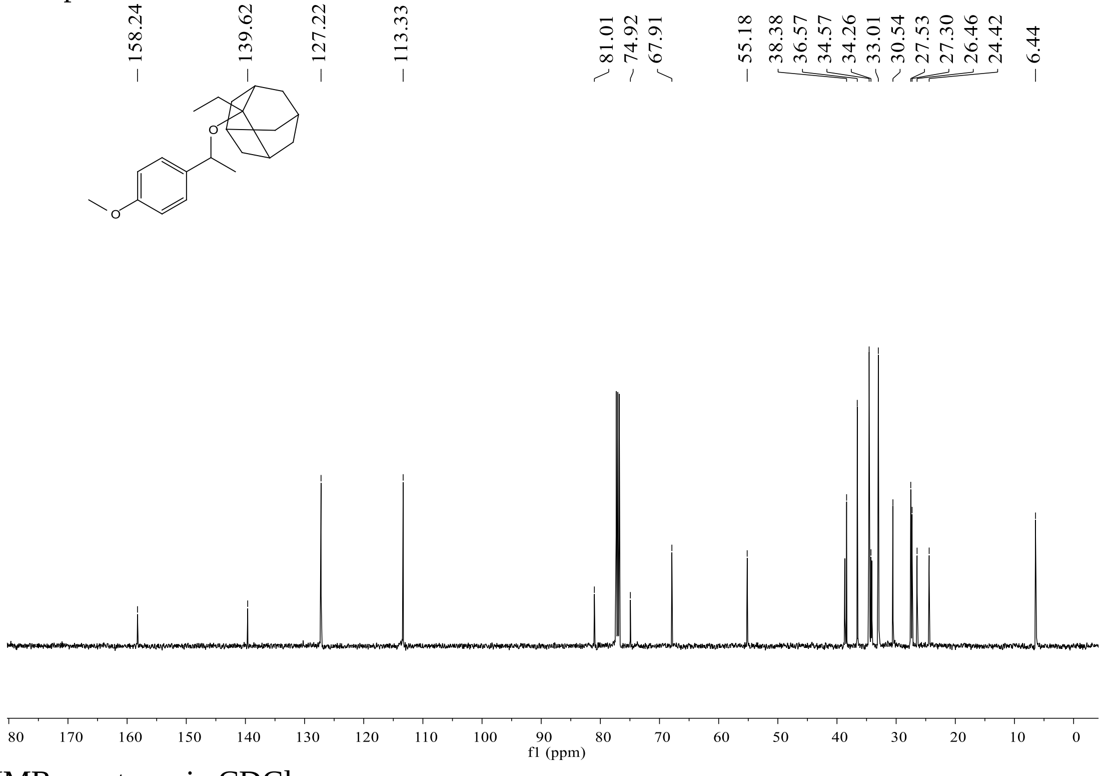

^{13}C NMR spectrum in $CDCl_3$.

13d

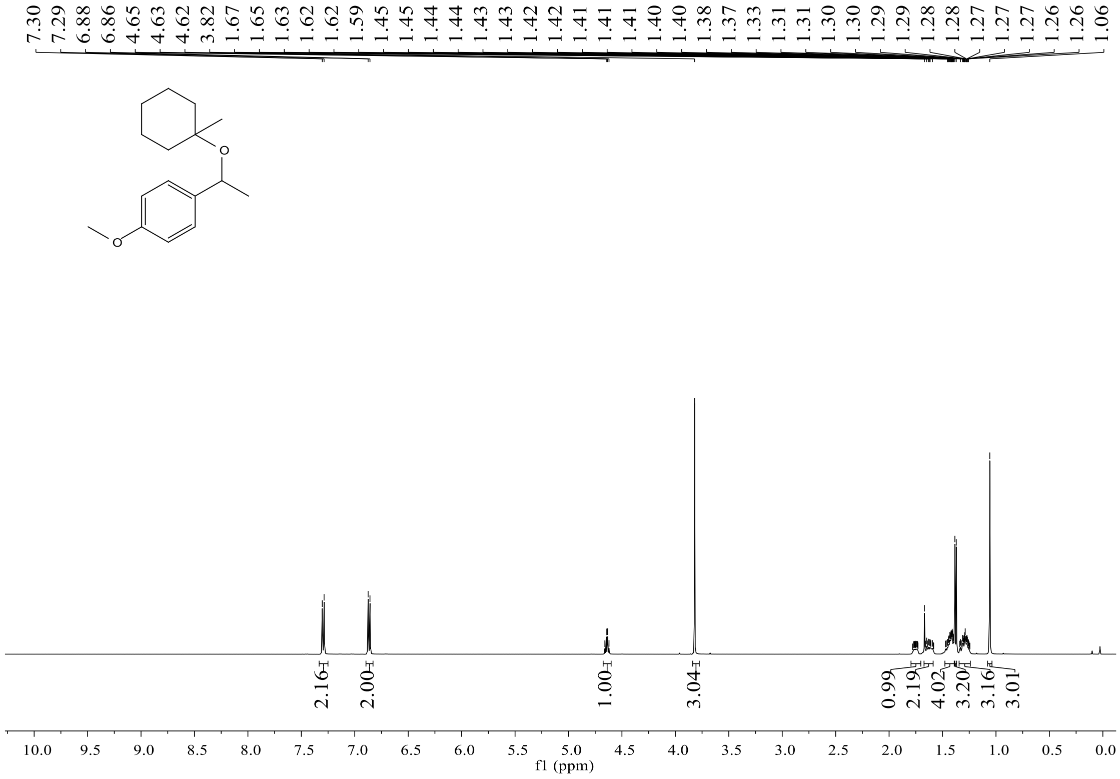

^{1}H NMR spectrum in $CDCl_3$.

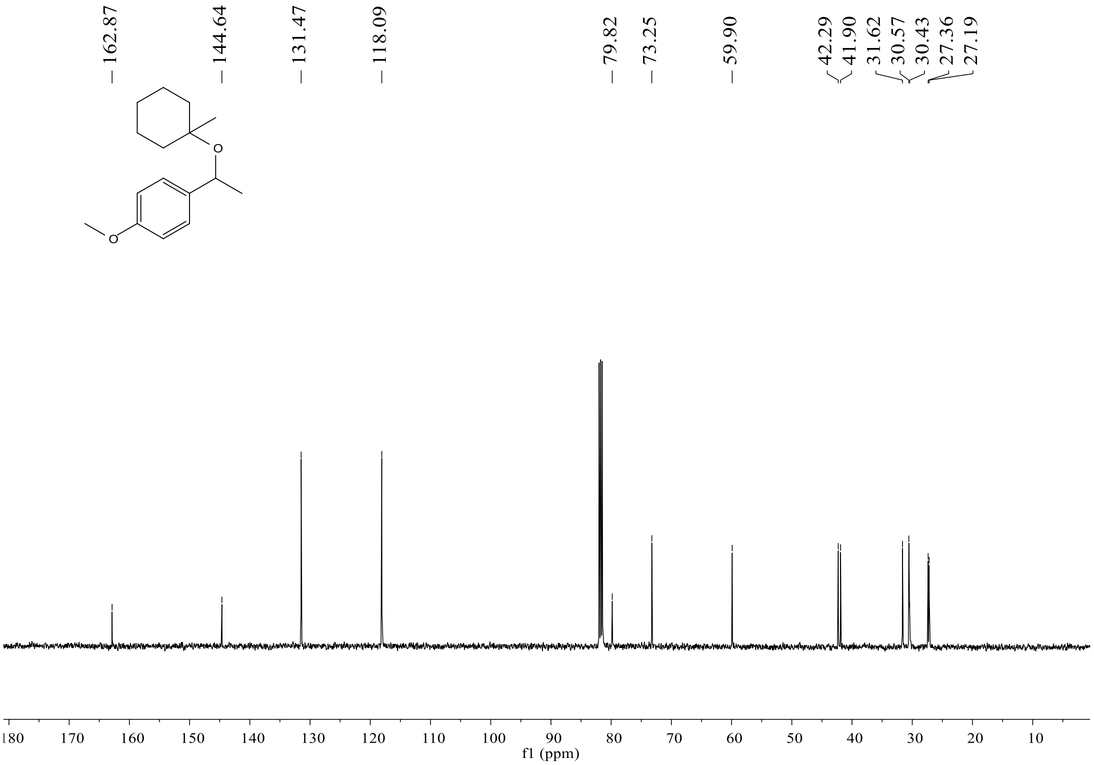

^{13}C NMR spectrum in $CDCl_3$.

14d

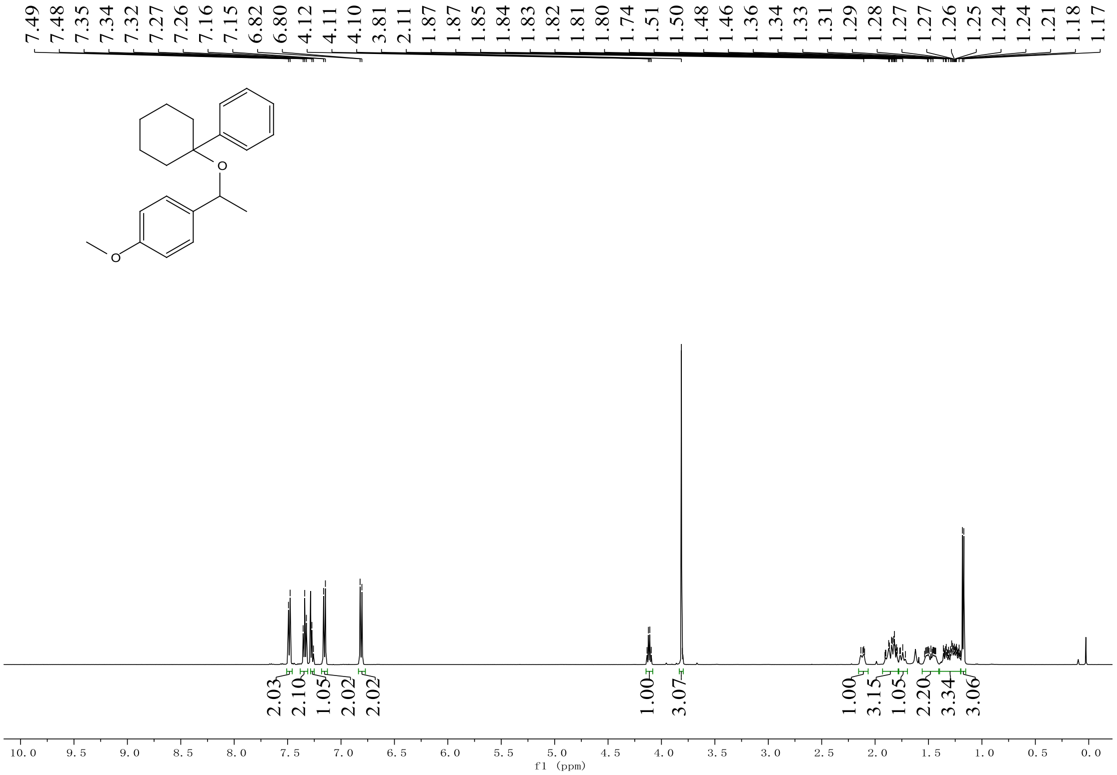

^{1}H NMR spectrum in $CDCl_3$.

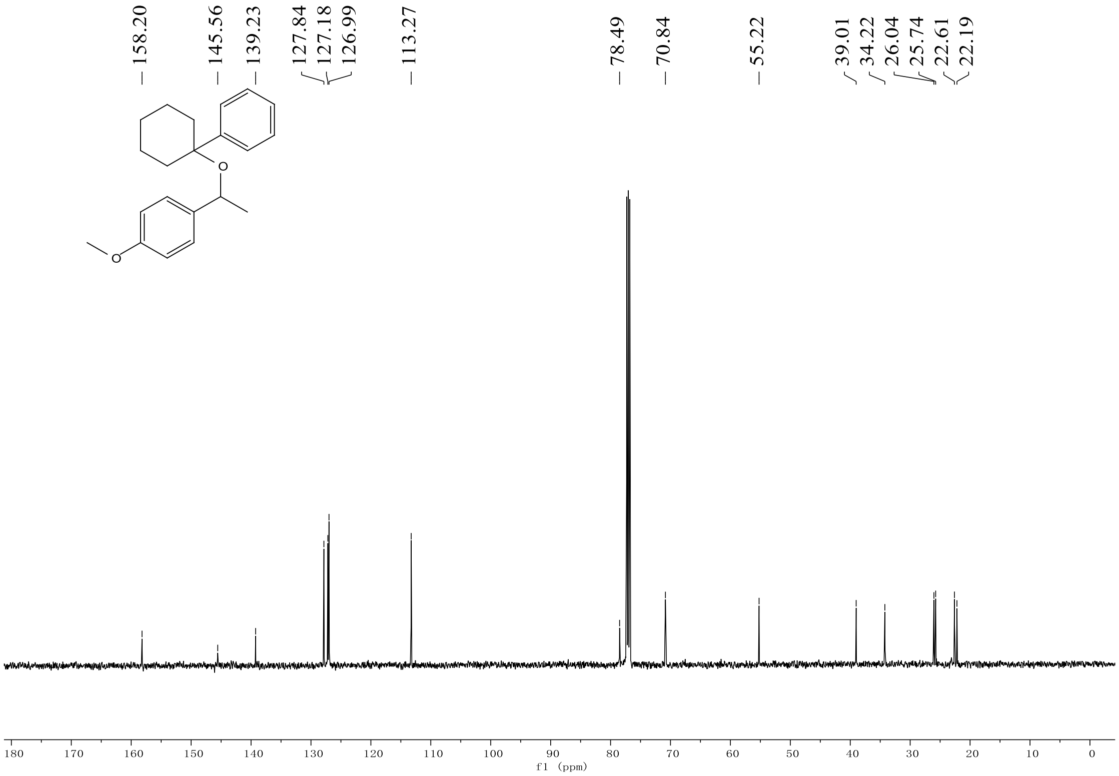

^{13}C NMR spectrum in $CDCl_3$.

15d

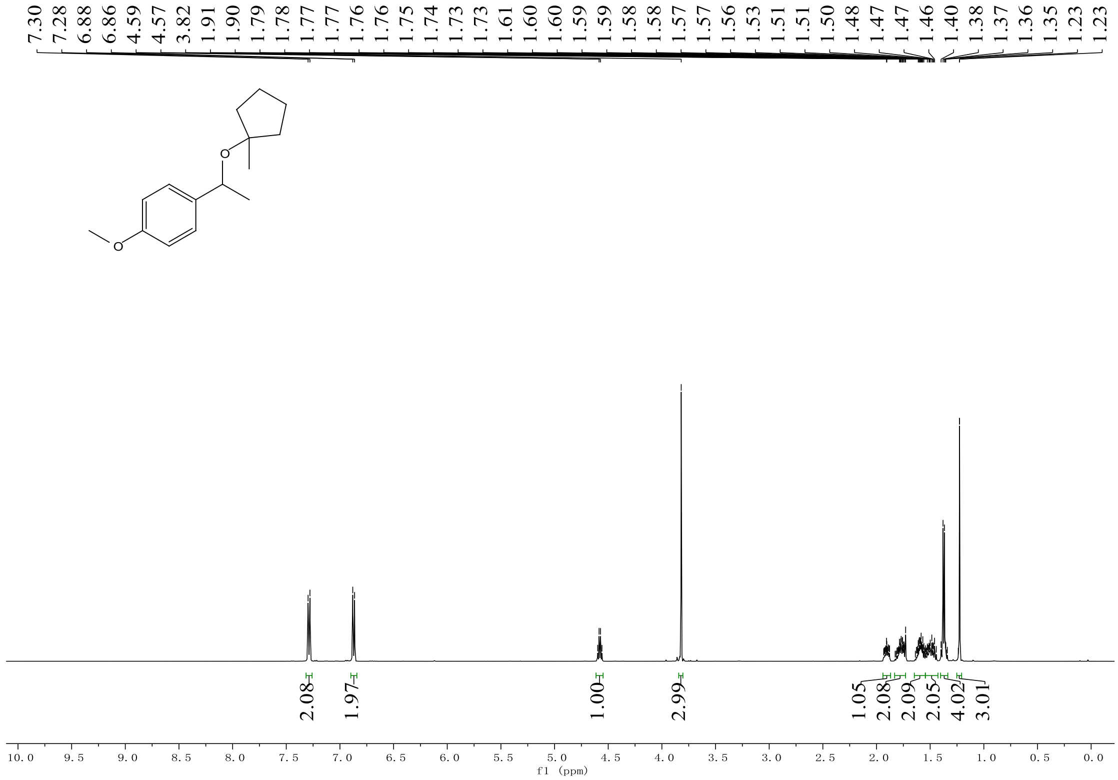

^{1}H NMR spectrum in $CDCl_3$.

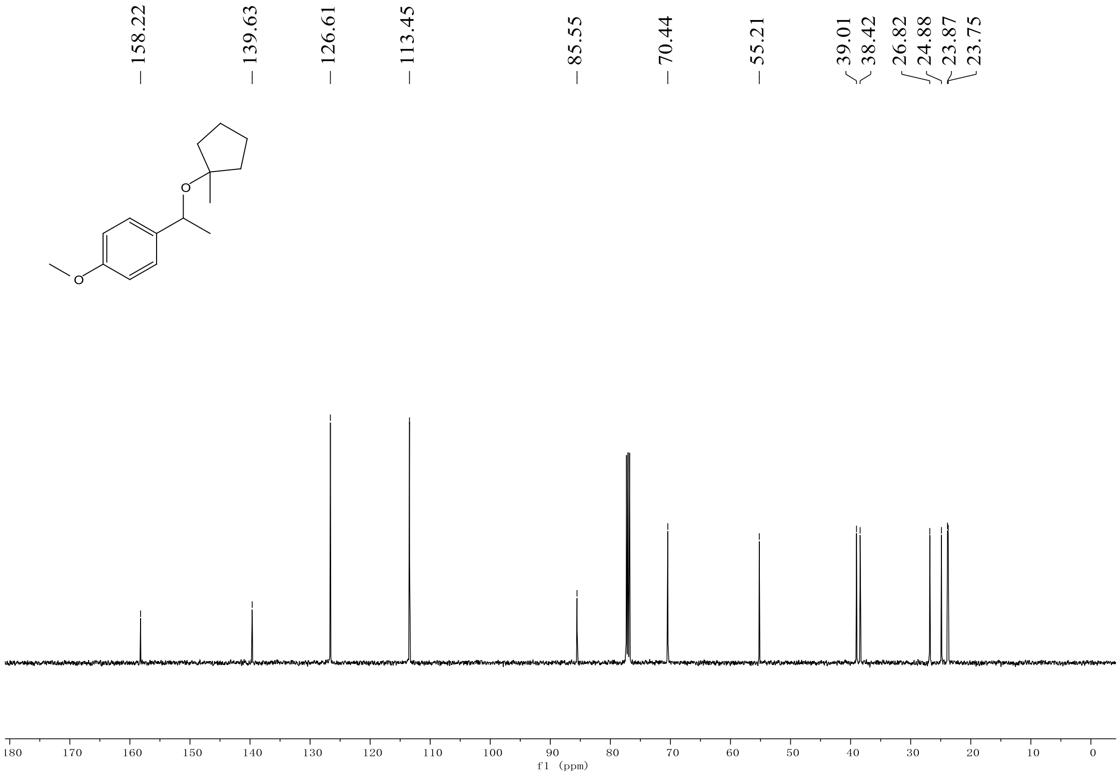

^{13}C NMR spectrum in $CDCl_3$.

16d

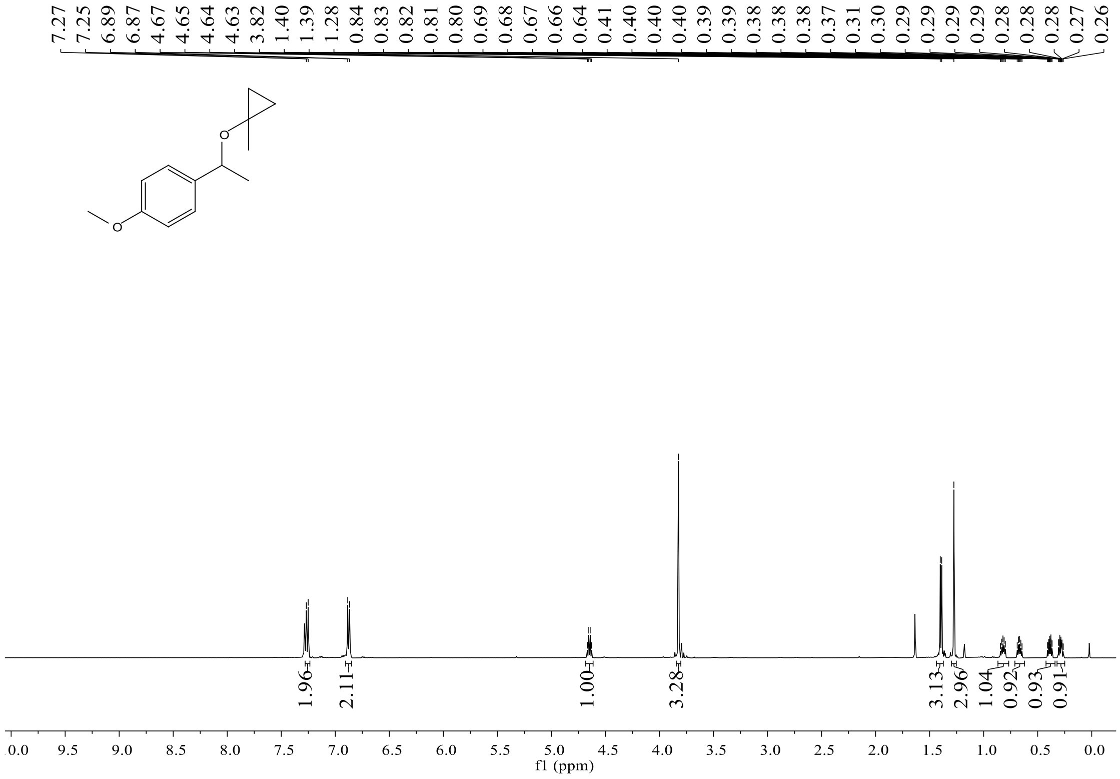

^{1}H NMR spectrum in $CDCl_3$.

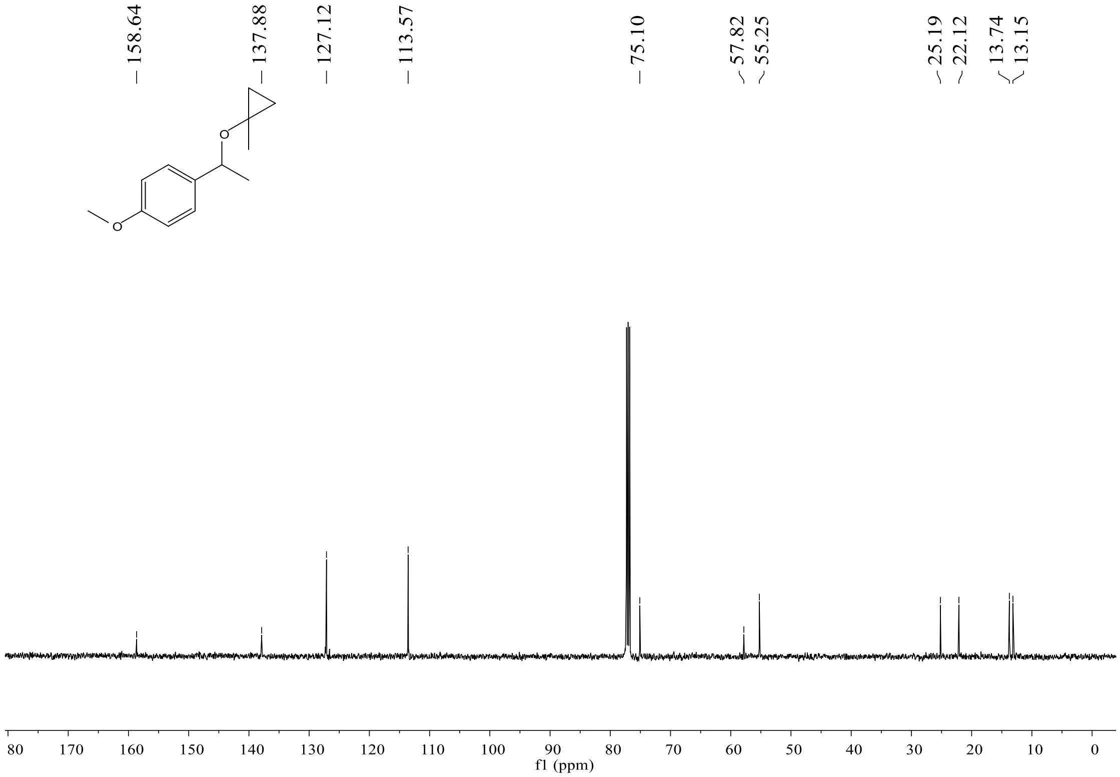

^{13}C NMR spectrum in $CDCl_3$.

17d

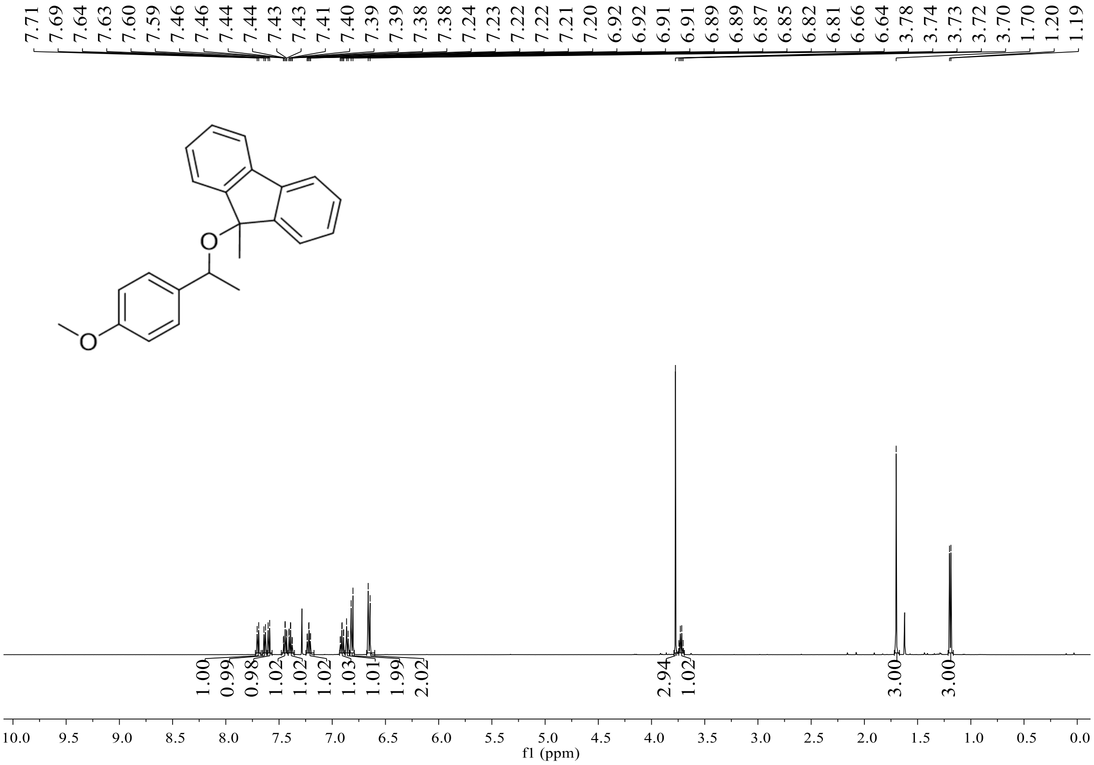

^{1}H NMR spectrum in $CDCl_3$.

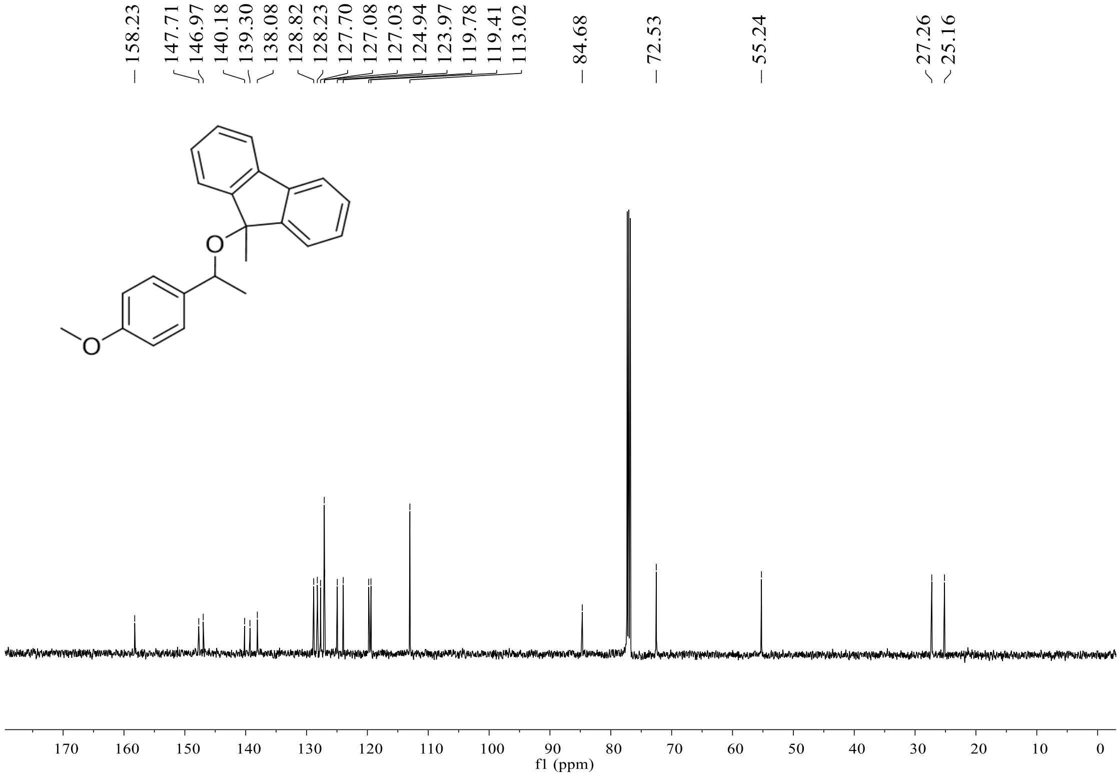

^{13}C NMR spectrum in $CDCl_3$.

18d

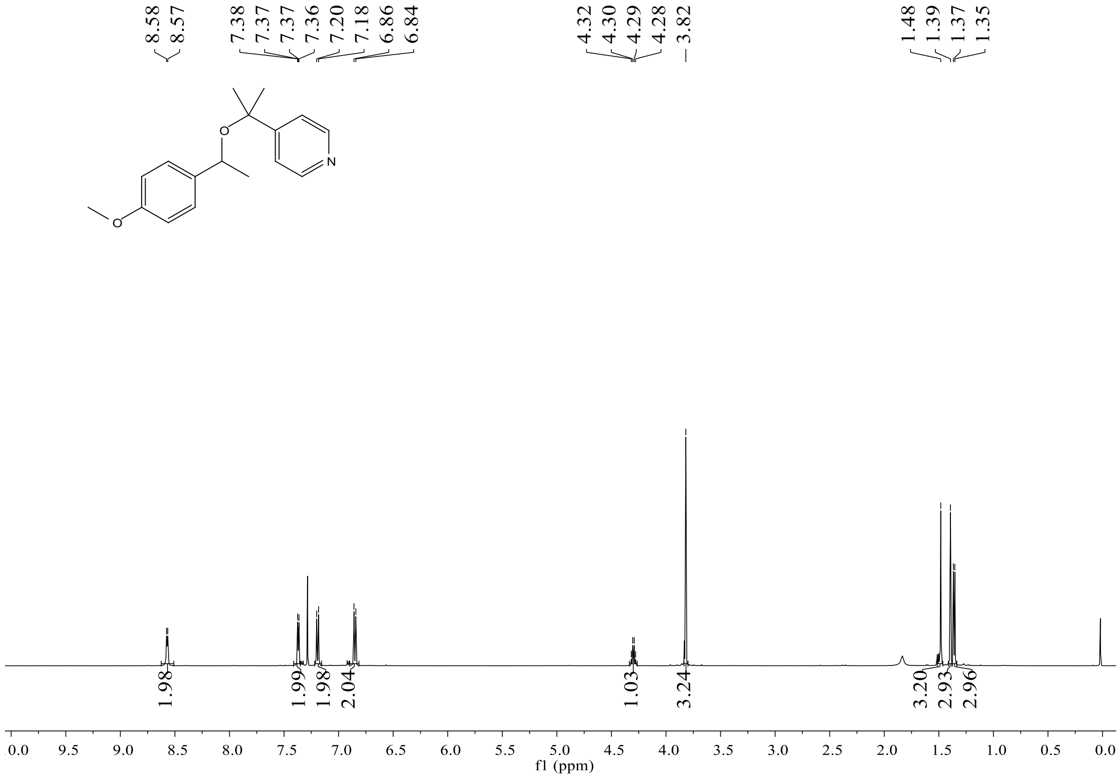

^{1}H NMR spectrum in $CDCl_3$.

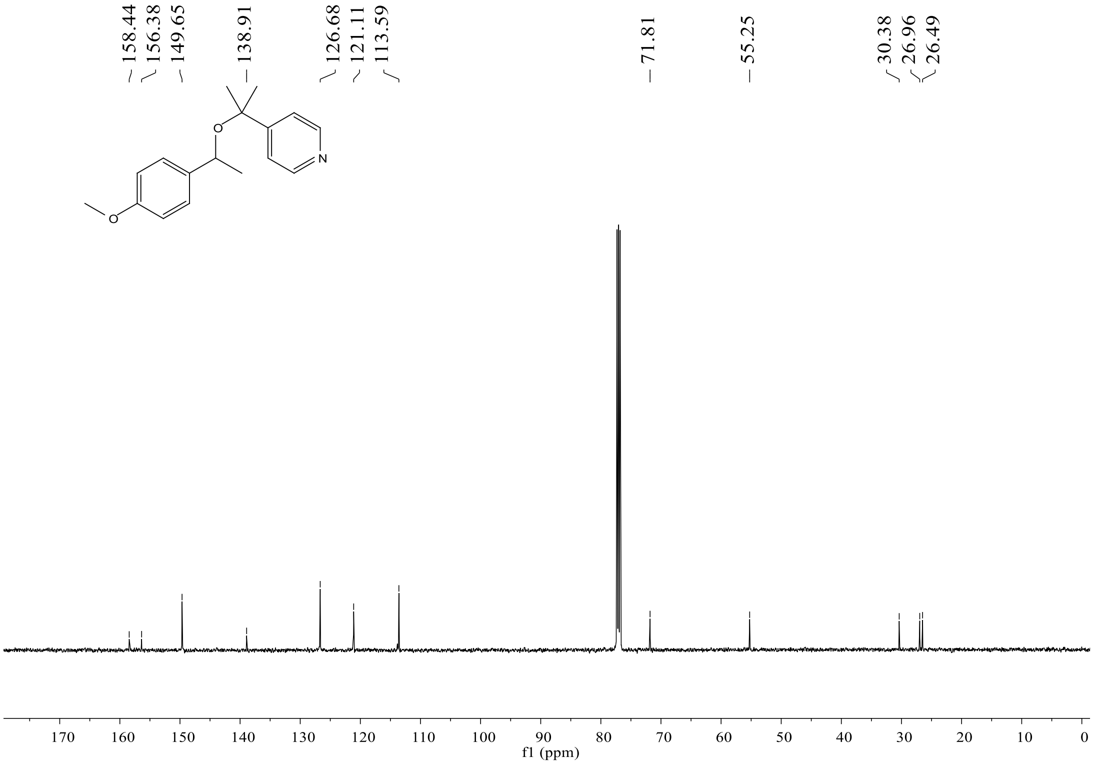

^{13}C NMR spectrum in $CDCl_3$.

19d

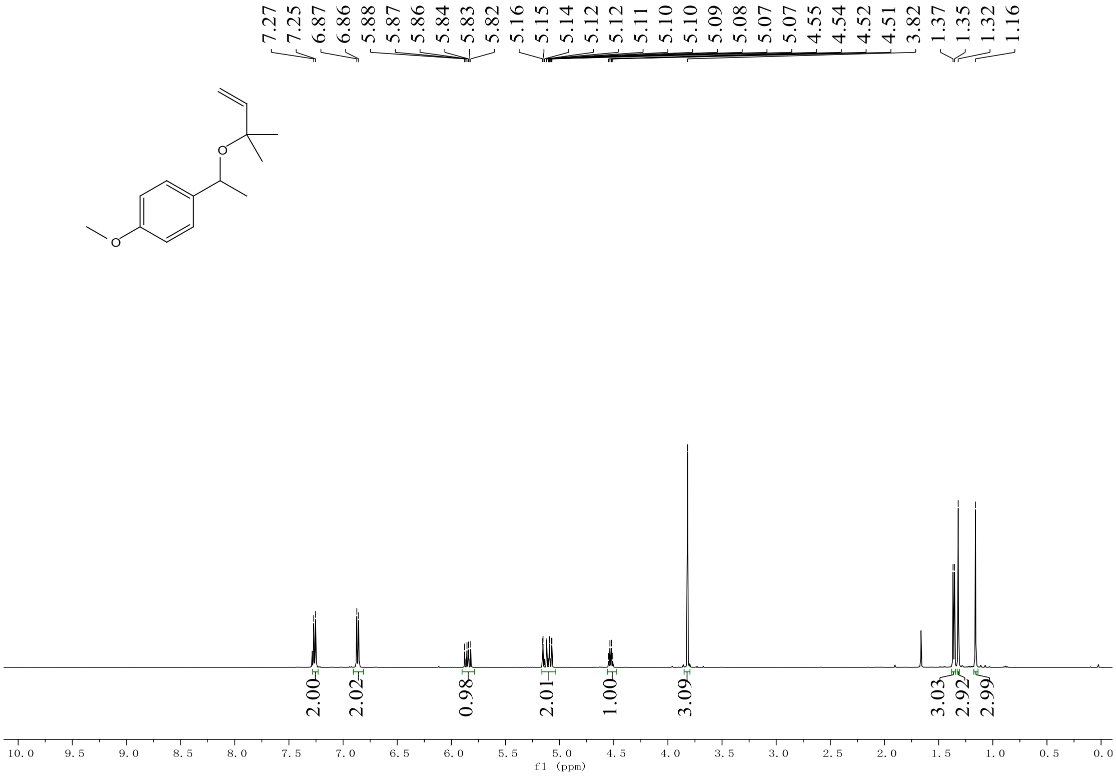

^{1}H NMR spectrum in $CDCl_3$.

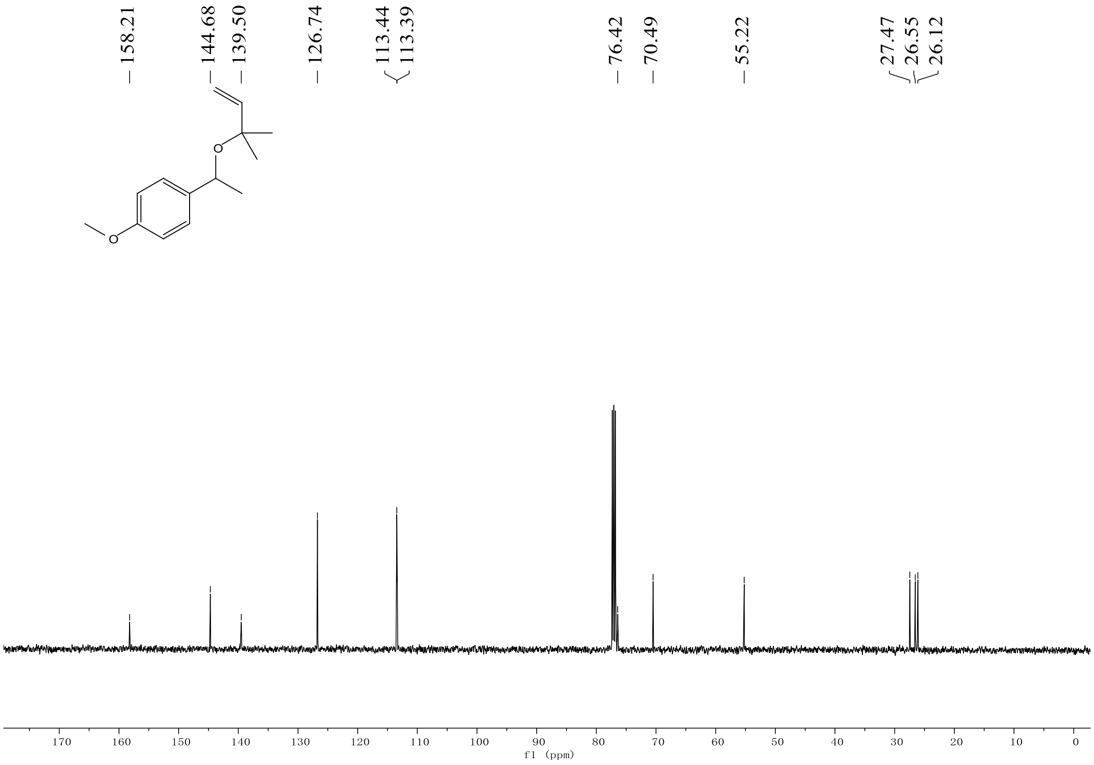

^{13}C NMR spectrum in $CDCl_3$.

20d

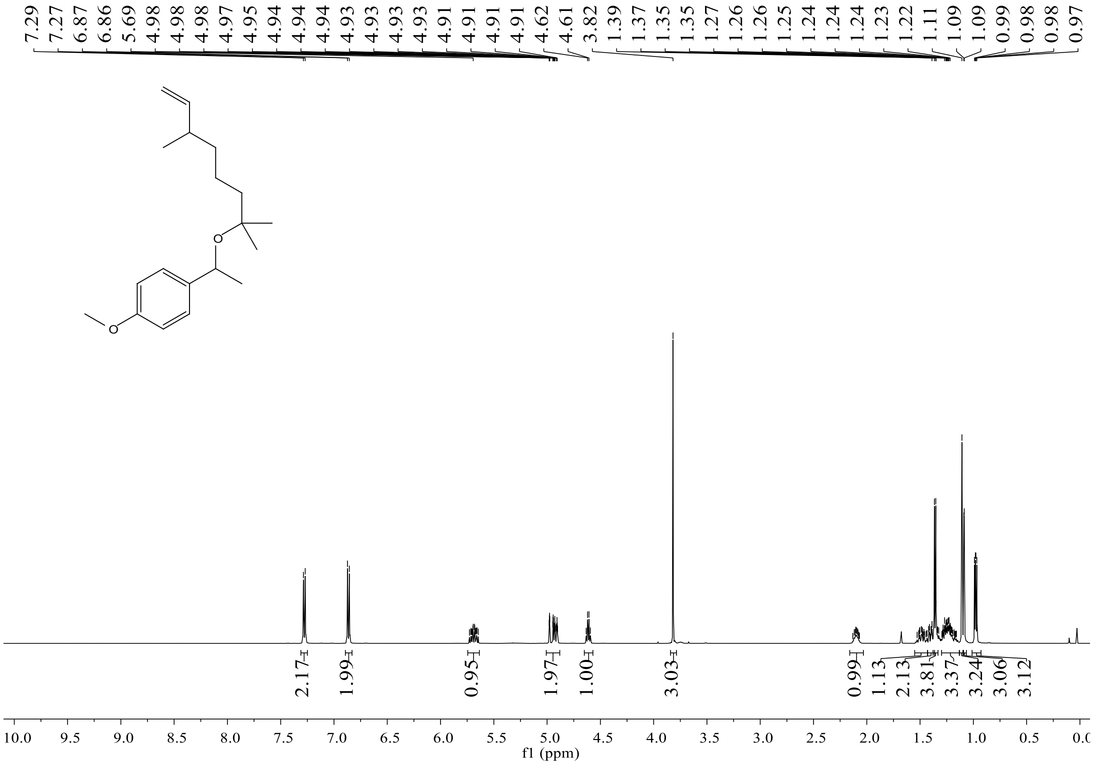

^{1}H NMR spectrum in $CDCl_3$.

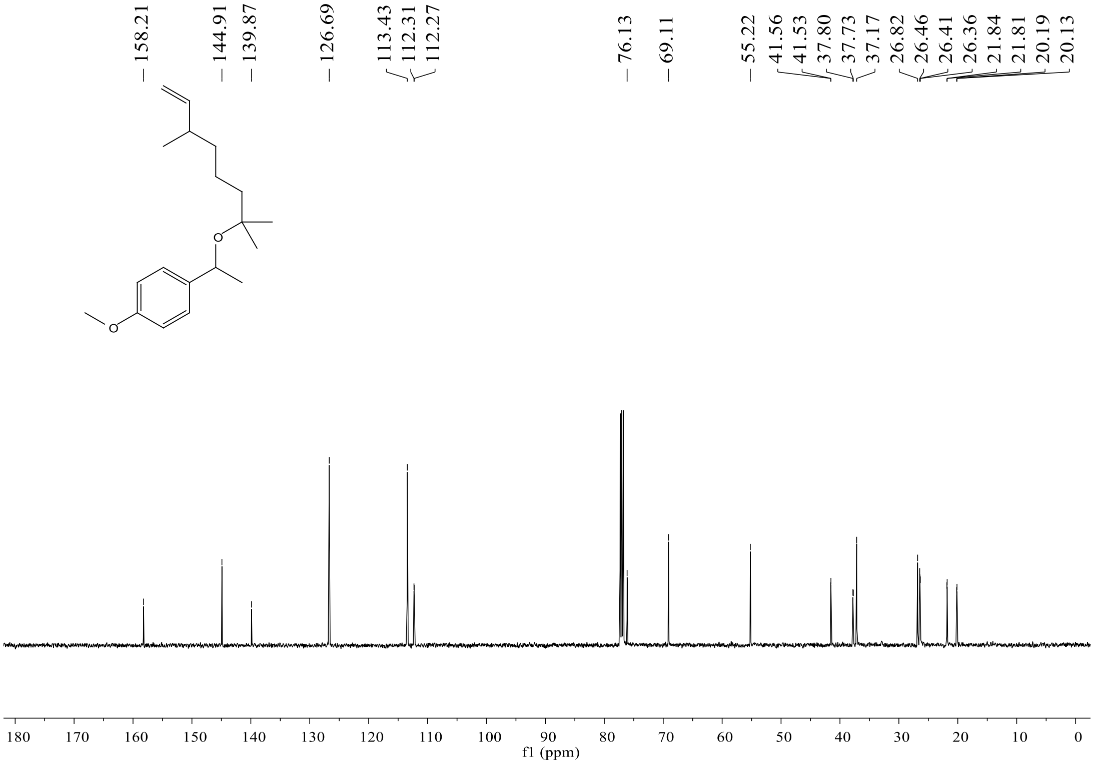

^{13}C NMR spectrum in $CDCl_3$.

21d

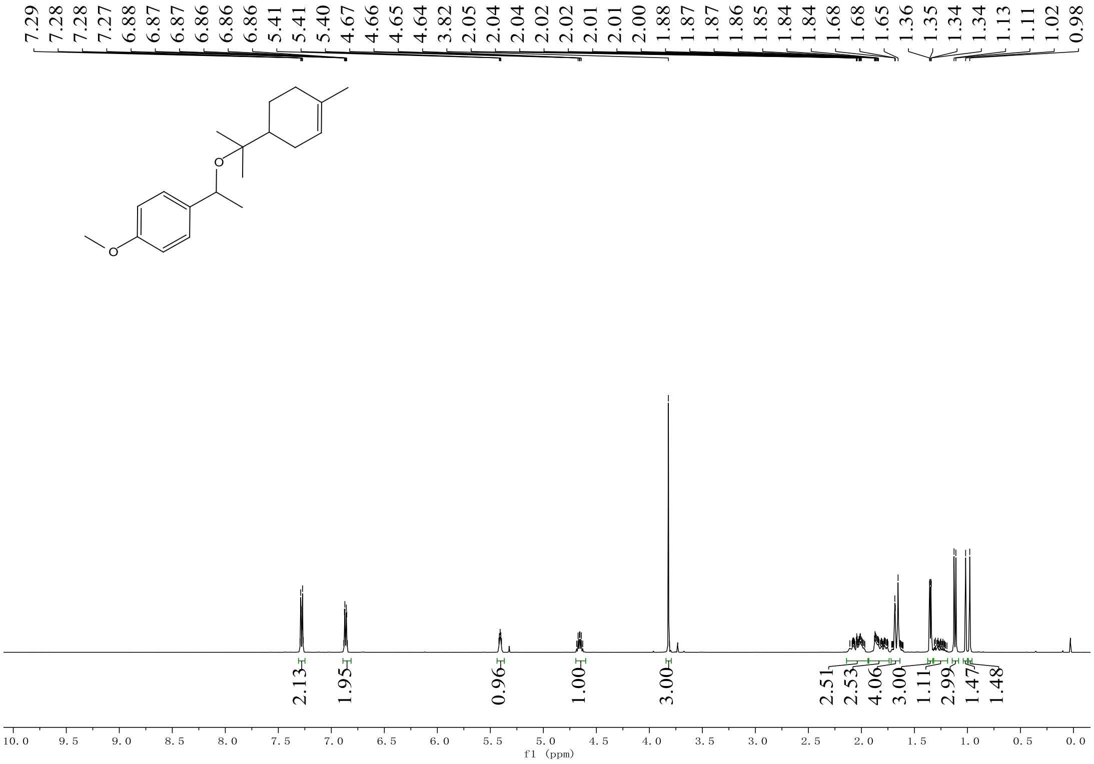

^{1}H NMR spectrum in $CDCl_3$.

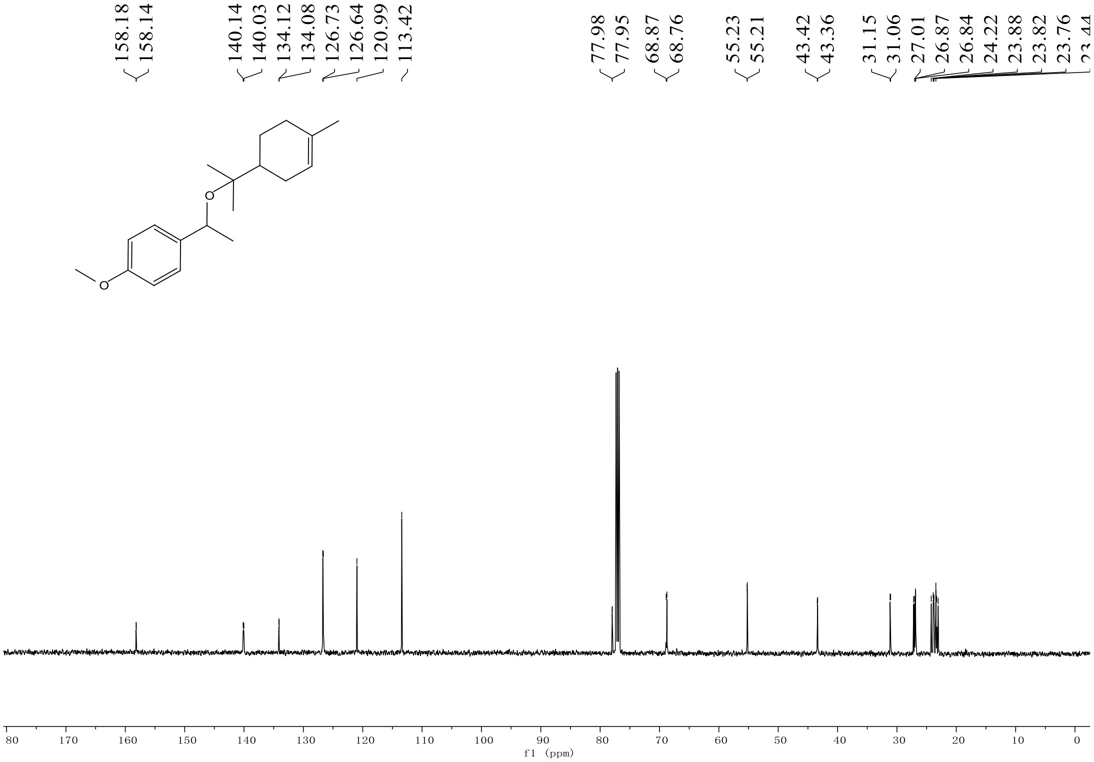

^{13}C NMR spectrum in $CDCl_3$.

22d

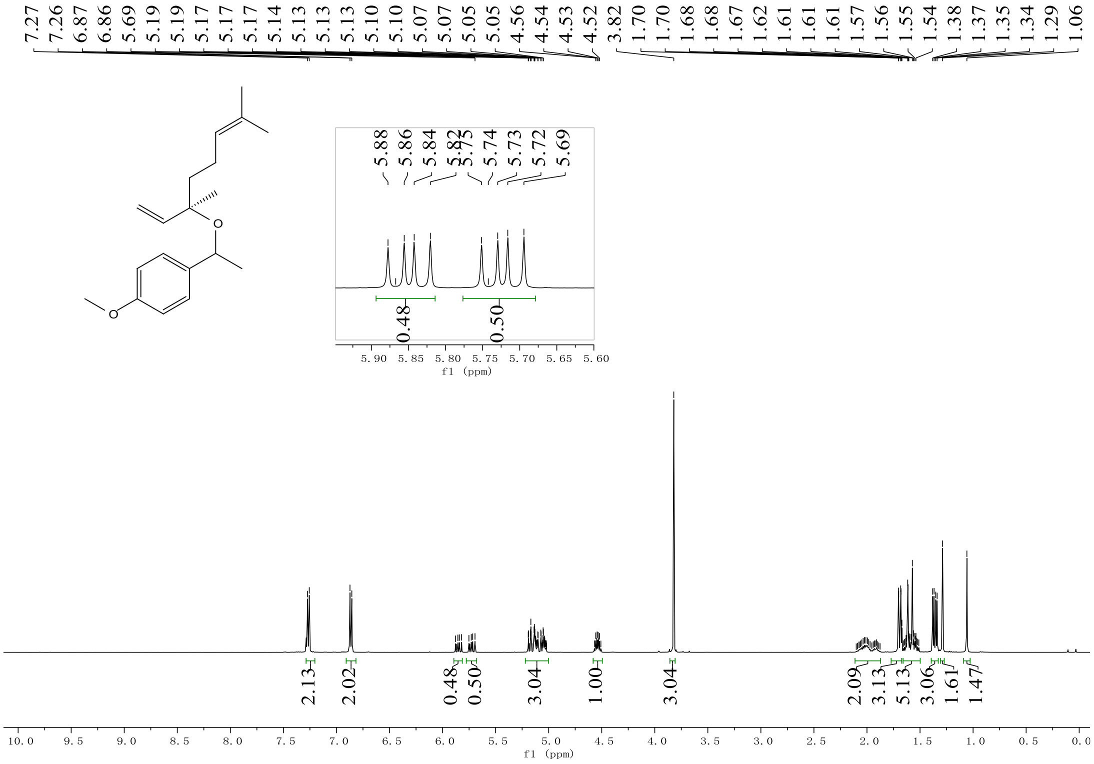

^{1}H NMR spectrum in $CDCl_3$.

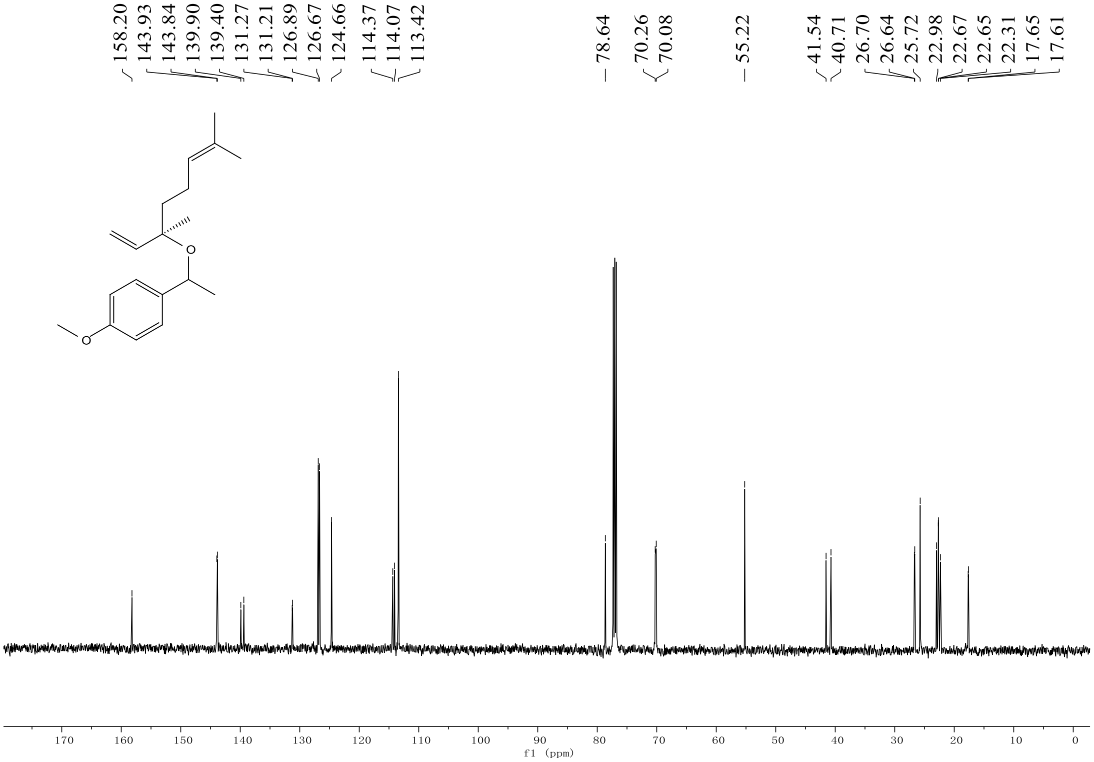

^{13}C NMR spectrum in $CDCl_3$.

23d

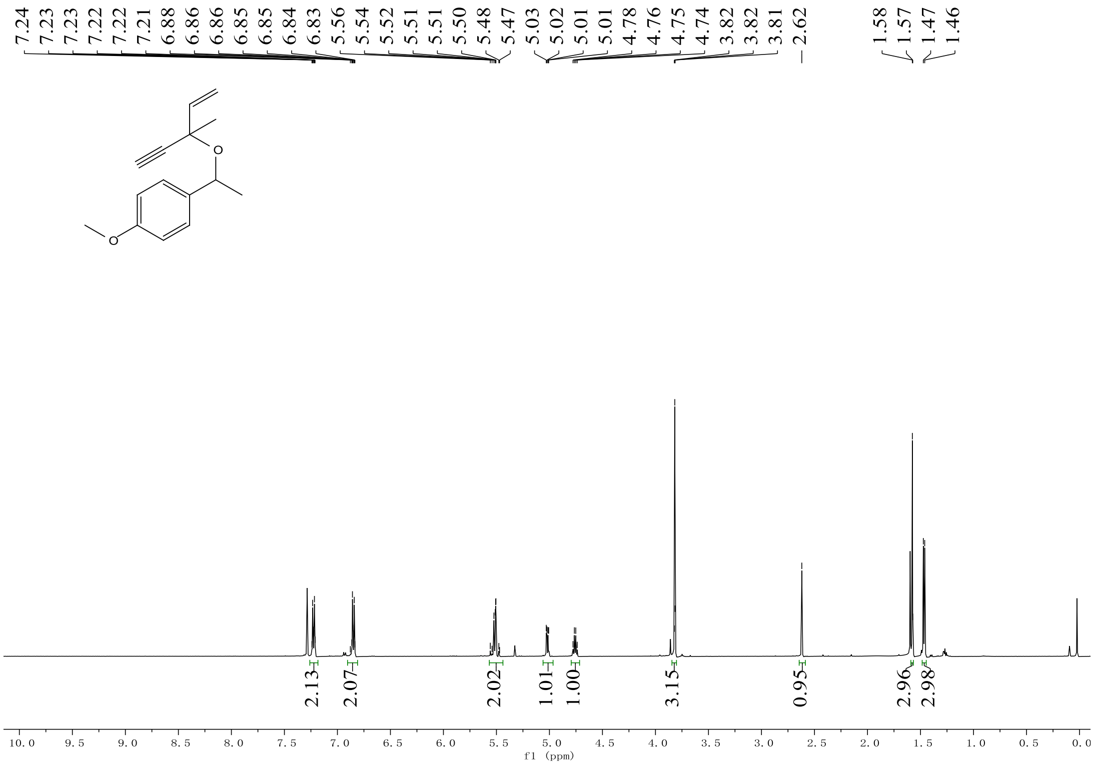

^{1}H NMR spectrum in $CDCl_3$.

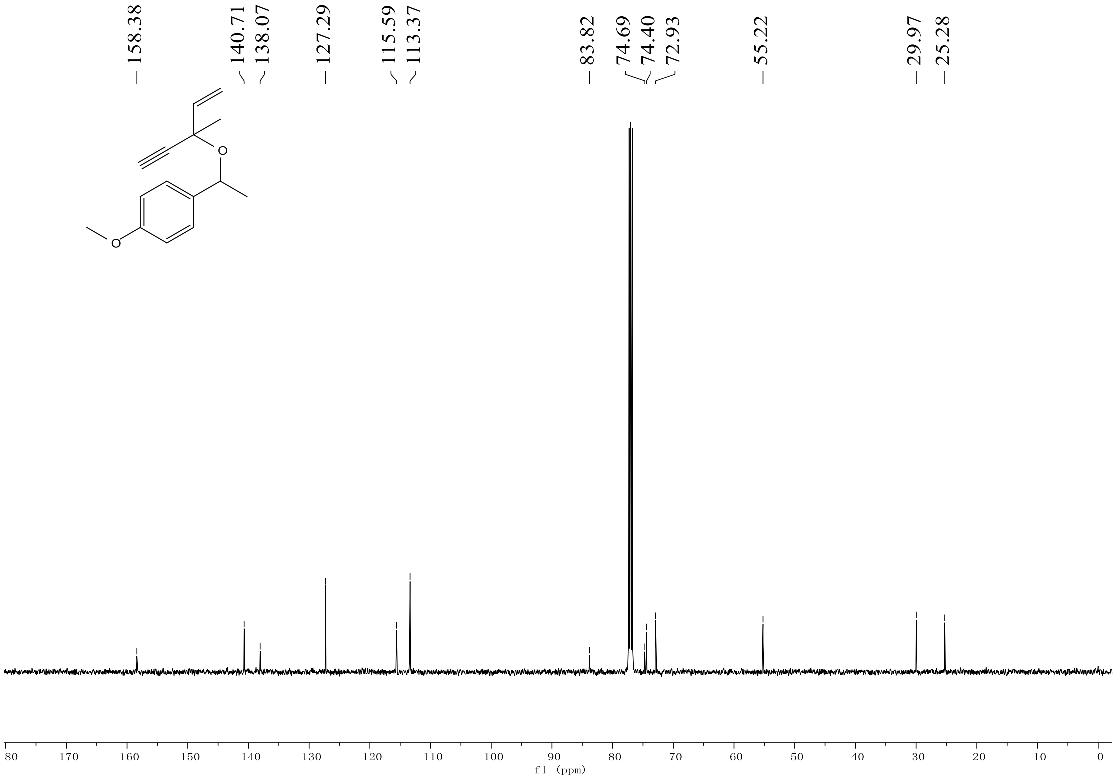

^{13}C NMR spectrum in $CDCl_3$.

24d

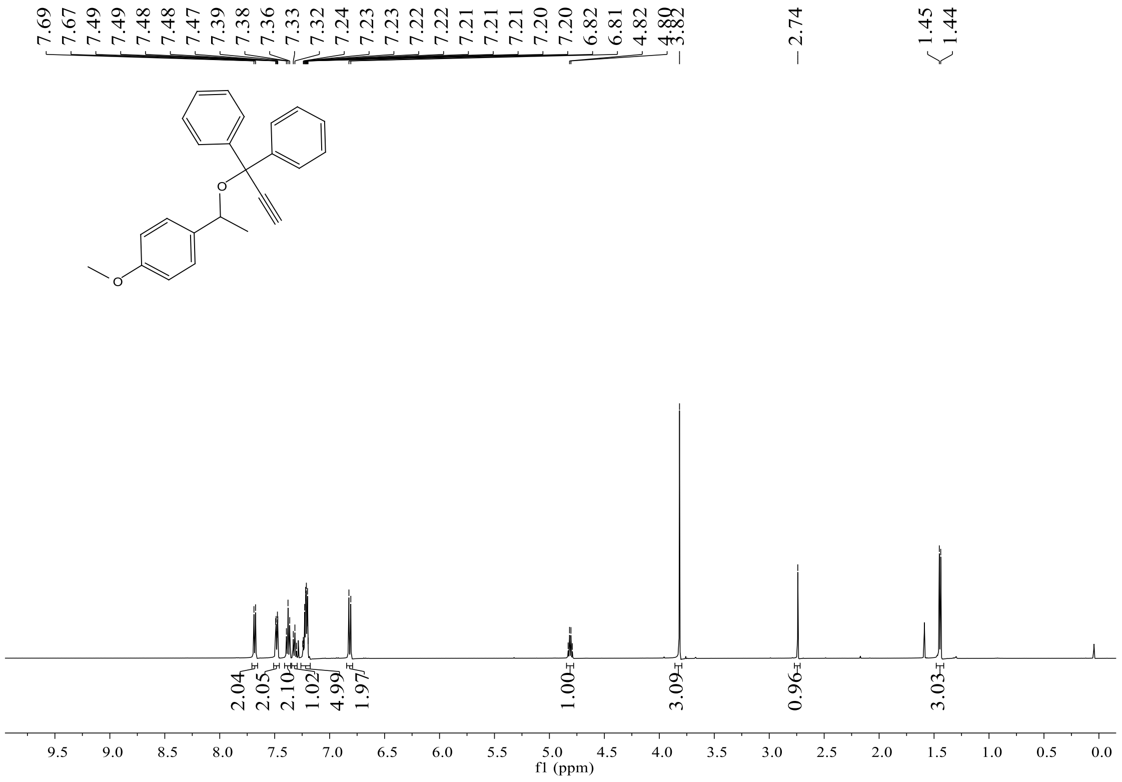

^{1}H NMR spectrum in $CDCl_3$.

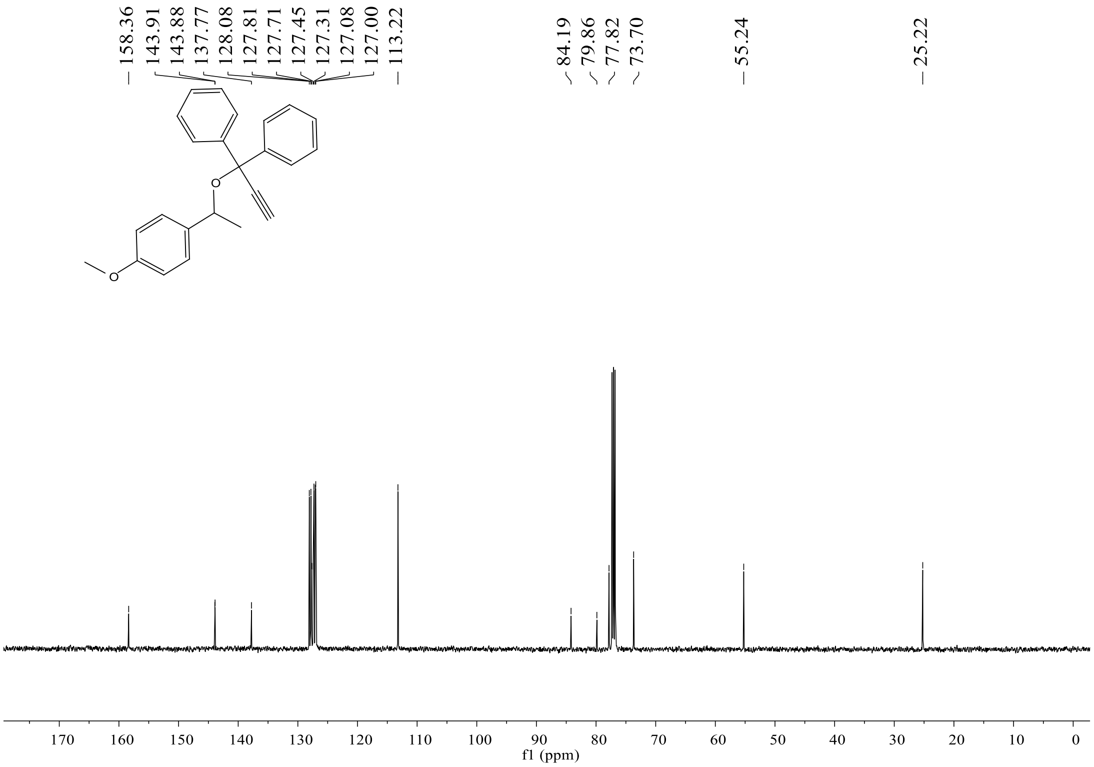

^{13}C NMR spectrum in $CDCl_3$.

25d

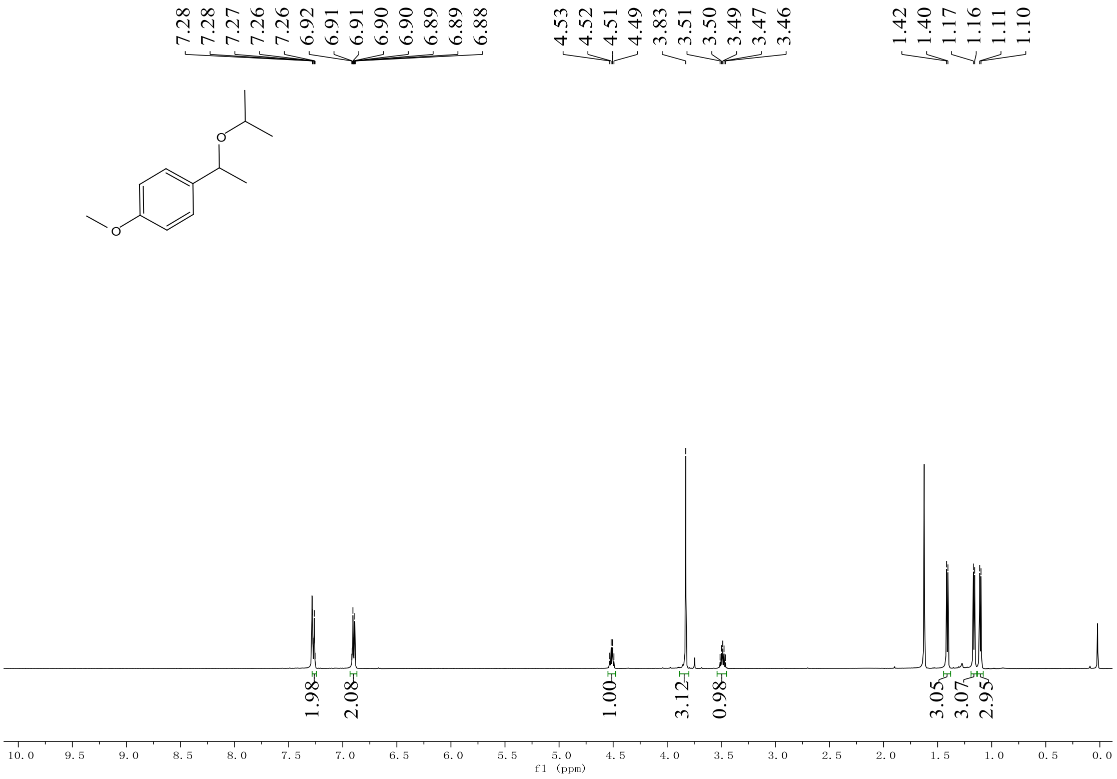

^{1}H NMR spectrum in $CDCl_3$.

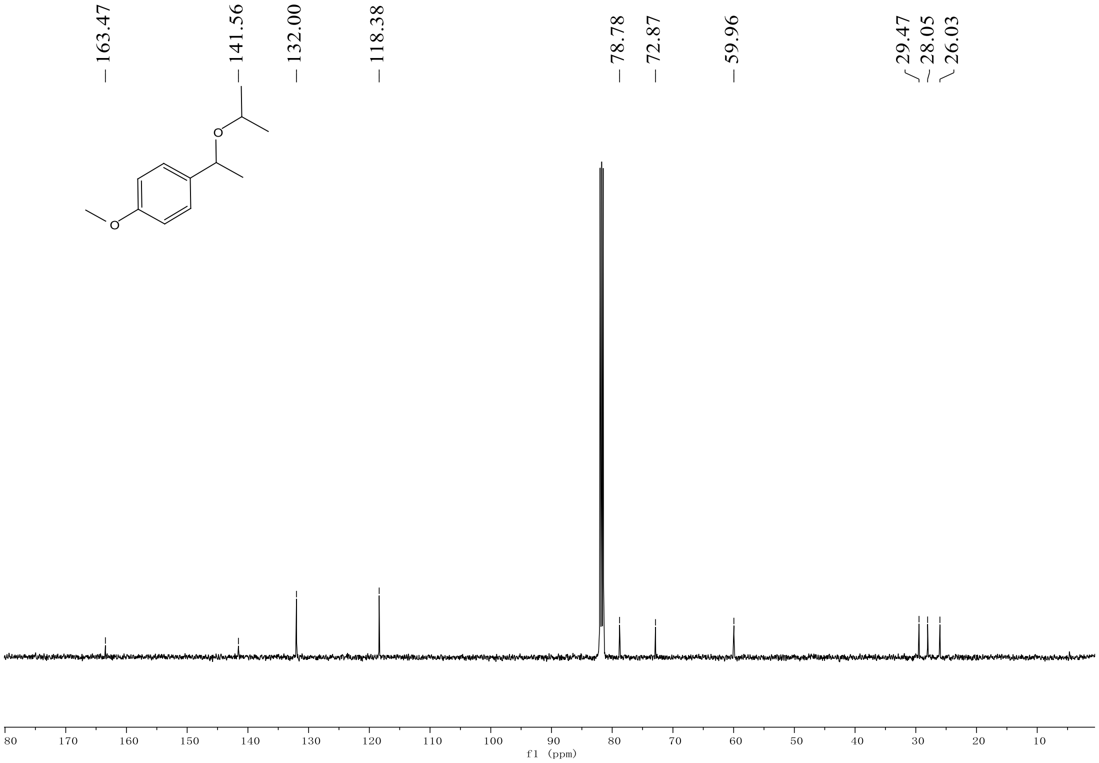

^{13}C NMR spectrum in $CDCl_3$.

26d

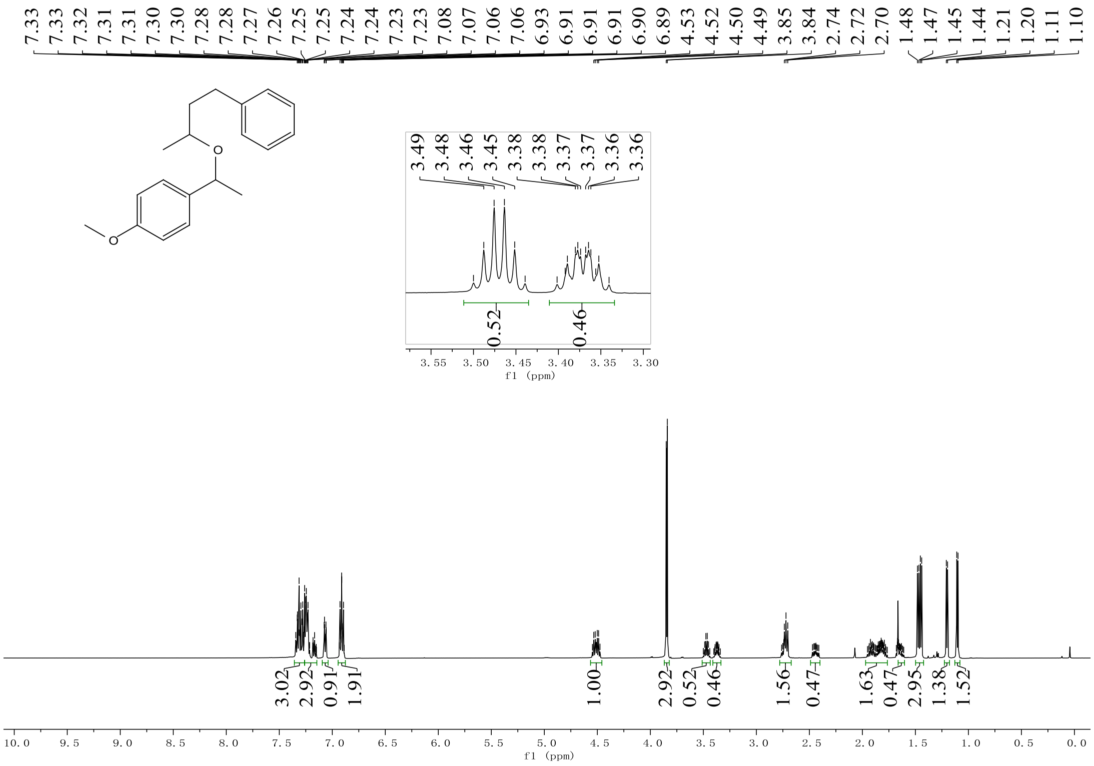

^{1}H NMR spectrum in $CDCl_3$.

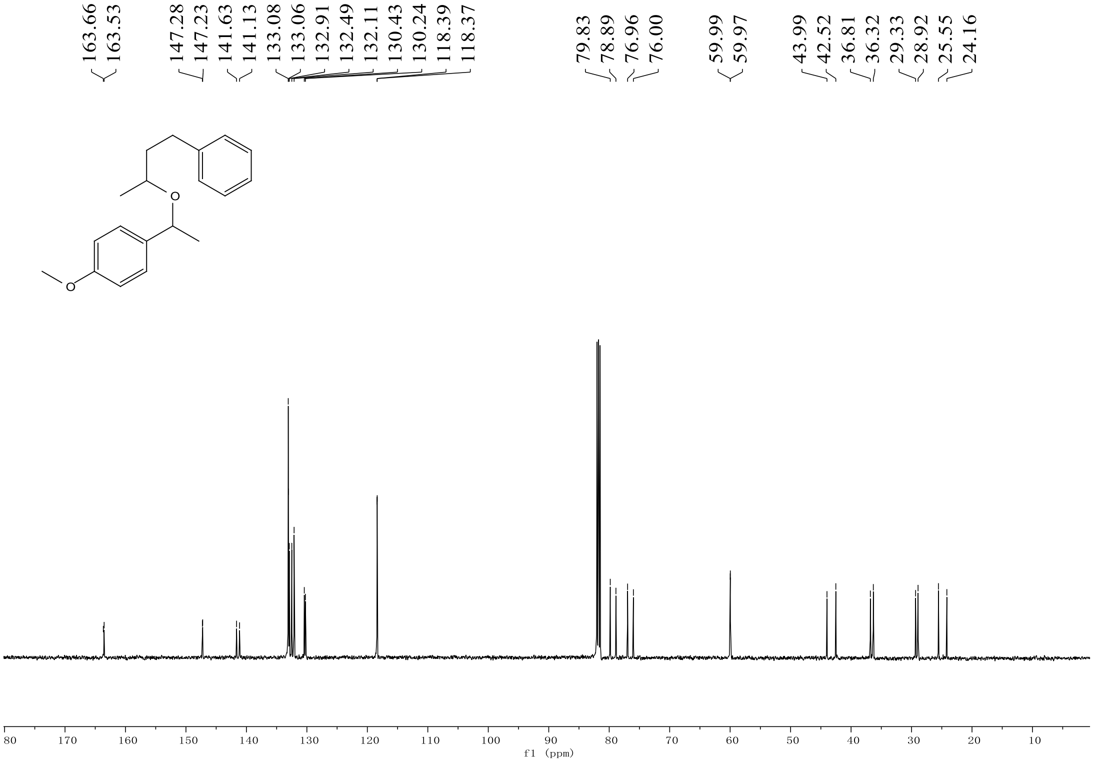

^{13}C NMR spectrum in $CDCl_3$.

27d

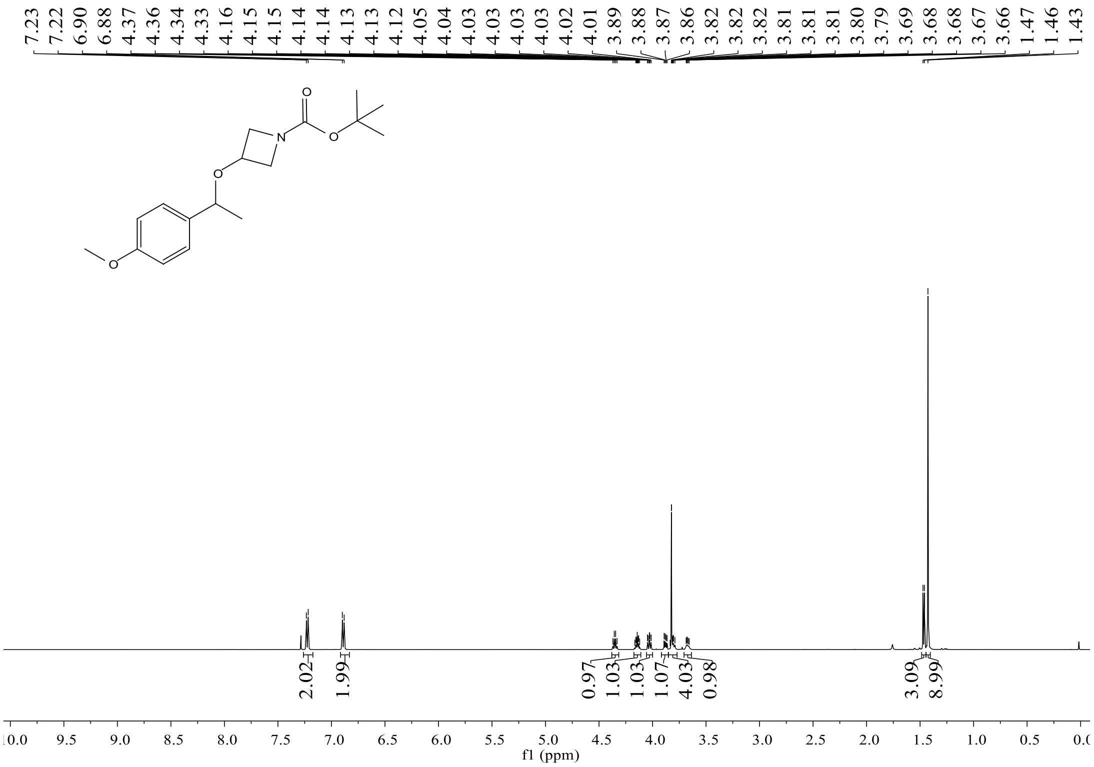

^{1}H NMR spectrum in $CDCl_3$.

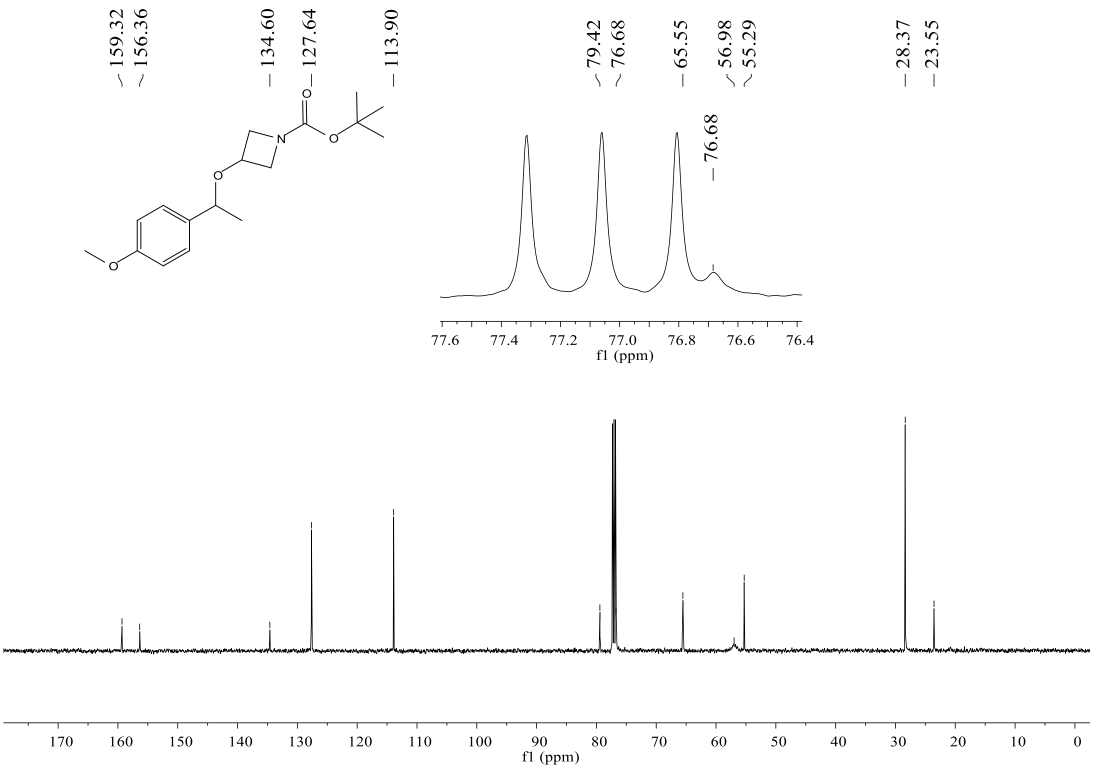

^{13}C NMR spectrum in $CDCl_3$.

28d

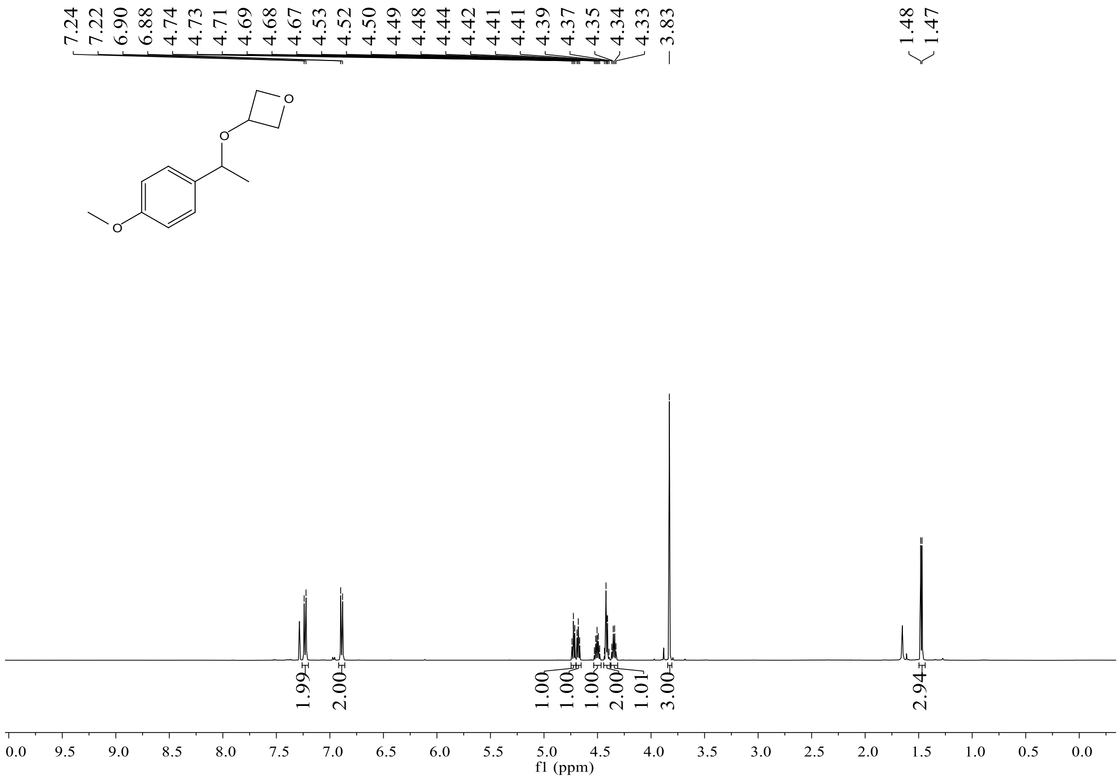

^{1}H NMR spectrum in $CDCl_3$.

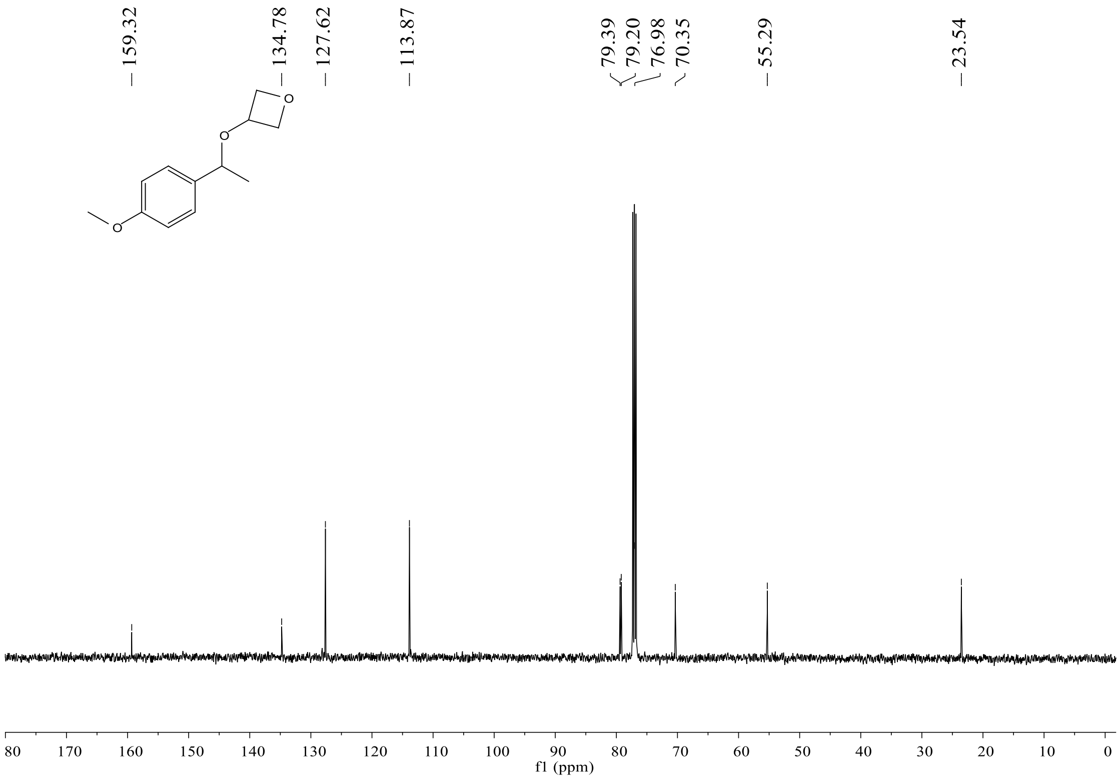

^{13}C NMR spectrum in $CDCl_3$.

29d

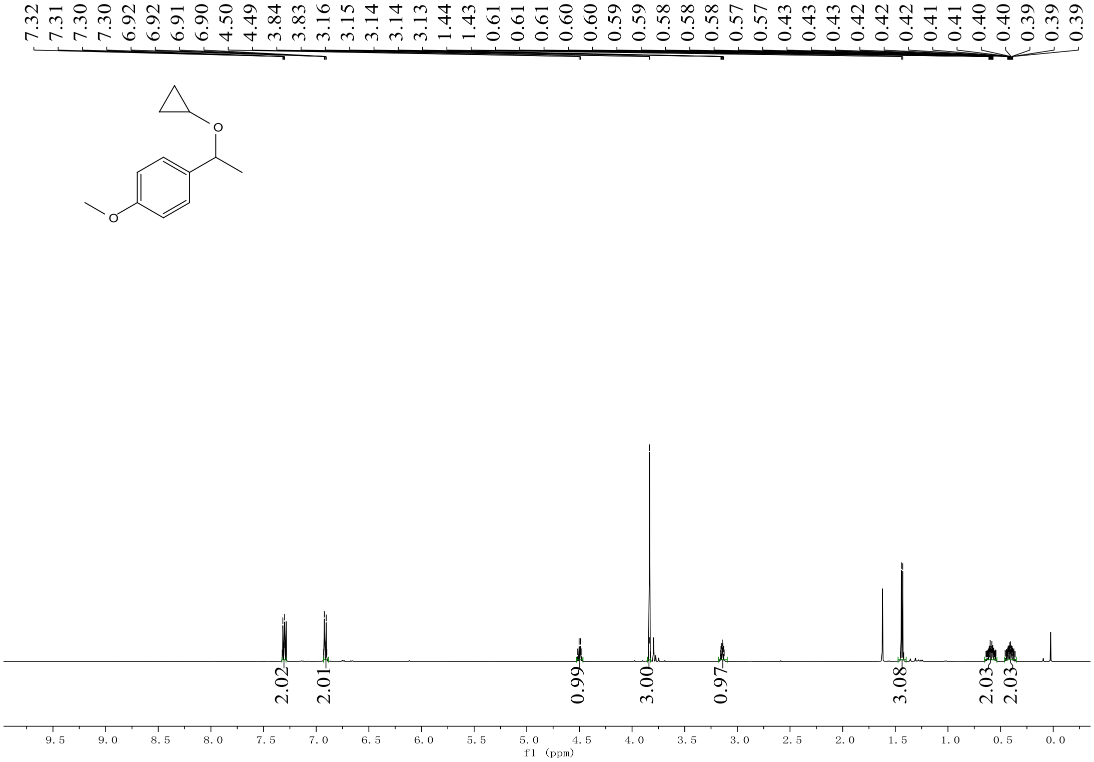

^{1}H NMR spectrum in $CDCl_3$.

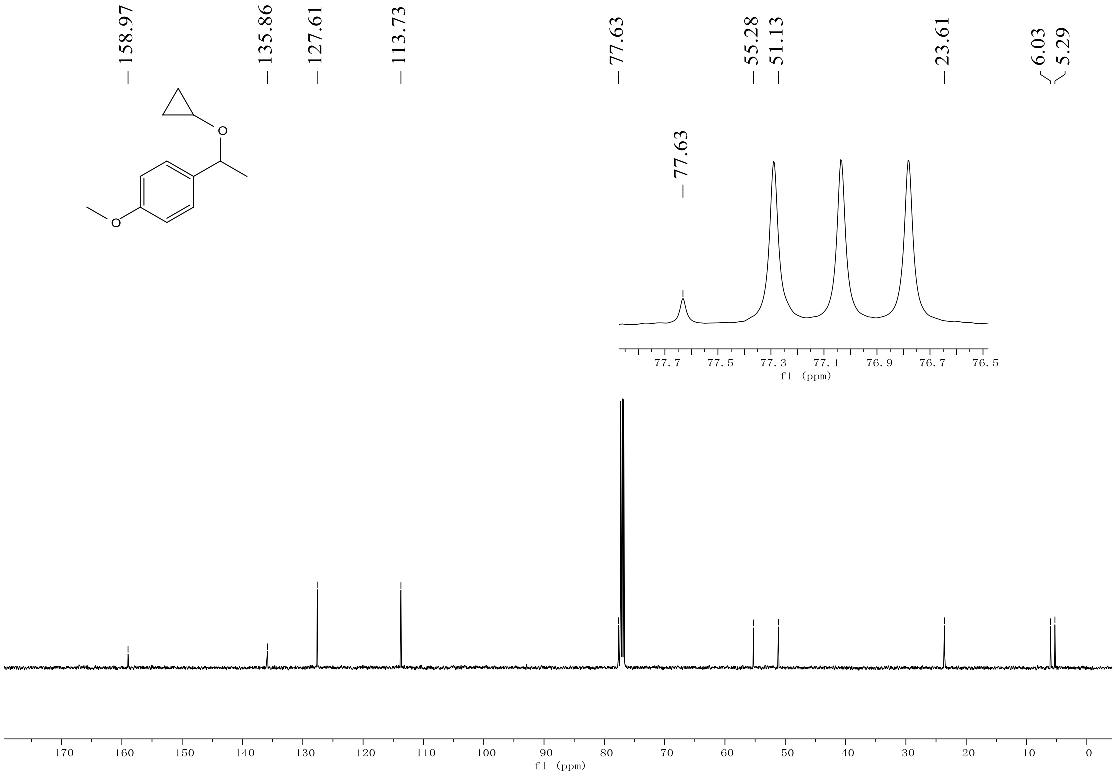

^{13}C NMR spectrum in $CDCl_3$.

30d

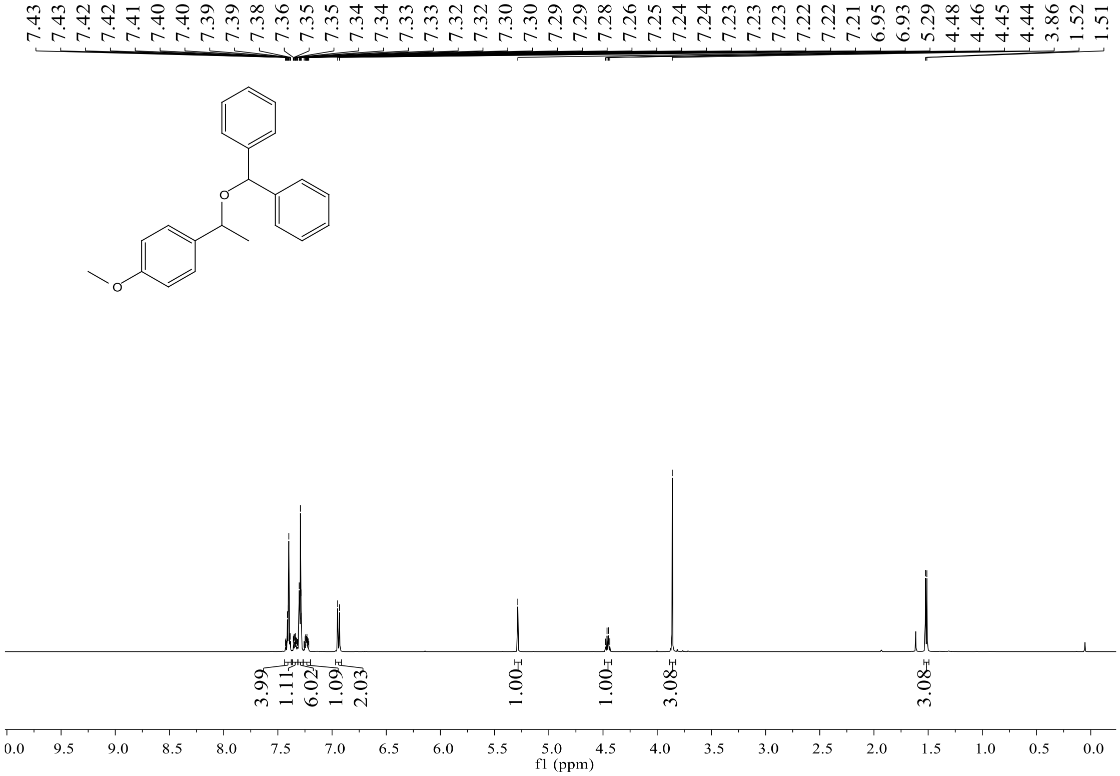

^{1}H NMR spectrum in $CDCl_3$.

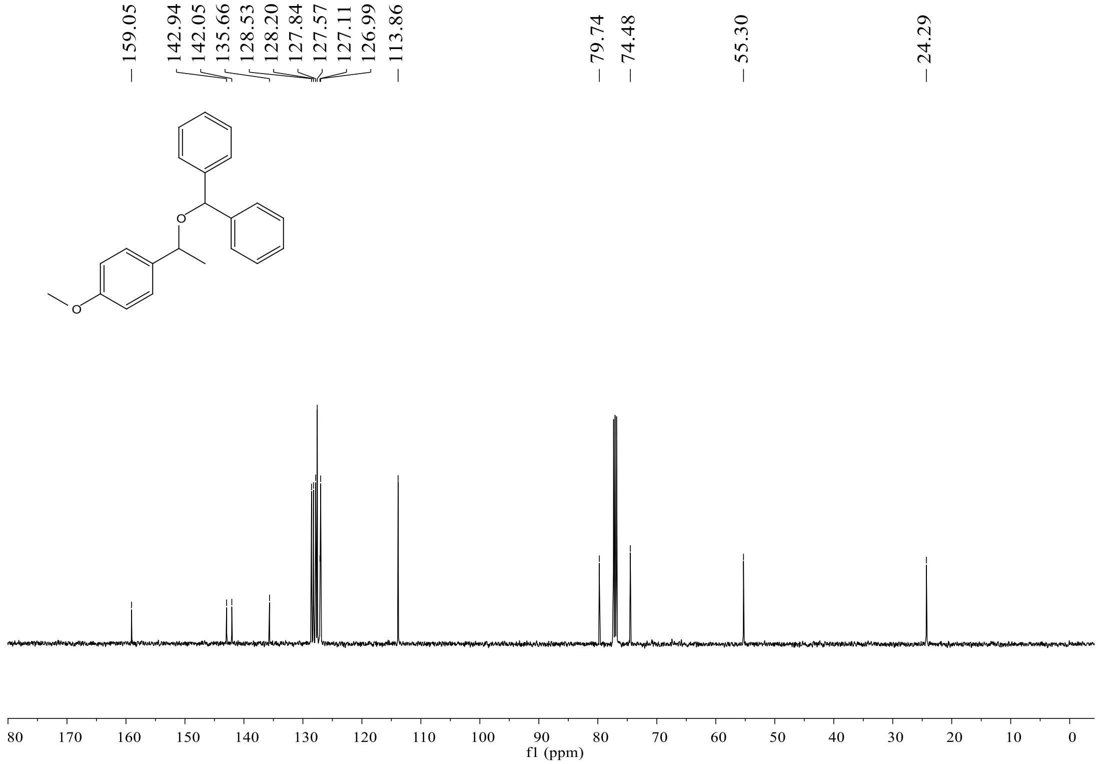

^{13}C NMR spectrum in $CDCl_3$.

31d

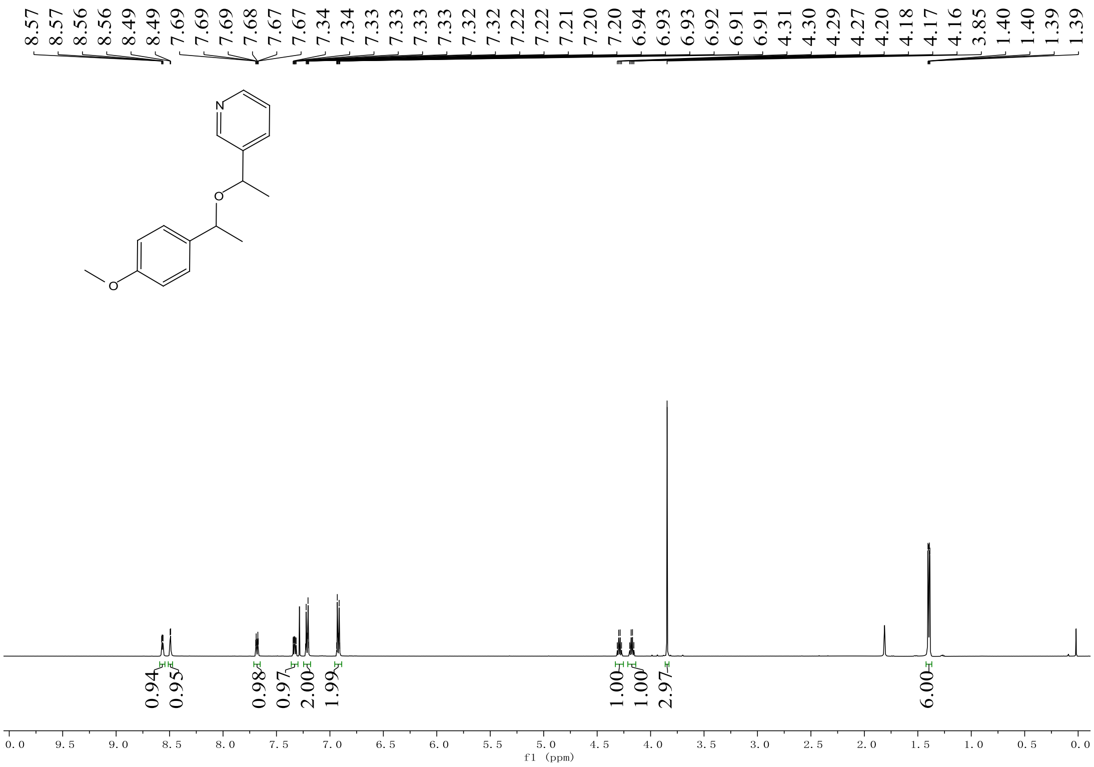

^{1}H NMR spectrum in $CDCl_3$.

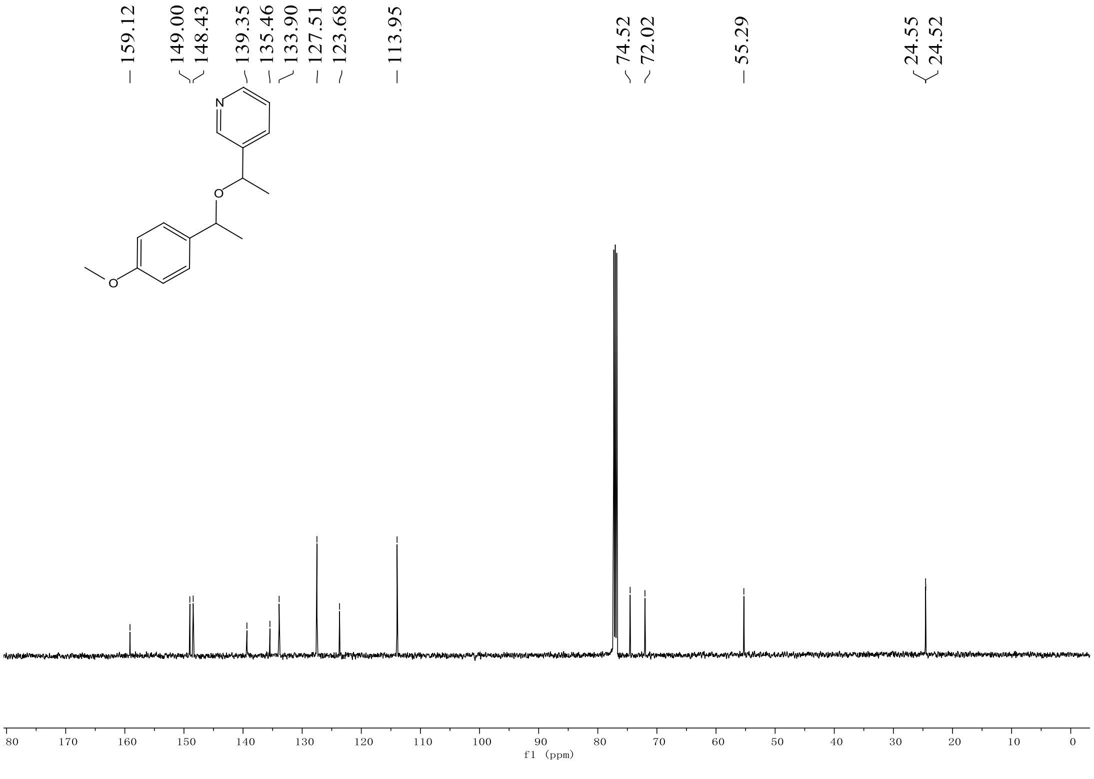

^{13}C NMR spectrum in $CDCl_3$.

32d

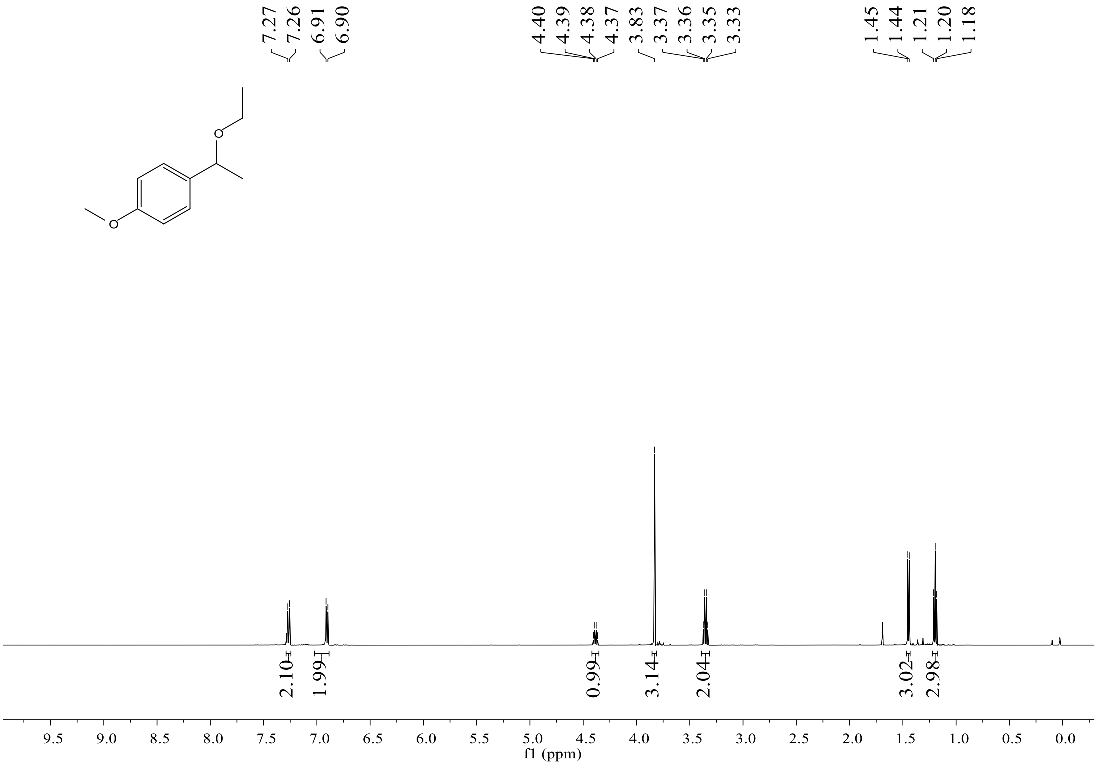

^{1}H NMR spectrum in $CDCl_3$.

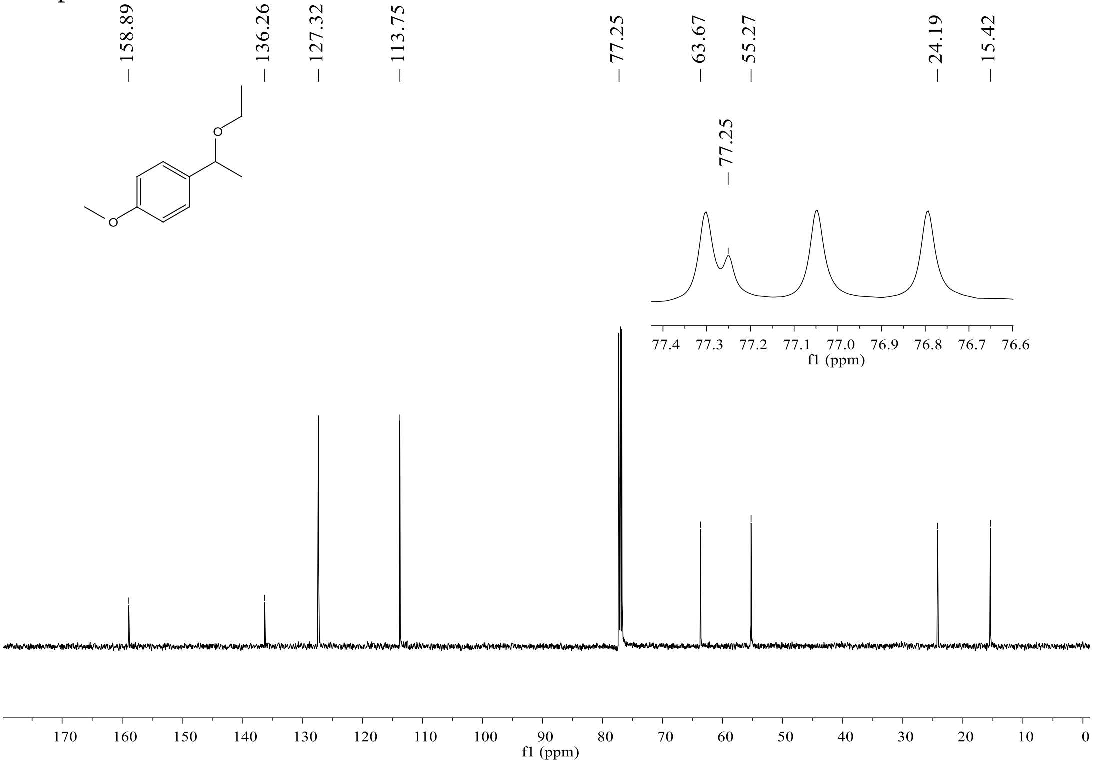

^{13}C NMR spectrum in $CDCl_3$.

33d

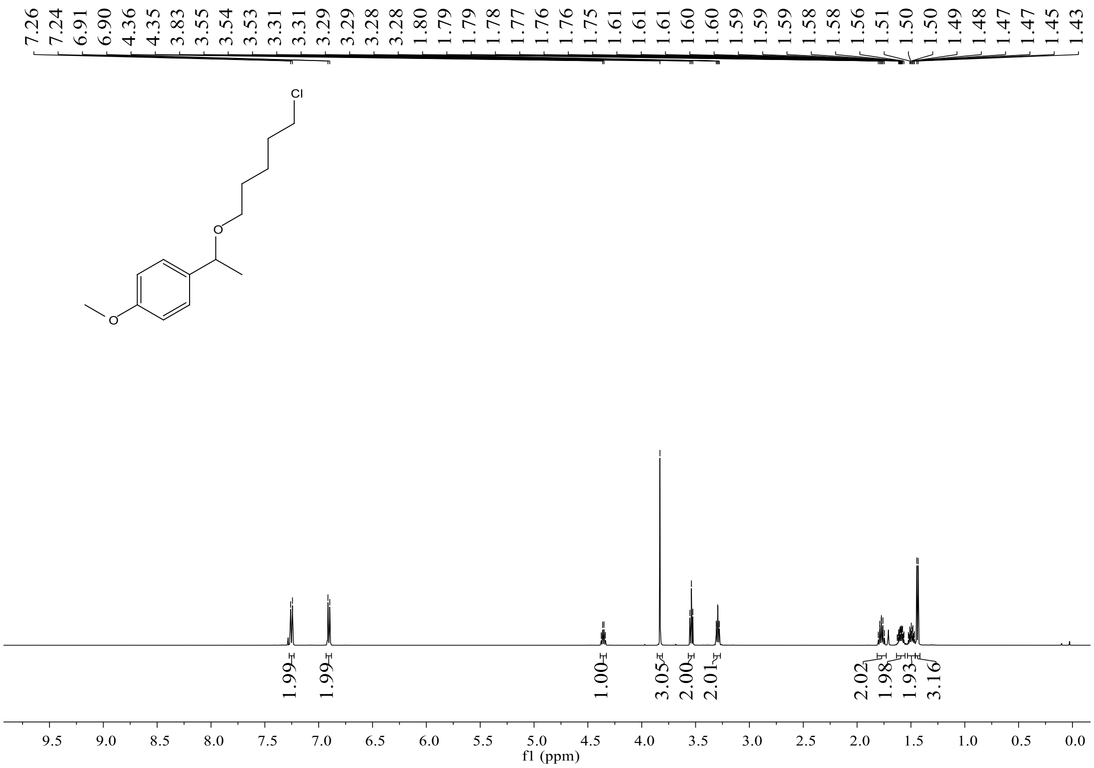

^{1}H NMR spectrum in $CDCl_3$.

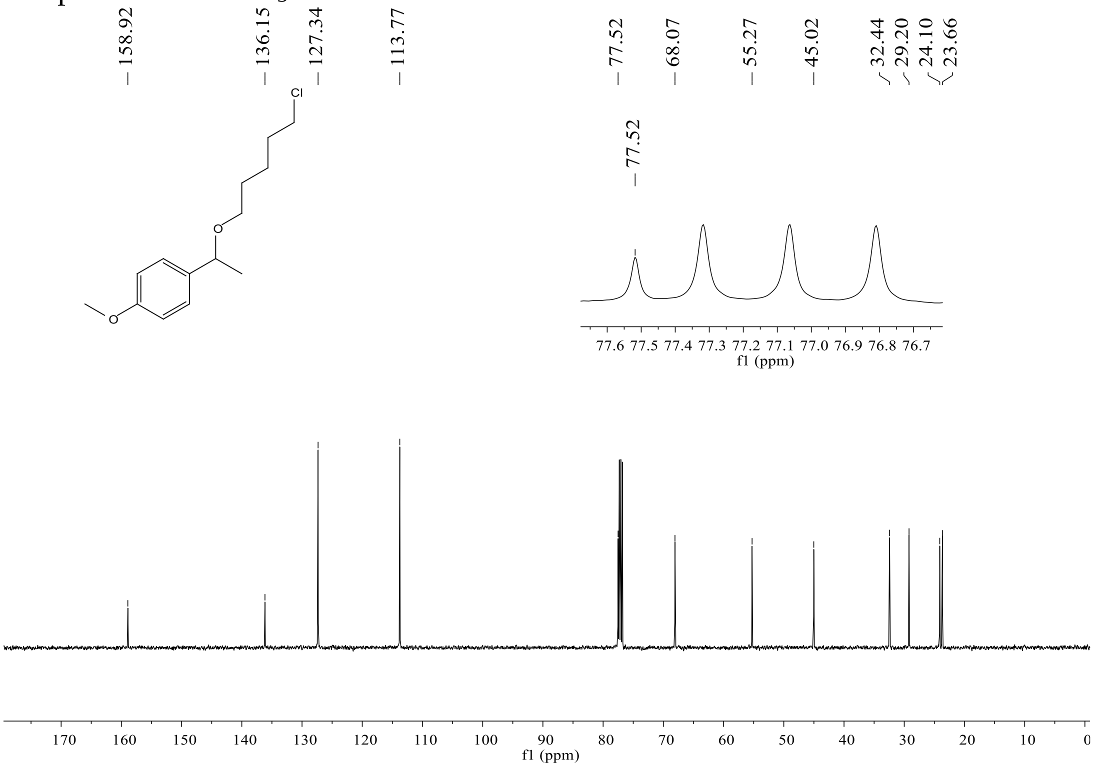

^{13}C NMR spectrum in $CDCl_3$.

34d

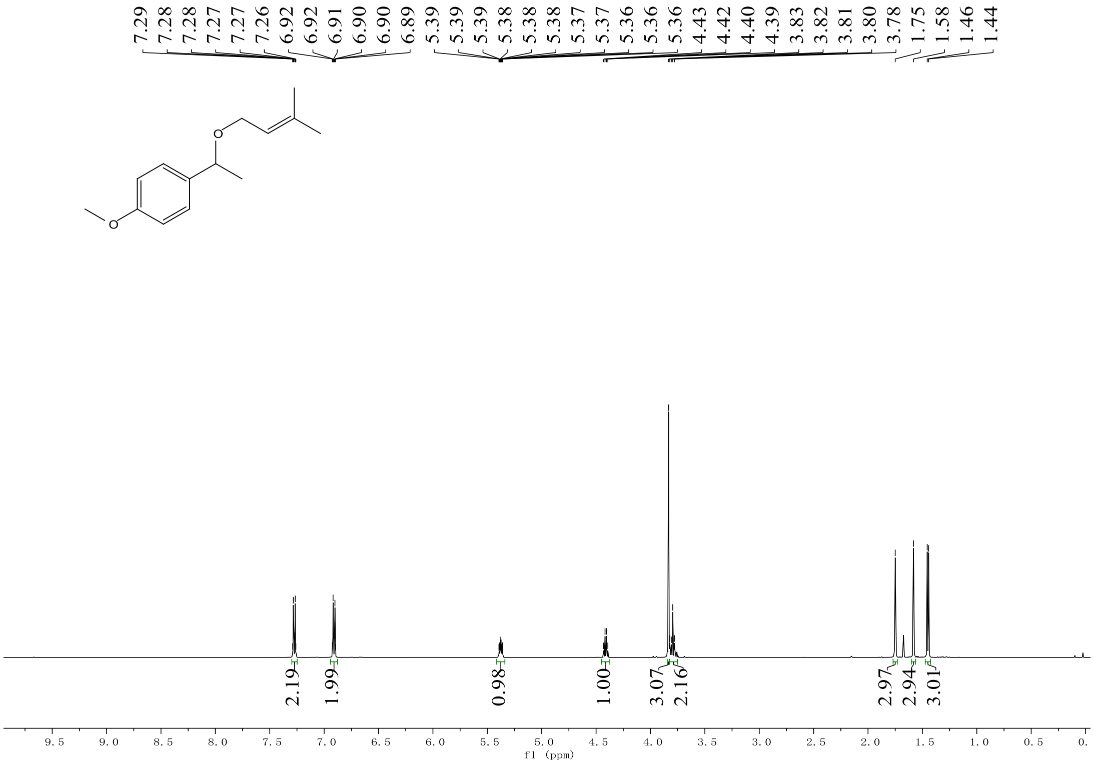

^{1}H NMR spectrum in $CDCl_3$.

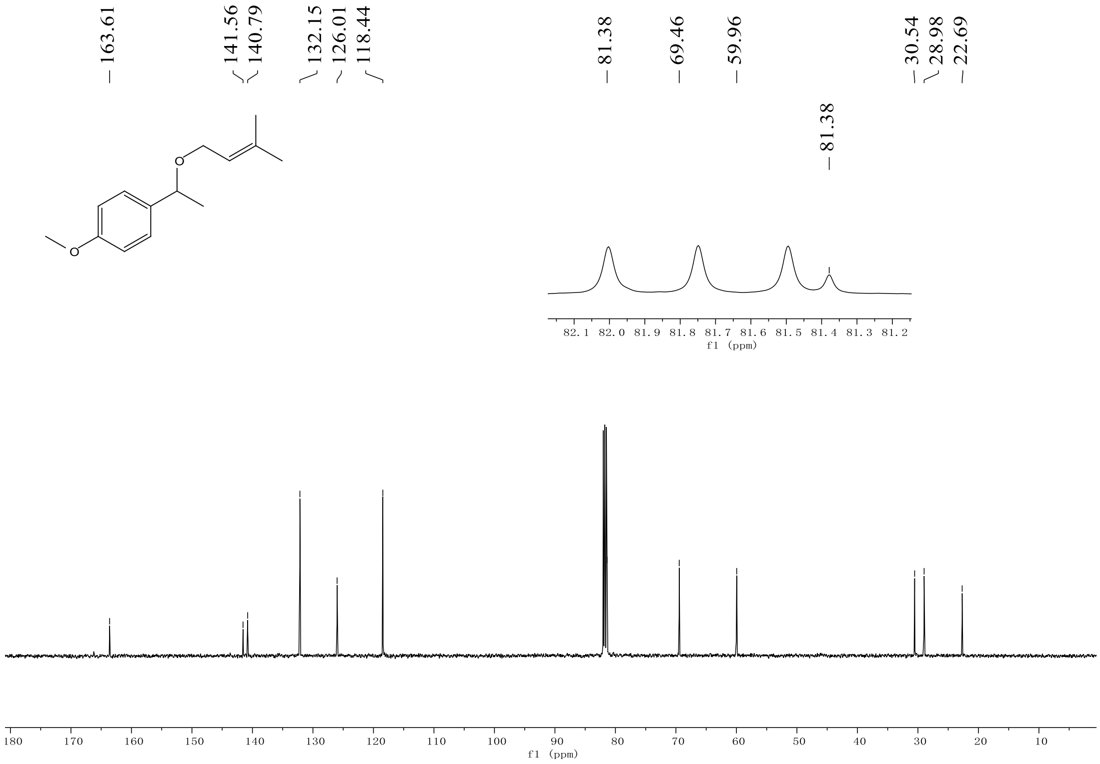

^{13}C NMR spectrum in $CDCl_3$.

35d

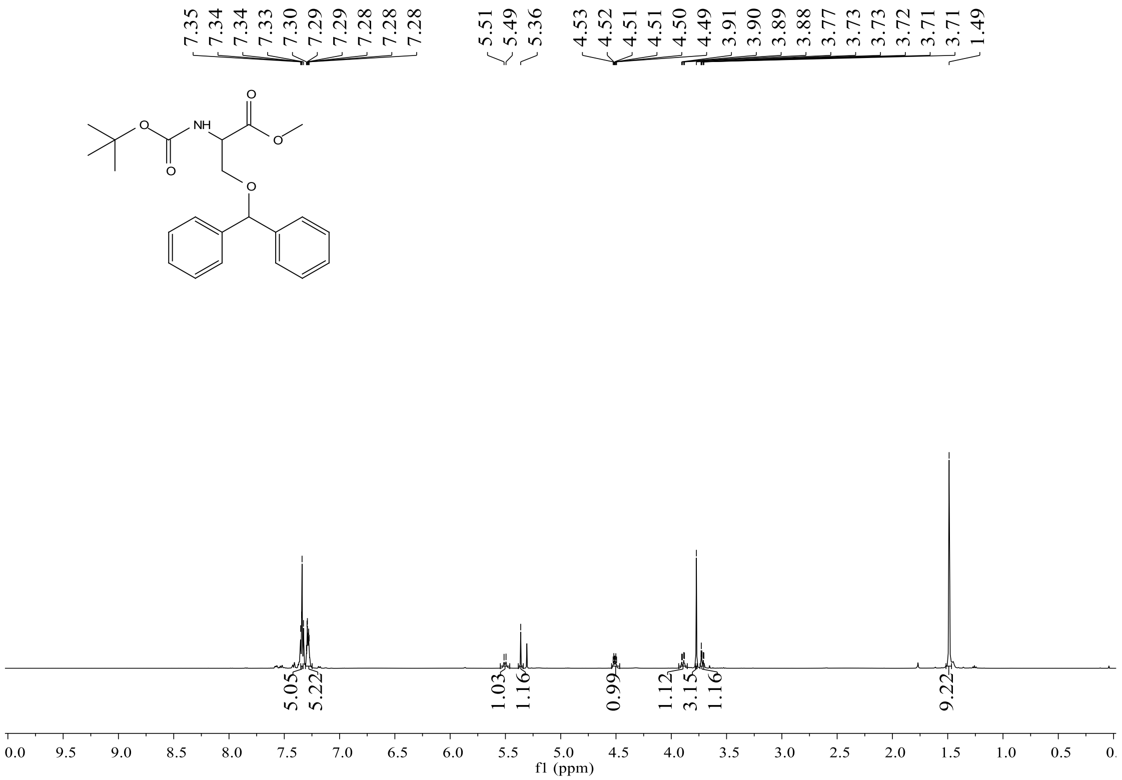

^{1}H NMR spectrum in $CDCl_3$.

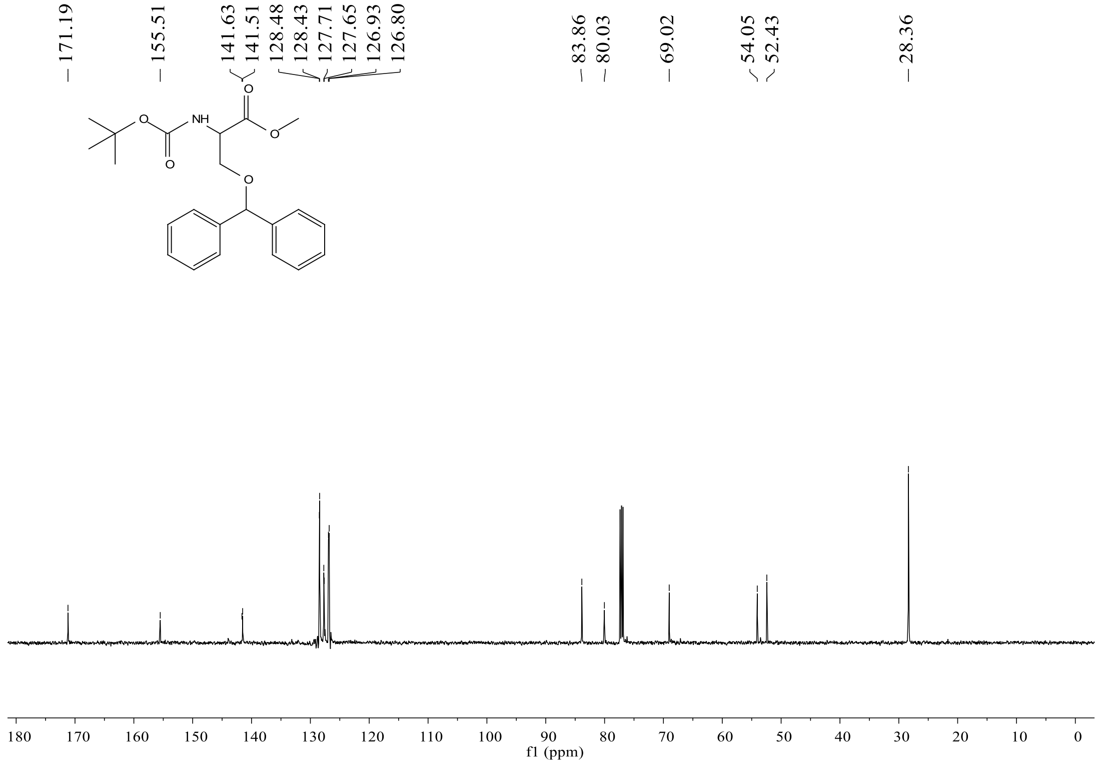

^{13}C NMR spectrum in $CDCl_3$.

36d

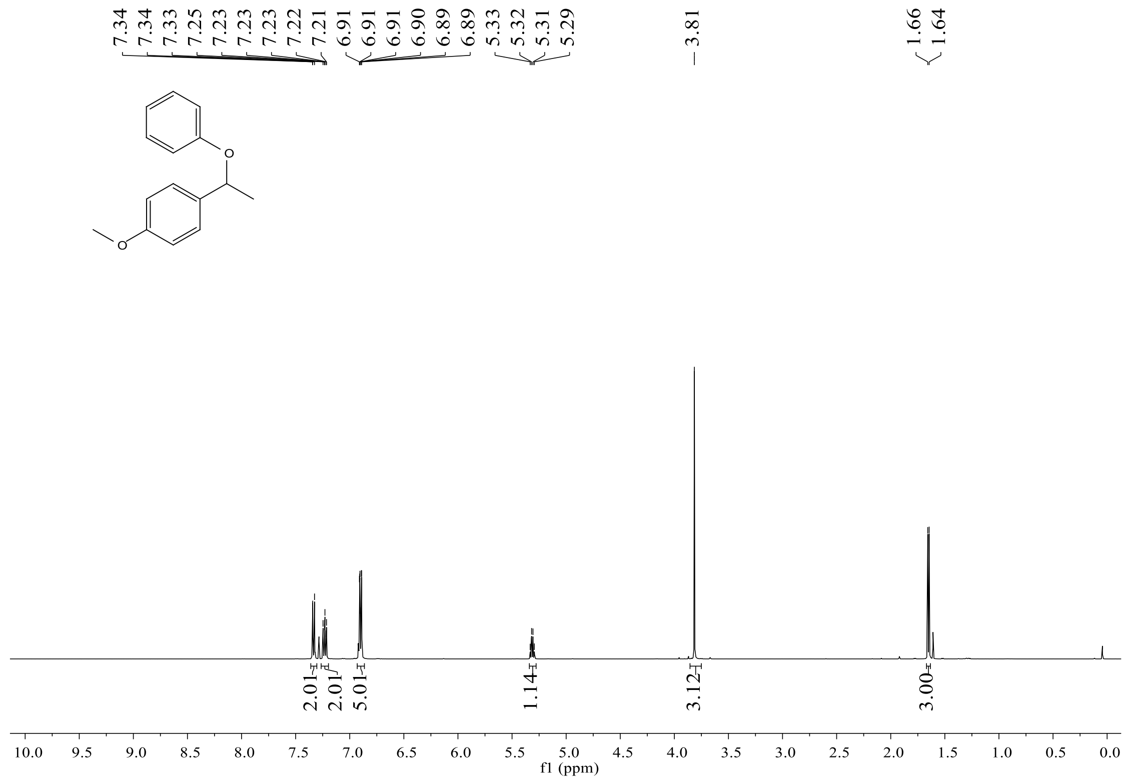

^{1}H NMR spectrum in $CDCl_3$.

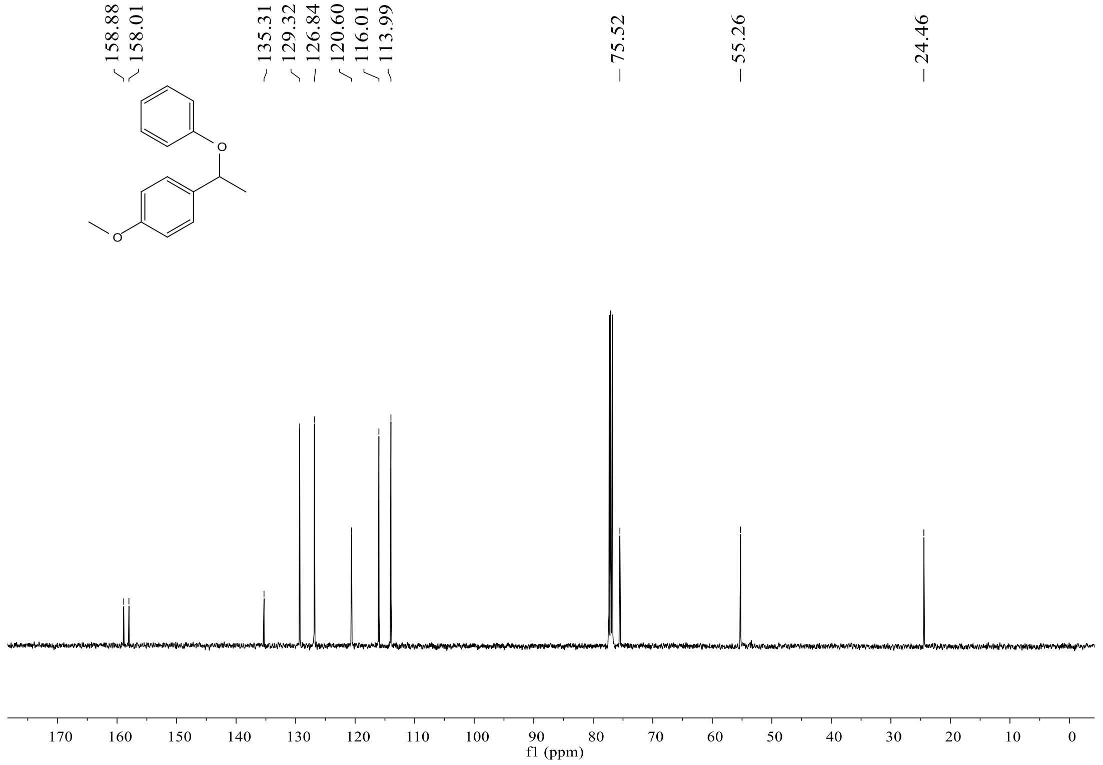

^{13}C NMR spectrum in $CDCl_3$.

37d

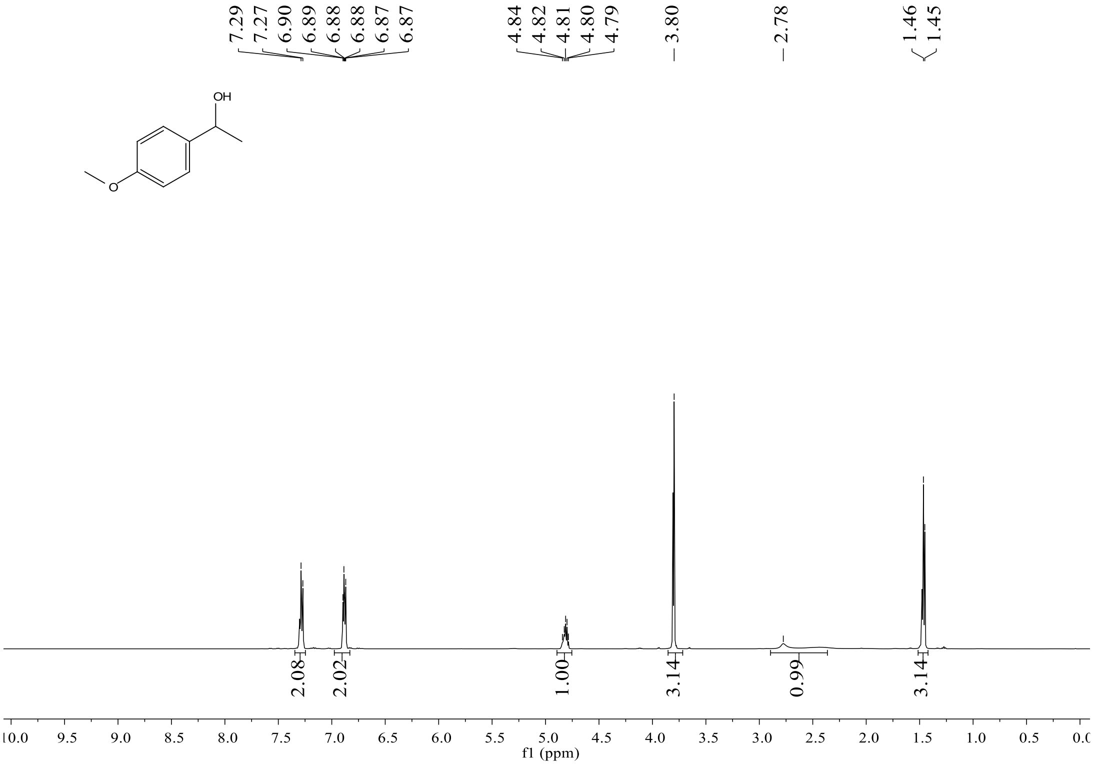

^{1}H NMR spectrum in $CDCl_3$.

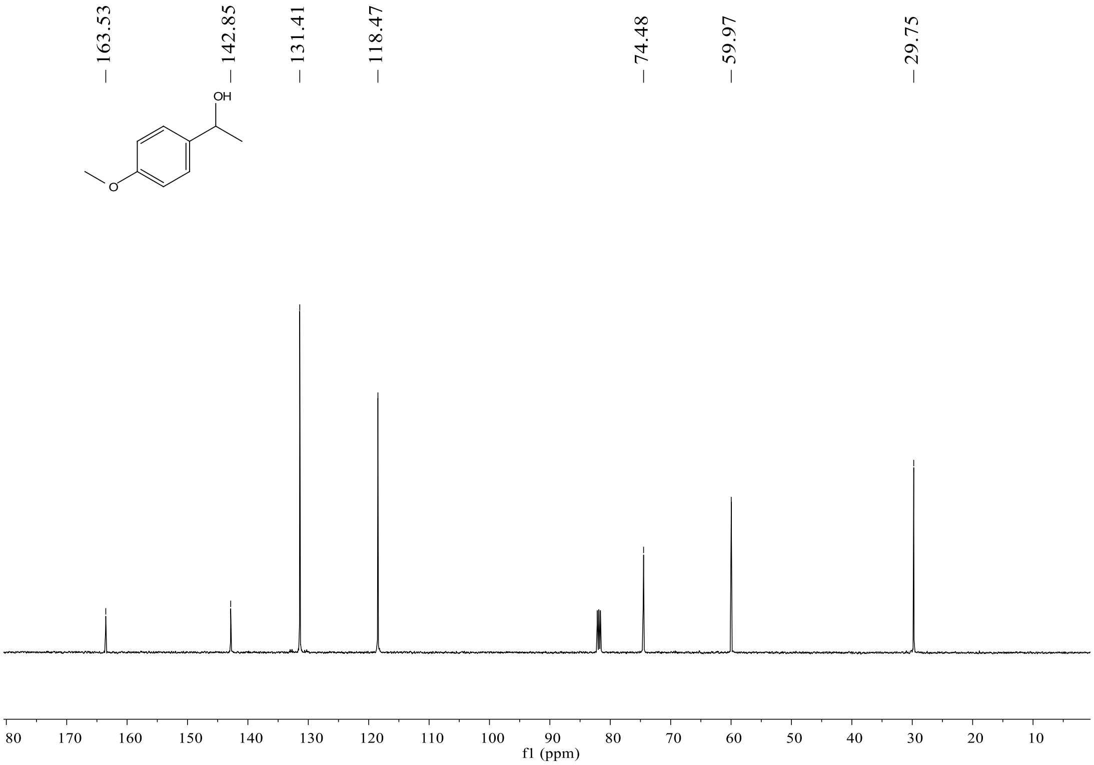

^{13}C NMR spectrum in $CDCl_3$.

38d

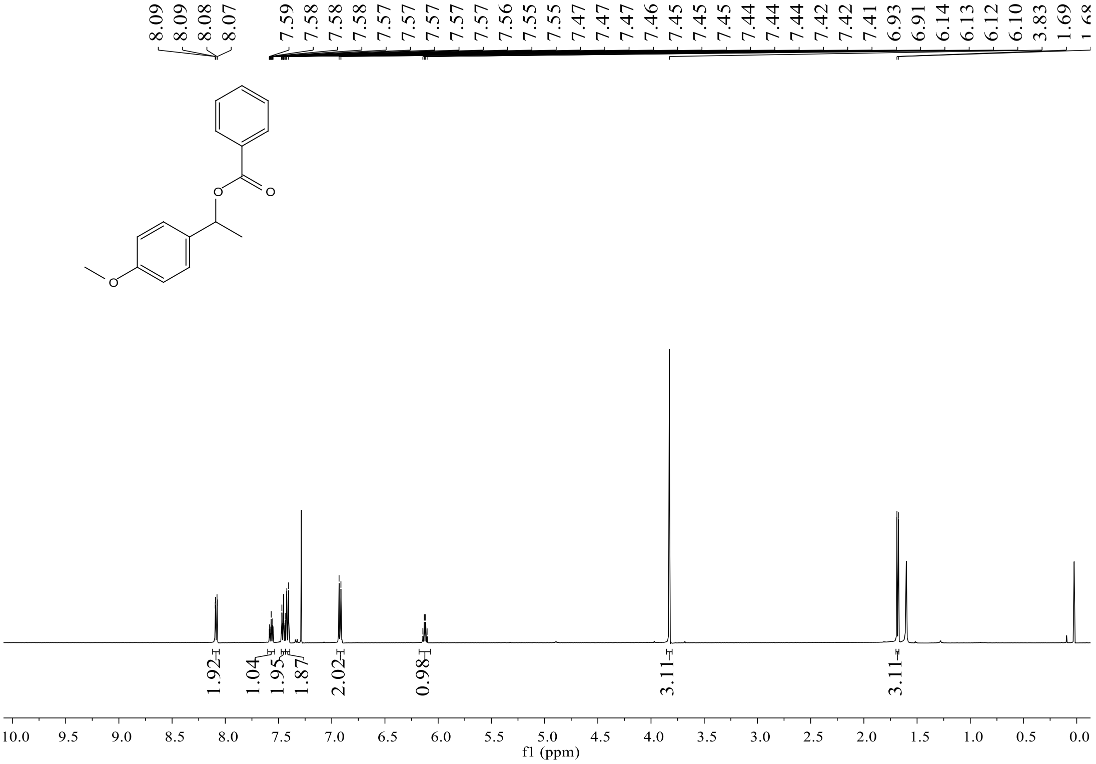

^{1}H NMR spectrum in $CDCl_3$.

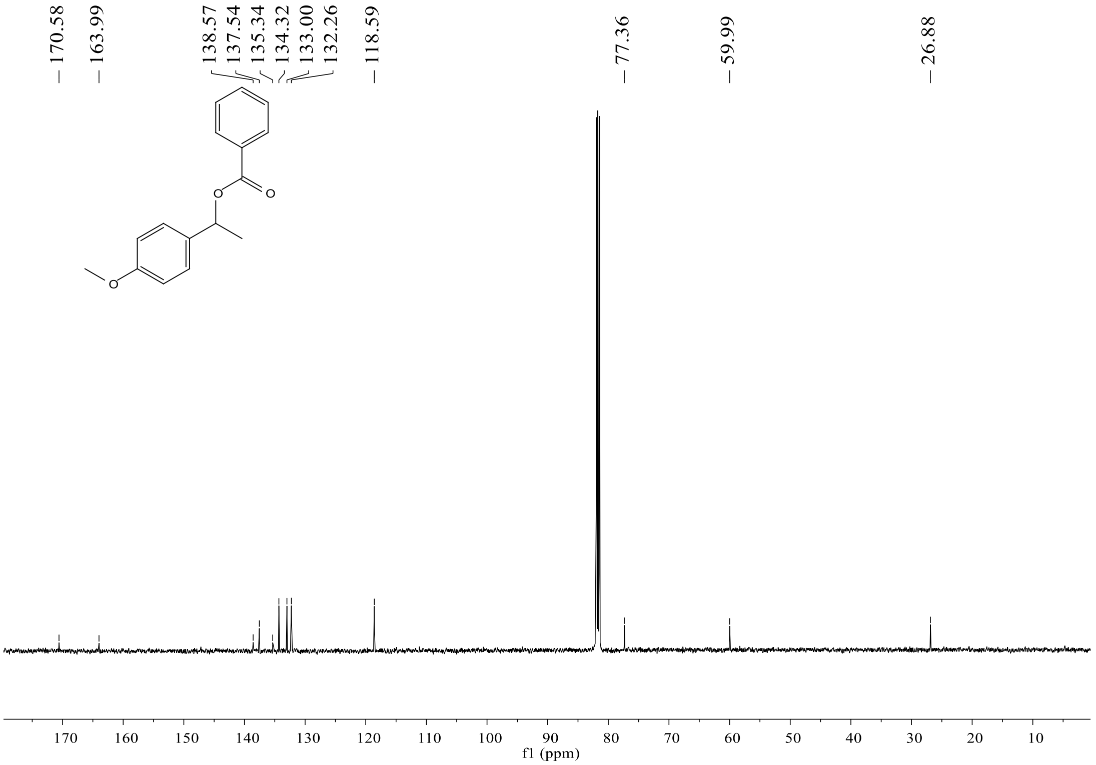

^{13}C NMR spectrum in $CDCl_3$.

39d

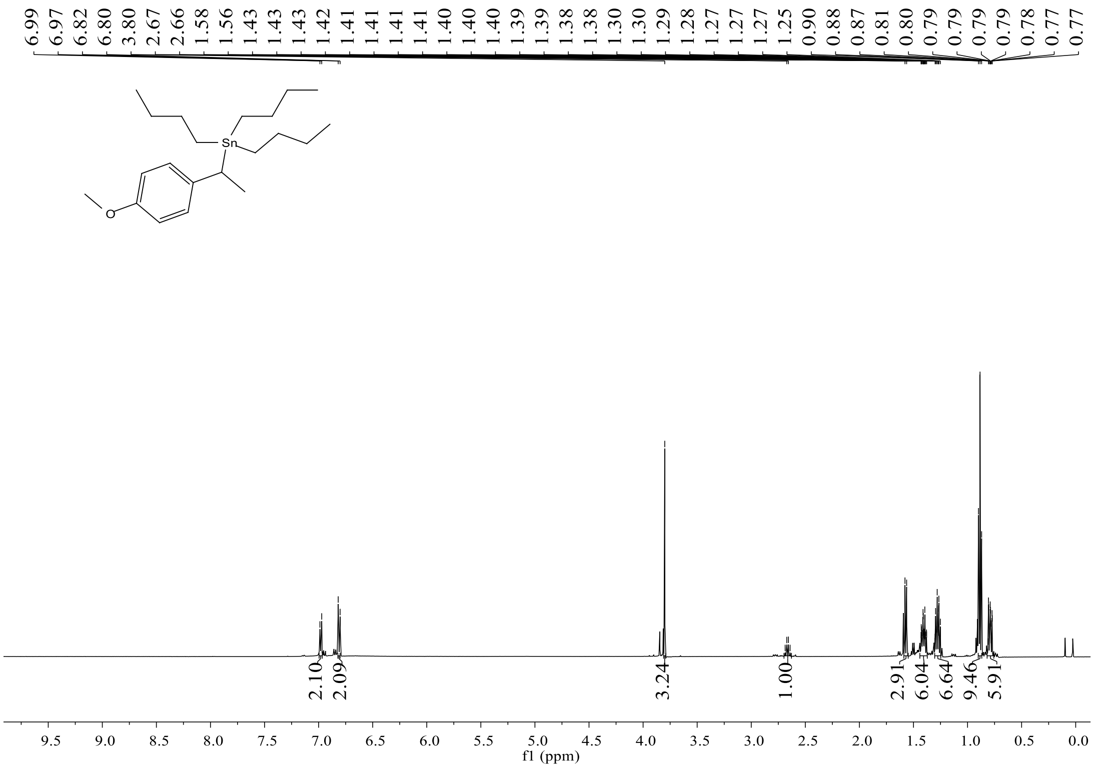

^{1}H NMR spectrum in $CDCl_3$.

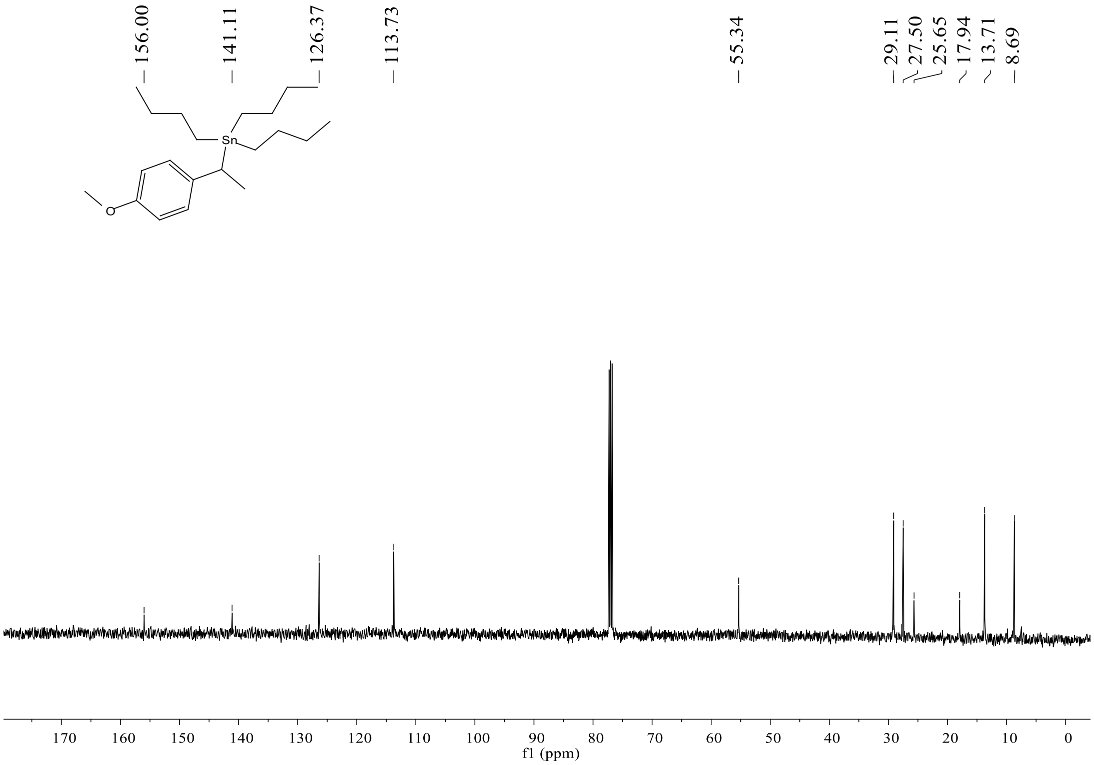

^{13}C NMR spectrum in $CDCl_3$.

40d

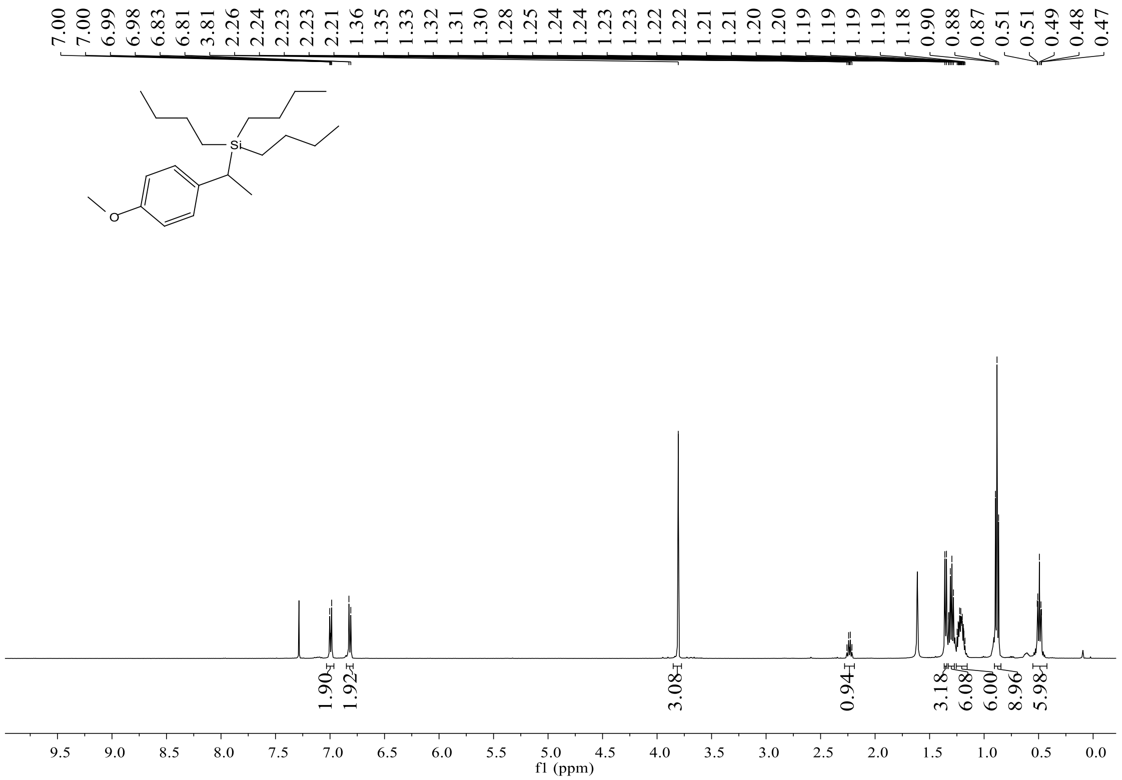

^{1}H NMR spectrum in $CDCl_3$.

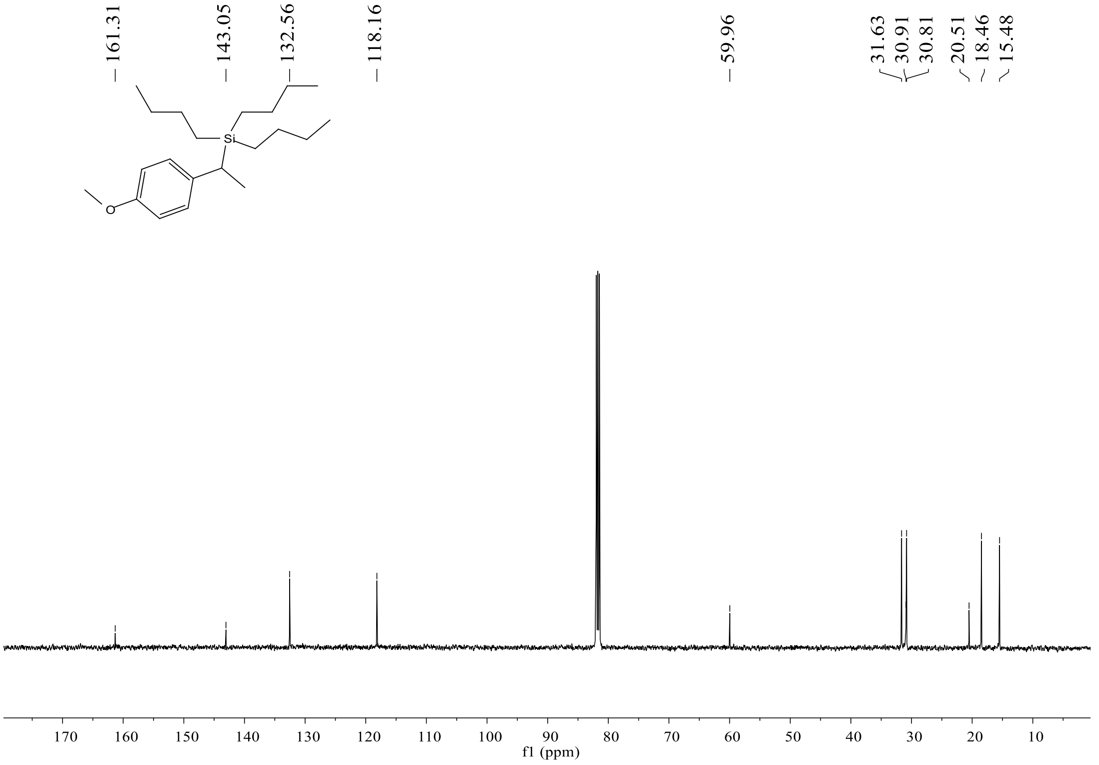

^{13}C NMR spectrum in $CDCl_3$.

41d

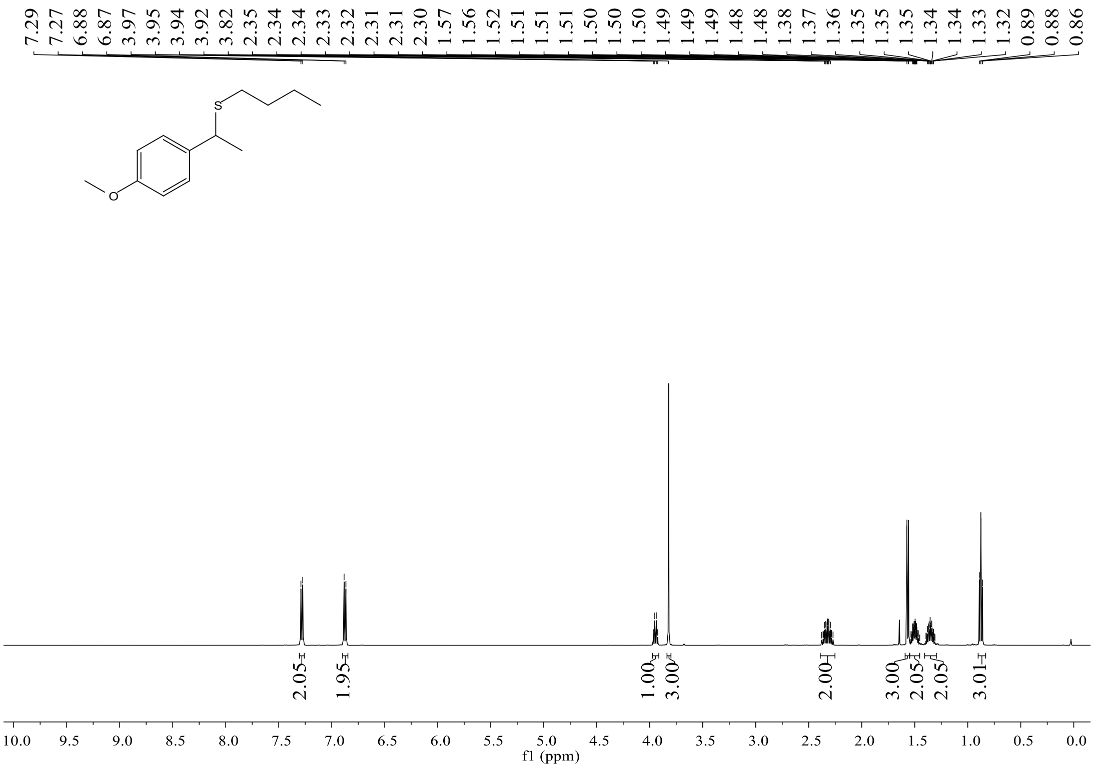

^{1}H NMR spectrum in $CDCl_3$.

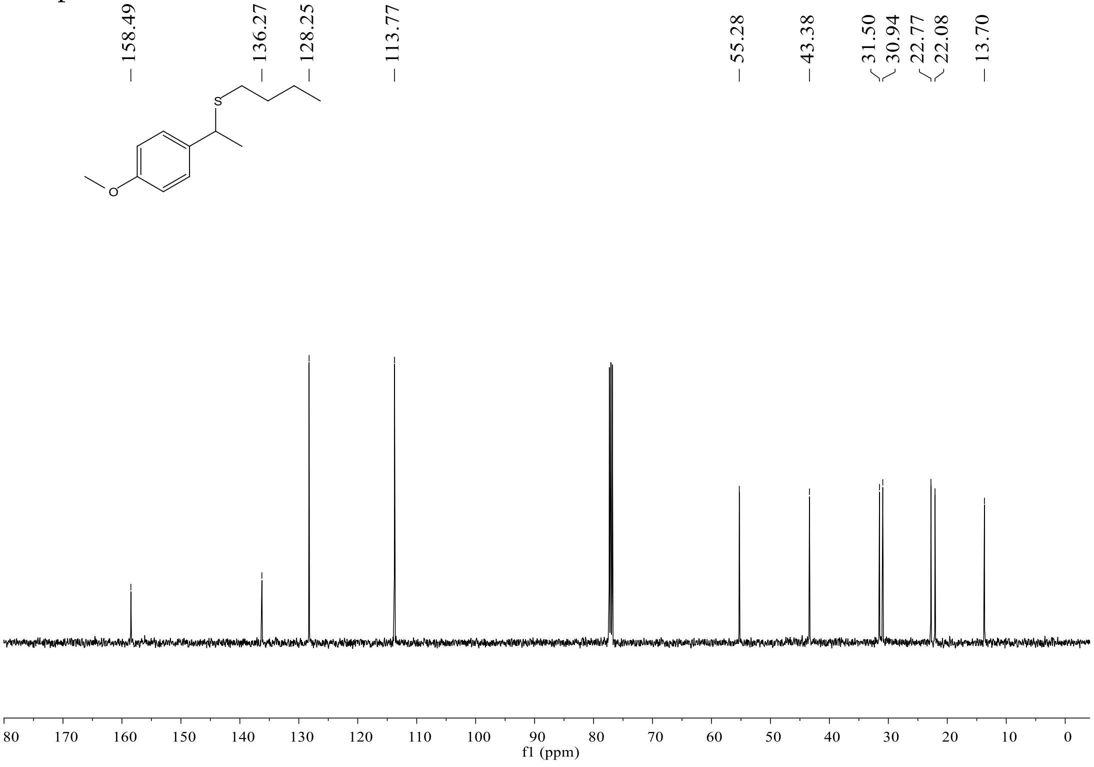

^{13}C NMR spectrum in $CDCl_3$.

42d

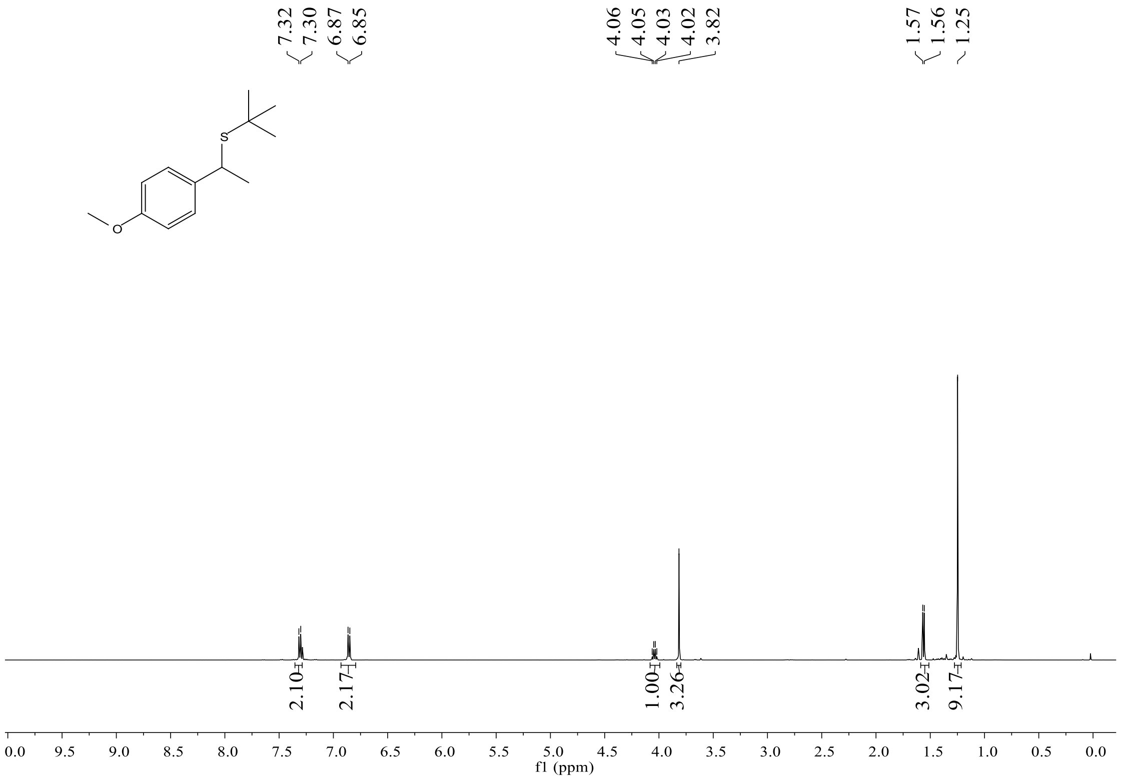

^{1}H NMR spectrum in $CDCl_3$.

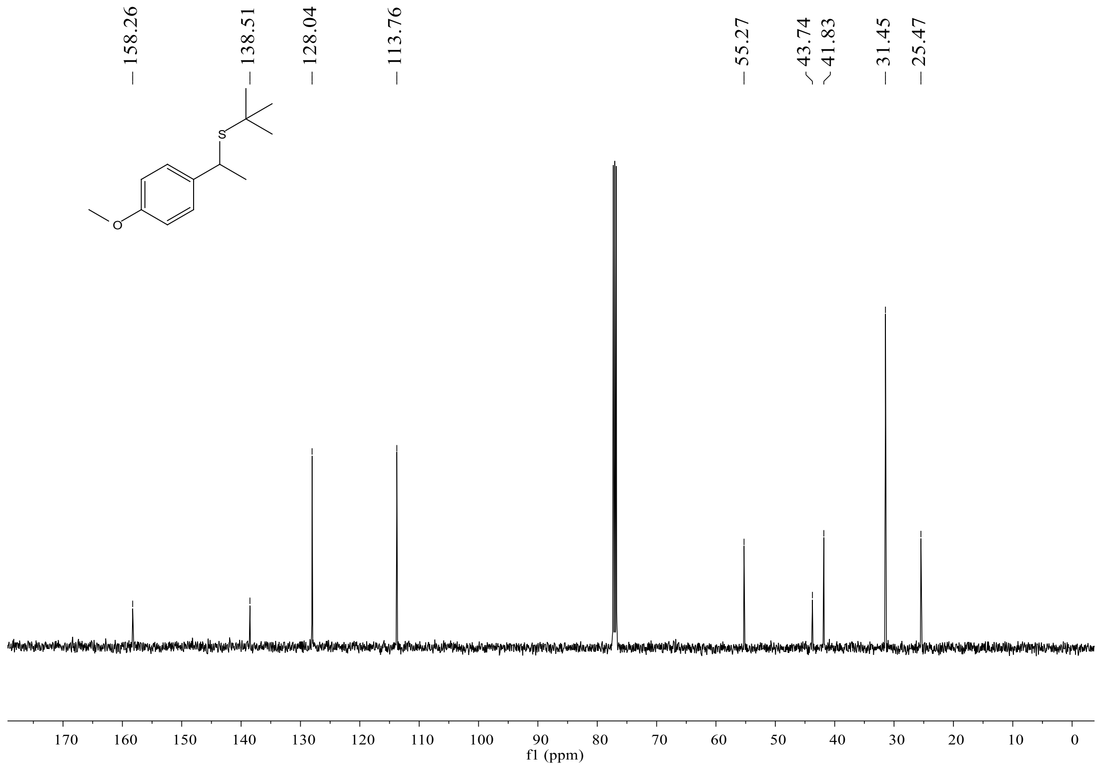

^{13}C NMR spectrum in $CDCl_3$.

43d

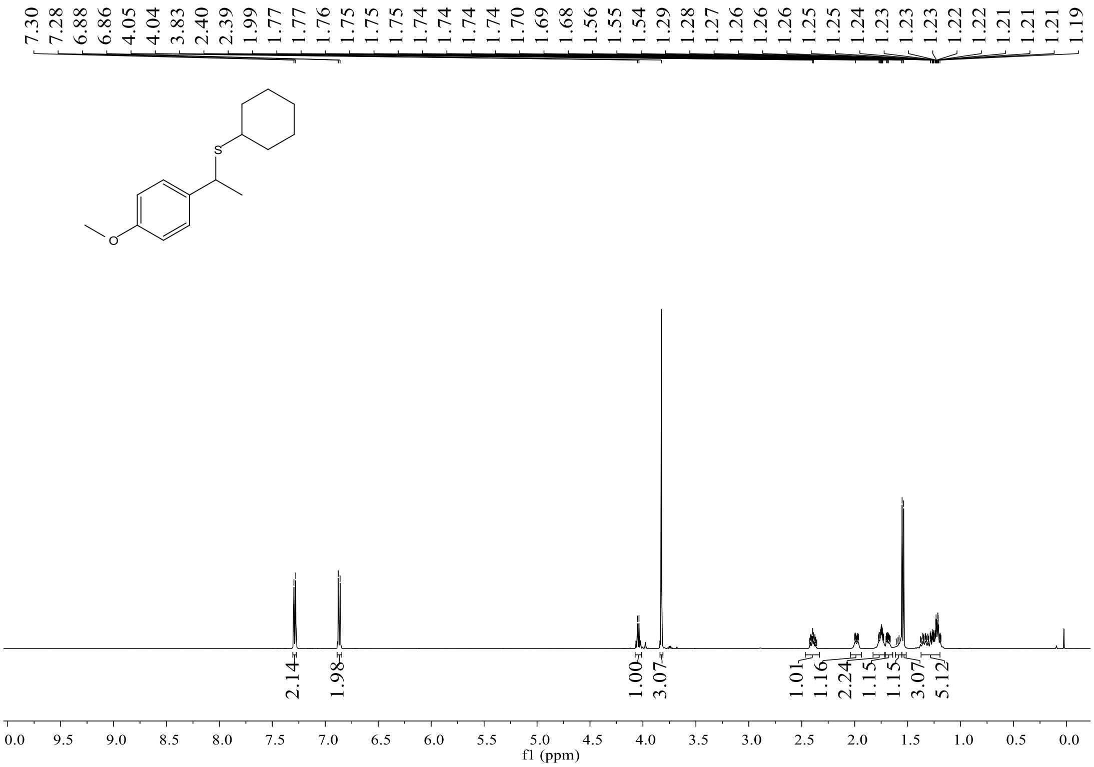

^{1}H NMR spectrum in $CDCl_3$.

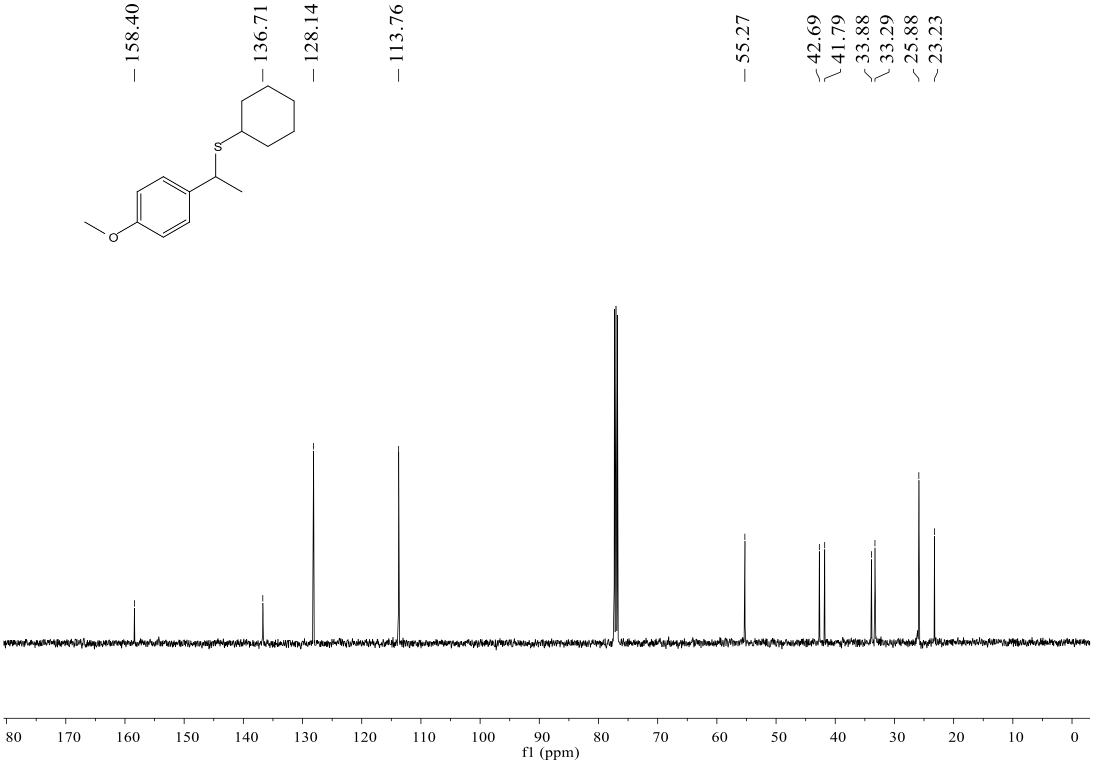

^{13}C NMR spectrum in $CDCl_3$.

44d

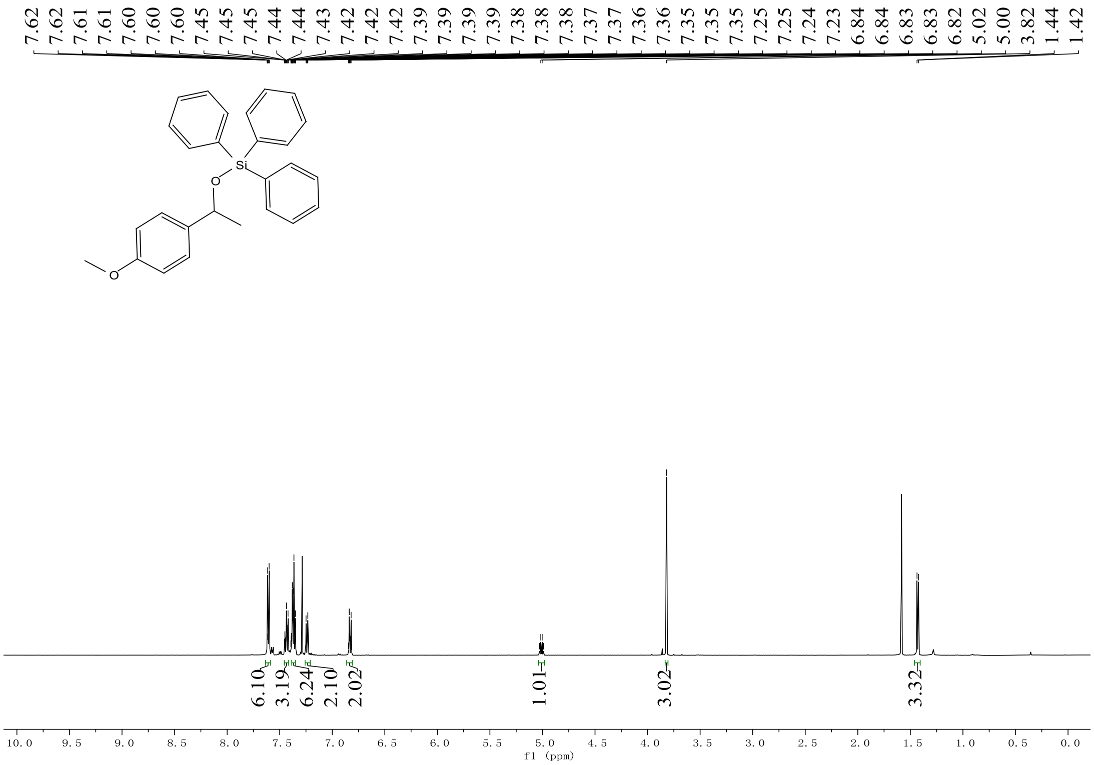

^{1}H NMR spectrum in CDCl$_3$.

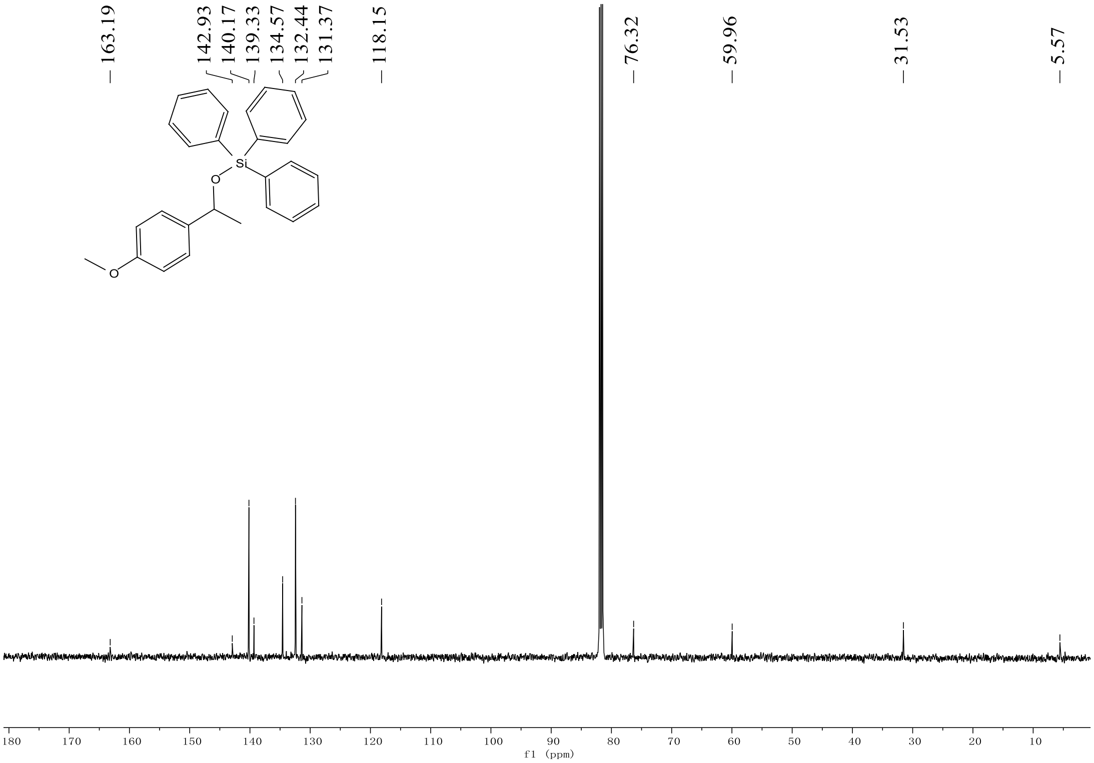

^{13}C NMR spectrum in CDCl$_3$.

45d

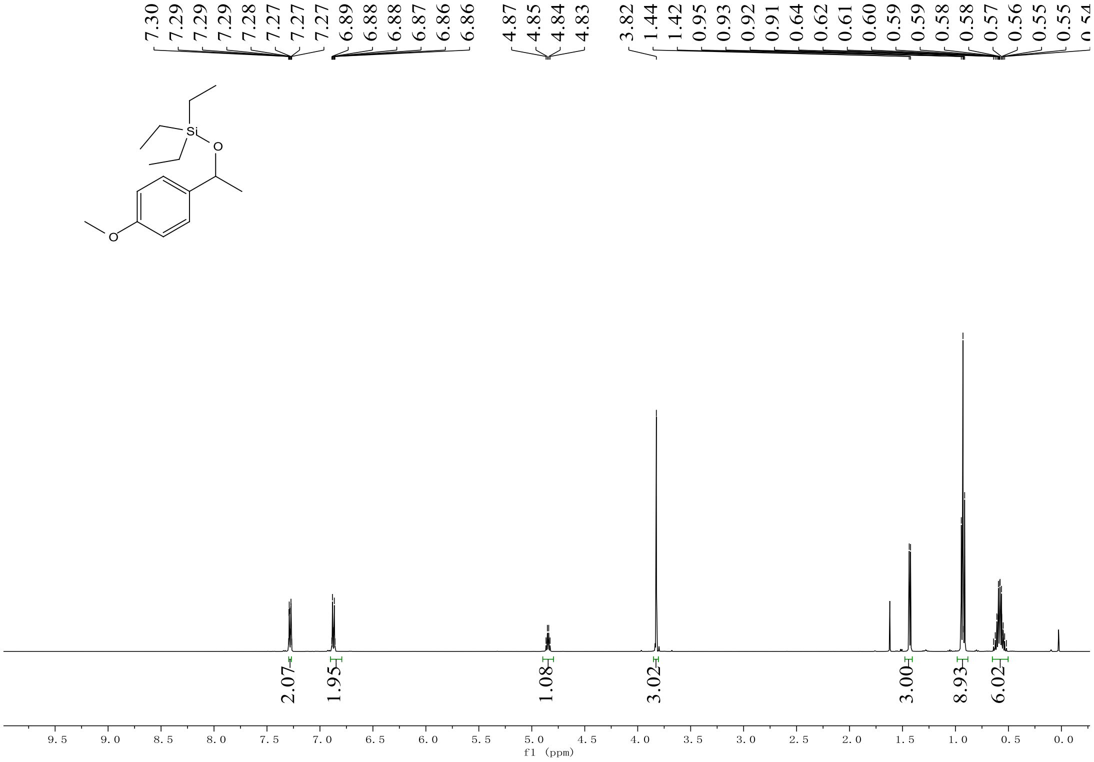

^{1}H NMR spectrum in $CDCl_3$.

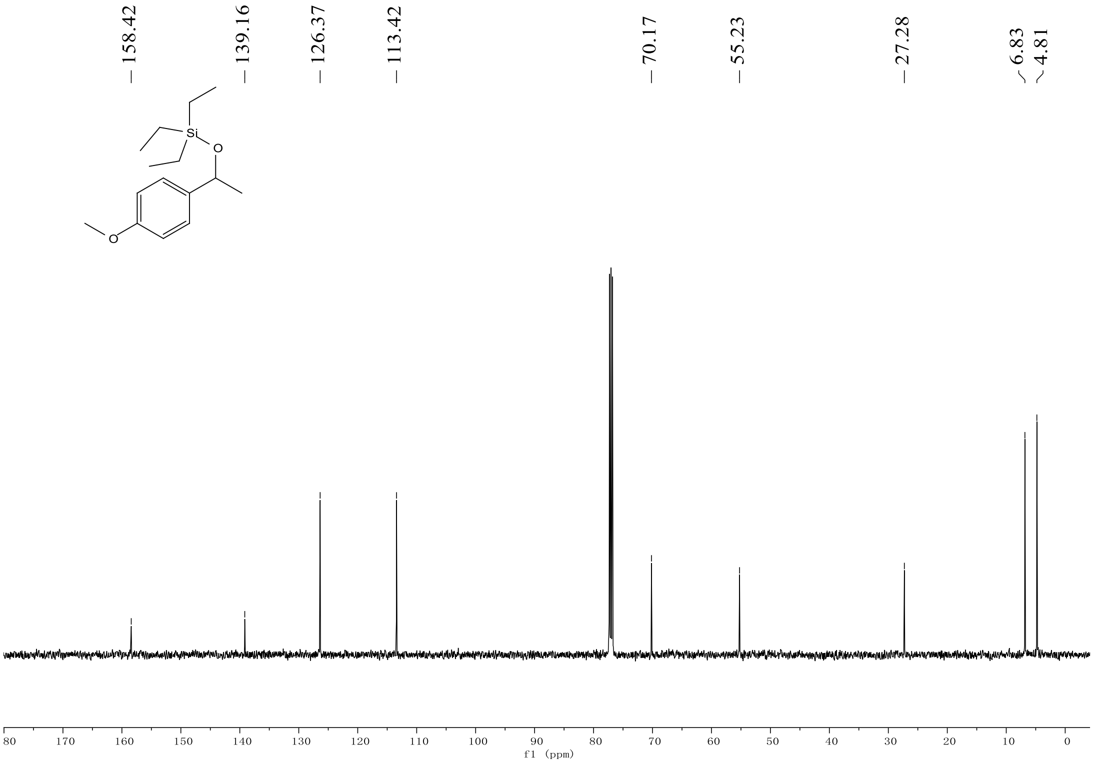

^{13}C NMR spectrum in $CDCl_3$.

46d

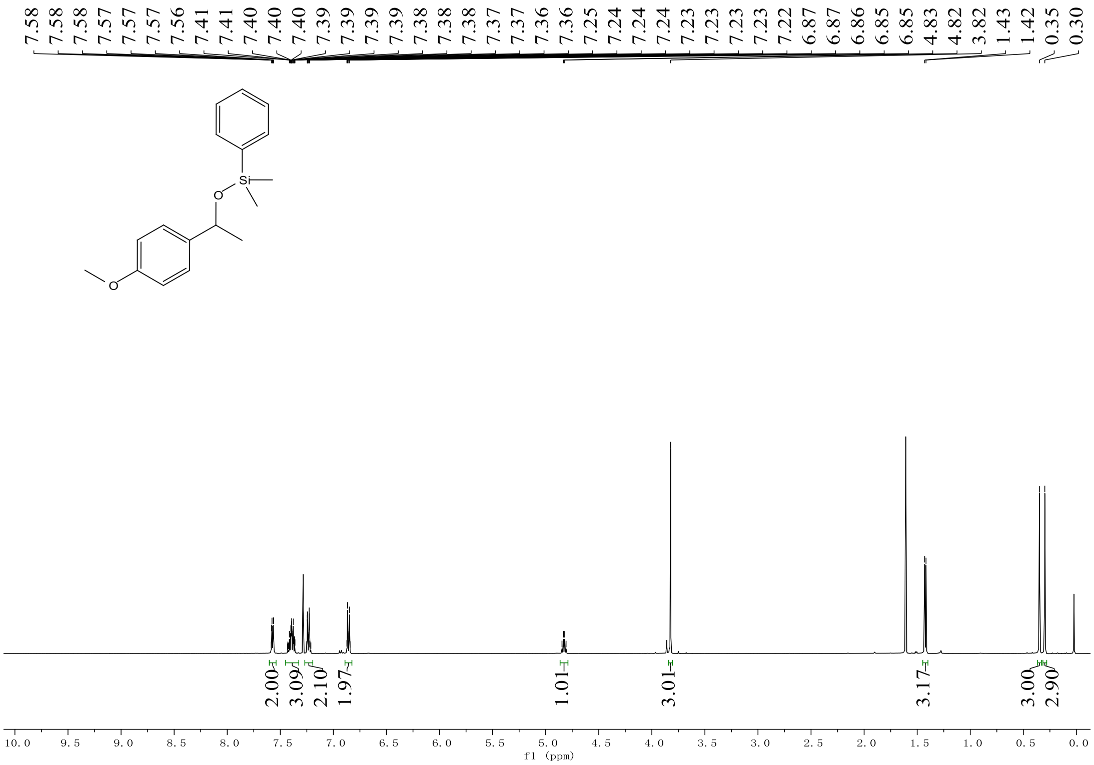

^{1}H NMR spectrum in $CDCl_3$.

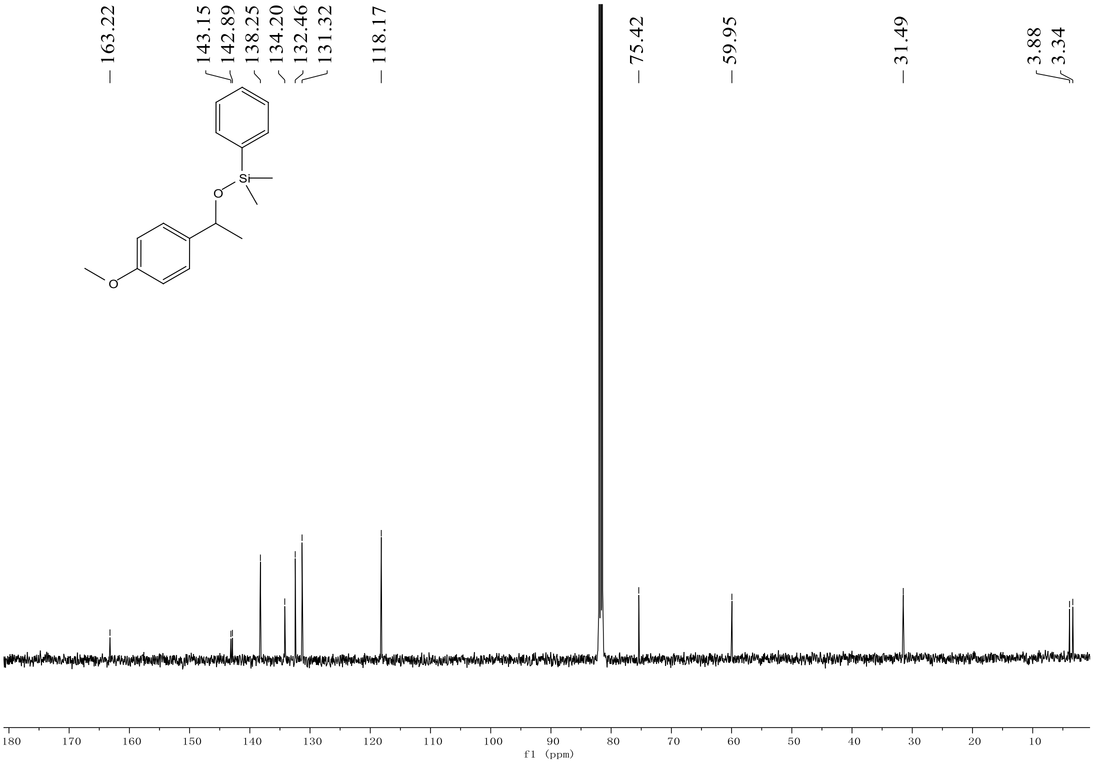

^{13}C NMR spectrum in $CDCl_3$.

47d

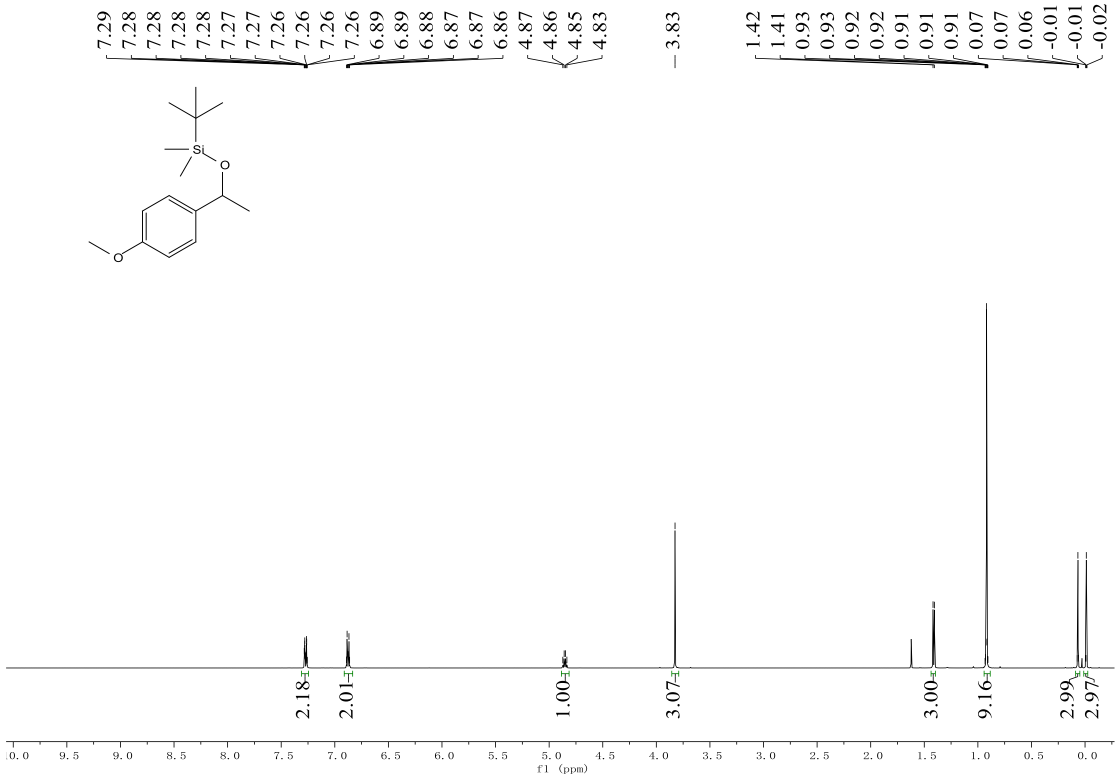

^{1}H NMR spectrum in $CDCl_3$.

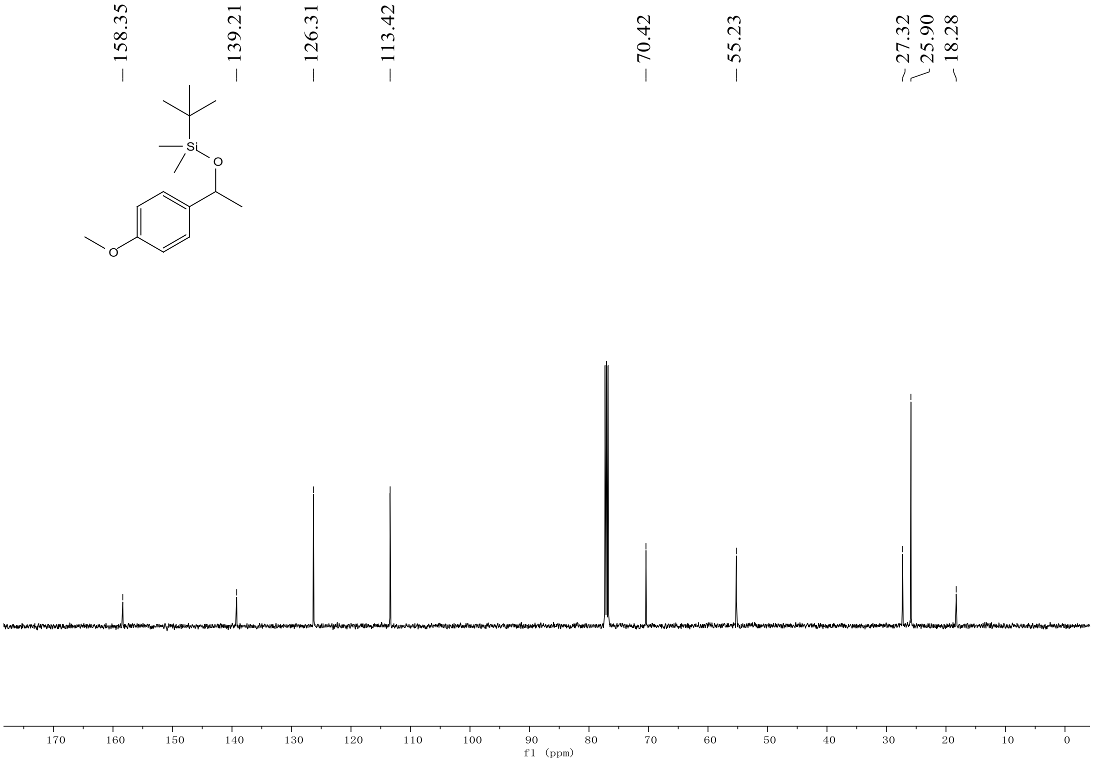

^{13}C NMR spectrum in $CDCl_3$.

48d

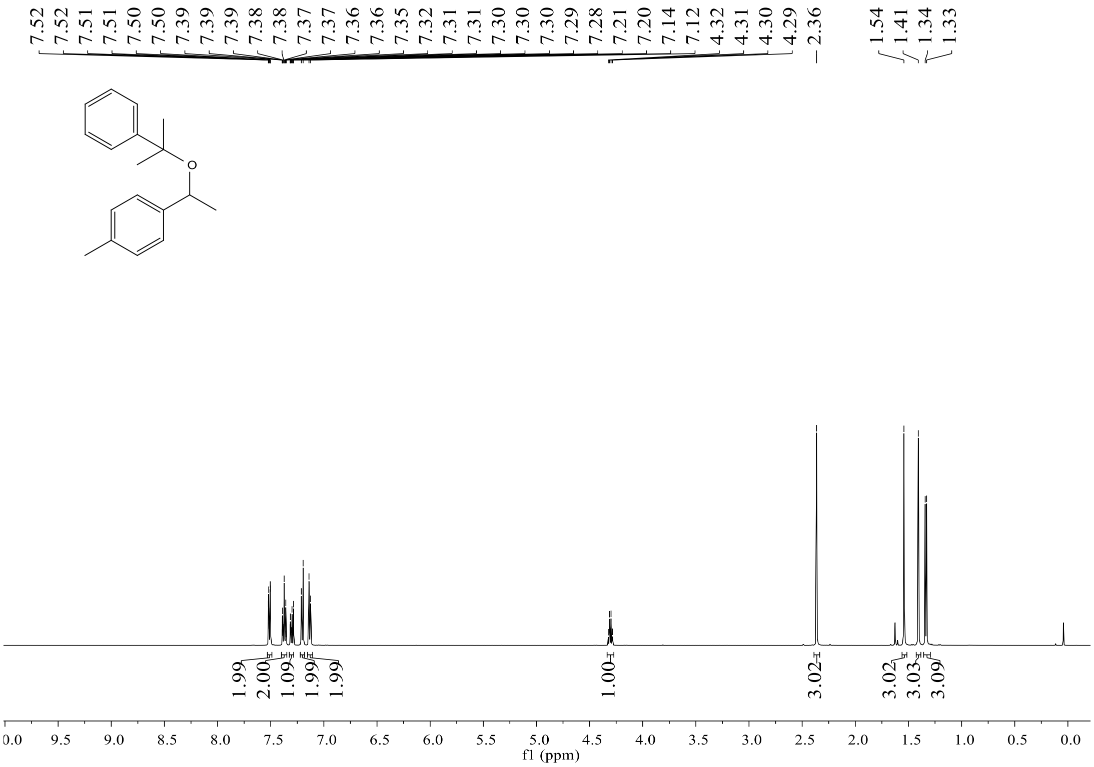

^{1}H NMR spectrum in $CDCl_3$.

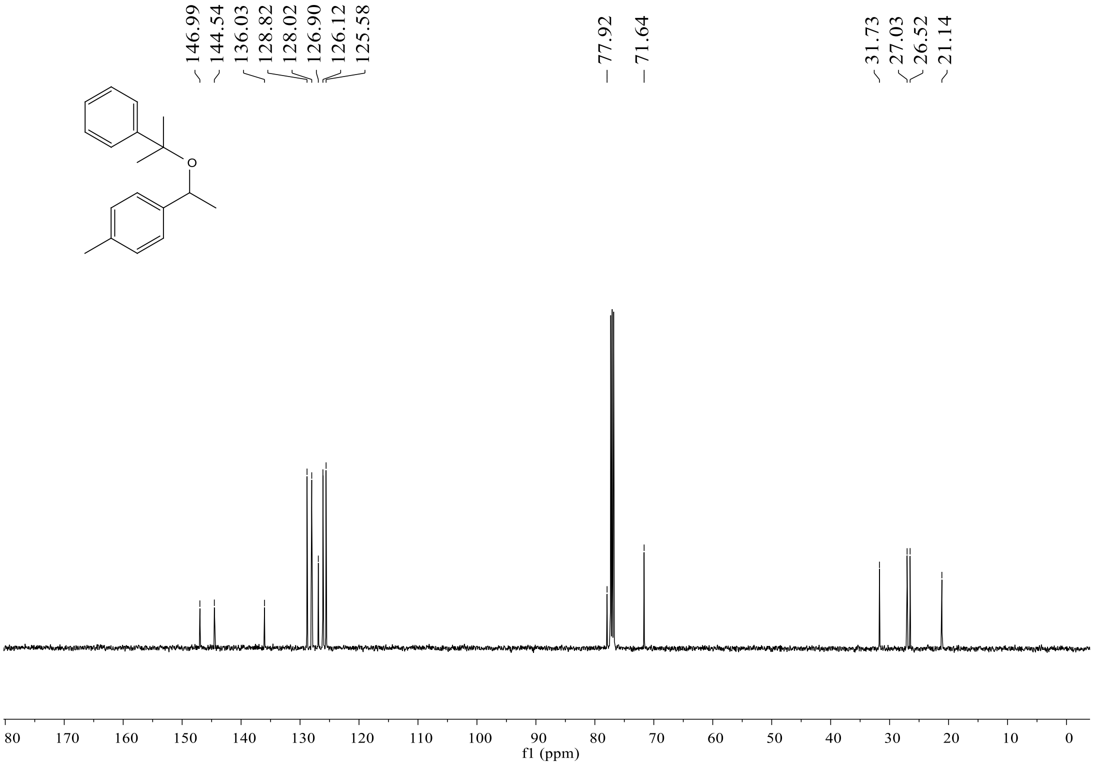

^{13}C NMR spectrum in $CDCl_3$.

49d

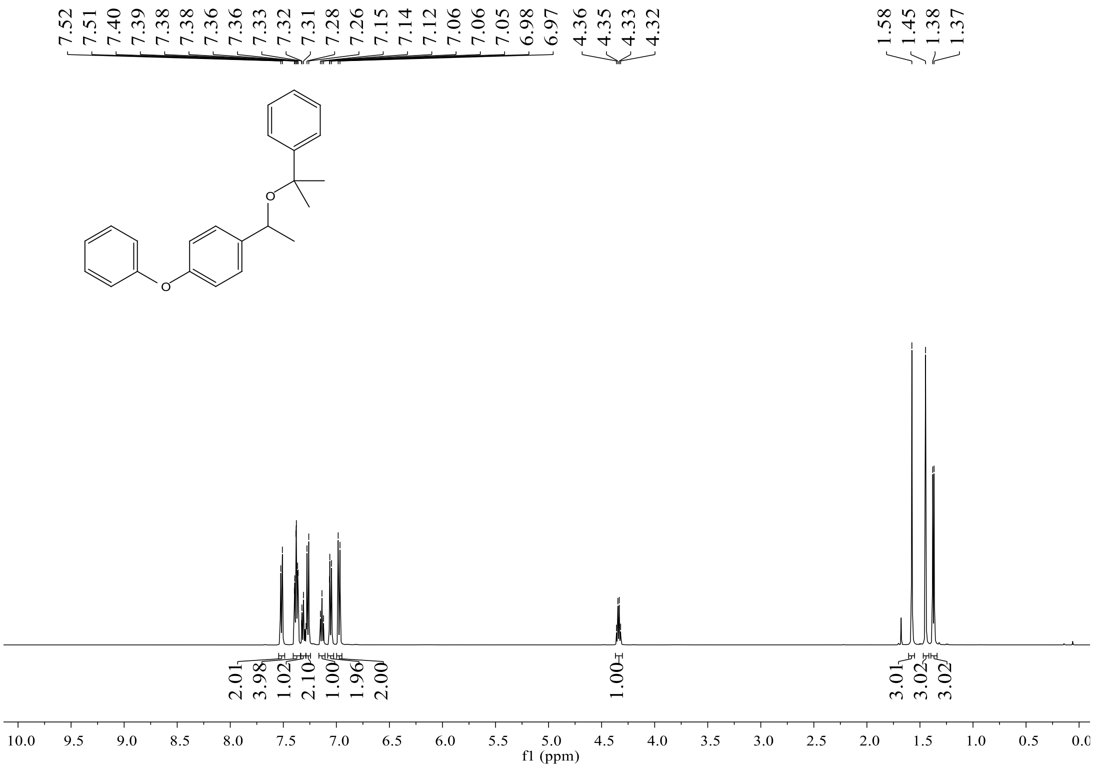

^{1}H NMR spectrum in $CDCl_3$.

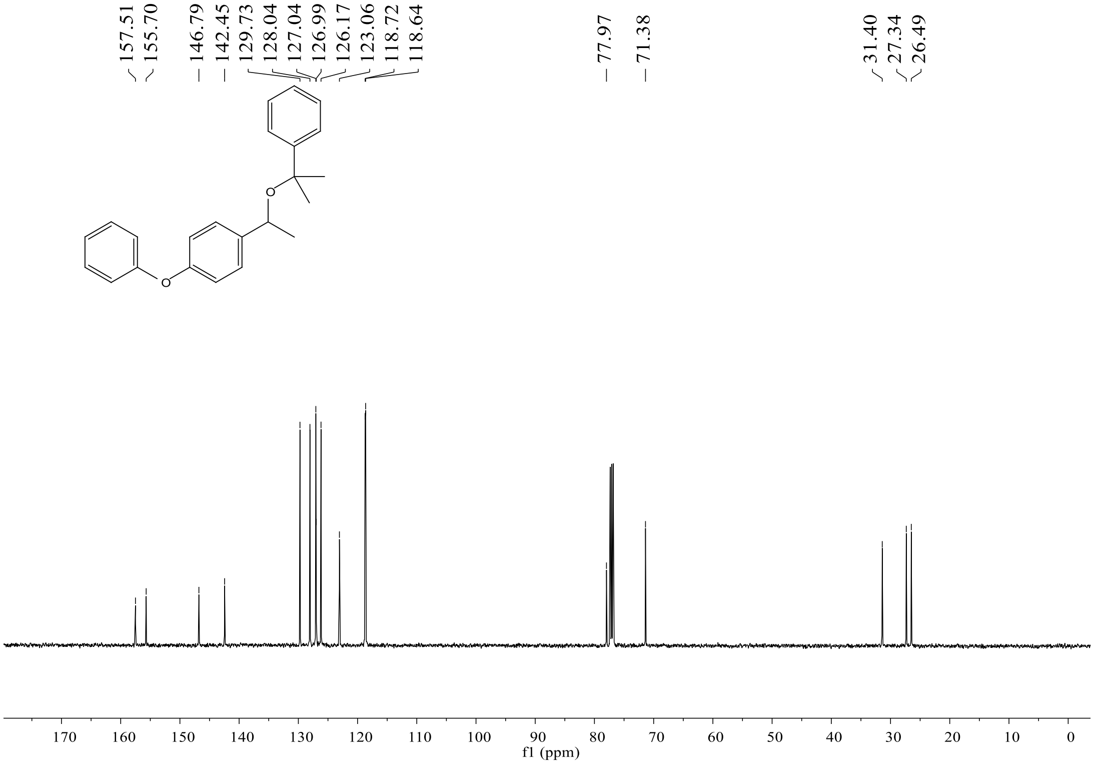

^{13}C NMR spectrum in $CDCl_3$.

50d

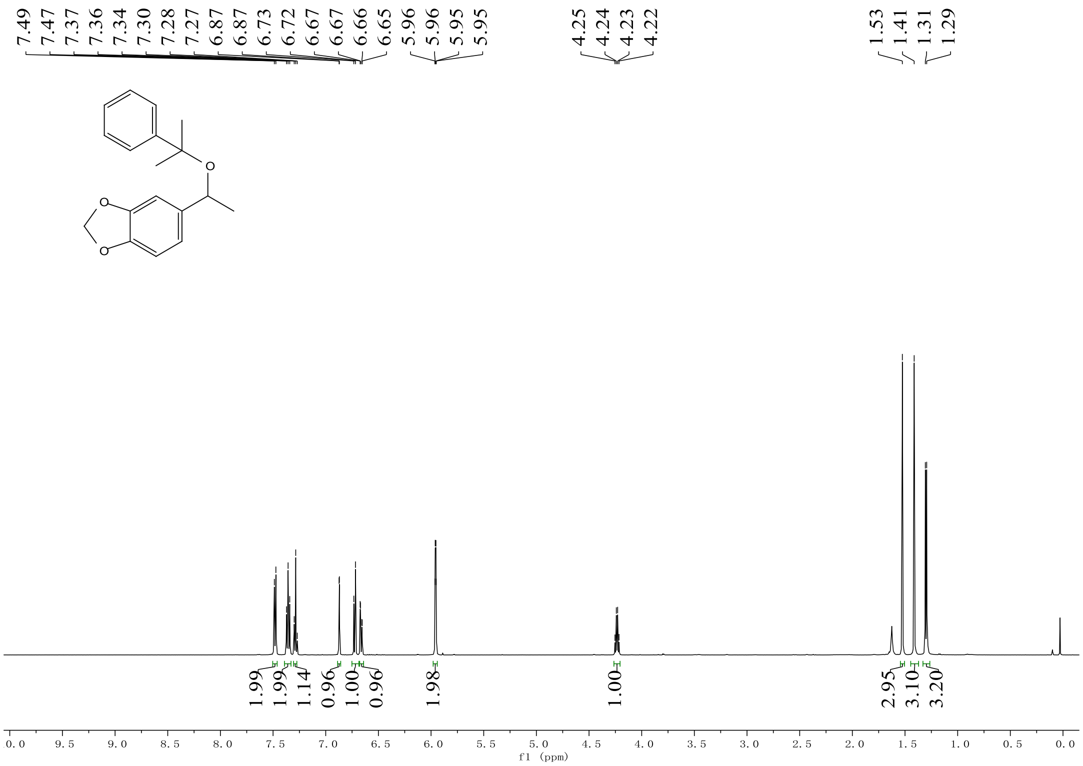

^{1}H NMR spectrum in $CDCl_3$.

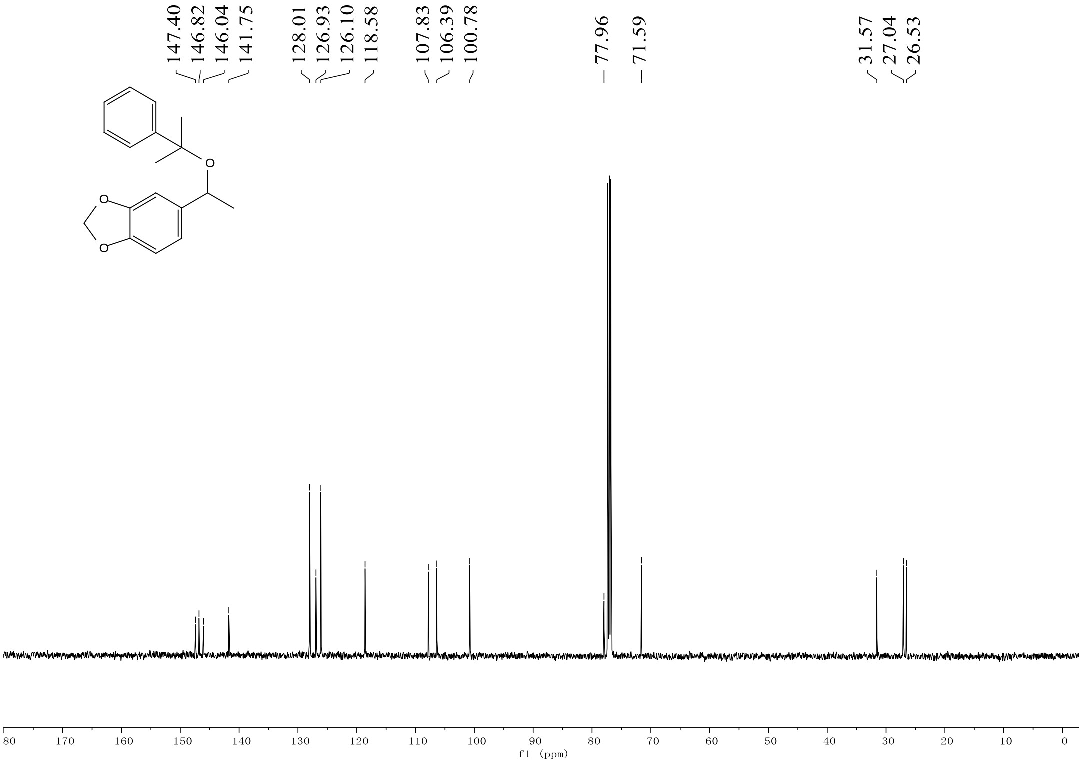

^{13}C NMR spectrum in $CDCl_3$.

51d

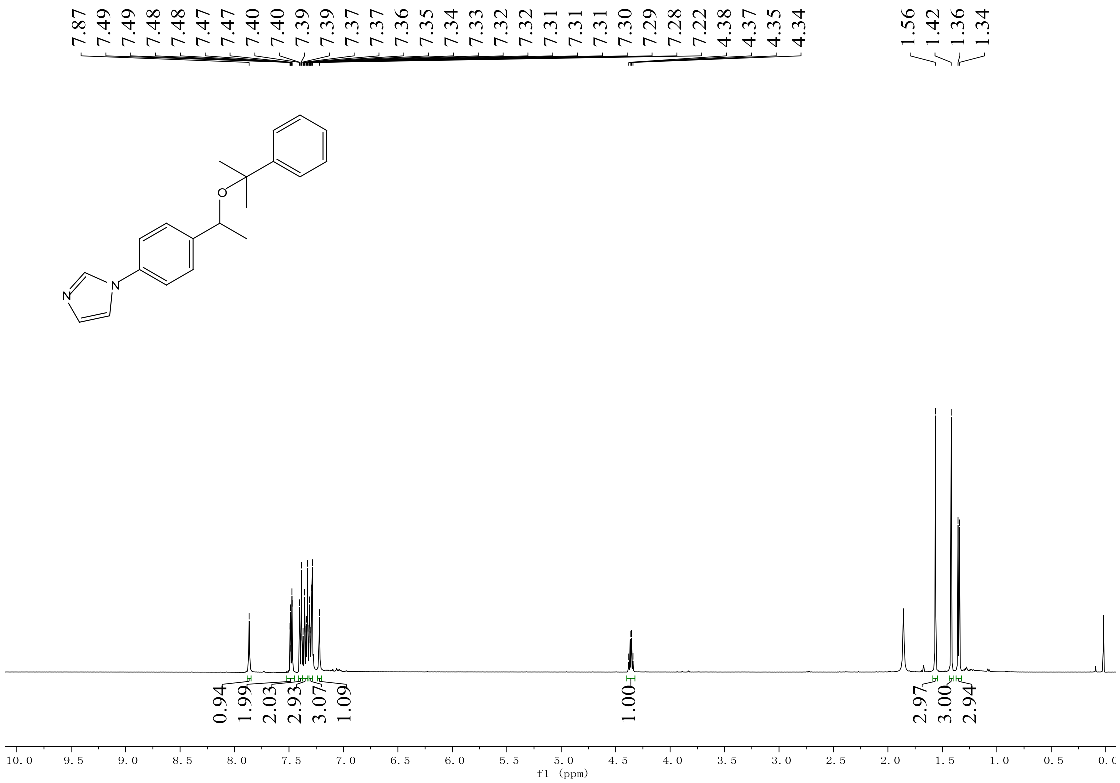

^{1}H NMR spectrum in $CDCl_3$.

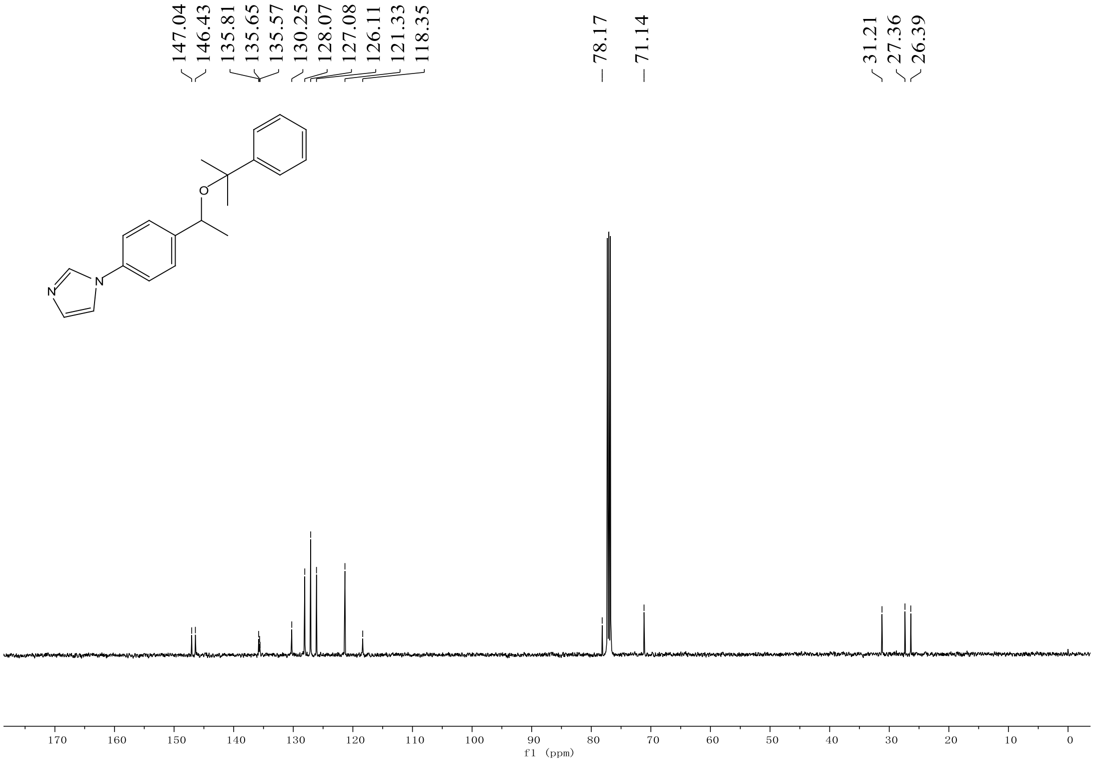

^{13}C NMR spectrum in $CDCl_3$.

52d

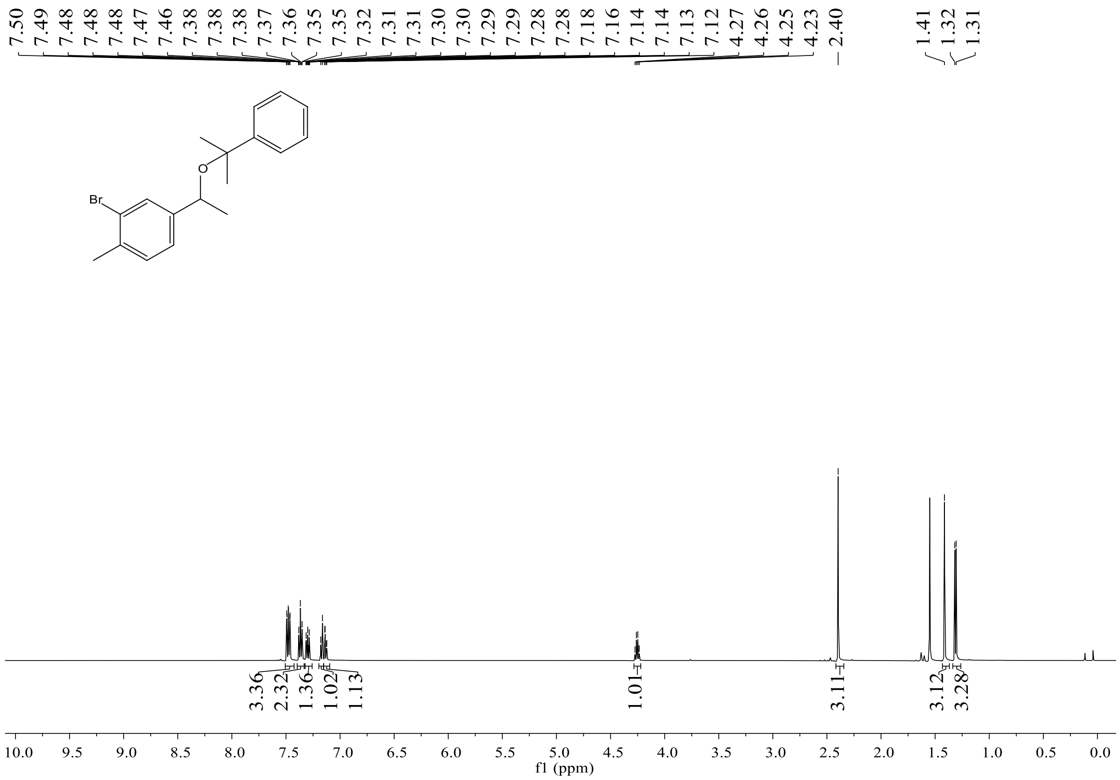

^{1}H NMR spectrum in $CDCl_3$.

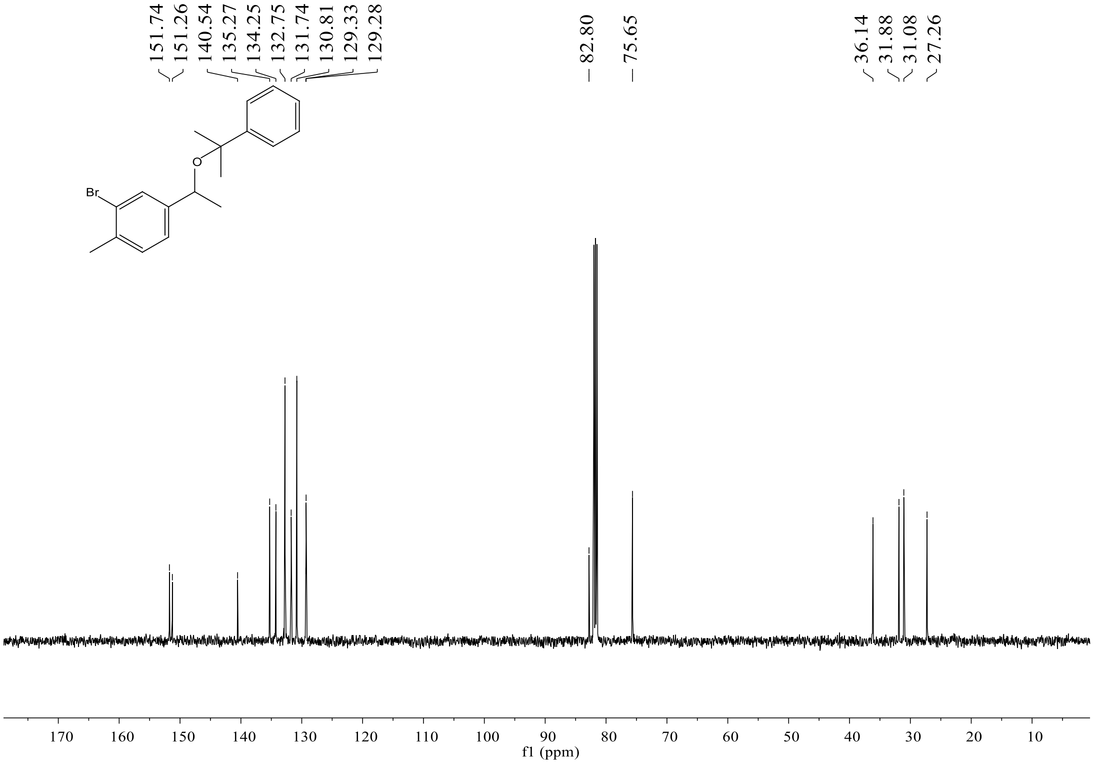

^{13}C NMR spectrum in $CDCl_3$.

53d

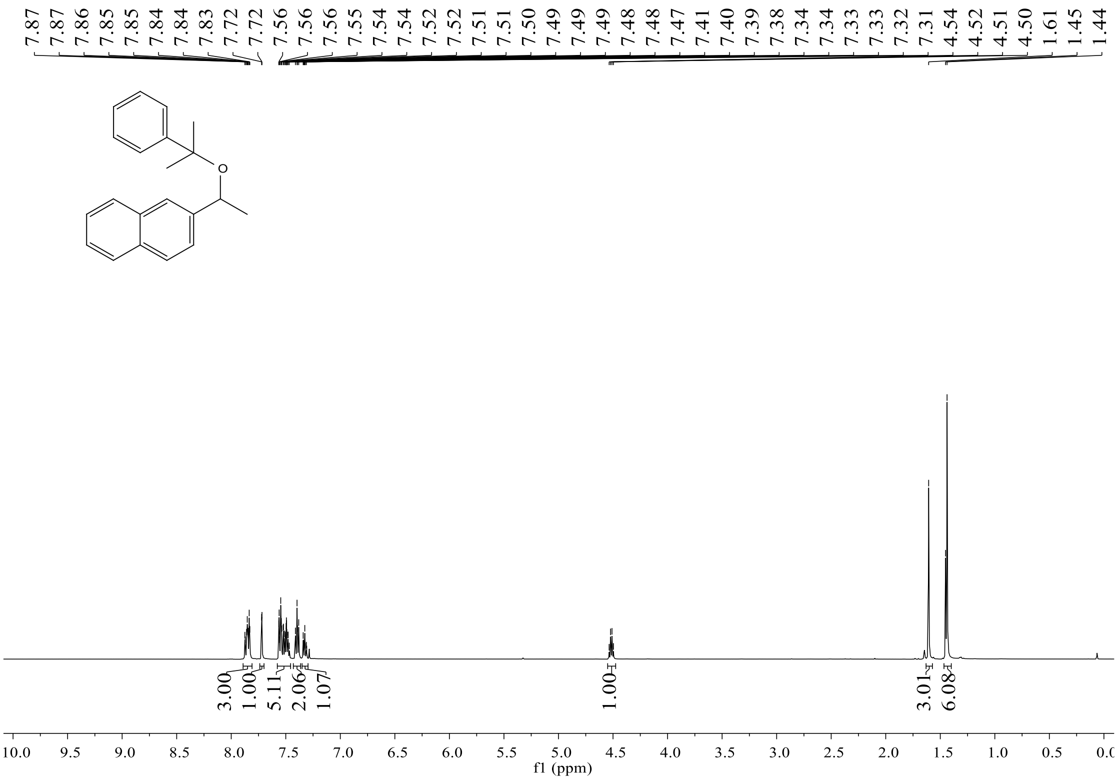

^{1}H NMR spectrum in $CDCl_3$.

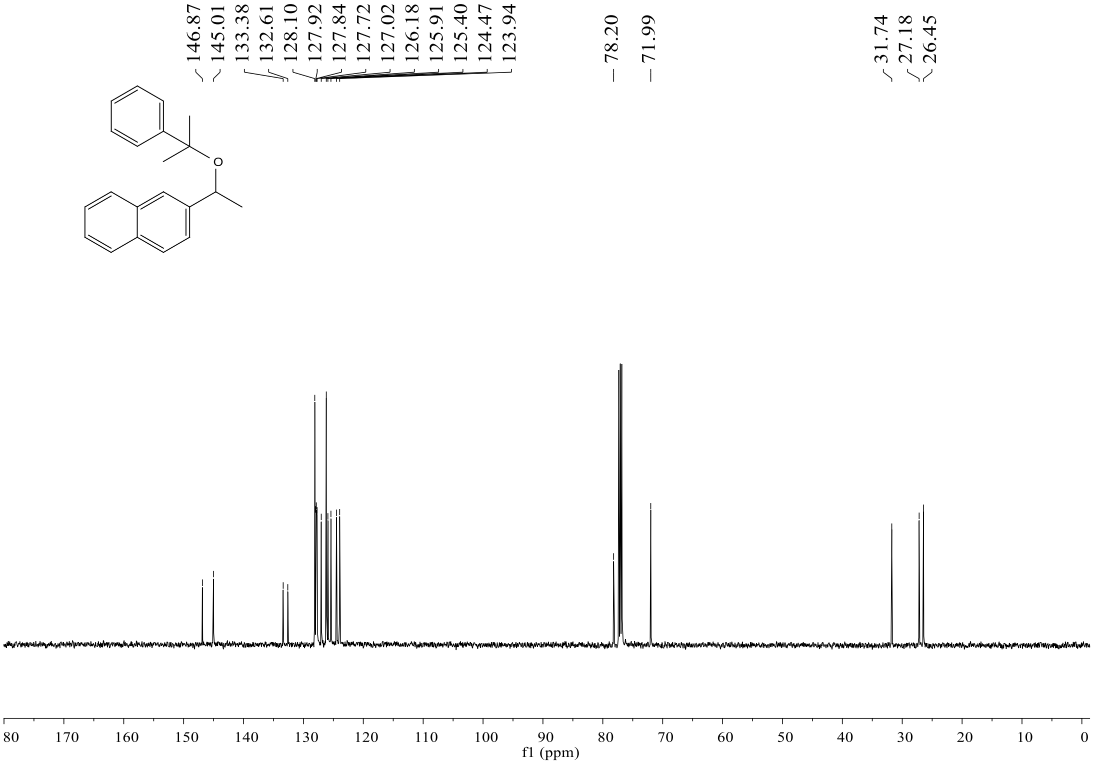

^{13}C NMR spectrum in $CDCl_3$.

54d

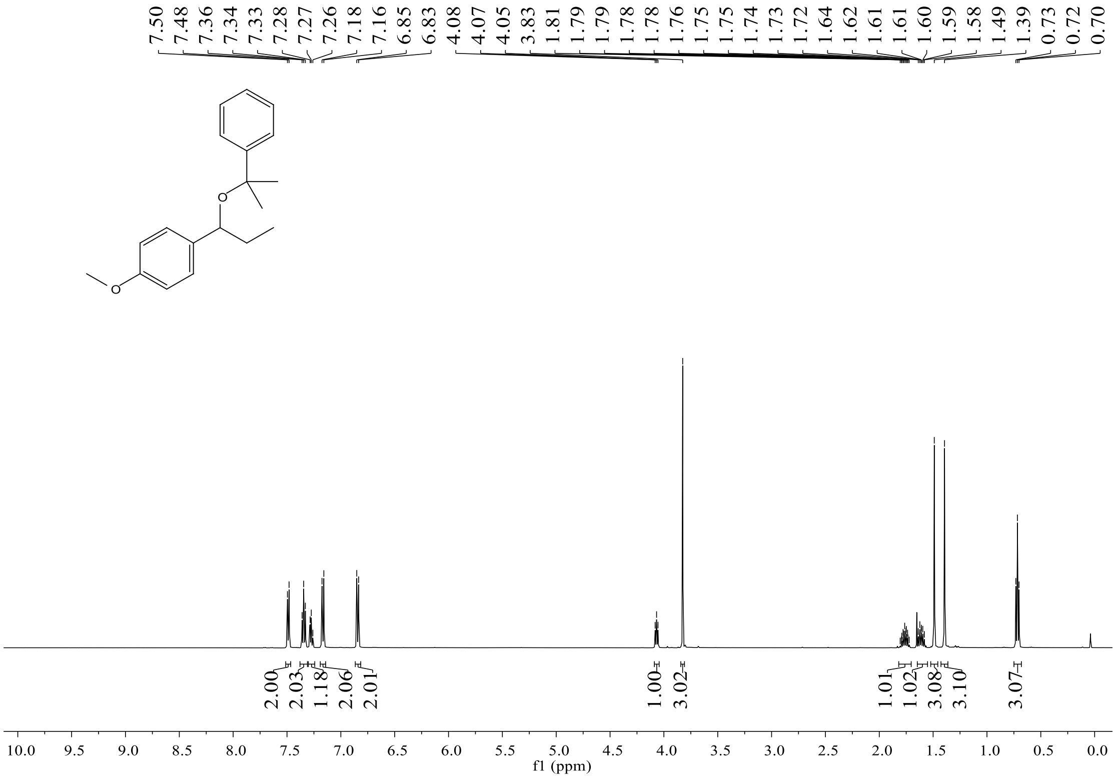

^{1}H NMR spectrum in $CDCl_3$.

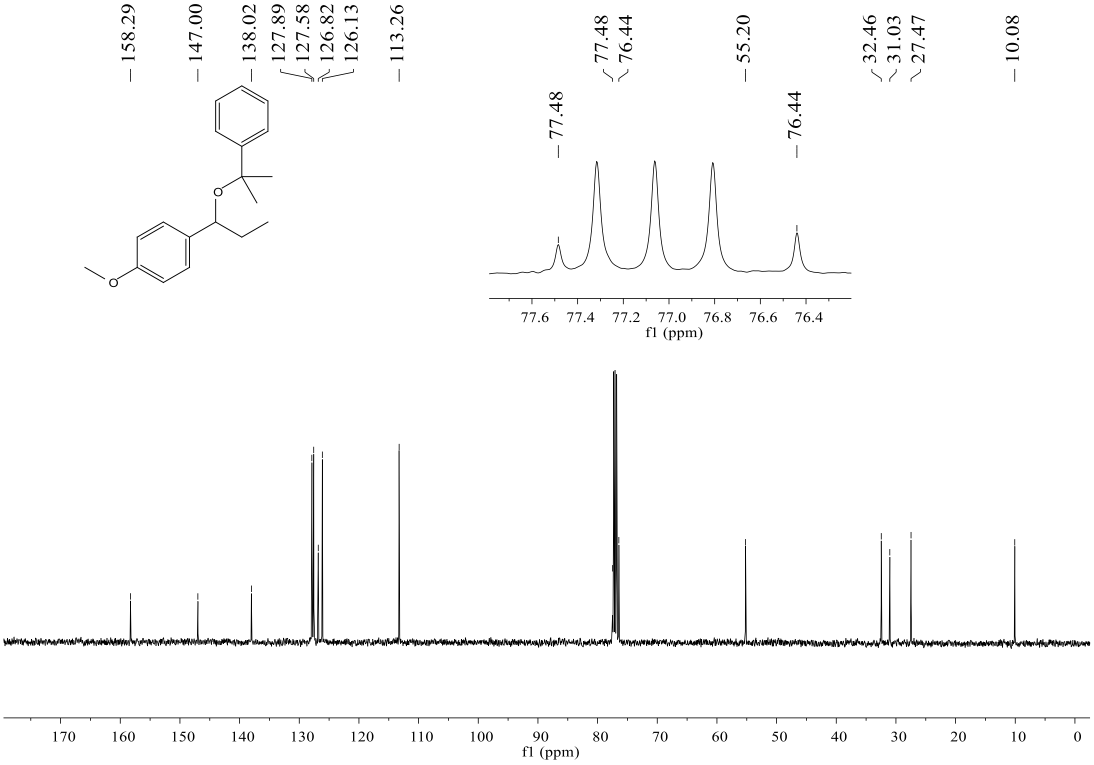

^{13}C NMR spectrum in $CDCl_3$.

55d

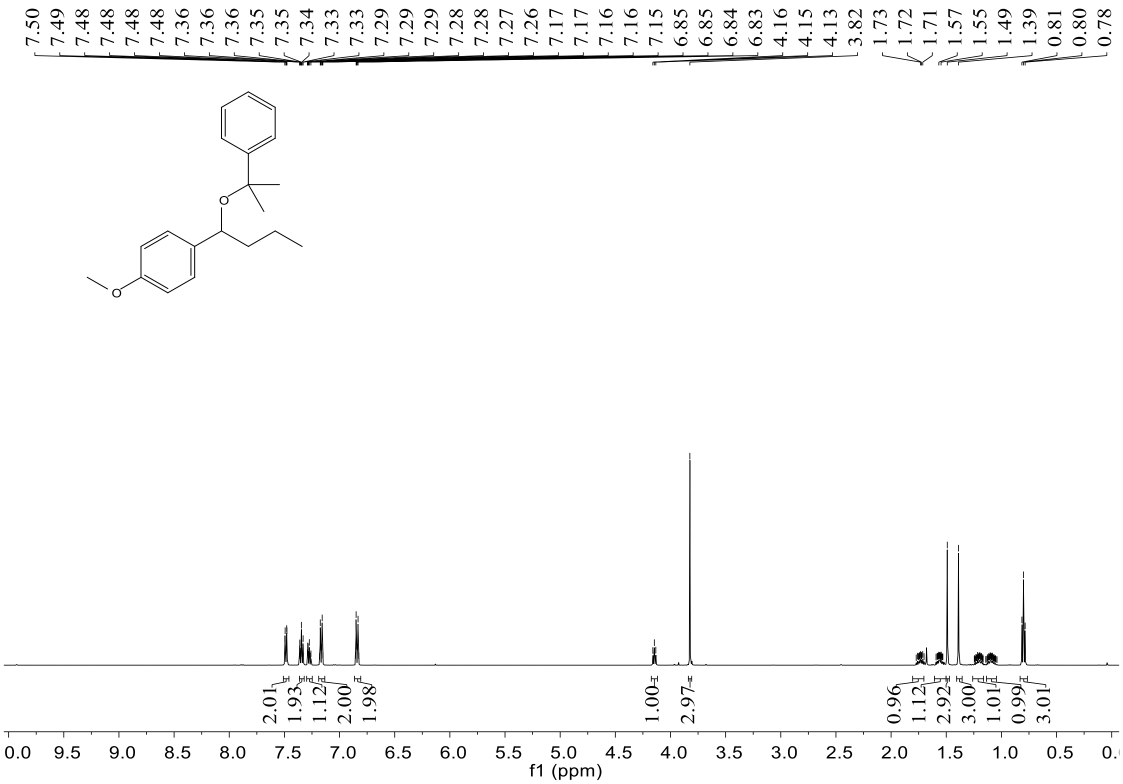

^{1}H NMR spectrum in $CDCl_3$.

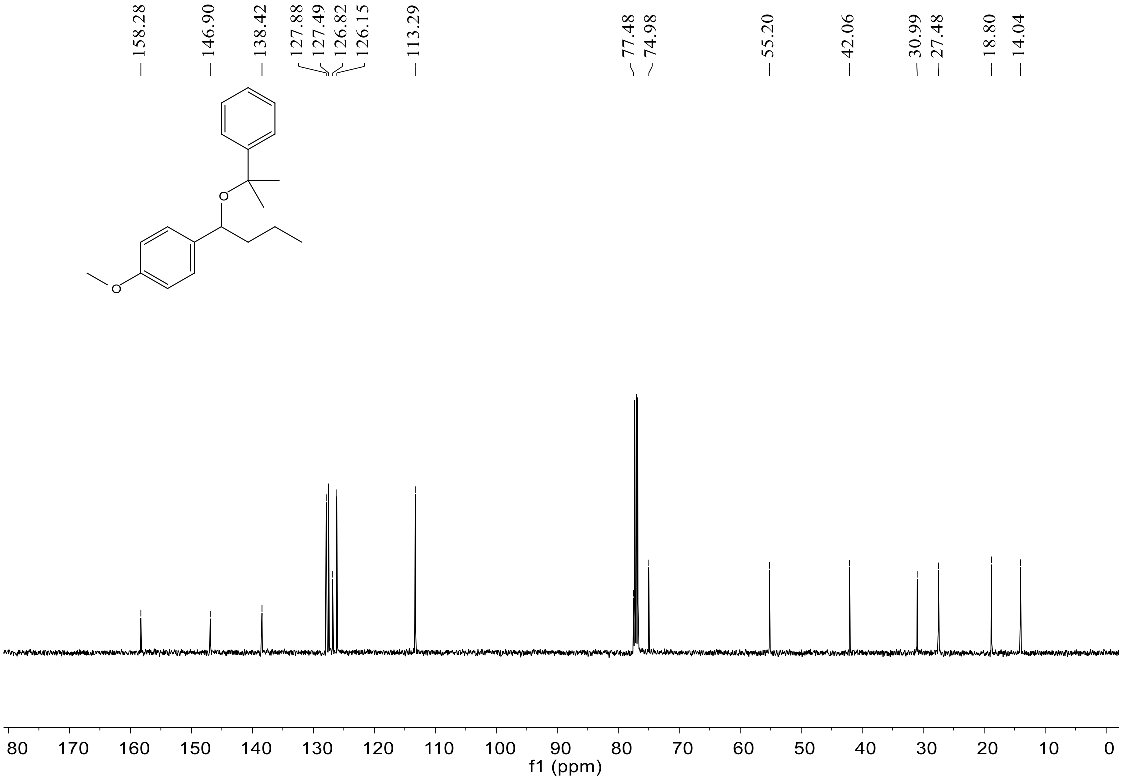

^{13}C NMR spectrum in $CDCl_3$.

56d

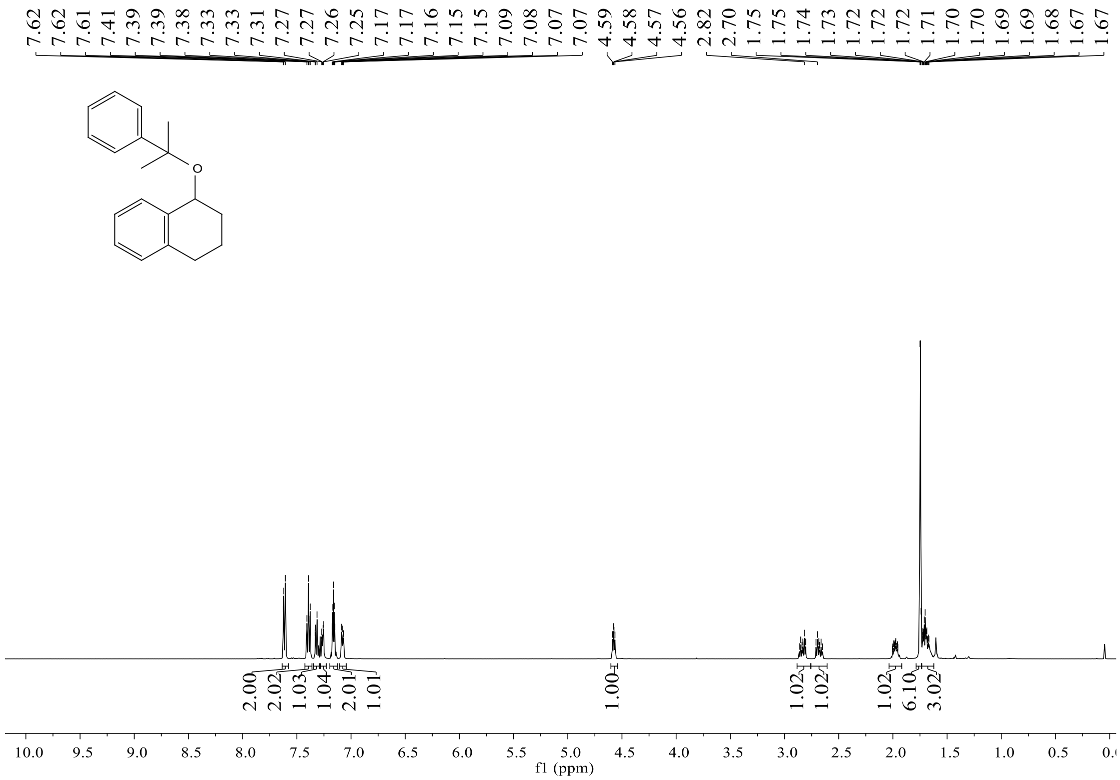

^{1}H NMR spectrum in $CDCl_3$.

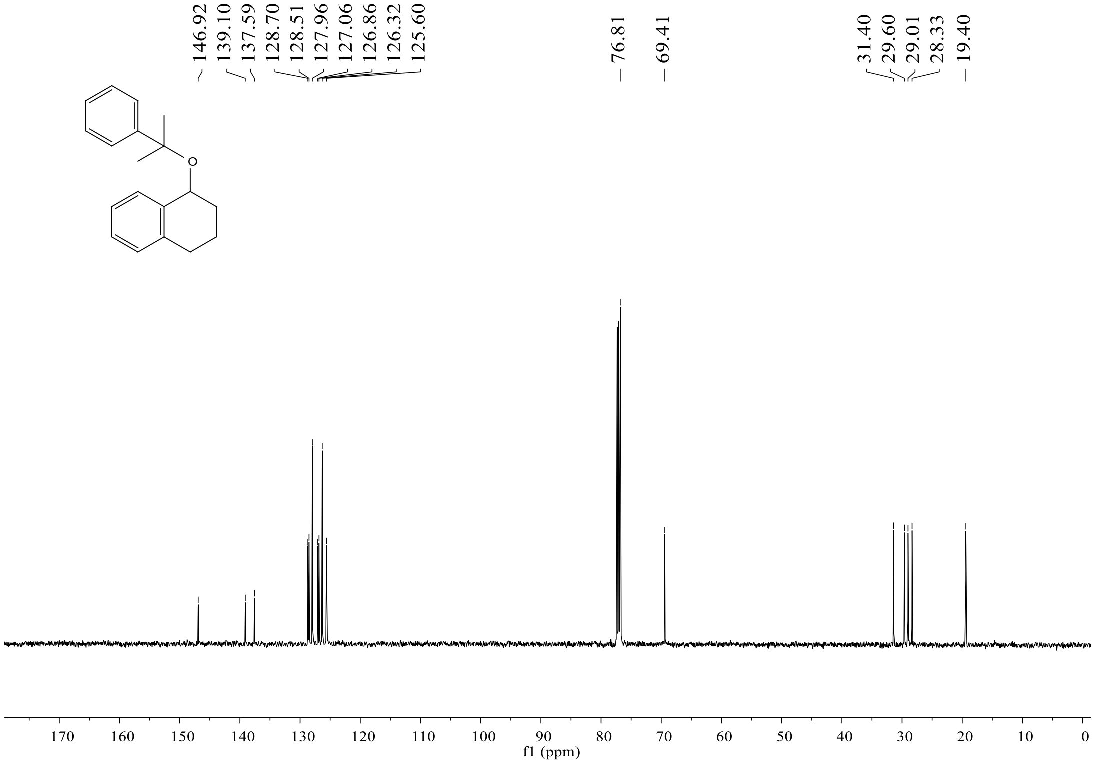

^{13}C NMR spectrum in $CDCl_3$.

57d

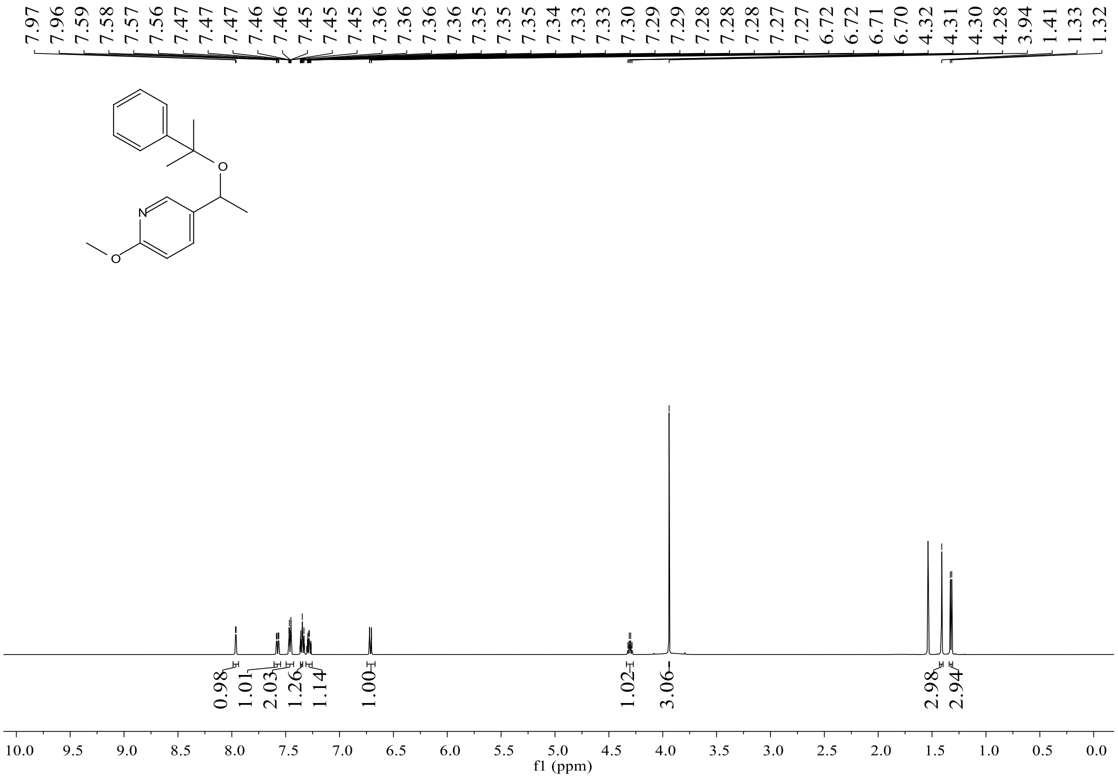

^{1}H NMR spectrum in $CDCl_3$.

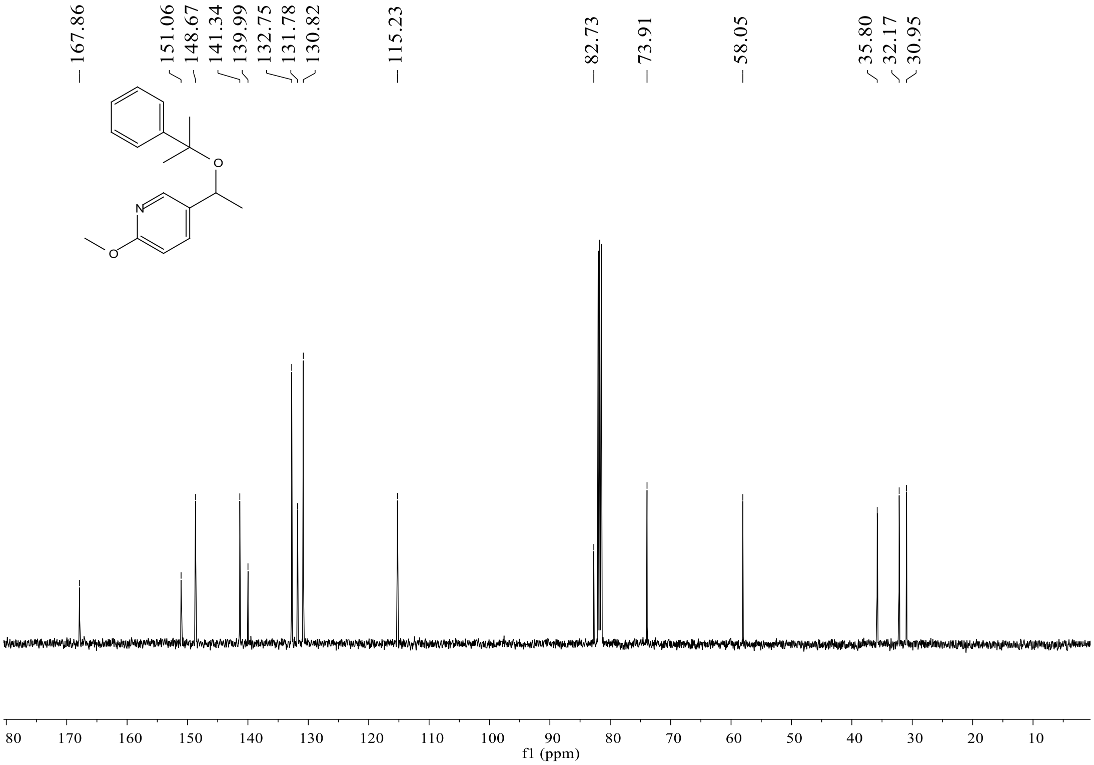

^{13}C NMR spectrum in $CDCl_3$.

58d

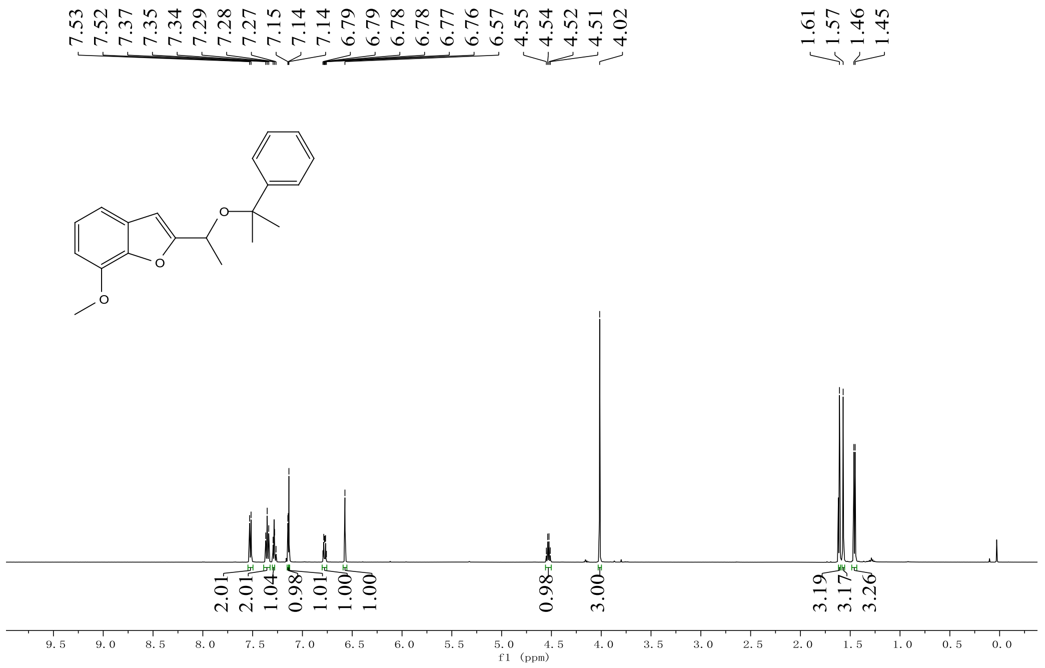

^{1}H NMR spectrum in $CDCl_3$.

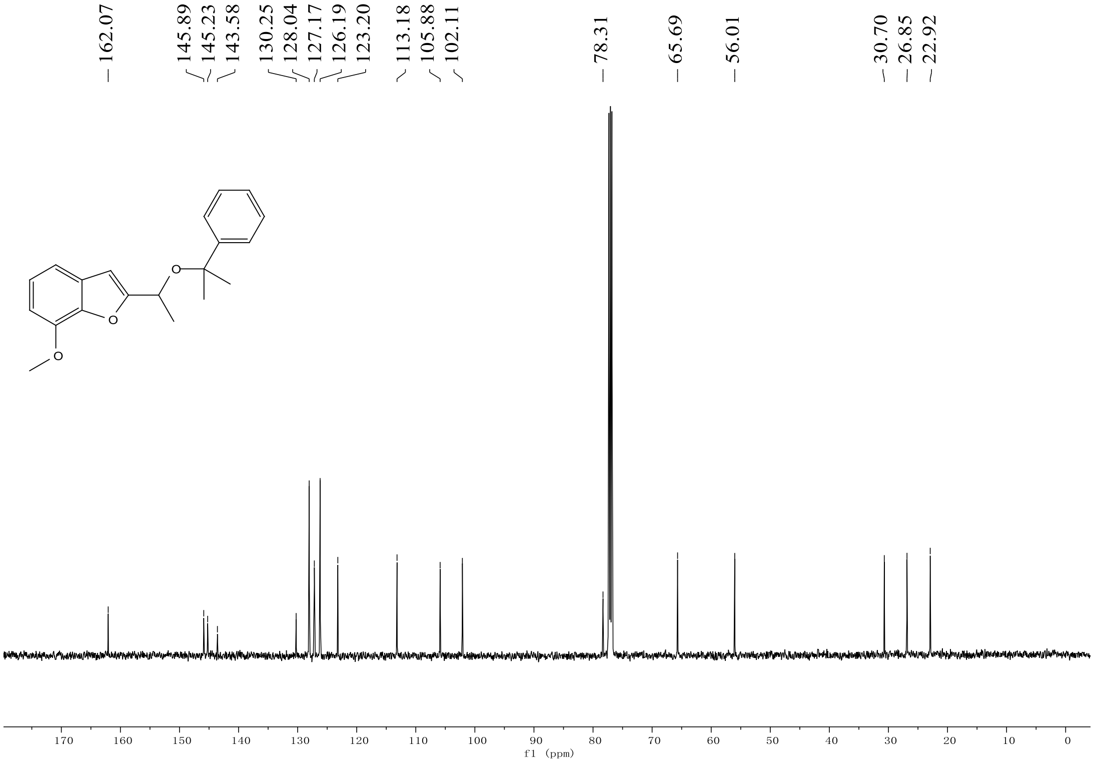

^{13}C NMR spectrum in $CDCl_3$.

59d

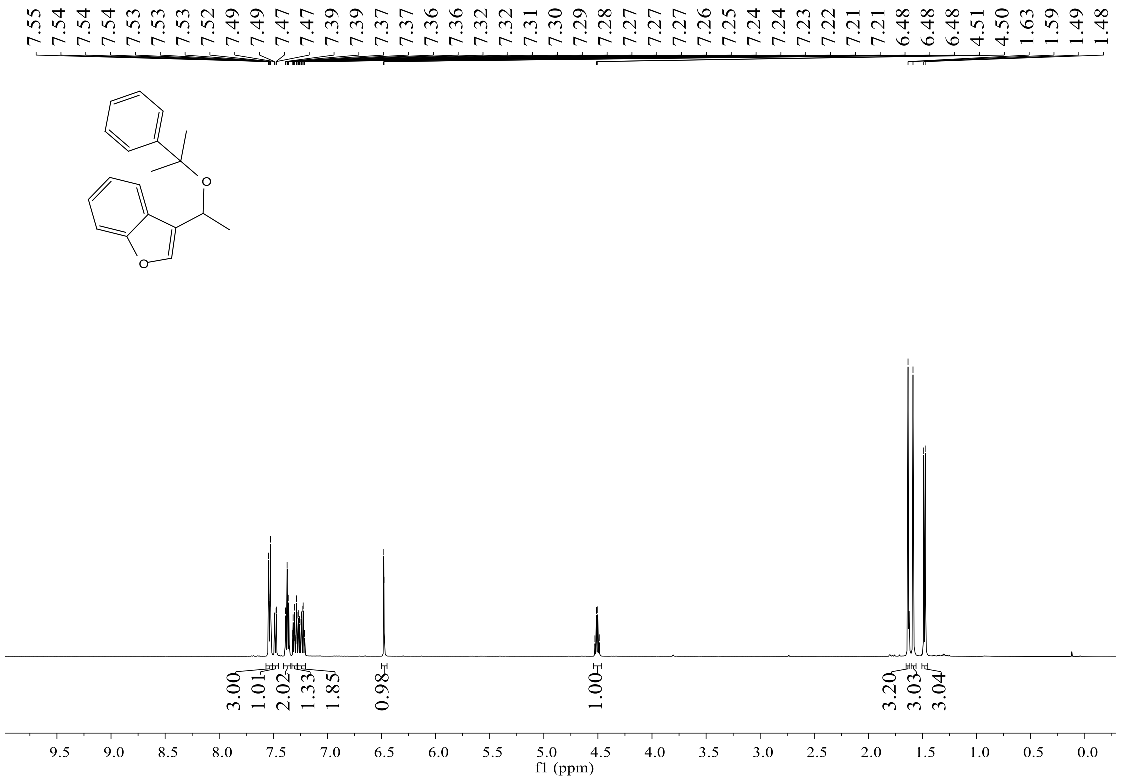

^{1}H NMR spectrum in $CDCl_3$.

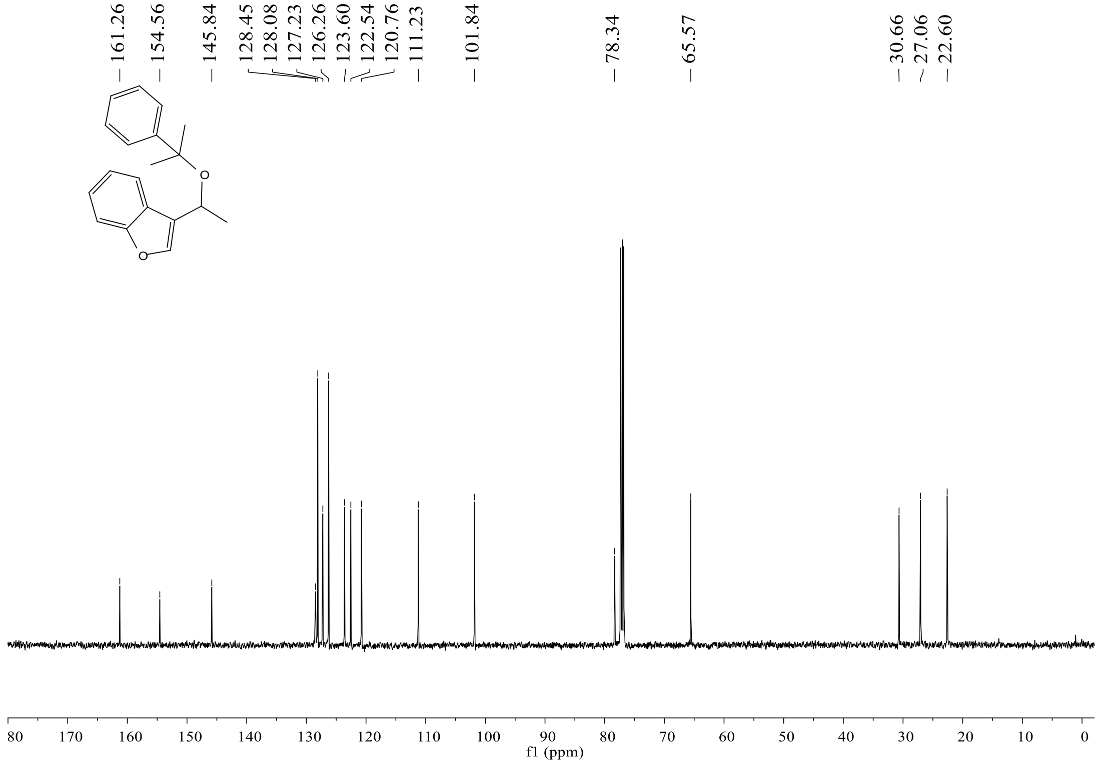

^{13}C NMR spectrum in $CDCl_3$.

60d

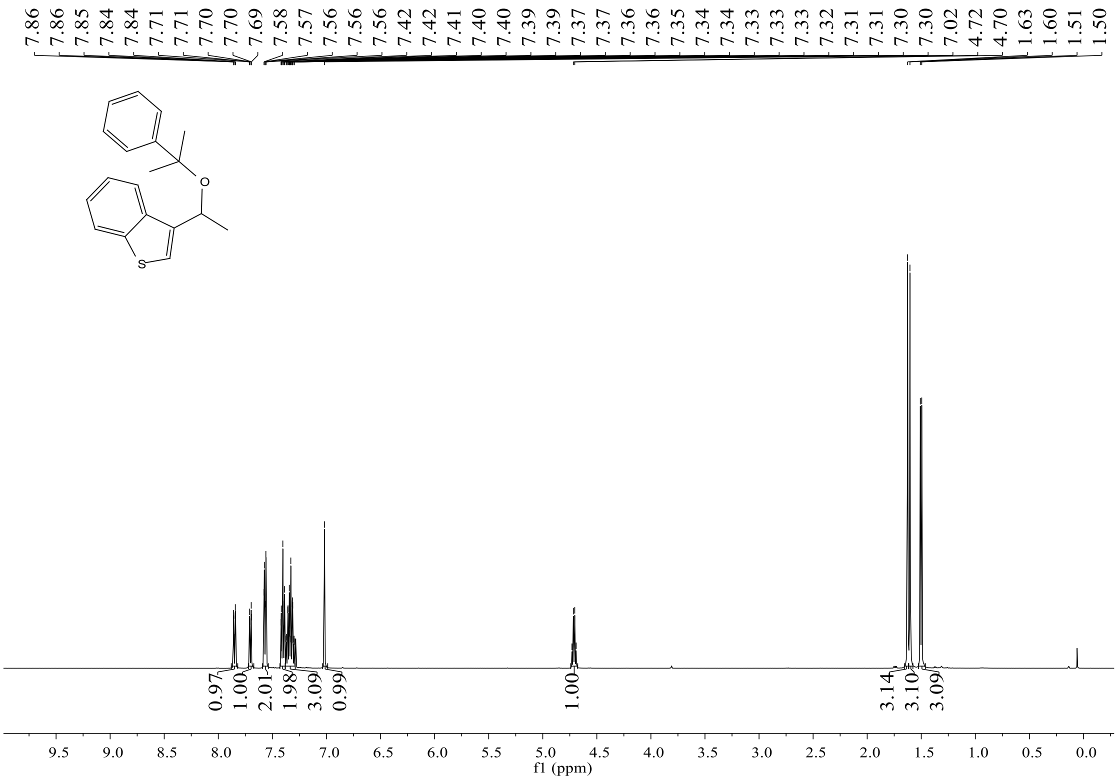

^{1}H NMR spectrum in $CDCl_3$.

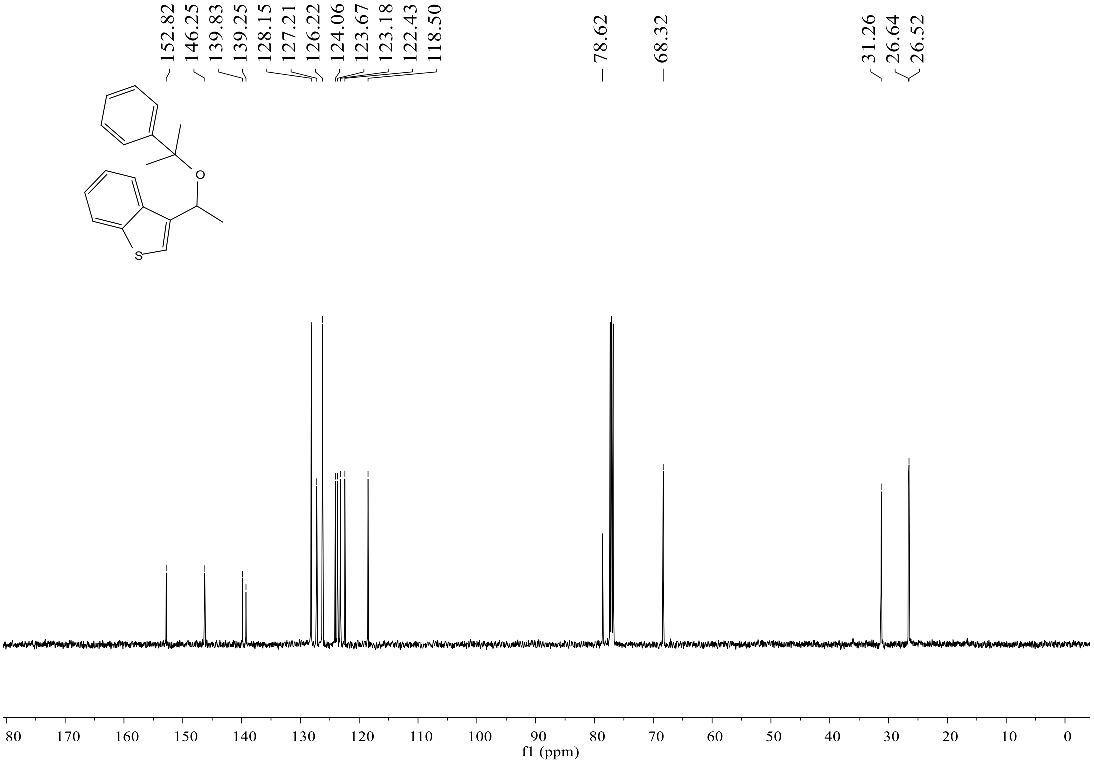

^{13}C NMR spectrum in $CDCl_3$.

61d

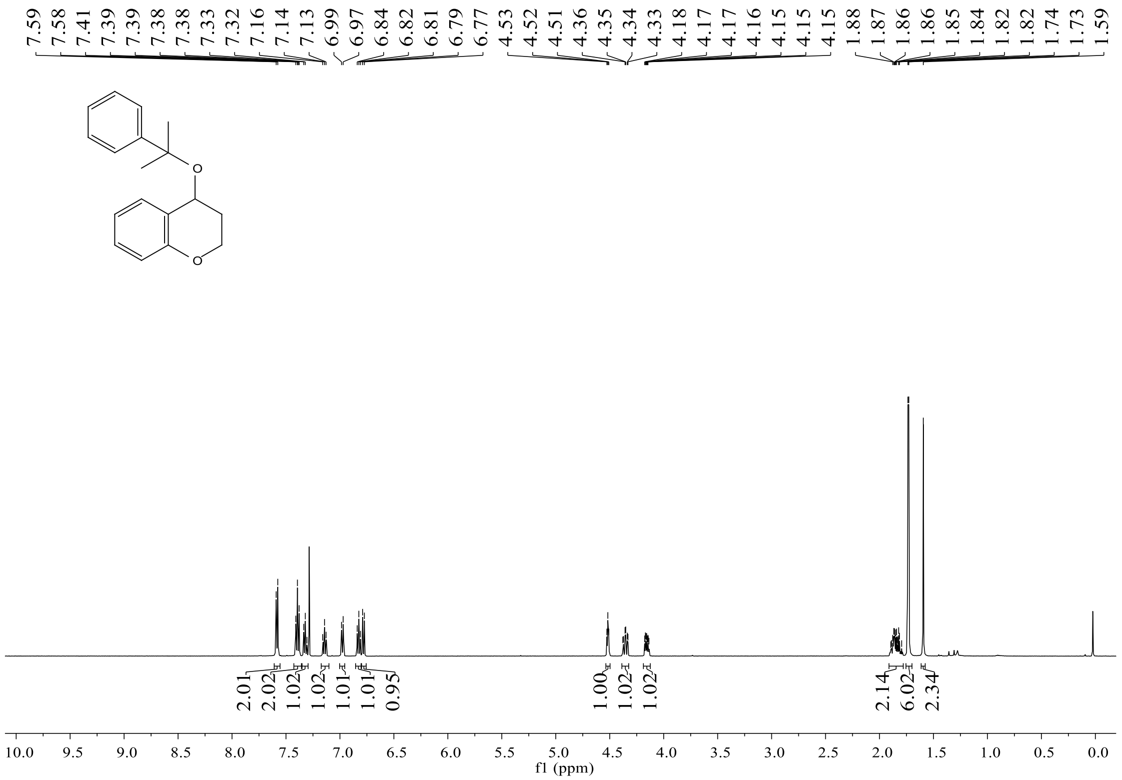

^{1}H NMR spectrum in $CDCl_3$.

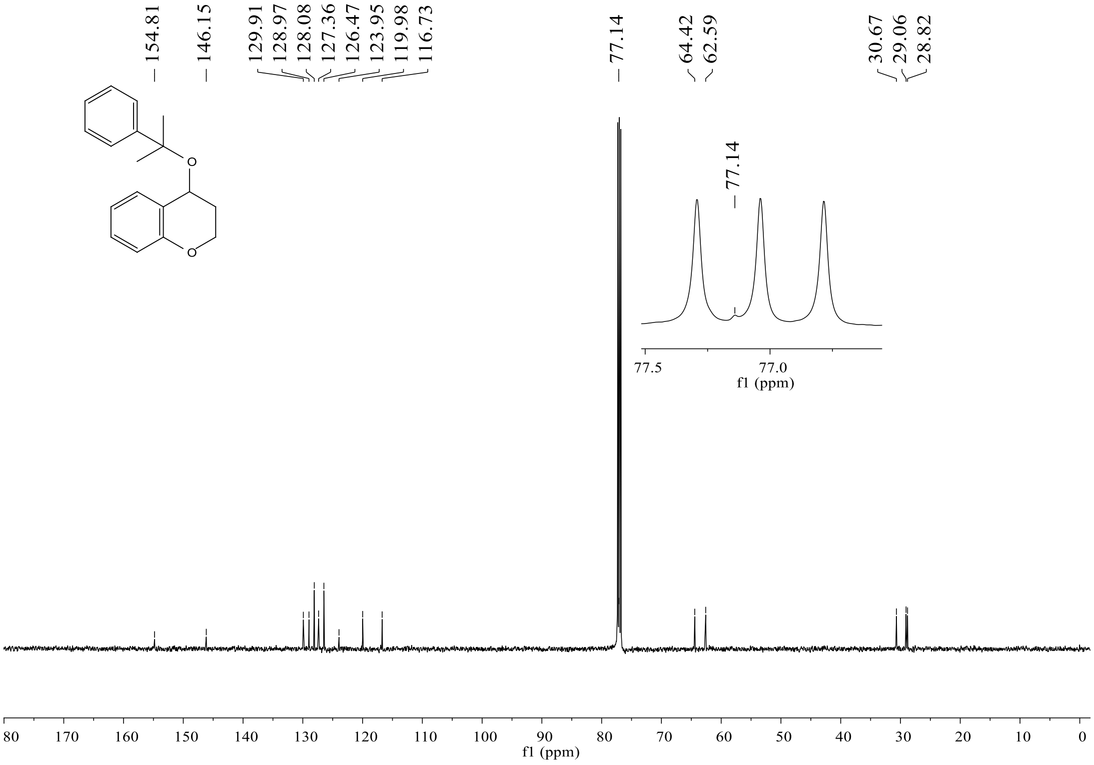

^{13}C NMR spectrum in $CDCl_3$.

62d

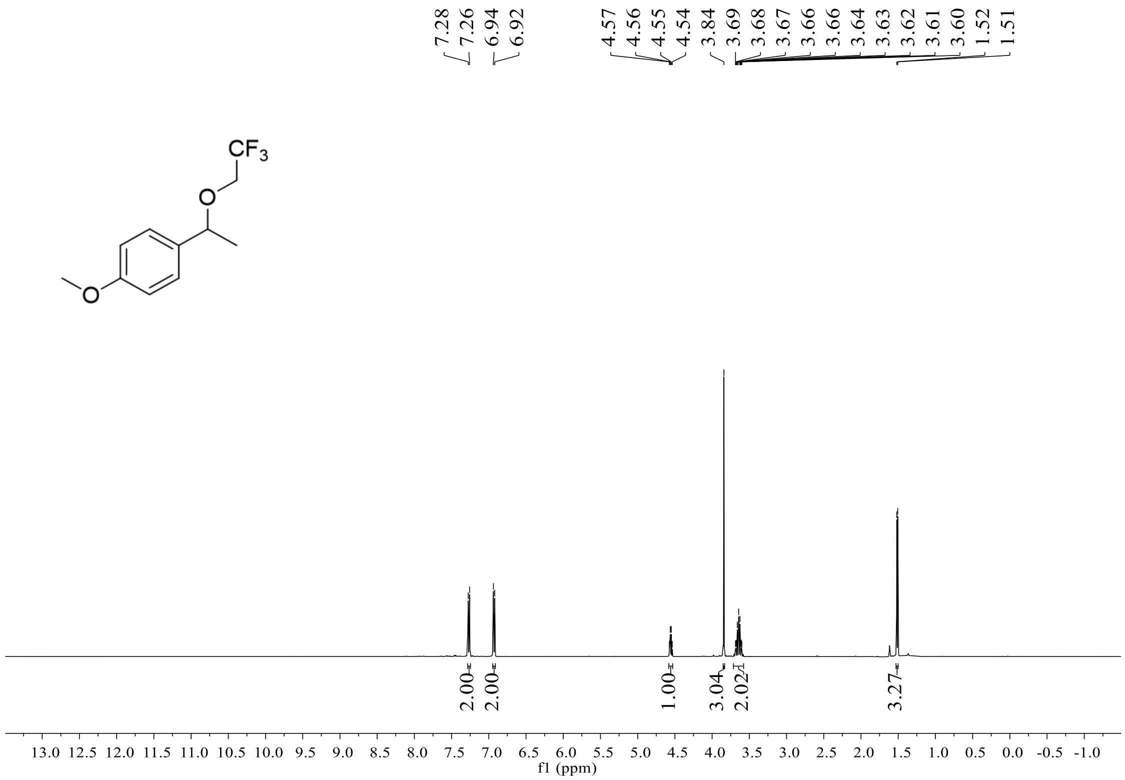

^{1}H NMR spectrum in $CDCl_3$.

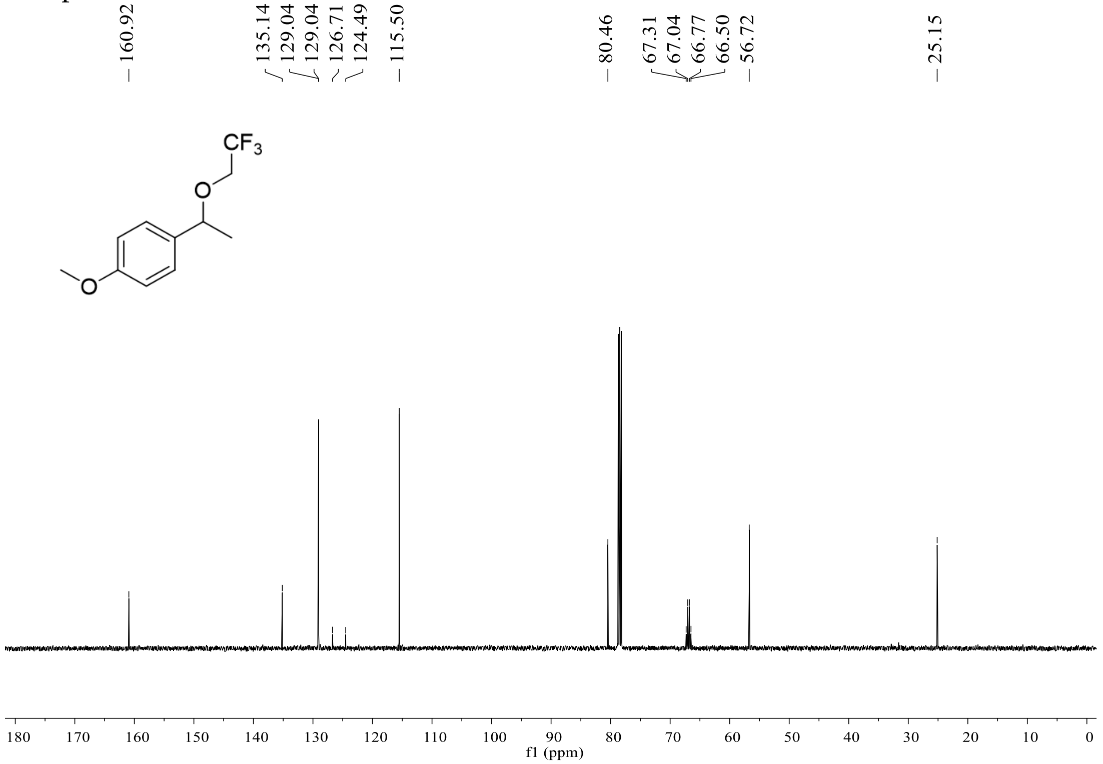

^{13}C NMR spectrum in $CDCl_3$.

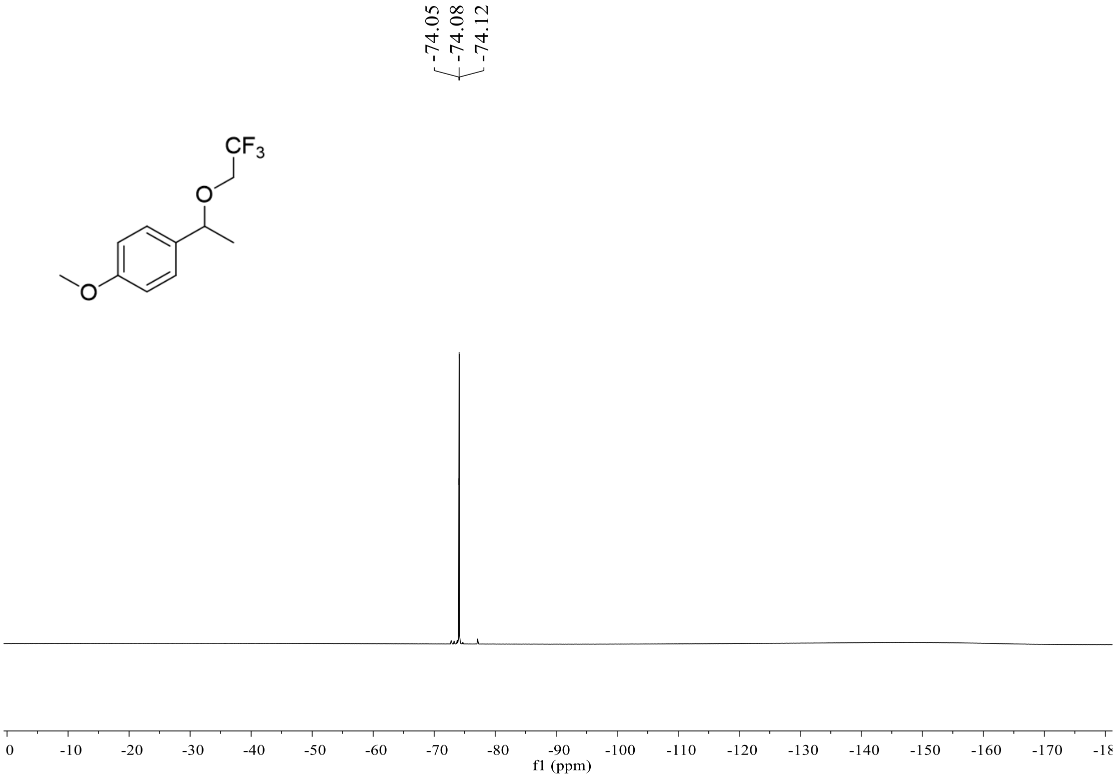

^{19}F NMR spectrum in $CDCl_3$.

63d

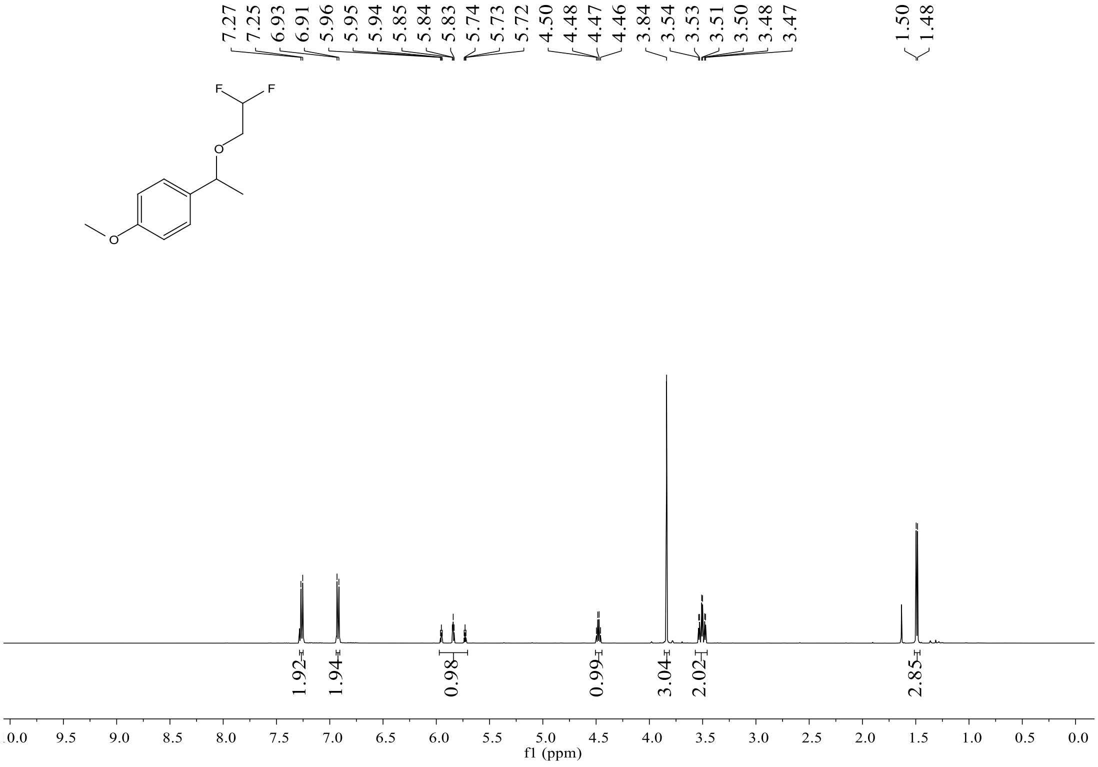

^1H NMR spectrum in $CDCl_3$.

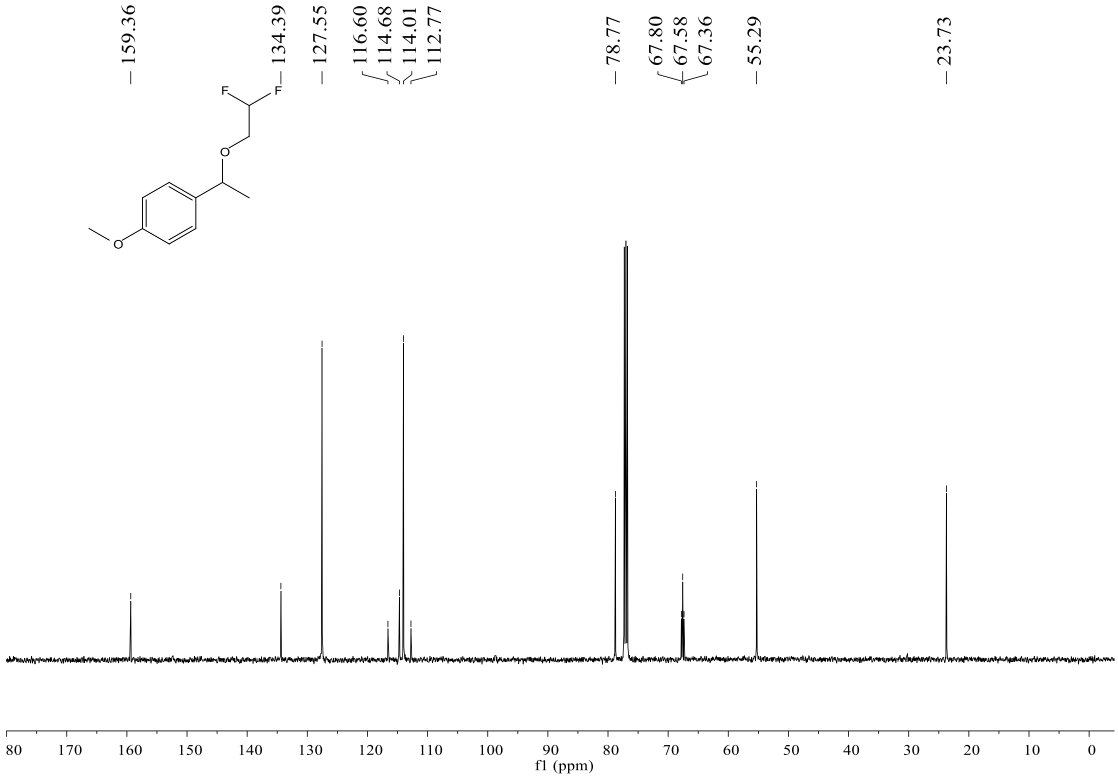

^{13}C NMR spectrum in $CDCl_3$.

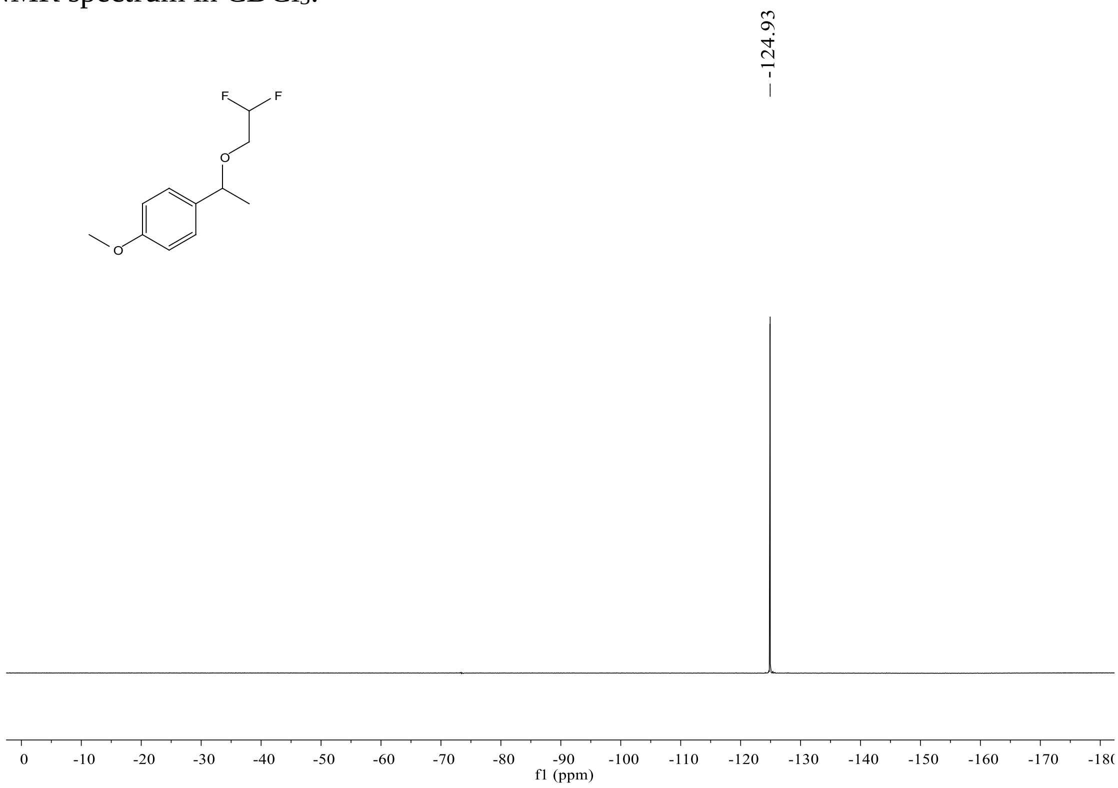

^{19}F NMR spectrum in $CDCl_3$.

64d

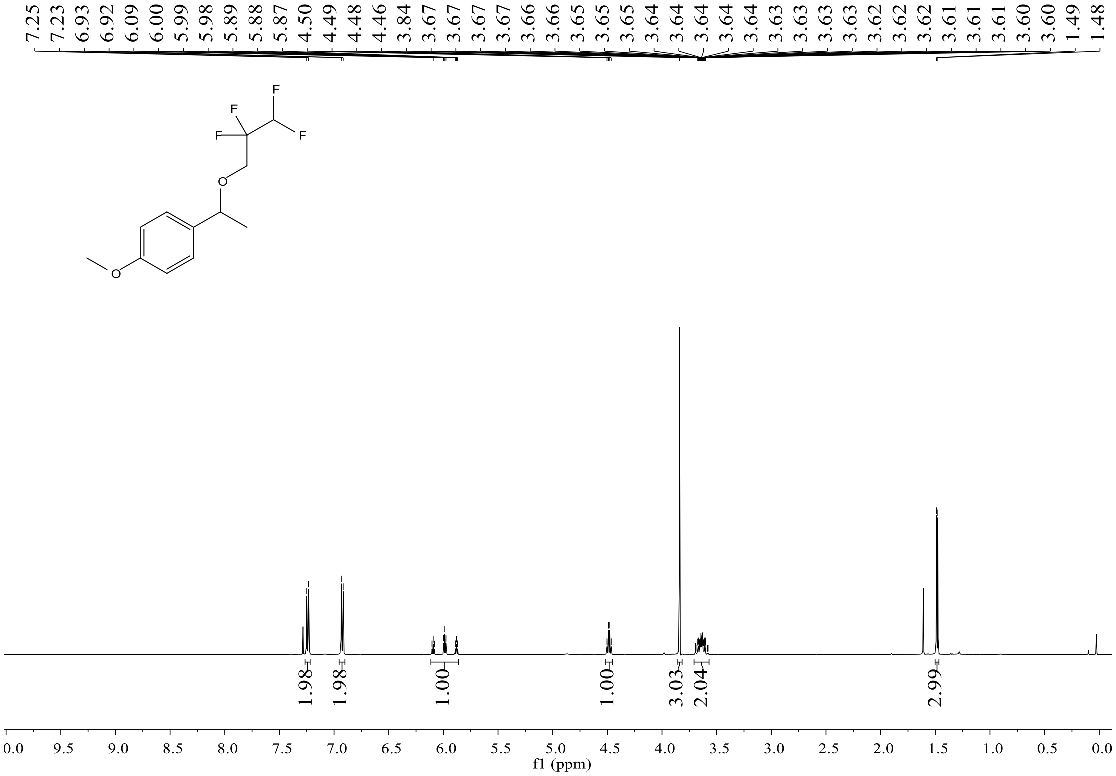

^{1}H NMR spectrum in $CDCl_3$.

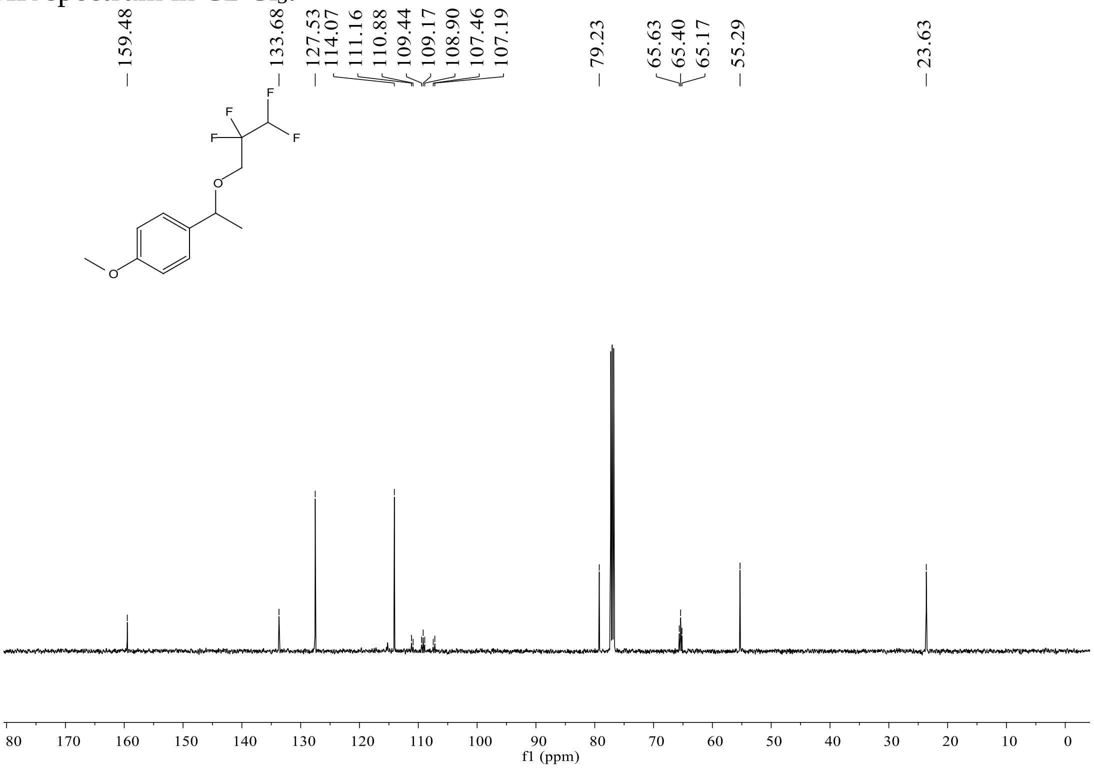

^{13}C NMR spectrum in $CDCl_3$.

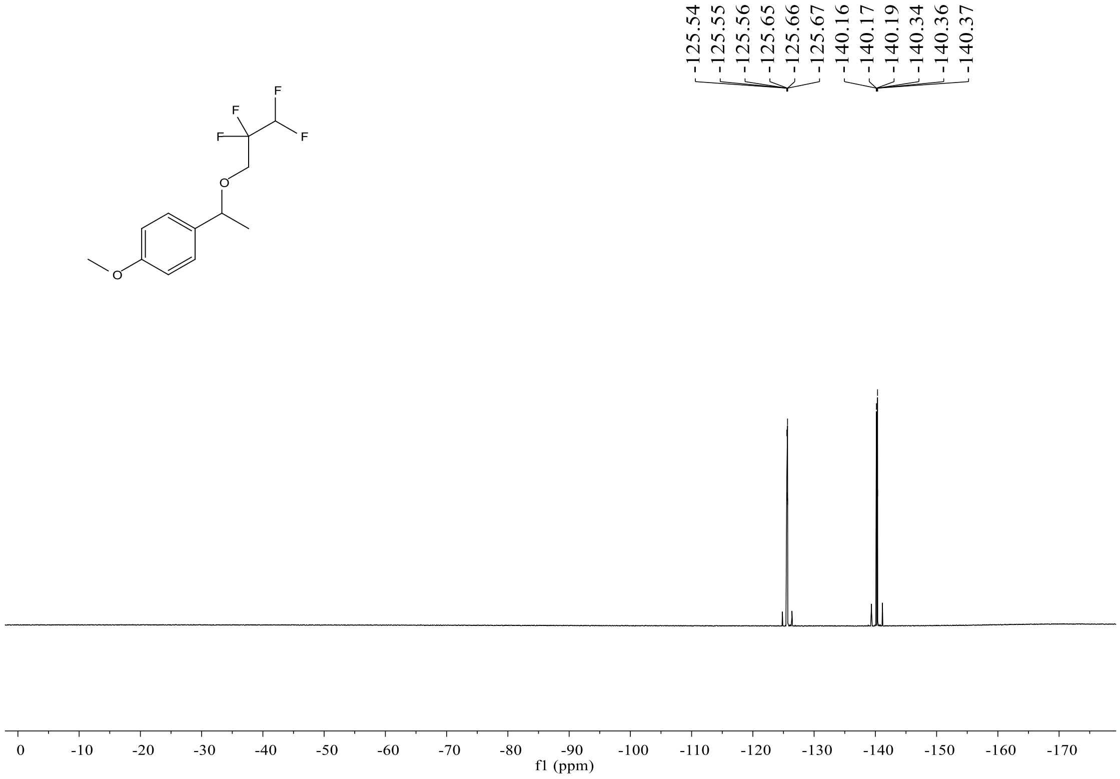

^{19}F NMR spectrum in $CDCl_3$.

65d

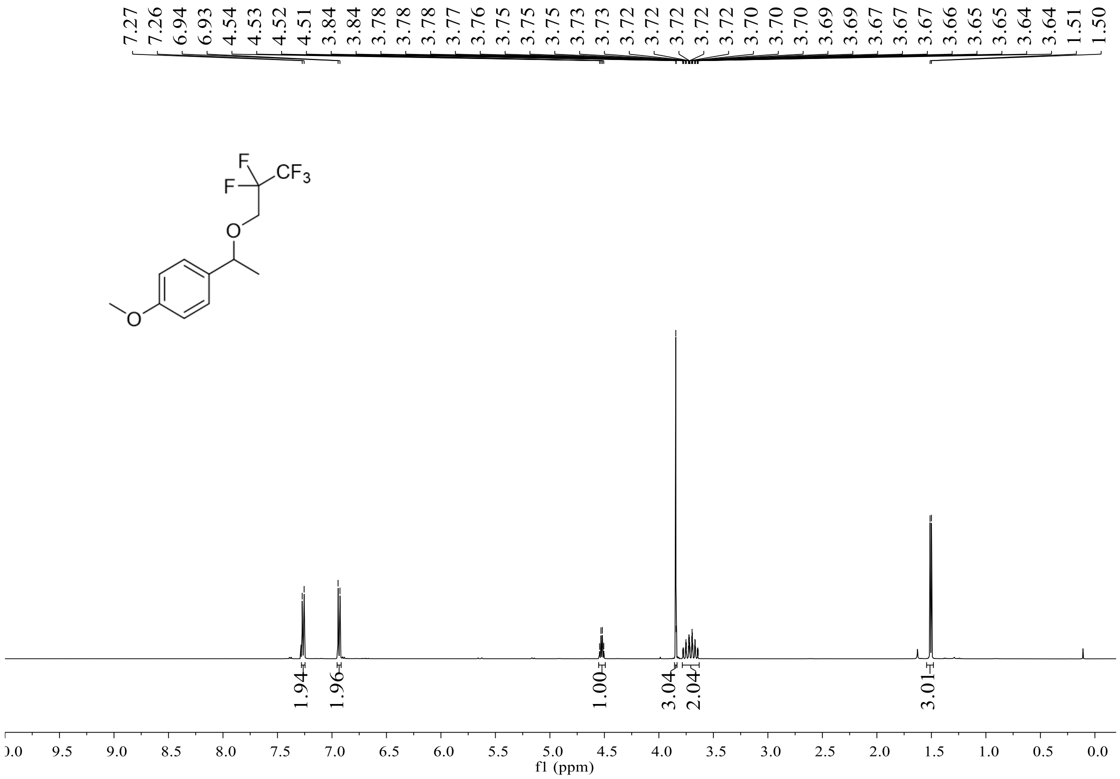

^{1}H NMR spectrum in $CDCl_3$.

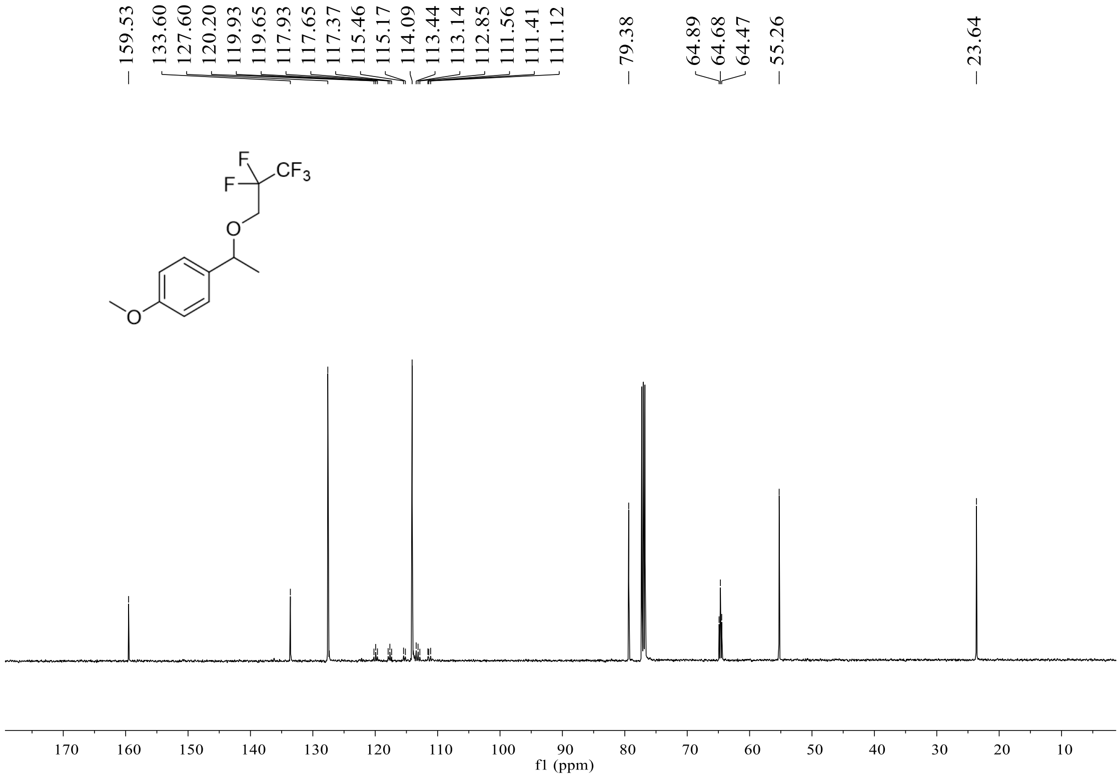

^{13}C NMR spectrum in $CDCl_3$.

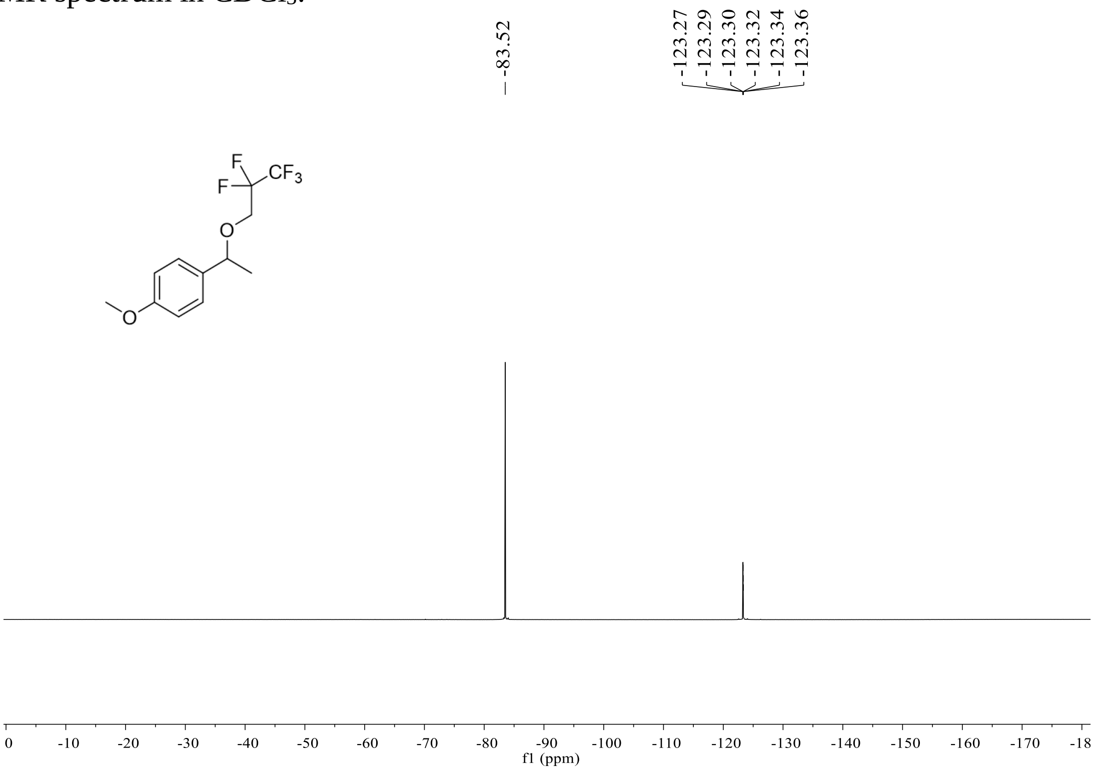

^{19}F NMR spectrum in $CDCl_3$.

66d

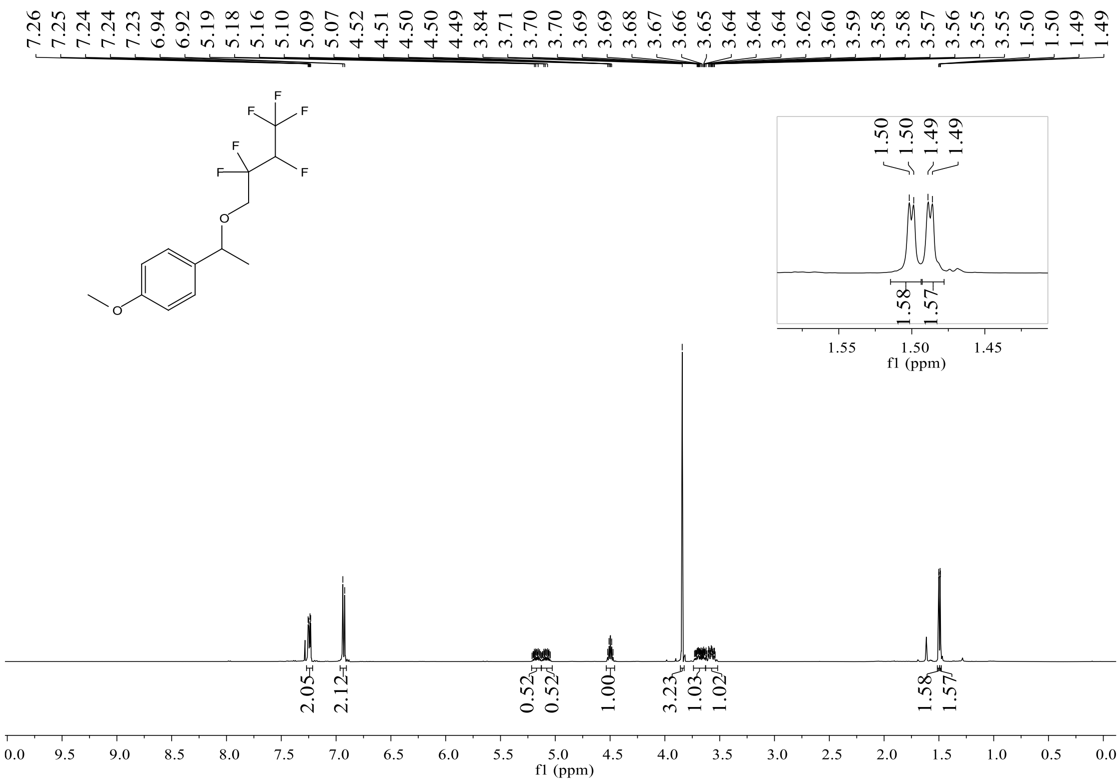

^{1}H NMR spectrum in $CDCl_3$.

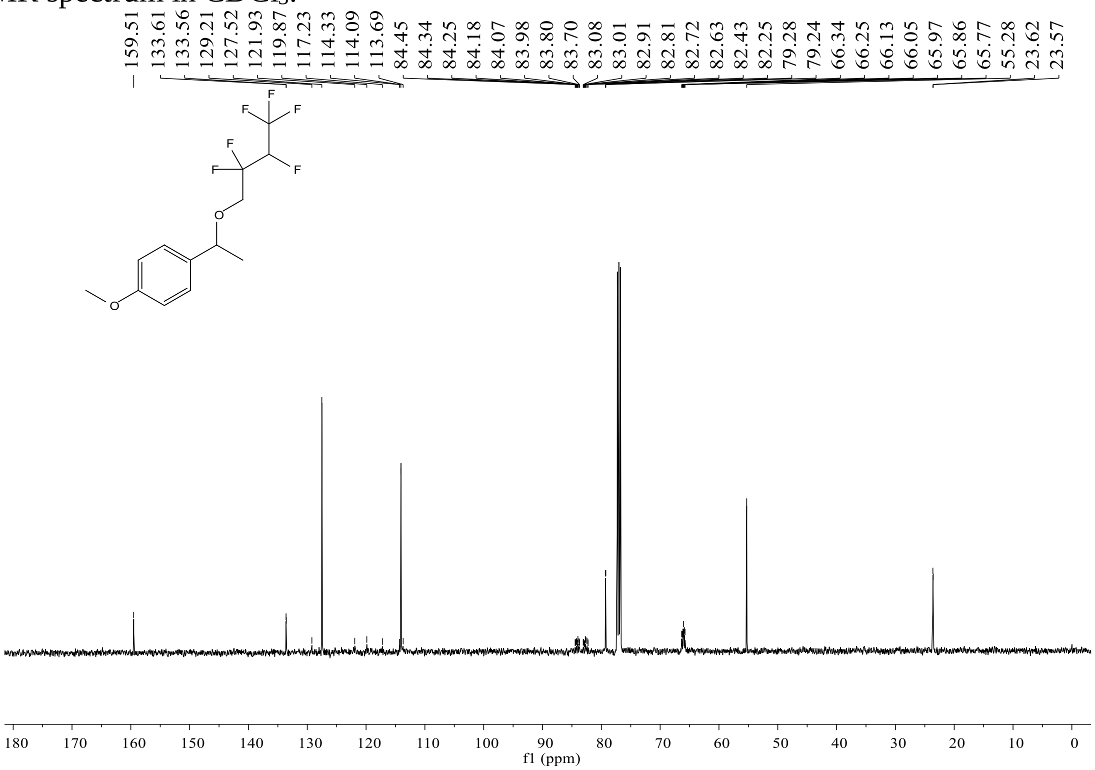

^{13}C NMR spectrum in $CDCl_3$.

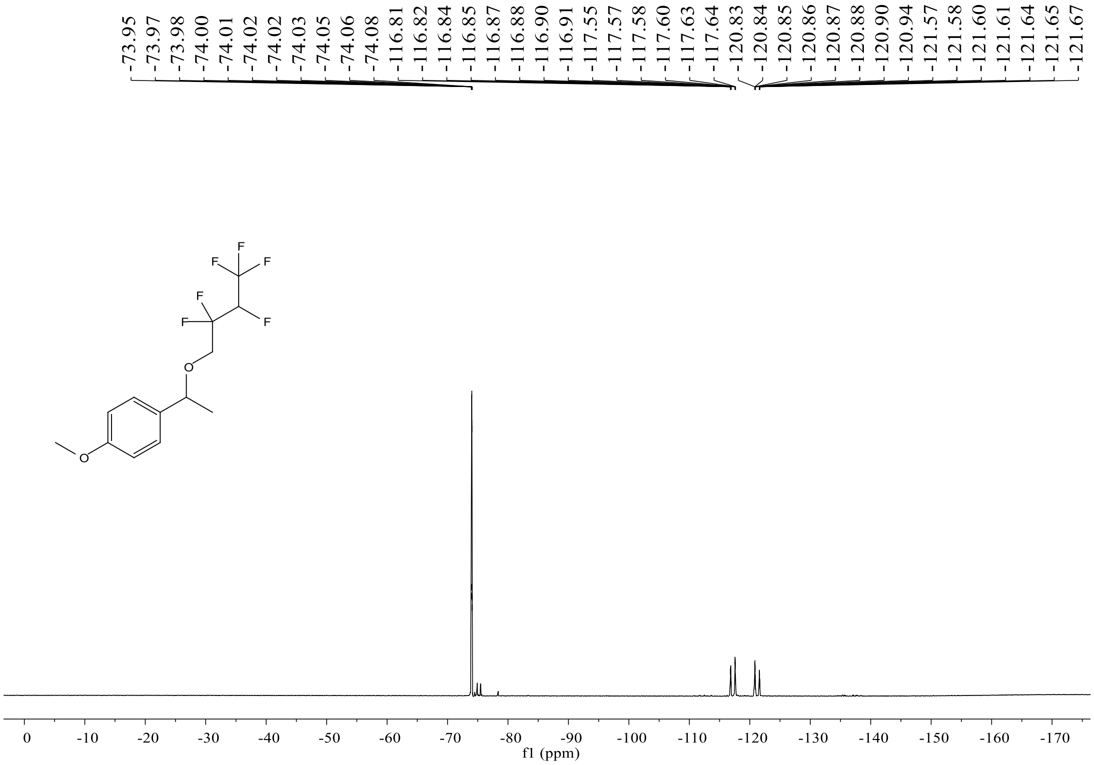

^{19}F NMR spectrum in $CDCl_3$.

67d

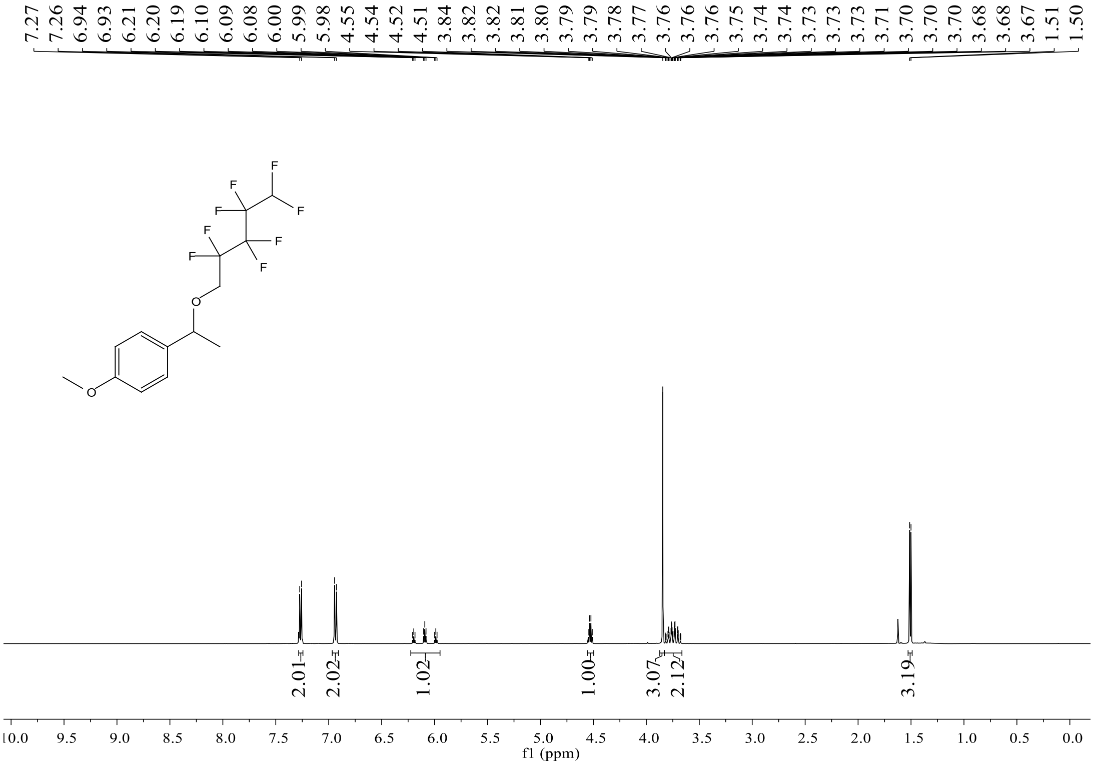

^{1}H NMR spectrum in $CDCl_3$.

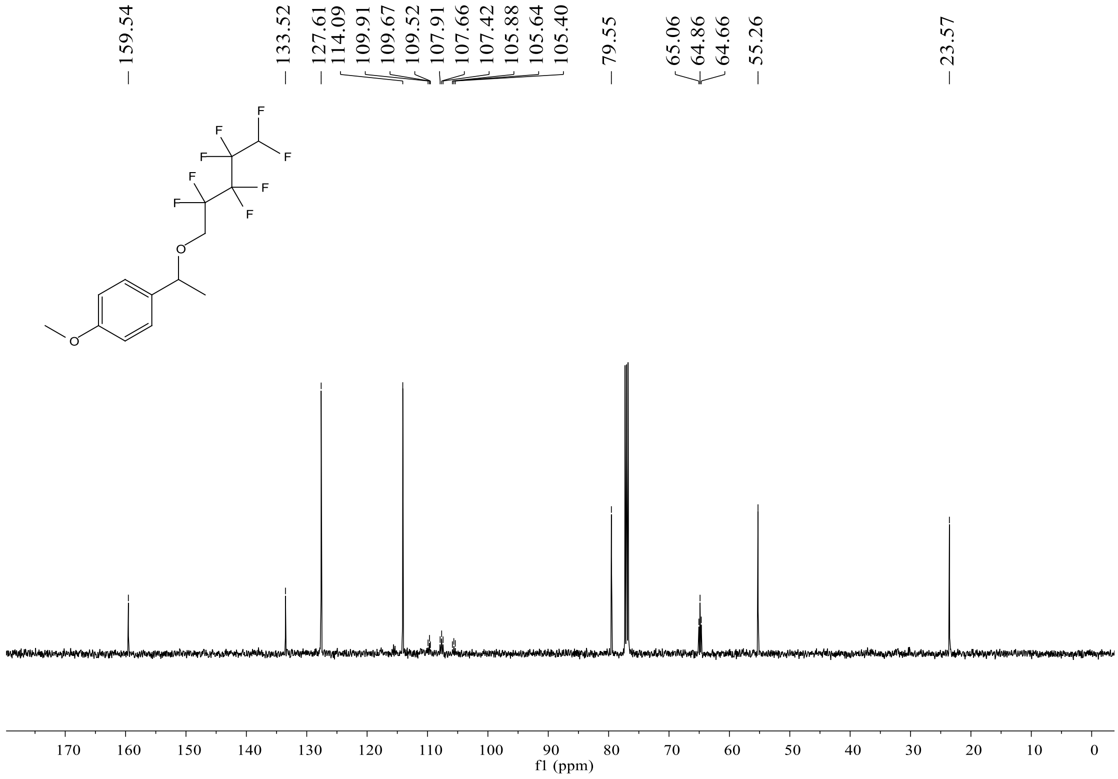

^{13}C NMR spectrum in $CDCl_3$.

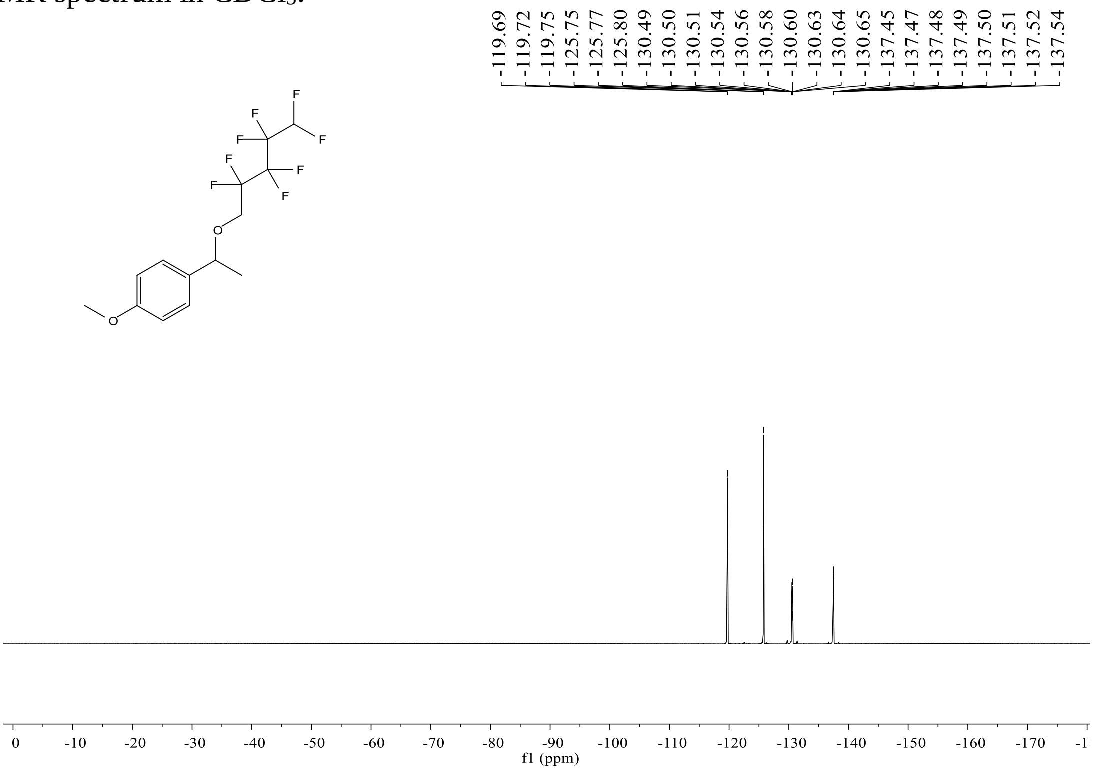

^{19}F NMR spectrum in $CDCl_3$.

68d

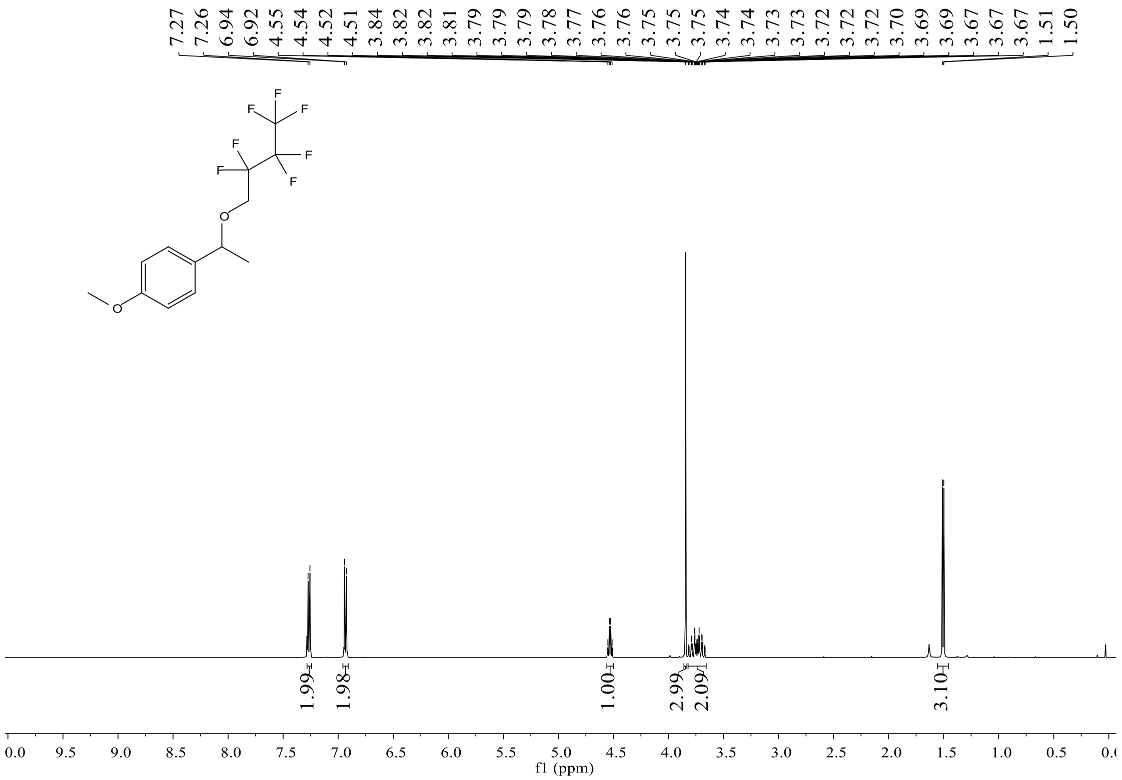

^{1}H NMR spectrum in $CDCl_3$.

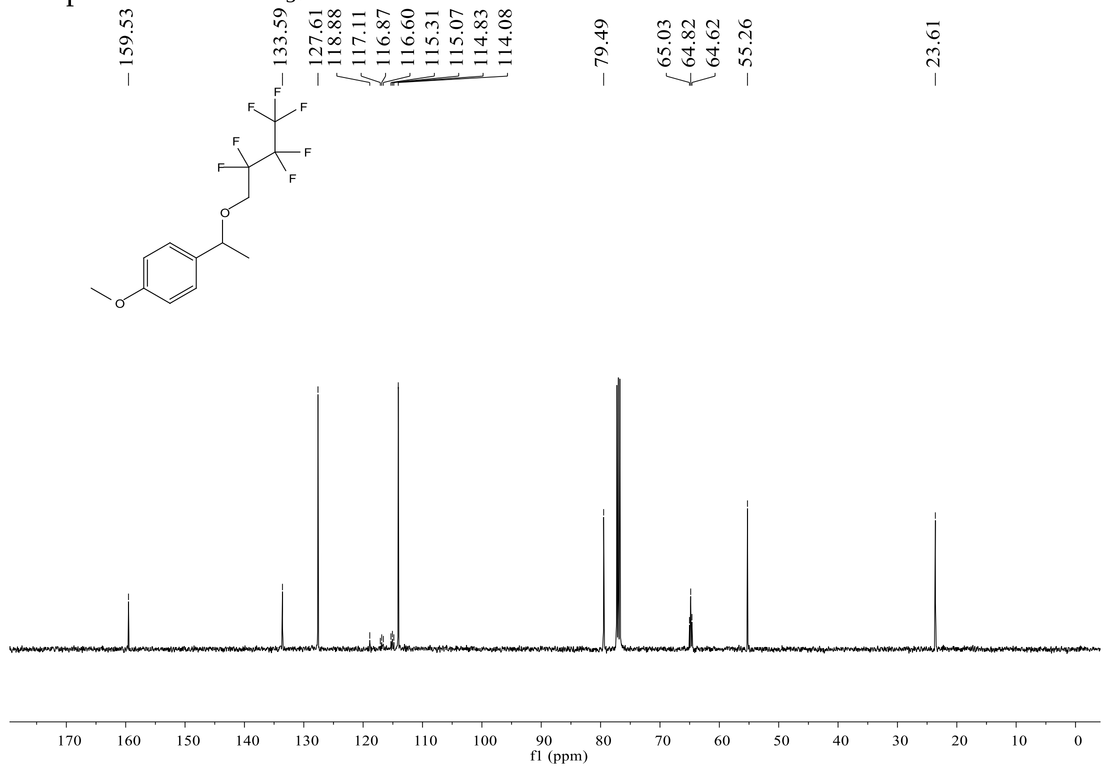

^{13}C NMR spectrum in $CDCl_3$.

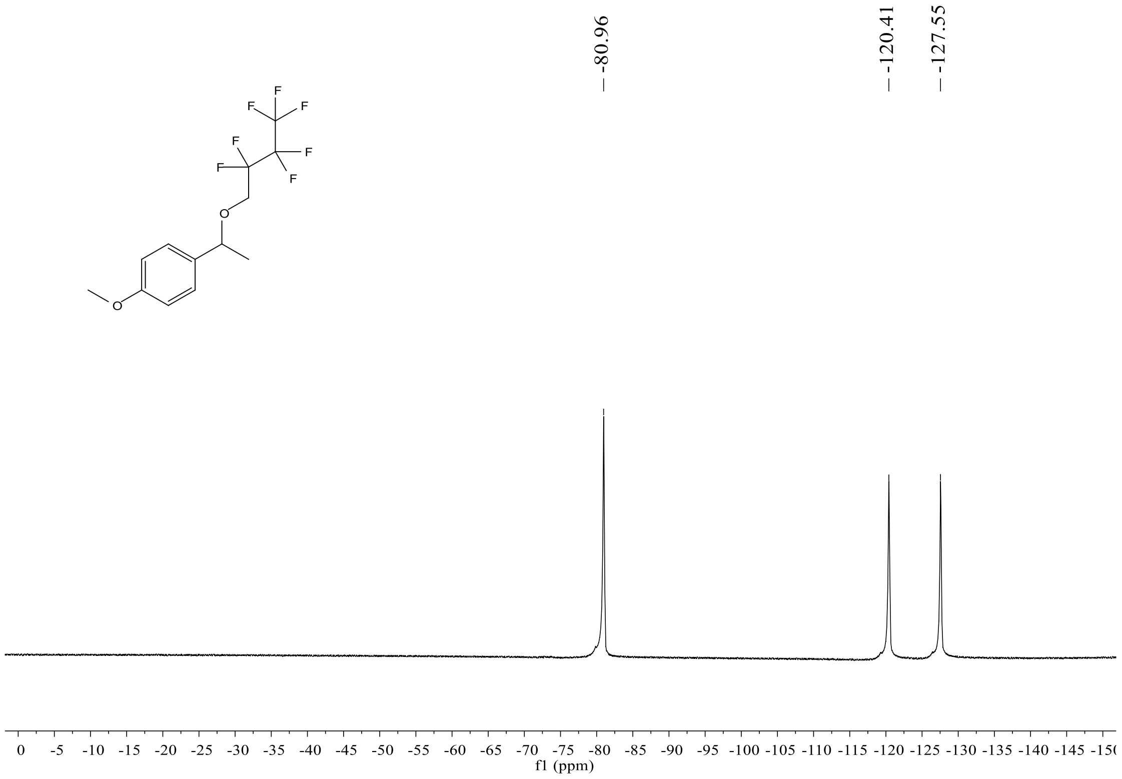

^{19}F NMR spectrum in $CDCl_3$.

69d

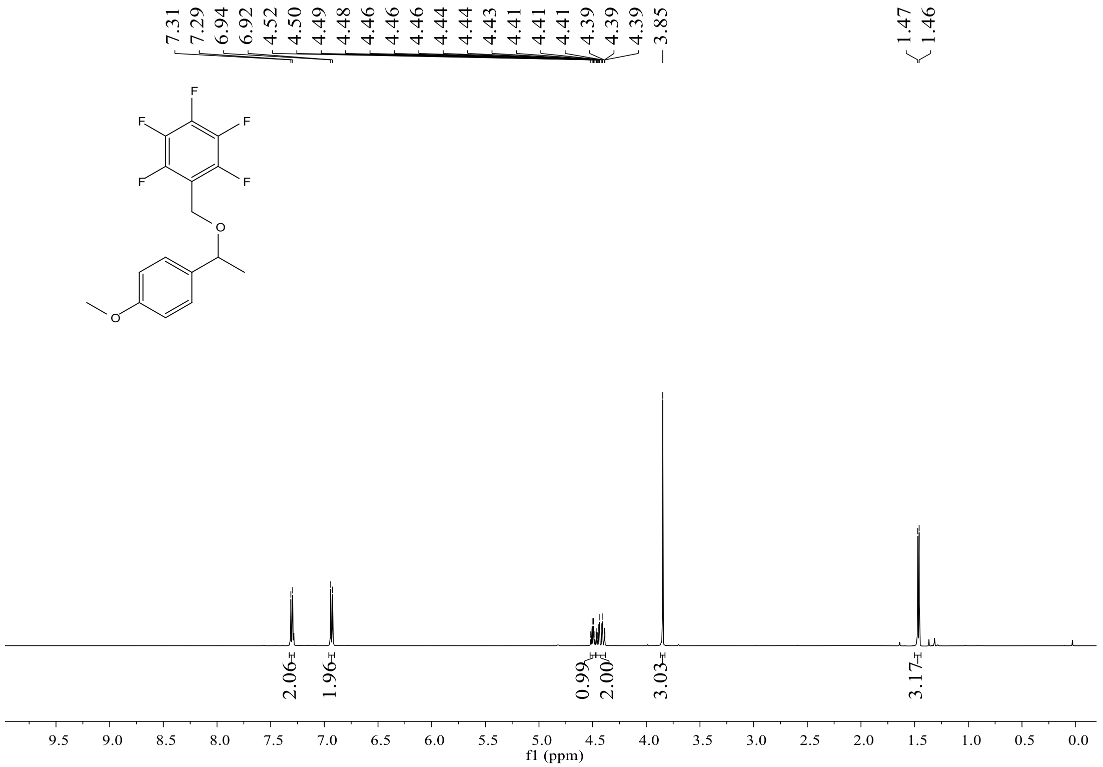

^{1}H NMR spectrum in $CDCl_3$.

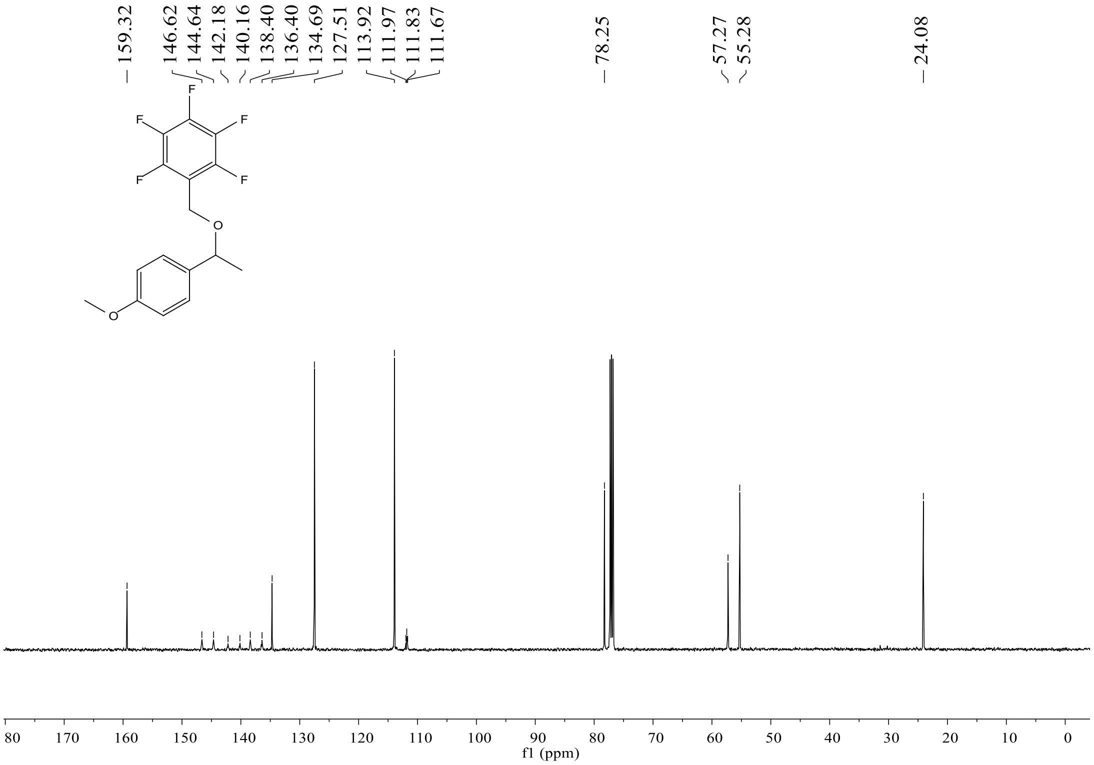

^{13}C NMR spectrum in $CDCl_3$.

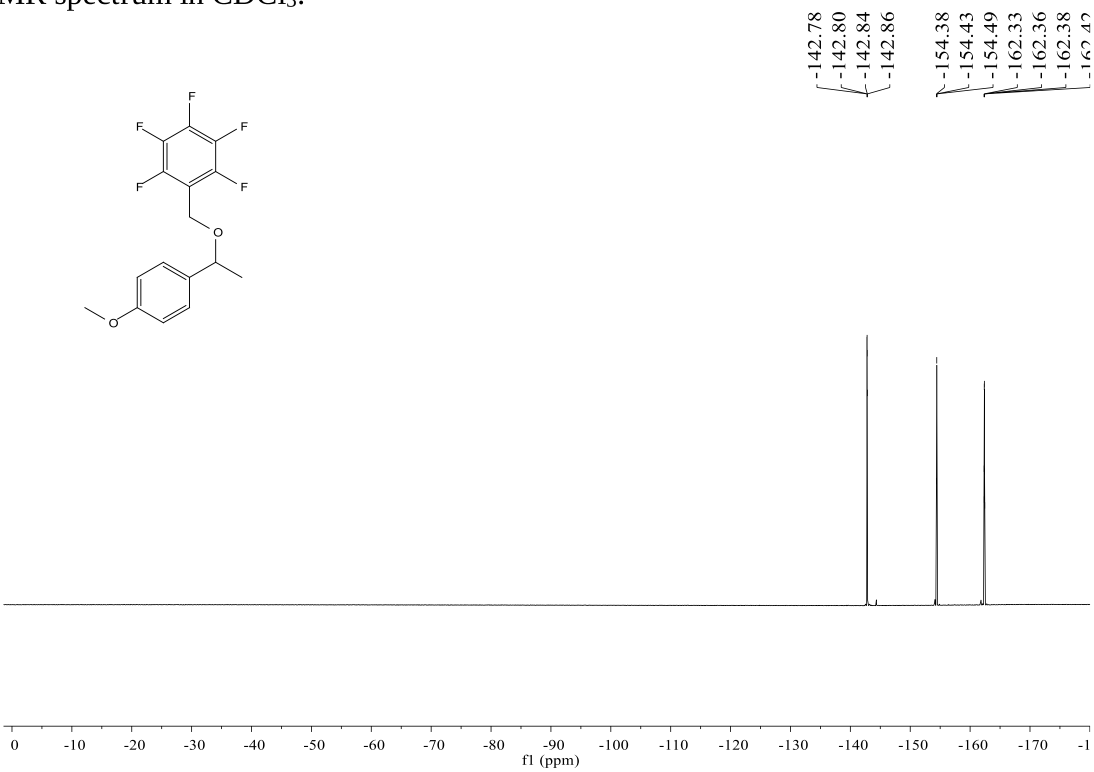

^{19}F NMR spectrum in $CDCl_3$.

70d

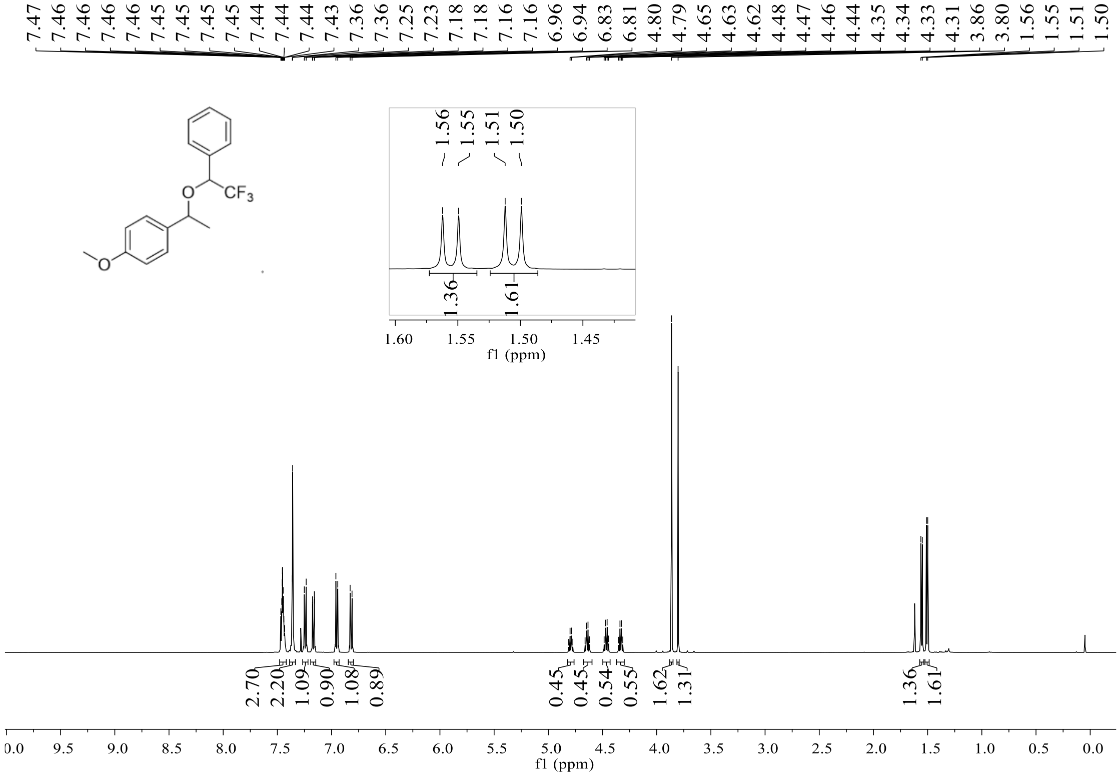

^{1}H NMR spectrum in $CDCl_3$.

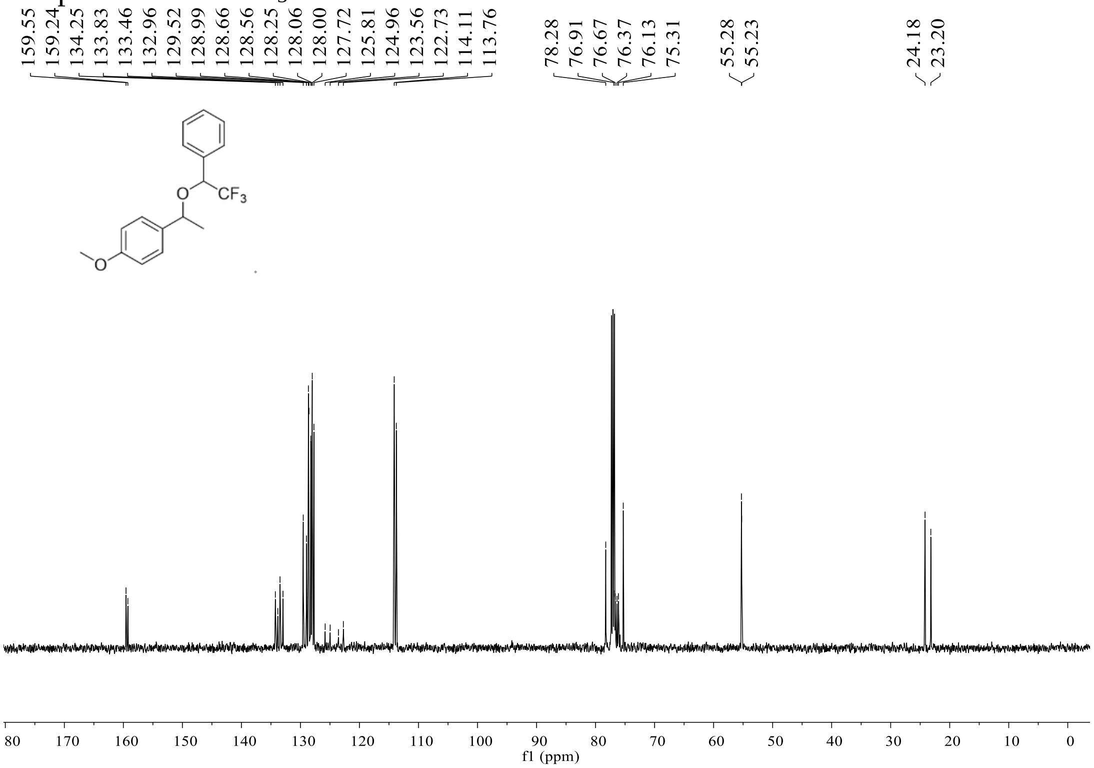

^{13}C NMR spectrum in $CDCl_3$.

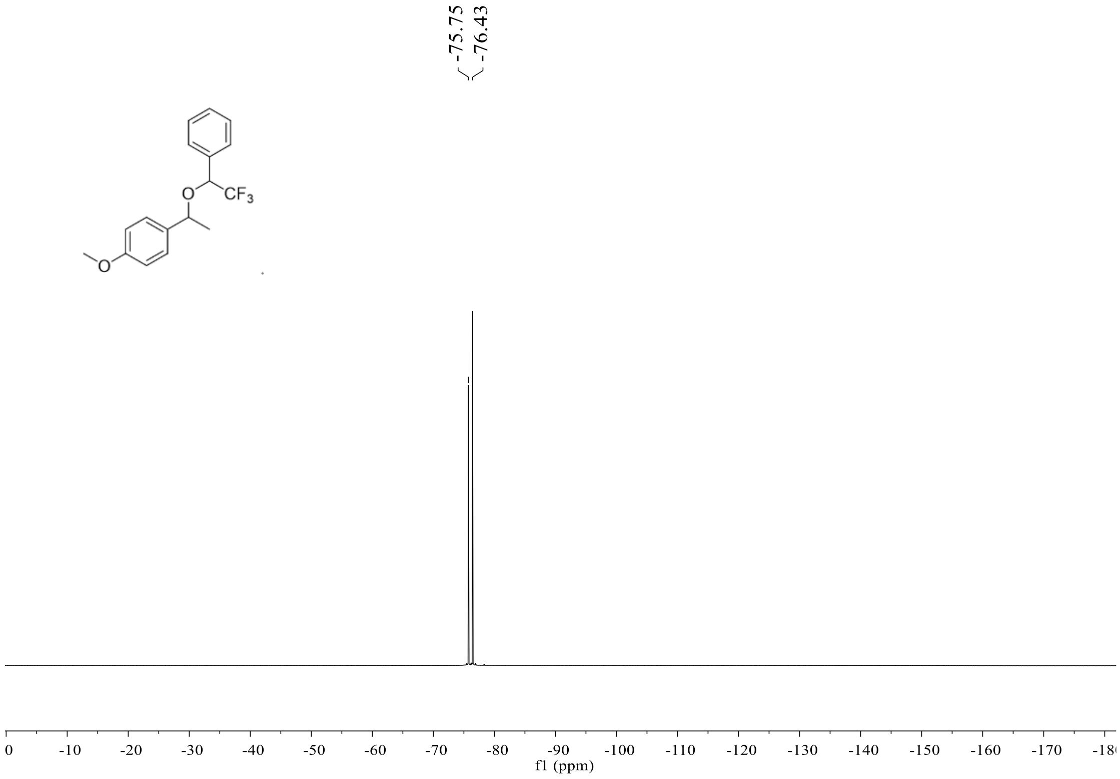

^{19}F NMR spectrum in $CDCl_3$.

71d

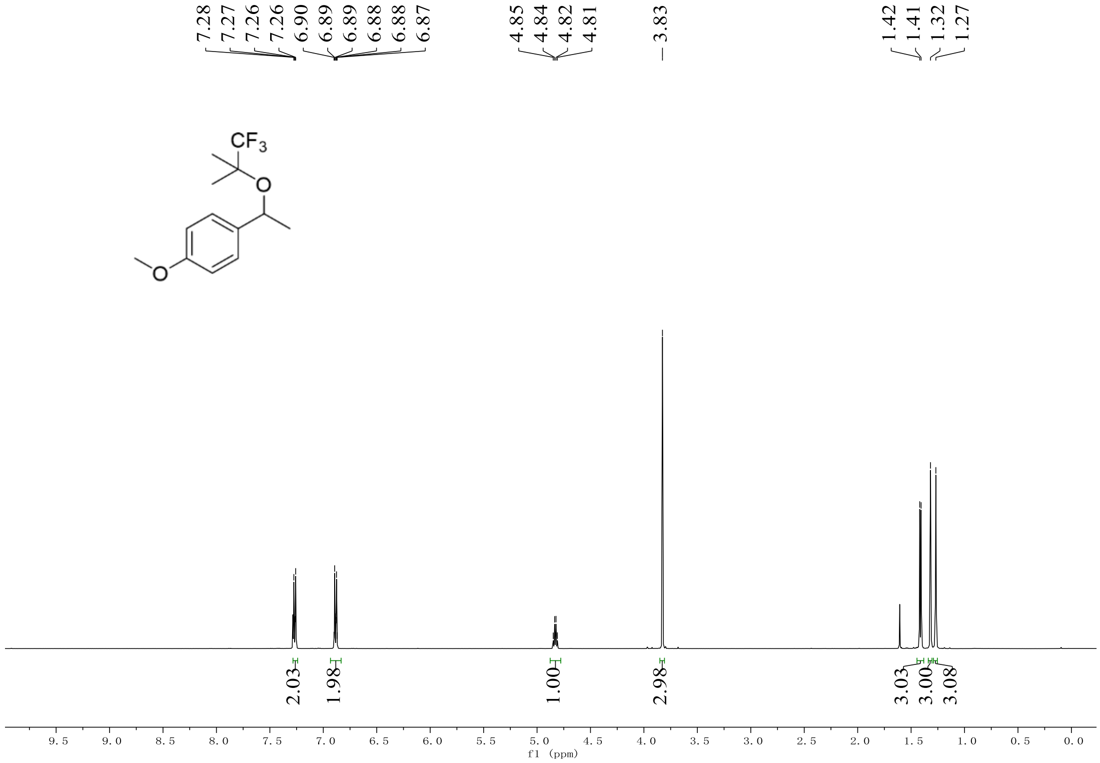

^{1}H NMR spectrum in $CDCl_3$.

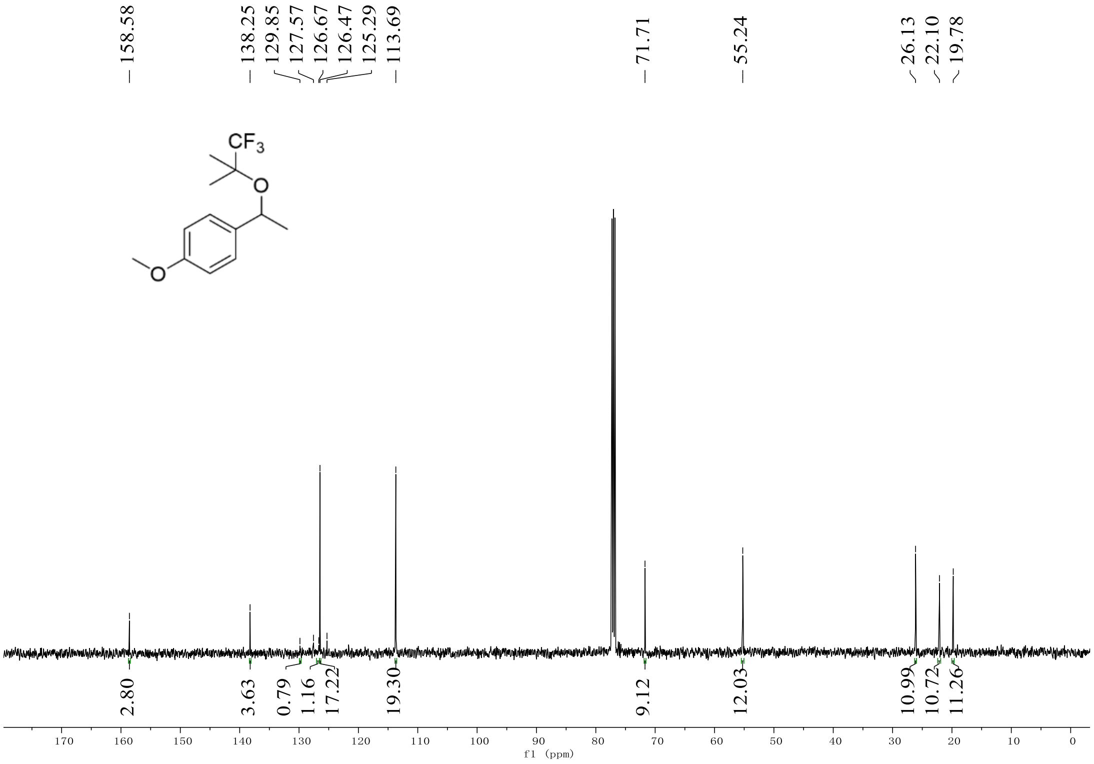

^{13}C NMR spectrum in $CDCl_3$.

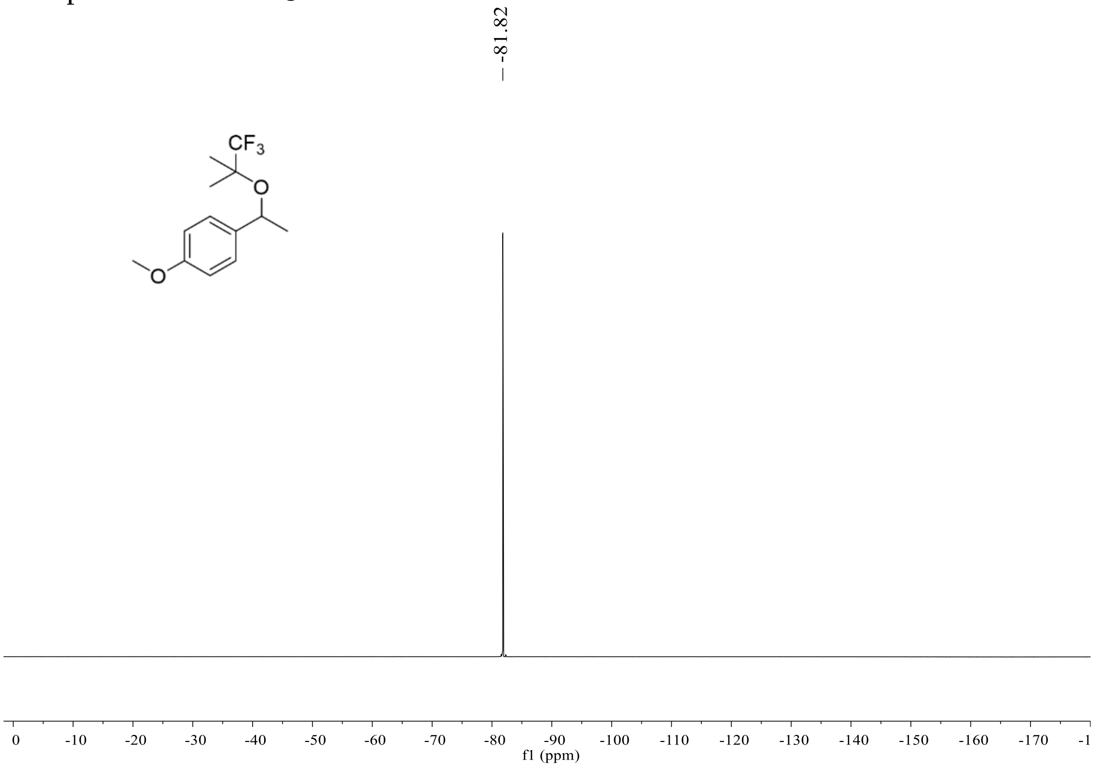

^{19}F NMR spectrum in $CDCl_3$.

72d

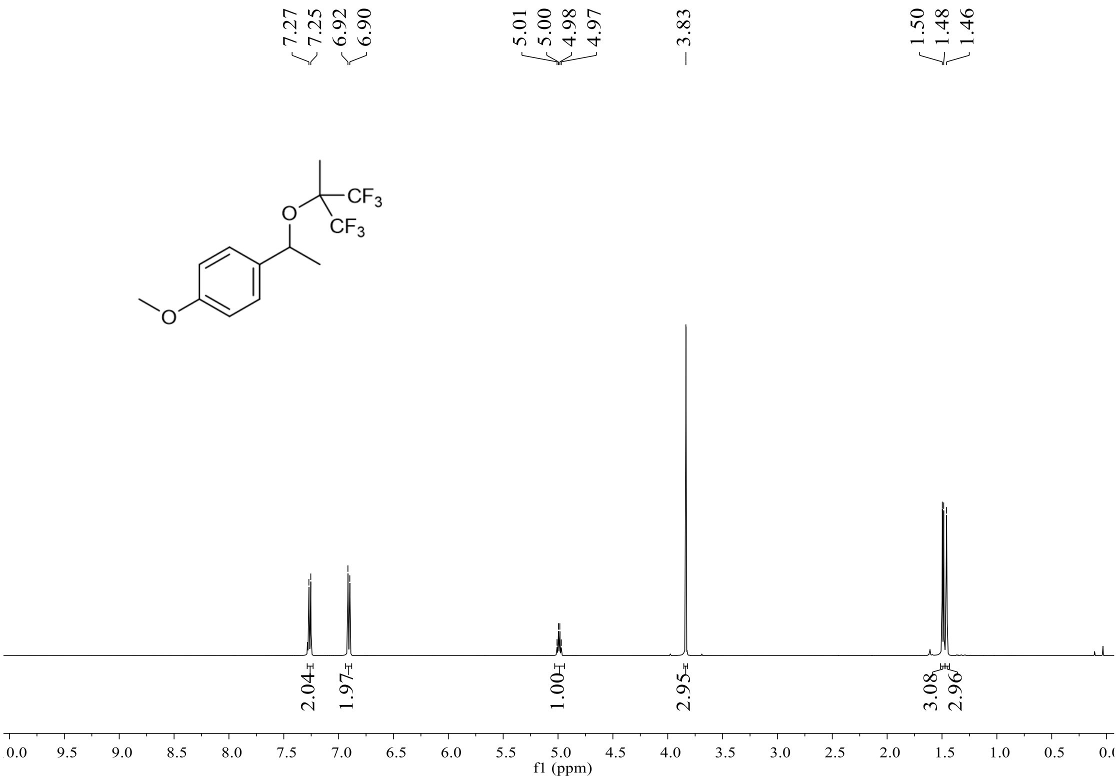

^{1}H NMR spectrum in $CDCl_3$.

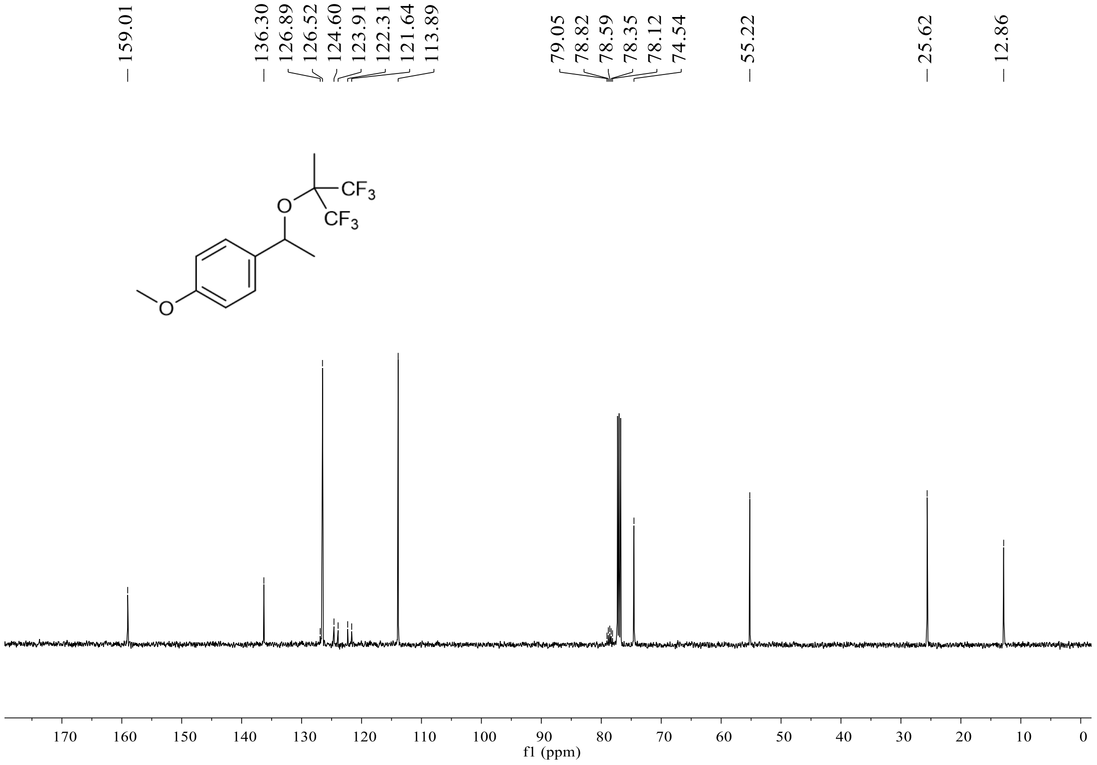

^{13}C NMR spectrum in $CDCl_3$.

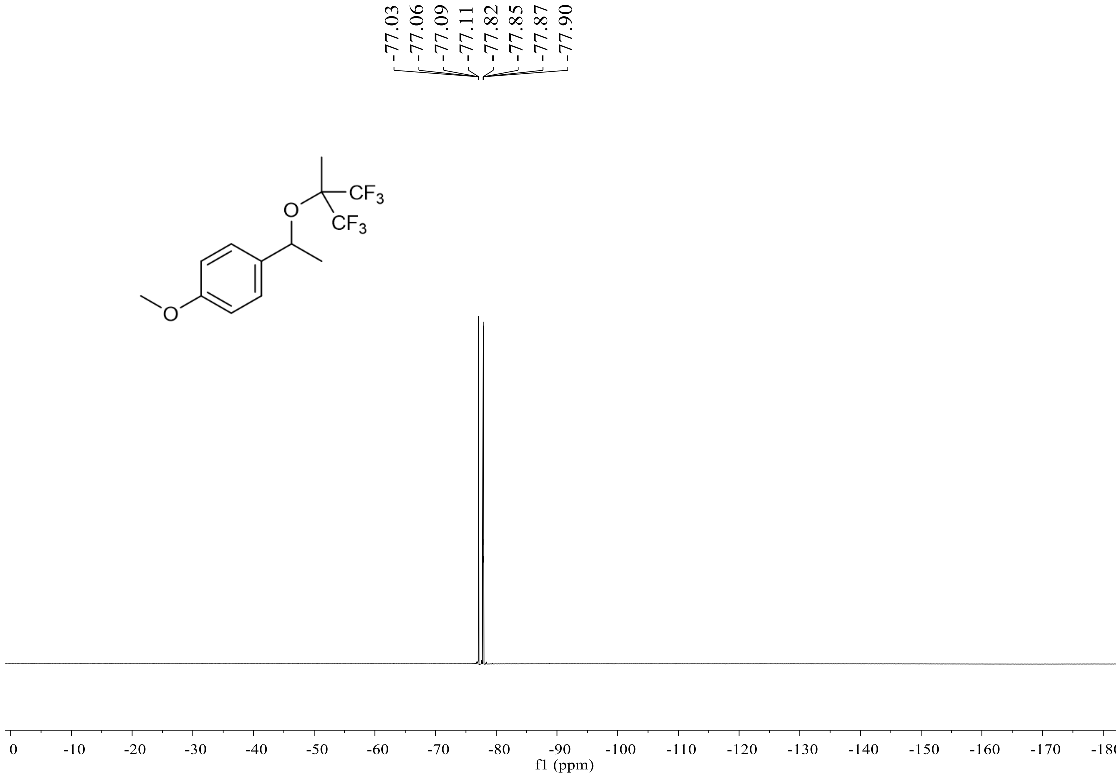

^{19}F NMR spectrum in $CDCl_3$.

73d

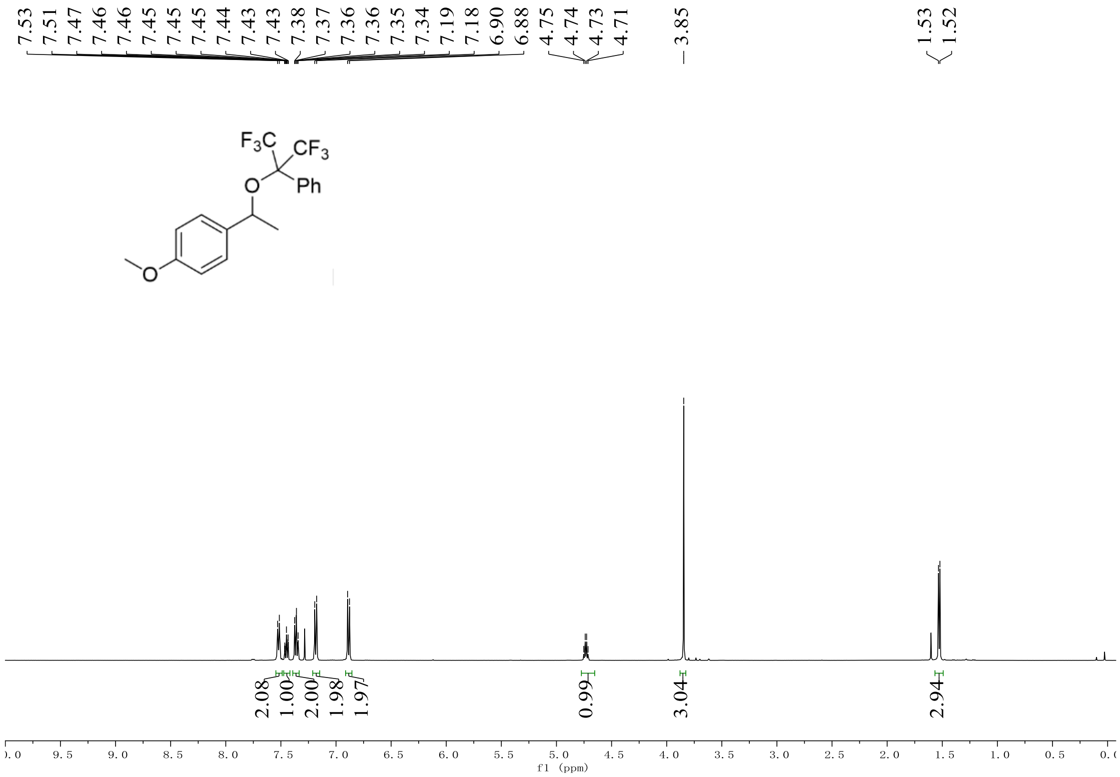

^{1}H NMR spectrum in $CDCl_3$.

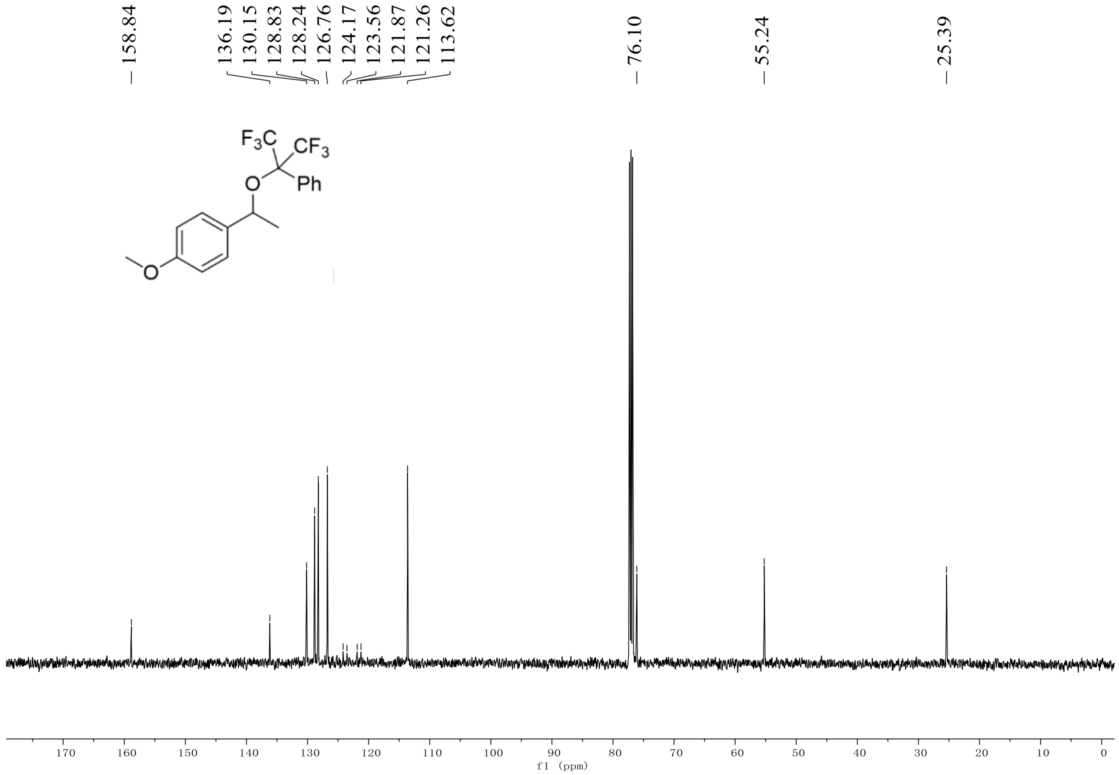

^{13}C NMR spectrum in $CDCl_3$.

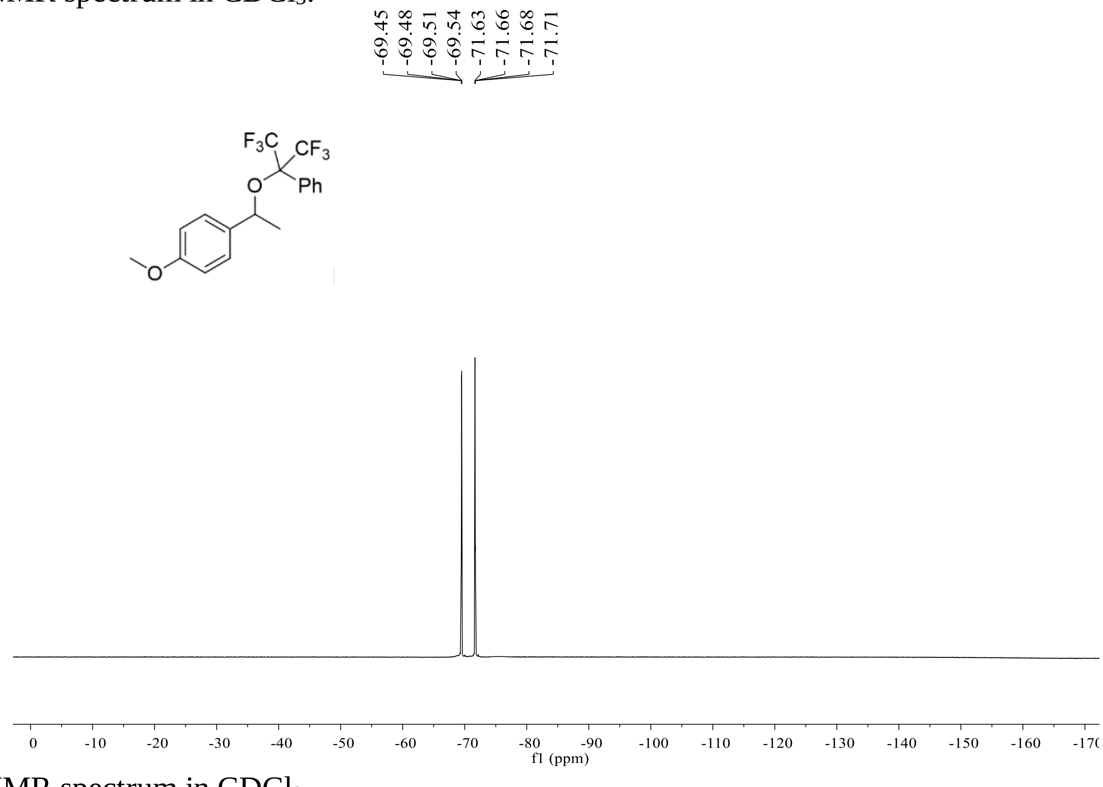

^{19}F NMR spectrum in $CDCl_3$.

74d

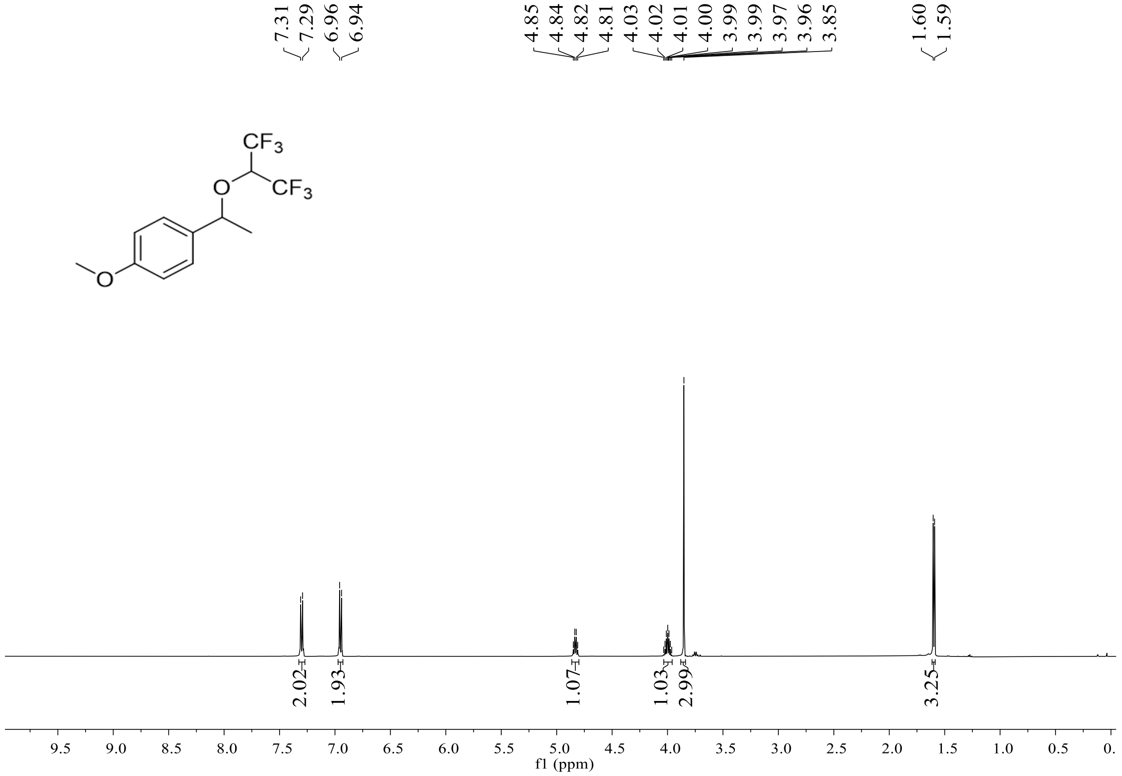

^{1}H NMR spectrum in $CDCl_3$.

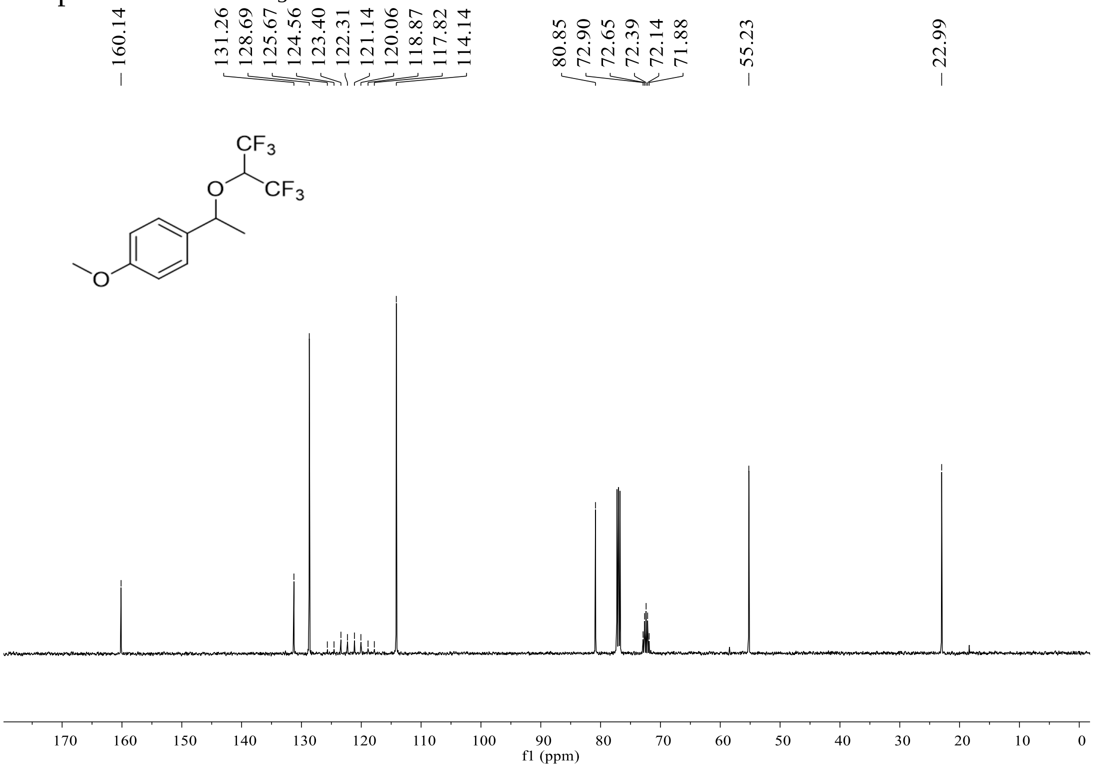

^{13}C NMR spectrum in $CDCl_3$.

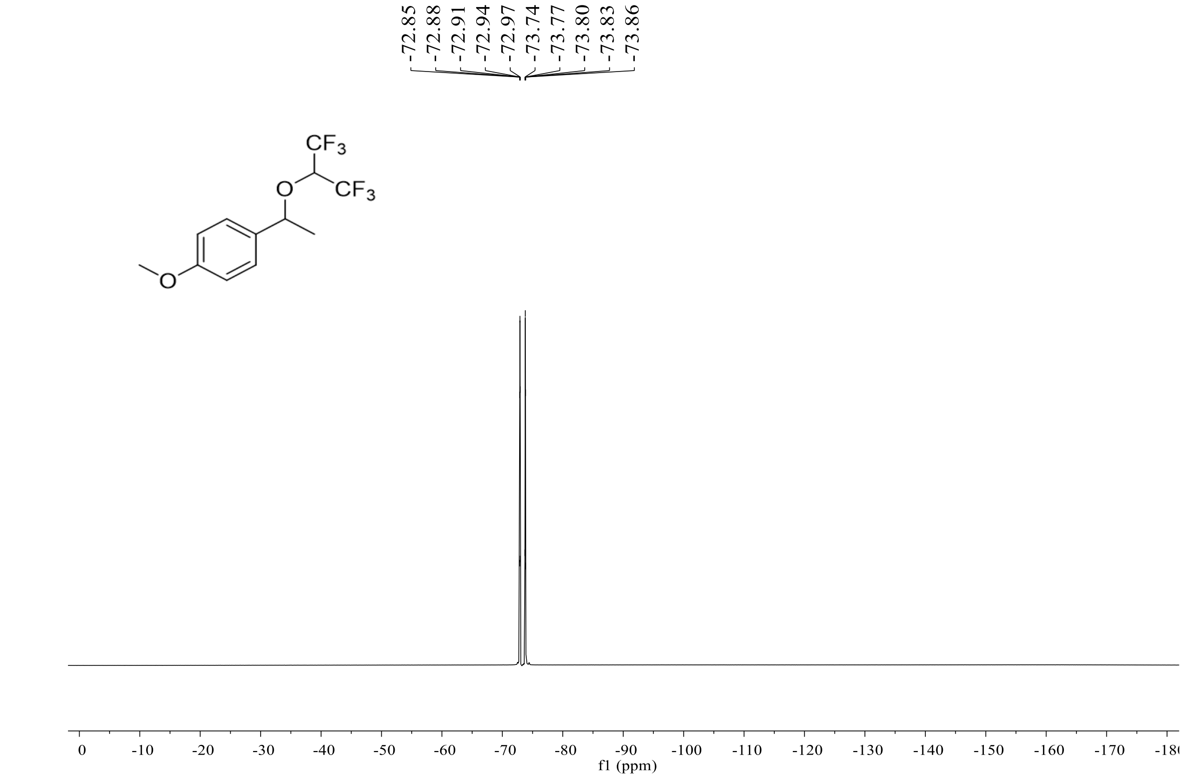

^{19}F NMR spectrum in $CDCl_3$.

74e

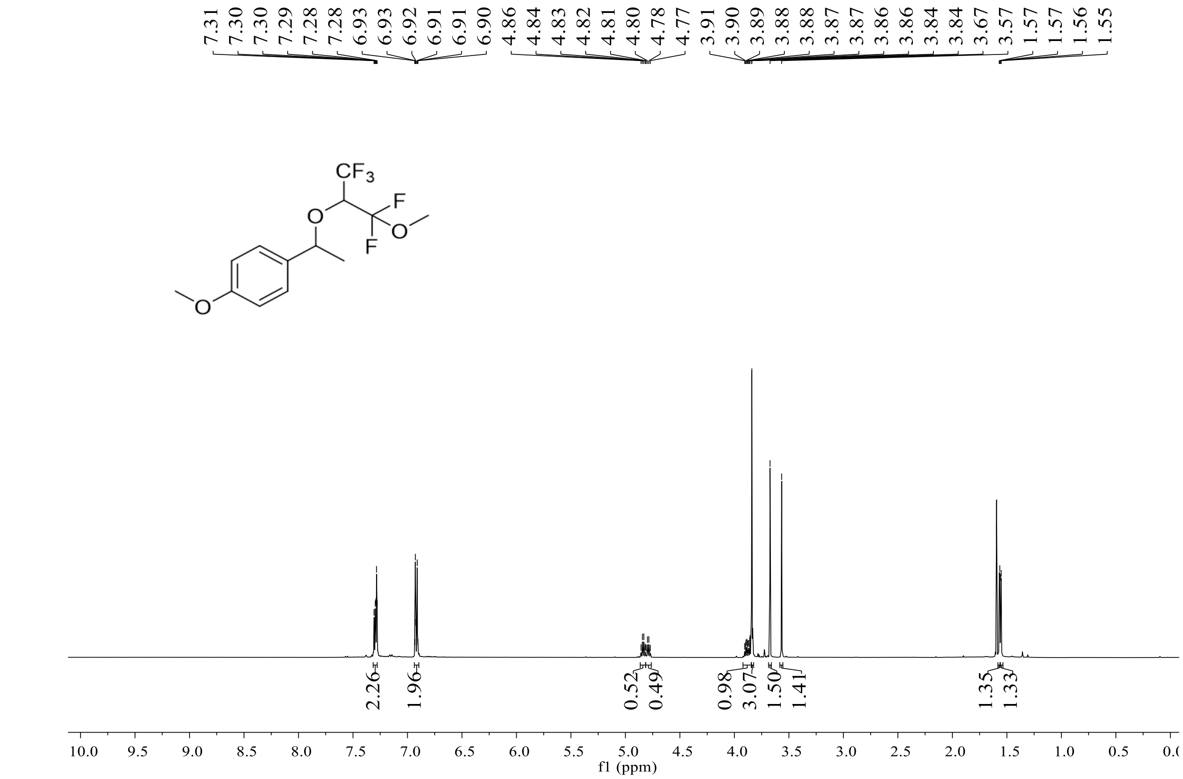

^{1}H NMR spectrum in $CDCl_3$.

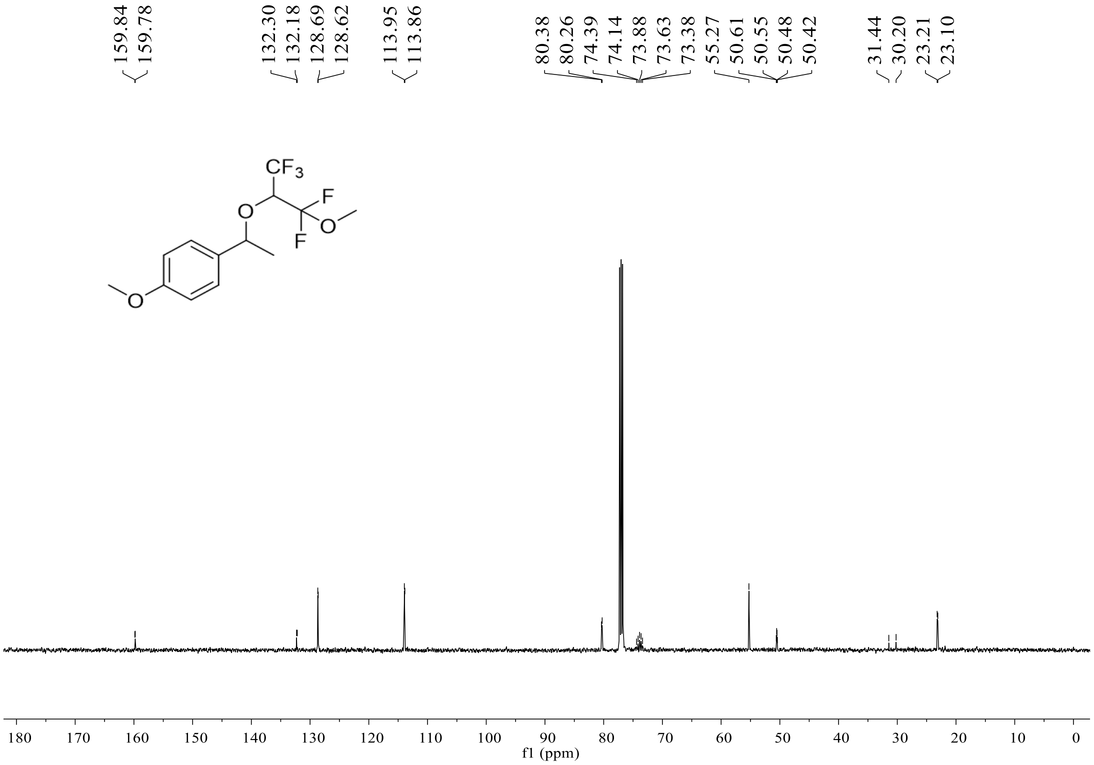

^{13}C NMR spectrum in $CDCl_3$.

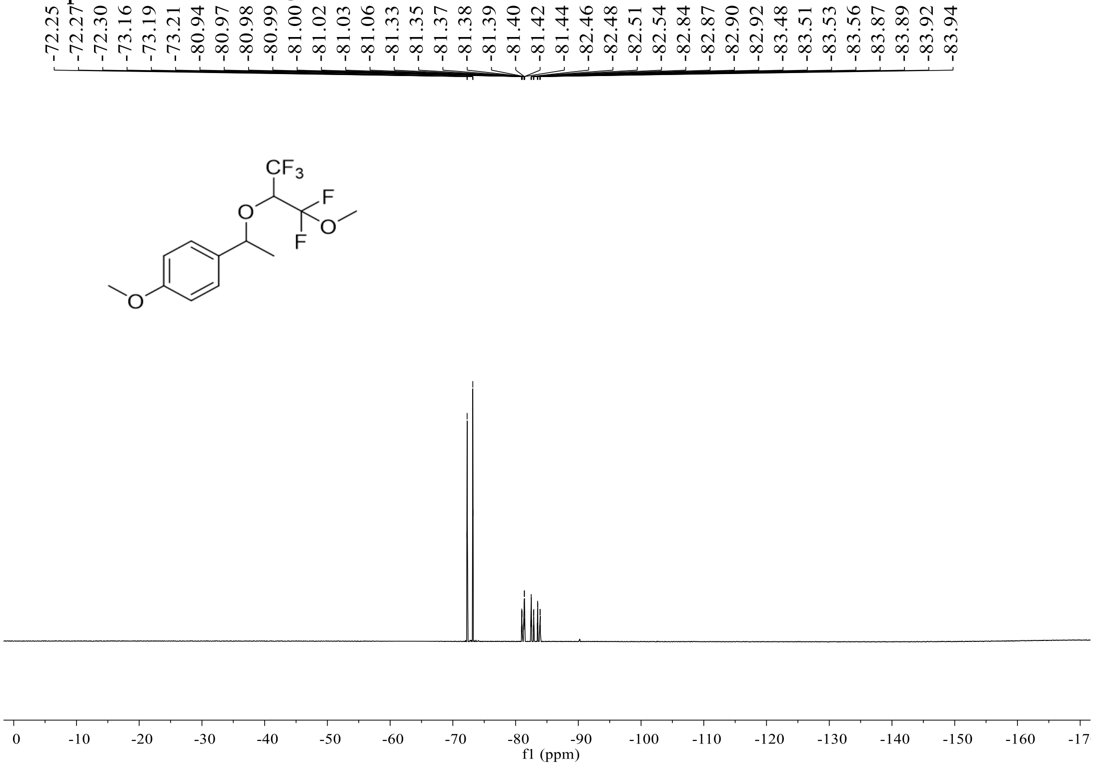

^{19}F NMR spectrum in $CDCl_3$.

75d

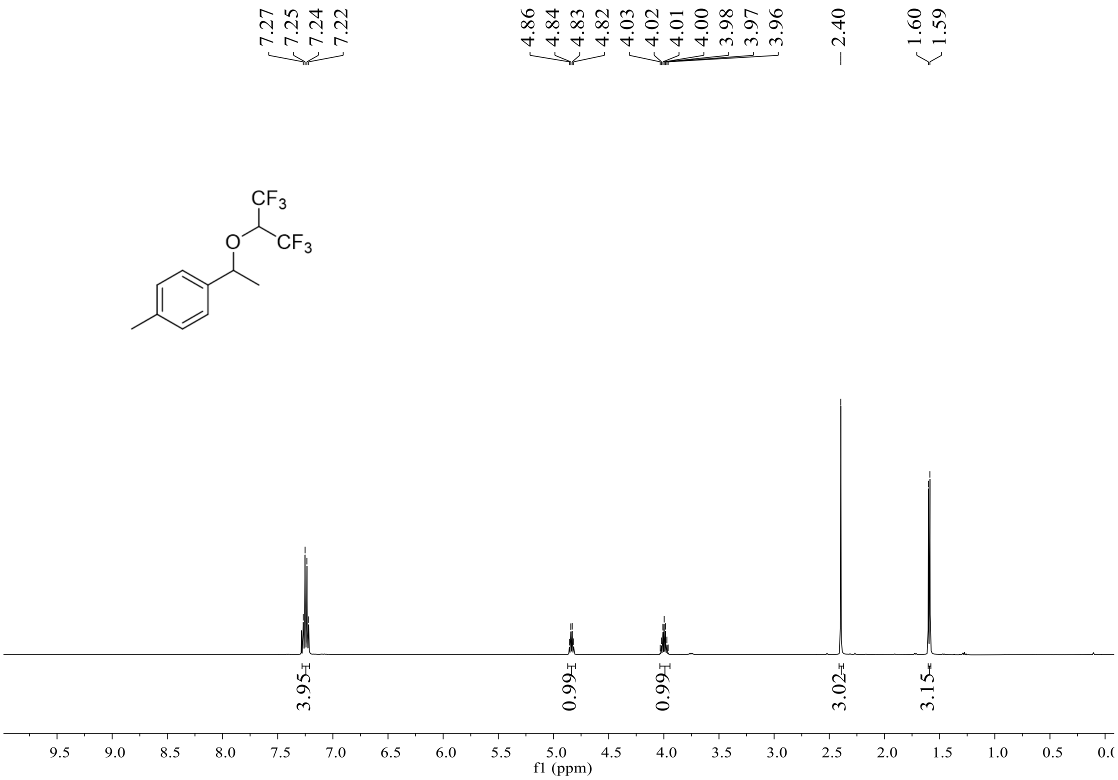

^{1}H NMR spectrum in $CDCl_3$.

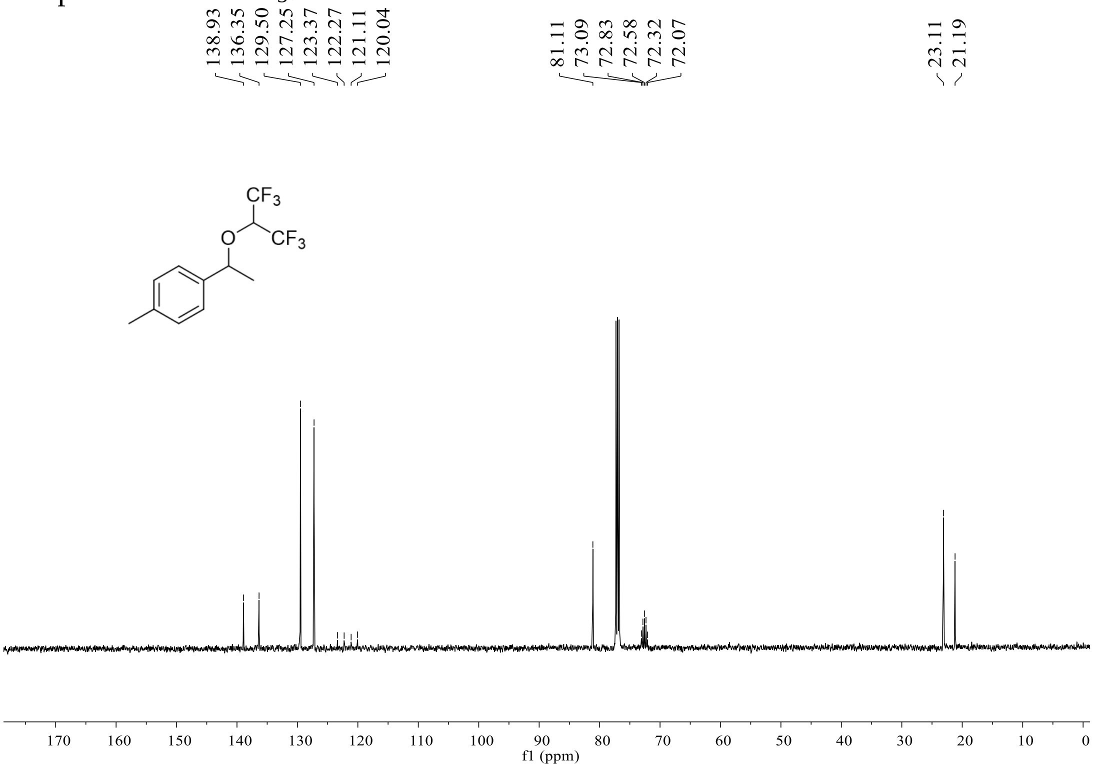

^{13}C NMR spectrum in $CDCl_3$.

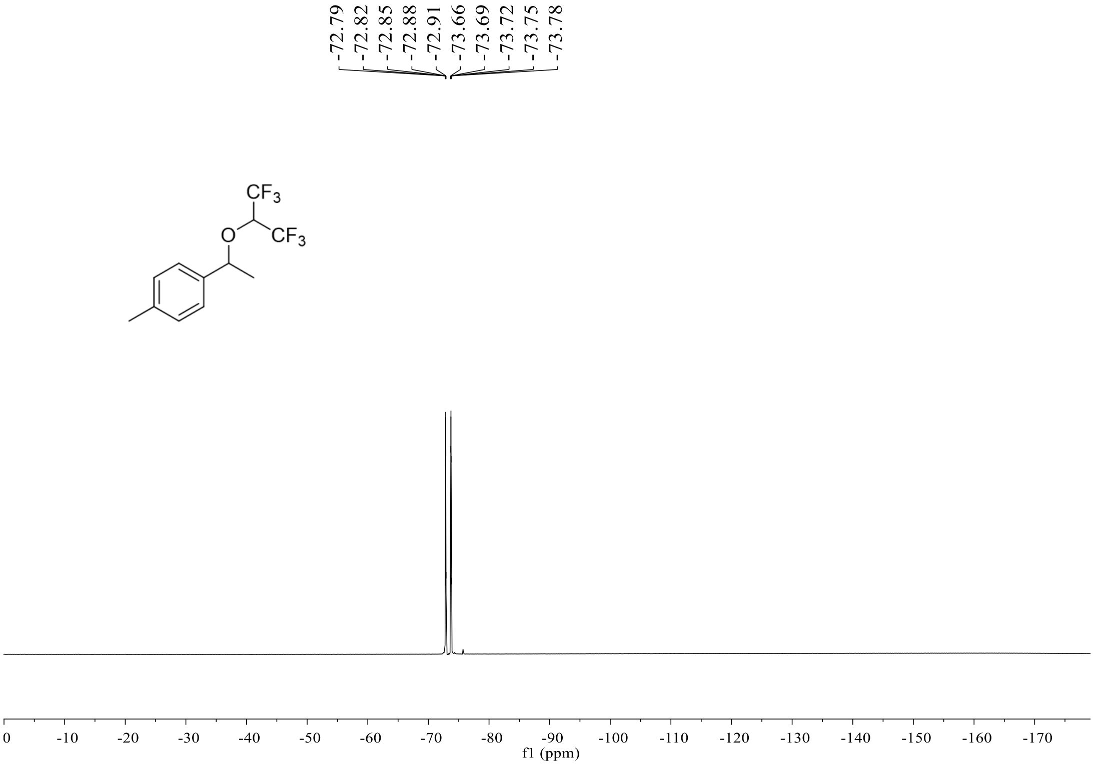

^{19}F NMR spectrum in $CDCl_3$.

76d

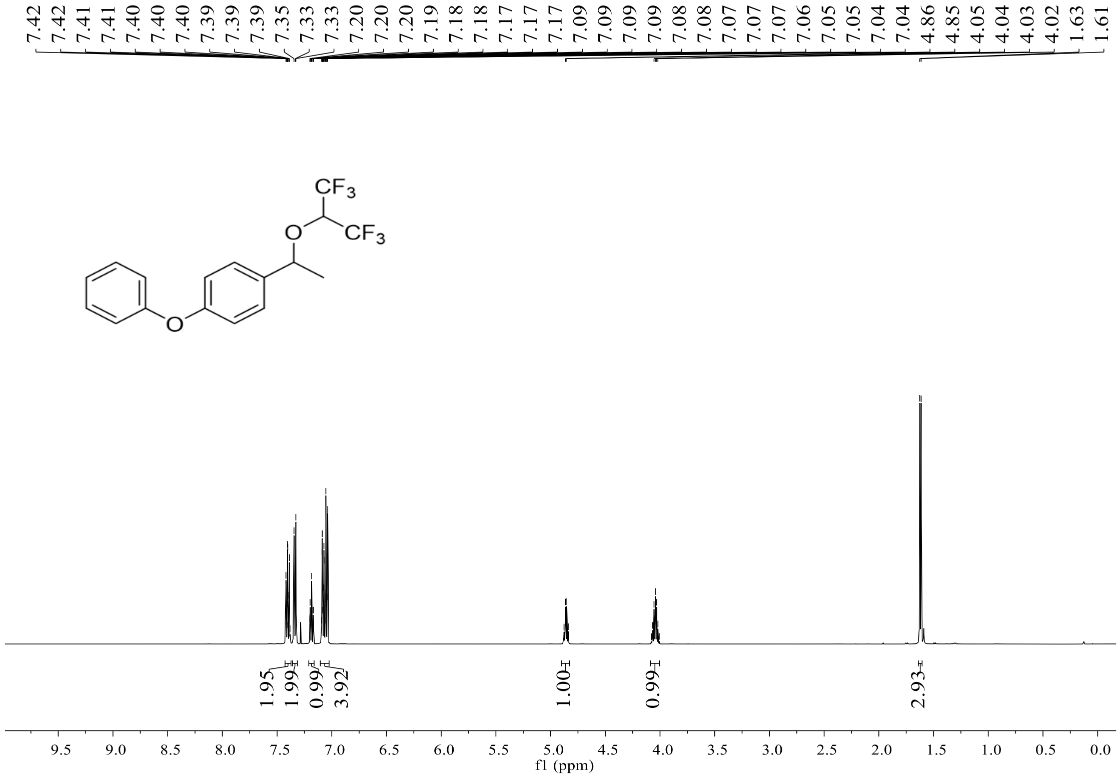

^{1}H NMR spectrum in $CDCl_3$.

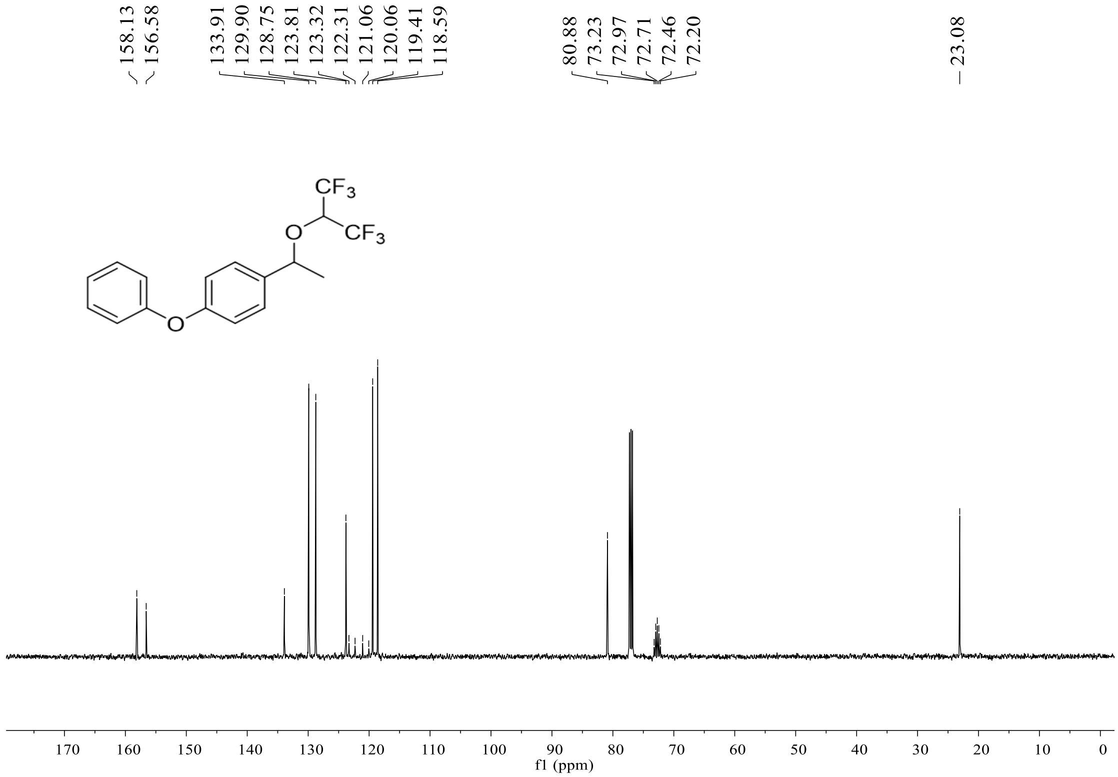

^{13}C NMR spectrum in $CDCl_3$.

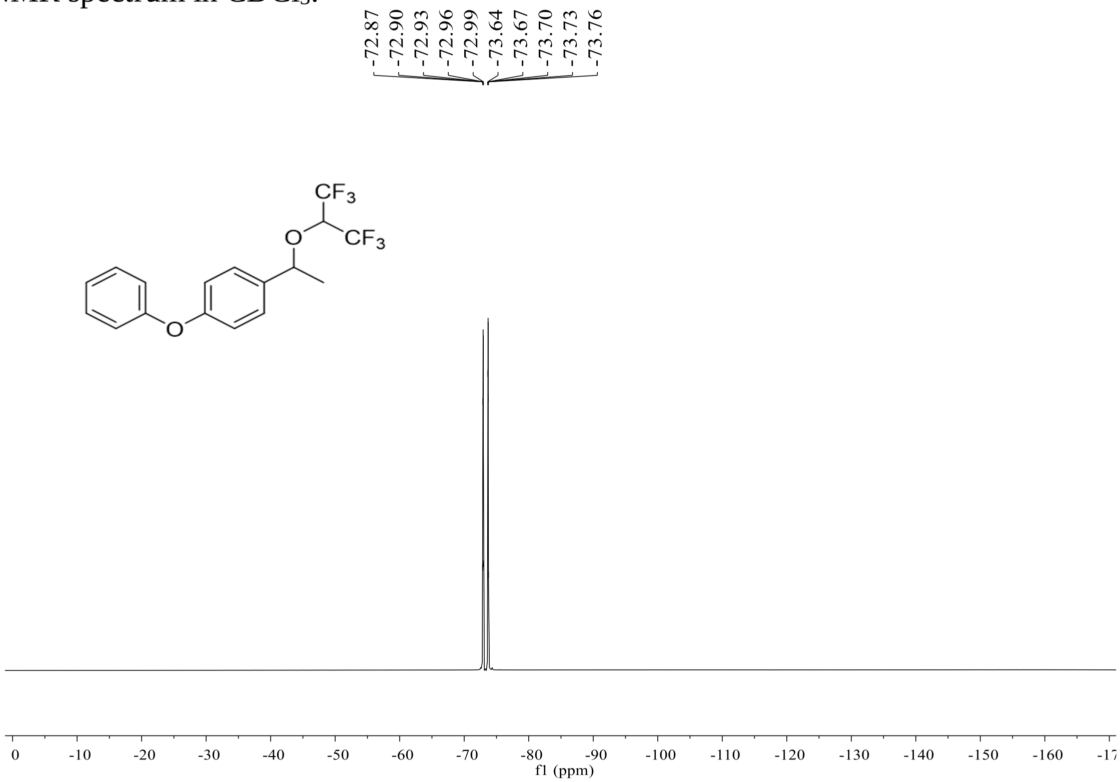

^{19}F NMR spectrum in $CDCl_3$.

77d

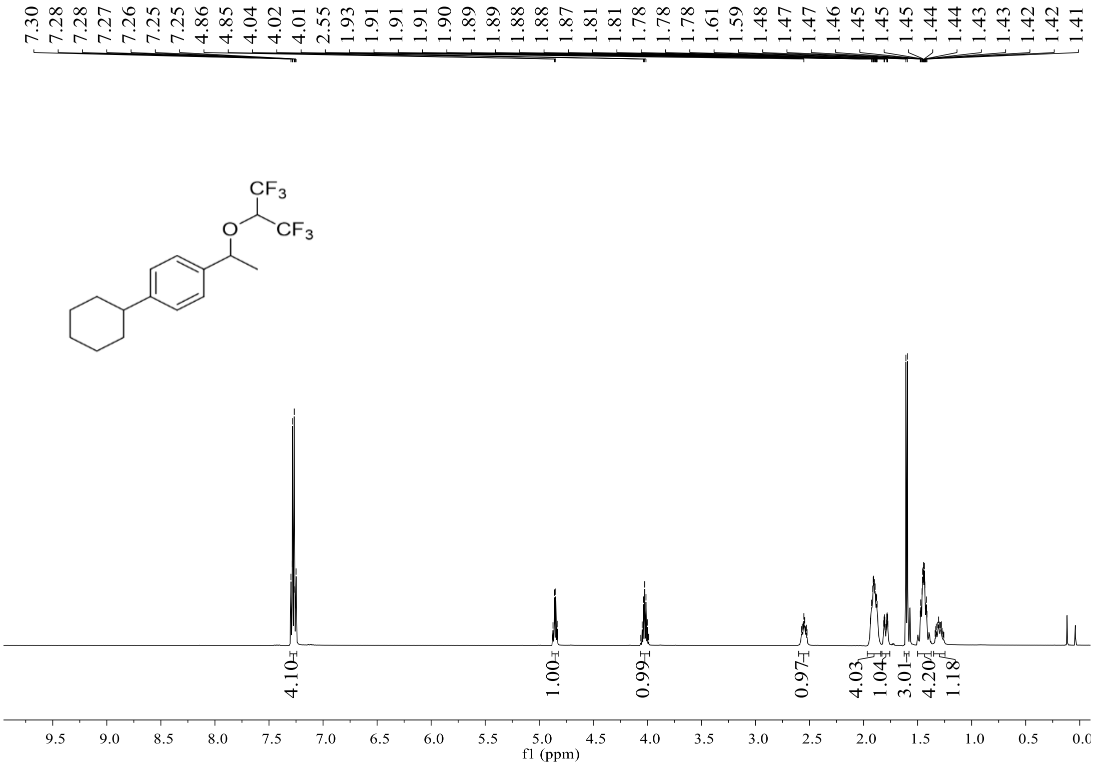

^{1}H NMR spectrum in $CDCl_3$.

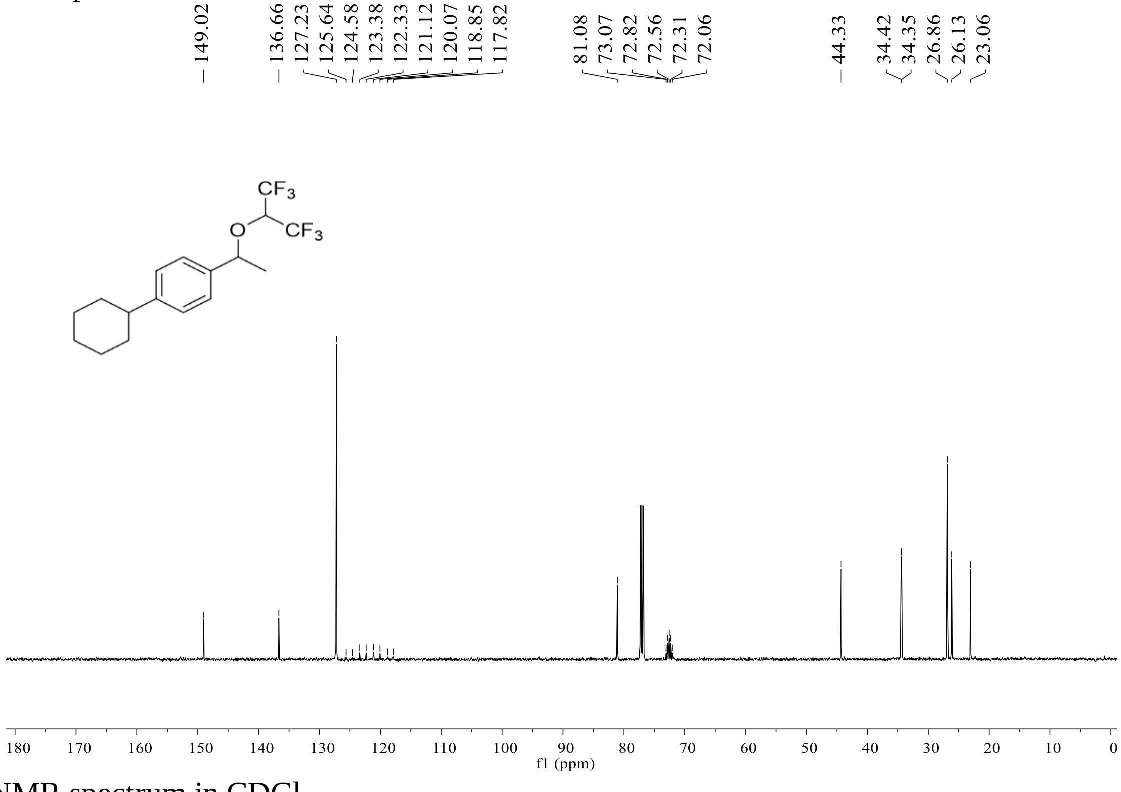

^{13}C NMR spectrum in $CDCl_3$.

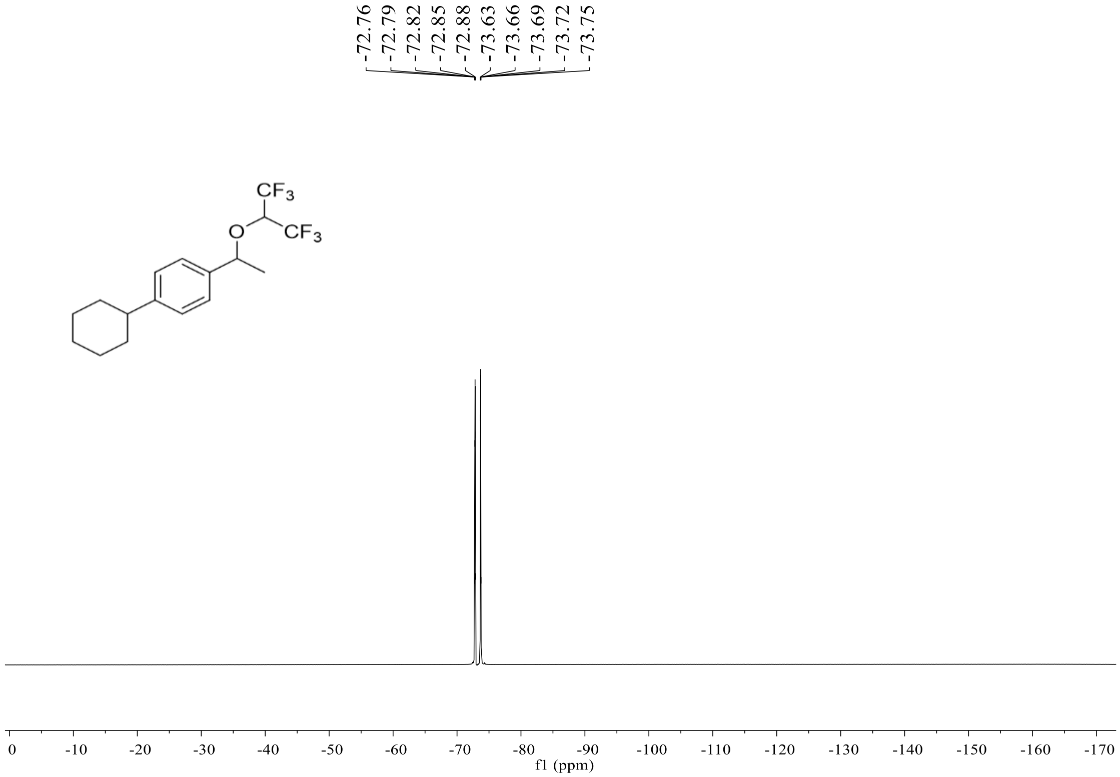

^{19}F NMR spectrum in $CDCl_3$.

78d

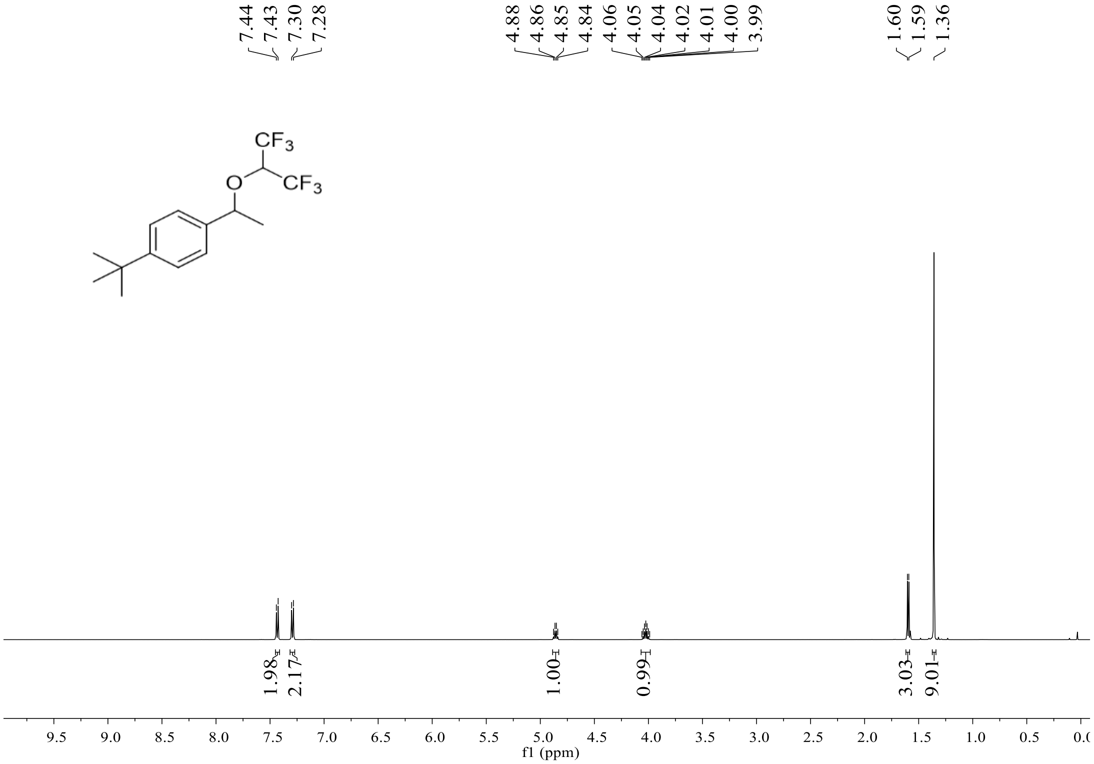

^{1}H NMR spectrum in $CDCl_3$.

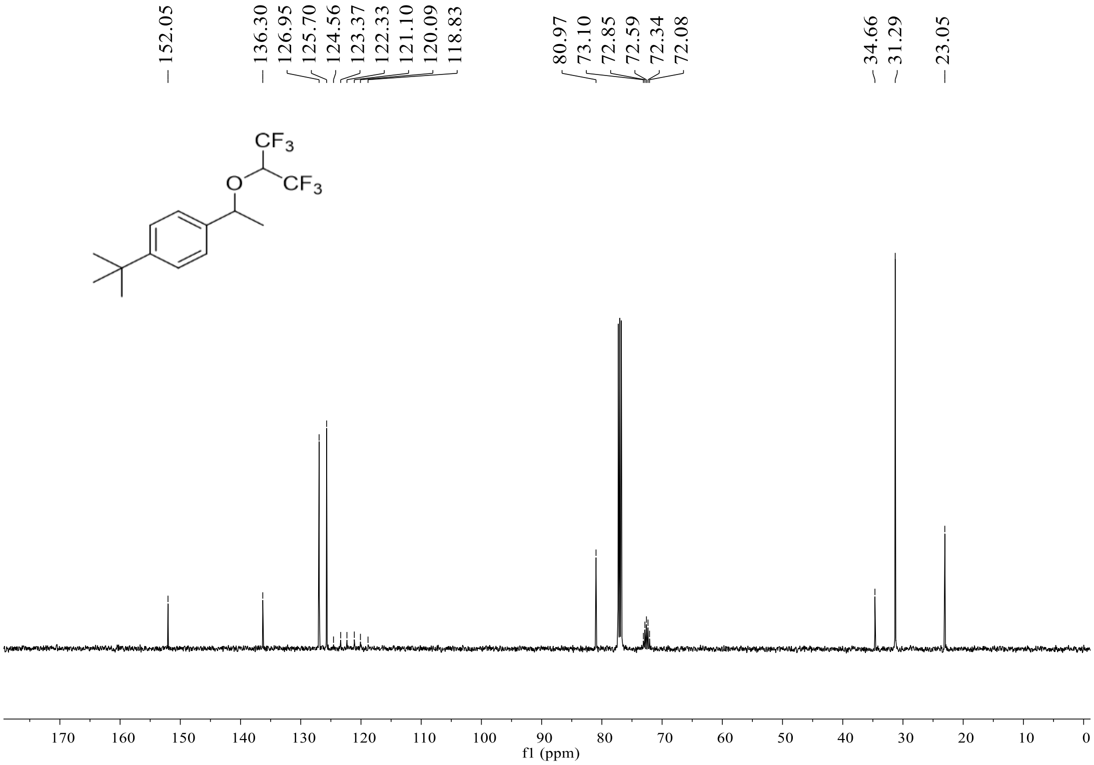

^{13}C NMR spectrum in $CDCl_3$.

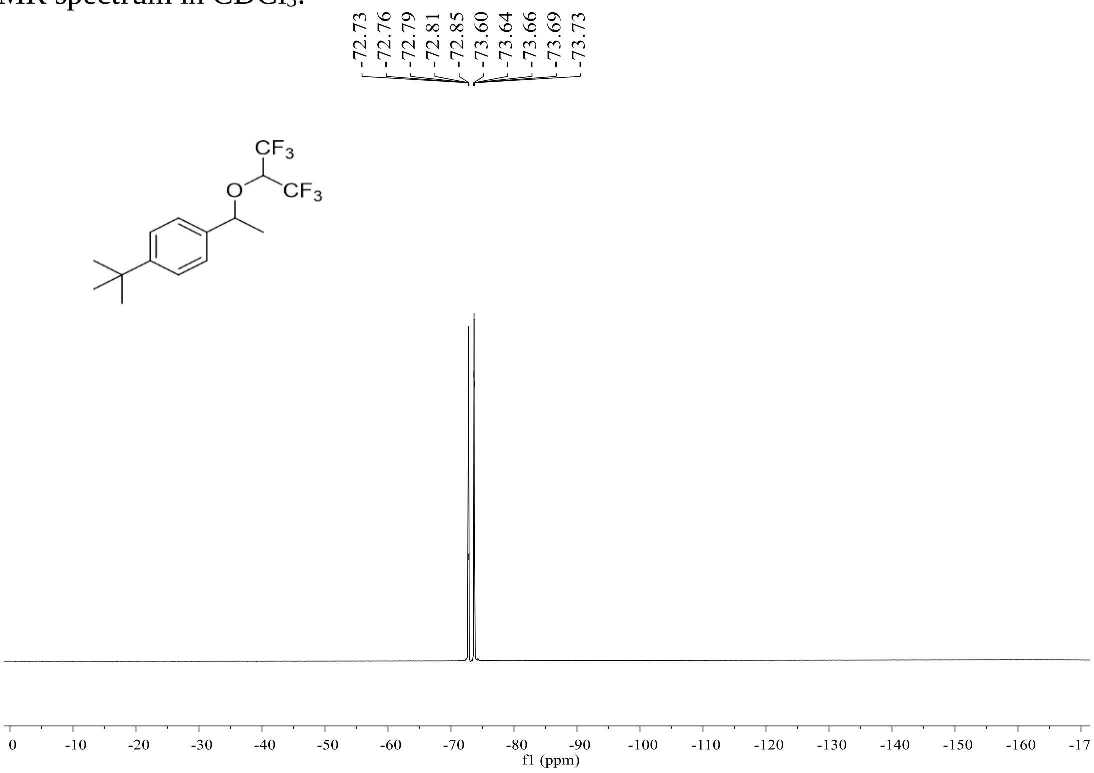

^{19}F NMR spectrum in $CDCl_3$.

79d

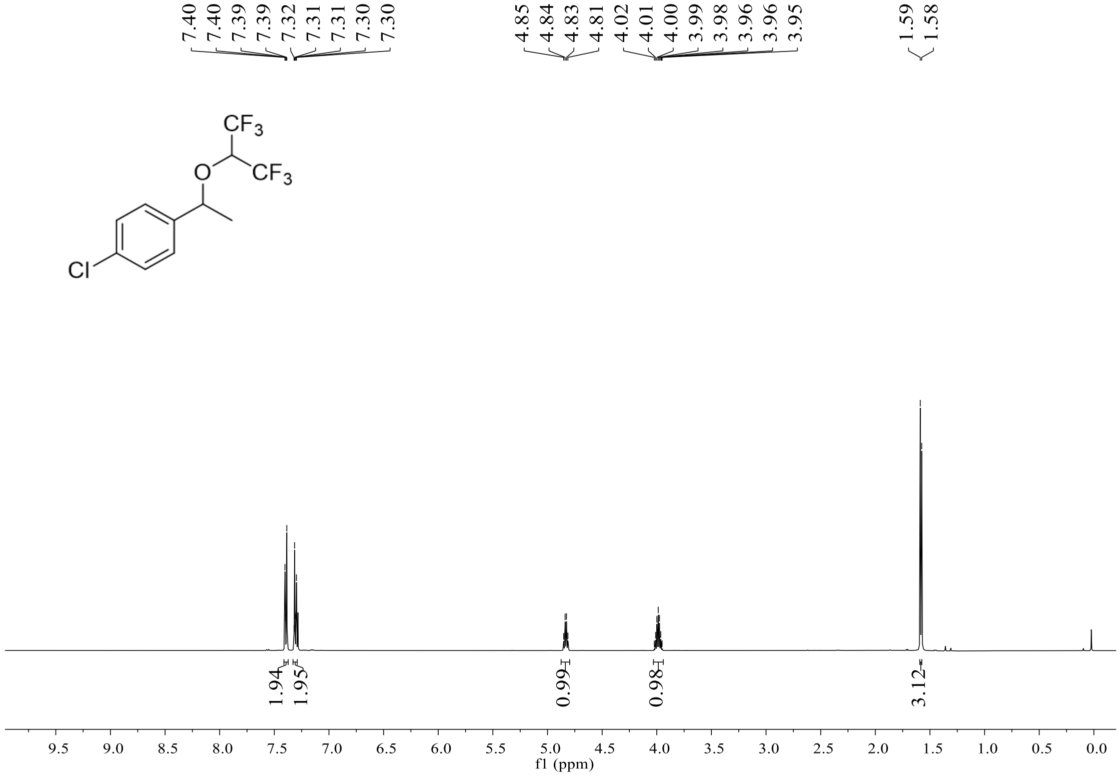

^{1}H NMR spectrum in $CDCl_3$.

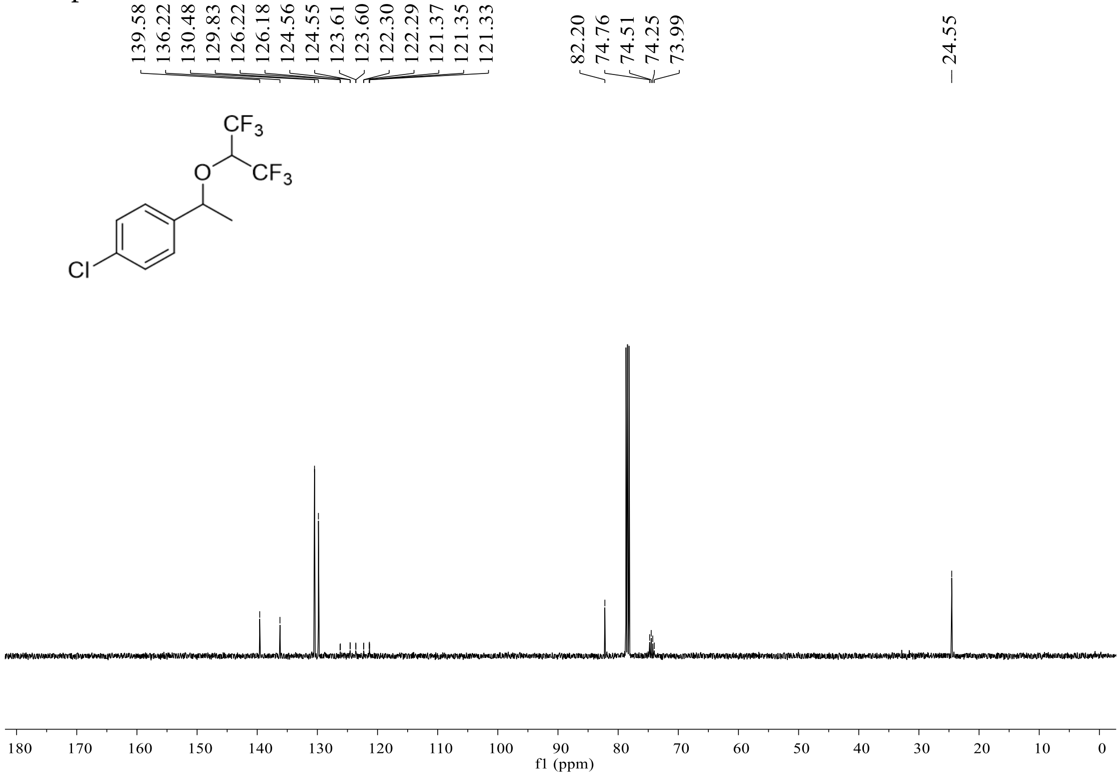

^{13}C NMR spectrum in $CDCl_3$.

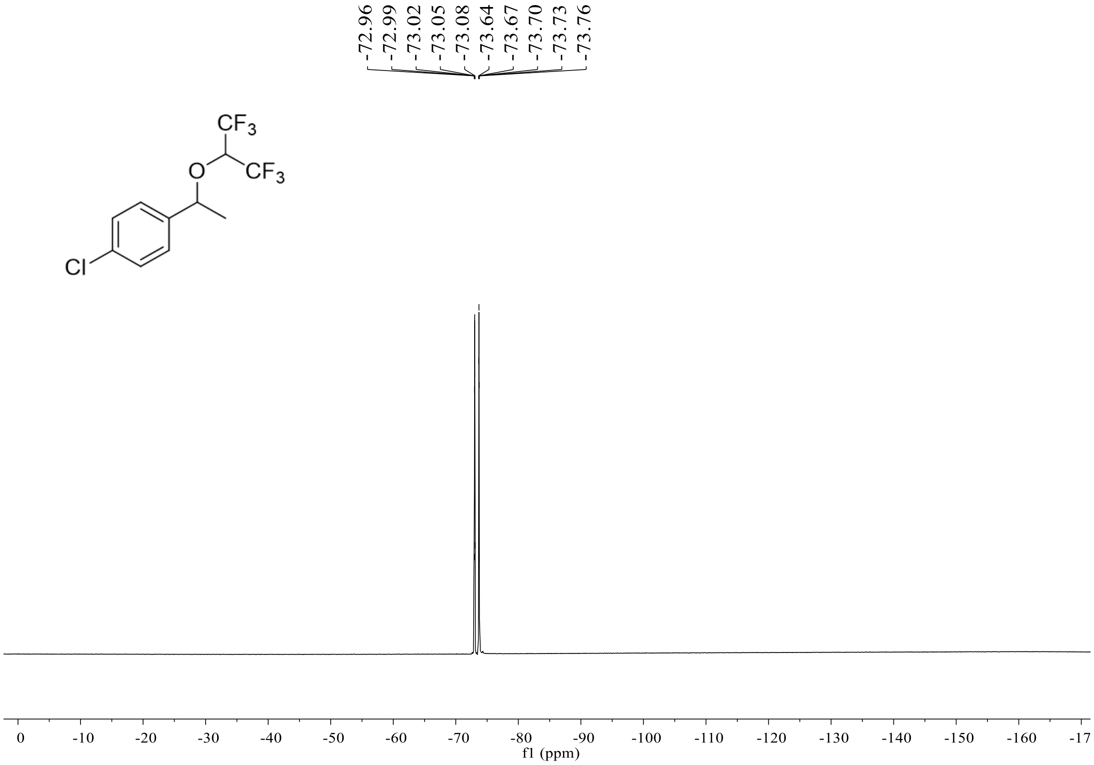

^{19}F NMR spectrum in $CDCl_3$.

80d

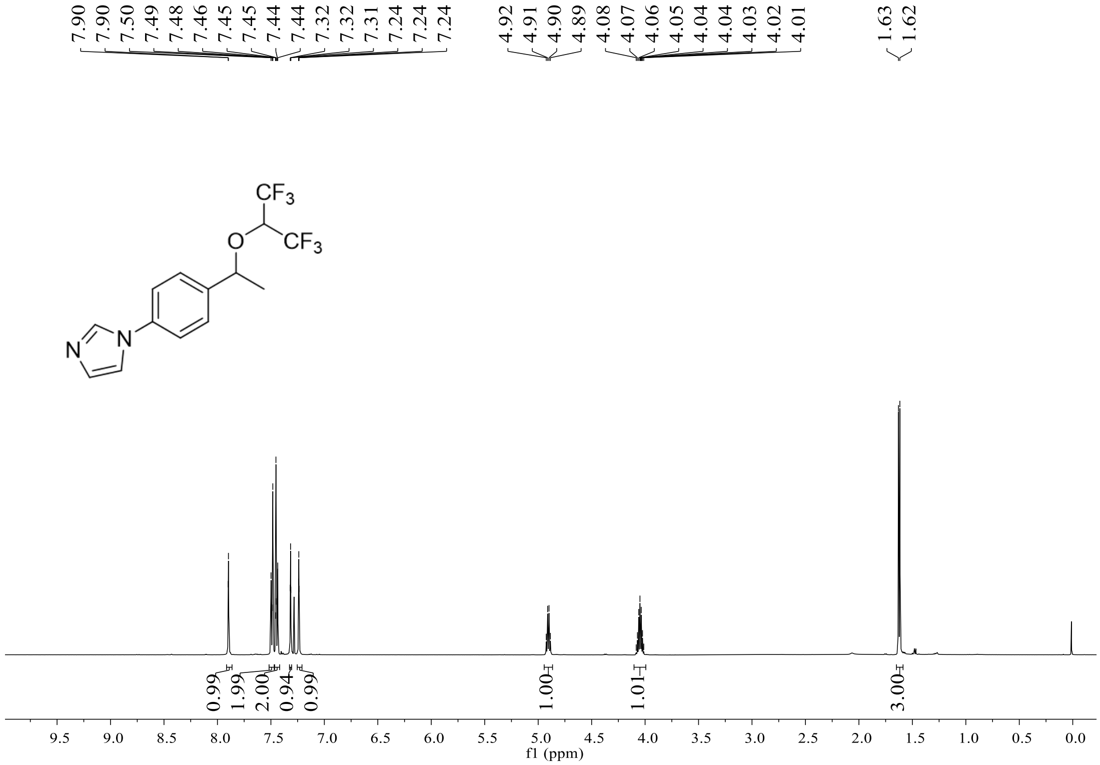

^{1}H NMR spectrum in $CDCl_3$.

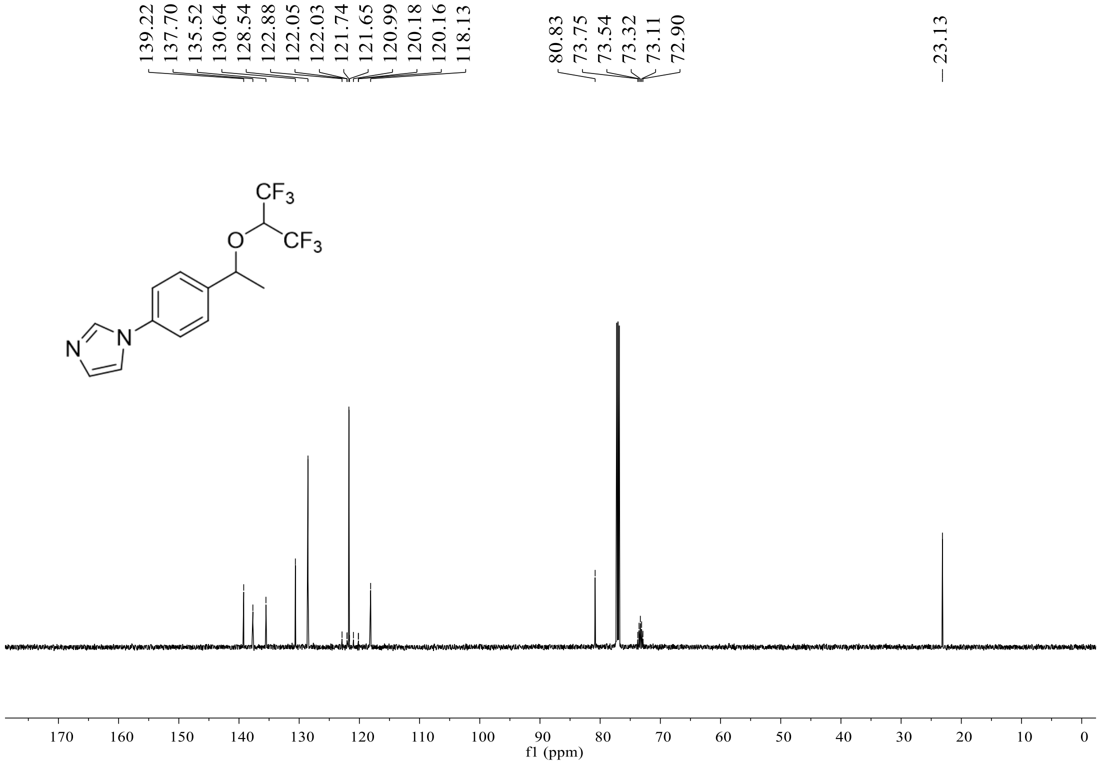

^{13}C NMR spectrum in $CDCl_3$.

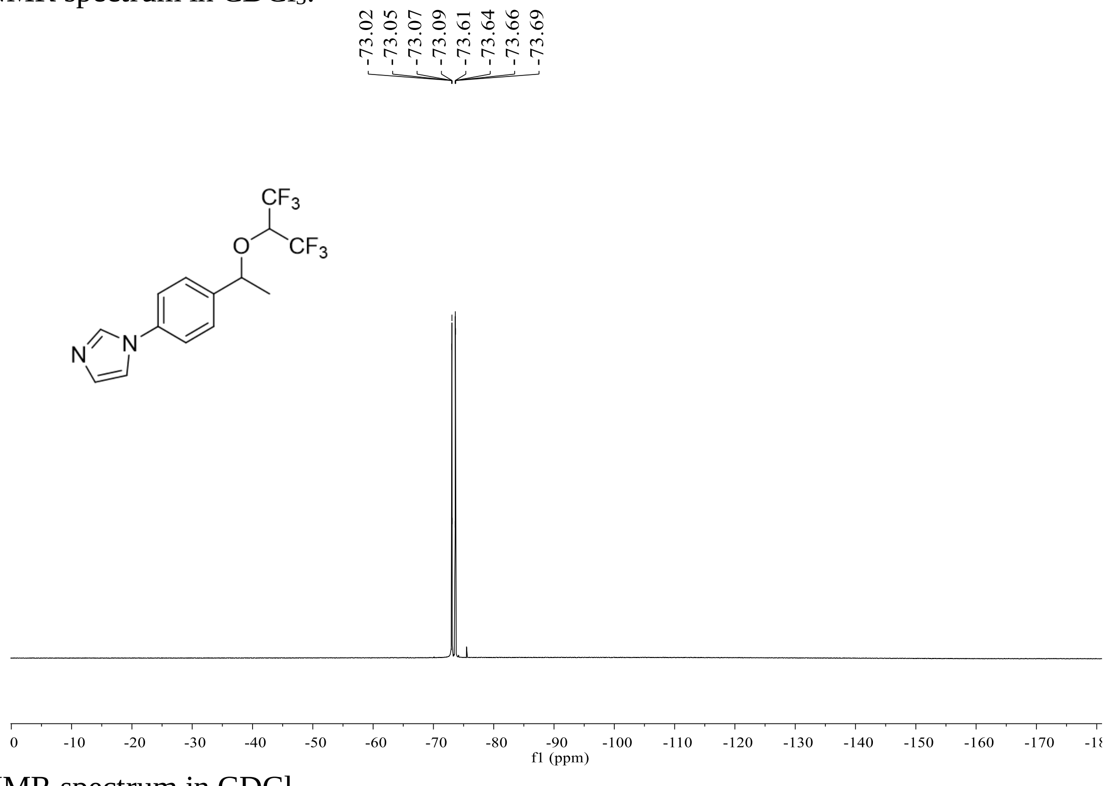

^{19}F NMR spectrum in $CDCl_3$.

81d

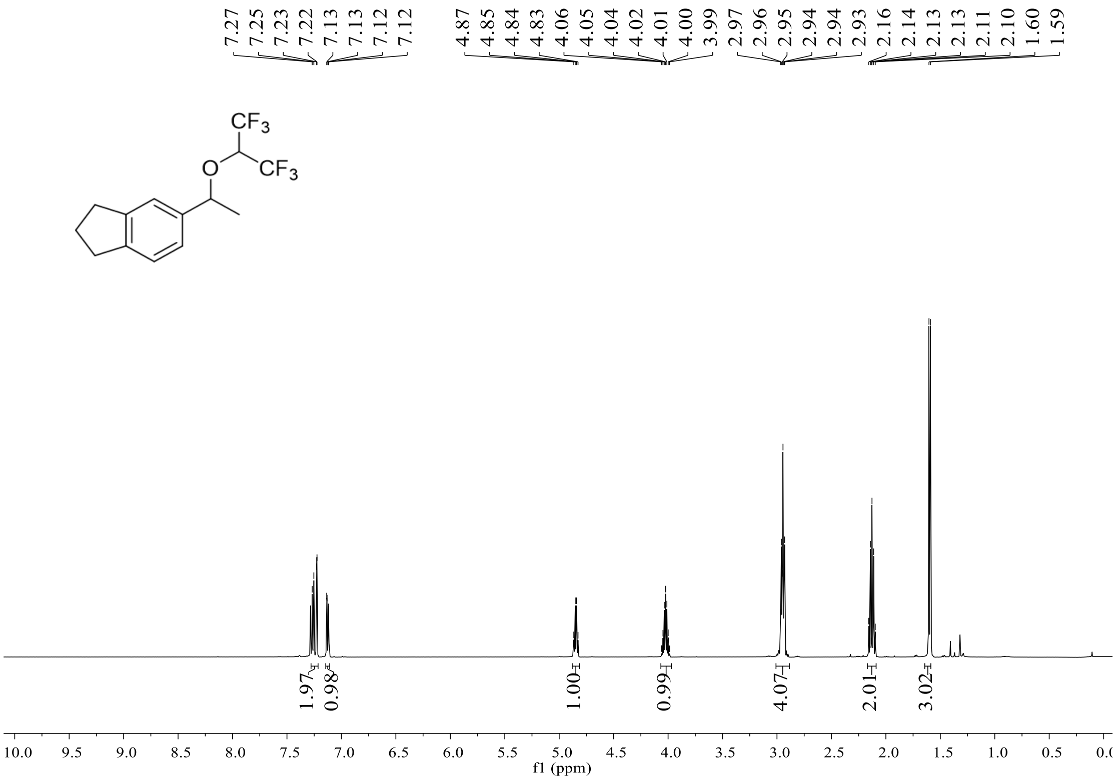

^{1}H NMR spectrum in $CDCl_3$.

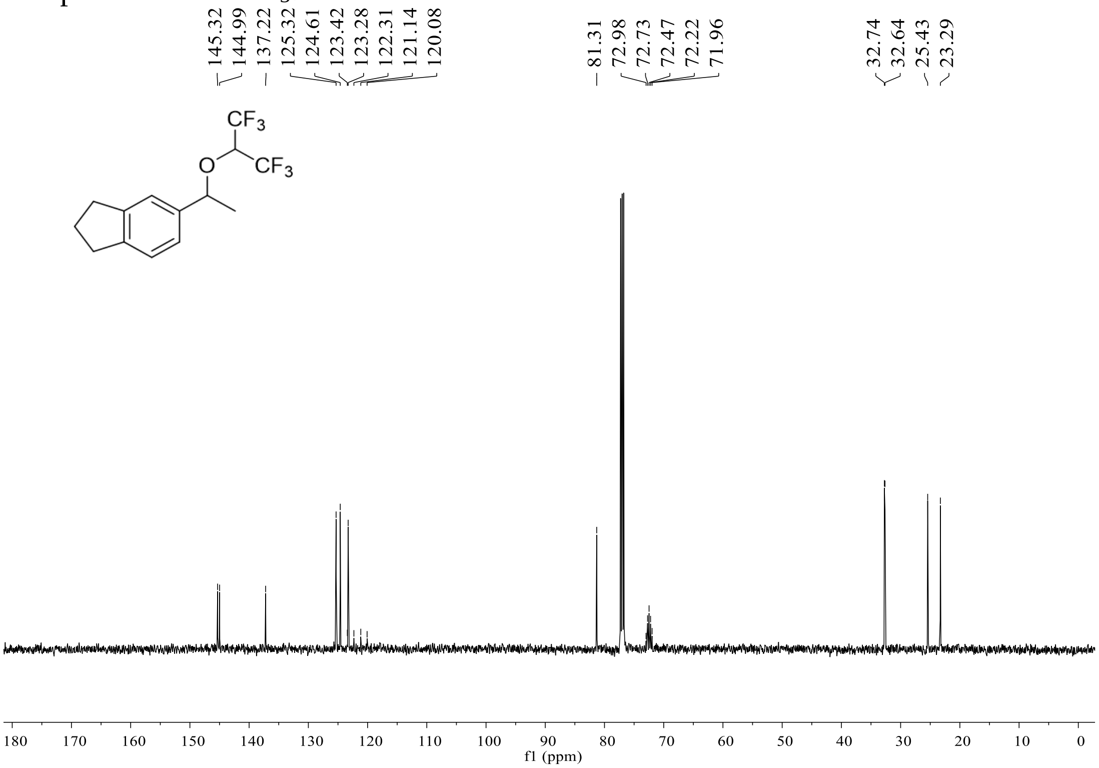

^{13}C NMR spectrum in $CDCl_3$.

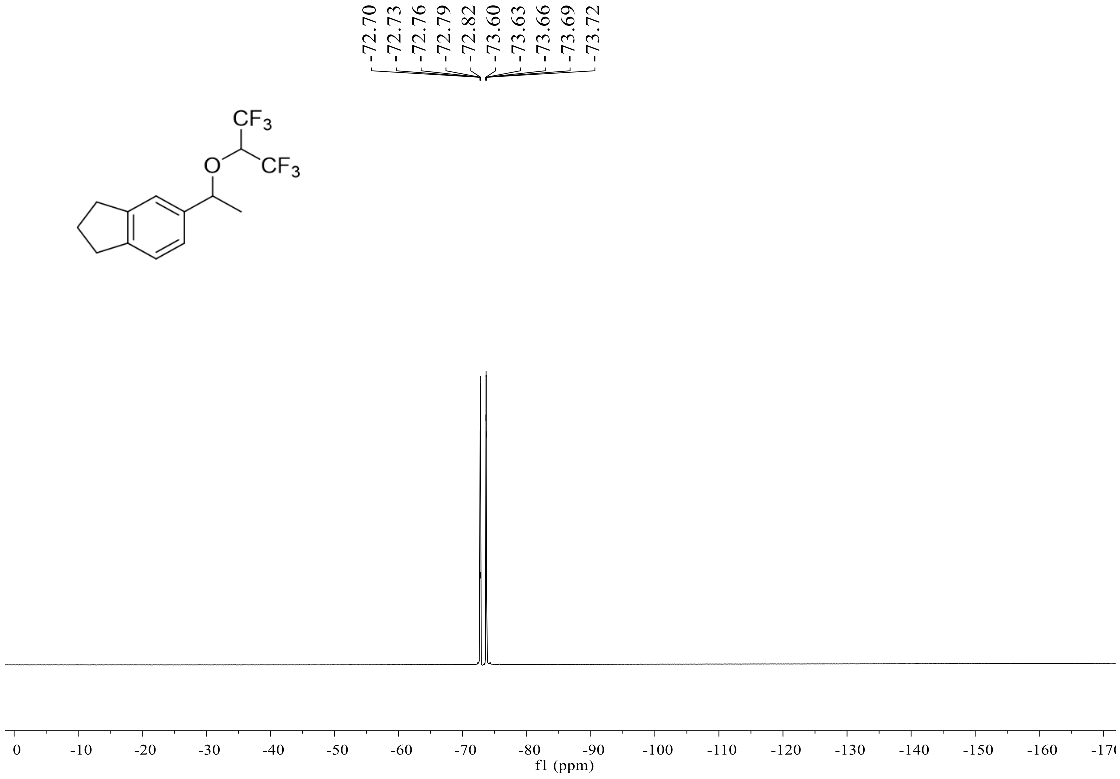

^{19}F NMR spectrum in $CDCl_3$.

82d

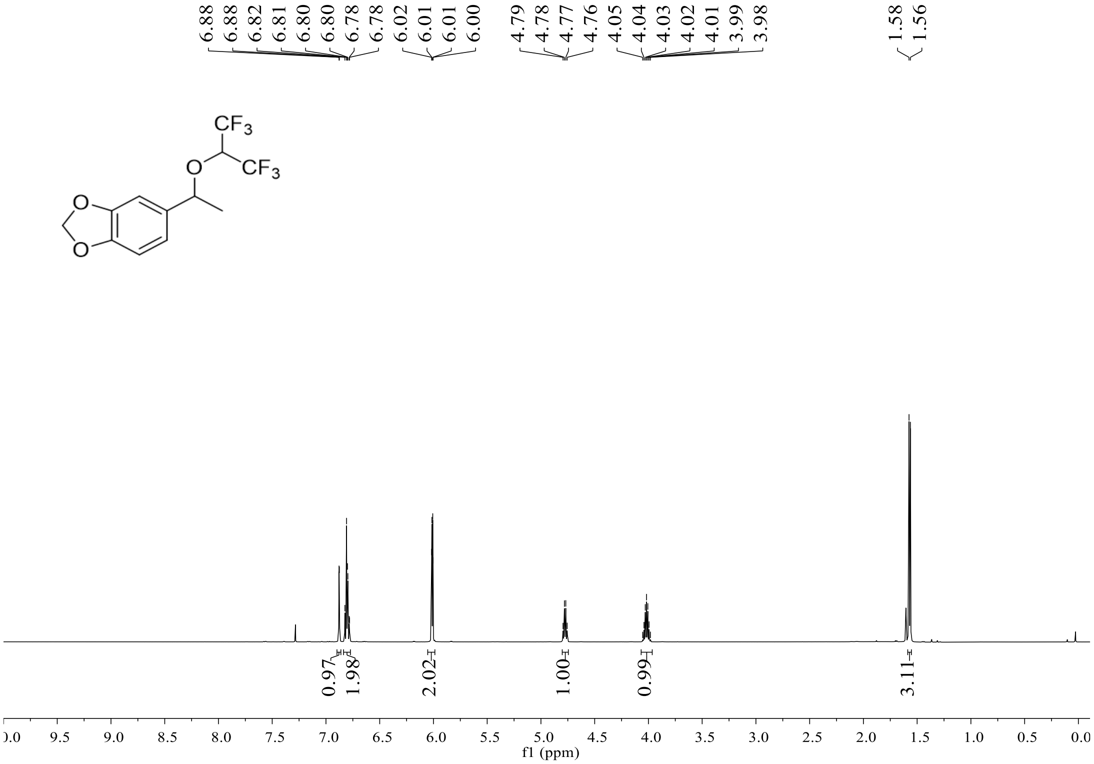

^{1}H NMR spectrum in $CDCl_3$.

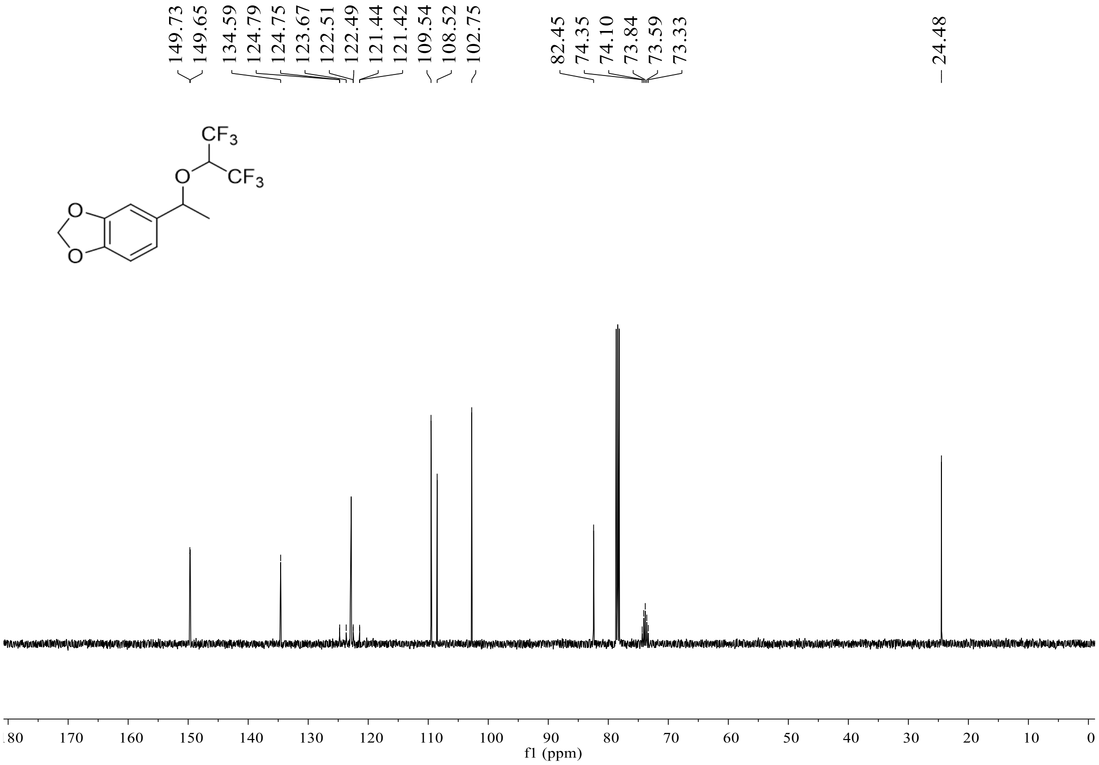

^{13}C NMR spectrum in $CDCl_3$.

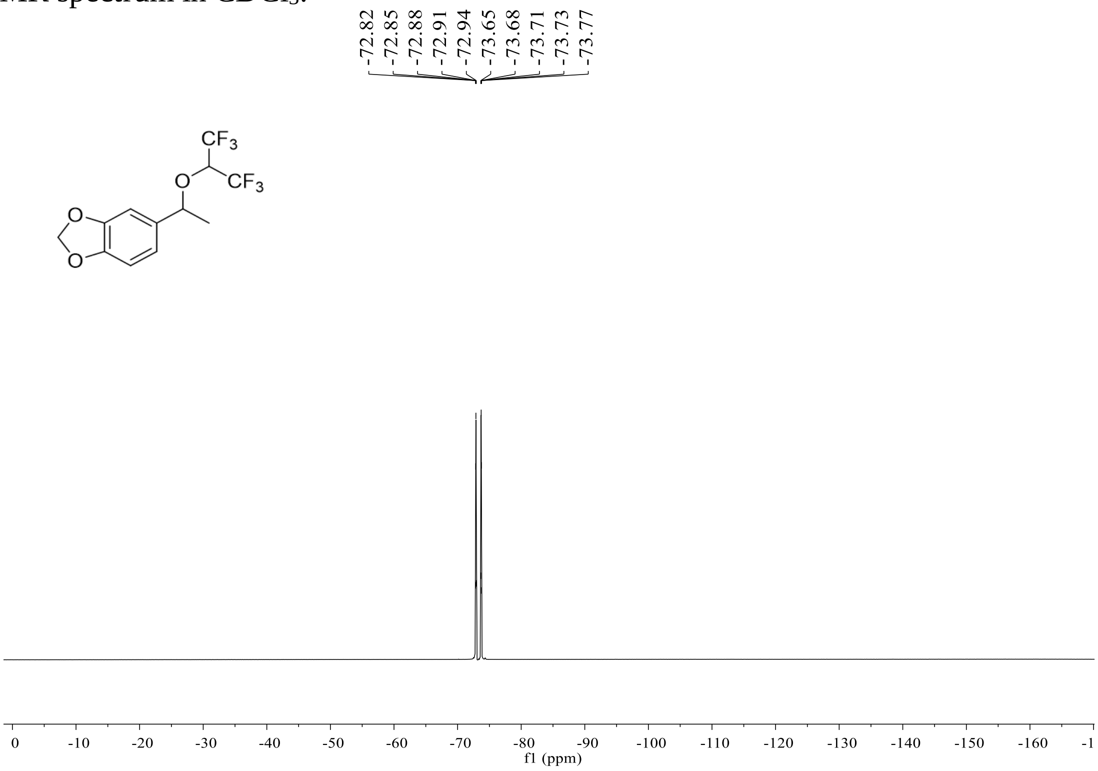

^{19}F NMR spectrum in $CDCl_3$.

83d

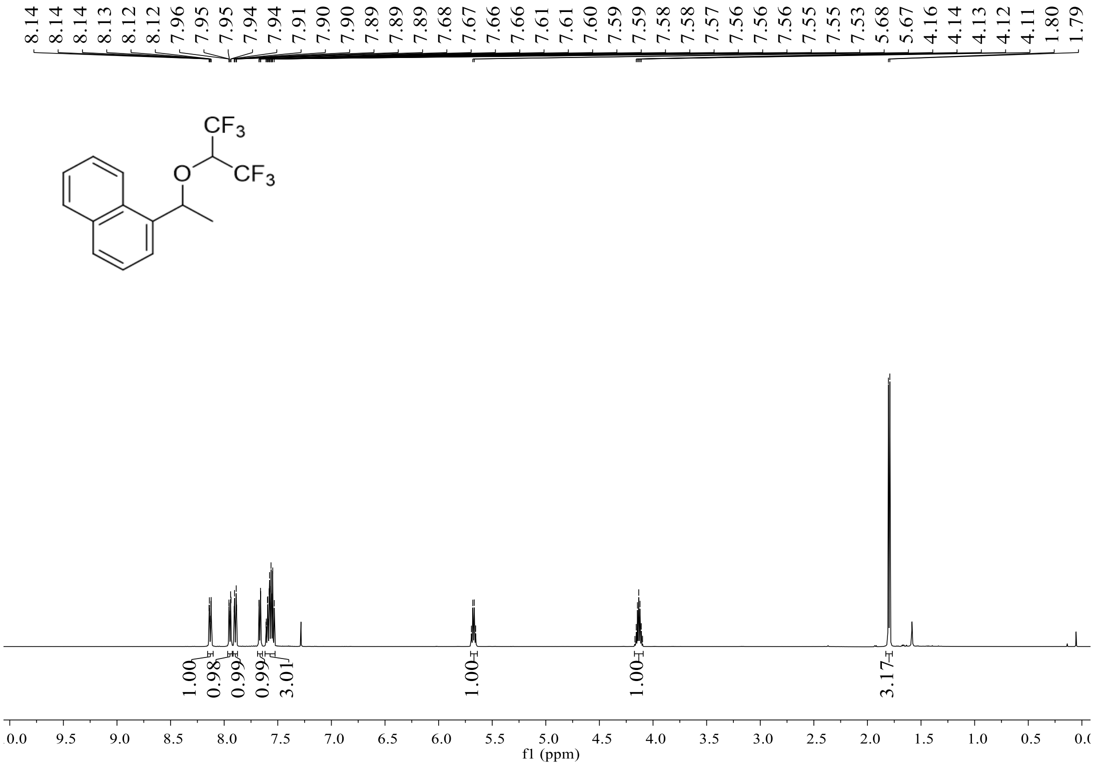

^{1}H NMR spectrum in CDCl$_3$.

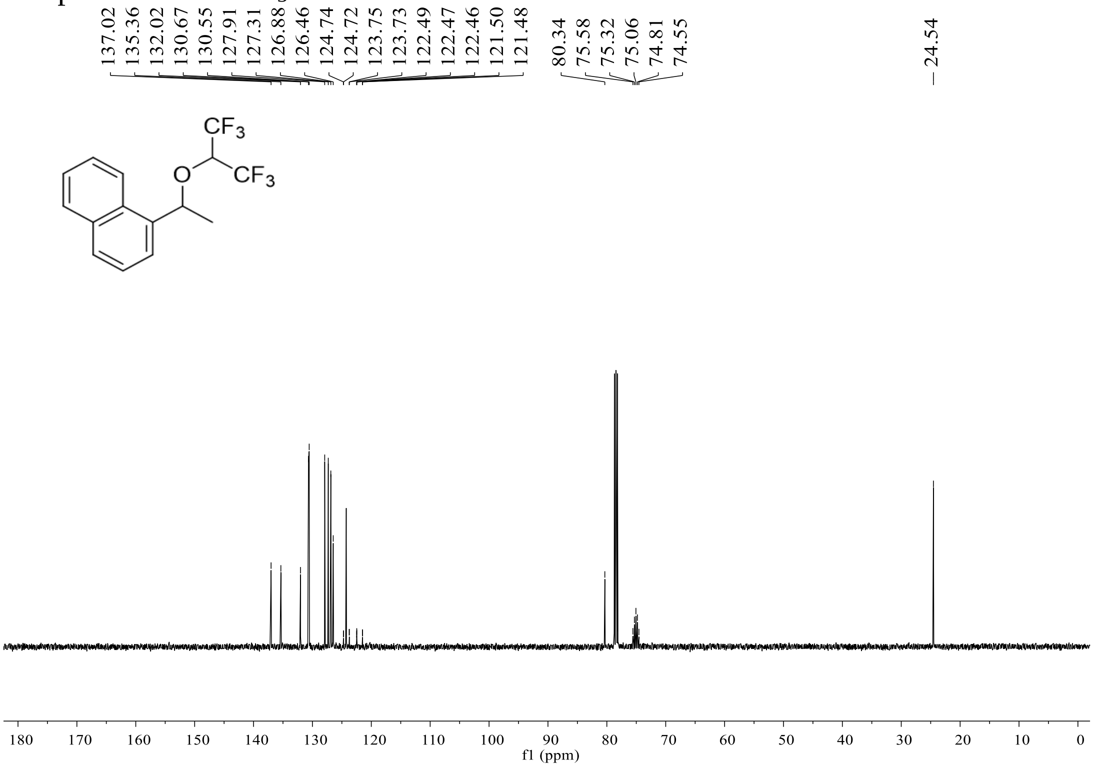

^{13}C NMR spectrum in CDCl$_3$.

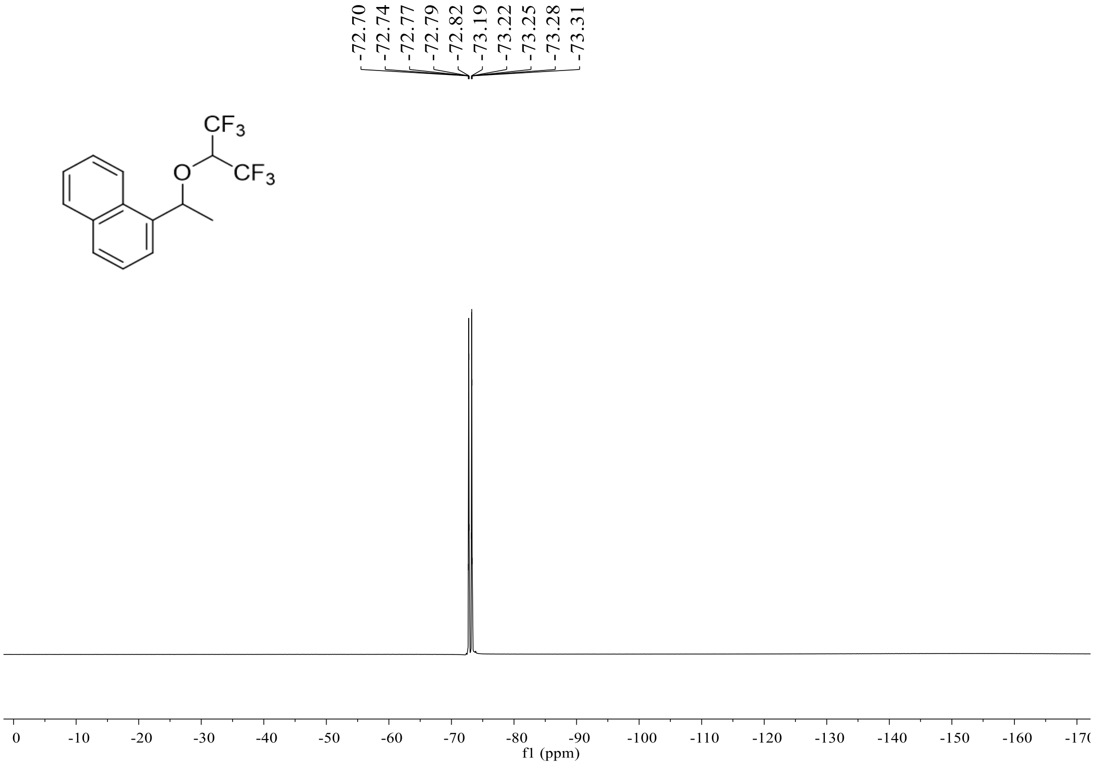

^{19}F NMR spectrum in $CDCl_3$.

84d

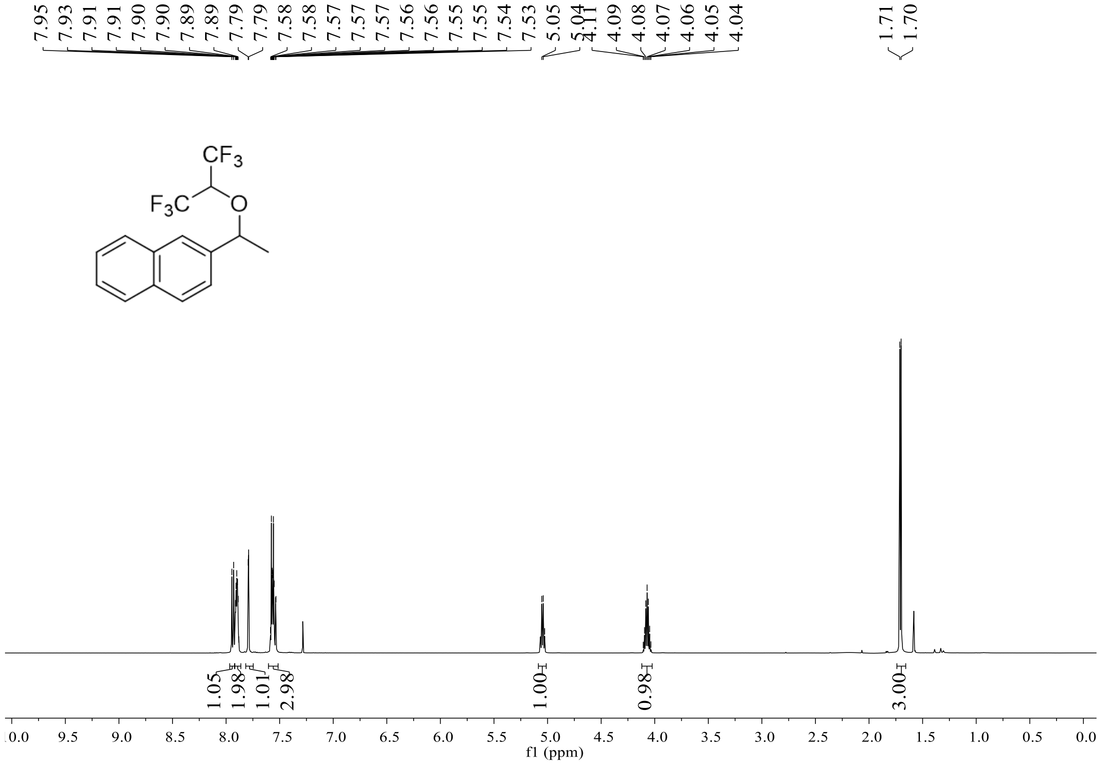

^{1}H NMR spectrum in $CDCl_3$.

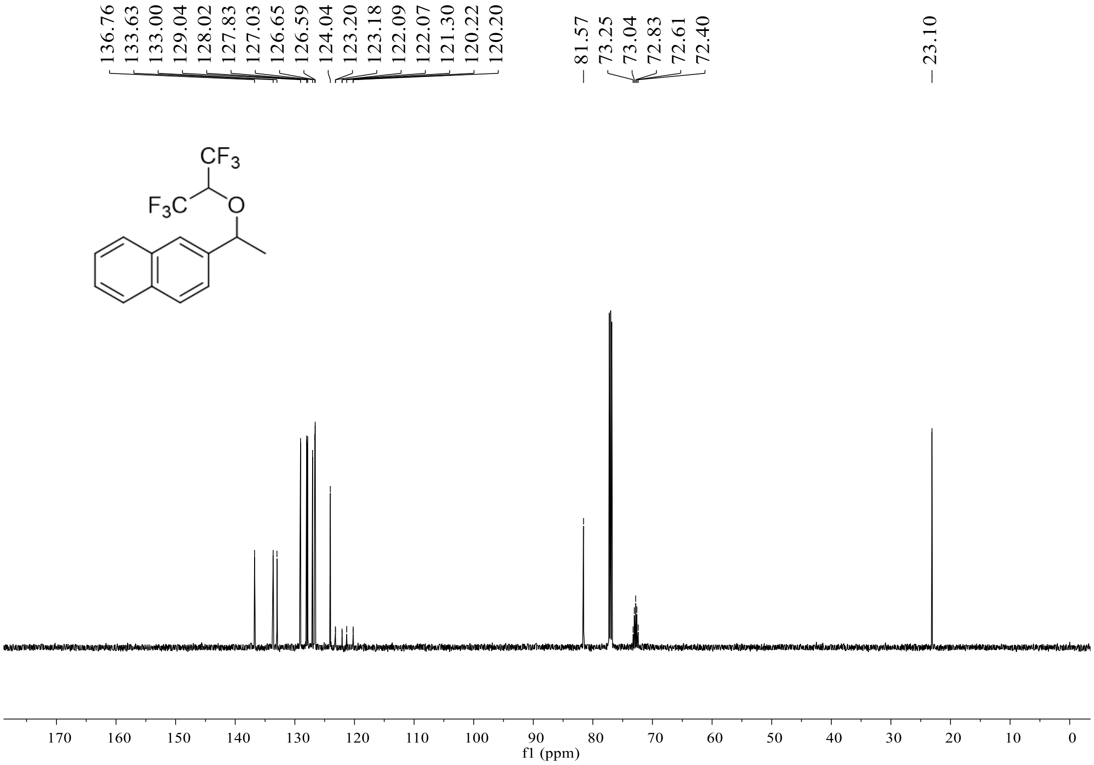

^{13}C NMR spectrum in $CDCl_3$.

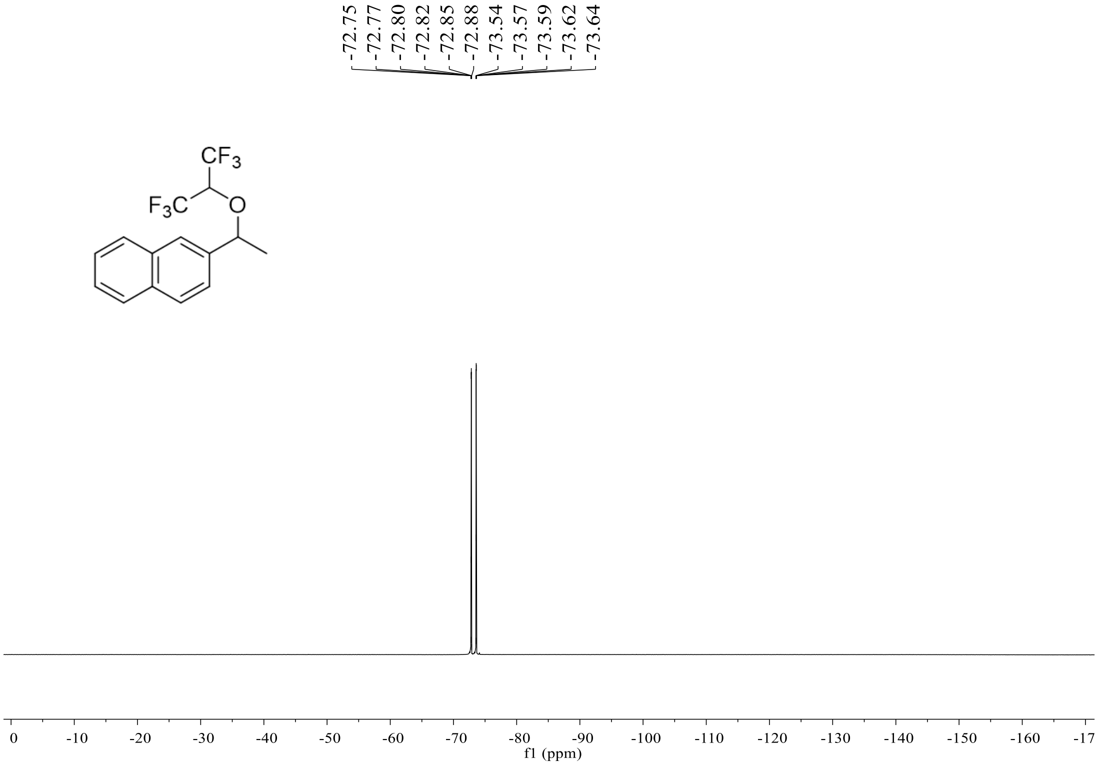

^{19}F NMR spectrum in $CDCl_3$.

85d

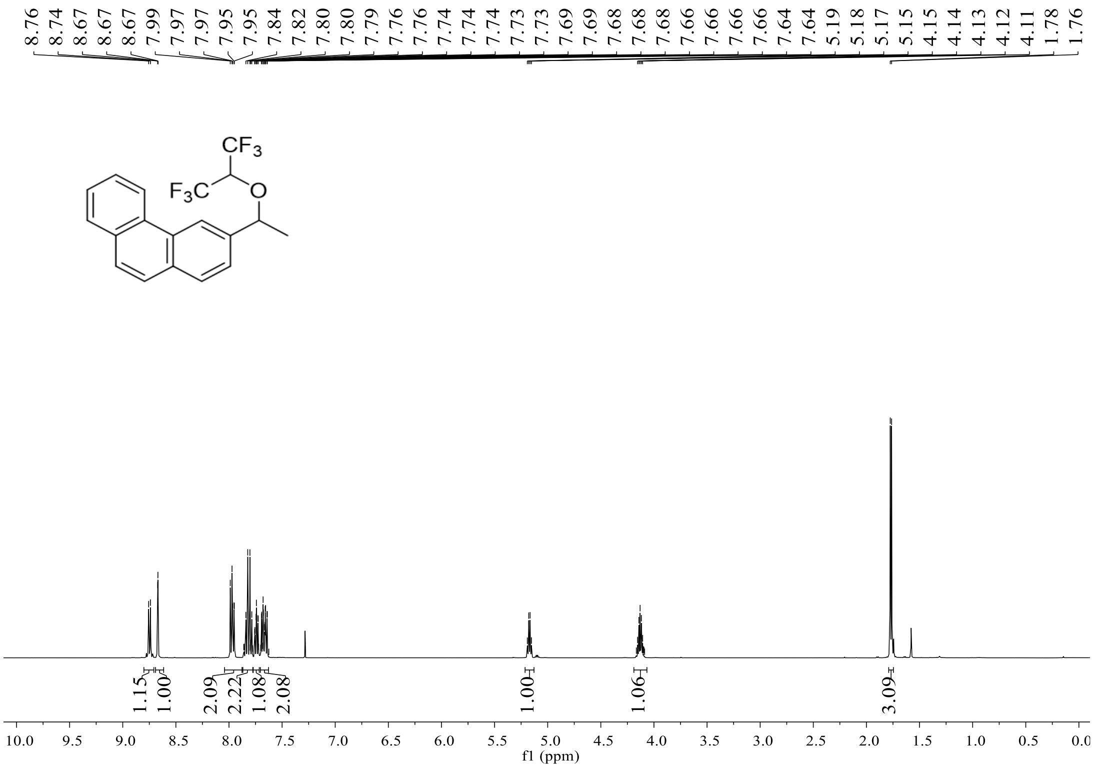

^{1}H NMR spectrum in $CDCl_3$.

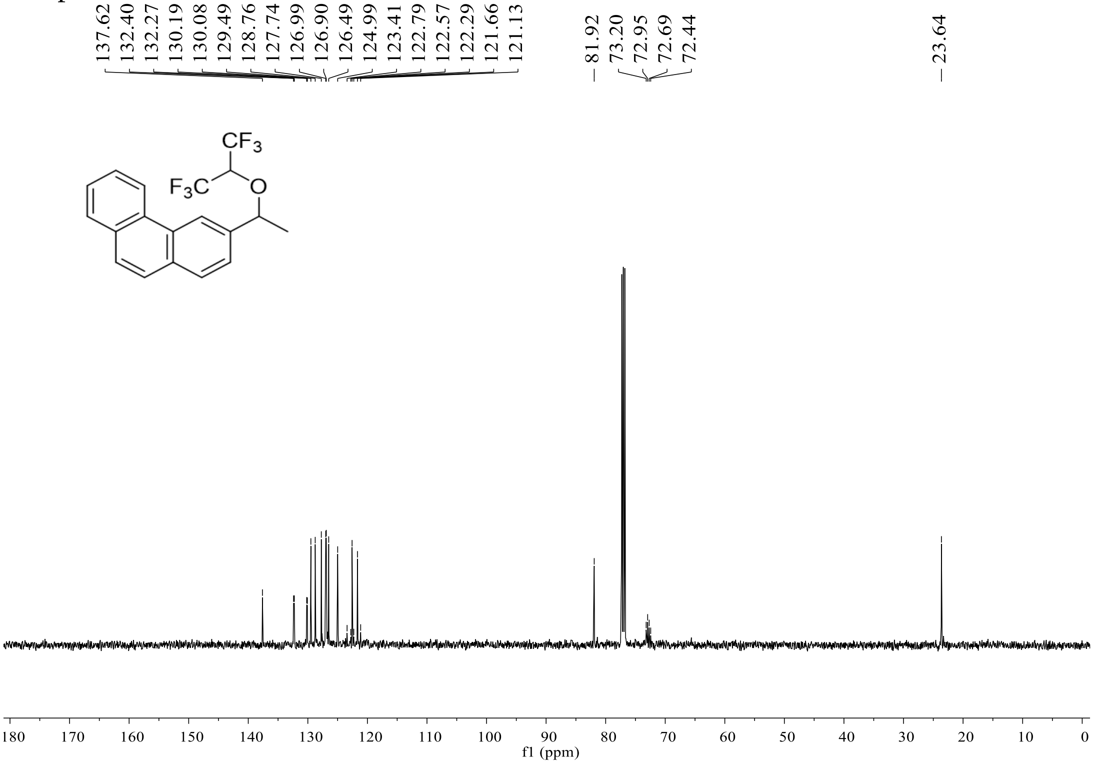

^{13}C NMR spectrum in $CDCl_3$.

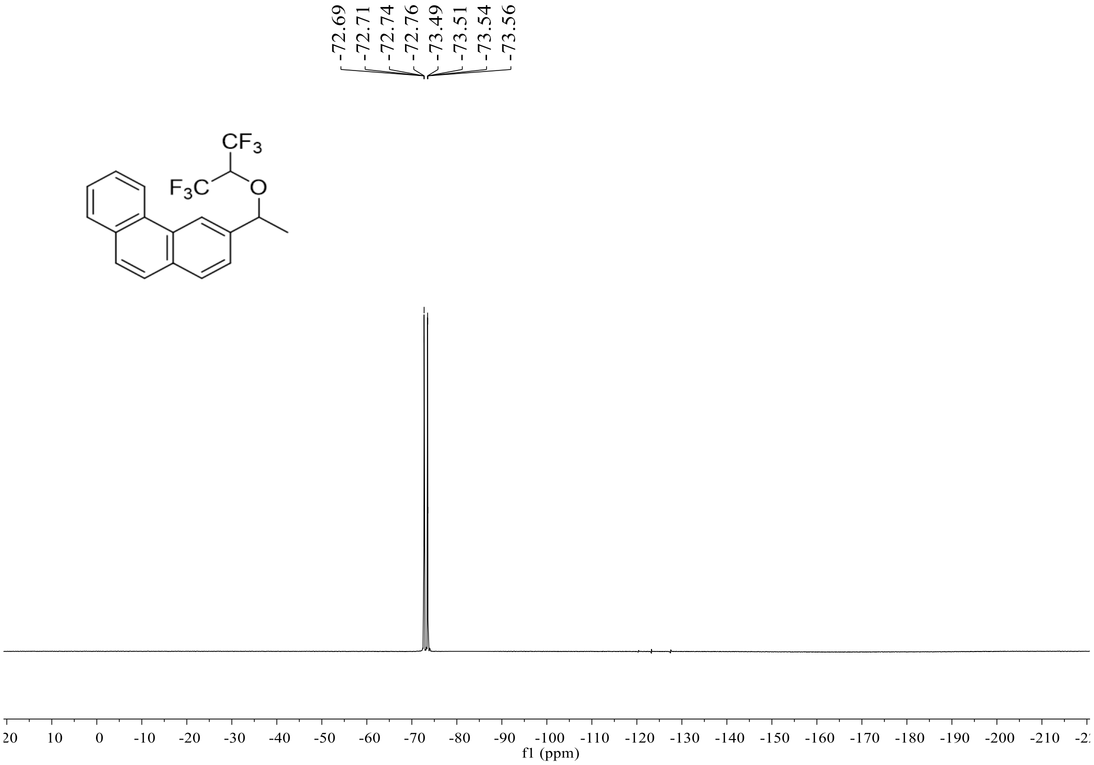

^{19}F NMR spectrum in $CDCl_3$.

86d

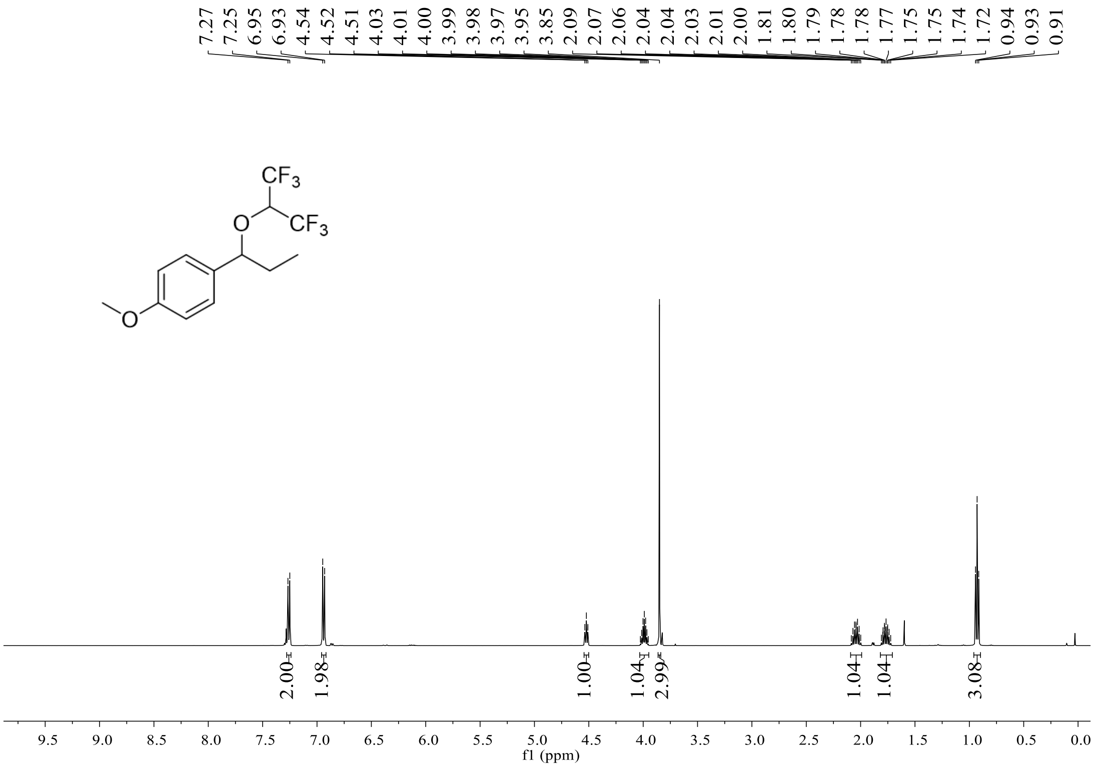

^{1}H NMR spectrum in $CDCl_3$.

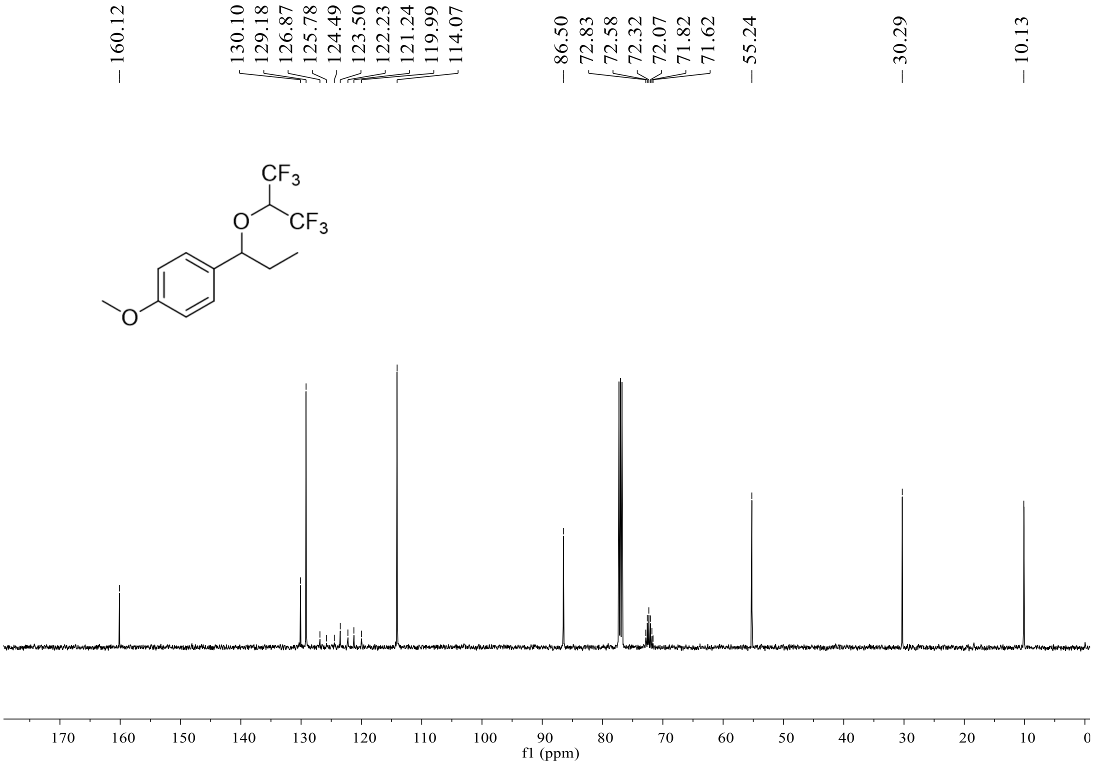

^{13}C NMR spectrum in $CDCl_3$.

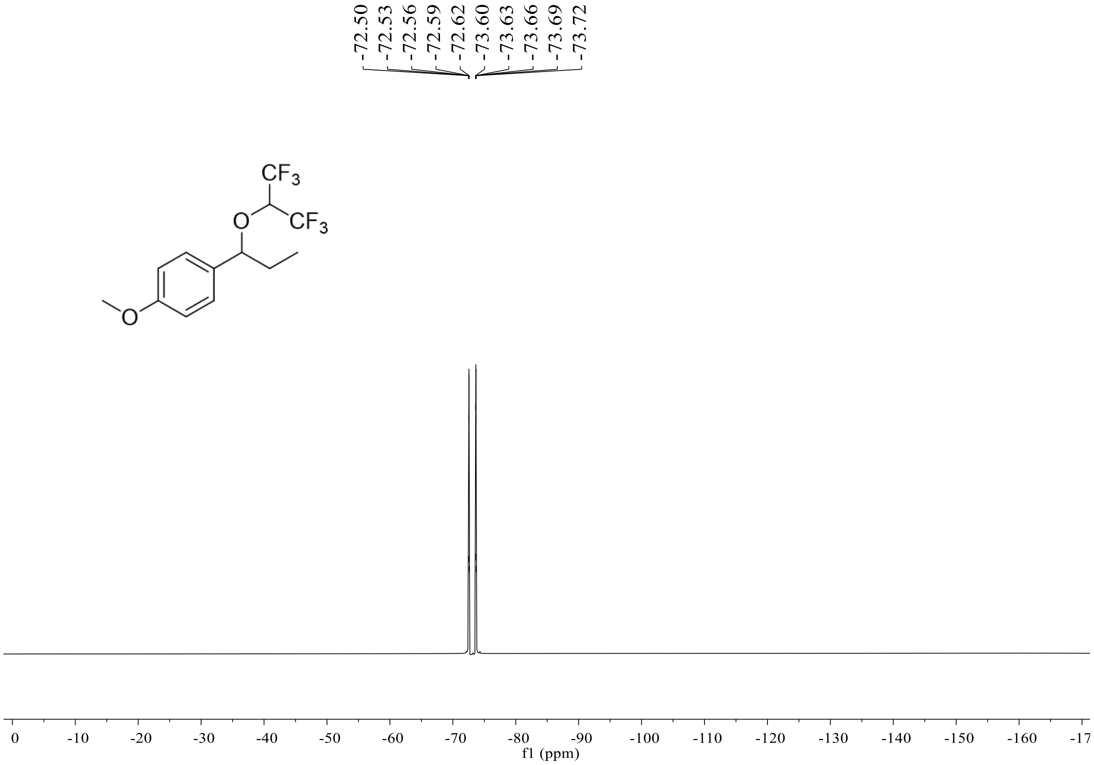

^{19}F NMR spectrum in $CDCl_3$.

87d

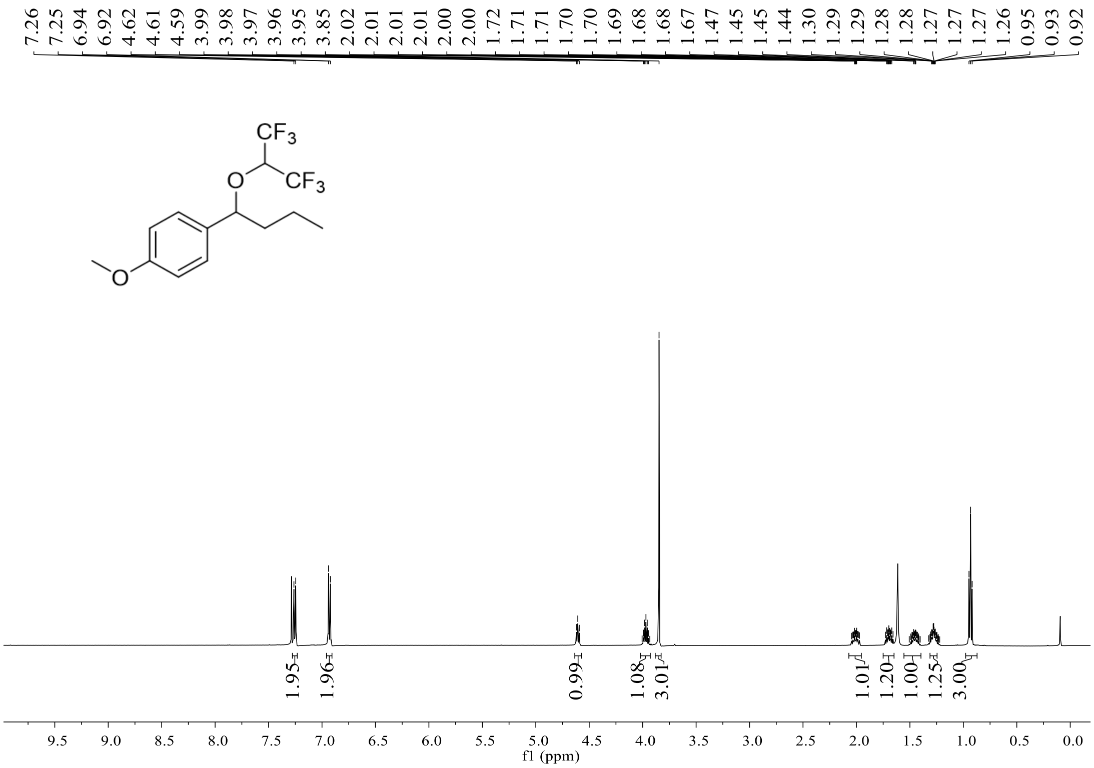

^{1}H NMR spectrum in $CDCl_3$.

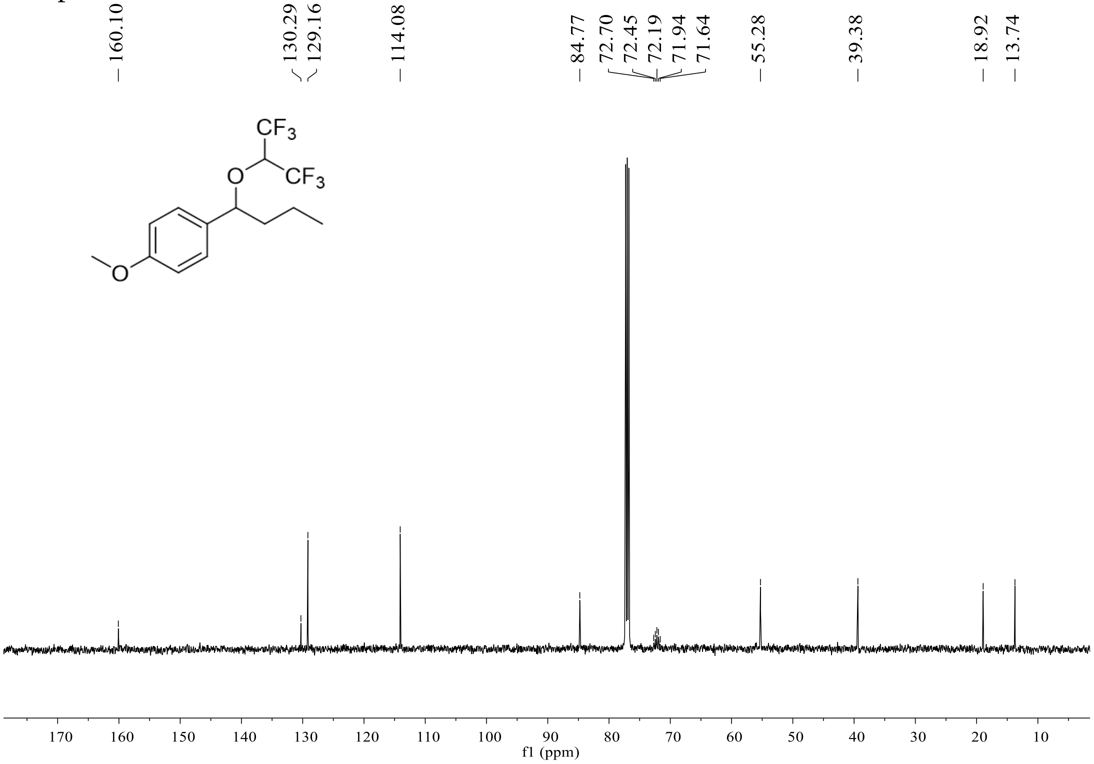

^{13}C NMR spectrum in $CDCl_3$.

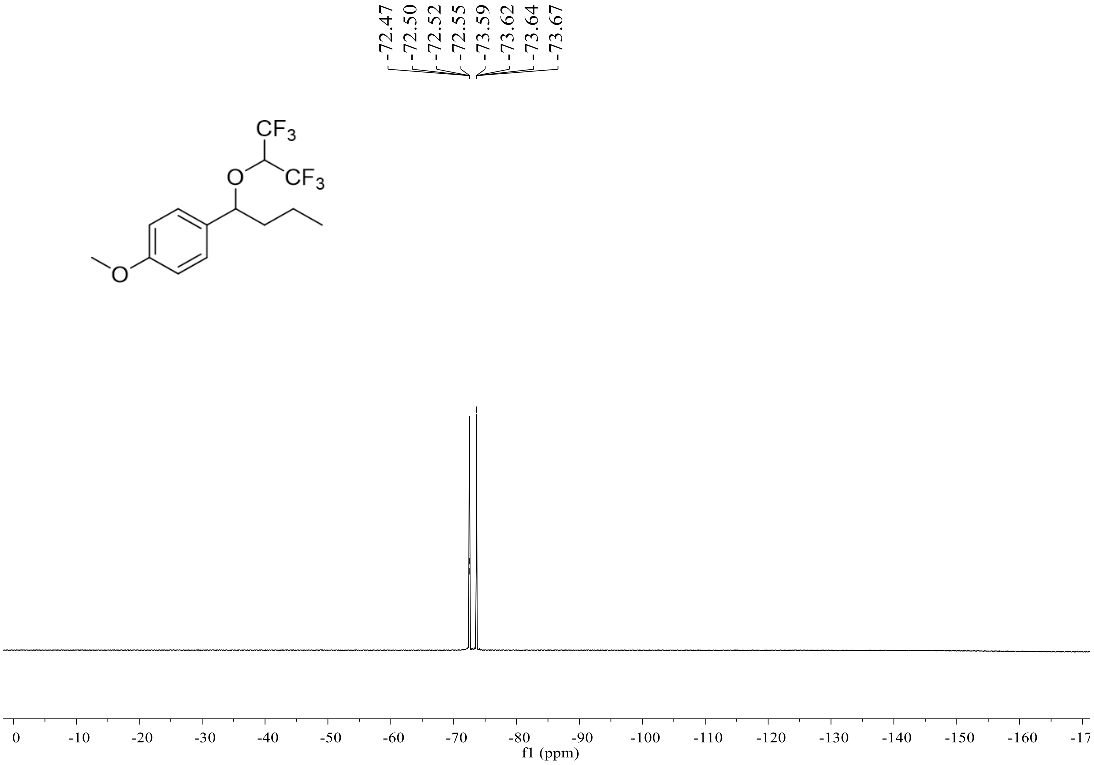

^{19}F NMR spectrum in $CDCl_3$.

88d

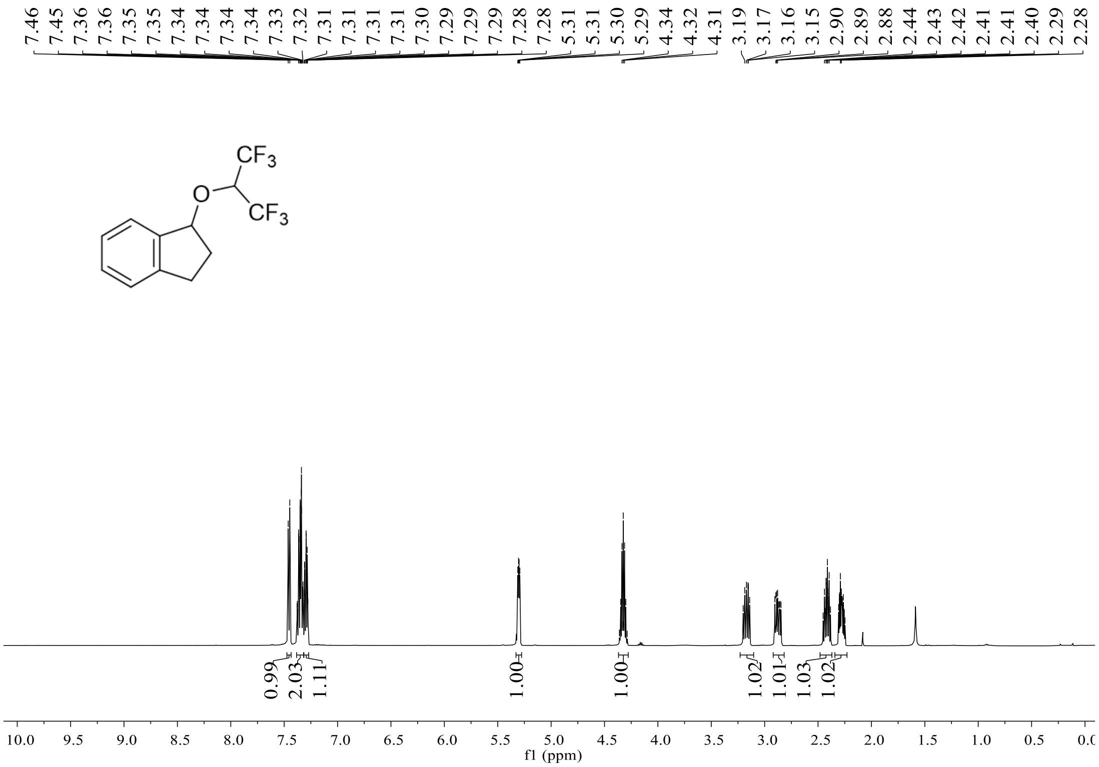

^{1}H NMR spectrum in $CDCl_3$.

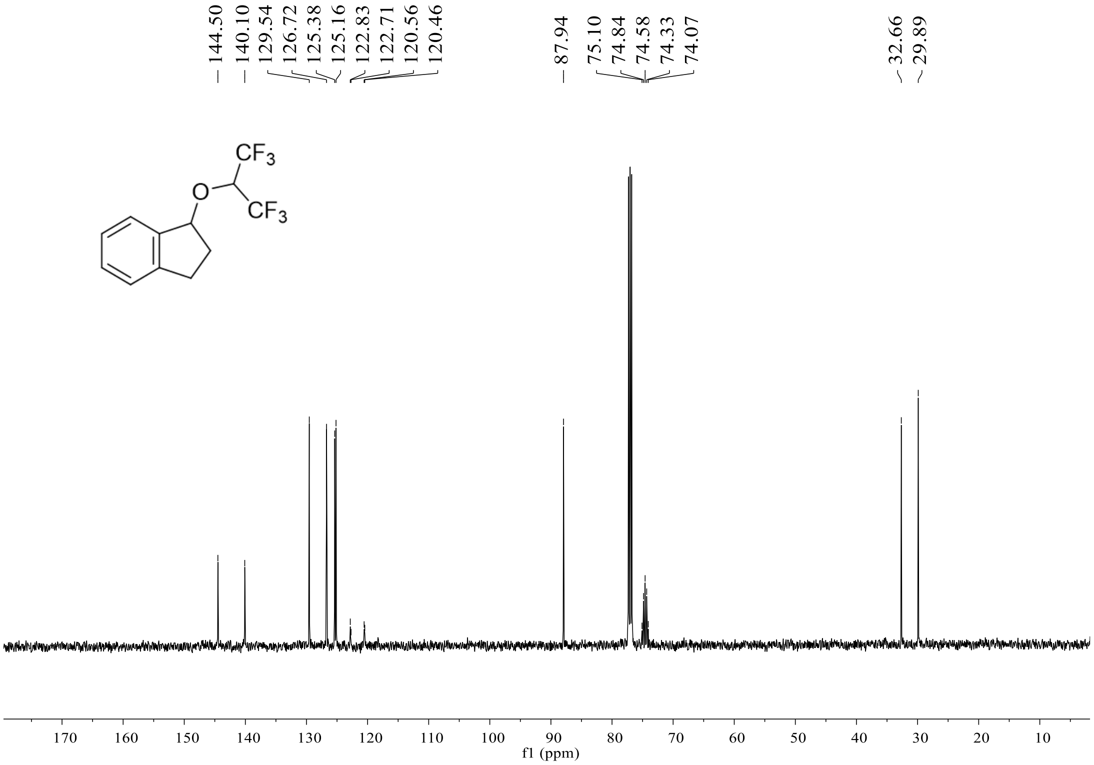

^{13}C NMR spectrum in $CDCl_3$.

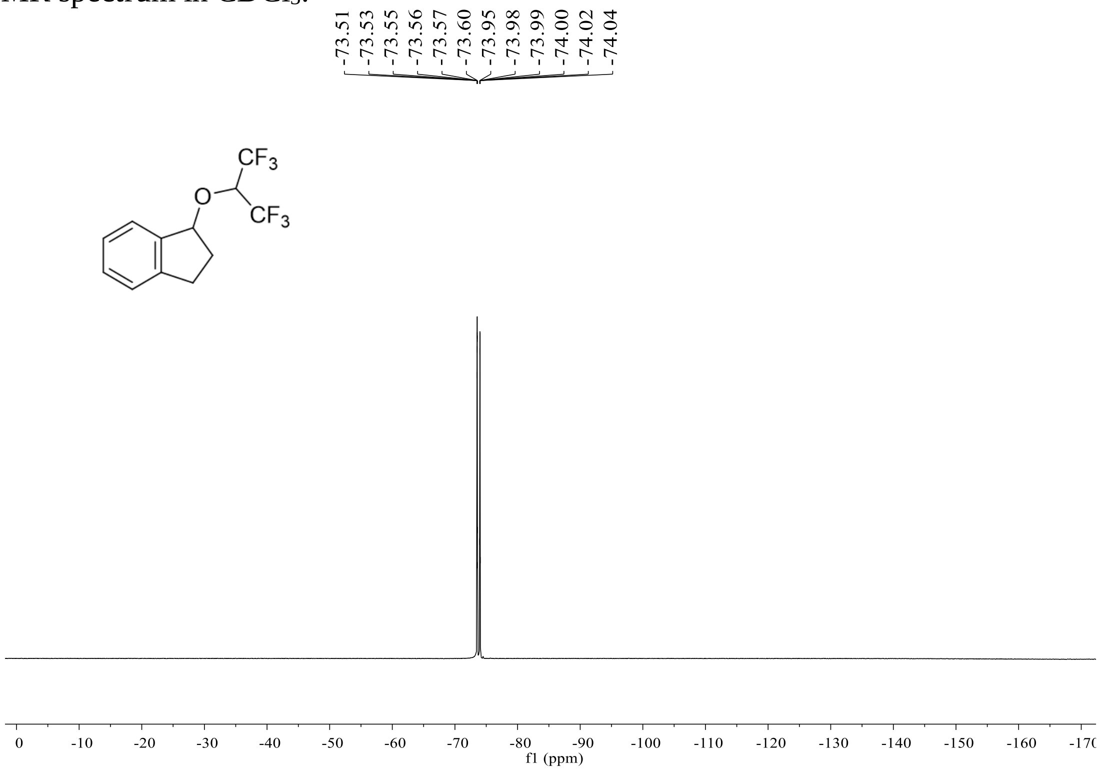

^{19}F NMR spectrum in $CDCl_3$.

89d

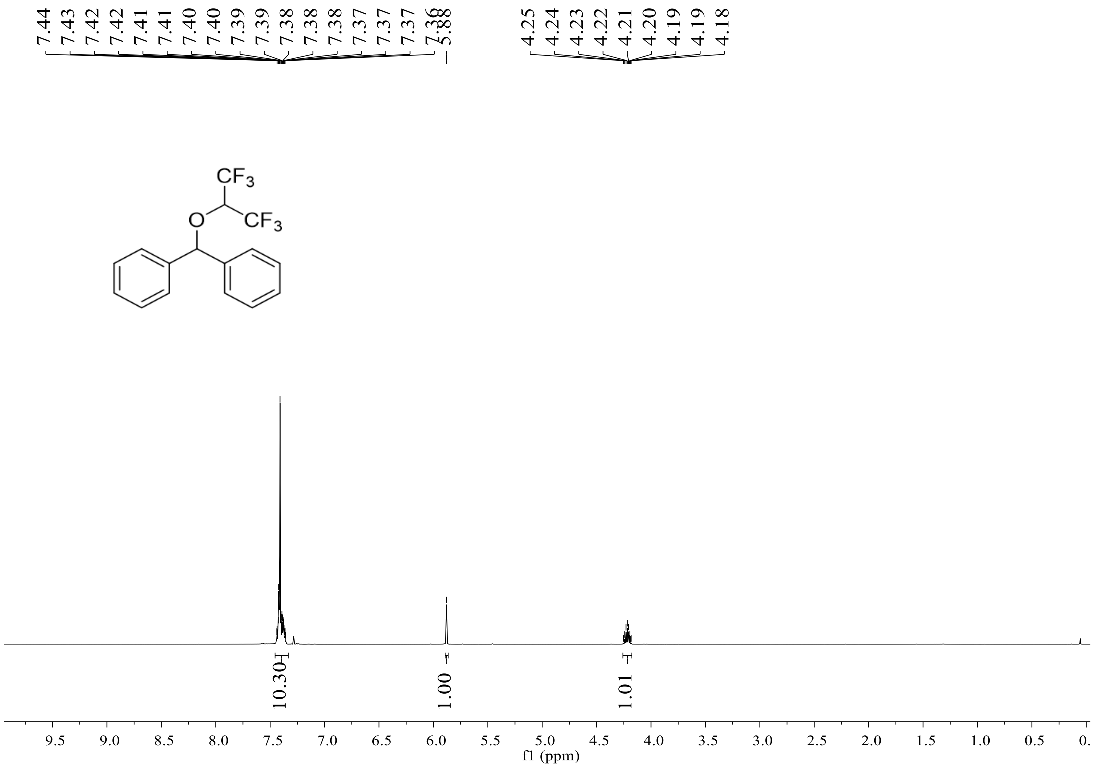

^{1}H NMR spectrum in $CDCl_3$.

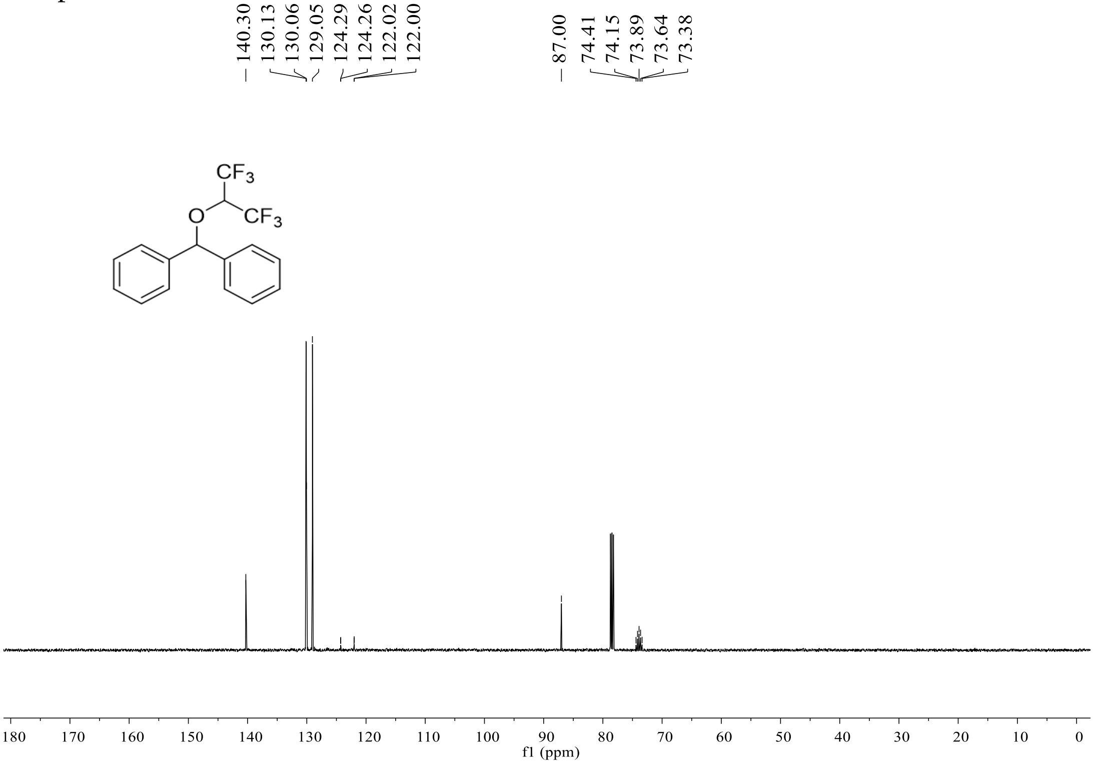

^{13}C NMR spectrum in $CDCl_3$.

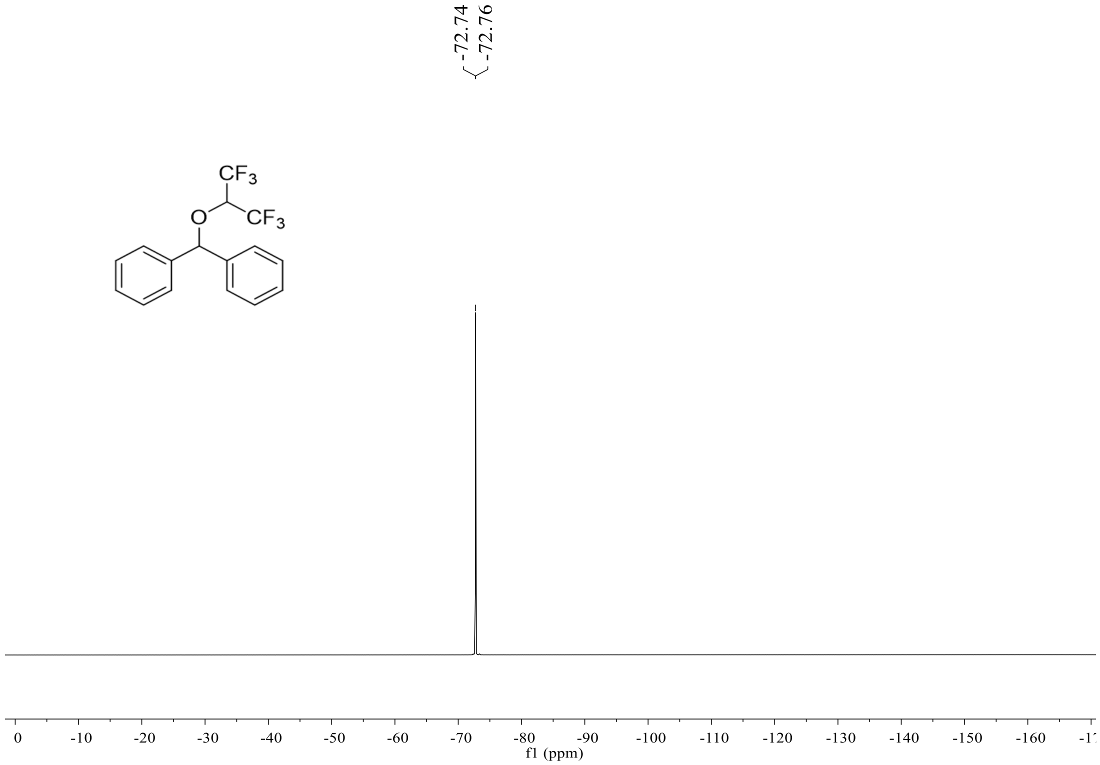

^{19}F NMR spectrum in $CDCl_3$.

90d

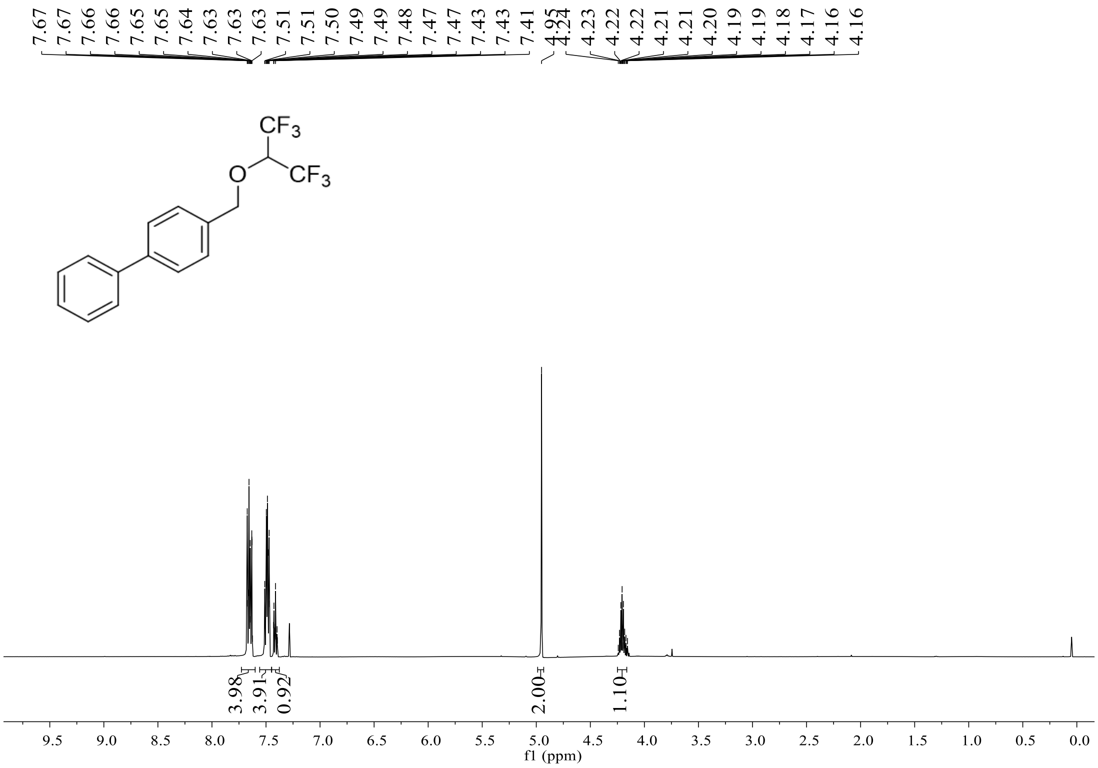

^{1}H NMR spectrum in $CDCl_3$.

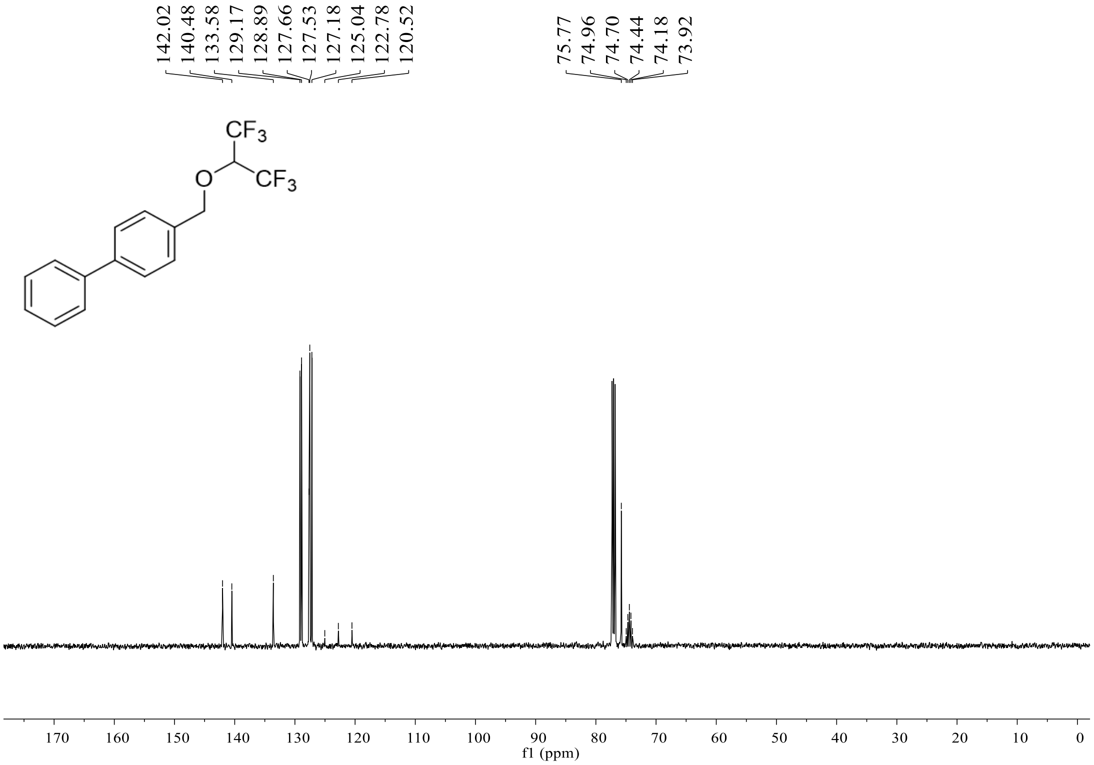

^{13}C NMR spectrum in $CDCl_3$.

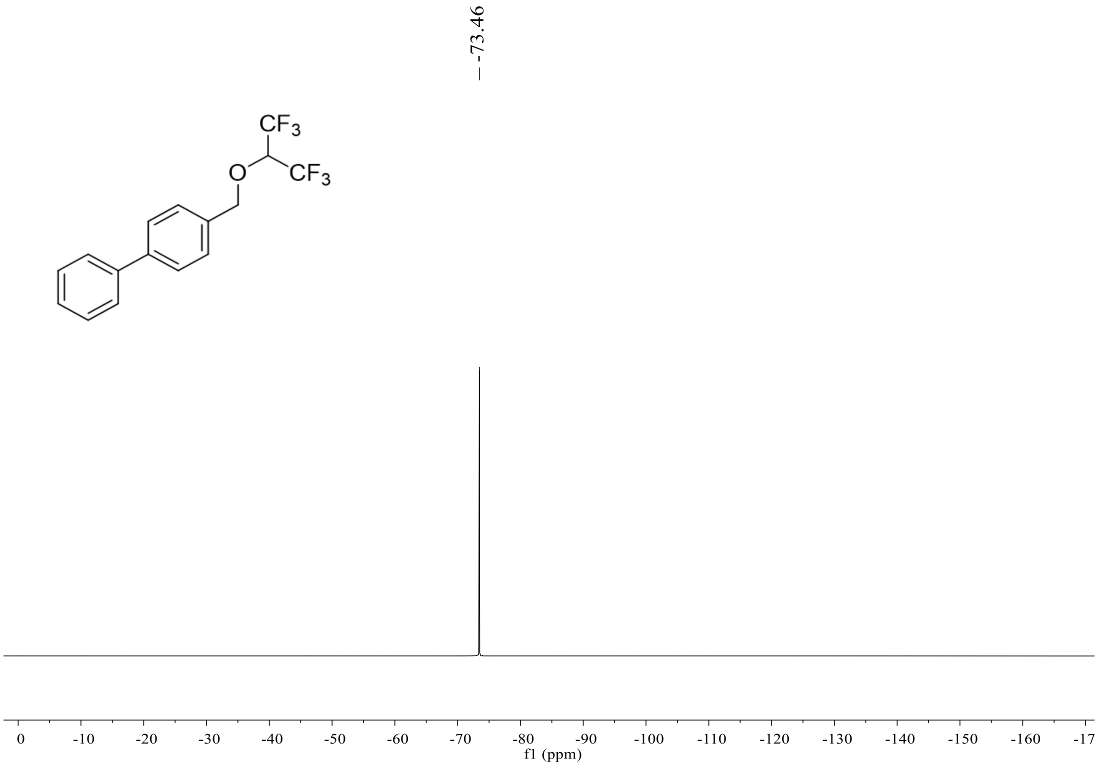

^{19}F NMR spectrum in $CDCl_3$.

91d

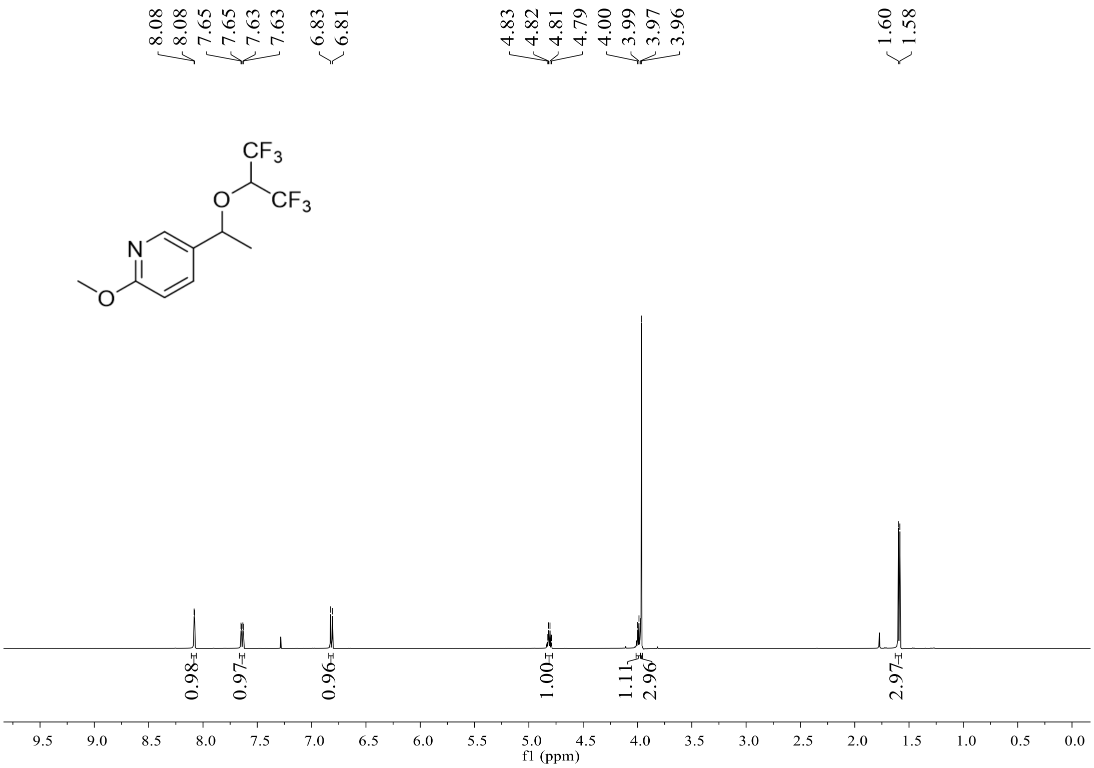

^{1}H NMR spectrum in $CDCl_3$.

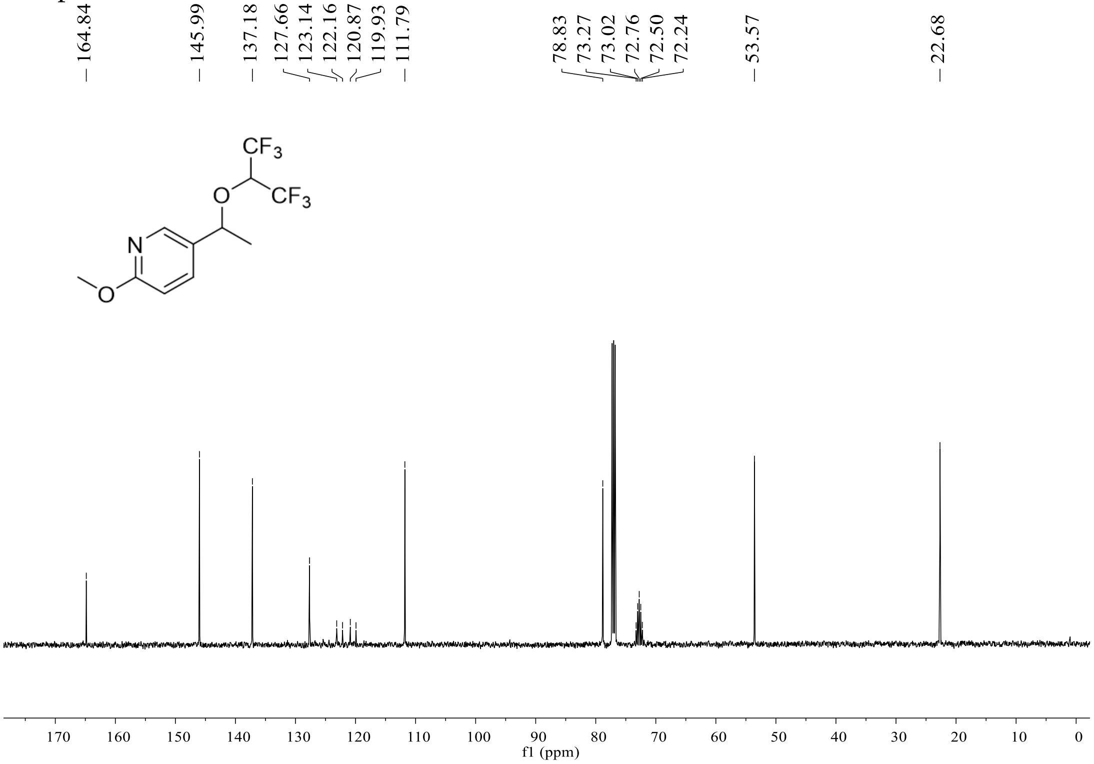

^{13}C NMR spectrum in $CDCl_3$.

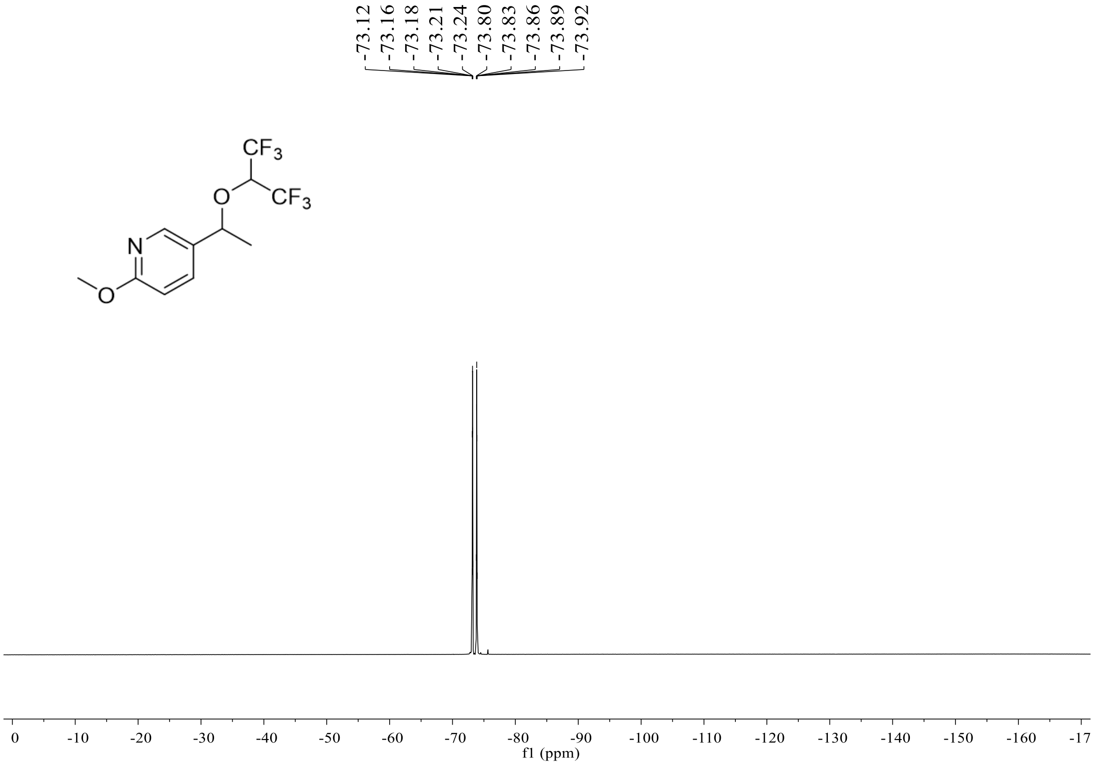

^{19}F NMR spectrum in $CDCl_3$.

92d

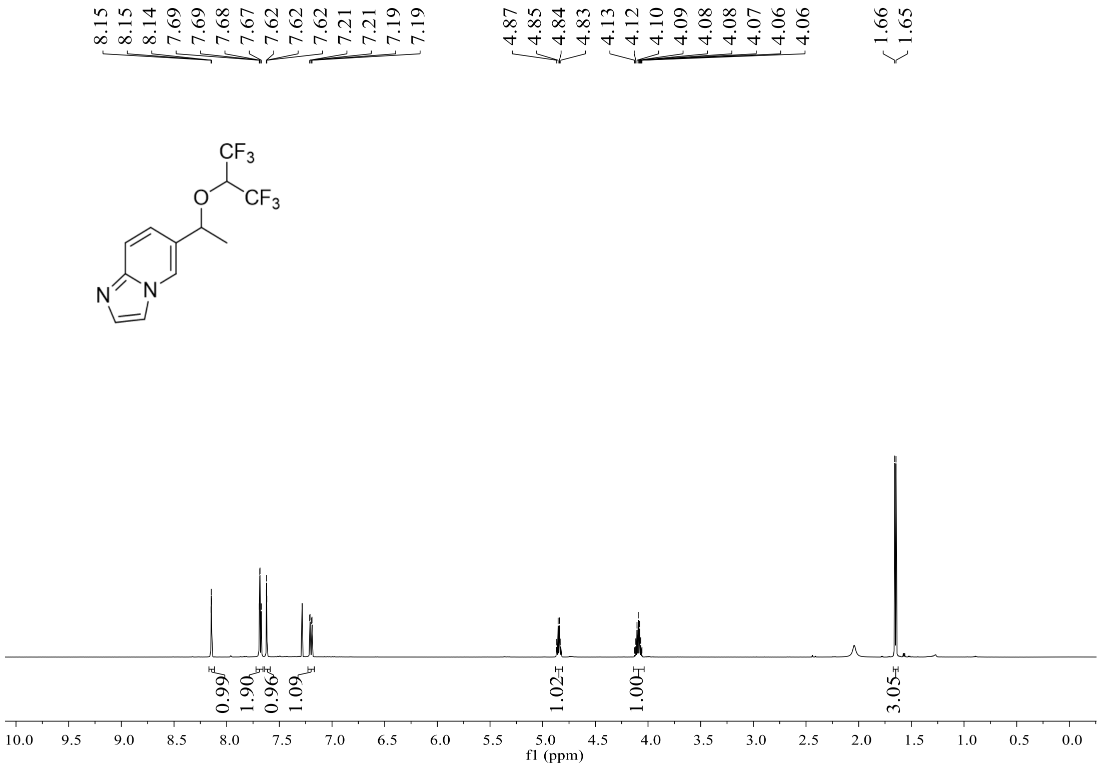

^{1}H NMR spectrum in $CDCl_3$.

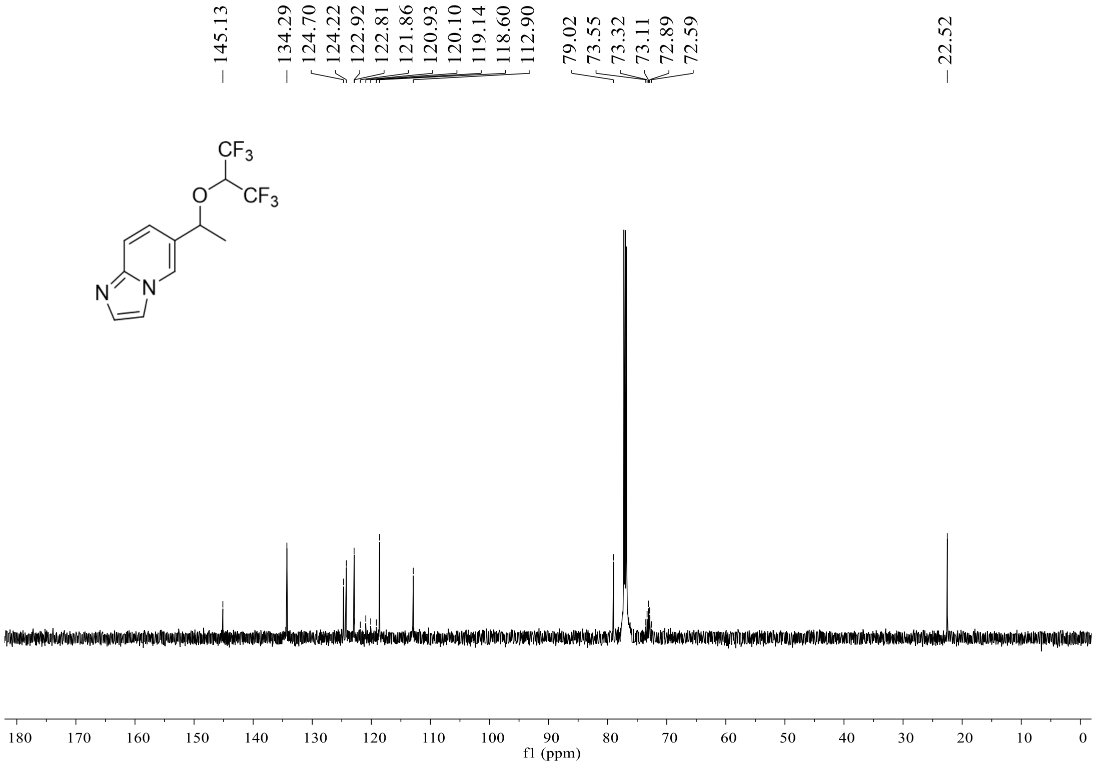

^{13}C NMR spectrum in $CDCl_3$.

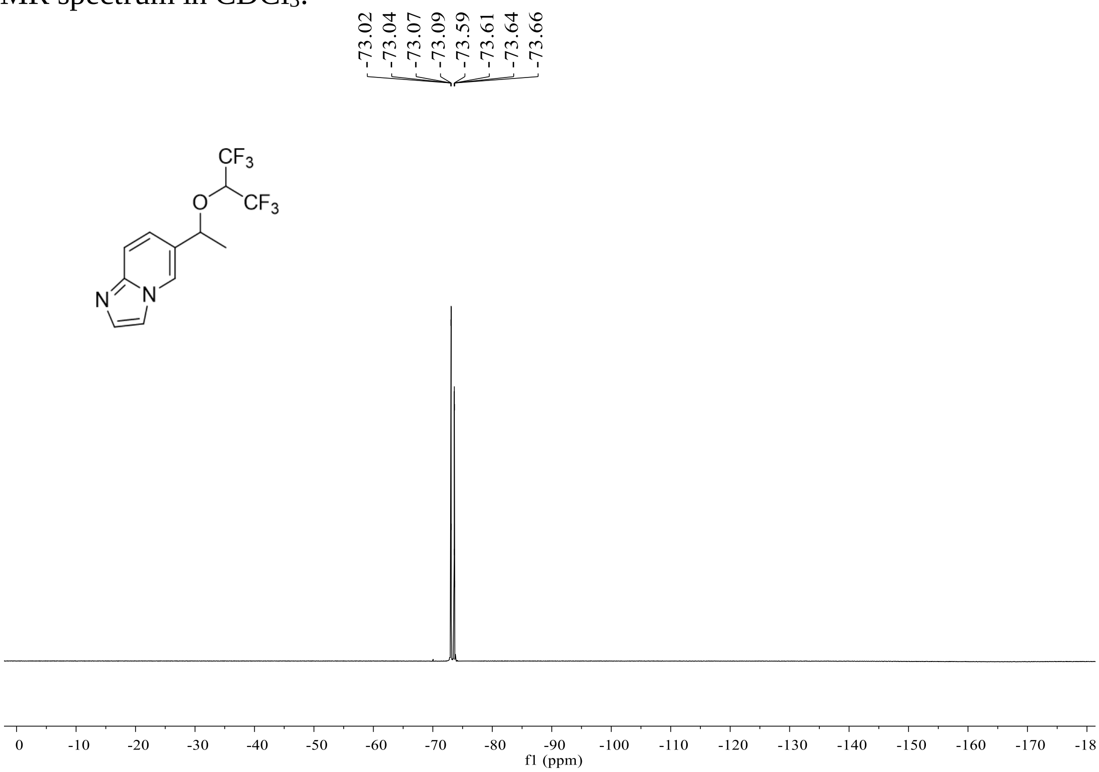

^{19}F NMR spectrum in $CDCl_3$.

93d

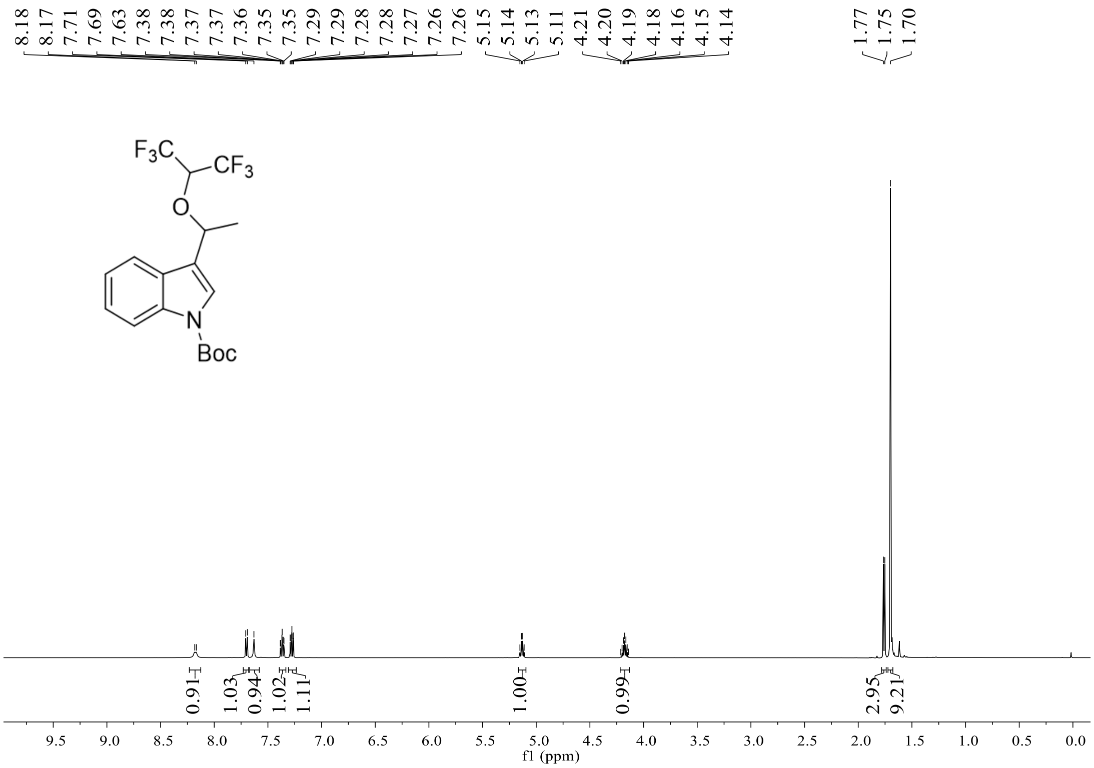

^{1}H NMR spectrum in $CDCl_3$.

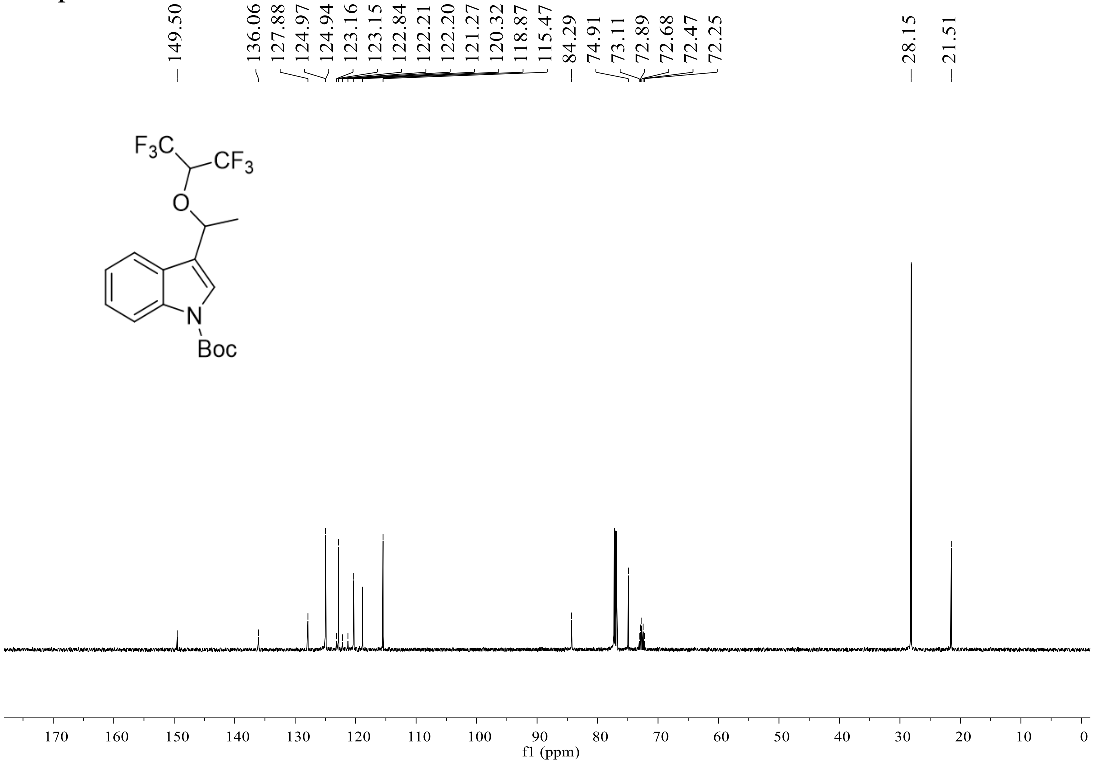

^{13}C NMR spectrum in $CDCl_3$.

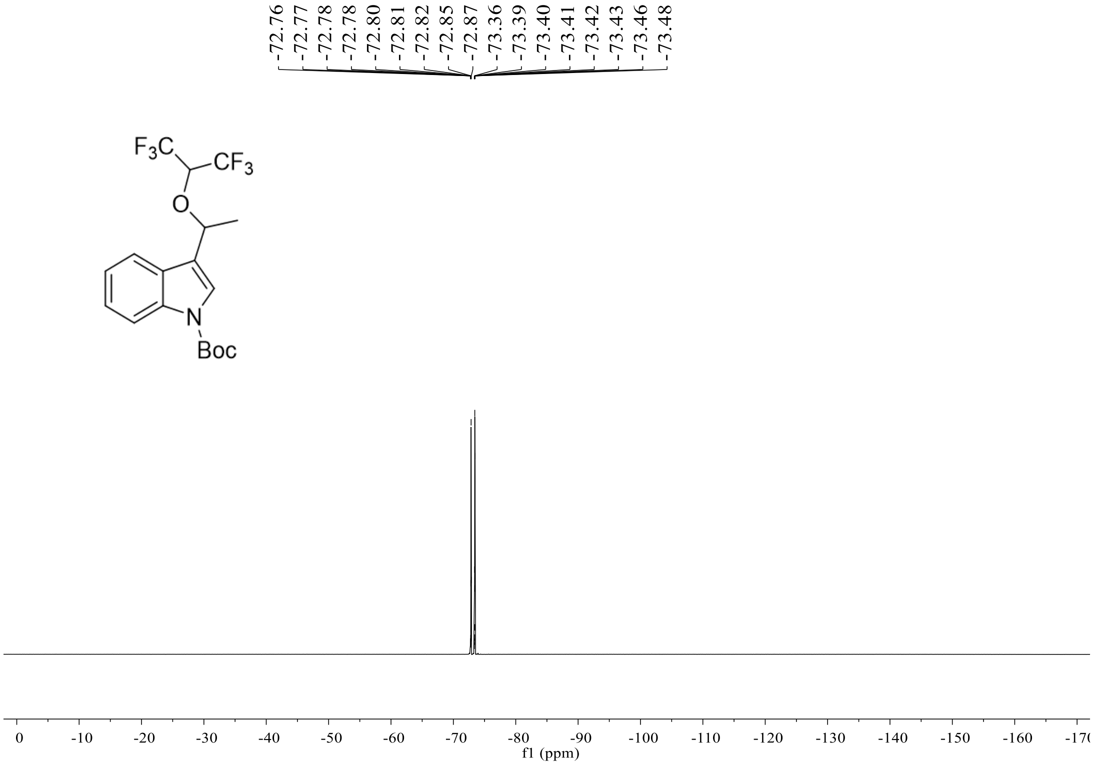

^{19}F NMR spectrum in $CDCl_3$.

94d

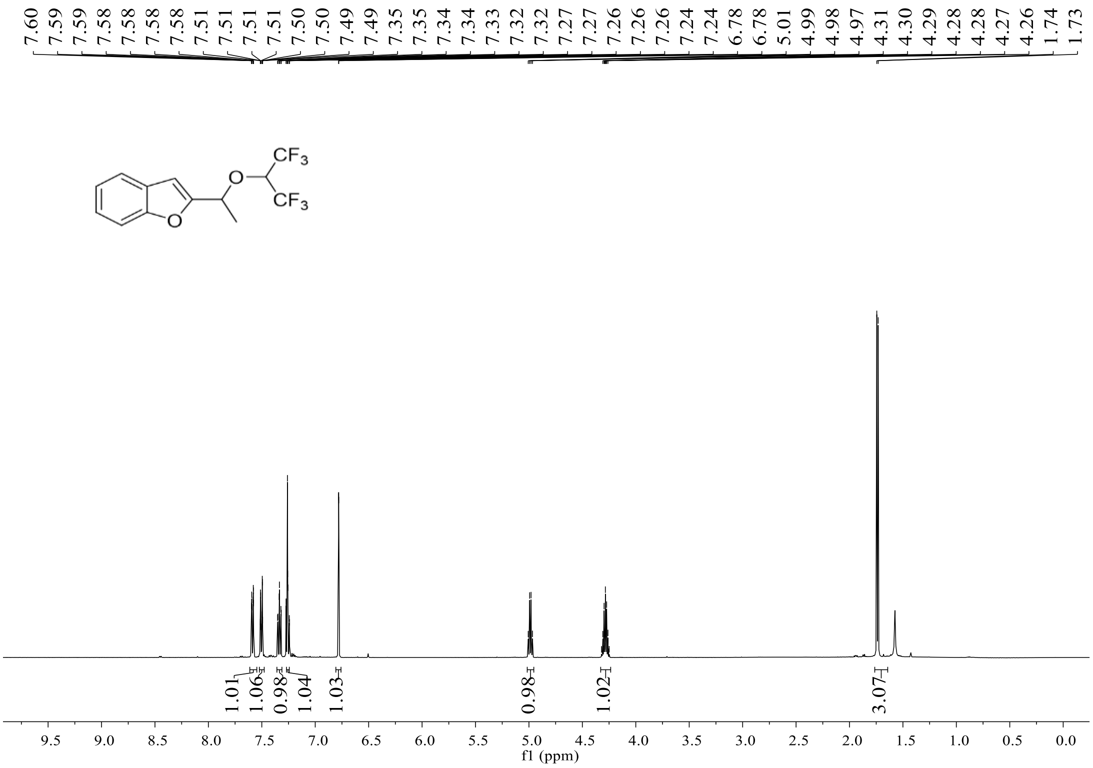

^{1}H NMR spectrum in $CDCl_3$.

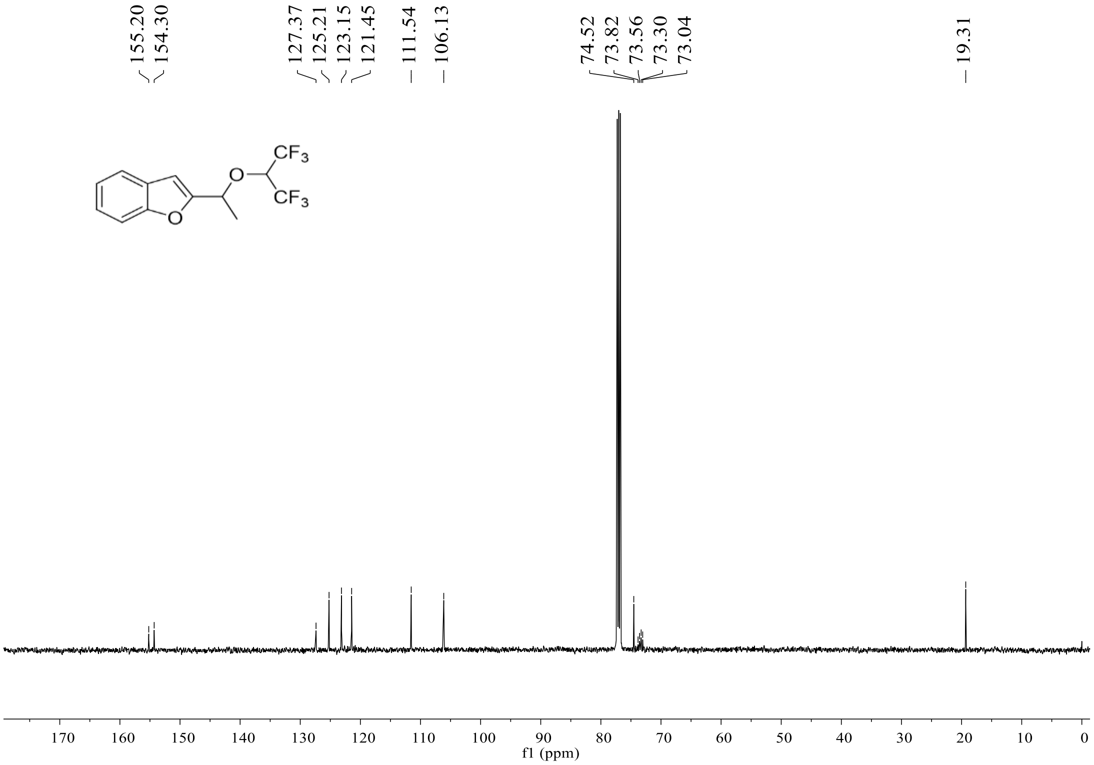

^{13}C NMR spectrum in $CDCl_3$.

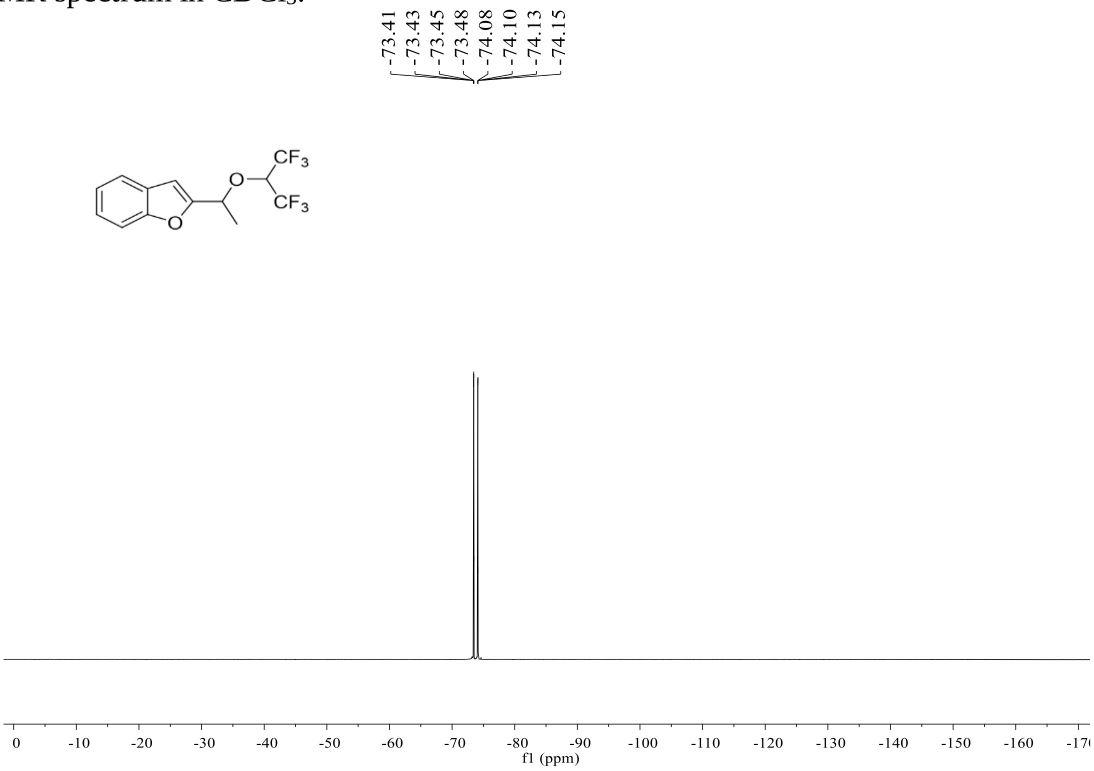

^{19}F NMR spectrum in $CDCl_3$.

95d

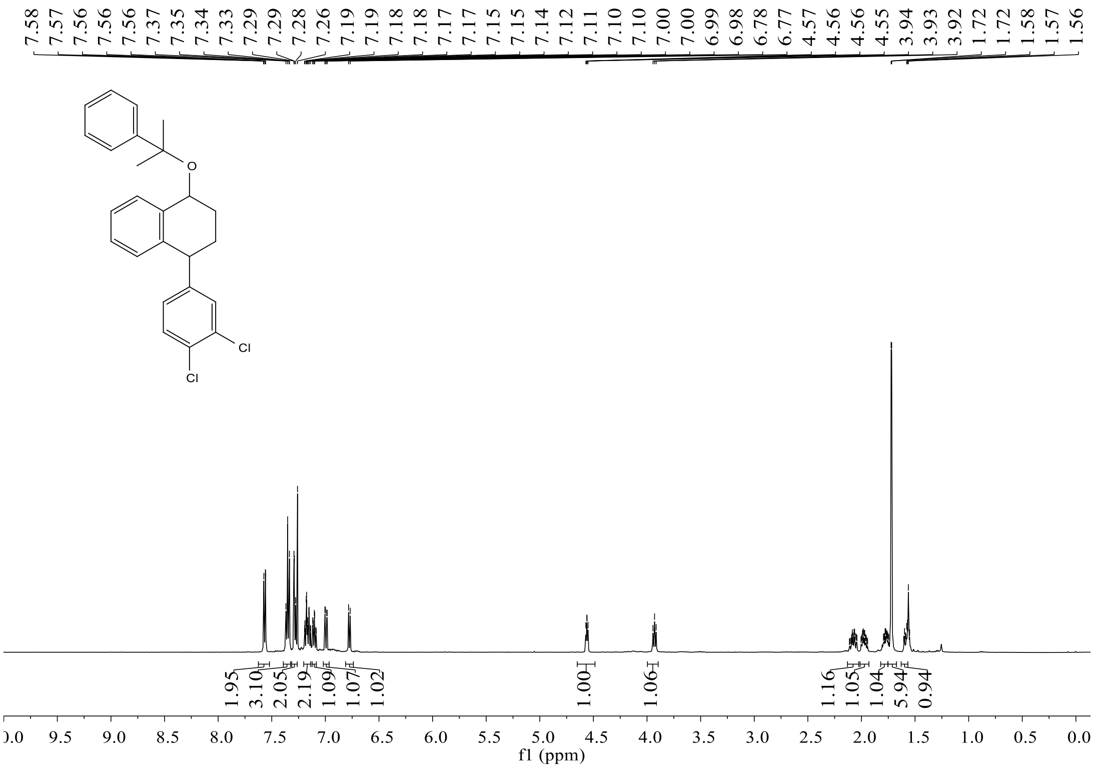

^{1}H NMR spectrum in $CDCl_3$.

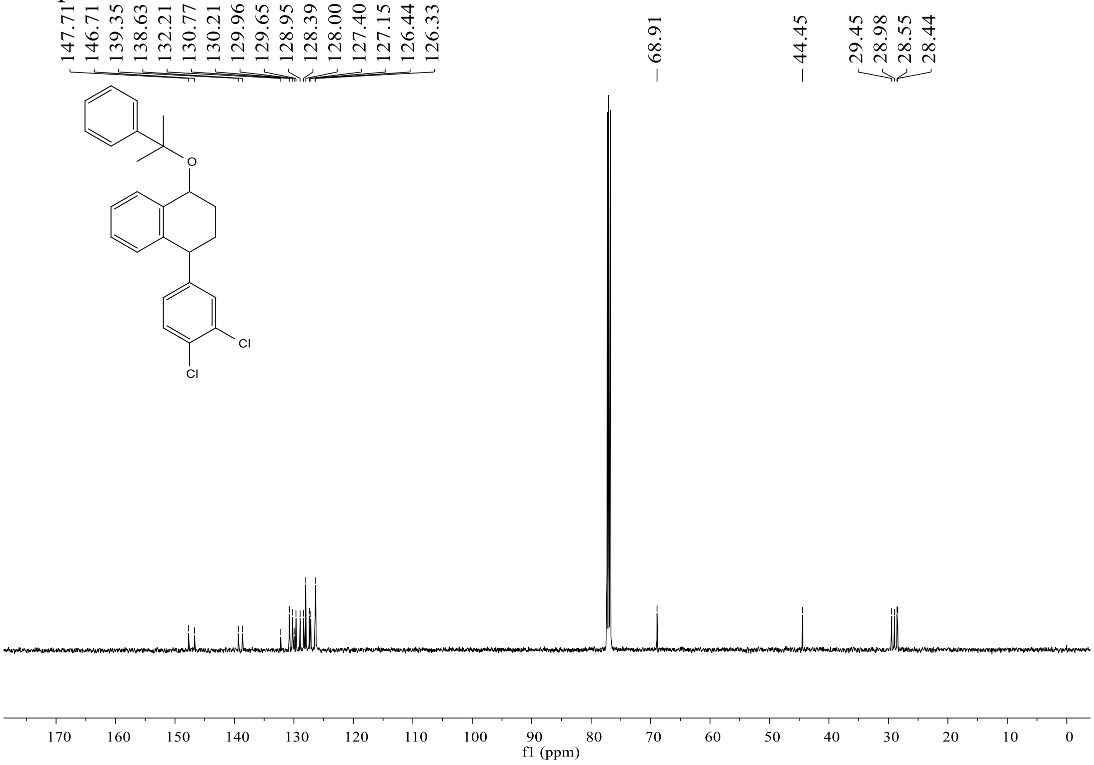

^{13}C NMR spectrum in $CDCl_3$.

96d

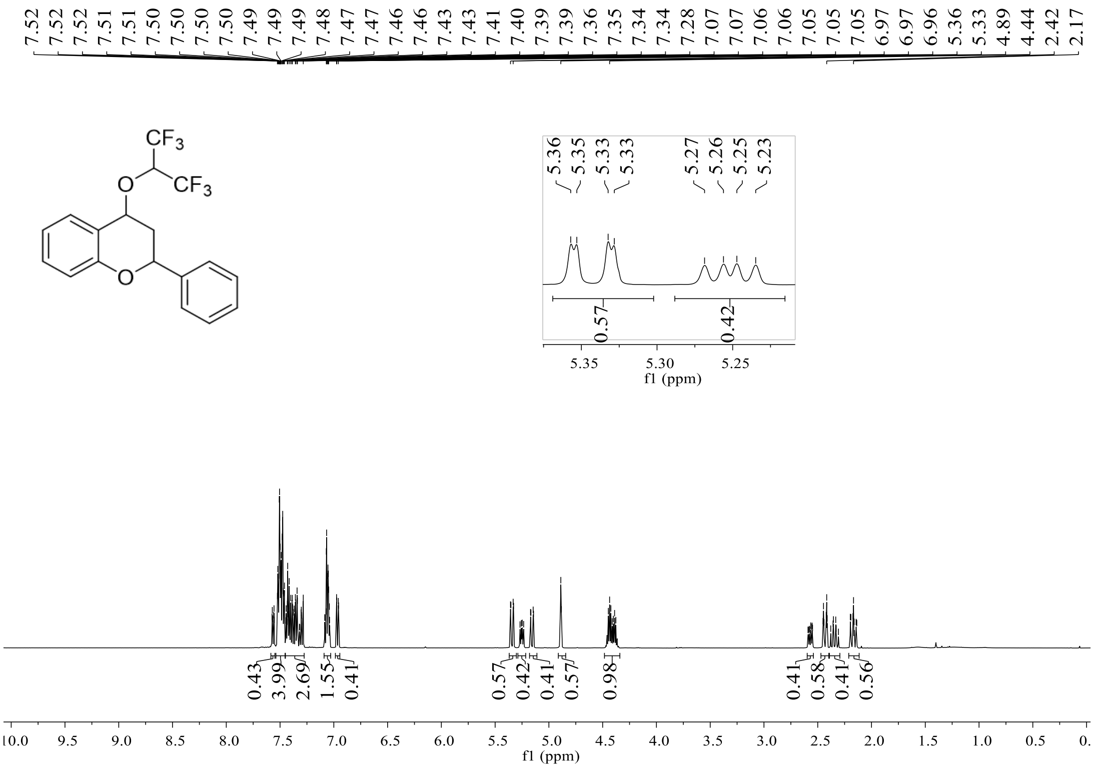

^{1}H NMR spectrum in $CDCl_3$.

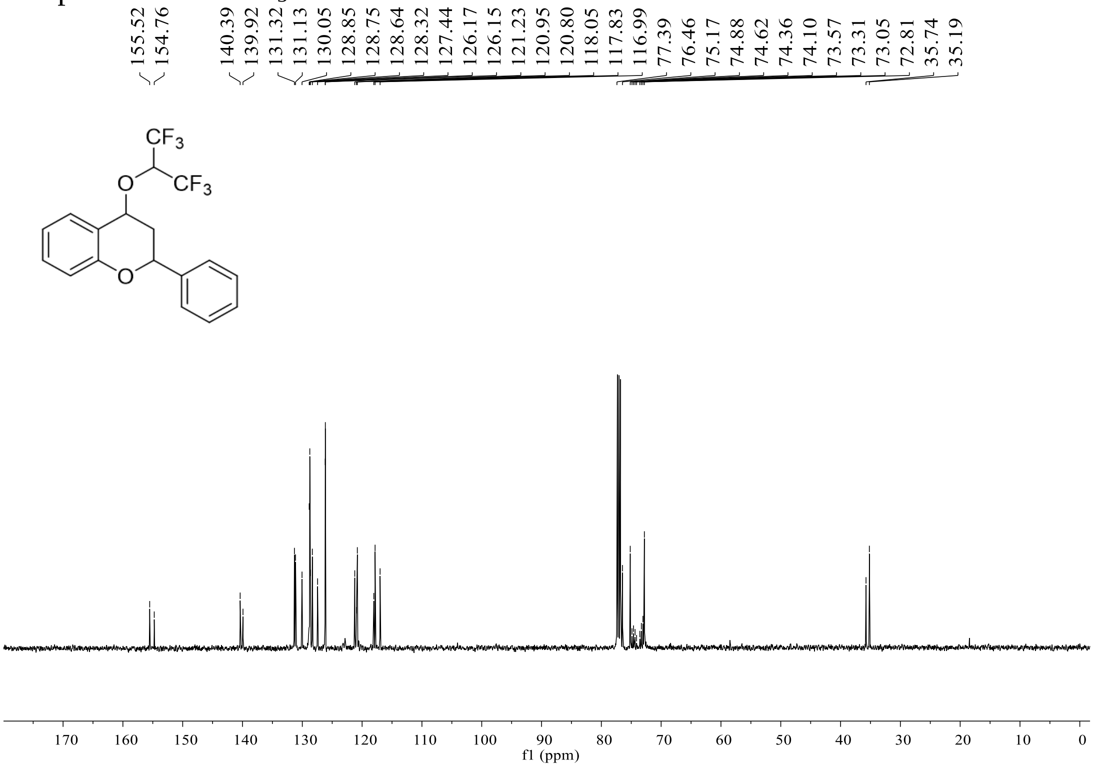

^{13}C NMR spectrum in $CDCl_3$.

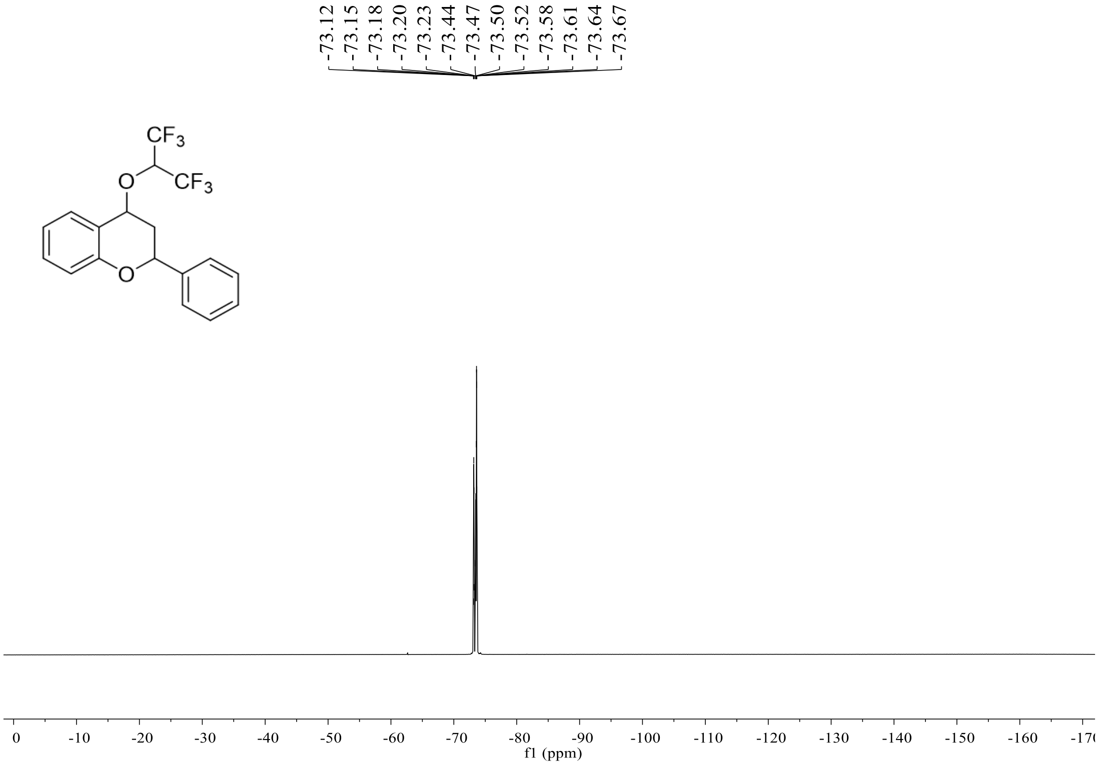

^{19}F NMR spectrum in $CDCl_3$.

96e

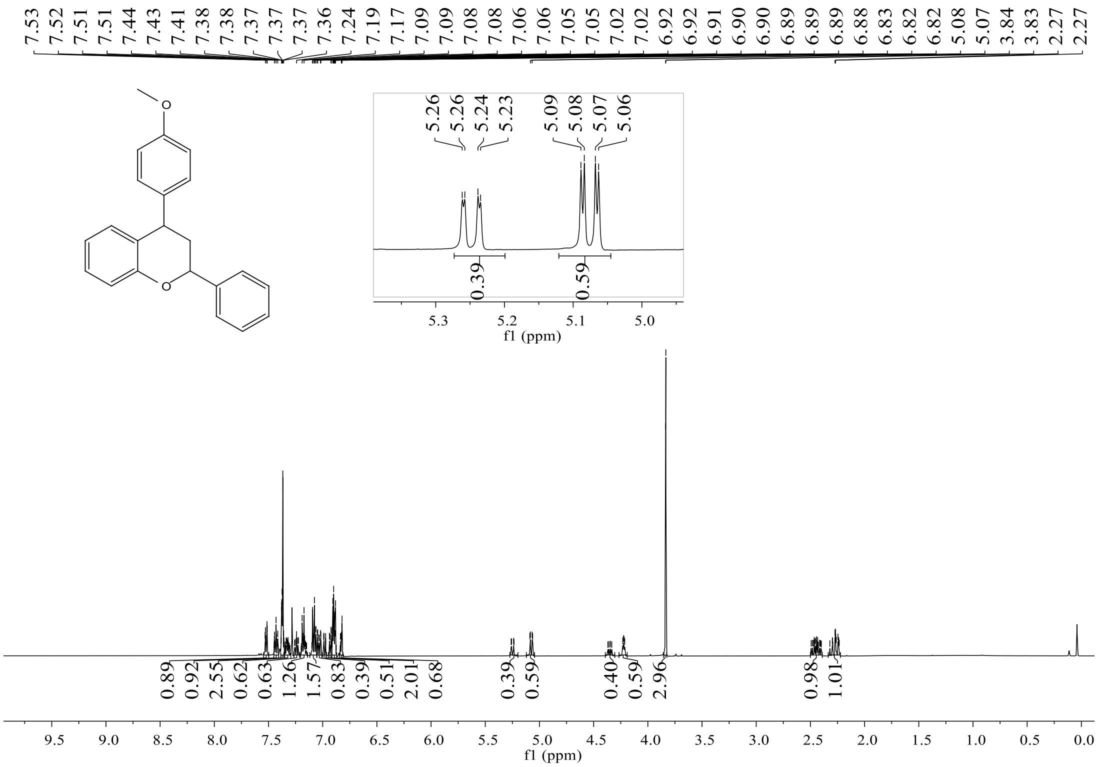

^{1}H NMR spectrum in $CDCl_3$.

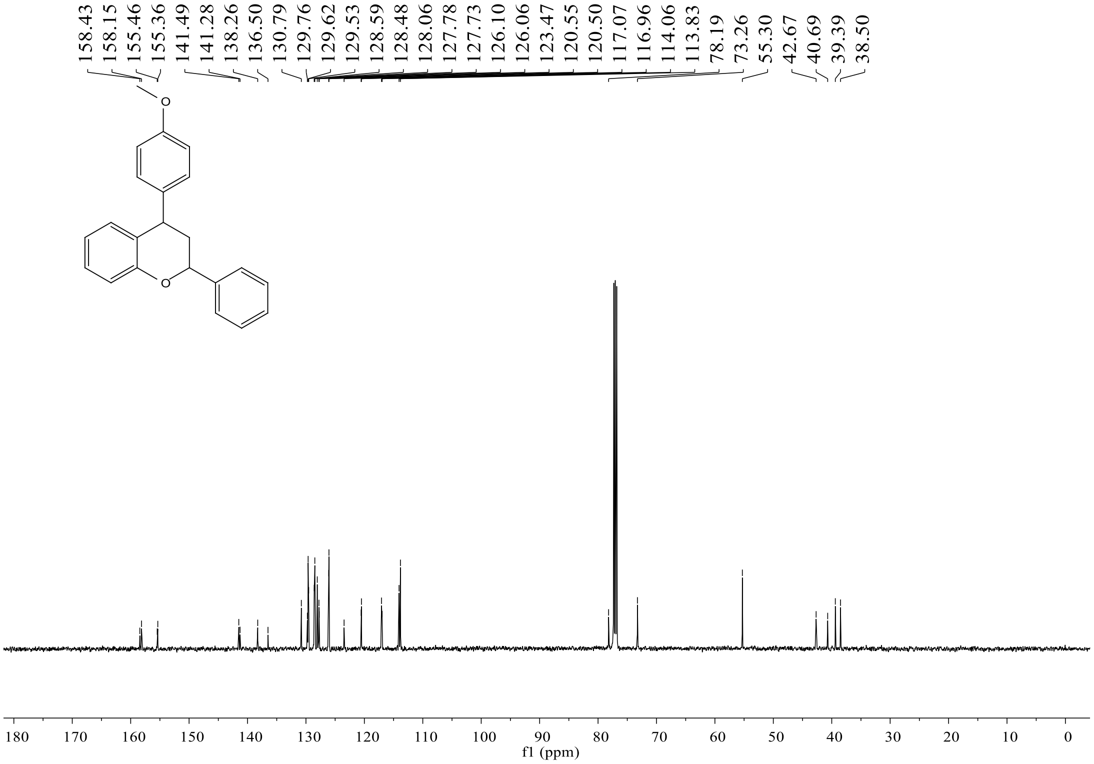

^{13}C NMR spectrum in $CDCl_3$.

96f

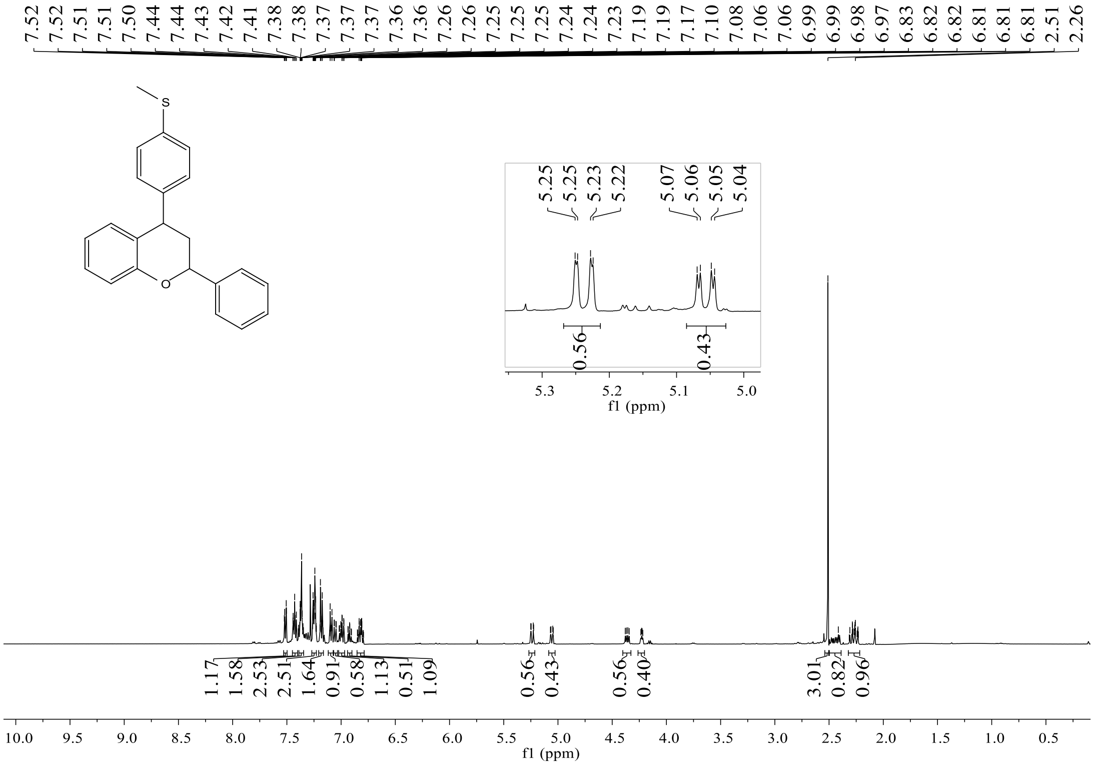

^{1}H NMR spectrum in $CDCl_3$.

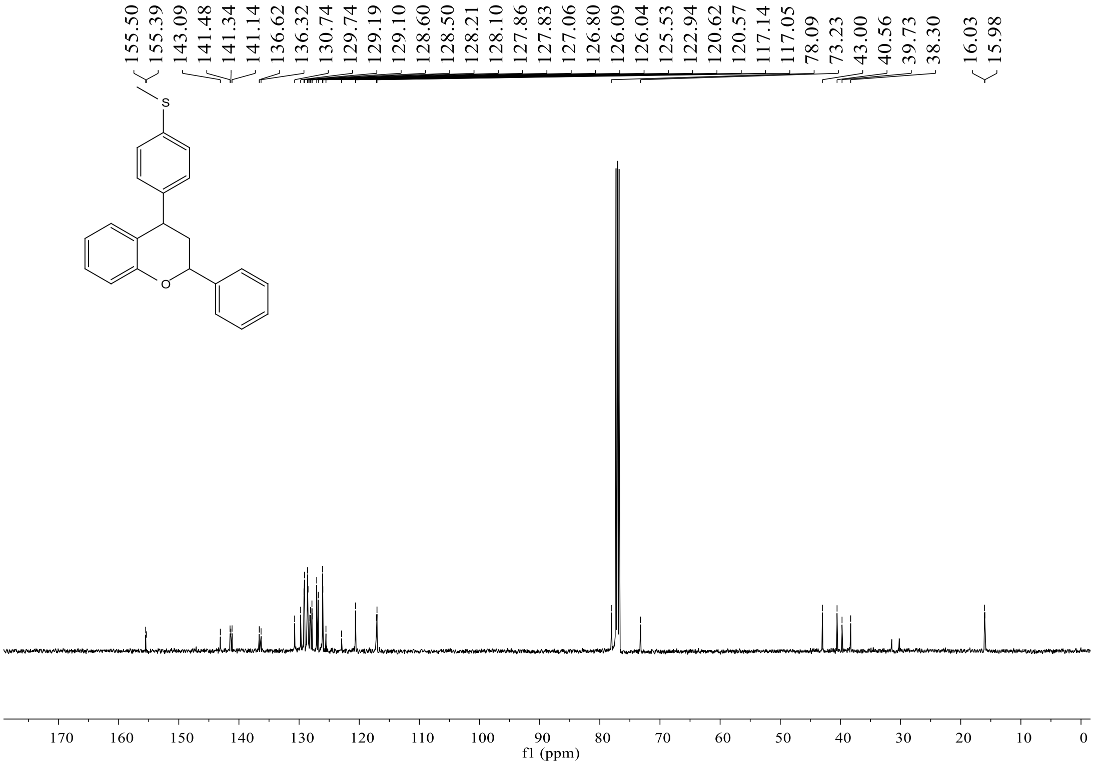

^{13}C NMR spectrum in $CDCl_3$.

97d

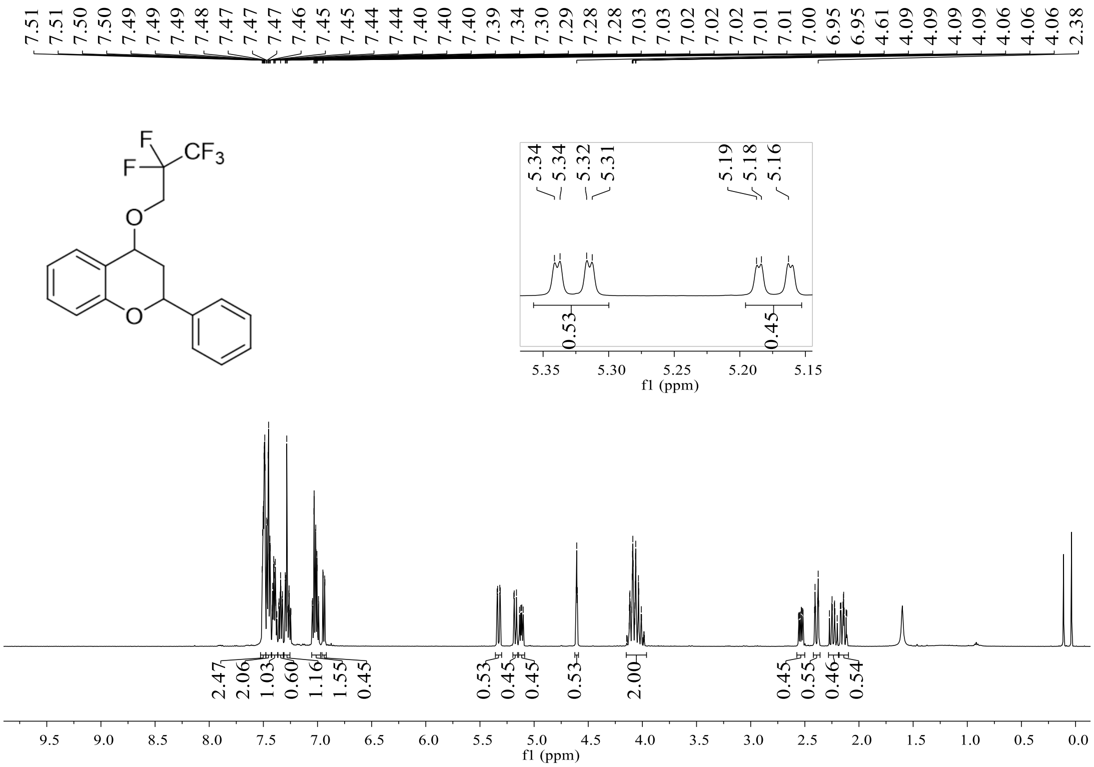

^{1}H NMR spectrum in $CDCl_3$.

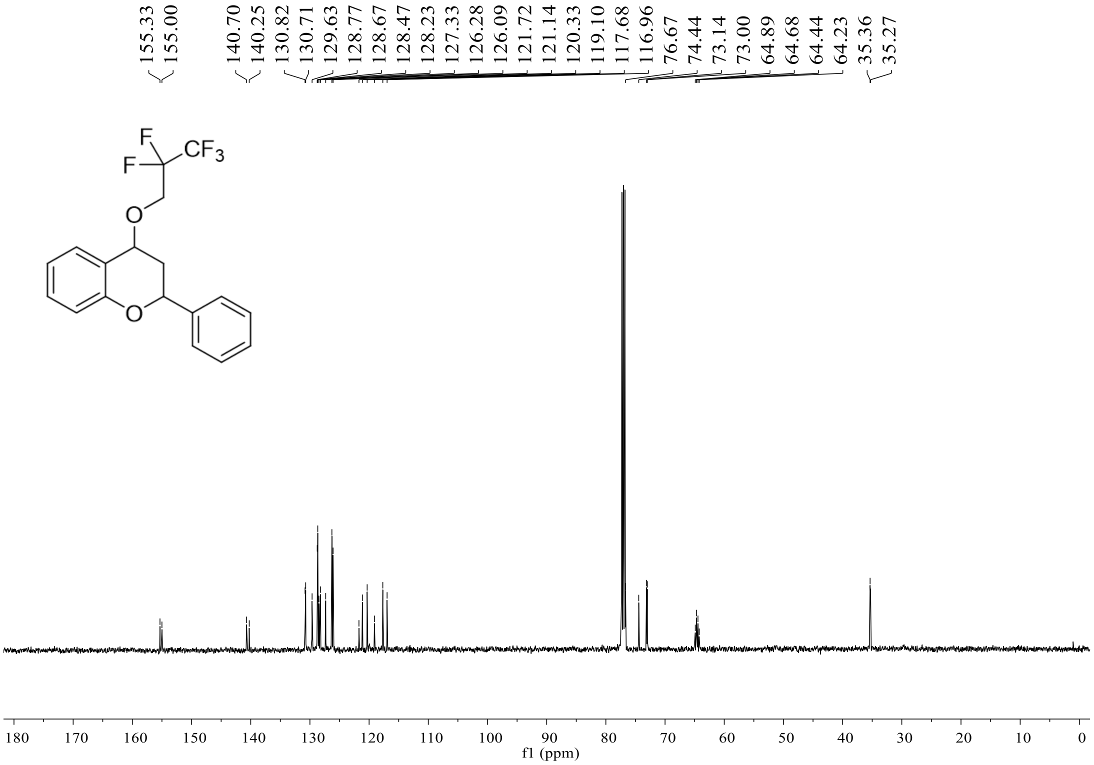

^{13}C NMR spectrum in $CDCl_3$.

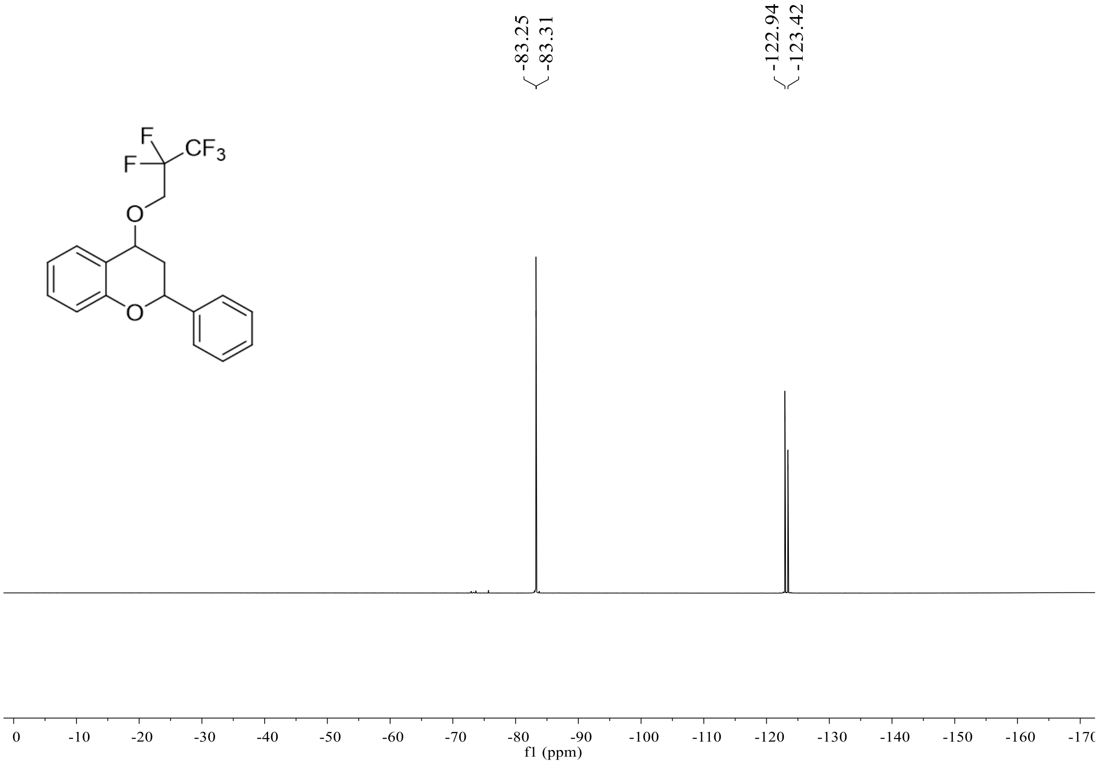

^{19}F NMR spectrum in $CDCl_3$.

98d

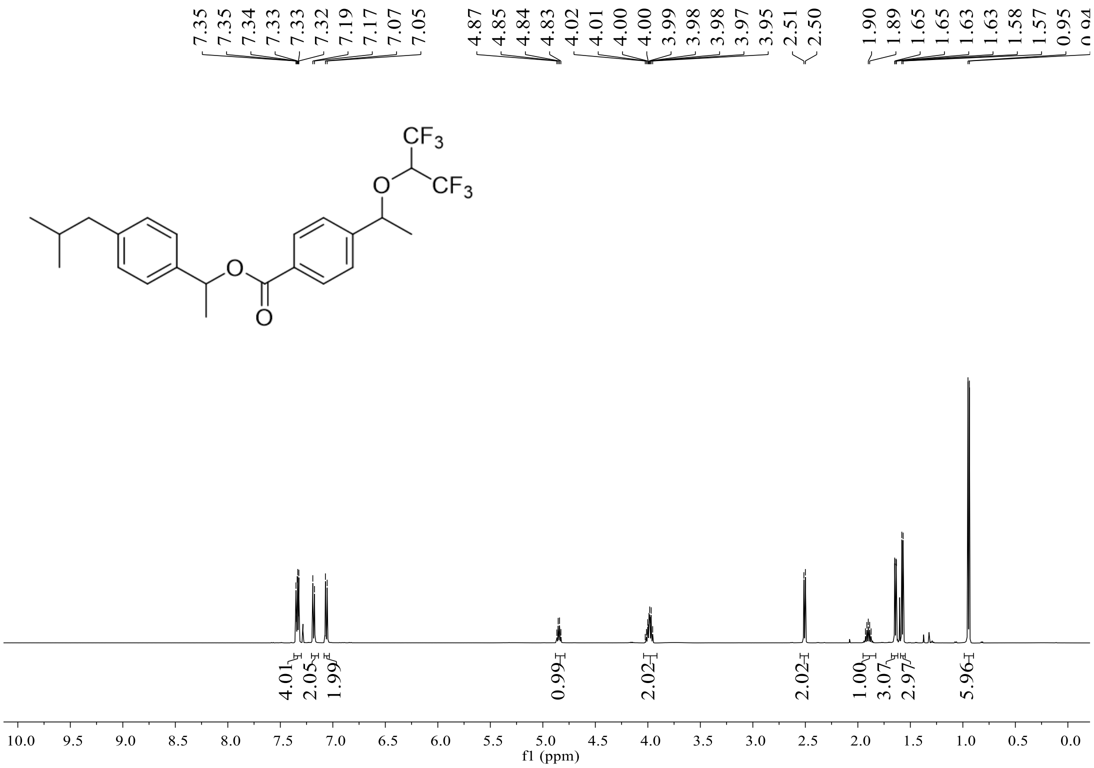

^{1}H NMR spectrum in $CDCl_3$.

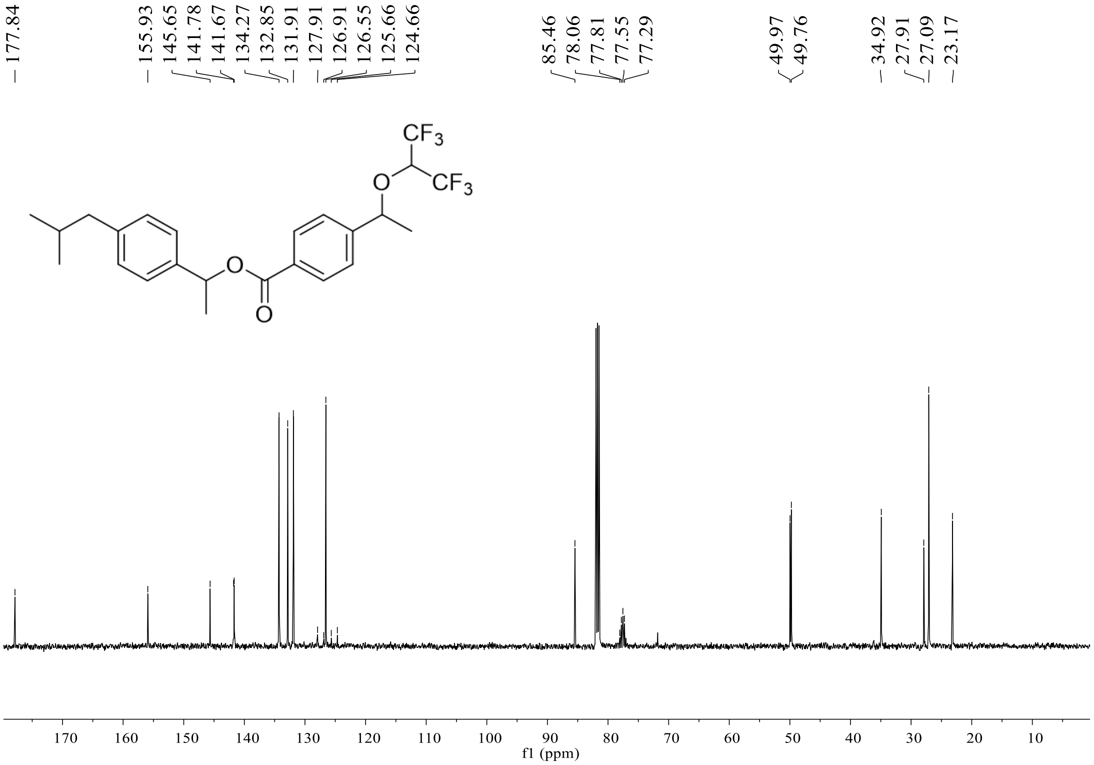

^{13}C NMR spectrum in $CDCl_3$.

99d

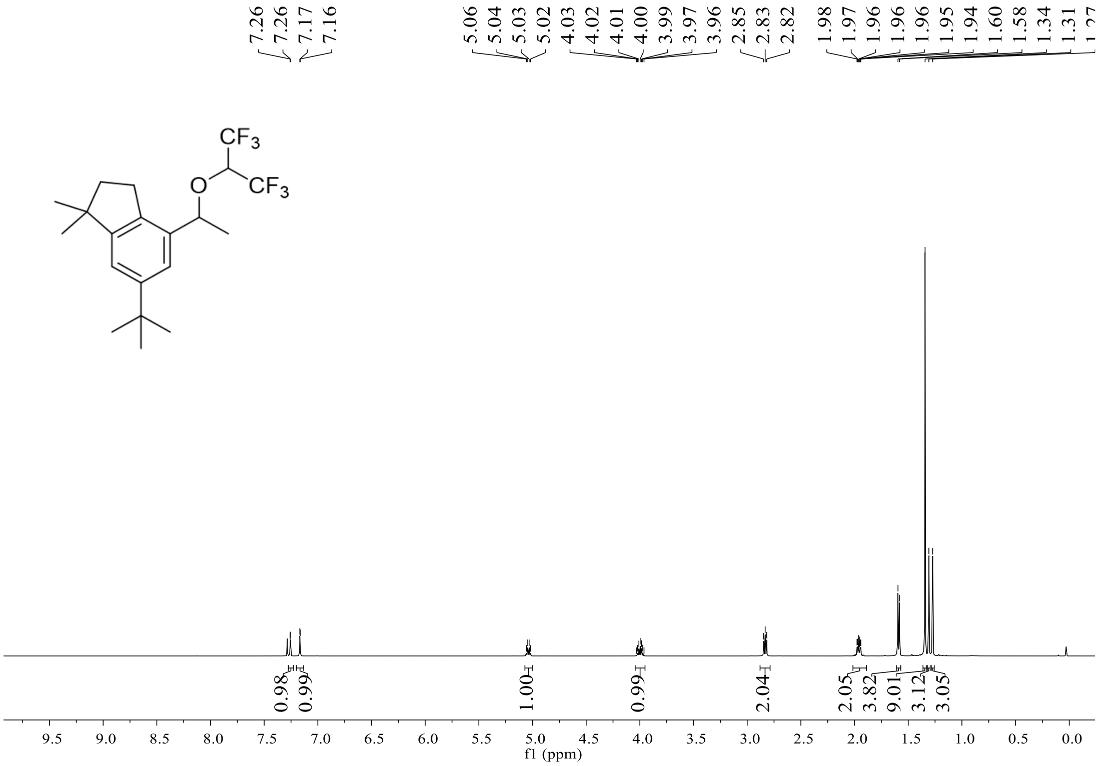

^{1}H NMR spectrum in $CDCl_3$.

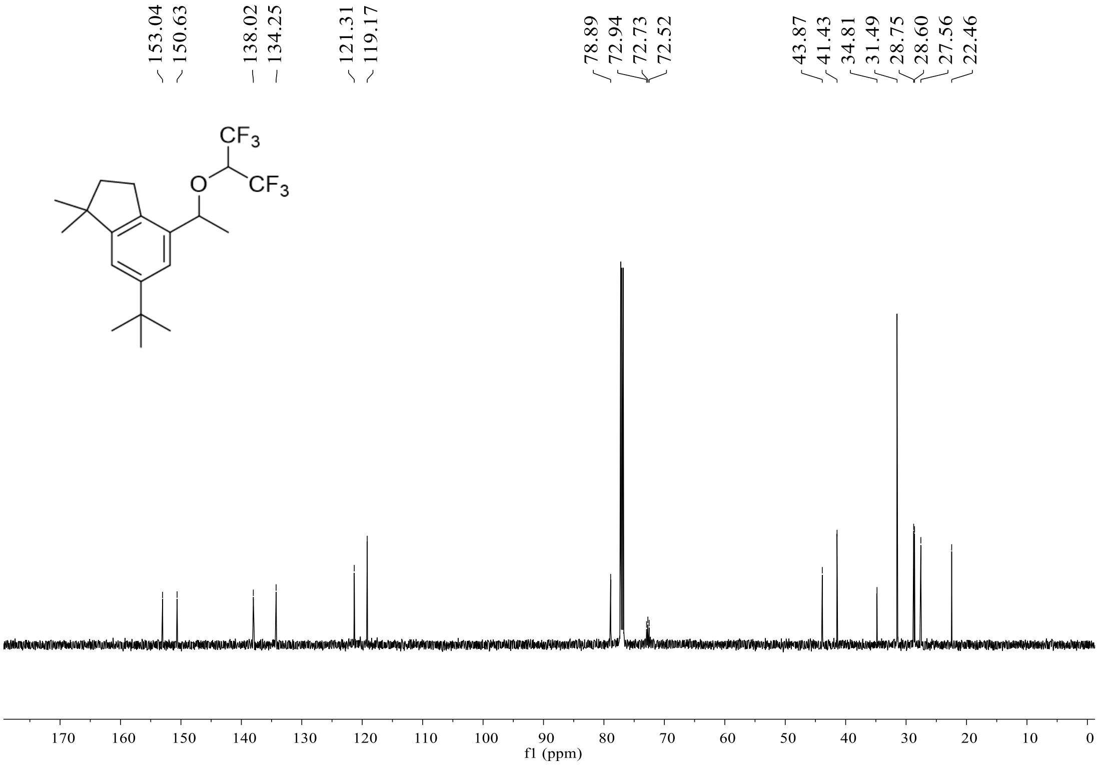

^{13}C NMR spectrum in $CDCl_3$.

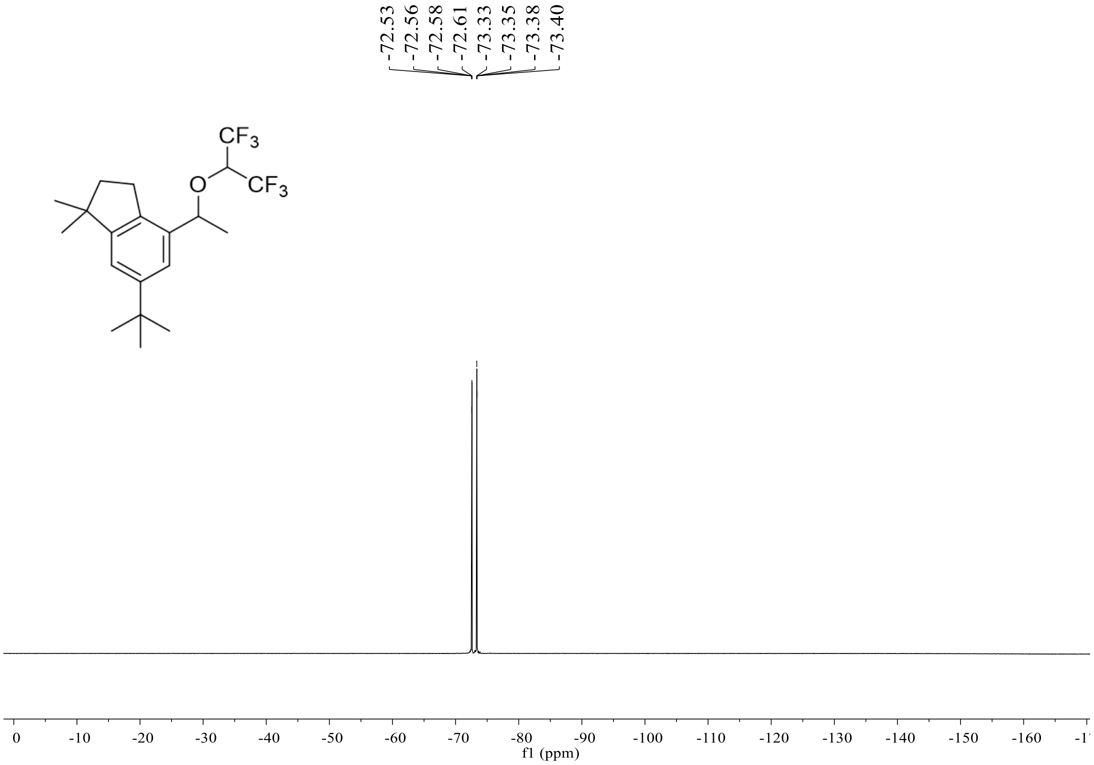

^{19}F NMR spectrum in $CDCl_3$.

100d

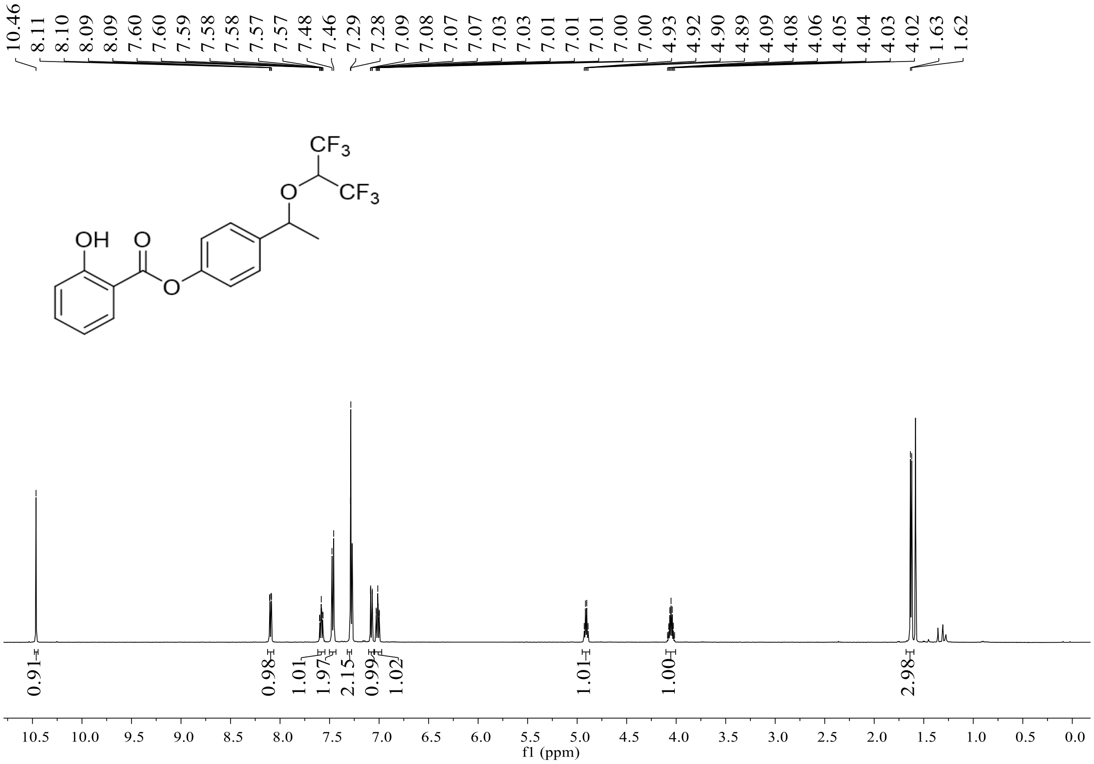

^{1}H NMR spectrum in $CDCl_3$.

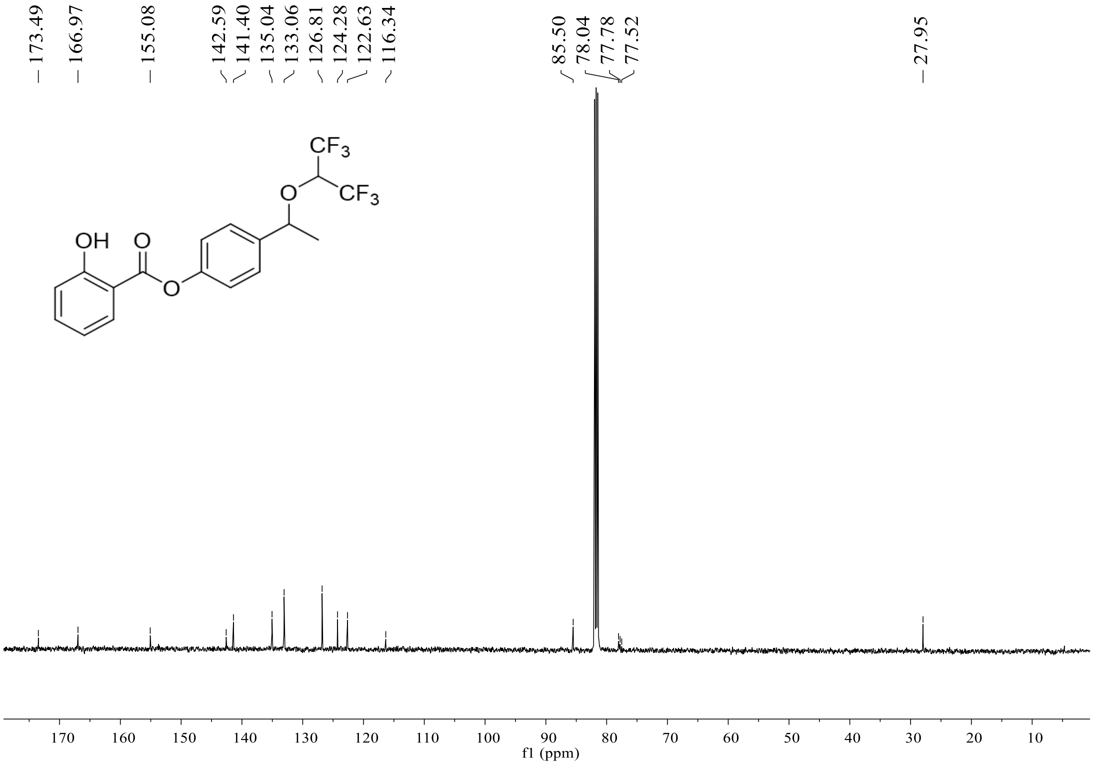

^{13}C NMR spectrum in $CDCl_3$.

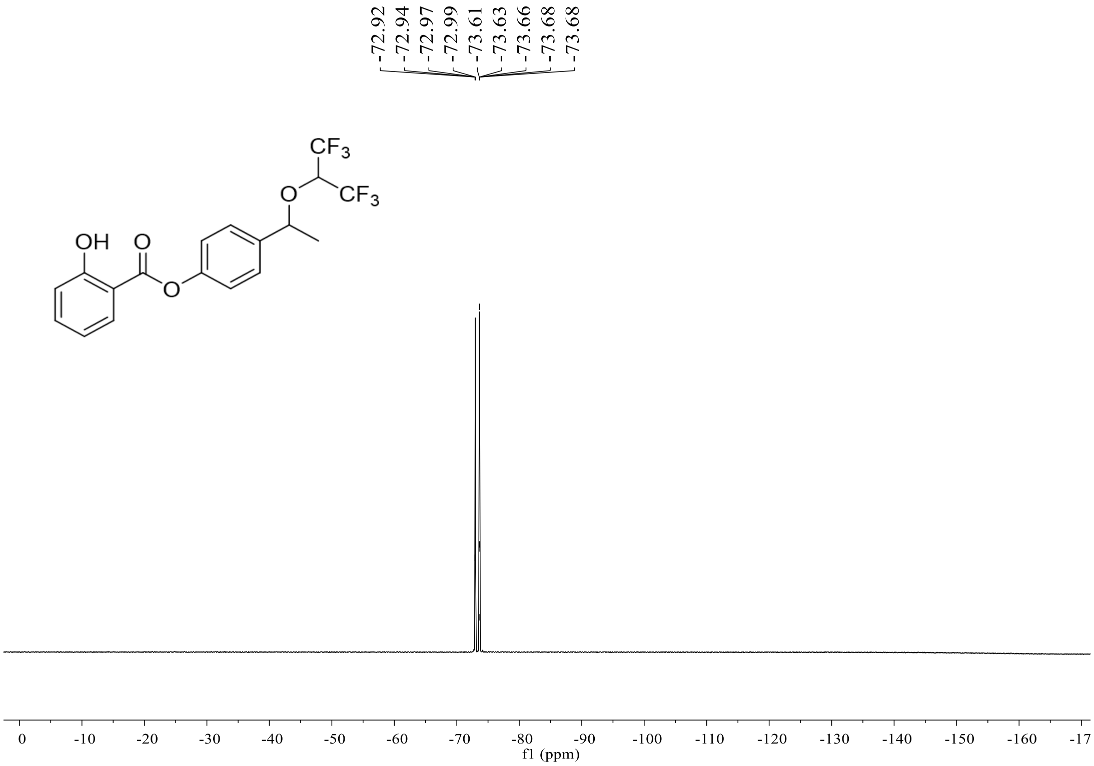

^{19}F NMR spectrum in $CDCl_3$.

101d

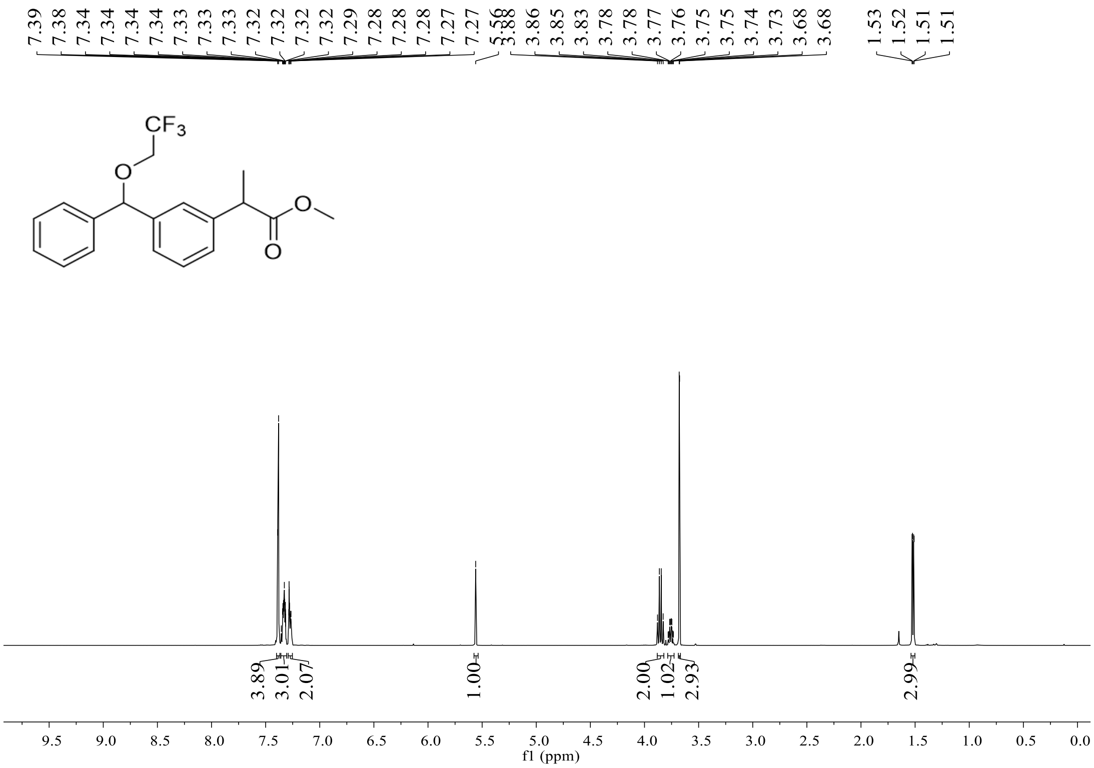

^{1}H NMR spectrum in $CDCl_3$.

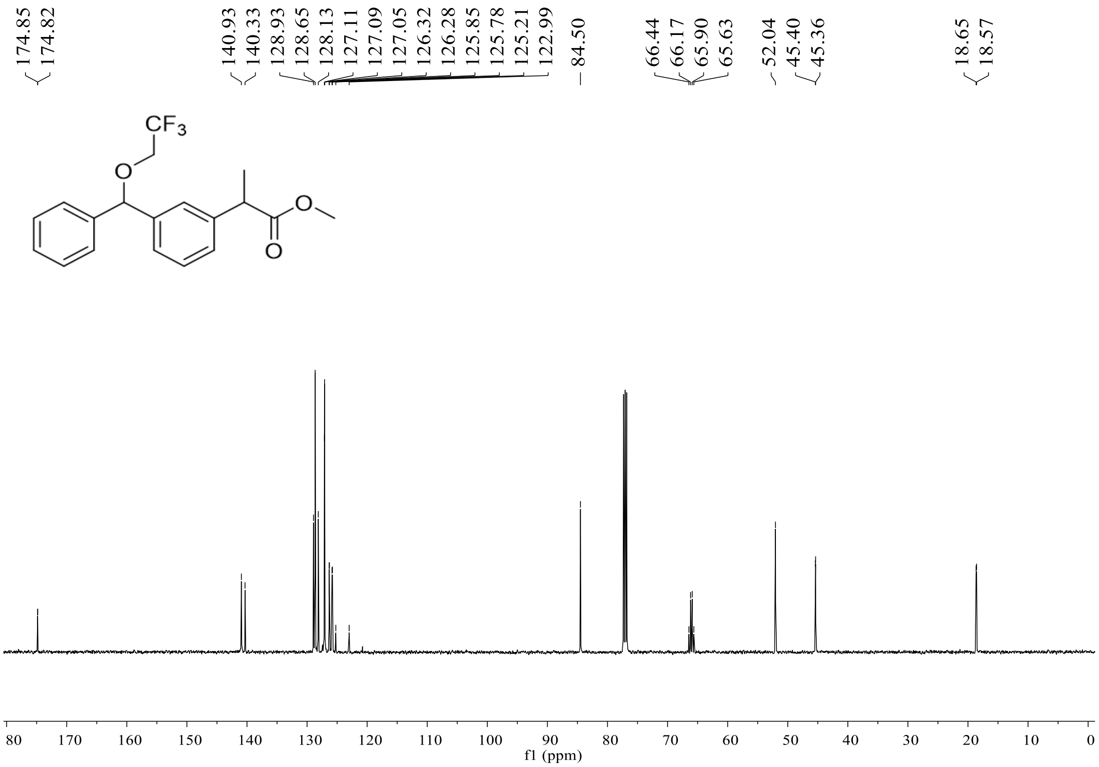

^{13}C NMR spectrum in $CDCl_3$.

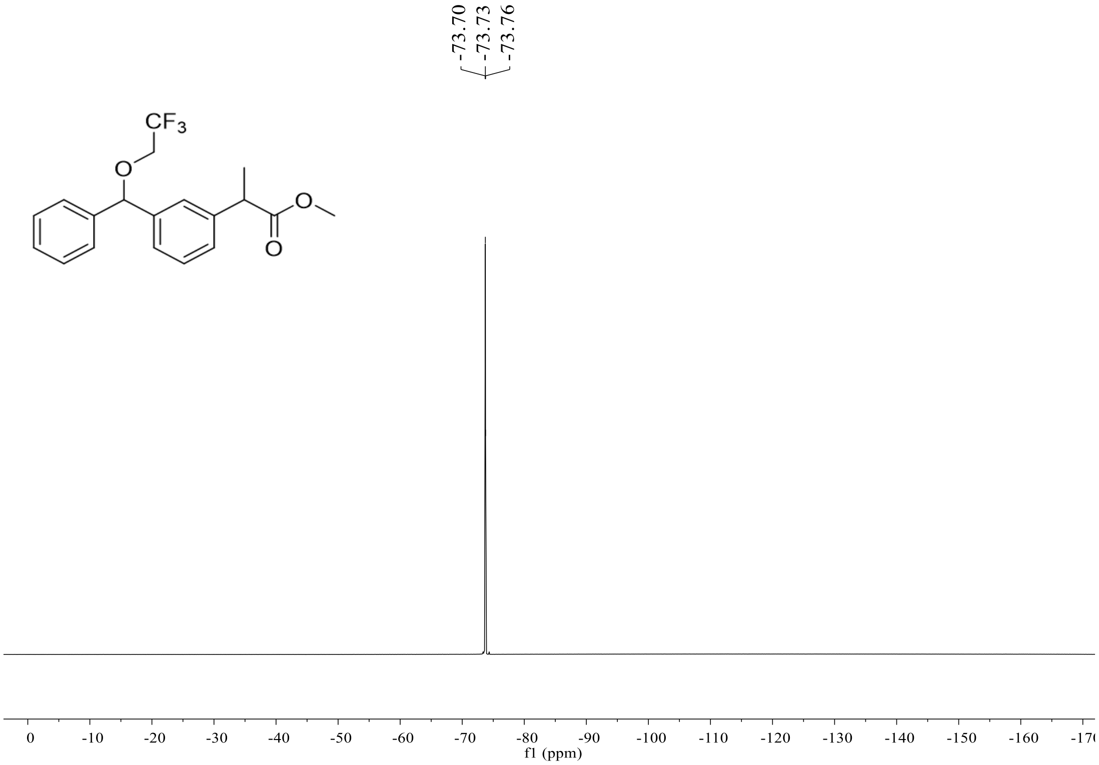

^{19}F NMR spectrum in $CDCl_3$.

102d

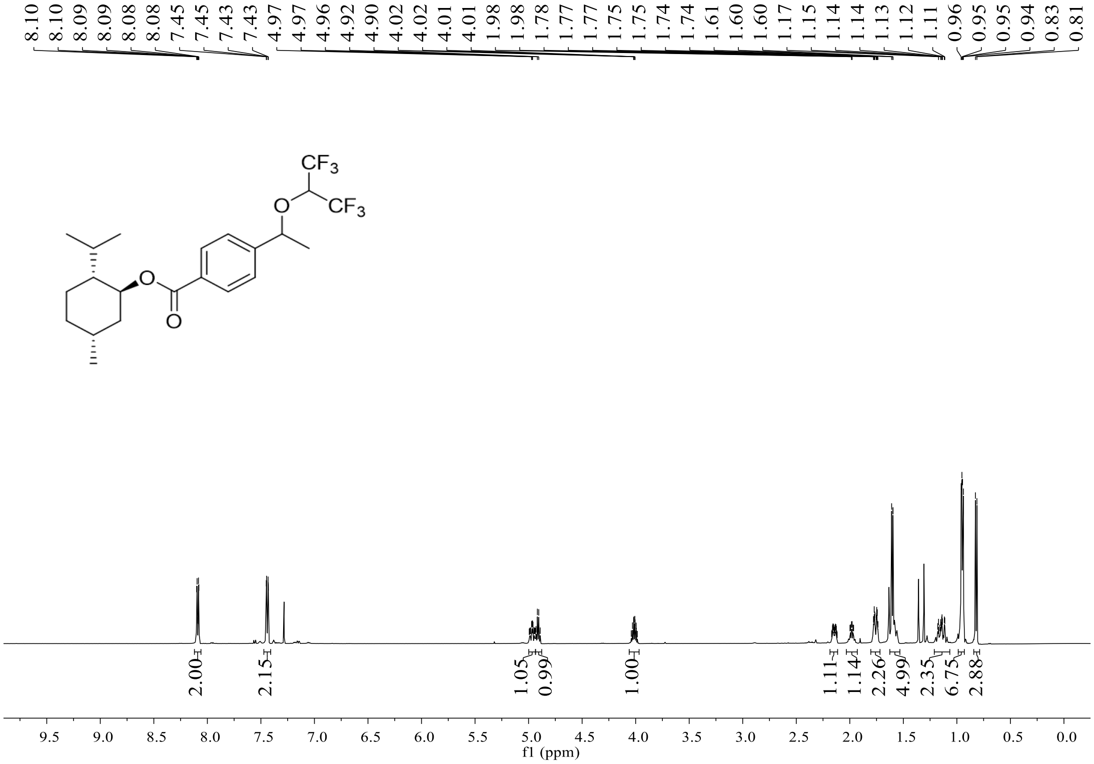

^{1}H NMR spectrum in $CDCl_3$.

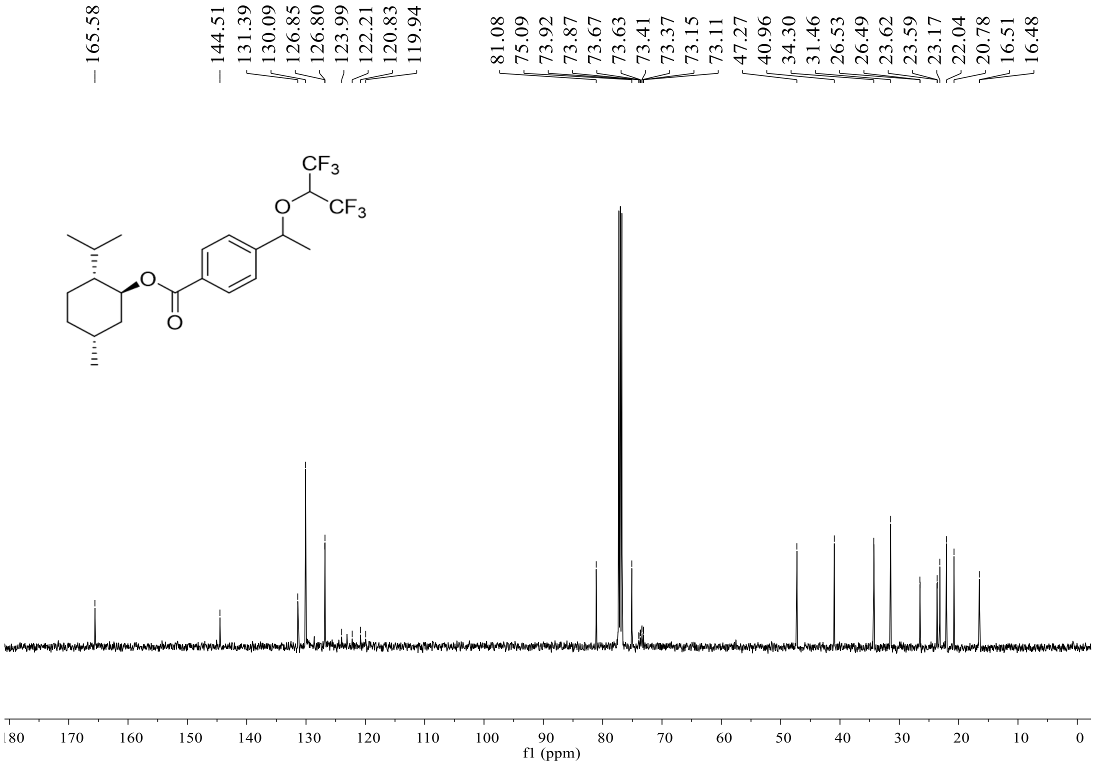

^{13}C NMR spectrum in $CDCl_3$.

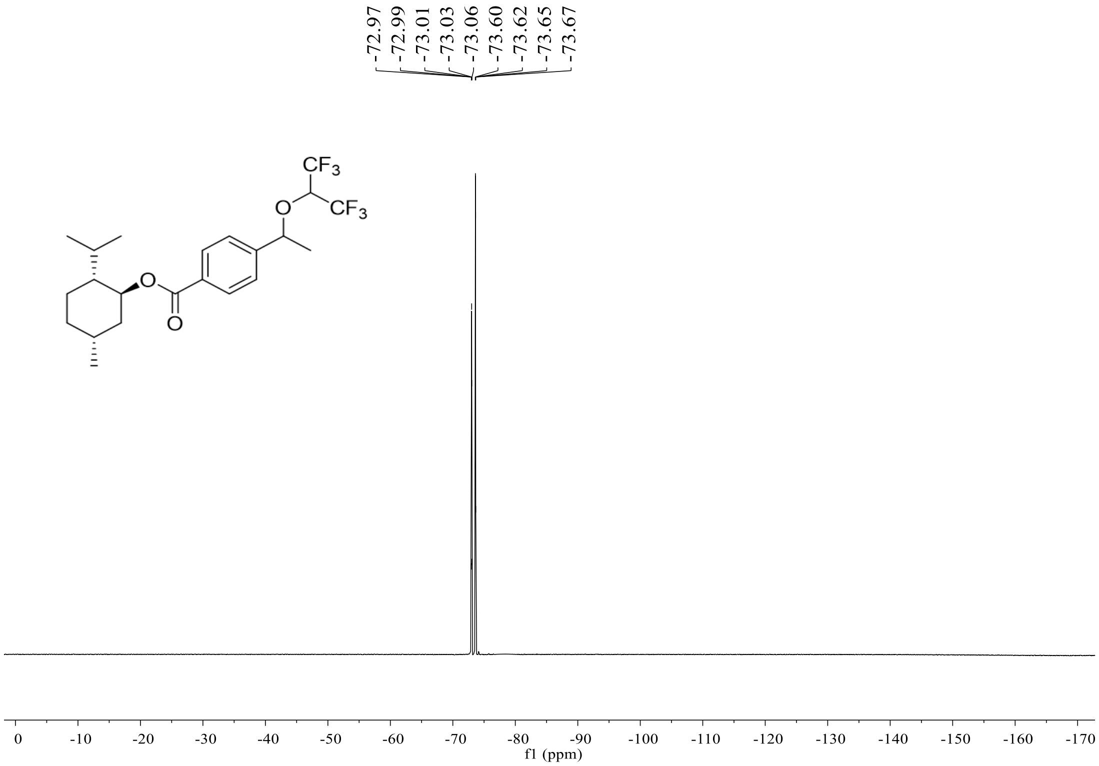

^{19}F NMR spectrum in $CDCl_3$.

103d

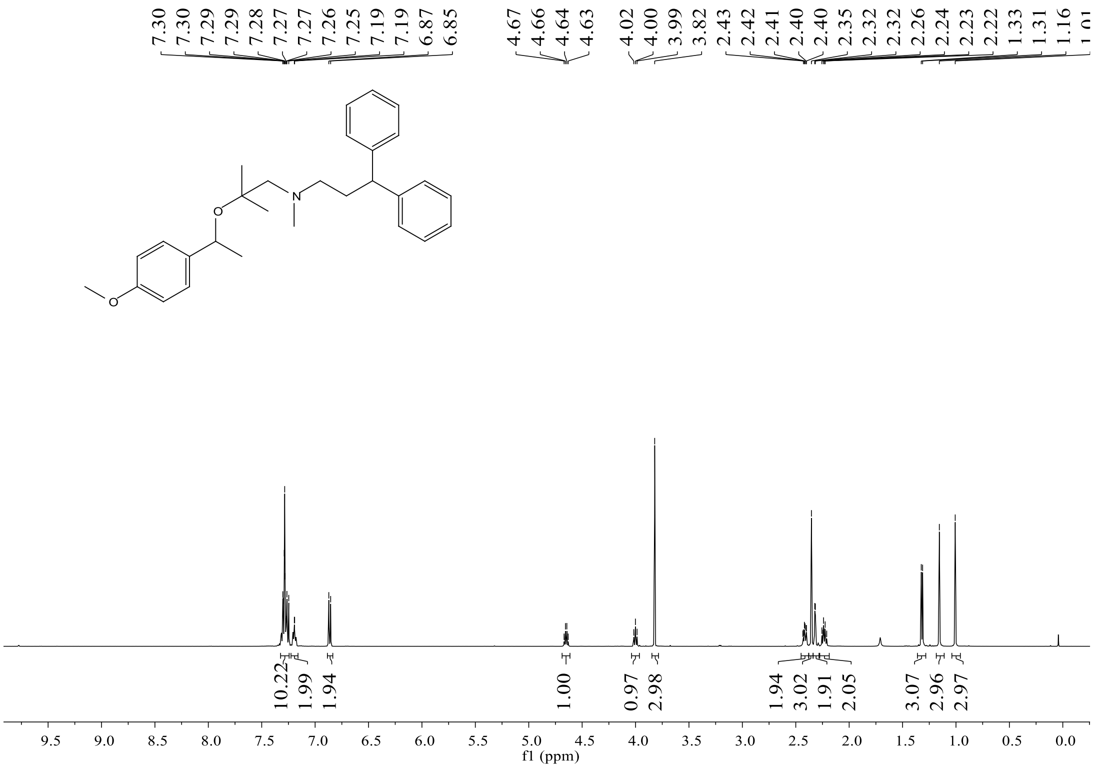

^{1}H NMR spectrum in $CDCl_3$.

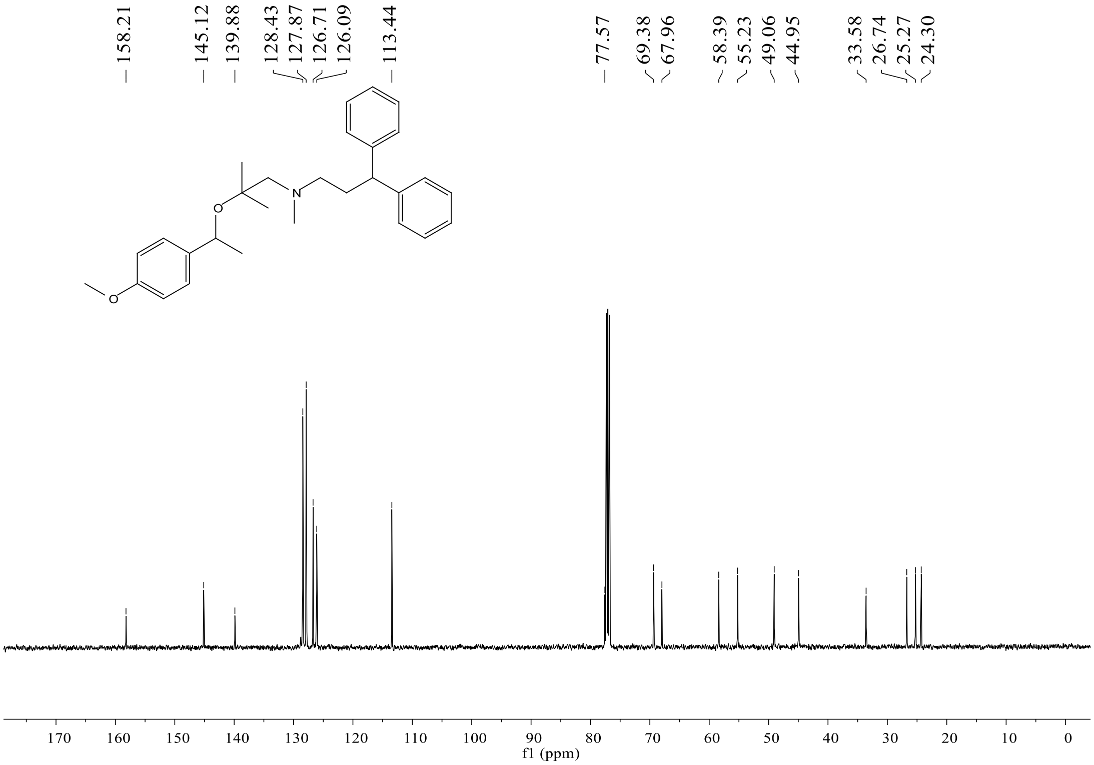

^{13}C NMR spectrum in $CDCl_3$.

104d

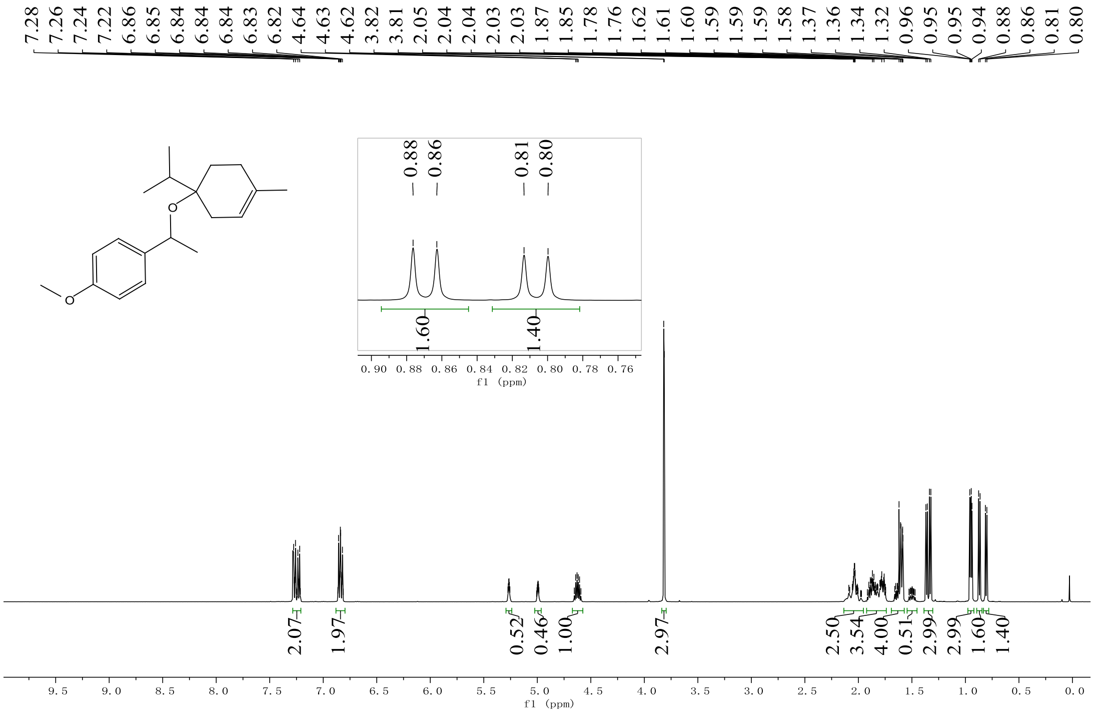

^{1}H NMR spectrum in $CDCl_3$.

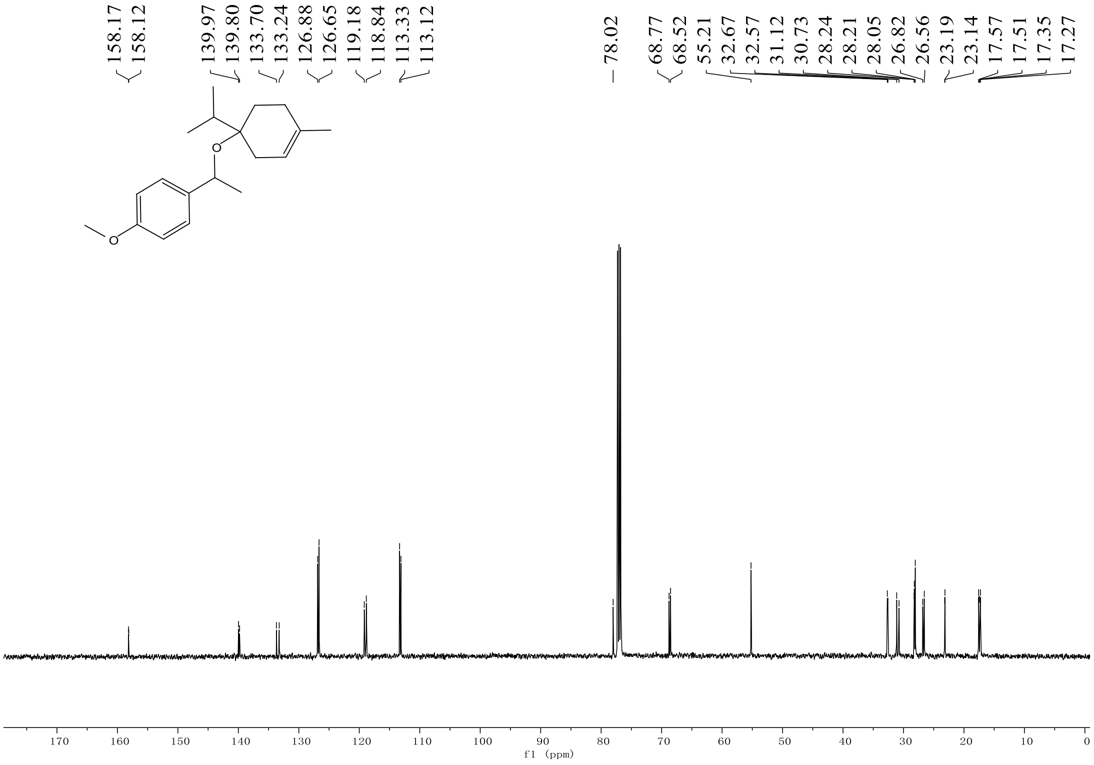

^{13}C NMR spectrum in $CDCl_3$.

105d

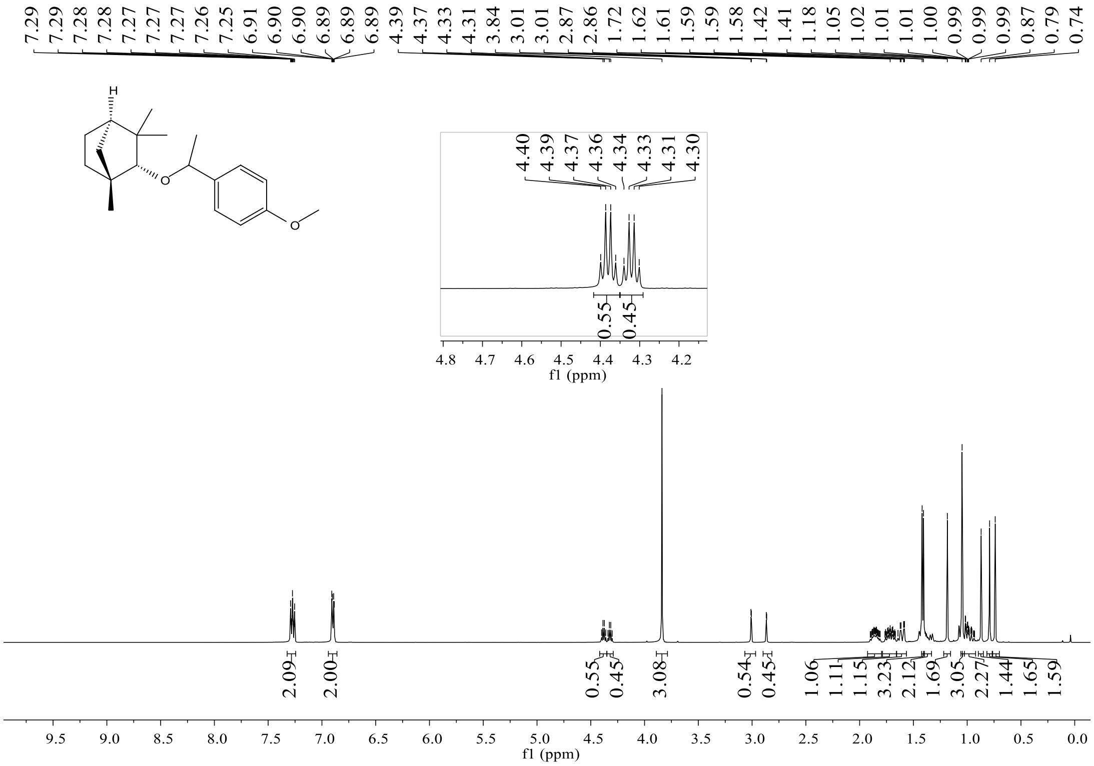

^{1}H NMR spectrum in $CDCl_3$.

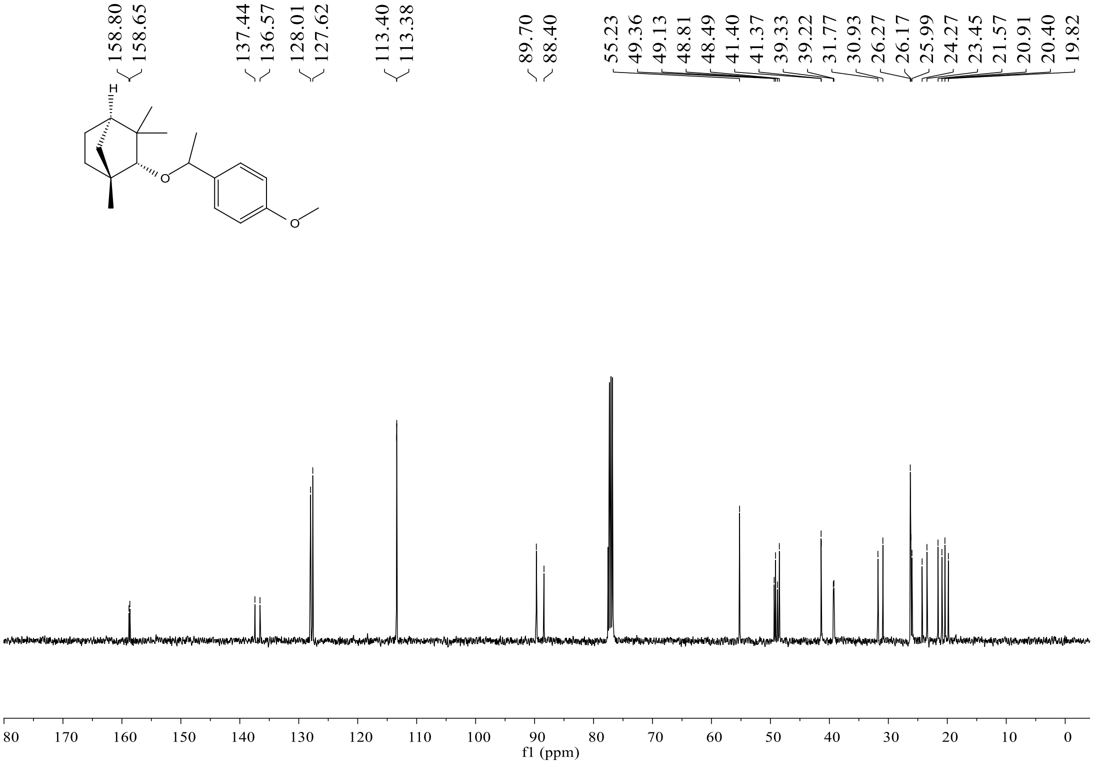

^{13}C NMR spectrum in $CDCl_3$.

106d

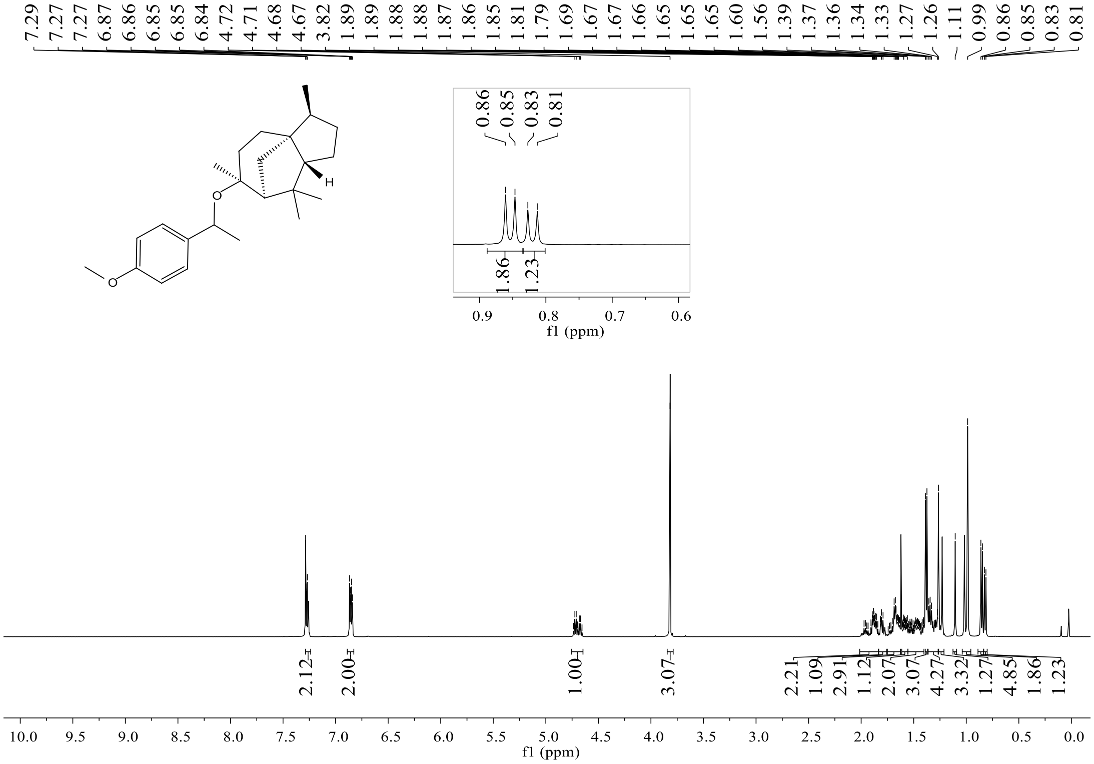

^{1}H NMR spectrum in $CDCl_3$.

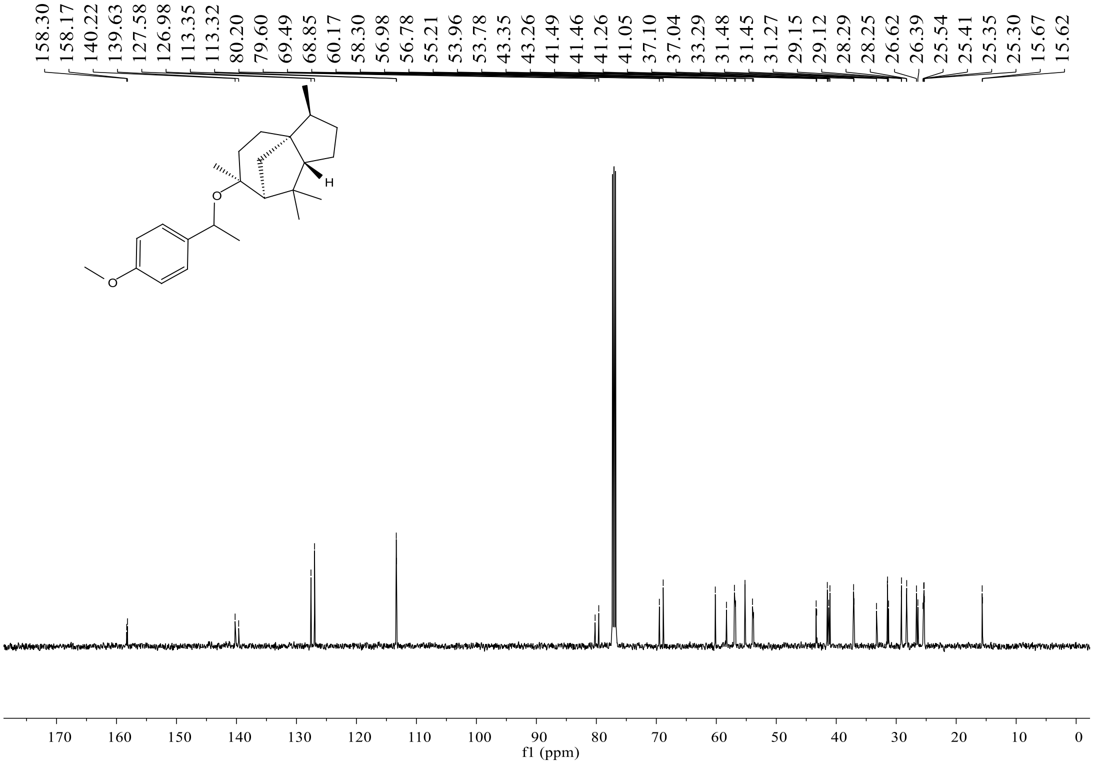

^{13}C NMR spectrum in $CDCl_3$.

107d

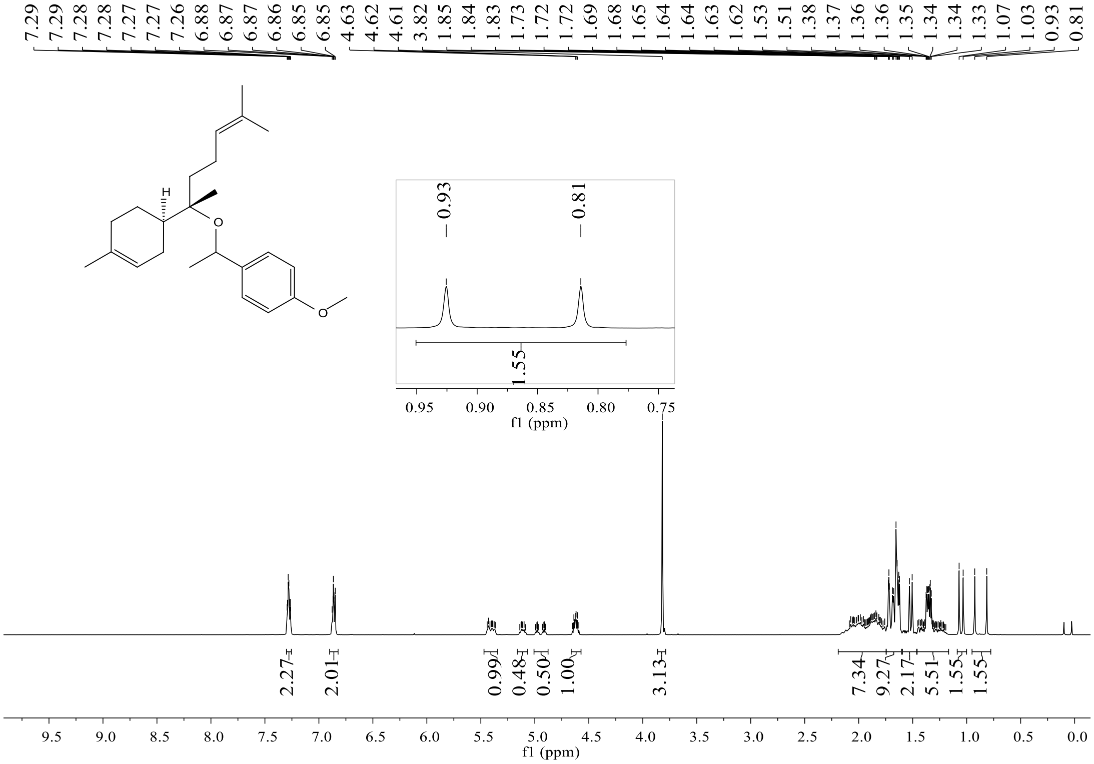

^{1}H NMR spectrum in $CDCl_3$.

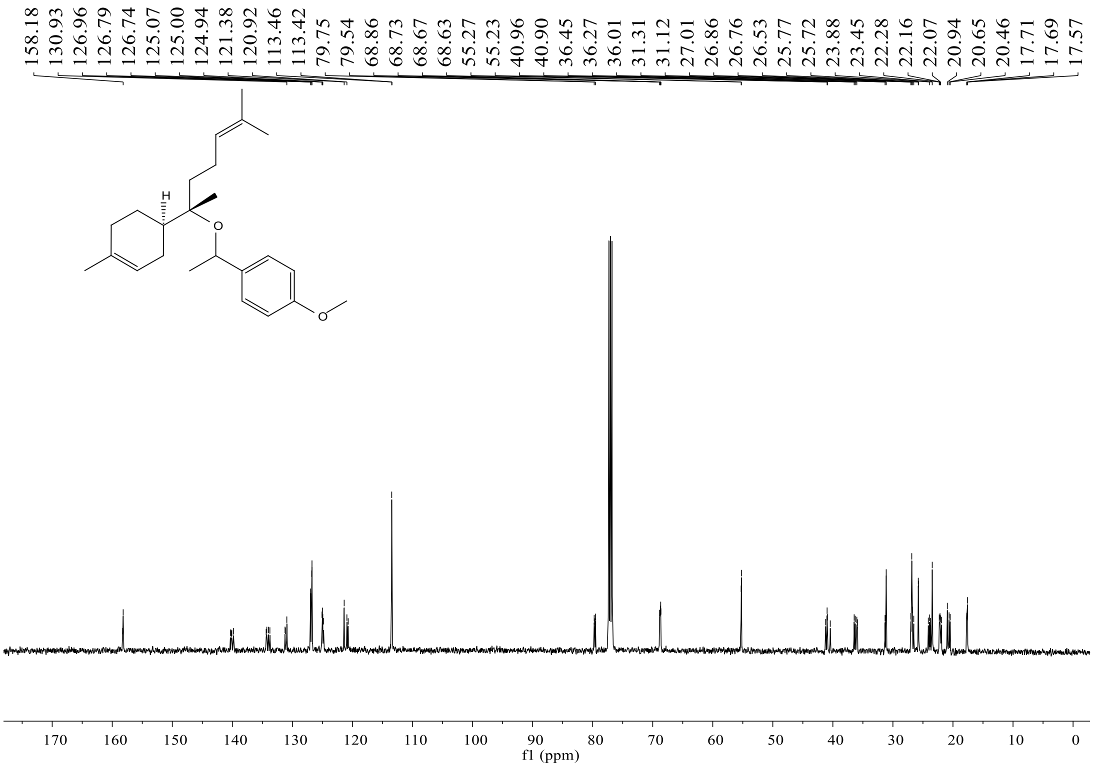

^{13}C NMR spectrum in $CDCl_3$.

108d

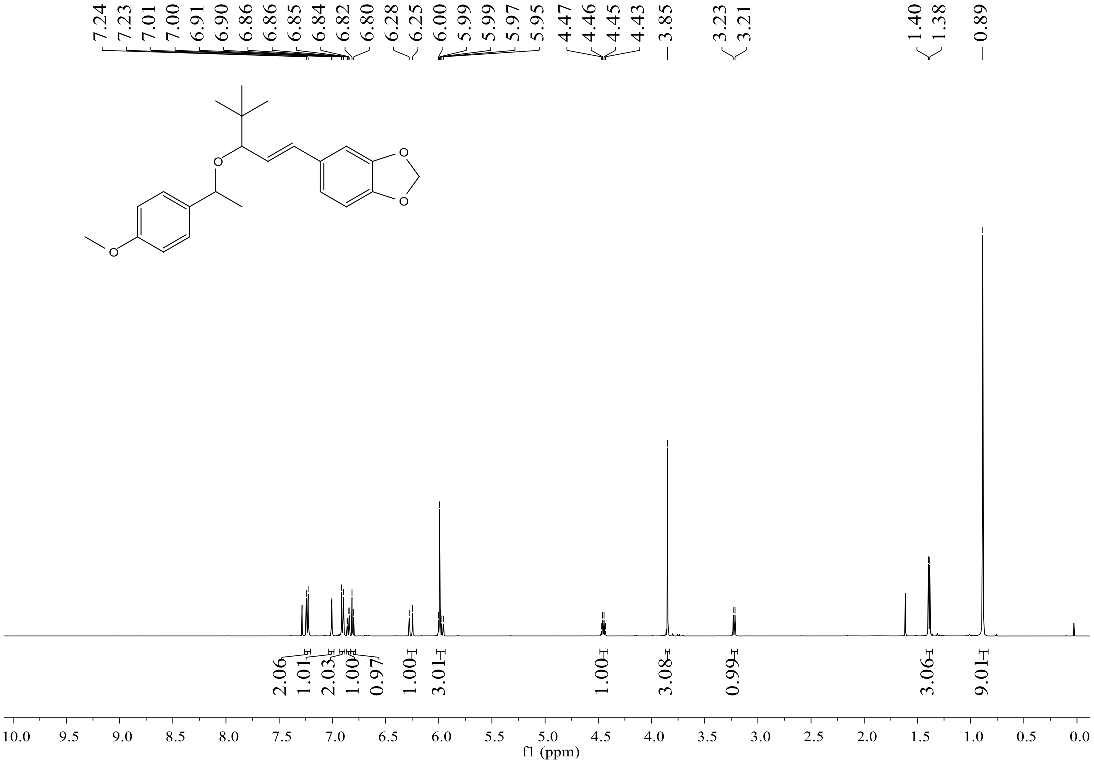

^{1}H NMR spectrum in $CDCl_3$.

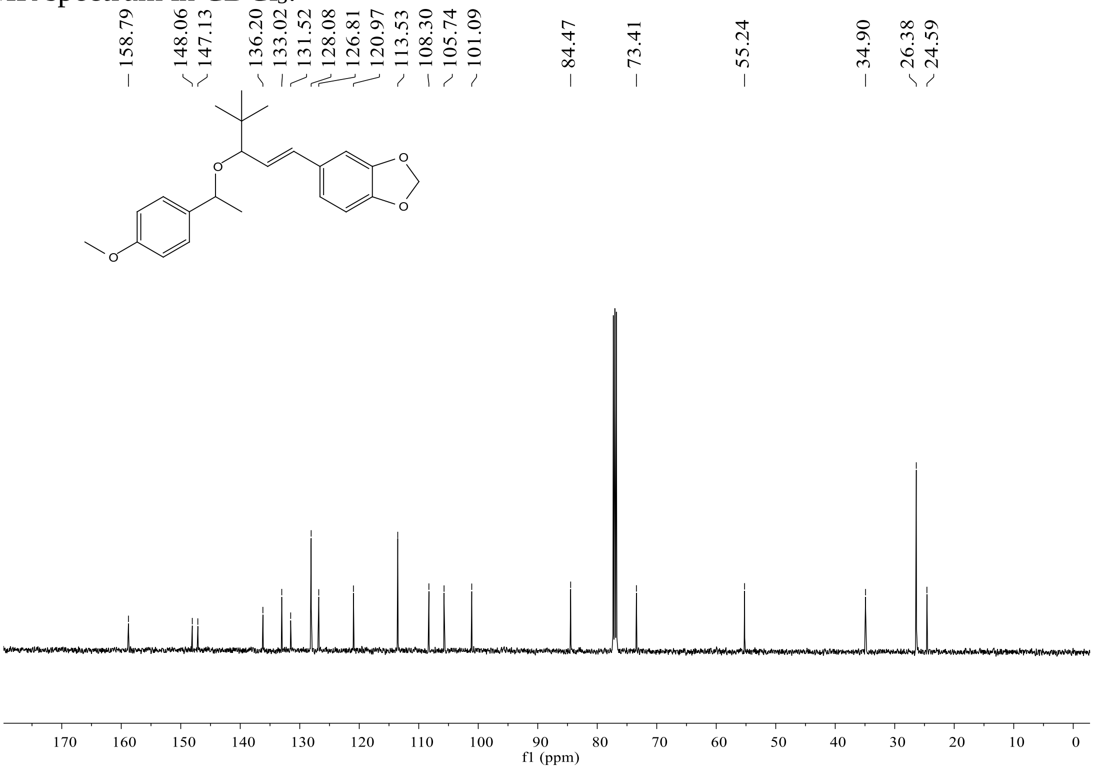

^{13}C NMR spectrum in $CDCl_3$.

109d

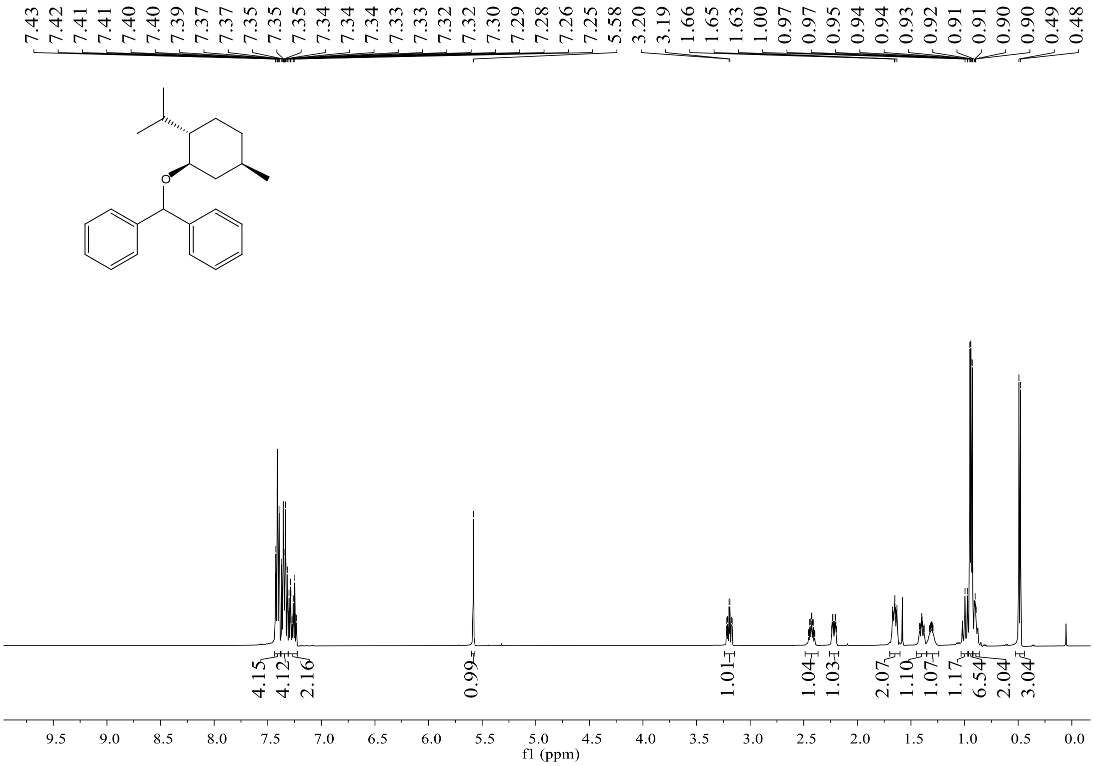

^{1}H NMR spectrum in $CDCl_3$.

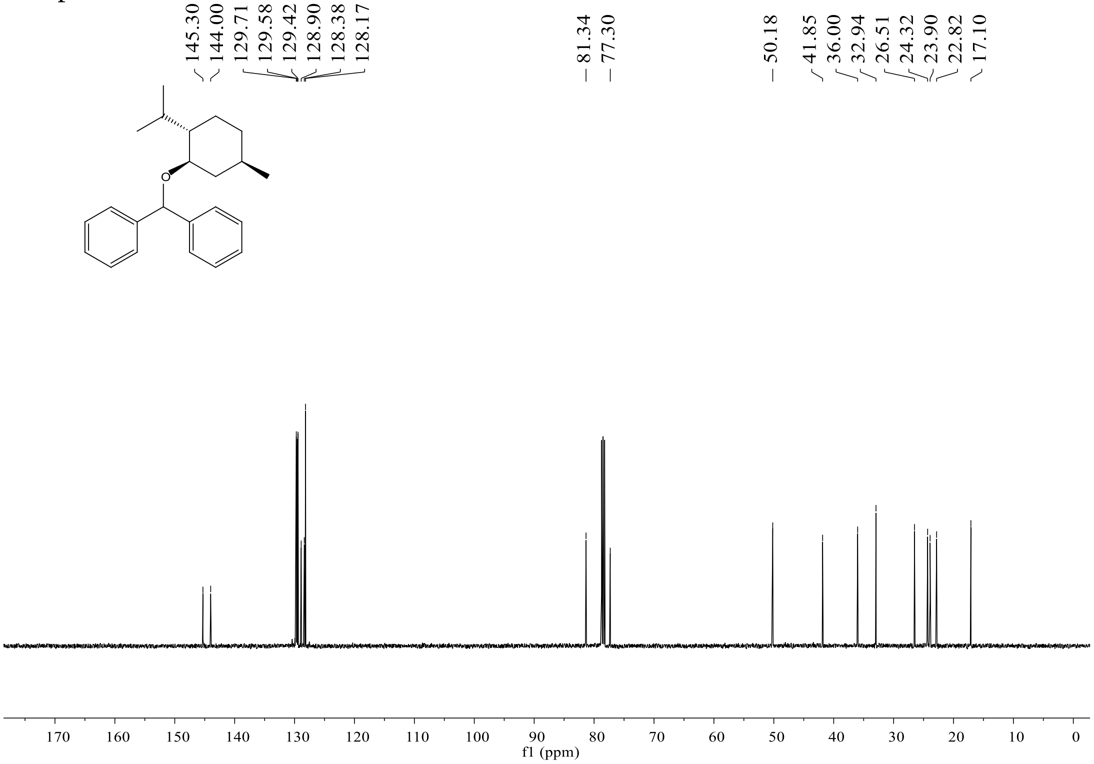

^{13}C NMR spectrum in $CDCl_3$.

110d

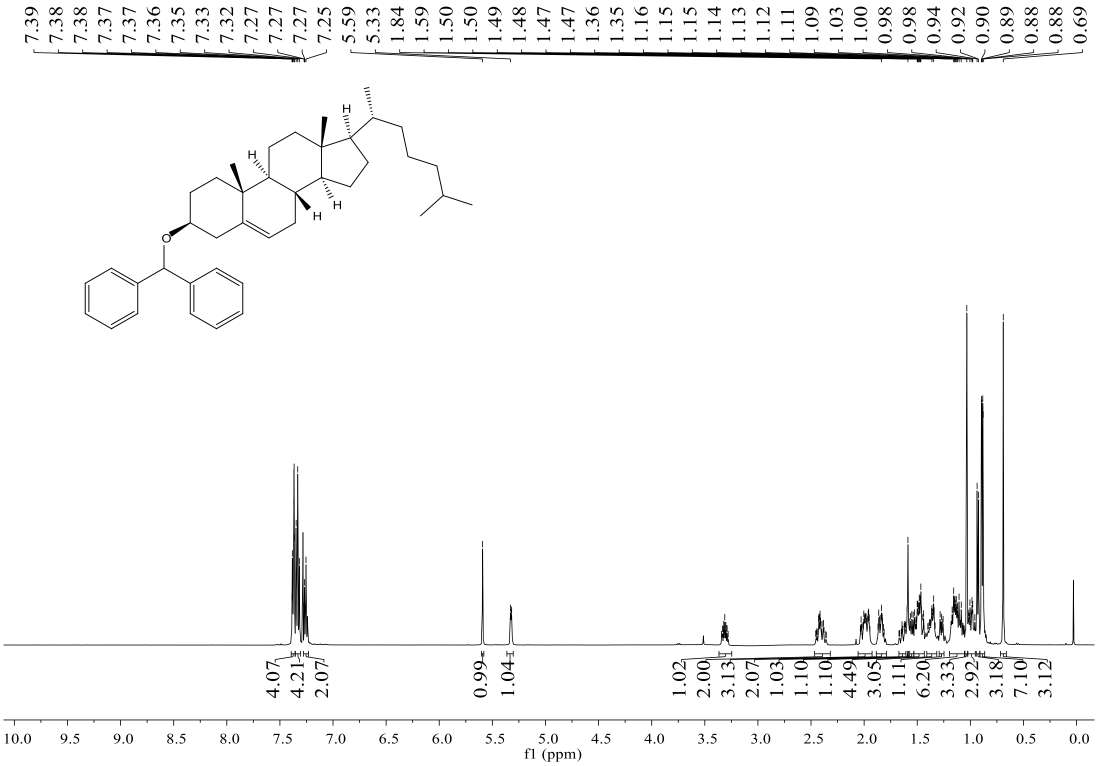

^{1}H NMR spectrum in $CDCl_3$.

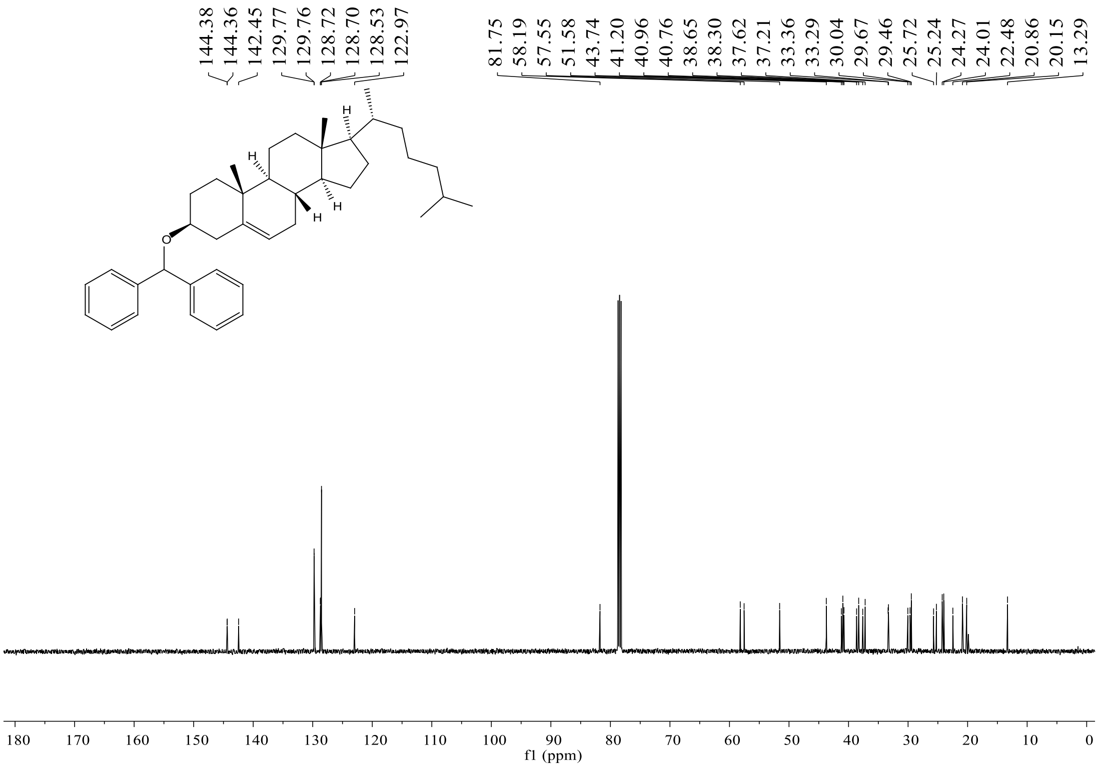

^{13}C NMR spectrum in $CDCl_3$.

111d

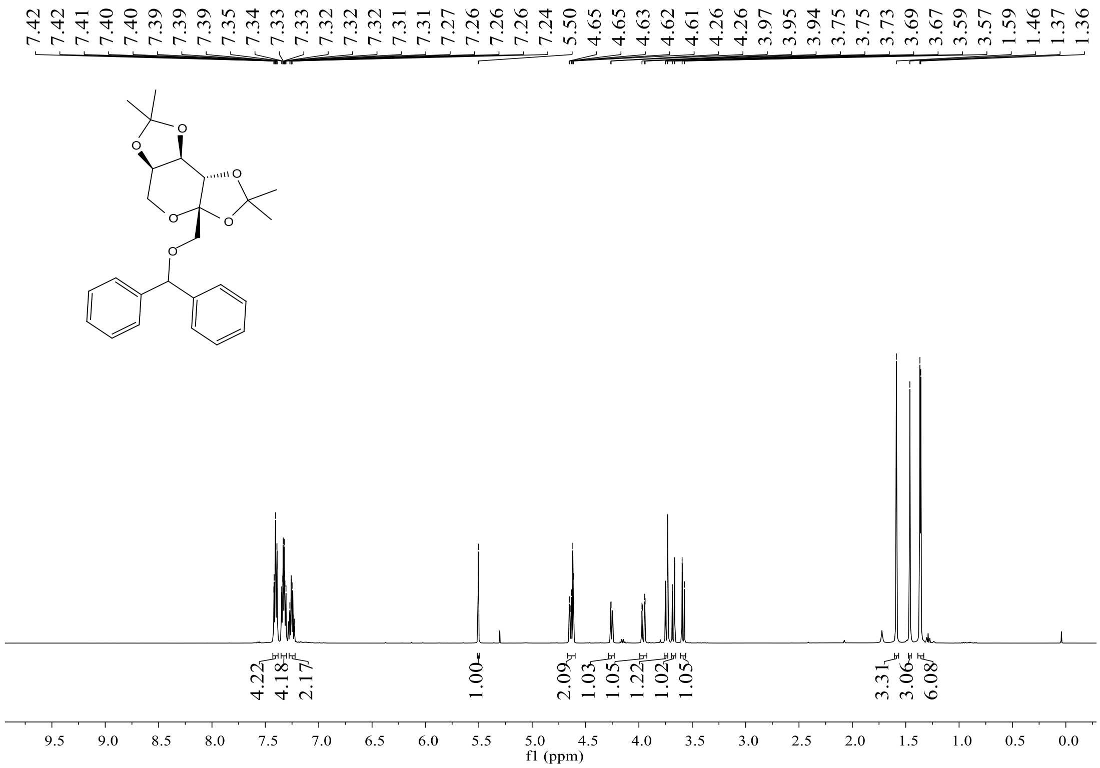

^{1}H NMR spectrum in $CDCl_3$.

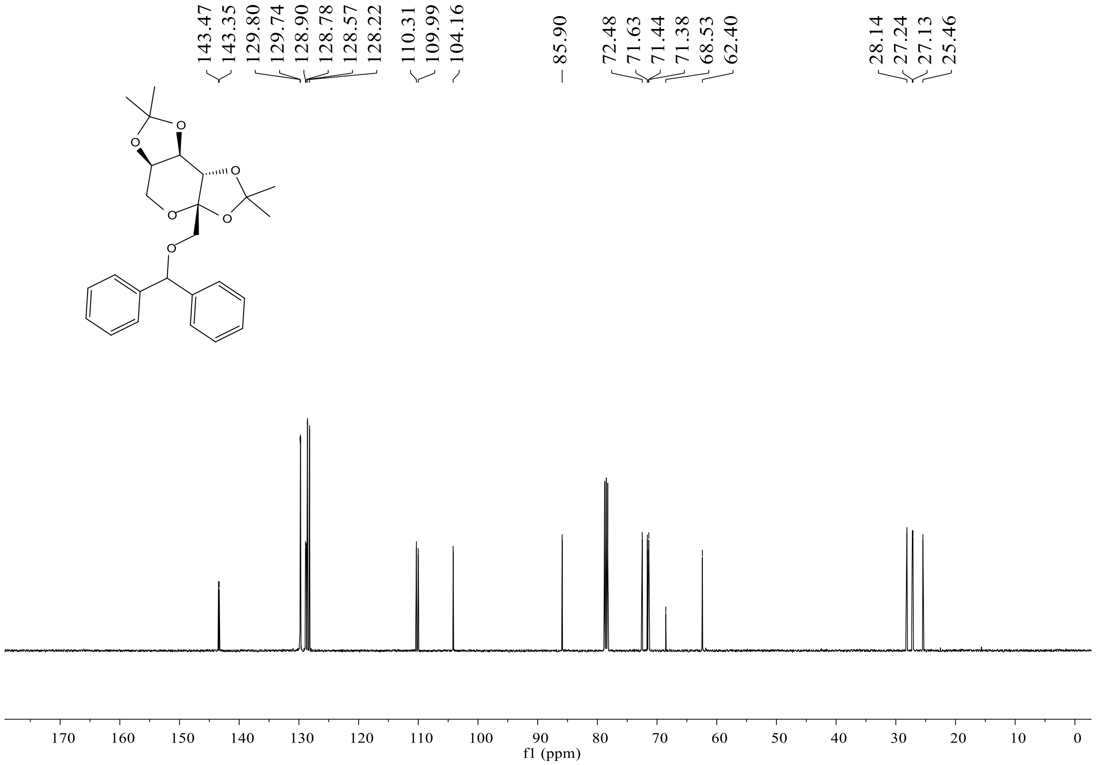

^{13}C NMR spectrum in $CDCl_3$.

112d

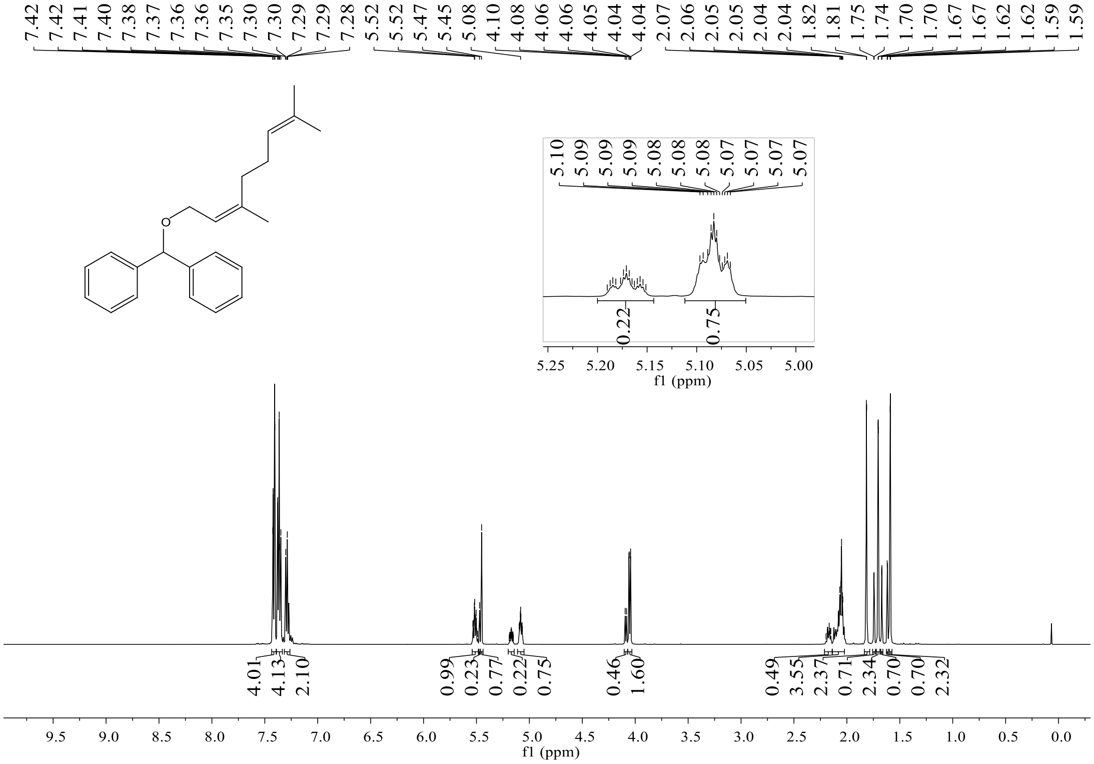

^{1}H NMR spectrum in $CDCl_3$.

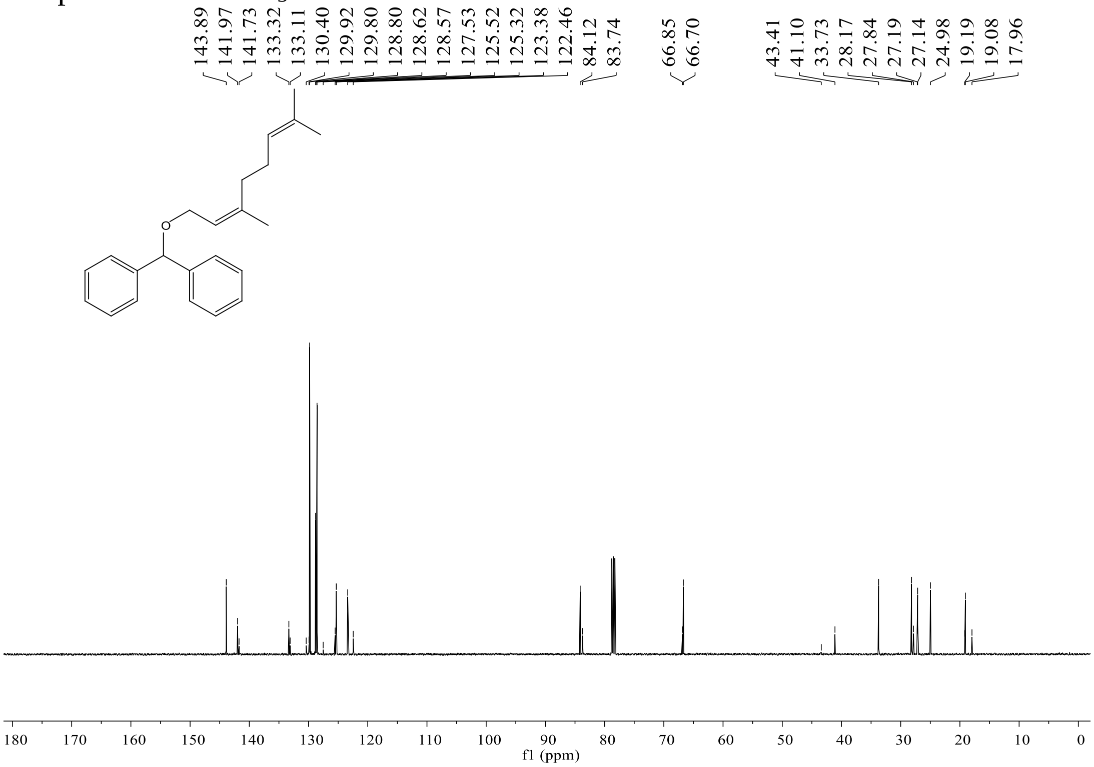

^{13}C NMR spectrum in $CDCl_3$.

113d

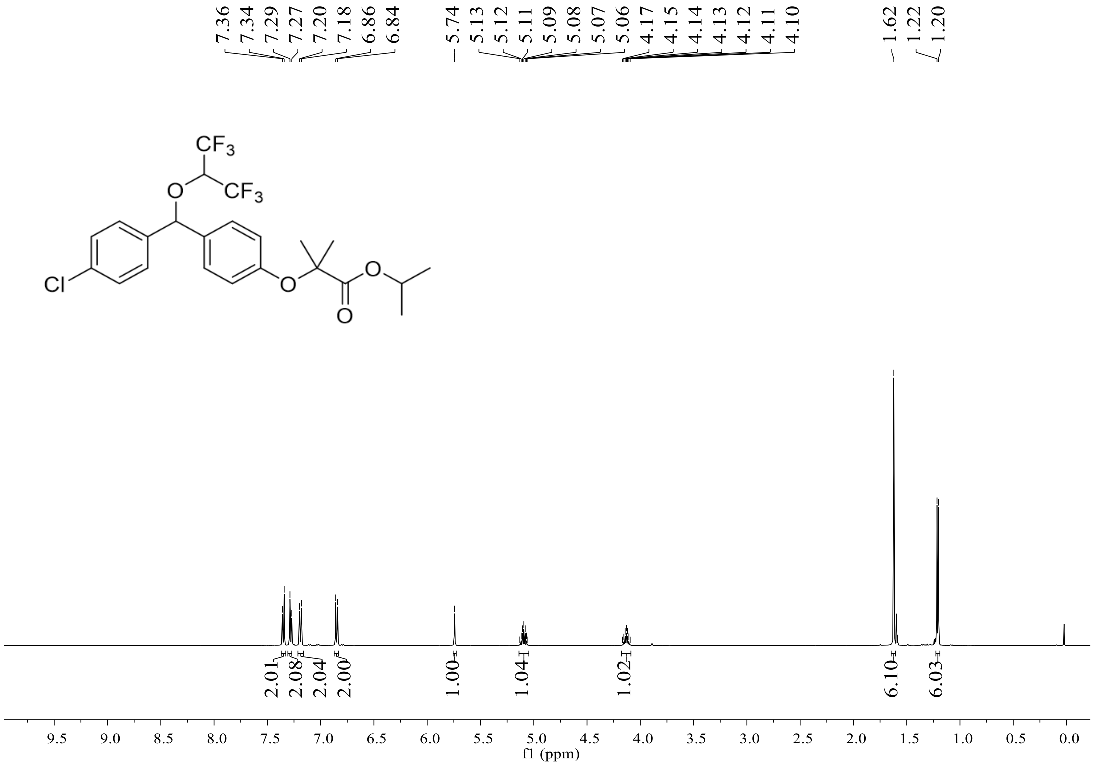

^{1}H NMR spectrum in $CDCl_3$.

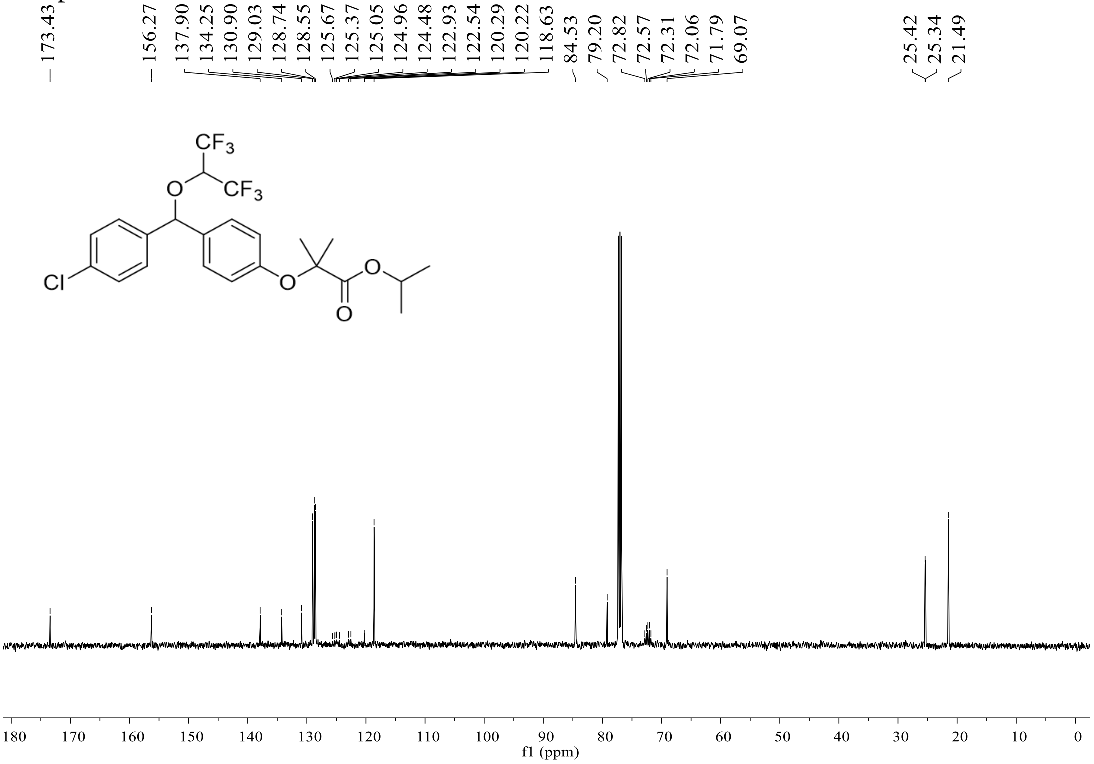

^{13}C NMR spectrum in $CDCl_3$.

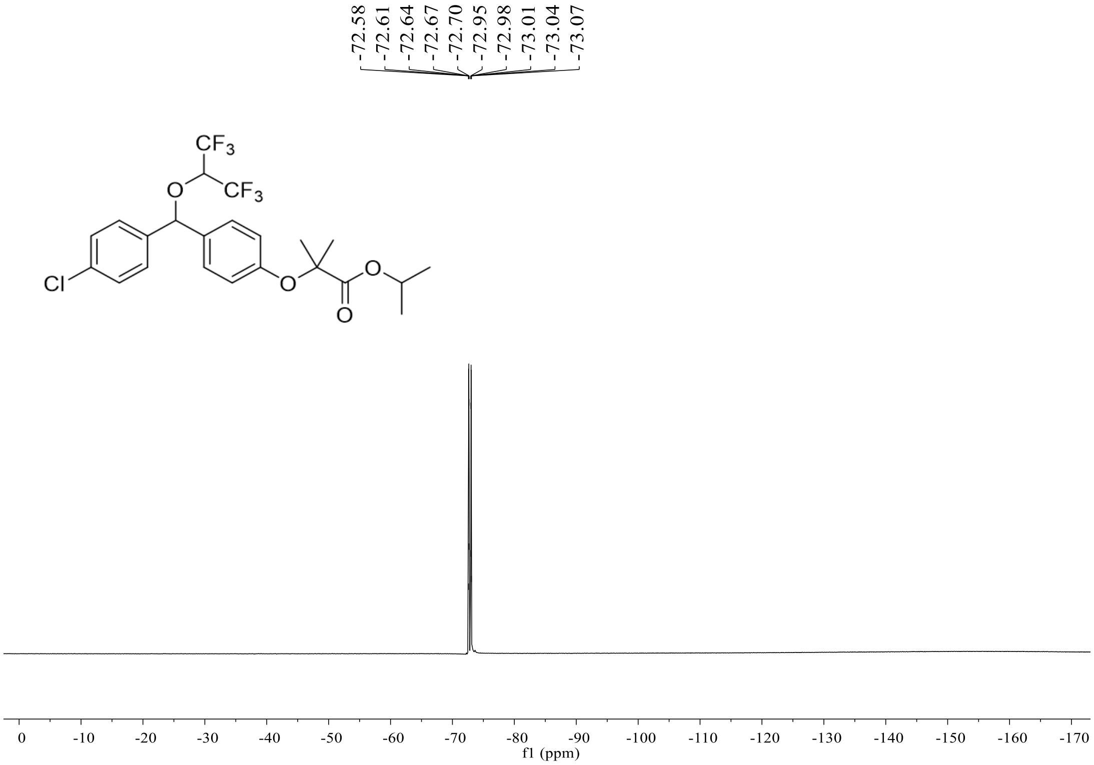

^{19}F NMR spectrum in $CDCl_3$.

114d

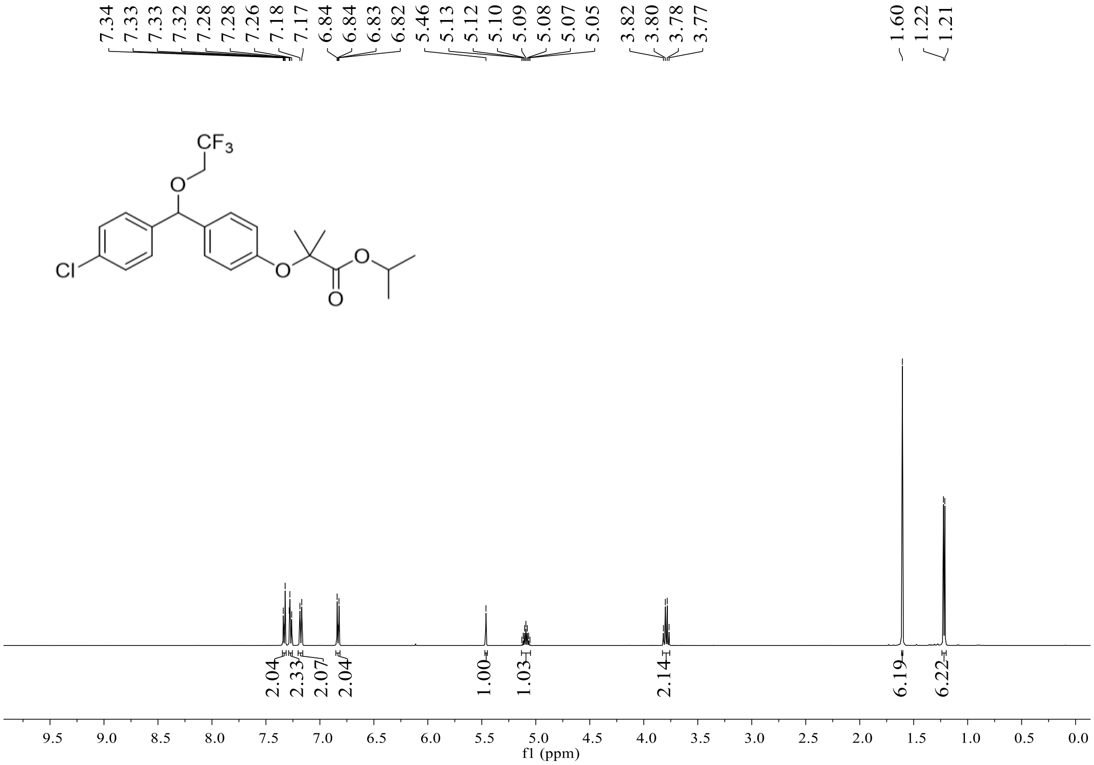

^{1}H NMR spectrum in $CDCl_3$.

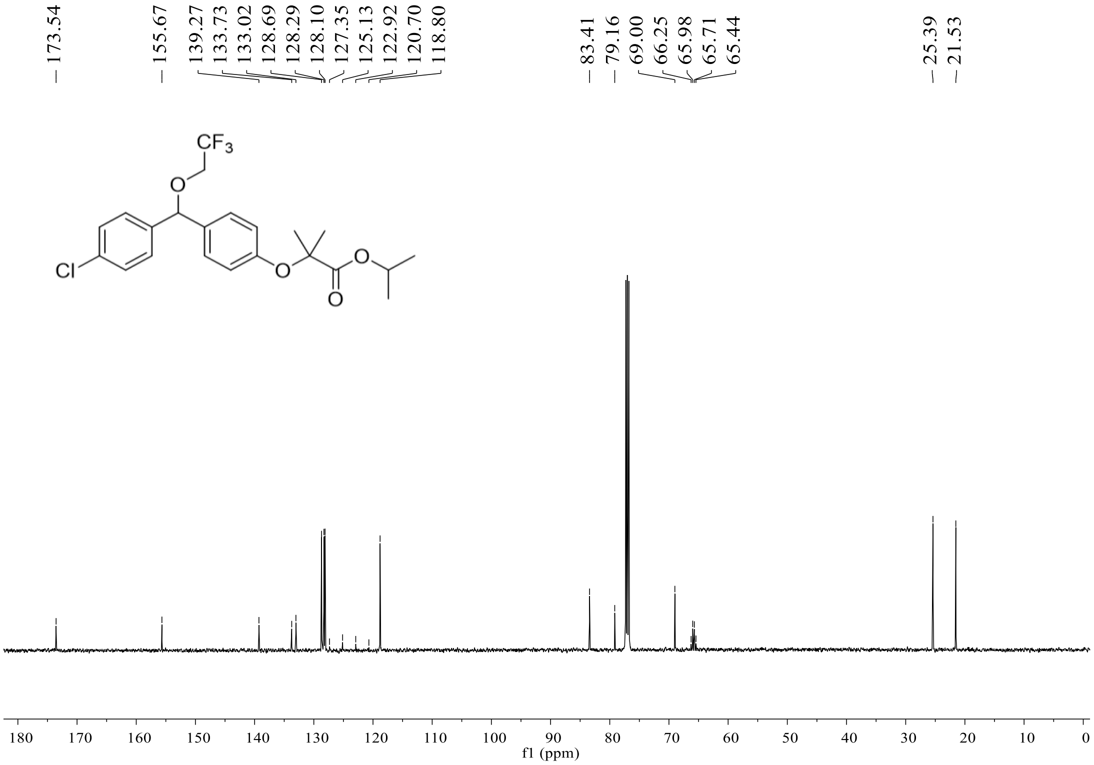

^{13}C NMR spectrum in $CDCl_3$.

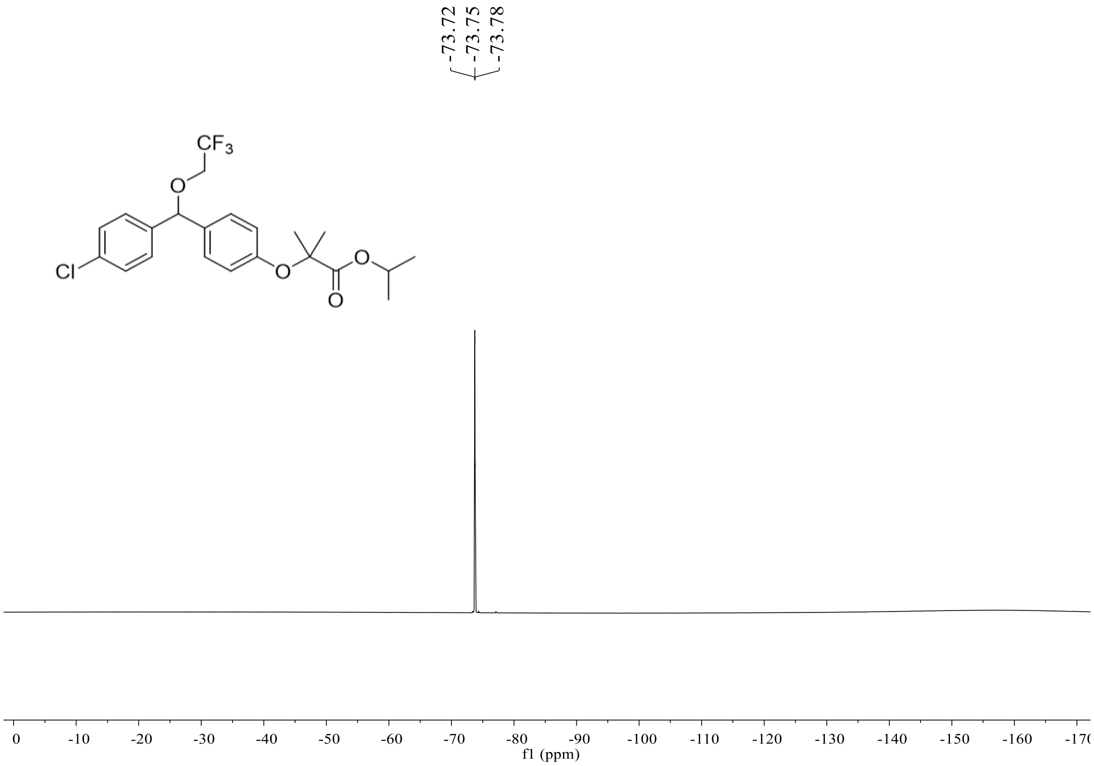

^{19}F NMR spectrum in $CDCl_3$.

115d

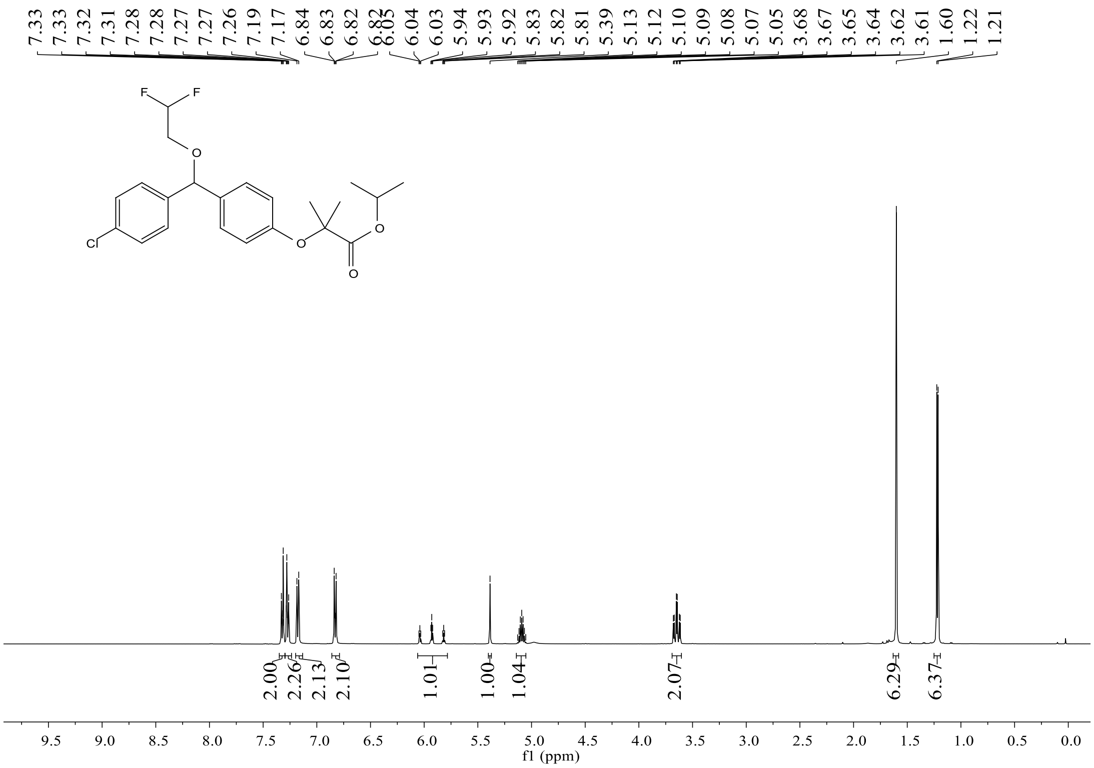

^{1}H NMR spectrum in $CDCl_3$.

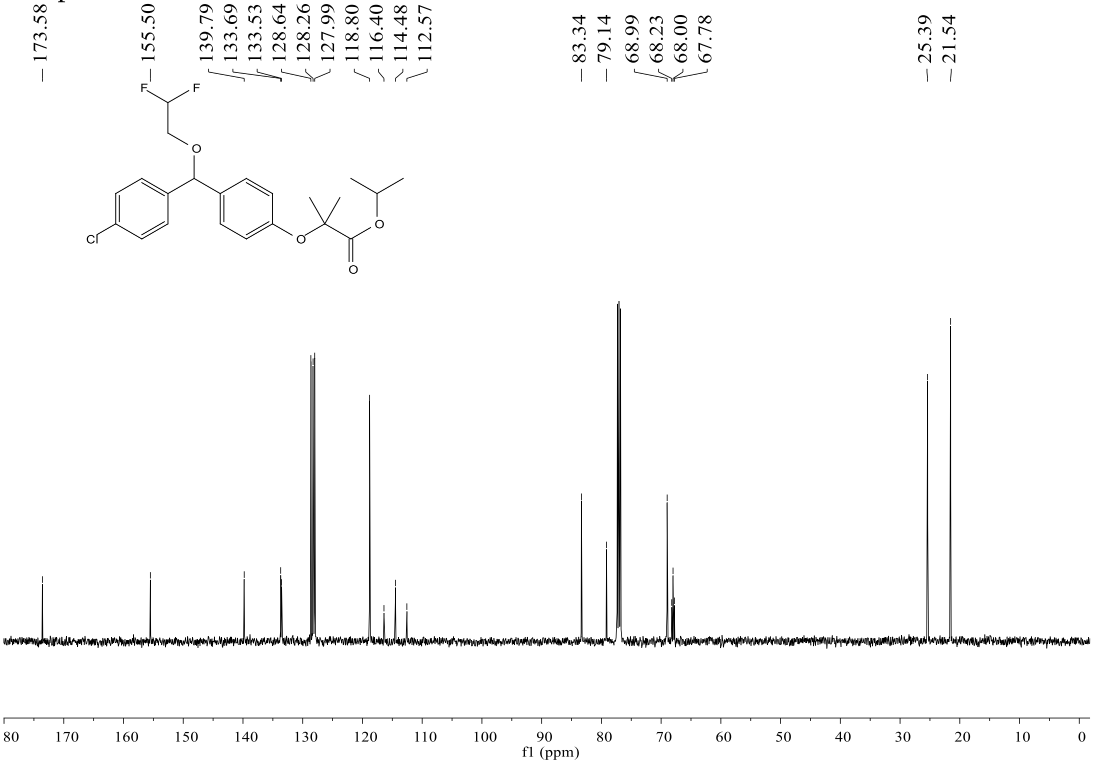

^{13}C NMR spectrum in $CDCl_3$.

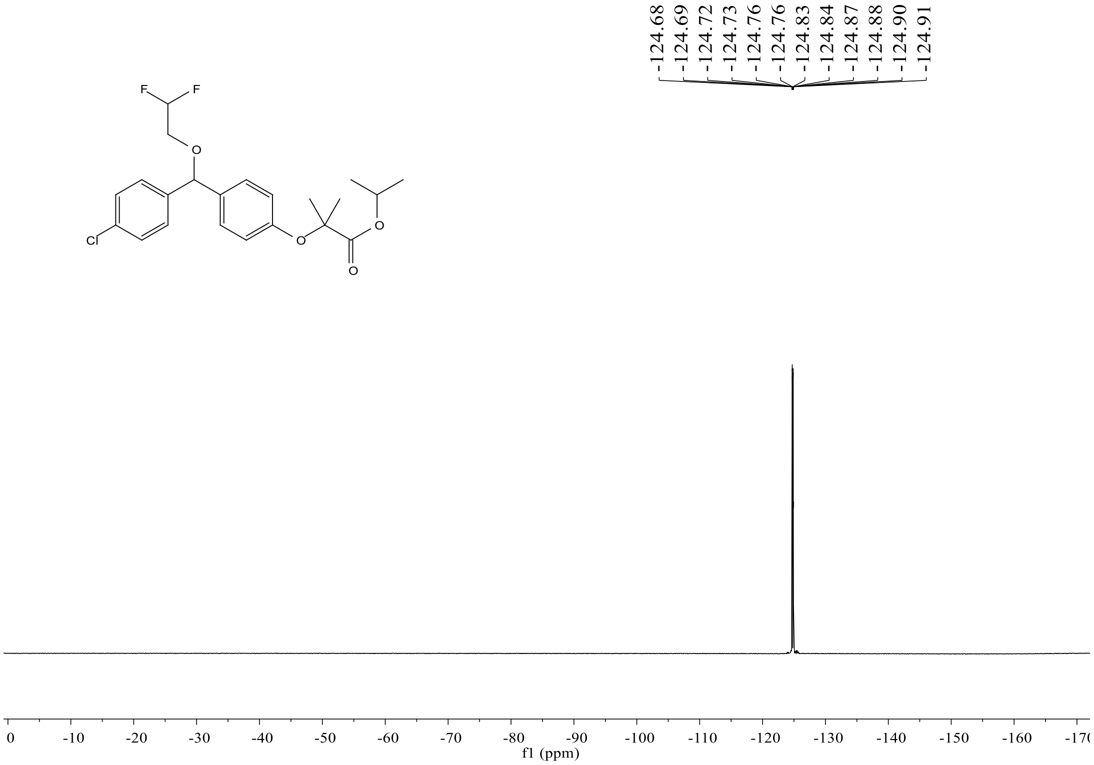

^{19}F NMR spectrum in $CDCl_3$.

116d

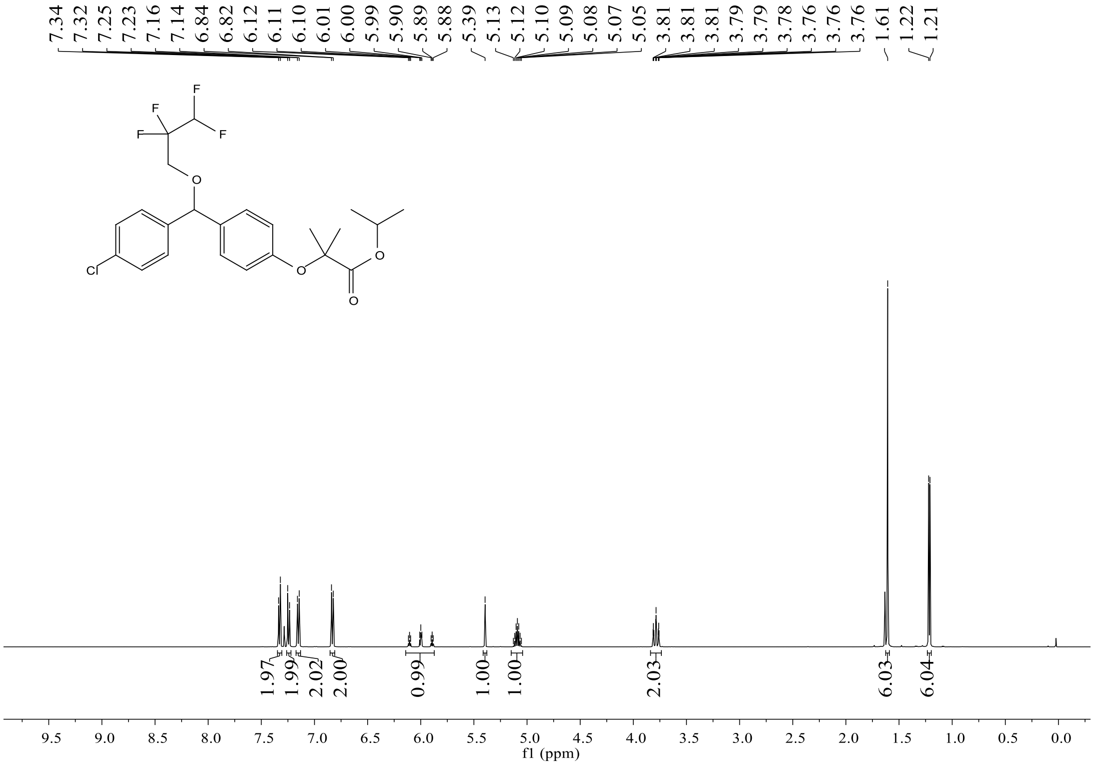

^{1}H NMR spectrum in $CDCl_3$.

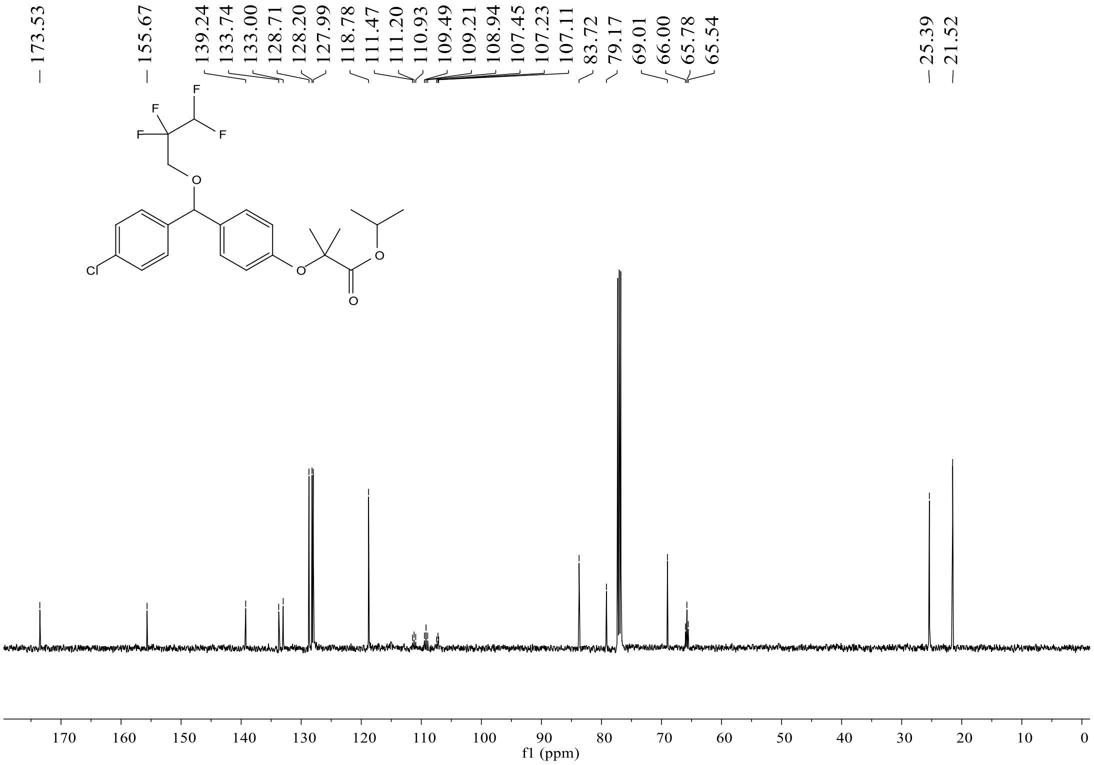

^{13}C NMR spectrum in $CDCl_3$.

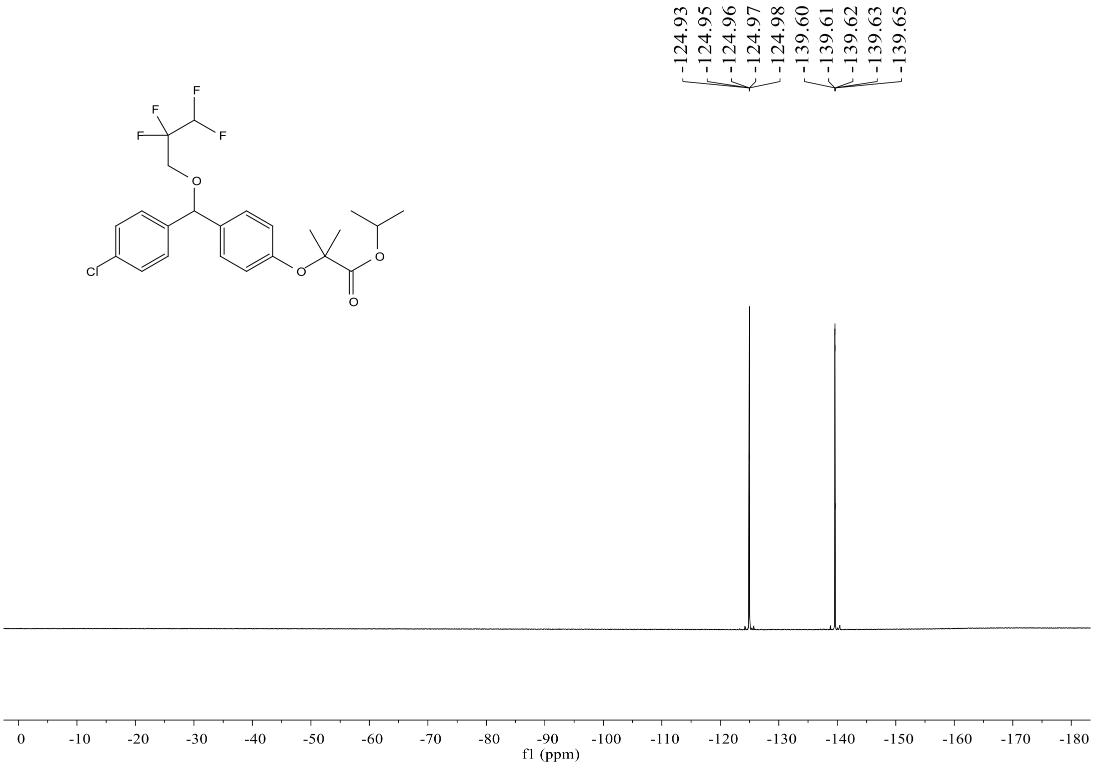

^{19}F NMR spectrum in $CDCl_3$.

117d

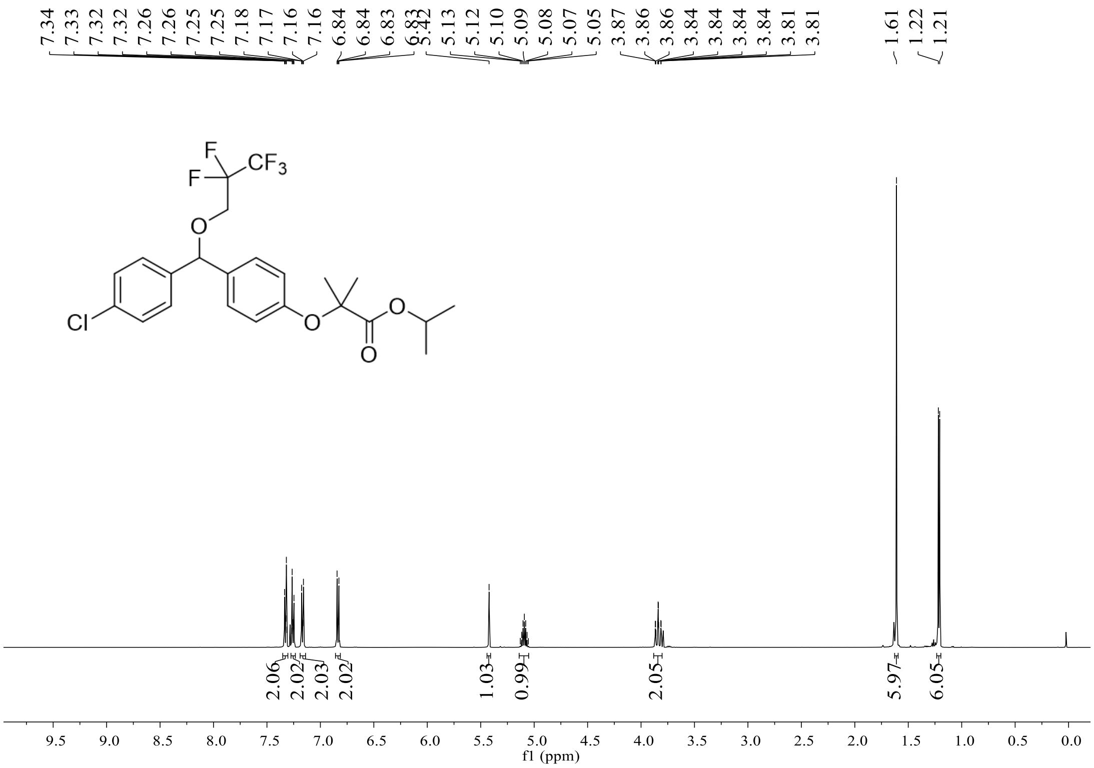

^{1}H NMR spectrum in $CDCl_3$.

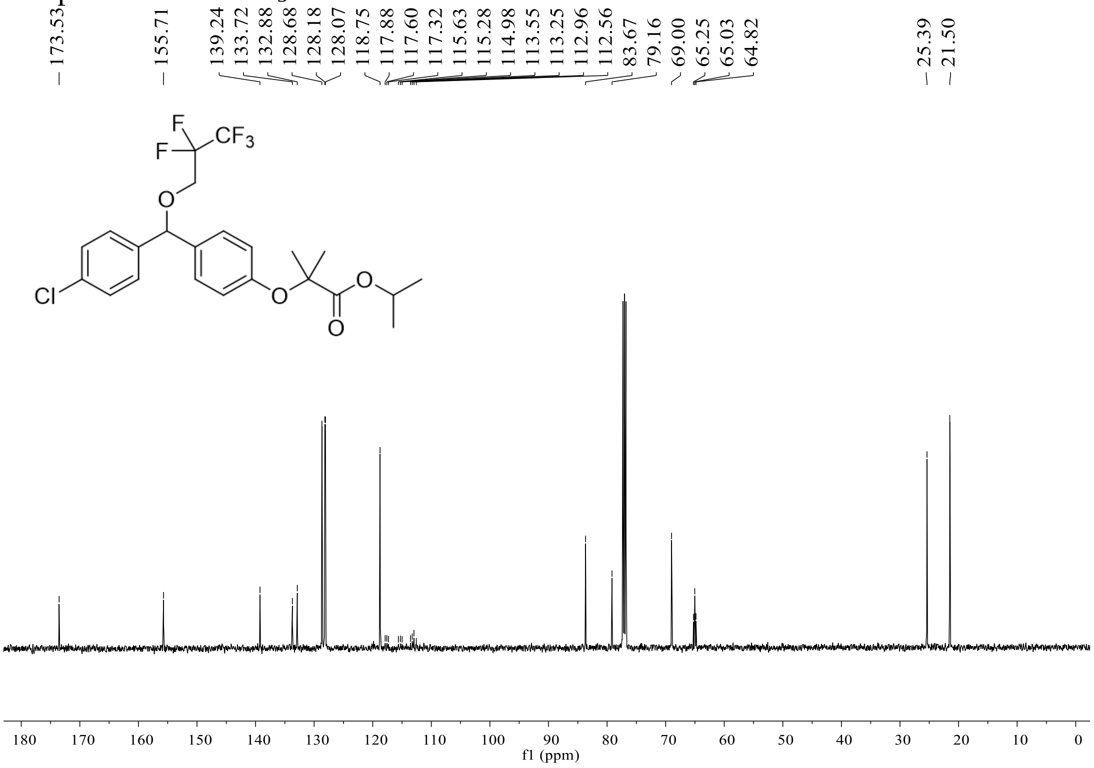

^{13}C NMR spectrum in $CDCl_3$.

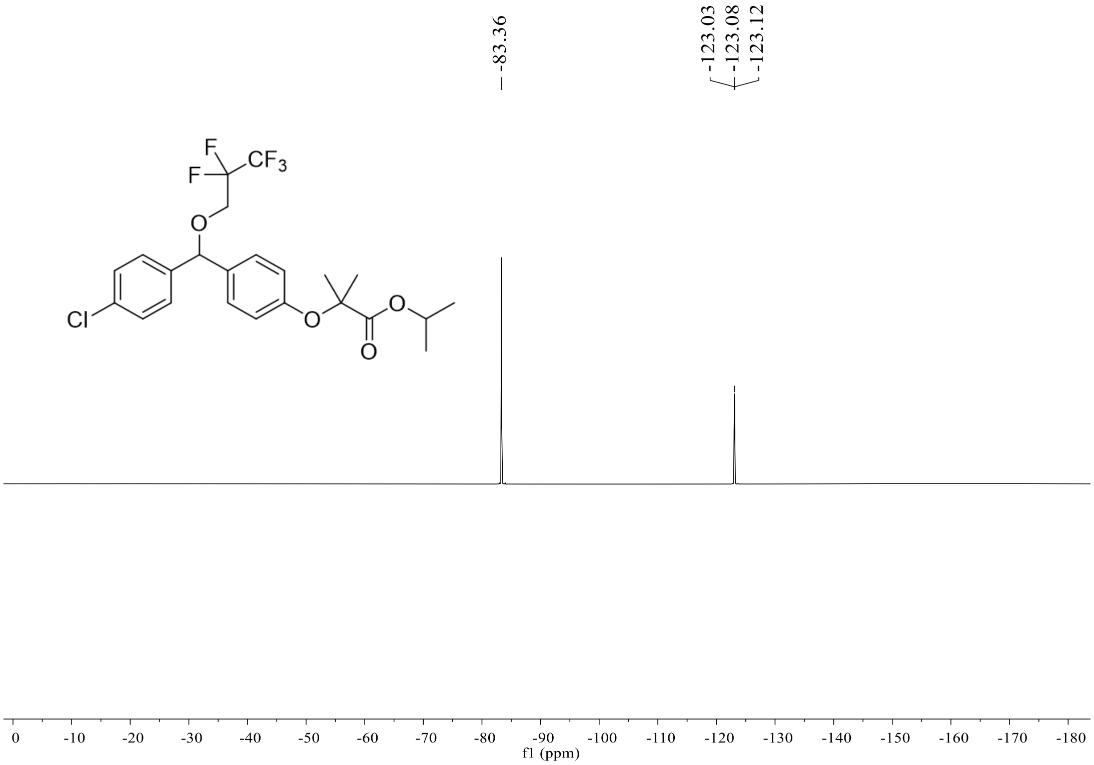

^{19}F NMR spectrum in $CDCl_3$.

118d

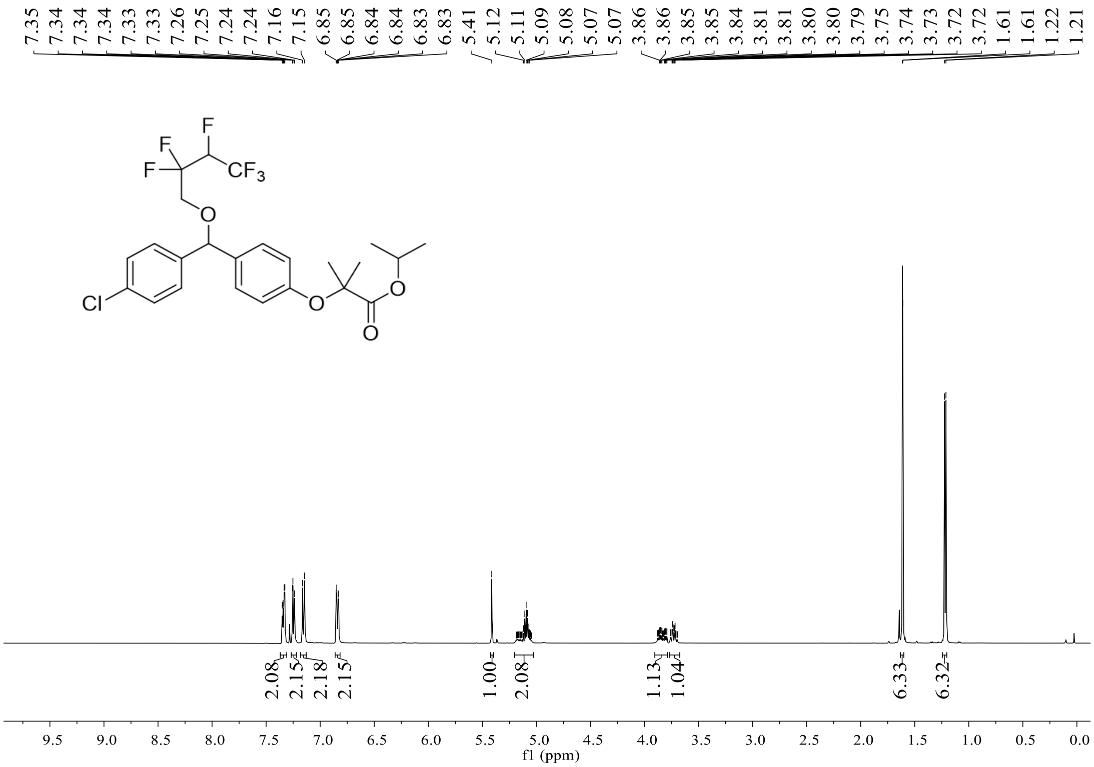

^{1}H NMR spectrum in $CDCl_3$.

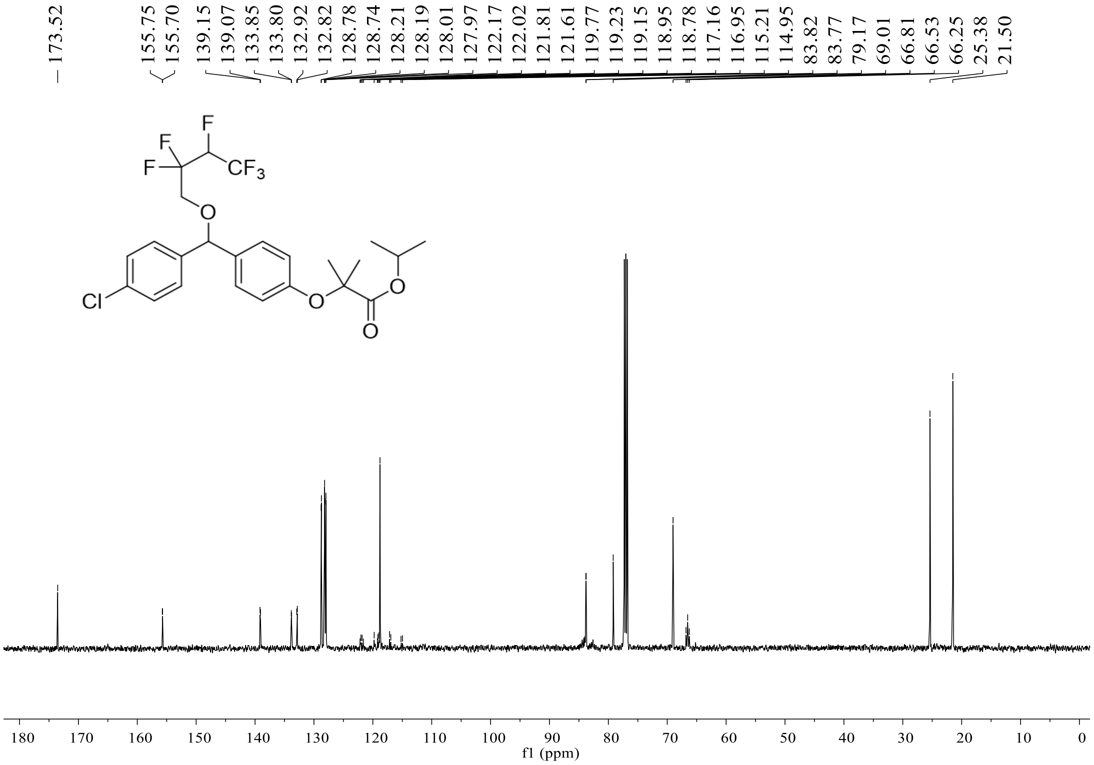

^{13}C NMR spectrum in $CDCl_3$.

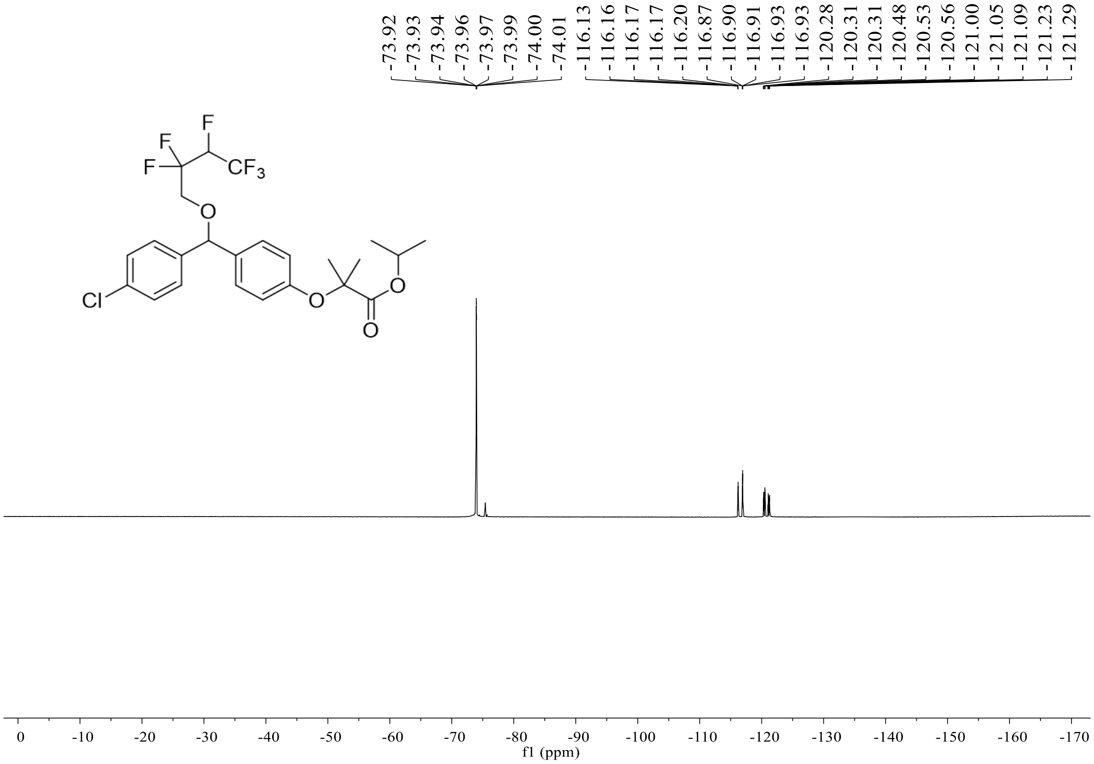

^{19}F NMR spectrum in $CDCl_3$.

119d

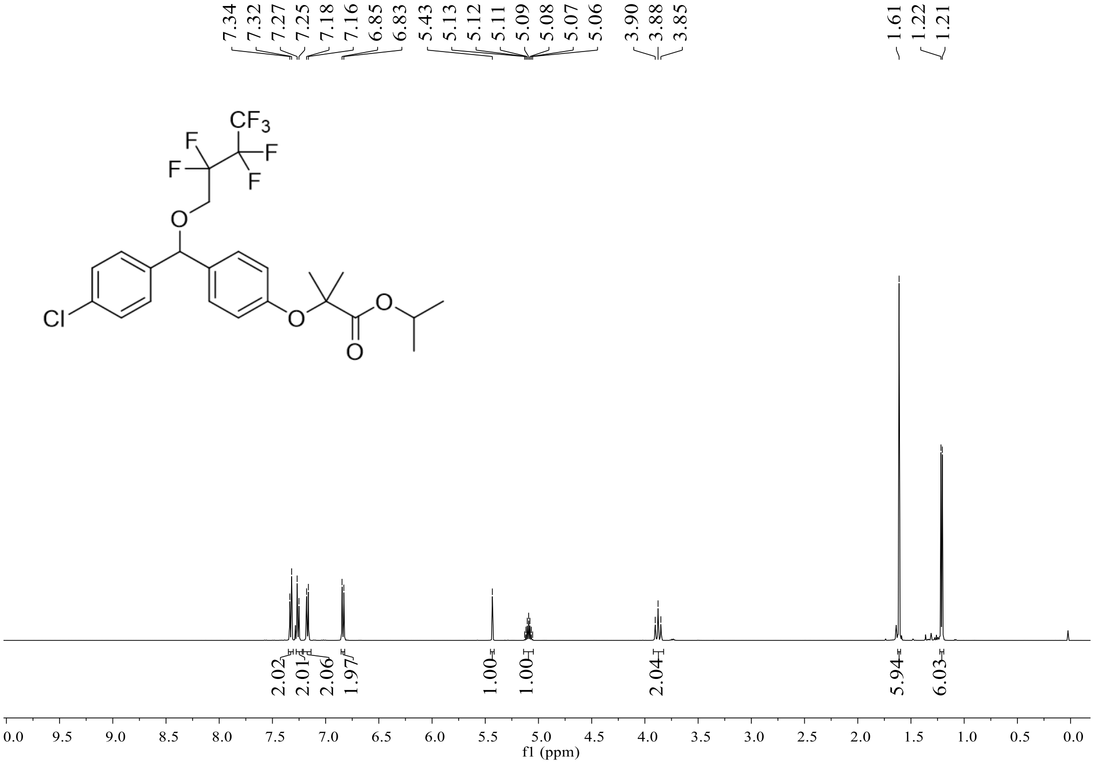

^{1}H NMR spectrum in $CDCl_3$.

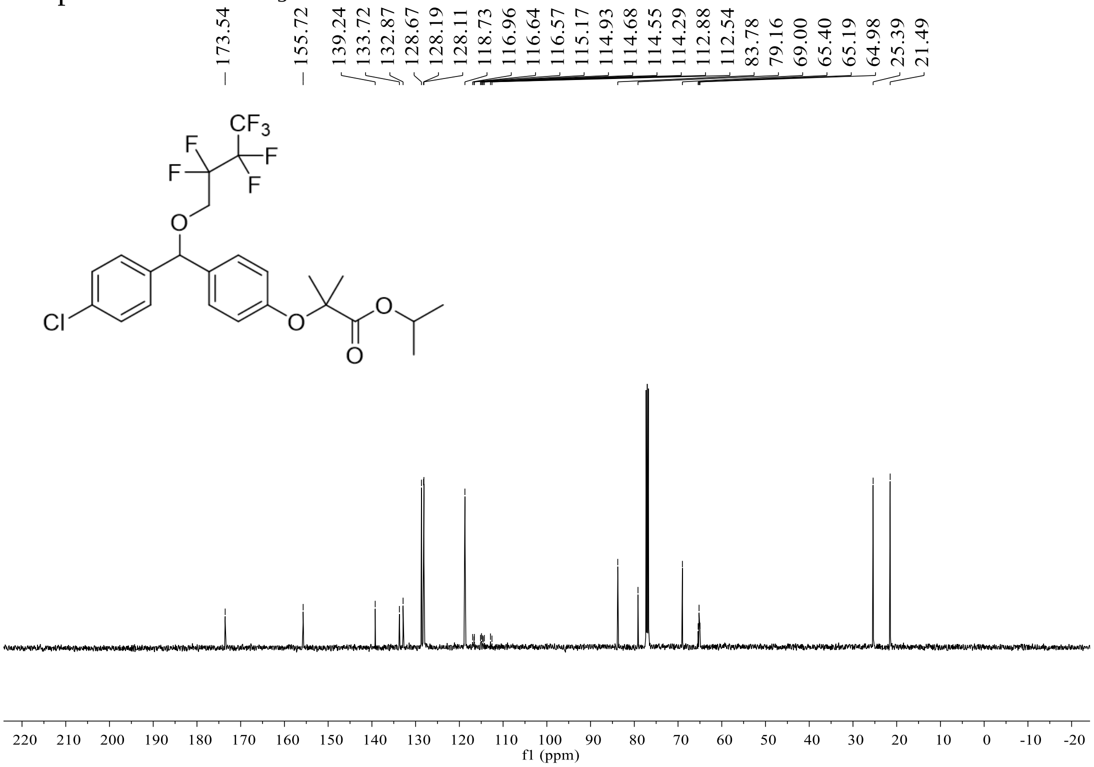

^{13}C NMR spectrum in $CDCl_3$.

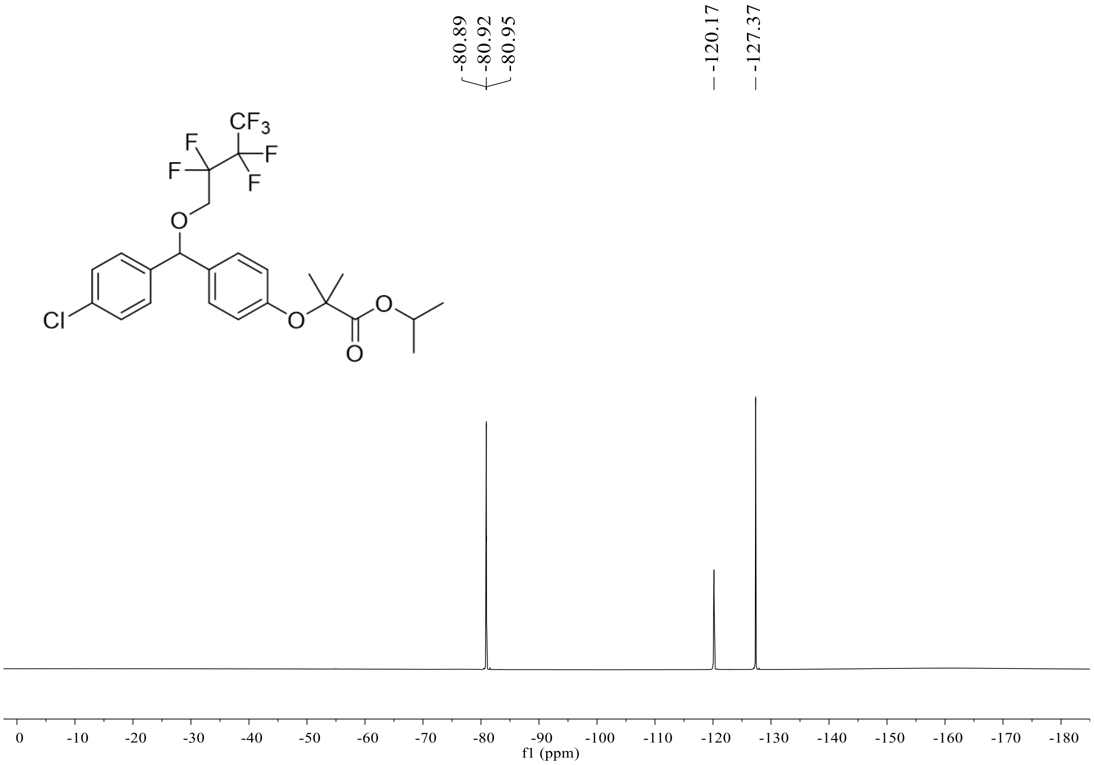

^{19}F NMR spectrum in $CDCl_3$.

120d

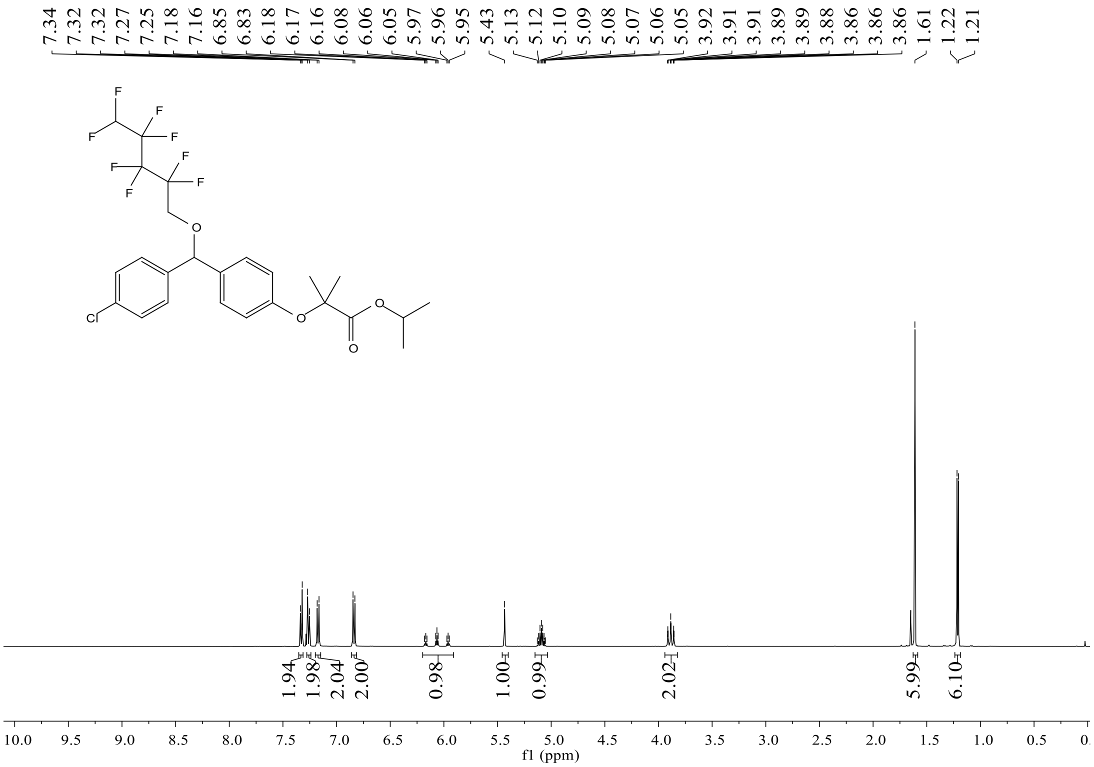

^{1}H NMR spectrum in $CDCl_3$.

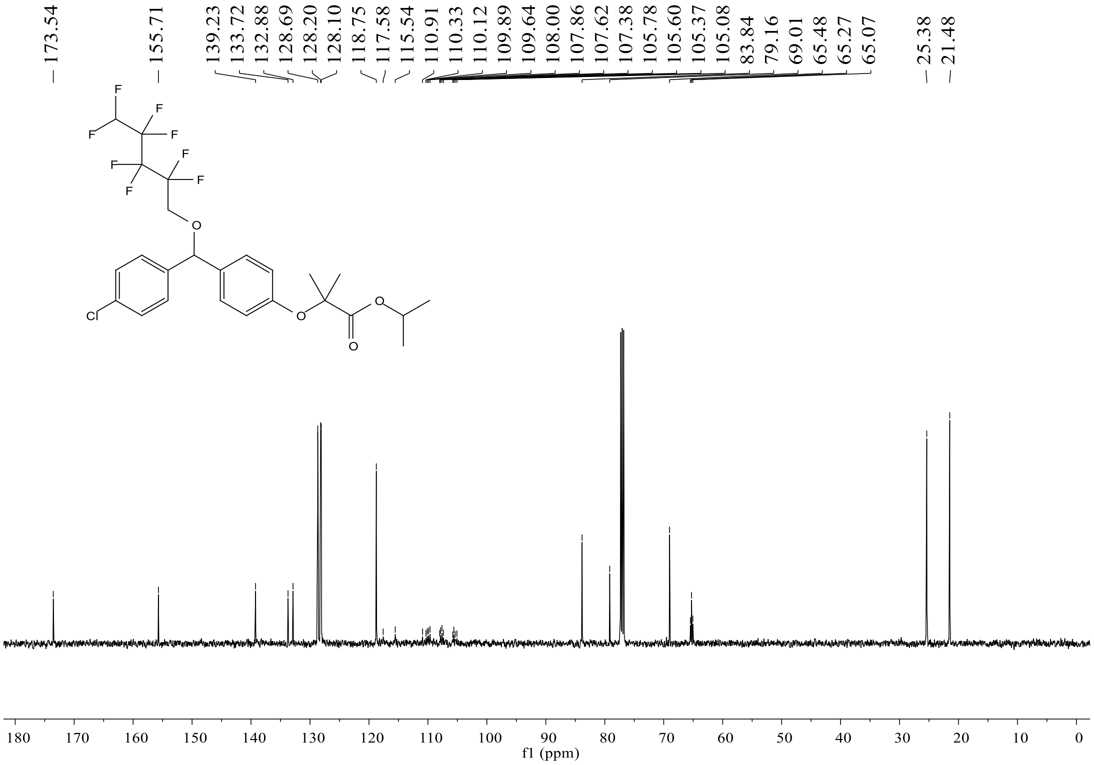

^{13}C NMR spectrum in $CDCl_3$.

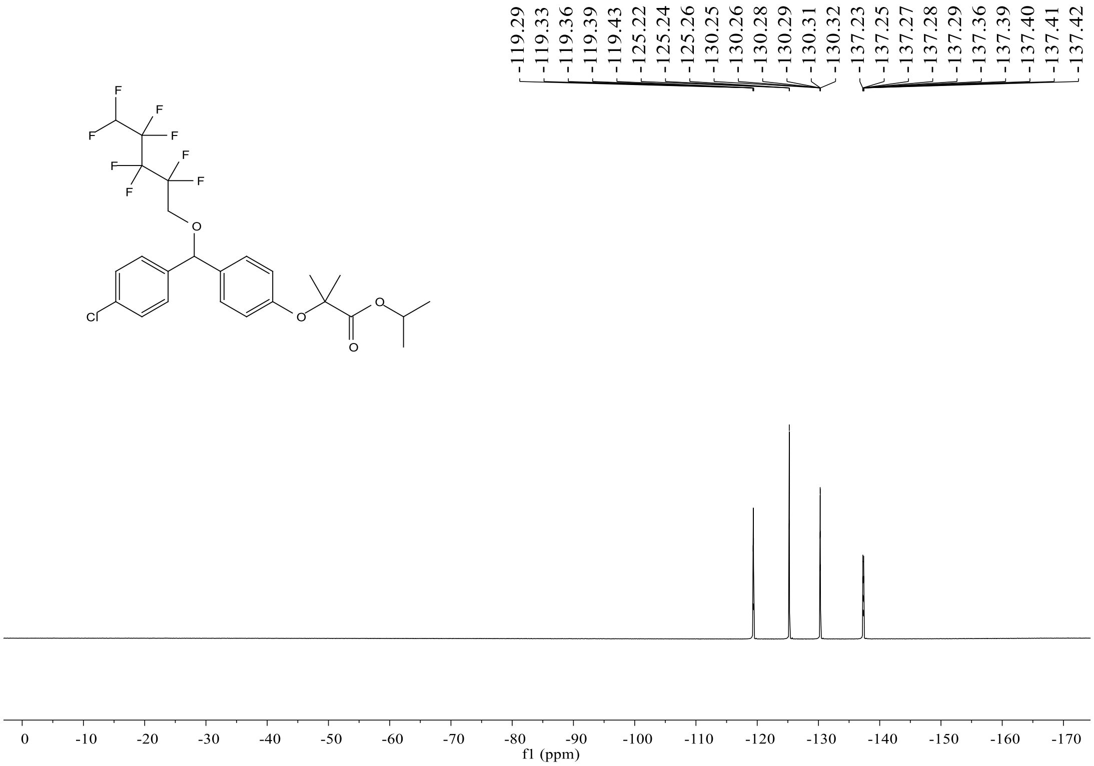

^{19}F NMR spectrum in $CDCl_3$.

121d

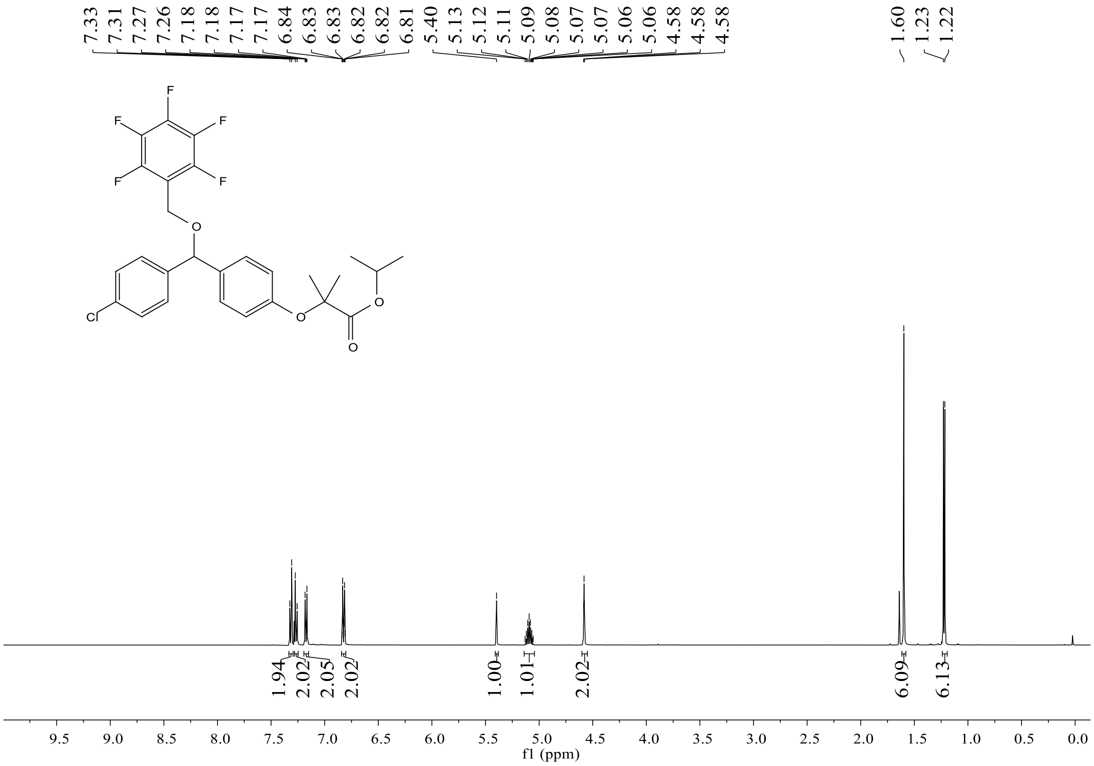

^{1}H NMR spectrum in $CDCl_3$.

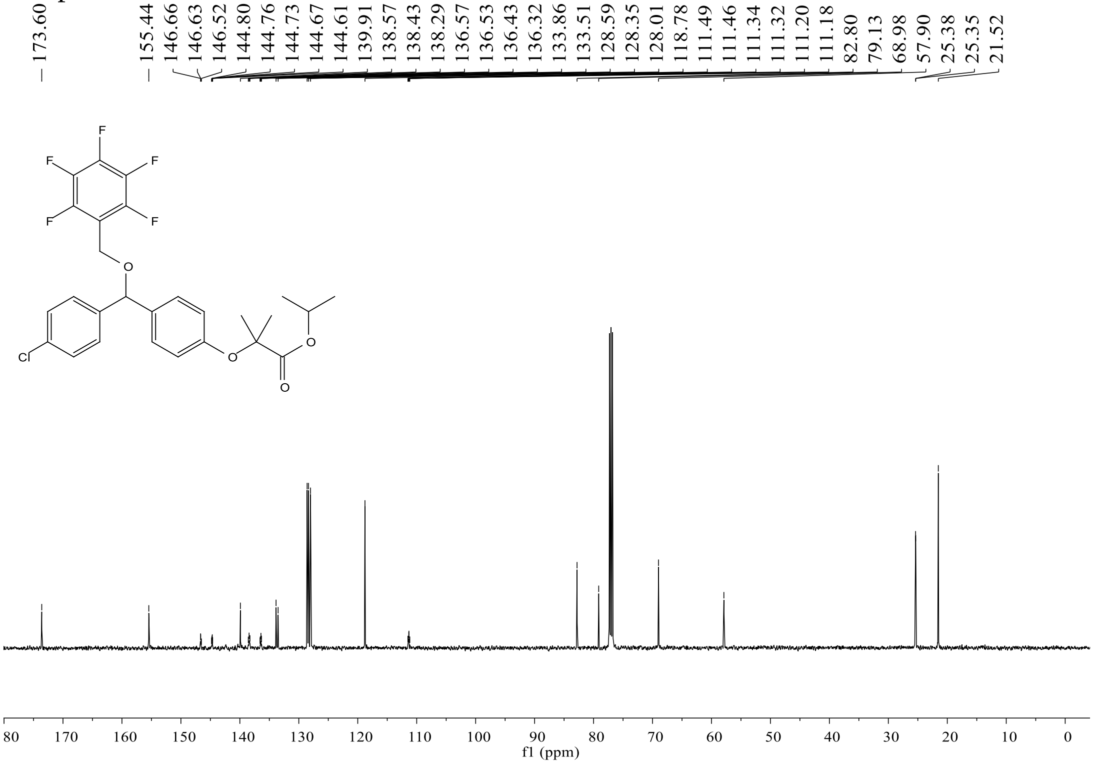

^{13}C NMR spectrum in $CDCl_3$.

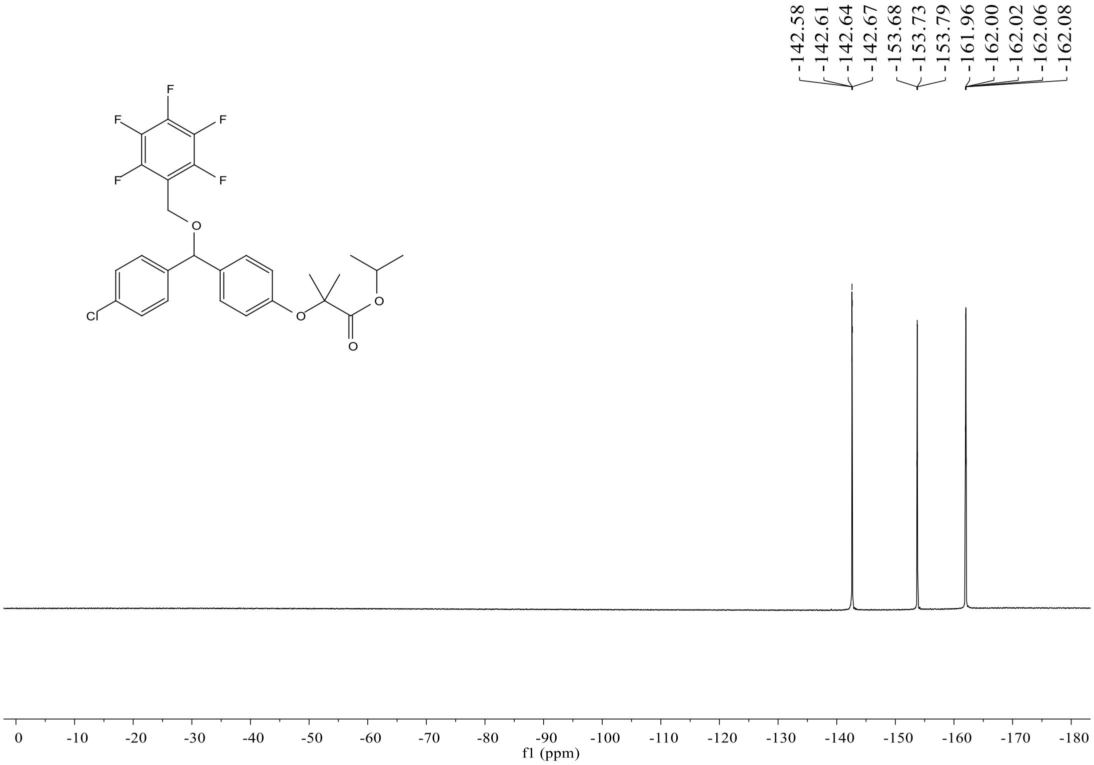

^{19}F NMR spectrum in $CDCl_3$.

122d

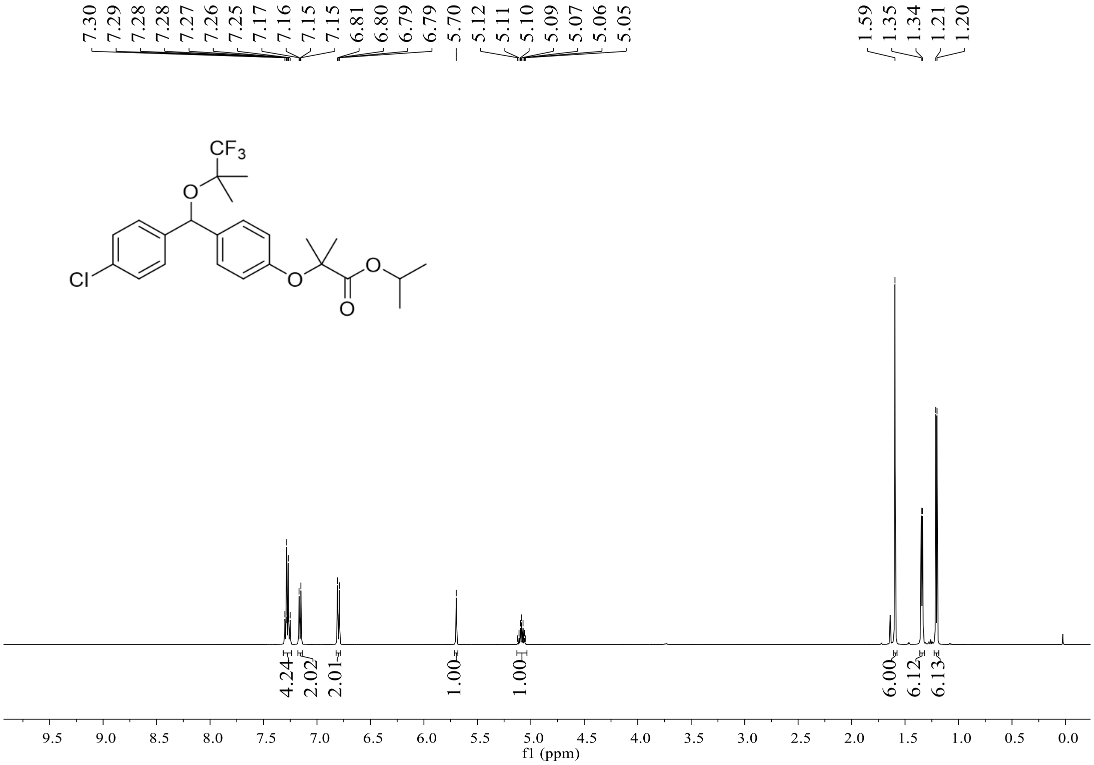

^{1}H NMR spectrum in $CDCl_3$.

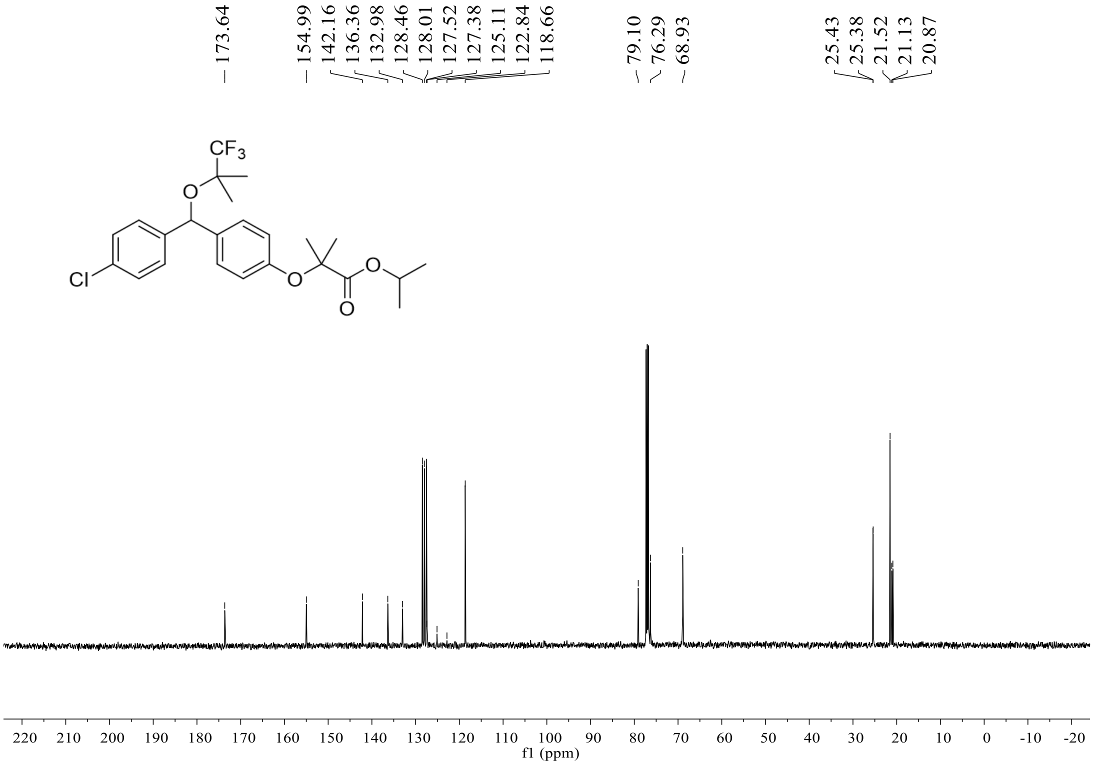

^{13}C NMR spectrum in $CDCl_3$.

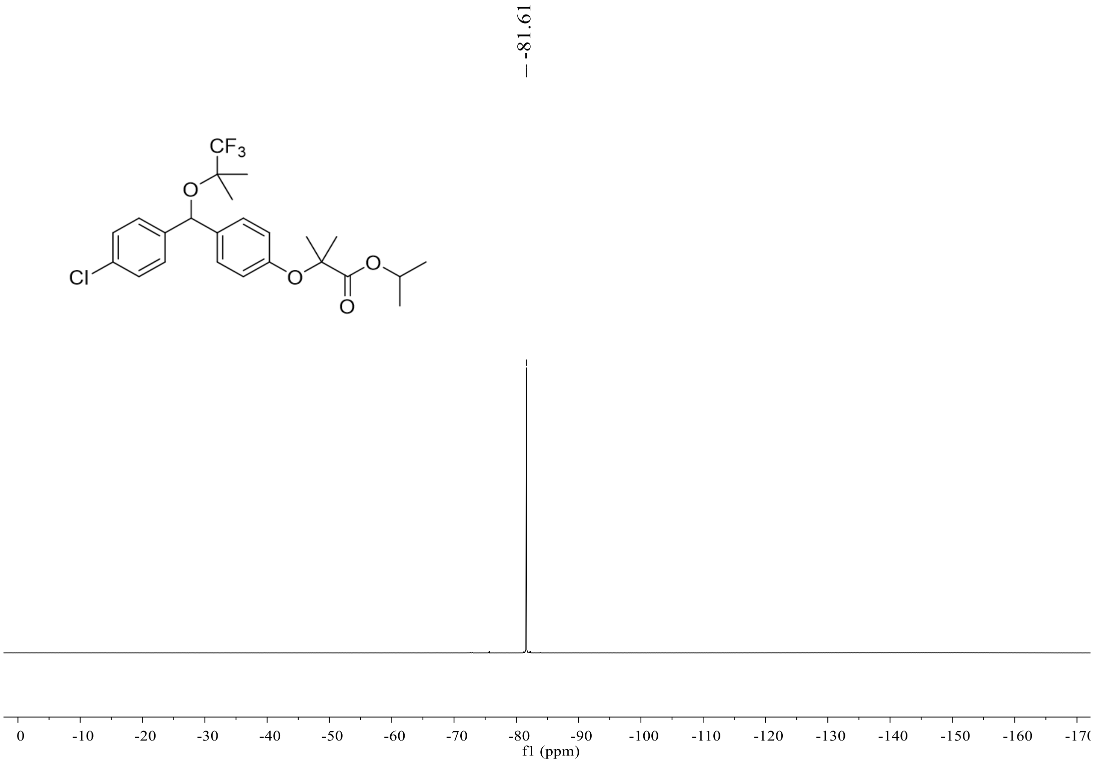

^{19}F NMR spectrum in $CDCl_3$.

123d

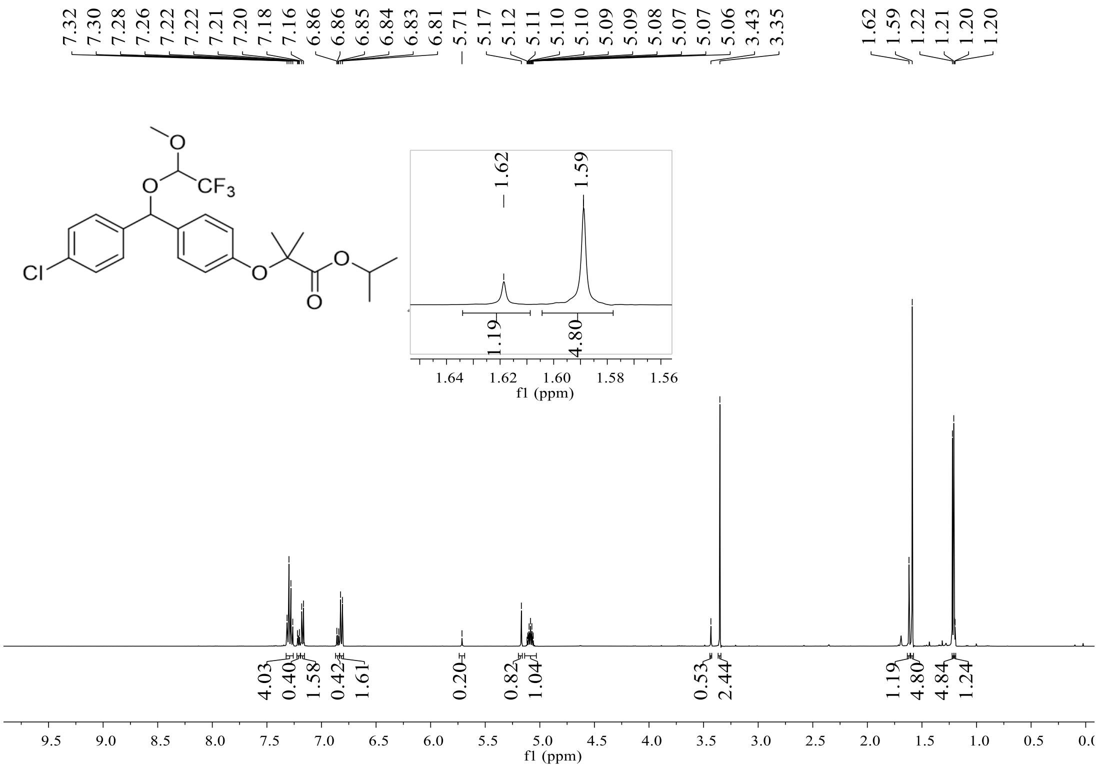

^{1}H NMR spectrum in $CDCl_3$.

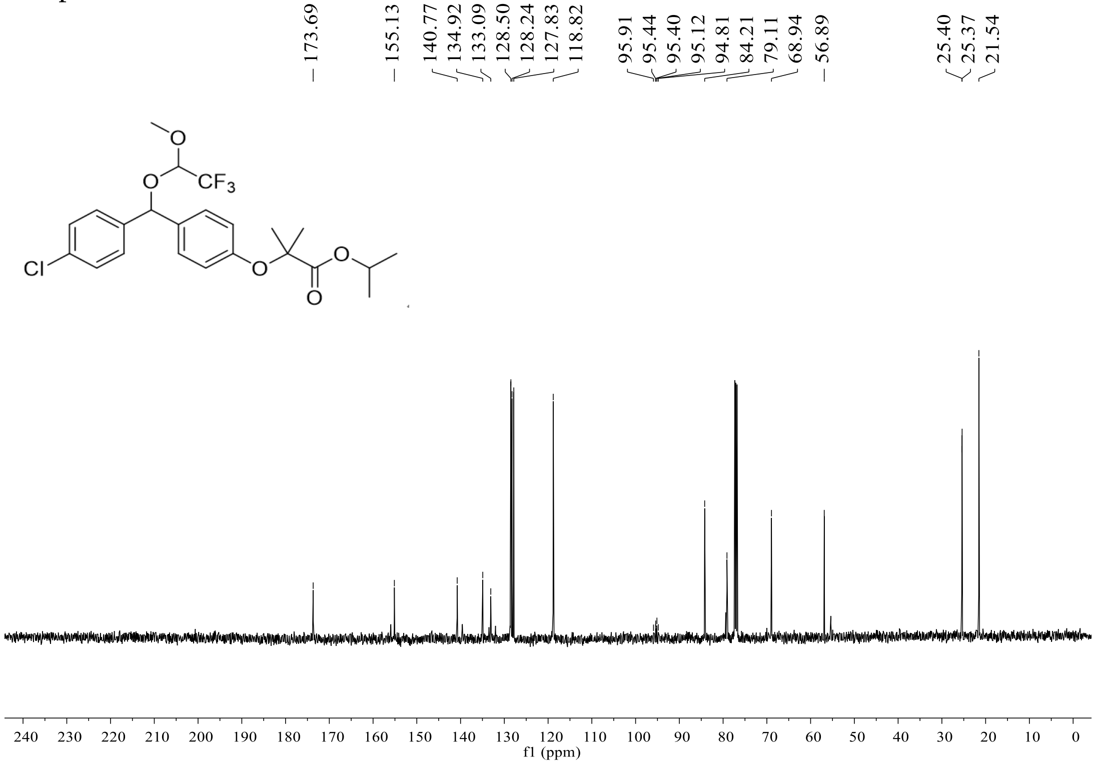

^{13}C NMR spectrum in $CDCl_3$.

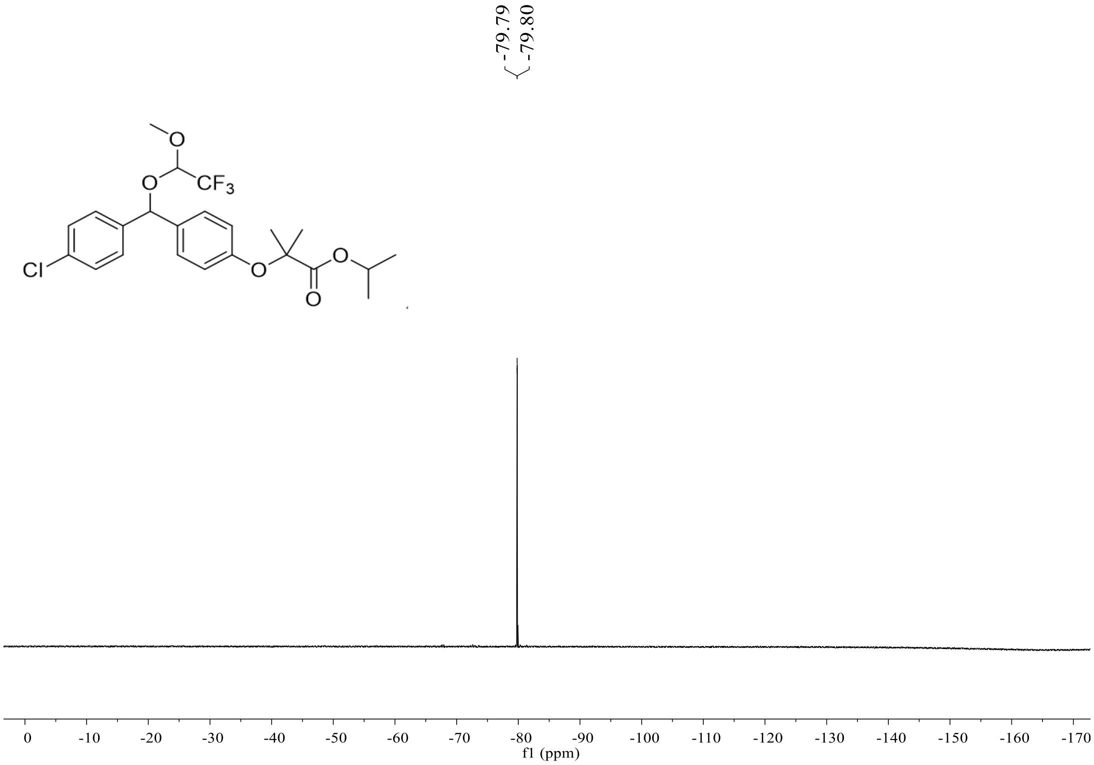

^{19}F NMR spectrum in $CDCl_3$.

124d

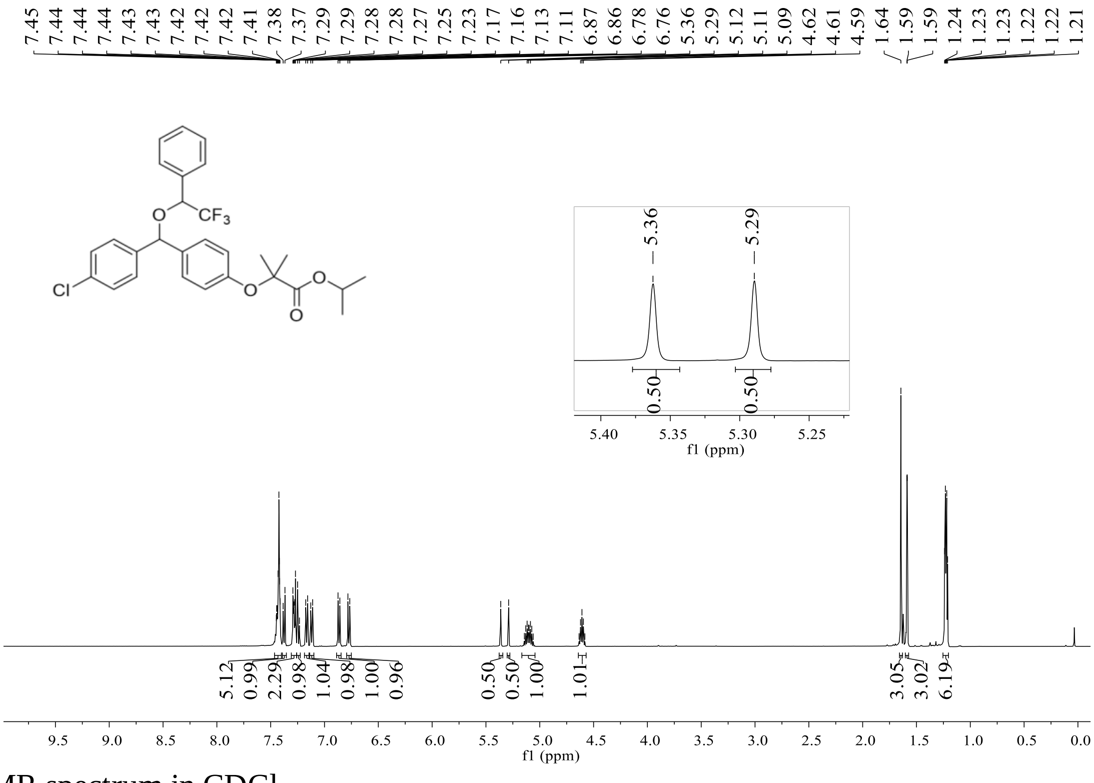

^{1}H NMR spectrum in $CDCl_3$.

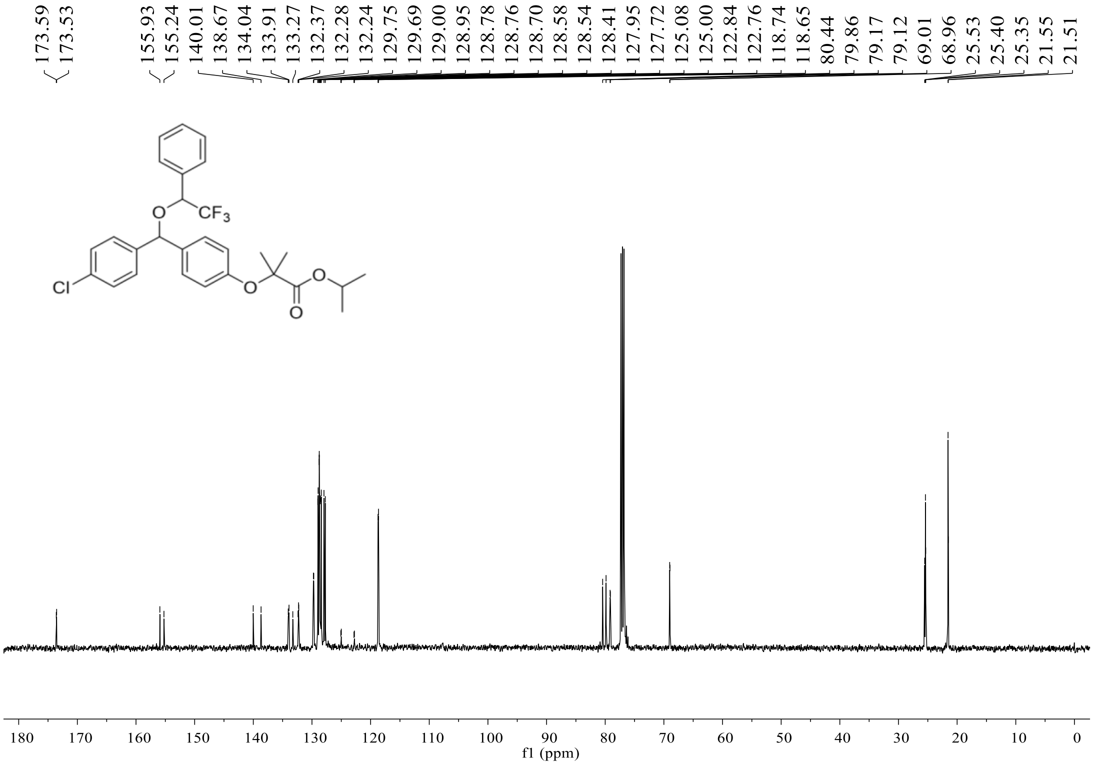

^{13}C NMR spectrum in $CDCl_3$.

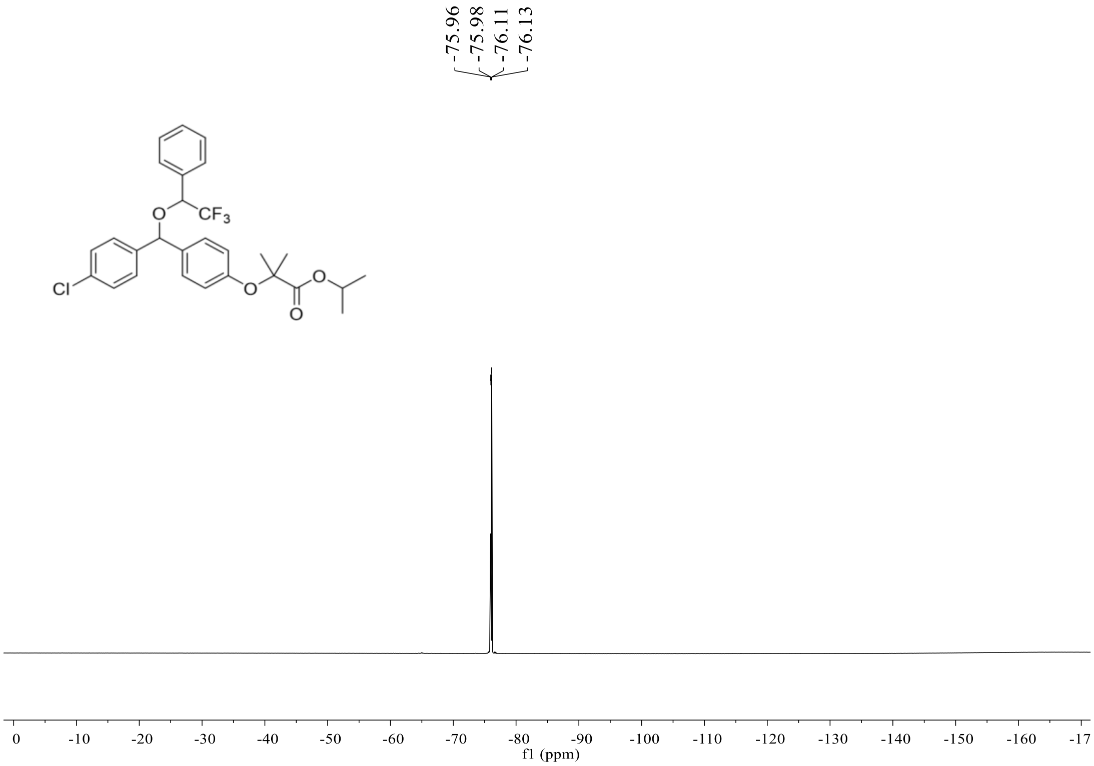

^{19}F NMR spectrum in $CDCl_3$.

125d

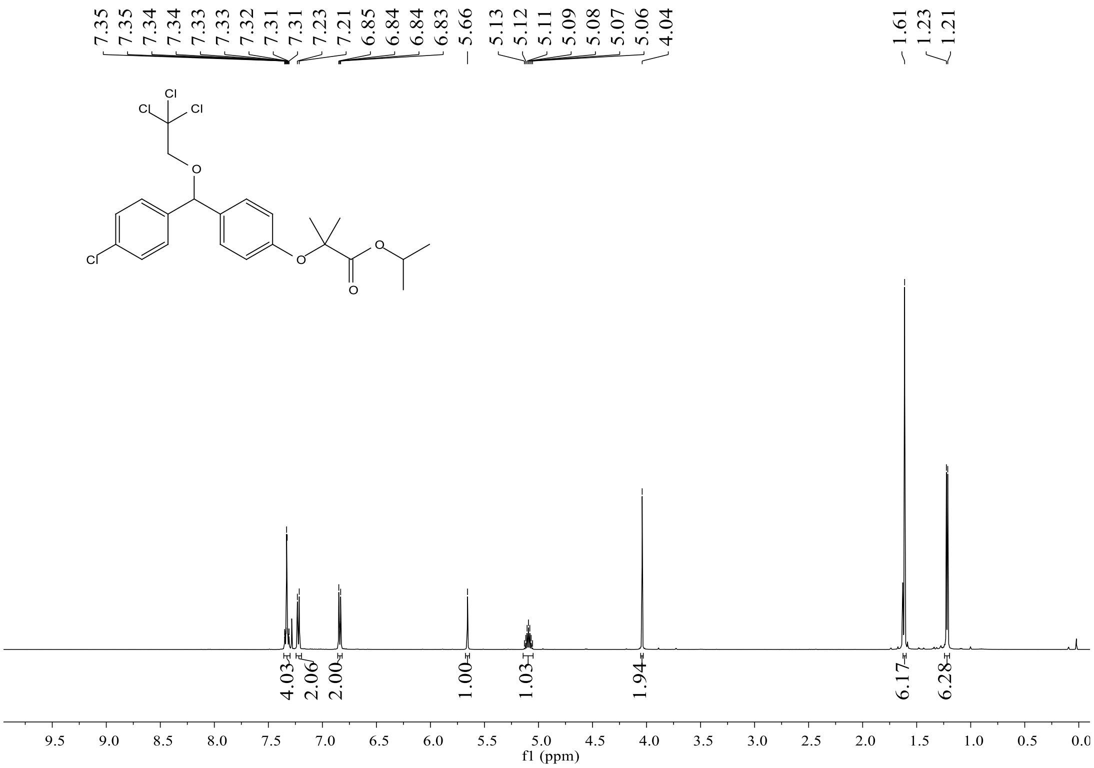

^{1}H NMR spectrum in $CDCl_3$.

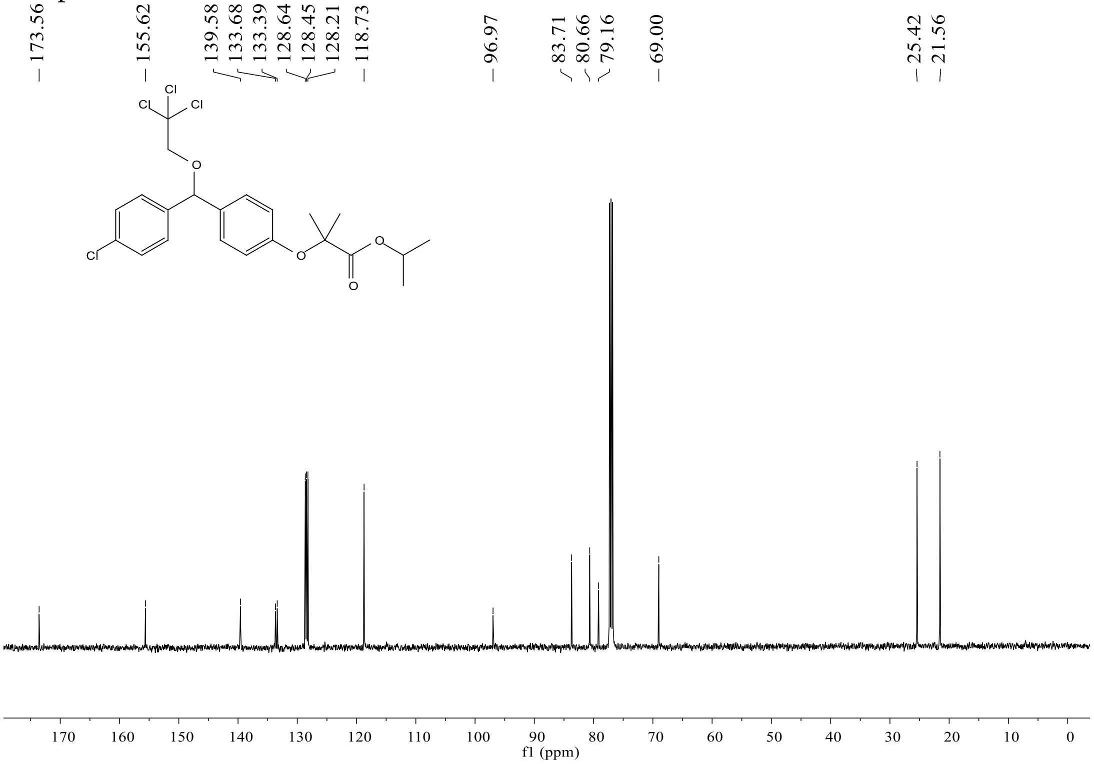

^{13}C NMR spectrum in $CDCl_3$.

132d

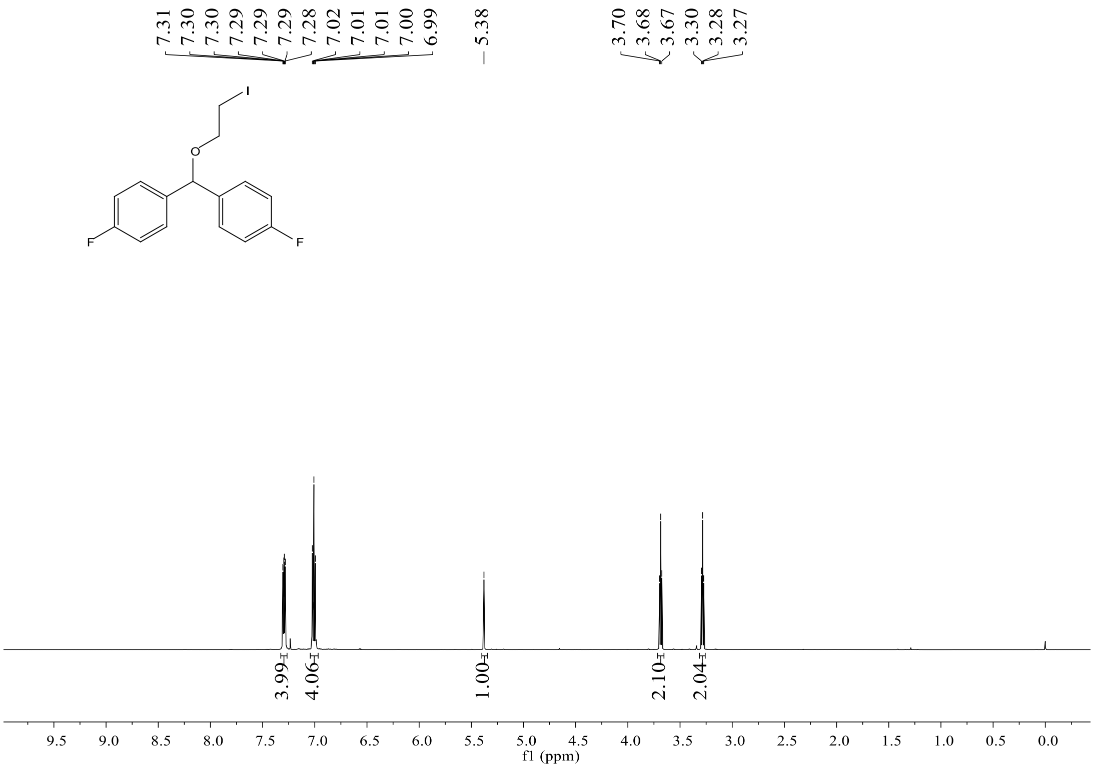

^{1}H NMR spectrum in $CDCl_3$.

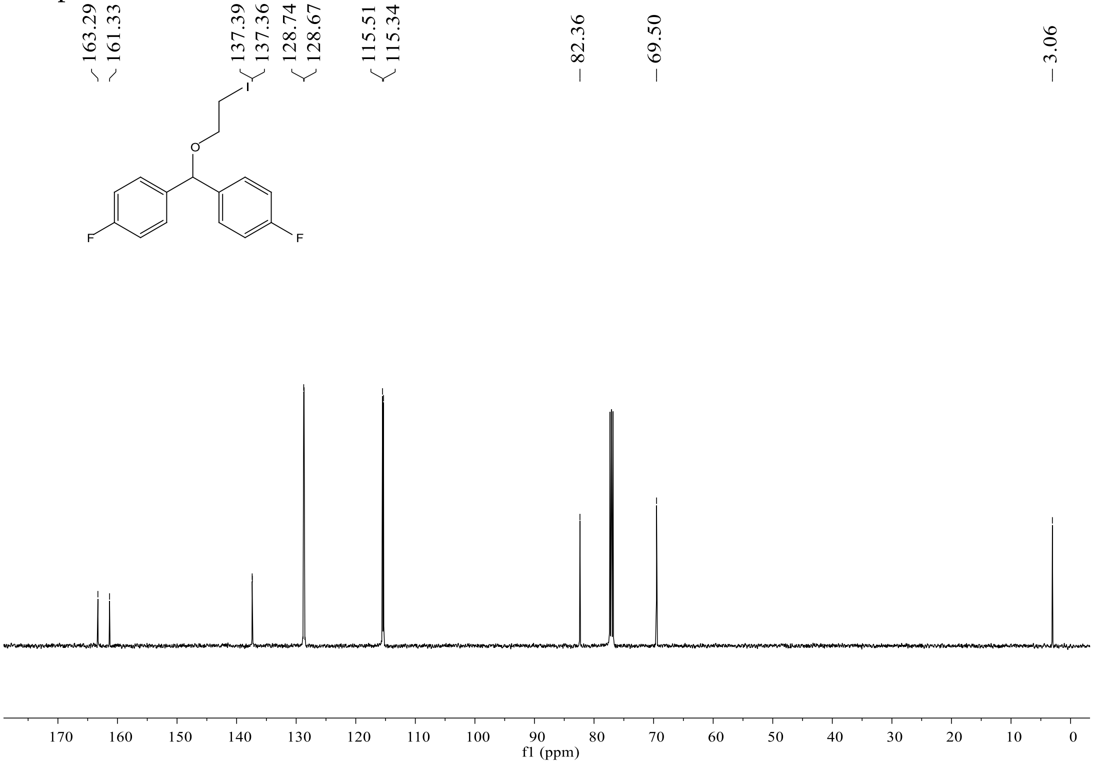

^{13}C NMR spectrum in $CDCl_3$.

126e

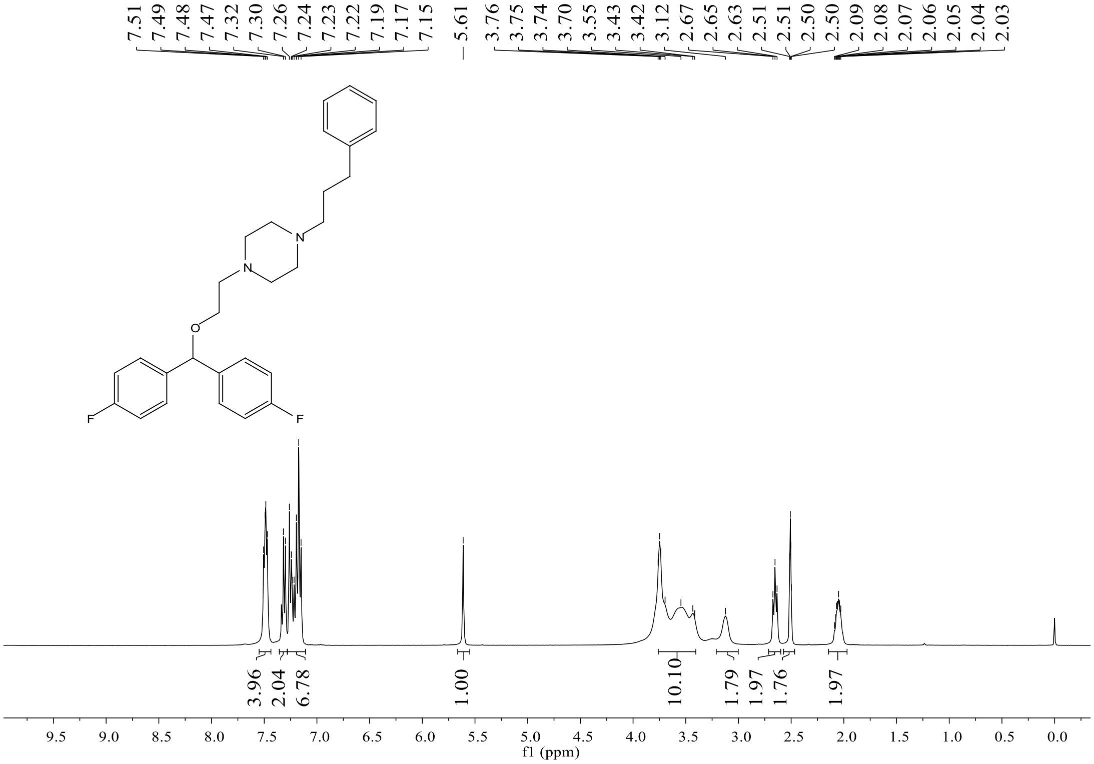

^{1}H NMR spectrum in DMSO-d_6.

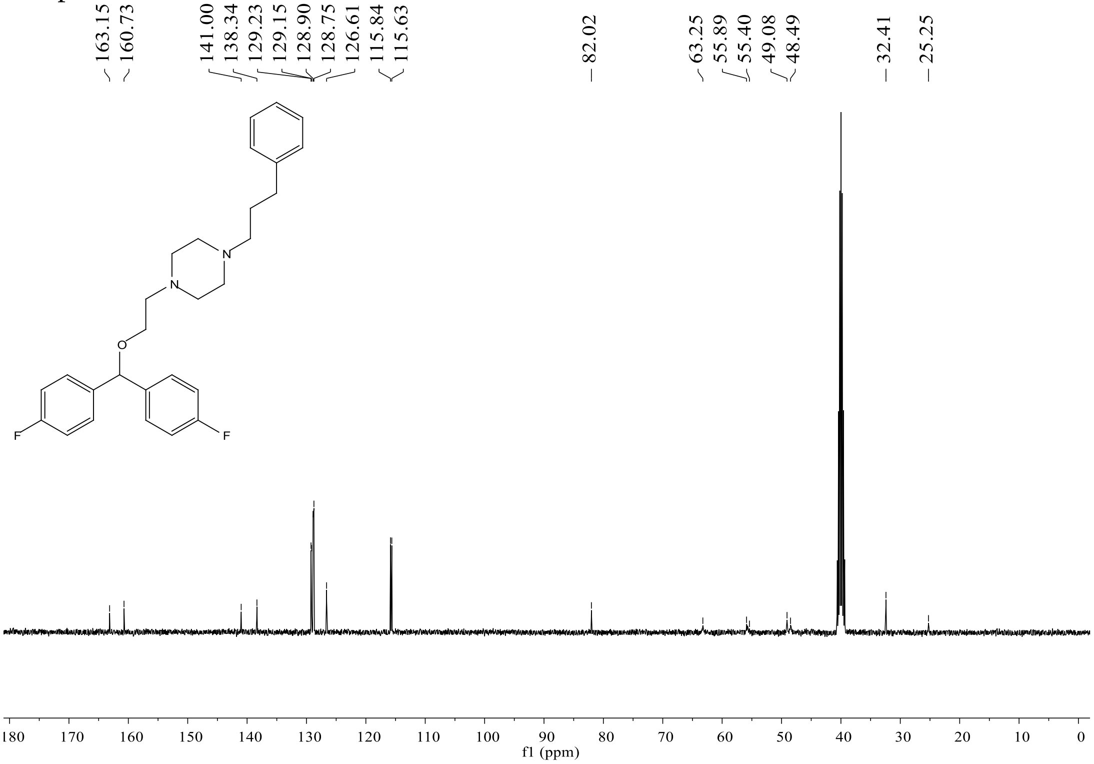

^{13}C NMR spectrum in DMSO-d_6.

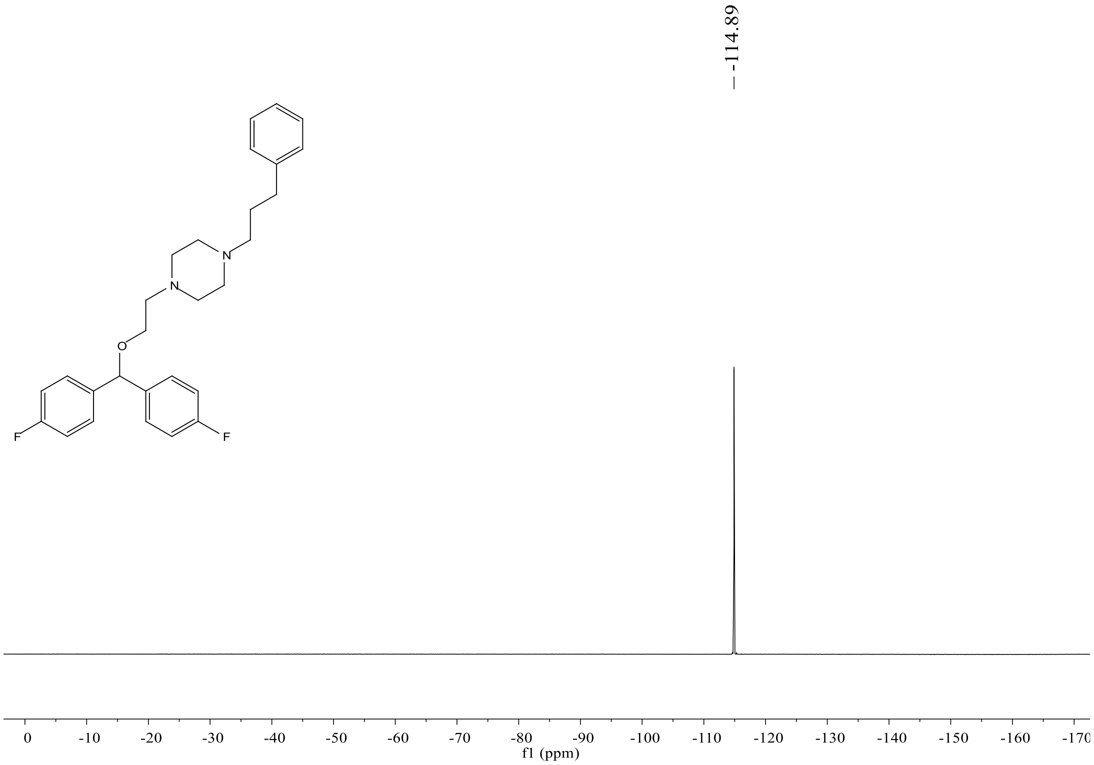

^{19}F NMR spectrum in DMSO-d_6.

10 Partial spectra of synthesized starting material

55b

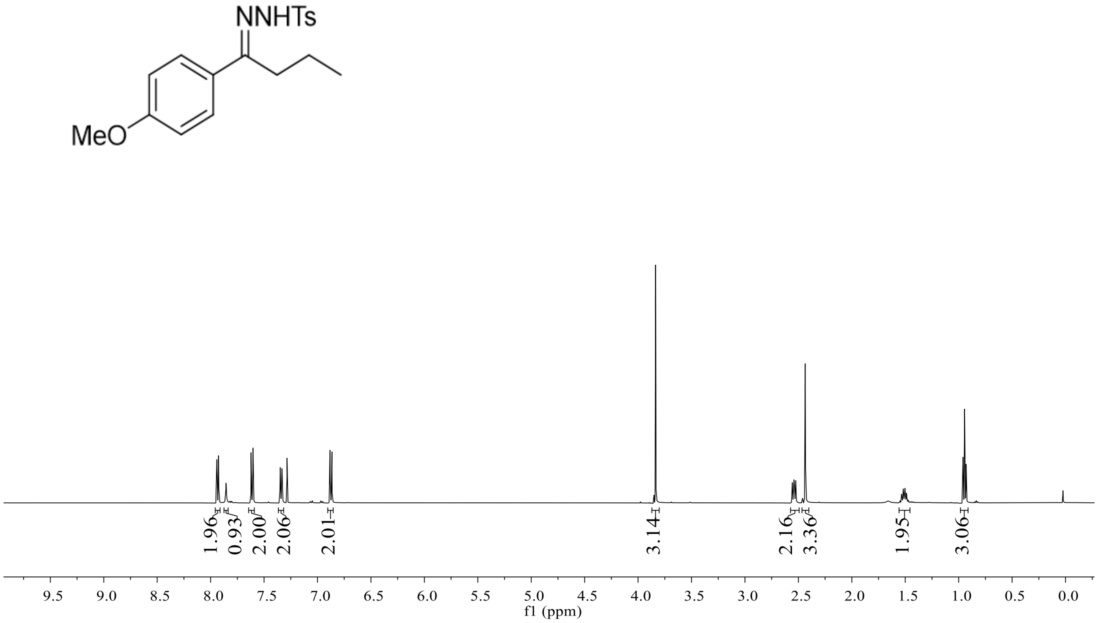

^{1}H NMR spectrum in CDCl3.

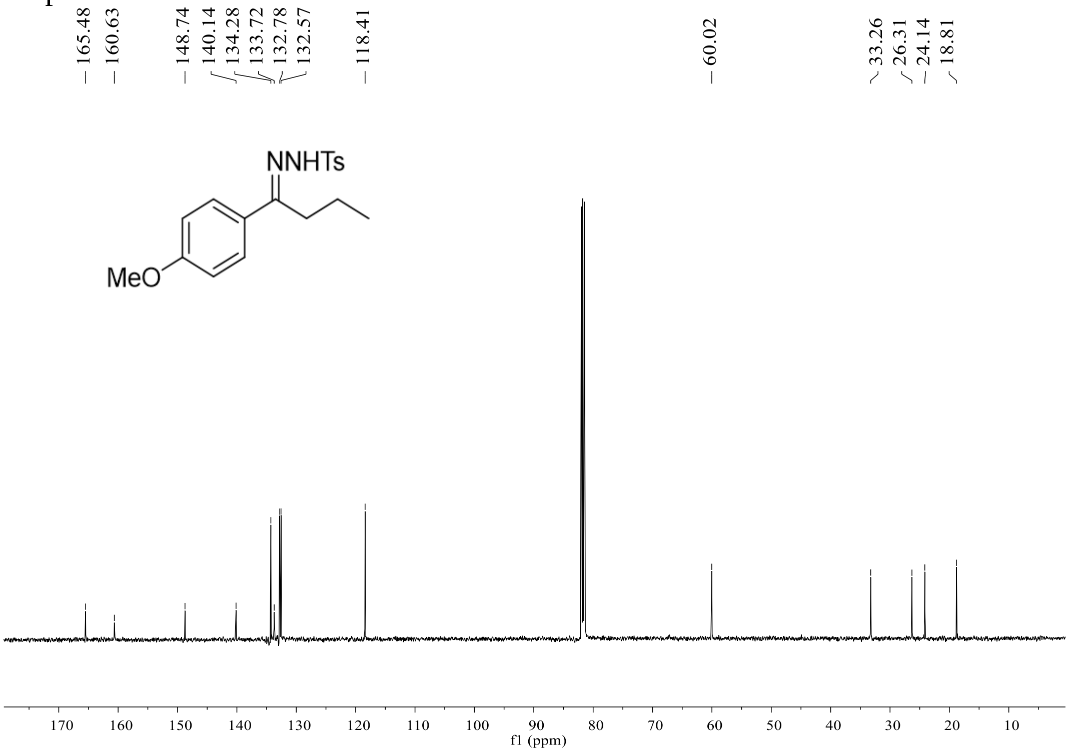

^{13}C NMR spectrum in CDCl3.

83b

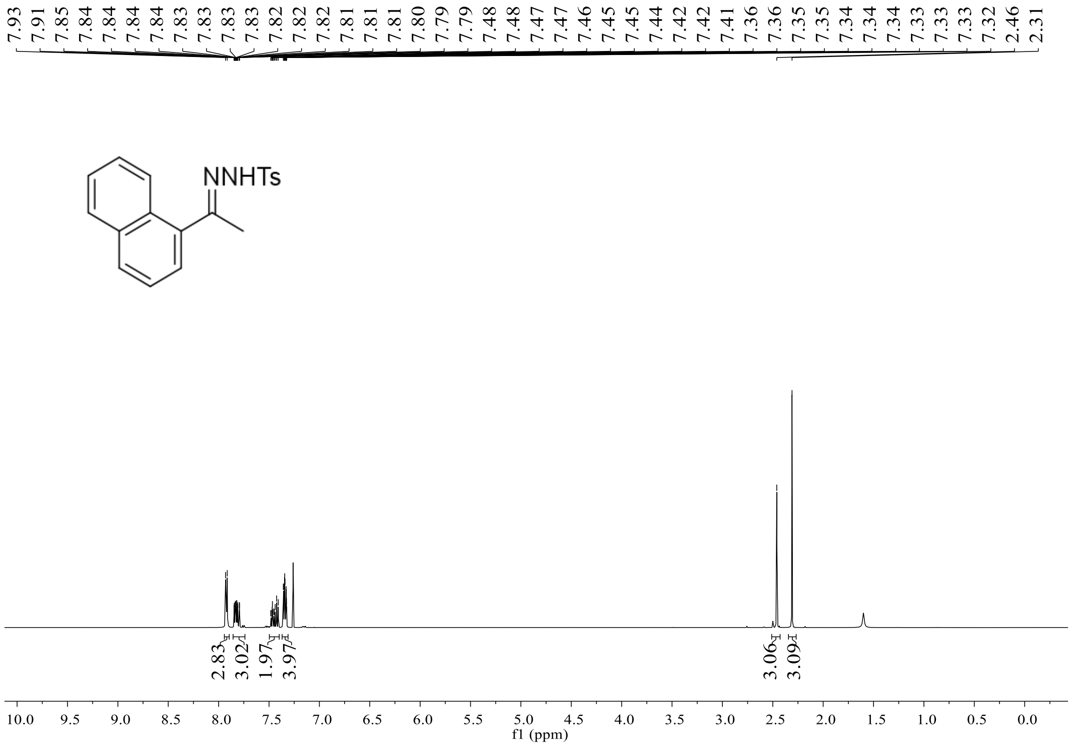

^{1}H NMR spectrum in $CDCl_3$.

92b

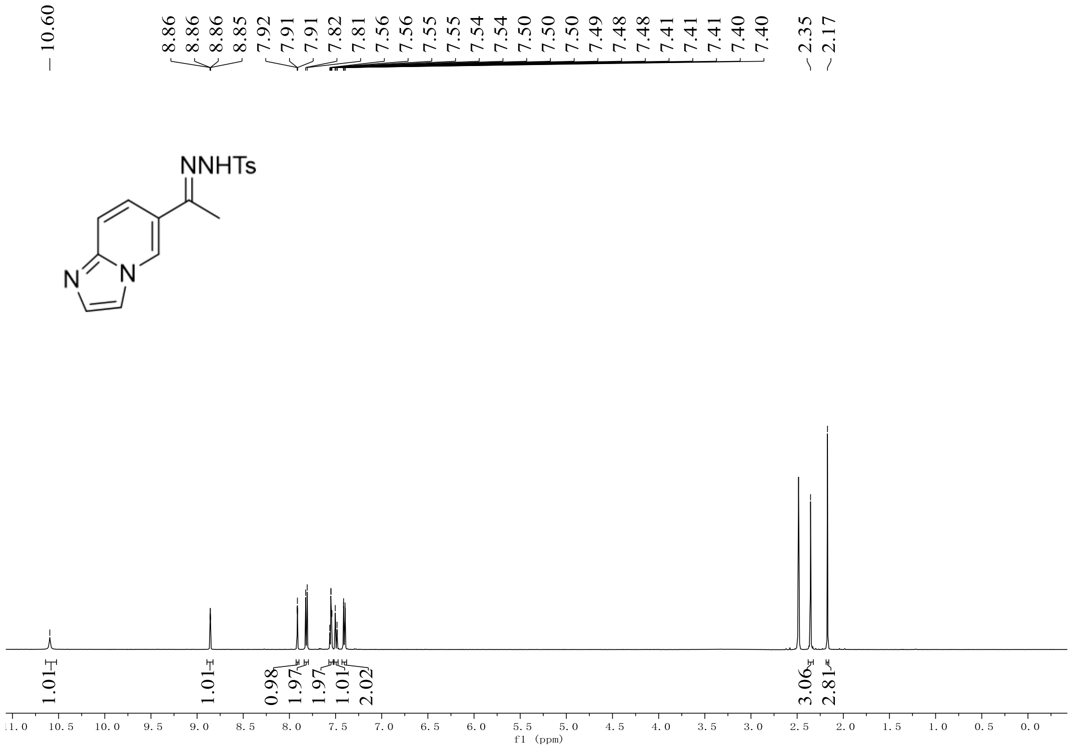

^{1}H NMR spectrum in DMSO-d_6.

126b

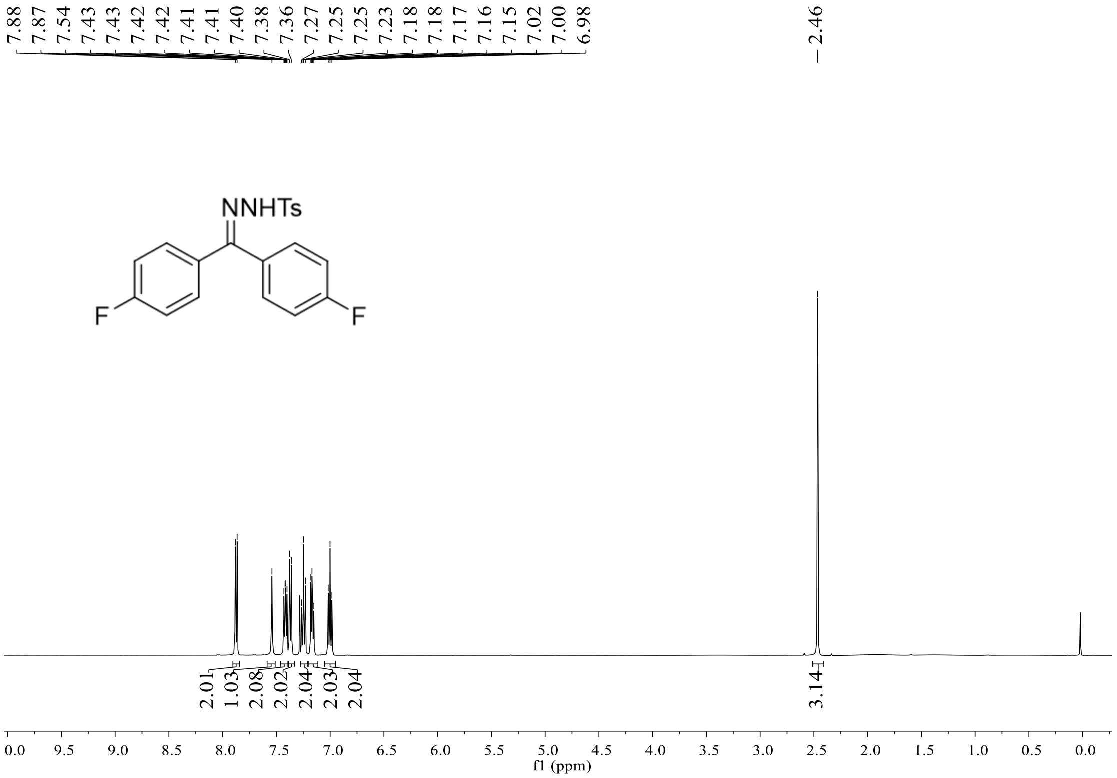

^{1}H NMR spectrum in $CDCl_3$.

11 References

1. Zhang, Y., Han, X., Wu, R., Wang, J., Li, Q., Lin, J., Xia, D., Hong, X., Das, S., Zhang, W. D. Metal-free and visible-light-mediated method enables the synthesis of olefins from ketones. Green. Synth. Catal. doi.org/10.1016/j.gresc.2024.02.001.
2. Su, J., Chen, K., Kang, Q. K., Shi, H. Catalytic SNAr hexafluoroisopropoxylation of aryl chlorides and bromides. Angew. Chem. Int. Ed. 135, e202302908 (2023).
3. Imada, Y., Röckl, J. L., Wiebe, A., Gieshoff, T., Schollmeyer, D., Chiba, K., Franke, R., Waldvogel, S. R. Metal- and Reagent-Free Dehydrogenative Formal Benzyl–Aryl Cross-Coupling by Anodic Activation in 1,1,1,3,3,3-Hexafluoropropan-2-ol. Angew. Chem. Int. Ed. 57, 12136–12140 (2018).
4. Ironside, M. D., Sugathapala, P. M., Robertson, J., Darey, M. C., Zhang, J. Scale-up synthesis of the dopamine uptake inhibitor GBR-12909. Org. Proc. Res. Dev. 6, 621–627 (2002).
5. Gaussian 16, Revision A.03, M. J. Frisch, G. W. Trucks, H. B. Schlegel, G. E. Scuseria, M. A. Robb, J. R. Cheeseman, G. Scalmani, V. Barone, G. A. Petersson, H. Nakatsuji, X. Li, M. Caricato, A. V. Marenich, J. Bloino, B. G. Janesko, R. Gomperts, B. Mennucci, H. P. Hratchian, J. V. Ortiz, A. F. Izmaylov, J. L. Sonnenberg, D. Williams-Young, F. Ding, F. Lipparini, F. Egidi, J. Goings, B. Peng, A. Petrone, T. Henderson, D. Ranasinghe, V. G. Zakrzewski, J. Gao, N. Rega, G. Zheng, W. Liang, M. Hada, M. Ehara, K. Toyota, R. Fukuda, J. Hasegawa, M. Ishida, T. Nakajima, Y. Honda, O. Kitao, H. Nakai, T. Vreven, K. Throssell, J. A. Montgomery, Jr., J. E. Peralta, F. Ogliaro, M. J. Bearpark, J. J. Heyd, E. N. Brothers, K. N. Kudin, V. N. Staroverov, T. A. Keith, R. Kobayashi, J. Normand, K. Raghavachari, A. P. Rendell, J. C. Burant, S. S. Iyengar, J. Tomasi, M. Cossi, J. M. Millam, M. Klene, C. Adamo, R. Cammi, J. W. Ochterski, R. L. Martin, K. Morokuma, O. Farkas, J. B. Foresman, and D. J. Fox, Gaussian, Inc., Wallingford CT, 2016.
6. Zhao, Y., Truhlar, D. G. The M06 suite of density functionals for main group thermochemistry, thermochemical kinetics, noncovalent interactions, excited states, and transition elements: two new functionals and systematic testing of four M06-class functionals and 12 other functionals. Theor Chem Account., 120, 215 (2008).
7. Marenich, A. V., Cramer, C. J., Truhlar, D. G. Universal Solvation Model Based on Solute Electron Density and on a Continuum Model of the Solvent Defined by the Bulk Dielectric Constant and Atomic Surface Tensions. J. Phys. Chem. B., 113, 6378 (2009).
8. Xia, D., Wu, R., Wang, J., Han, X., Li, Y., Li, Q., Luan, X., Hong, X., Y. Zhang, Zhang, W. D. Visible-Light-Mediated Energy Transfer Enables Cyclopropanes Bearing Contiguous All-Carbon Quaternary Centers. ACS Catal, 13, 9806–9816 (2023).
9. Zhang, Y., Li, Y., Ni, S. F., Li, J. P., Xia, D., Han, X., Lin, J., J. Wang, S. Das, Zhang, W. D. Visible-light-induced [3+2] cycloadditions of donor/donor diazo intermediates with alkenes to achieve (spiro)-pyrazolines and pyrazoles. Chem. Sci, 14, 10411–10419 (2023).
10. Zhang, Y., Li, Q., Wang, P., Wang, J., Lin, J., Xia, D., Hao, E., Luan, X., Das, S., Zhang, W. D. A visible-light-promoted metal-free approach for N–H insertions by using donor/donor diazo precursors. Green Chem. 26, 4600–4608 (2024).

11. Lin, J., Zhang, Y., Wang, J., Han, X., Zhu, S., Li, T., Zhu, Y., Zhang, W. D. Visible-light-mediated catalyst-free synthesis of trifluoromethyl (spiro)-epoxides bearing contiguous quaternary centers. Org. Chem. Front., 11, 3080–3088 (2024).
12. Lonka, M. R., Zhang, J., Gogula, T., Zou, H. Copper (i)-catalyzed benzylation of triazolopyridine through direct C–H functionalization. Org. Biomol. Chem., 17, 7455–7460 (2019).
13. Deshmukh, D. S., Gangwar, N., Bhanage, B. M. *N*-Tosylhydrazone as an oxidizing directing group for the redox-neutral access to isoquinolines *via* Cp∗Co(III)-Catalyzed C–H/N–N activation. Journal of the Indian Chemical Society, 98, 100001 (2021).
14. Ye, Q., Huang, W., Wei, L., Cai, M. Recyclable Copper(I)-Catalyzed Cross-Coupling of Trialkylsilylethynes and *N*-Tosylhydrazones Leading to the Formation of C(sp)–C(sp^3) Bonds. The J. Org. Chem., 88, 2973–2984 (2023).
